# ﻿A camouflaged diversity: taxonomic revision of the thorny lacewing subfamily Symphrasinae (Neuroptera, Rhachiberothidae)

**DOI:** 10.3897/zookeys.1199.115442

**Published:** 2024-04-30

**Authors:** Adrian Ardila-Camacho, Renato José Pires Machado, Michael Ohl, Atilano Contreras-Ramos

**Affiliations:** 1 Posgrado en Ciencias Biológicas, Instituto de Biología, UNAM, Depto. de Zoología, Apdo. Postal 70-153, 04510 Ciudad de México, Mexico; 2 Universidad Distrital “Francisco José de Caldas”, Facultad de Ciencias Matemáticas y Naturales, Carrera 4 # 26D-31, Bogotá, Colombia; 3 Universidade Federal do Paraná (UFPR), Departamento de Zoologia, Curitiba, Paraná, Brazil; 4 Museum für Naturkunde Berlin, Leibniz-Institut für Evolutions- und Biodiversitätsforschung, Invalidenstraße 43, 10115 Berlin, Germany; 5 Instituto de Biología, UNAM, Departamento de Zoología, Colección Nacional de Insectos, Apdo. Postal 70-153, 04510 Ciudad de México, Mexico

**Keywords:** Lacewings, Mantispoidea, morphology, New World, systematics

## Abstract

Species of the thorny lacewing subfamily Symphrasinae (Neuroptera: Rhachiberothidae) are revised. Prior to this work, 42 species were known in the genera *Anchieta* Navás, 1909, *Plega* Navás, 1928, and *Trichoscelia* Westwood, 1852. Herein, the number of species is increased to 60, 23 of which are newly described. Species previously known are redescribed, and their taxonomic status is revised. Keys, diagnoses, and high-resolution images for all species are presented. The distribution range of *Anchieta* is now known from Costa Rica to southern Brazil with a total of 11 species, of which three are newly described. The genus *Plega* is known from southwestern United States to southern Brazil and includes 28 species of which 14 are described as new. Moreover, the genus *Trichoscelia* occurs from central and southern Mexico to Argentina, with a total of 21 species, of which six are herein newly described. A phylogenetic analysis of Symphrasinae based on morphological characters recovered the three symphrasine genera as monophyletic, with *Anchieta* sister to *Plega* + *Trichoscelia*. The three genera are newly diagnosed based on a cladistic framework. Within the genus *Anchieta*, bee-mimicking species comprise a monophyletic group, while wasp-mimicking species form a laddered sequence to that lineage. Within *Plega*, three lineages are recovered, the first mostly composed of South and Mesoamerican species, the second with species predominantly from Central America and central and southern Mexico, and a third clade encompassing species mostly from central and northern Mexico and southwestern United States. By contrast, relationships between species of *Trichoscelia* were poorly resolved because of a simplified and conserved morphology of this group.

## ﻿﻿Dedication

We dedicate this work to the memory of neuropterists who eased the way for today’s taxonomy of Symphrasinae. They have made possible our current understanding on this enigmatic group: Robert G. Beard intended to write a revision of American Mantispidae before his death in 1968, and many type specimens in European museums have determination and synonymy labels from him. Norman D. Penny (1946–2016) revised the Symphrasinae from the Amazon Basin and these publications motivated AAC since an undergraduate (2007–2013). Finally, Lionel A. Stange (1935–2020) engaged in revising the fauna of Symphrasinae from North and Central America; however he did not conclude this project as he dedicated strong efforts to Myrmeleontidae through his long career, yet he generously shared many observations, drawings, and materials with AAC one year before his death.

## ﻿﻿Introduction

The superfamily Mantispoidea includes three extant families, i.e., Berothidae, Rhachiberothidae, and Mantispidae, of which the latter two are collectively known as raptorial Mantispoidea or Raptoneuroptera due to the presence of grasping forelegs for prey capture (Engel et al. 2018; Ardila-Camacho et al. 2021a). Understanding of the phylogenetic relationships between these families remain contentious, in part because of a highly diverse morphology, disparate distribution, relatively low diversity, and a long evolutionary history with a considerable number of extinct lineages (Snyman et al. 2020; Lu et al. 2020; Ardila-Camacho et al. 2021a). The traditional scheme of classification of Mantispidae considers four subfamilies, namely Symphrasinae, Drepanicinae, Calomantispinae, and Mantispidae, of which the first has been considered as sister of the latter three for long time and often referred to as “the most primitive” subfamily of mantidflies (Penny and da Costa 1983; Lambkin 1986a, b; Liu et al. 2015; Shi et al. 2019). Moreover, the Rhachiberothidae, which was originally proposed as a subfamily of Berothidae, includes a single extant subfamily, i.e., the Rhachiberothinae, plus the extinct Paraberothinae from the Cretaceous. This group has been also considered as a subfamily of Mantispidae, sister to the rest of mantispid subfamilies (Willmann 1990), or a separated family, either sister to Berothidae (Aspöck and Mansell 1994; Song et al. 2019) or sister to Mantispidae (Liu et al. 2015; Wang et al. 2017).

In a recent phylogenomic study of Neuropterida, previous hypotheses of the relationships between the Mantispoid subfamilies were challenged, as the Mantispidae was recovered as a polyphyletic group, with Symphrasinae and Rhachiberothinae as sister groups in a clade sister to Berothidae and the remainder Mantispidae subfamilies (Winterton et al. 2018). In connection with these results, consistent morphological support for a new concept of Rhachiberothidae, including Symphrasinae was proposed by Ardila-Camacho et al. (2021a). This study recovered the Rhachiberothidae as a monophyletic group with Symphrasinae sister to the extinct Paraberothinae in a clade, which is sister to the Rhachiberothinae based on the presence of modified setae on the closing surface of the forebasitarsus and the presence of forebasitarsal Stitz organ. Also, this family was recovered as sister to the true Mantispidae (i.e., Mantispinae + (Calomantispinae + Drepanicinae)) whose monophyly is strongly supported by many characters of the raptorial apparatus, wing venation, and genitalia. The hypothesis of the sister-group relationship between these families assumes a single origin of the raptorial condition within the superfamily, and the monophyly of each subfamily and their relationships itself are supported by characters of the raptorial apparatus, primarily of the foretarsus (Ardila-Camacho et al. 2021a).

The subfamily Symphrasinae was proposed by Lambkin (1986a), although it was originally created by Navás (1909) as a tribe of Mantispidae, i.e., the Symphrasini. Since then, this group has undergone numerous nomenclatural changes due to a considerable homonymy and synonymy of its genera (Penny 1982b). Currently this group includes three extant genera restricted to the New World, namely, *Anchieta* Navás, 1909 with, previous to the current revision, eight valid species ranging from Panama to southern Brazil, *Plega* Navás, 1928 composed of 17 valid species distributed from southwestern United States to southern Brazil, and *Trichoscelia* Westwood, 1852 with 17 valid species known from southern Mexico to Argentina (Ardila-Camacho et al. 2018; Machado 2018; Ardila-Camacho et al. 2019). Nevertheless, the subfamily has a relatively rich fossil record, with six genera, i.e., *Archaeosymphrasis*Shi et al. 2019, *Habrosymphrasis*Shi et al. 2019, *Parasymphrasites*Lu et al. 2020, *Haplosymphrasites*Lu et al. 2020, *Parvosymphrasites*Li et al. 2023 and *Proplega*Li et al. 2023 from the Upper Cretaceous deposits of Myanmar (Shi et al. 2019; Lu et al. 2020; Li et al. 2023). Among these, the first three were proposed as stem-group representatives of the Symphrasinae (Shi et al. 2019; Lu et al. 2020). On the other hand, *Haplosymphrasites* and *Symphrasites* Wedmann & Makarkin, 2007 from the Middle Eocene of Germany are considered the crown-group of Symphrasinae together the extant genera (Wedmann and Makarkin 2007; Shi et al. 2019; Lu et al. 2020).

The type genus of this subfamily, i.e., *Symphrasis* was proposed by Hagen (1877) to accommodate two species: *S.signata* and *S.myrapetrella* (Westwood, 1867), although according to Enderlein (1910), the type species of the genus is actually *Raphidiavaria* Walker, 1853. The genus *Plega* was synonymized with *Symphrasis* by Tjeder (1959), although Parker and Stange (1965) proposed *Symphrasis* as a synonym of *Trichoscelia* (Penny 1982b) and reestablished *Plega* after finding consistent differences between both genera. Moreover, *Trichoscelia* and *Symphrasis* were synonymized with *Anisoptera* Schneider by Gerstaecker (1888), with the synonymy of the former genus confirmed by Banks (1913). Nonetheless, *Anisoptera* is not an available name, and its type species belongs to *Anchieta*. The latter genus was synonymized with *Anisoptera* by Enderlein (1910), and this synonymy was confirmed by Banks (1913) and Penny (1982b). Meanwhile, the generic names *Platymantispa* Rehn, 1939 and *Anisopterana* Strand, 1942 were unnecessarily proposed and independently created, because *Anisoptera* is a junior homonym. Considering this background of confusing nomenclatural changes, the oldest available name for the subfamily, i.e., Symphrasini prevailed, because according to the article 40 of the International Code of Zoological Nomenclature, the invalidity of its type genus was not recorded before 1961 (Parker and Stange 1965; Lambkin 1986a).

The phylogenetic relationships of the genera in the Symphrasinae are not well understood, and the boundaries of the genera are not clearly outlined (Liu et al. 2015). The first hypothesis of relationships between the symphrasine genera recovered *Trichoscelia* sister to *Anchieta* + *Plega* (Penny and da Costa 1983), which was also recovered by Willmann (1990) and Liu et al. (2015) based on morphology and total evidence analyses, respectively. Further morphological studies including extant and extinct genera recovered *Archaeosymphrasis*, *Habrosymphrasis* and *Symphrasites* as transitional genera between Mesomantispinae and the extant crown-group of Symphrasinae, where *Trichoscelia* was recovered as sister of the other two genera (Shi et al. 2019). In a subsequent research, Shi et al. (2020) presented hypotheses from different morphological datasets, mostly without clear relationships between extinct and extant genera, and between extant genera. Such work recovered *Archaeosymphrasis* and *Habrosymphrasis* in a clade sister to *Plega* and a trichotomy of *Symphrasites*, *Anchieta*, and *Trichoscelia* from a matrix, where the genitalia characters were excluded. Additionally, from the same dataset excluding genitalia characters and poorly preserved fossils, *Plega* was recovered as sister to *Anchieta* + *Trichoscelia* (Shi et al. 2020). Nearly simultaneously, Lu et al. (2020), recovered *Parasymphrasites* and *Habrosymphrasis* as stem group Symphrasinae, and *Symphrasites* as sister of a polytomy between the extant genera and *Haplosymphrasites*.

Besides the contentious background on the understanding of the phylogenetic relationships between the symphrasine genera, the extant diversity of the group has never been previously revised, except for the fauna from Amazonia and the United States (Rehn 1939b; Penny 1982a; Penny and da Costa 1983). Nevertheless, morphological descriptions for many species lack enough details and the taxonomic status of the majority of them needs to be clarified. Consequently, a reliable taxonomic identification of the species is often difficult to achieve, particularly in northern South America, Mexico and Central America, where the group has its higher diversity. For these reasons, the objectives of this study are to test the monophyly of the extant genera in the Symphrasinae and to infer their phylogenetic relationships based on morphological data. Additionally, we reconstruct the phylogenetic relationships between the species in the Symphrasinae and provide diagnosis and descriptions for all of them, clarifying their taxonomic status.

## ﻿﻿Materials and methods

We examined and identified > 3000 specimens of Symphrasinae deposited in collections and museums of Europe, United States, Mexico, Costa Rica, Colombia, and Brazil (see collections section below). Nearly all the primary types of the subfamily were directly examined and redescribed, and for the majority high resolution photographs were obtained.

**Figure 1.**
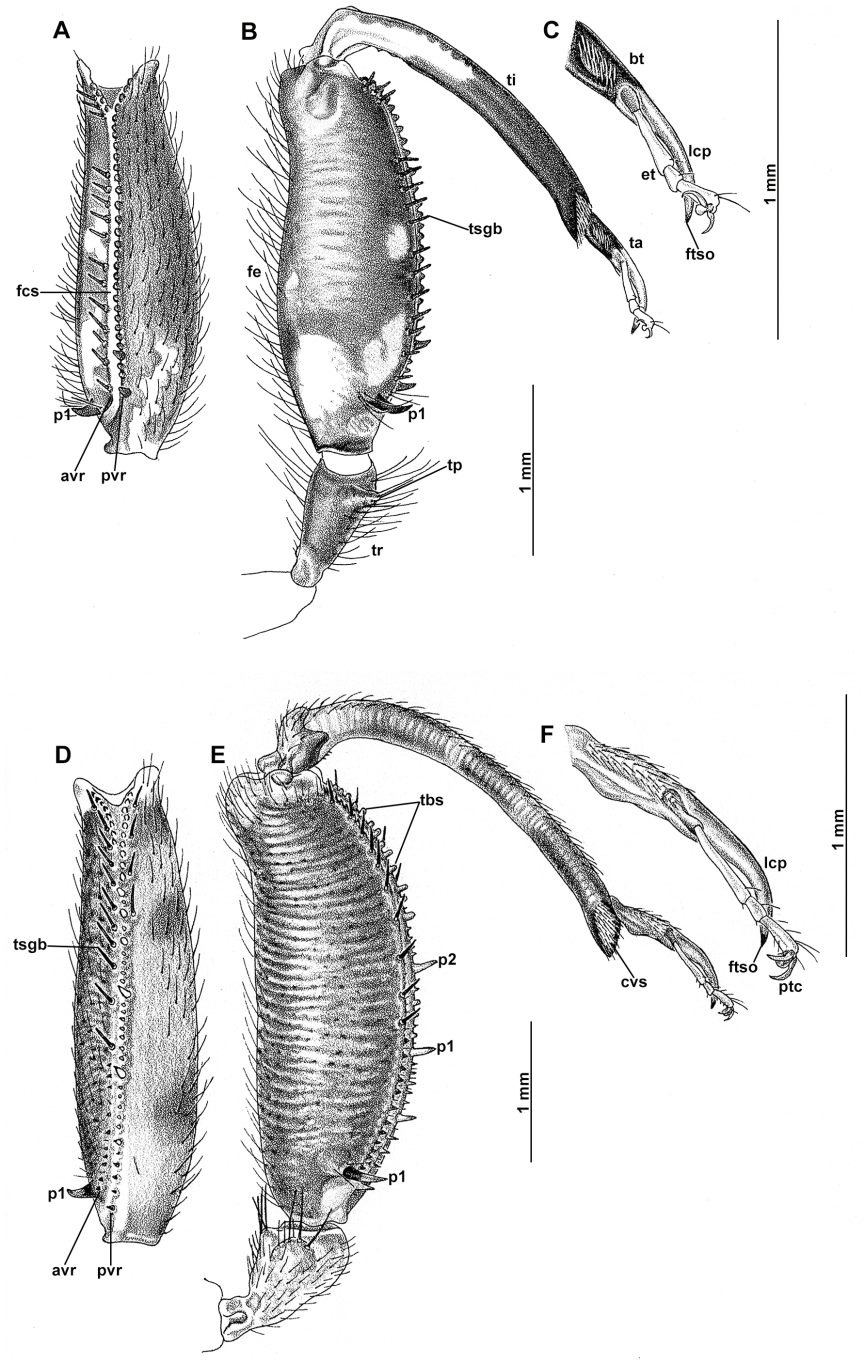
Foreleg morphology of Symphrasinae**A–C***Anchietafumosellus* (Westwood, 1867) **A** forefemur, ventral Surface **B** trochanter, femur, tibia, and tarsus, anterior surface **C** fore- tarsus and pretarsus, anterior surface **D–F***Plegadactylota* Rehn, 1939 **D** forefemur, ventral Surface **E** trochanter, femur, tibia, and tarsus, anterior surface **F** fore- tarsus and pretarsus, anterior surface. Abbreviations: avr, anteroventral processes row bt, basitarsus cvs, clavate setae et, eutarsus fe, femur fcs, femoral closing surface ftso, foretarsal Stitz organ lcp, lanceolate process p, primary process ptc, pretarsal claw pvr, posteroventral processes row ta, tarsus ti, tibia tp, trochanter process tr, trochanter tbs, tubercle-shaped specialization tsgb, thickened seta with globular base.

**Figure 2.**
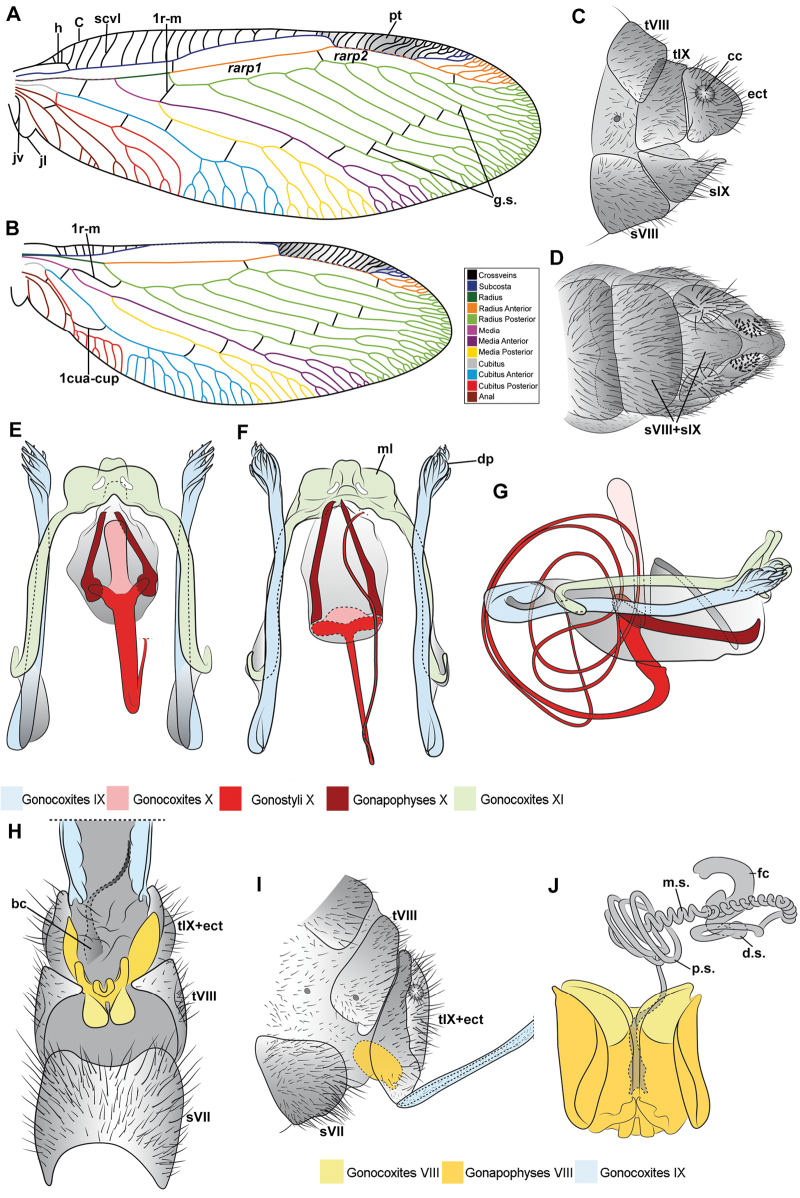
Wing venation and genitalic morphology of *Plegadactylota* Rehn, 1939 as an example for Symphrasinae**A** forewing **B** hind wing **C** male terminalia, lateral **D** same, ventral **E** male genitalia, dorsal **F** same, ventral **G** same, lateral **H** female terminalia, ventral **I** same, lateral **J** female genitalia, ventral. Abbreviations: h, humeral vein C, Costa scvl, subcostal veinlets 1r-m, first radiomedial crossvein *rarp1*, first radial anterior cell *rarp2*, second anterior radial cell pt, pterostigma g.s., gradate series jv, jugal vein jl, jugal lobe 1cua-cup, first intracubital crossvein tVIII, eighth tergite, tIX, ninth tergite cc, callus cercus ect, ectoproct sVII, seventh sternite sVIII, eighth sternite sIX, ninth sternite ml, median lobe of gonocoxites XI dp, digitiform processes bc, bursa copulatrix p.s., proximal section of spermatheca m.s., medial section of spermatheca d.s., distal section of spermatheca fc, fertilization canal.

Genitalia preparations were made by clearing the last abdominal segments in a hot solution of 10% potassium hydroxide (KOH). Residuals of the alkaline solution, gut contents, and tracheae were washed with a series of washings of distilled water and 80% ethyl alcohol, sometimes with the aid of a tuberculin syringe, a minute steel hook inserted at the tip of a wood stick, and fine forceps with blunt tip. A cut along the pleural membrane allowed extraction of the male genital complex for examination on an excavated microscope slide (Ardila-Camacho et al. 2019). Genital complexes were placed in a setup composed of a petri dish with a layer of paraffin at the bottom and filled with alcohol. The genitalia were situated at different views in a canal made with the tip of entomological forceps. Genital structures were placed in microvials filled with glycerin for permanent storage. External morphology was studied in a Zeiss Discovery V8 stereomicroscope. High resolution photographs were taken with a ZEISS AxioZoom V16 microscope, and then stacked using the ZENpro201 software. Images were processed using Adobe Photoshop CS6. Morphological terminology and homology followed Ardila-Camacho et al. (2021a), the terms for foreleg morphology are summarized in Fig. 1, and for wing venation and male and female genitalia are depicted in Fig. 2.

### ﻿﻿Museums and collections cited across the manuscript

**AMNH**American Museum of Natural History, New York, USA.

**ANSP** Academy of Natural Sciences, Philadelphia, Pennsylvania, USA

**CAS** California Academy of Sciences, San Francisco, USA.

**CAUD** Colección de Artrópodos, Universidad Distrital Francisco José de Caldas, Bogotá, Colombia.

**CN** Navás collection housed at (MZBS) Museo Zoologia, Barcelona, Spain.

**CNIN** Colección Nacional de Insectos, Instituto de Biología, Universidad Nacional Autónoma de México, Ciudad de México, México.

**CSCA** California State collection of Arthropods, California Department of Food and Agriculture, Sacramento, California, USA.

**CZMA** Coleção Zoológica do Maranhão, Caixias, Maranhão, Brazil.

**DZUP** Coleção Entomológica Padre Jesus Santiago Moure, Curitiba, Paraná, Brazil.

**FSCA** Florida State Collection of Arthropods, Gainesville, Florida, USA.

**IAvH** Instituto Alexander von Humbolt, Villa de Leyva, Colombia.

**ICN** Instituto Nacional de Ciencias Naturales, Universidad Nacional de Colombia, Bogotá, Colombia.

**INPA** Coleção Sistematica da Entomologia, Manaus, Amazonas, Brazil.

**LEUA** Colección de Entomología del Laboratorio de Entomología, Universidad de la Amazonia, Florencia, Caquetá, Colombia.

**MCZ** Museum of Comparative Zoology, Cambridge, Massachusetts, USA.

**MEFLG** Museo Entomológico “Francisco Luis Gallego”, Universidad Nacional de Colombia, Medellín, Colombia.

**MHNL** Musée des Confluences, Lyon, France.

**MHN-UPN** Museo de Historia Natural, Universidad Pedagógica Nacional, Bogotá, Colombia.

**MNCR** Museo Nacional de Costa Rica, Santo Domingo de Heredia, Costa Rica.

**MNHN** Museum national d’Histoire naturelle, Paris, France.

**MPEG** Museu Paraense Emilio Goeldi, Belem, Para, Brazil.

**MPUJ** Museo Javeriano de Ciencias Naturales, Bogotá, Colombia.

**MUSENUV** Museo Entomológico de la Universidad del Valle, Cali, Colombia.

**MZPW** Museum of the Institute of Zoology, Polish Academy of Sciences, Warszawa, Poland.

**NHMUK** The Natural History Museum, London, United Kingdom.

**NHRS** Natuhistoriska Riksmuseet, Stockholm, Sweden.

**NMBS** Naturhistorisches Museum, Bern, Switzerland.

**NMNH** National Museum of Natural History, Smithsonian Institution, Washington DC, USA.

**OUMNH** Oxford University Museum of Natural History, Oxford, Great Britain.

**SDEI**Senckenberg Deutsches Entomologisches Institut, Eberswalde, Germany (formerly in Berlin-Dahlem).

**TAMUIC** Insect collection of Texas A&M University, Texas, USA.

**UAAM** University of Arkansas Arthropod Museum, Fayetteville, Arkansas, USA.

**UCD** Bohart Museum of Entomology, University of California, Davis, California, USA.

**UFMG** Universidade Federal de Minas Gerais, Belo Horizonte, Brazil.

**UNAB** Museo de la Facultad de Agronomía Universidad Nacional de Colombia, Bogotá, Colombia.

**UMSP** Entomology collection of the University of Minnesota, St. Paul, Minnesota, USA.

**ZIN** Zoological Museum, Academy of Sciences, St. Petersburg, Russia.

**ZIMG** Ernst-Moritz-Arndt-Universität, Greifswald, Germany.

**ZMB** Museum für Naturkunde, Berlin, Germany.

### ﻿﻿Phylogenetic analysis

For the phylogenetic analysis of Symphrasinae, 10 species of *Anchieta*, 25 of *Plega*, and 19 of *Trichoscelia* studied herein were included. One species of Mantispidae, i.e., *Gerstaeckerellairrorata* (Erichson, 1839), and one of Rhachiberothinae, i.e., *Mucroberothavesicaria* Tjeder, 1968 were selected as outgroups.

The morphological characters used for the phylogenetic analysis were determined by comparing the majority of species of Symphrasinae described herein with the outgroups, plus the detailed comparative study of the raptorial Mantispoidea by Ardila-Camacho et al. (2021a). The definition of the characters and character states was based on Strong and Lipscomb (1999). A matrix with 102 adult characters and 56 terminals was handled using the software WinClada® (Nixon 2002) and exported as a nexus file to perform the phylogenetic analysis under the method of parsimony with the program TNT^®^ 1.5 (Goloboff and Catalano 2016). The morphological matrix was analyzed using New Technology search set to 50 employing algorithms of sectorial search, ratchet, drift, and tree fusing, finding the minimum tree 40 times. The characters were treated as unordered and with equal weight. For comparison purposes, an analysis of traditional search of TBR (Tree Bisection and Reconnection) based on 100 random addition sequences, plus an analysis of New Technology with implied weighting with a value of the constant k of 10.717 calculated by means of the TNT script “setk.run” (Goloboff et al. 2008) were conducted. Strict consensus of the most parsimonious trees, as well as optimization and analysis of the evolution of the characters were made using WinClada.

The Bremer absolute (Bremer 1994) was used to calculate support of the branches. This index calculates the number of extra steps needed for a consensus branch to collapse. The Bremer support were calculated using suboptimal trees of 1–10 steps with TBR on the software TNT.

The final topology, including all characters, states and Bremer values, was edited using Adobe Illustrator CS® software.

### ﻿﻿Morphological characters employed to infer the phylogenetic relationships of the genera and species of Symphrasinae

**Head**

1. ***Ratio of height of vertexal region above compound eyes to distance between upper limit of compound eyes and frontoclypeal ridge*** : (0) 1:1; (1) 0.5:1; (2) 0.3:1 or 0.2:1.
2. ***Overall shape of head, frontal view*** : (0) globular; (1) ovoid; (2) quadrangular; (3) diamond-shaped.
3. ***Shape of frontal sclerite (frons), frontal view*** : (0) subquadrate; (1) pentagonal; (2) rectangular; (3) bar-shaped.
4. ***Ratio of the compound eye width to interocular distance at toruli level, frontal view*** : (0) ~ 0.75:1; (1) 0.5:1; (2) <0.5:1.
5. ***Ocular plane morphology*** : (0) anteriorly curved inwards; (1) concave; (2) straight.
6. ***Antennal scape*** : (0) as long as wide; (1) 1.5 × as long as wide; (2) ≥2.0× as long as wide.
7. ***Basal flagellomeres*** : (0) as wide as long; (1) 1.5 × as wide as long; (2) two to three × as wide as long; (3) >3 × as wide as long (discoidal).
8. ***Shape of palpimacula*** : (0) fusiform; (1) sulcate; (2) broadly ovoid.
9. ***Hypostomal bridge*** : (0) absent; (1) present.
10. ***Supraantenal region*** : (0) unmodified; (1) with fused tubercles; (2) with lateral, setose protuberances.
11. ***Coronal suture*** : (0) conspicuous; (1) indistinct.

**Prothorax**

1. ***Procoxal insertion*** : (0) at anterior apex; (1) at posterior end; (2) approximately at mid-length.
2. ***Prothorax*** : (0) two × as long as wide; (1) approximately as long as wide; (2) 1.5 × as long as wide.
3. ***Precoxal bridge*** : (0) present; (1) absent. Uninformative
4. ***Pronotum structure*** : (0) tubular (i.e., posteroventrally fused); (1) shield-shaped.
5. ***Ornamentation of the pronotum*** : (0) coarsely wrinkled; (1) with posterior, transversal furrow; (2) with three uneven, raised areas; (3) with short, raised posterior area; (4) with lateral, short protuberances.
6. ***Maculae*** : (0) present; (1) absent.
7. ***Postfurcasternum, shape*** : (0) narrow-shaped; (1) semi-triangular; (2) plate-shaped; (3) collar-like (i.e., ventrally fused).

**Foreleg**

1. ***Foretrochanter, anterior surface ornamentation*** : (0) absent; (1) with blunt process.
2. ***Trochanter-femur complex*** : (0) present; (1) absent.
3. ***Forefemur, shape*** : (0) fusiform; (1) subcylindrical (i.e., with approximately subparallel margins); (2) robust, with convex anterior and posterior surfaces.
4. ***Posteroventral row of integumentary specializations*** : (0) not raised; (1) carinate on distal 1/3; (2) carinate on distal ½.
5. ***Anteroventral row of integumentary specializations*** : (0) mostly reduced to apex; (1) fully developed; (2) reduced to proximal region and apex.
6. ***Proximal-most process morphology of anteroventral processes row*** : (0) as a prominent major process; (1) thorn-shaped; (2) as a primary, curved process; (3) not differentiated from other processes.
7. ***Posteroventral processes row, arrangement*** : (0) with two primary processes on distal ½; (1) uniform, having processes with similar shape and size; (2) with single sub-basal, primary process; (3) with two primary processes on proximal ½.
8. ***Type of specializations predominant on the rows*** : (0) spine-shaped; (1) tubercle-shaped.
9. ***Stinger-shaped setae on the rows of integumentary specializations*** : (0) absent; (1) present.
10. ***Thickened setae with globular base, adjacent to processes rows*** : (0) absent; (1) present.
11. ***Extension of the posteroventral row of thickened setae with globular base (when present)*** : (0) present on nearly the entire femoral, ventral surface; (1) present on distal ½ to apex of femoral, ventral surface.
12. ***Shape of the closing surface of the forefemur*** : (0) straight; (1) proximally curved.
13. ***Shape of the tibia*** : (0) smoothly curved; (1) straight (does not touch the entire surface of the femur when closed); (2) curved (making contact with the femur along its entire surface).
14. ***Tibial spur*** : (0) present; (1) absent.
15. ***Prostrate setae on tibial, ventral keel*** : (0) present; (1) absent.
16. ***Foretarsus condition on males and females*** : (0) 5-segmented in both sexes; (1) 4-segmented in both sexes; (2) sexually dimorphic, 4-segmented on males and 5-segmented on females.
17. ***Forebasitarsus morphology*** : (0) cylindrical; (1) with distal, ventral process; (2) with lanceolate process.
18. ***Length of lanceolate process relative to the eutarsus (when present)*** : (0) short, reaching the apex of the third tarsomere; (1) long, reaching the middle of the fourth tarsomere.
19. ***Number of rows of specialized setae on ventral surface of forebasitarsus*** : (0) two; (1) one; (2) absent.
20. ***Foretarsal Stitz organ*** : (0) absent; (1) present.

**Mid- and hind leg**

1. ***Shape of the hind tibia*** : (0) unmodified (thin and cylindrical); (1) remarkably expanded (oar-shaped) or fusiform.

**Wings**

1. ***Wings shape*** : (0) elongate and narrow; (1) broadly oval.
2. ***Vesicae*** : (0) absent; (1) present.

**Forewing**

1. ***Pterostigma shape*** : (0) fusiform; (1) oval; (2) rectangular; (3) trapezoidal.
2. ***Pterostigma color pattern*** : (0) variegated; (1) transversally divided into two colors; (2) dark with pale medial area.
3. ***Costal space width at basal region*** : (0) slightly expanded; (1) narrow (i.e., C and Sc veins nearly subparallel); (2) notably expanded.
4. ***Number of trichosors between the apex of two longitudinal veins on posterior wing margin*** : (0) 0; (1) 1; (2) ≥ 2.
5. ***Forked Subcostal veinlets*** : (0) absent; (1) present.
6. ***Number of crossveins in the subcostal space before pterostigma*** : (0) 2; (1) 3; (2) 1.
7. ***Humeral vein*** : (0) simple or forked; (1) branched.
8. **
Subcostal vein** : (0) not fused to RA at pterostigma level; (1) fused to RA at pterostigma level.
9. ***Number of crossveins in the radial space*** : (0) 3; (1) 4; (2) 1; (3) 2.
10. ***Shape of the sub-stigmal cell of the radial space*** : (0) gently curved; (1) oblique; (2) straight; (3) sigmoid; (4) arched.
11. ***Shape of the rm1 cell*** : (0) bar-shaped; (1) arrow-shaped; (2) trapezoidal.
12. ***Size of the rm1 cell (if trapezoidal) in relation to M divergence from R*** : (0) small (i.e., the M vein diverges from R close to RP stem origin); (1) enlarged (i.e., the M vein diverges from R far from RP stem origin).
13. ***M and R veins*** : (0) running parallel and very close at wing base; (1) fused at wing base.
14. ***M fork*** : (0) well beyond to R fork; (1) opposite or nearly opposite to R fork.
15. ***Shape of the Cubitus anterior (CuA)*** : (0) gently curved or only slightly bent near the middle; (1) forming a well-defined obtuse angle.
16. ***Cubitus posterior (CuP) of forewing*** : (0) not approaching A1; (1) approaching or touching A1.
17. ***Cubitus posterior (CuP), fork position*** : (0) slightly before 1m-cu level; (1) at midpoint between 1m-cu and RP origin; (2) opposite to RP origin level.
18. ***First anal vein (A1)*** : (0) basally forked; (1) branched; (2) simple; (3) distally forked.
19. ***Second anal vein (A2)*** : (0) branched; (1) forked.
20. ***Third anal vein (A3)*** : (0) close to jugal lobe; (1) forming a loop with the A2.

**Hind wing**

1. ***Hind wing*** : (0) nearly as long as the forewing; (1) reaching the distal margin of the forewing pterostigma; (2) reaching the proximal margin of the forewing pterostigma.
2. ***Costal and subcostal veins*** : (0) running subparallel, not fused; (1) fused at or slightly beyond the level of R fork.
3. ***RP branches*** : (0) terminating along posterodistal margin of wing; (1) terminating at wing apex.
4. ***Shape of 1r-m*** (i.e., ***first crossvein of the radiomedial space)*** : (0) straight; (1) sigmoidal.
5. ***Crossvein connecting the 1r-m to M stem distally*** : (0) absent; (1) present.
6. ***Shape of the media vein (M)*** : (0) forming angles at its forks and at connection with crossveins; (1) tuning fork-shaped.
7. ***First branch of Cubitus anterior (CuA)*** : (0) simple; (1) forked; (2) candelabrum-shaped.
8. ***Cubitus posterior (CuP)*** : (0) distally forked (i.e., close to posterior wing margin); (1) deeply forked.
9. ***Cubitus posterior (CuP) forming a loop with CuA*** : (0) absent; (1) present.
10. ***Intracubital crossvein*** : (0) absent; (1) subparallel to longitudinal wing axis; (2) reclined, nearly transverse.
11. ***Number of crossveins in the cubitoanal space*** : (0) 0; (1) 1; (2) 2.
12. ***Anterior branch of the first anal vein (A1) on hind wing*** : (0) ending on posterior wing margin fused to CuP; (1) fused to CuP for a short distance; (2) not fused to CuP.

**Abdomen**

1. ***Ornamentation on abdominal tergites*** : (0) absent; (1) with keeled processes on tergites, including tergites V–VI or V–VII; (2) with keeled processes on tergites, but not including V, VI, and VII.

**Male genitalia**

1. ***Sternites VIII and IX*** : (0) not fused; (1) fused.
2. ***Sternite IX, shape*** : (0) narrow, with rounded posterior margin; (1) approximately rhomboid, forming a broad canal; (2) pentagonal to semi-triangular with straight vertexes; (3) pentagonal with rounded posterolateral corners; (4) trapezoidal; (5) broadly rounded.
3. ***Posteromedial, dorsal canal on sternite IX*** : (0) absent; (1) present.
4. ***Gonocoxites IX*** : (0) equipped with hook-shaped processes; (1) fused to gonocoxites XI; (2) equipped with digitiform processes; (3) filiform, lacking digitiform processes; (4) greatly reduced and fusiform; (5) bar-shaped with blade-shaped or pointed apex.
5. ***Ventral region of the ectoproct*** : (0) with ventromedial lobe; (1) unmodified; (2) equipped with a patch of stout setae; (3) equipped with anterior lobe and ventromedial curved groove; (4) equipped with anterior bulging area and posterior concave surface; (5) equipped with anterior, flattened lobe anteriorly covered with conical, translucent setae.
6. ***Modified setae on posterior surface of the ectoproct*** : (0) absent; (1) present.
7. ***Gonapophyses X*** : (0) indistinct; (1) paired, not fused to gonocoxites X.
8. ***Gonapophyses X (when not fused with gonocoxites X) fused at posterior apex, forming a process*** : (0) absent; (1) present.
9. ***Gonocoxites X (when not fused with gonapophyses X) in lateral view*** : (0) only slightly wider on anterior apex; (1) narrow, subparallel sided; (2) with anterior apex expanded and dorsally bent; (3) with anterior region conspicuously expanded and dorsally curved or bent.
10. ***Gonostyli X*** : (0) short; (1) long (i.e., whip-shaped).
11. ***Gonostyli X, overall shape*** : (0) dorsally coiled; (1) medially recurved posteriorly; (2) forming an apical incurvation; (3) anteriorly forming a single loop approximately at midlength; (4) anteriorly coiled, forming two loops; (5) dorsally directed and ventrally recurved.
12. ***Median lobe of gonocoxites XI*** : (0) as a transverse, straight bar; (1) as a projection with median notch; (2) as a flattened rounded or quadrangular lobe; (3) with anterior and posterior, lateral, flattened processes; (4) complex and enlarged, with dorsal and ventral part.
13. ***Ventromedial posterior area of the gonocoxites XI median lobe (if the median lobe is enlarged and complex)*** : (0) not developed; (1) convex or protuberant; (2) with prominent posteroventrally projected process; (3) with digitiform or enlarged, caudally projected process.
14. ***Ventral ornamentation of the gonocoxites XI median lobe (if the median lobe is enlarged and complex)*** : (0) absent; (1) a concave covering that forms a curved process; (2) a prominent hook-shaped process; (3) a curved process with bilobed apex; (4) a short and blunt process; (5) an enlarged, curved, and rugose process.

**Female genitalia**

1. ***Tergite IX and ectoproct*** : (0) not fused; (1) fused.
2. ***Gonocoxites VII*** : (0) unpaired, forming a sternite-like sclerite; (1) paired, as two lateral plates; (2) as lateral plates connected through narrow posterior or median bridge.
3. ***Crumena*** : (0) present; (1) absent.
4. ***Gonocoxites + gonapophyses VIII*** : (0) paired; (1) unpaired, forming a simple sclerite; (2) unpaired, forming a complex structure.
5. ***Gonapophyses VIII medial part shape (when forms a complex structure)*** : (0) forming a tubular projection; (1) forming a broad, box-shaped structure; (2) forming a broad or straight canal; (3) keel-shaped; (4) as a polygonal, convex plate; (5) chamber-shaped.
6. ***Bilobed posteromedian process of the gonapophyses VIII (when forming a complex structure)*** : (0) absent; (1) thickened, with short lobes; (2) elongated and curved; (3) tiny, with short lateral lobes; (4) elongated, Y-shaped.
7. ***Tergite IX*** : (0) continuous with gonocoxites IX; (1) articulated with gonocoxites IX.
8. ***Gonocoxites IX, overall shape*** : (0) ovoid; (1) with hypocauda; (2) hose-shaped.
9. ***Gonapophyses IX*** : (0) absent; (1) present as two tiny sclerites concealed on the inner surface of the gonocoxites IX base.
10. ***Callus cercus*** : (0) present, protuberant; (1) absent.
11. ***Bursa copulatrix, sclerotization*** : (0) completely strongly sclerotized; (1) entirely membranous to slightly sclerotized; (2) with sclerotized and membranous areas.
12. ***Distal section of the spermatheca*** : (0) unmodified, as wide as proximal and medial sections; (1) thin, with blunt diverticulum; (2) conspicuously expanded, sac-shaped; (3) slightly wider than proximal and medial sections, lacking diverticulum; (4) wider than proximal and medial sections, equipped with blunt diverticulum; (5) with conical or trumpet-shaped invagination.
13. ***Fertilization canal duct*** : (0) short; (1) long.
14. ***Overall shape of the fertilization canal duct*** : (0) sinusoidal; (1) curved; (2) spiral-shaped; (3) sinusoid with proximal, triangular, concave portion.

## ﻿﻿Results

### ﻿﻿Phylogeny of Symphrasinae

The cladistic analysis performed using equal weight and New Technology Search recovered 33 most parsimonious trees with 311 steps in length (L), consistency index (CI) of 62 and retention index (RI) of 86, which generated a strict consensus tree with L = 319, CI = 61, and RI = 86. A weighted analysis using New Technology Searching yielded similar results to the analysis using equal weighting, and thus was not used for further discussion.

**Figure 3.**
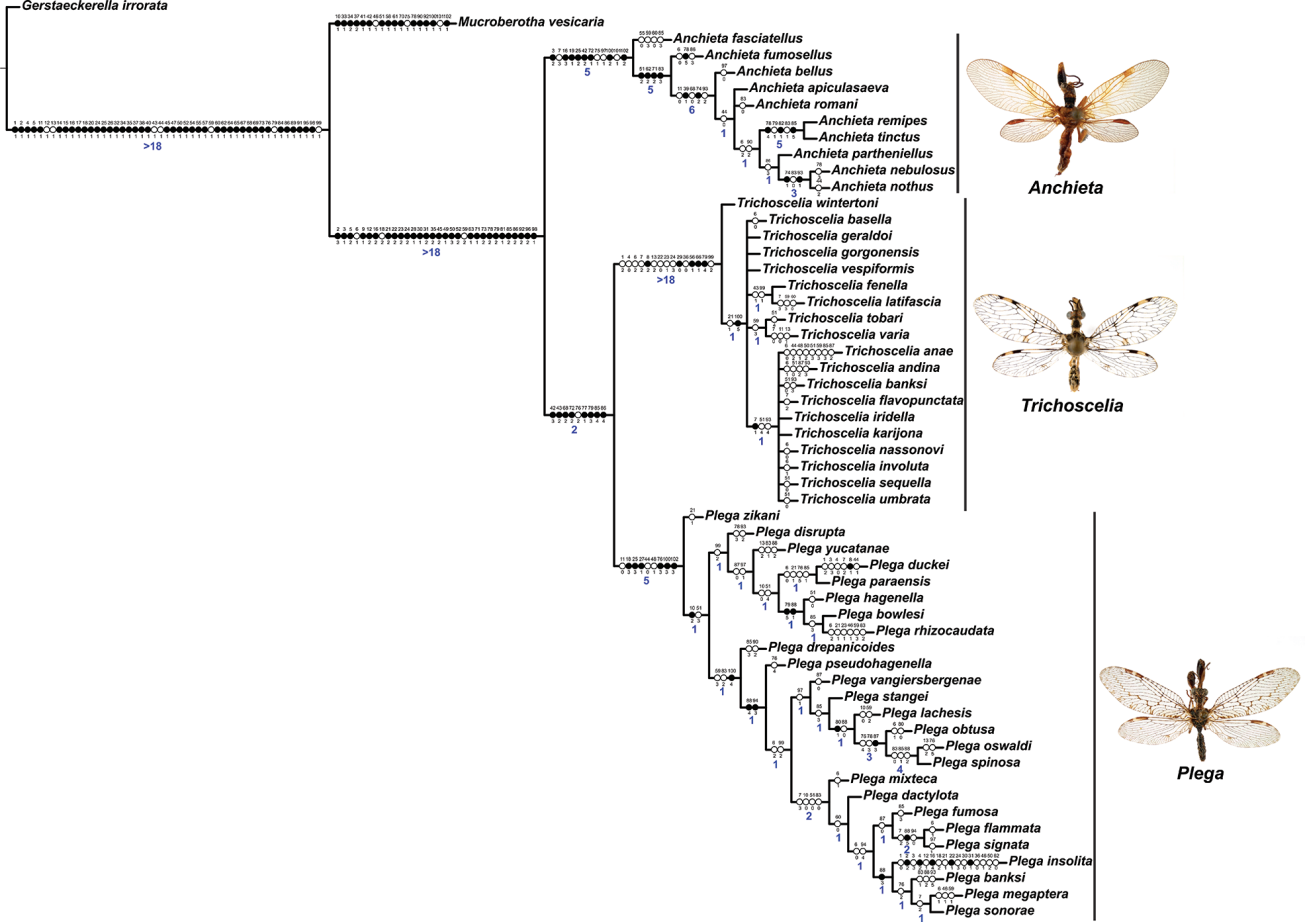
Morphological phylogeny of Symphrasinae. Strict consensus of 33 mot parsimonious trees resulting from a New Technology Searching and equal weight.

In this analysis, the subfamily Symphrasinae was recovered as a monophyletic group with strong support, with *Anchieta* as sister of *Plega* + *Trichoscelia* (Fig. 3). The unequivocal changes supporting this clade are the following: head characters such as the diamond-shaped head in frontal view (char 2:3), the pentagonal frontal sclerite (char 3:1), and the presence of hypostomal bridge (char 9:1). Thoracic features include the procoxal insertion approximately at mid-length of prothorax (char 12:2), and the presence of three uneven raised areas on the pronotum (char 16:2). Synapomorphies on the foreleg include the robust forefemur with convex anterior and posterior surfaces (char 21:2), the carinate distal ½ of the posteroventral row of processes of femur (char 22:2), the anteroventral row of processes reduced to proximal region and apex (char 23:2) with the proximal-most process a primary and curved process (char 24:2), presence of thickened setae with globular base adjacent to rows of processes of forefemur (char 28:1), the proximally curved closing surface of forefemur (char 30:1), and the curved tibia (char 31:2). Venational synapomorphies include the presence of more than two trichosors on the posterior wing margin between the apex of two longitudinal veins (char 45:2), the Sc vein fused to RA at pterostigma level (char 49:1), the presence of two crossveins in the radial space of FW (char 50:3), the C and Sc veins fused at or slightly beyond the level of R fork on HW (char 63:1), and the intracubital crossvein of the HW subparallel to longitudinal wing axis (char 71:1). Moreover, genital features supporting the monophyly of Symphrasinae in males are: gonocoxites IX set with apical, digitiform processes (char 78:2), the ventral region of ectoproct set with a patch of stout setae (char 79:2), the paired gonapophyses X not fused to gonocoxites X (char 81:1), the gonostyli X forming an apical incurvation (char 85:2), and the flattened, rounded or quadrangular median lobe of gonocoxites XI (char 86:2). Meanwhile, in the female genitalia, the absence of callus cercus (char 98:1) was recovered as a synapomorphy of the subfamily.

The genus *Anchieta* was recovered as monophyletic with *A.fasciatellus* (Westwood, 1867) sister to the rest of the species (Fig. 3). Synapomorphies for this clade include the rectangular frontal sclerite (char 3:2), a flattened pronotum with short, raised posterior margin (char 16:3), the presence of a blunt process on anterior surface of foretrochanter (char 19:1), the presence of single, sub-basal, primary process on the posteroventral row of processes of forefemur (char 25:2), the rectangular pterostigma of forewing (char 42:2), a single crossvein in the cubitoanal space of HW (char 72:1), the conspicuously expanded distal section of the spermatheca (char 100:2), and the spiral-shaped fertilization canal duct (char 102:2). The fused male sternites VIII and IX (char 75:1) also support the monophyly of *Anchieta*, yet this character is homoplastic in *Mucroberothavesicaria* Tjeder, 1968. *Anchietafumosellus* (Westwood, 1867) was recovered as sister of the remaining species of the genus based on the straight substigmal cell of the FW (char 51:2), the hind wing reaching the level of the proximal margin of pterostigma of the forewing (char 62:2), the reclined intracubital cell of HW (char 71:2), and the gonocoxites X with anterior region conspicuously expanded and dorsally curved or bent (char 83:3). The bee-mimicking species of *Anchieta* form a clade supported by the presence of an expanded or fusiform hind tibia (char 39:1) and the presence of keeled processes on abdominal tergites, but not including those of segments V, VI, and VII (char 74:2) as unequivocal changes (Fig. 3). Within this group of species, a sister group relationship between *A.remipes* (Gerstaecker, 1888) and *A.tinctus* Ardila-Camacho & Contreras-Ramos, sp. nov., is supported by the greatly reduced and fusiform male gonocoxites IX (char 78:4), the gonapophyses X fused at posterior apex, forming a process (char 82:1), and the dorsally directed and ventrally recurved gonostyli X (char 85:5). Moreover, *A.nebulosus* Ardila-Camacho & Machado, sp. nov. and *A.nothus* (Erichson, 1830) were recovered as sister species based on the presence of keeled processes on abdominal tergites, including tergites V–VI or V–VII, and the gonapophyses medial part of female forming a box-shaped structure (char 93:1).

The sister-group relationship between the genera *Trichoscelia* and *Plega* is supported by synapomorphic venational characters such as a trapezoidal pterostigma of FW (char 42:3) which is dark with pale medial area (char 43:2), a candelabrum-shaped first branch of CuA of HW (char 68:2), and two crossveins in the cubitoanal space of HW (char 72:2) (Fig. 3). Genitalic features supporting this group include the presence of posteromedial, dorsal canal on sternite IX (char 77:1), the ventral region of ectoproct set with anterior lobe and ventromedial groove (char 79:3), gonostyli X anteriorly coiled, forming two loops (char 85:4), and median lobe of gonocoxites XI complex and enlarged, with dorsal and ventral part (char 86:4).

The genus *Trichoscelia* was rendered monophyletic with strong support, although the relationships within the genus were largely unresolved and recovered in big polytomies (Fig. 3). As these results are difficult to explain, these will not be used for discussion. The characters supporting the monophyly of this genus include the broadly ovoid palpimacula (char 8:2), the posteroventral row of thickened setae with globular base present on nearly the entire femoral closing surface (char 29:0), the Cubitus anterior (CuA) of FW forming a well-defined obtuse angle (char 56:1), the presence of a crossvein connecting the 1r-m to the M stem distally on the HW (char 66:1), and the ventral region of male ectoproct equipped with anterior bulging area and posterior concave surface (char 79:4). Monophyly of this genus is also supported by the following homoplastic characters: the vertexal region above compound eyes approximately with the middle of the distance between upper limit of compound eyes and frontoclypeal ridge (char 1:2), the enlarged compound eyes (~0.75 of interocular distance at toruli level) (char 4:0), antennal scape > 2 × as long as wide (char 6:2), basal flagellomeres 2–3× as long as wide (char 7:2), prothorax 1.5 × as long as wide (char 13:2), the posteroventral row of processes of forefemur nor raised (char 22:0) and the anteroventral row fully developed (char 23:1) and with proximal-most process not differentiated from other processes (char 24:3). Furthermore, the short lanceolate process of forebasitarsus (i.e., reaching the apex of third tarsomere) (char 36:0) and the bursa copulatrix with sclerotized and membranous areas (char 99:2) (homoplastic in *P.insolita* Ardila-Camacho & Contreras-Ramos, sp. nov. and *Plega*, respectively) also support the monophyly of *Trichoscelia*. The conical or trumpet-shaped invagination on distal section of the spermatheca was recovered as a synapomorphy of all *Trichoscelia* species exclusive of *T.wintertoni* Ardila-Camacho & Contreras-Ramos, sp. nov., nonetheless it was an ambiguous character state in this latter species, so this feature should be considered as a synapomorphy of the whole genus.

The genus *Plega* was recovered as monophyletic with strong support based on six unequivocal changes such as the collar-like (i.e., ventrally fused) postfurcasterna (char 18:3), the posteroventral row of processes with two primary, spine-shaped processes on proximal ½ of forefemur (char 25:3), the presence of stinger-shaped setae on the integumentary specializations rows of forefemur (char 27:1), male sternite IX pentagonal with rounded posterolateral corners (char 76:3), distal section of the spermatheca slightly wider than proximal and medial sections, lacking diverticulum (char 100:3), and fertilization canal duct sinusoid with proximal, triangular, concave portion (char 102:3) (Fig. 3). In this clade, *P.zikani* Navás, 1936 was recovered as the sister species of the rest of the species of the genus which form a clade supported by a synapomorphy, i.e., supra-antennal region set with prominent lateral, setose protuberances (char 10:2), and a homoplasious character state, i.e., a sigmoid sub-stigmal cell of the FW (char 51:3).

The first clade in this group is composed by *P.disrupta* Ardila-Camacho & Contreras-Ramos, sp. nov., *P.yucatanae* Parker & Stange, 1965, *P.duckei* Penny, 1983, *P.paraensis* Penny, 1983, *P.hagenella* (Westwood, 1867), *P.bowlesi* Ardila-Camacho & Contreras-Ramos, sp. nov., and *P.radicaudata* Ardila-Camacho & Contreras-Ramos, sp. nov. In this group, the former species is sister of the rest, and the whole clade is supported by a homoplasious character state, i.e., a bursa copulatrix with sclerotized and membranous areas (char 99:2). Within this clade, there is a group conformed by three species, *P.hagenella*, *P.bowlesi*, and *P.radicaudata*, of which the former is sister of the latter two. Synapomorphies supporting this clade are the ventral region of the male ectoproct which is equipped with anterior, flattened lobe anteriorly covered with conical, translucent setae (char 79:5), and the ventral part of the gonocoxites XI medial lobe as a concave covering forming a curved process (char 88:1). A clade composed of *P.paraensis* and *P.duckei* was recovered as sister of that group and is supported only by homoplastic features such as a subcylindrical forefemur (char 21:1) and antennal scape as long as wide (char 6:0). These two sister clades are supported by homoplasious character states such as an unmodified supraantennal region (char 10:0) and an arched substigmal cell of FW (char 51:4). *Plegayucatanae* was found sister of these clades based on two homoplastic features, i.e., the ventromedial, posterior area of the gonocoxites XI median lobe being undeveloped (char 87:0), and the female gonapophyses IX as two tiny sclerites concealed on the inner surface of the base of gonocoxites IX (char 97:1).

Between the previous clade and the rest of the species of the genus, *P.drepanicoides* Ardila-Camacho & Contreras-Ramos, sp. nov., is sister of a large clade composed of two smaller clades and several intermediary species between them. This whole clade is supported by the distal section of the spermatheca which is wider than proximal and medial sections and equipped with blunt diverticulum (char 100:4) as an unequivocal change. Two homoplastic features further support this clade, i.e., distally forked A1 of FW (char 59:3) and male gonocoxites X with anterior apex expanded and dorsally bent (char 83:2). A transitional species between *P.drepanicoides* and the rest of species of *Plega* is *P.pseudohagenella* Ardila-Camacho & Contreras-Ramos, sp. nov. Such a clade is supported by two synapomorphies: a short and blunt process on the ventral part of the gonocoxites XI median lobe (char 88:4) and the presence of tiny posteromedian process of the female gonapophyses VIII with short lateral lobes (char 94:3). Inside this latter clade, there are two sister subclades whose monophyly is supported only by homoplastic features. The first one is composed of *P.vangiersbergenae* Ardila-Camacho, sp. nov., *P.stangei* Ardila et al., 2019, *P.lachesis* Ardila-Camacho & Contreras-Ramos, sp. nov., *P.obtusa* Ardila-Camacho & Contreras-Ramos, sp. nov., *P.oswaldi* Ardila-Camacho & Contreras-Ramos, sp. nov., and *P.spinosa* Ardila et al., 2019 and is supported by the presence of female gonapophyses IX as two tiny sclerites at the base of the gonocoxites IX (char 97:1). Of these species, the first one is sister of the rest, followed by *P.stangei* as sister of the remaining species. A clade composed of *P.lachesis* as sister of *P.obtusa* + (*P.oswaldi* + *P.spinosa*) is supported by an unequivocal change, the presence of modified setae on the posterior surface of the male ectoproct (char 80:1). The sister clade of *P.lachesis* is supported by a digitiform or enlarged ventromedial posterior area of the gonocoxites XI median lobe (char 87:3) as synapomorphic feature, while the trapezoidal male sternite IX (char 76:4), and the male gonocoxites IX lacking digitiform processes are homoplastic features also supporting this group.

The last clade in the phylogeny of *Plega* is composed by species found in the Mexican pacific coast, Baja California peninsula, Central and Northern Mexico, and Southwestern United States. This group is well defined and includes *P.mixteca* Ardila et al., 2019, *P.dactylota* Rehn, 1939, *P.fumosa* Linsley & MacSwain, 1955, *P.flammata* Ardila-Camacho & Contreras-Ramos, sp. nov., *P.signata* (Hagen, 1877), *P.insolita* Ardila-Camacho & Contreras-Ramos, sp. nov., *P.banksi* Rehn, 1939, *P.megaptera* Ardila-Camacho & Contreras-Ramos, sp. nov., and *P.sonorae* Ardila et al., 2019. Nonetheless, this a clade is supported only by four homoplasious characters states: basal antennal flagellomeres > 3 × as wide as long (char 7:3), the unmodified supraantennal region (char 10:0), the gently curved substigmal cell of FW (char 51:0), and the gonocoxites X only slightly wider on anterior apex (char 83:0). Within this clade, *P.mixteca* is sister to the rest of the species, followed by *P.dactylota* as sister of two sister clades which are supported by two homoplastic features. The first one, is the antennal scape as long as wide (char 6:0), and the posteromedial process on the female gonapophyses VIII medial part elongated and Y-shaped (char 94:4). Within this clade, the first subclade is composed by *P.fumosa* as sister of *P.flammata* + *P.signata*. Such a clade is supported by a homoplastic character state, i.e., the ventromedial posterior area of male gonocoxites XI median lobe not developed (char 87:0), while the sister relationship between the latter two species is supported by an enlarged, curved, rugose, ventral process on the male gonocoxites XI (char 88:3) as a synapomorphy. Additionally, the relationship between both species is supported by two homoplastic features, the basal antennal flagellomeres (char 7:2) and the absence of posteromedian process on the female gonapophyses VIII (char 94:0). The second clade is constituted by *P.insolita* as sister of *P.banksi* (*P.megaptera + P.sonorae*). This group is supported by a single synapomorphy, the presence of a ventral, curved process with bilobed apex on the ventral part of the gonocoxites XI median lobe (char 88:3). The latter clade is supported by a homoplastic character state, i.e., a pentagonal to semi-triangular male sternite IX with straight vertexes (char 76:2).

### ﻿﻿Taxonomy

**Class Insecta Linnaeus, 1758**

**Order Neuroptera Linnaeus, 1758**

**Family Rhachiberothidae Tjeder, 1959**

#### ﻿﻿Subfamily Symphrasinae

Taxon classificationAnimaliaNeuropteraRhachiberothidae

, Navás, 1909

Symphrasini Navás 1909: 484.
Anisopterinae Enderlein 1910: 341.
Platymantispinae Rehn 1939: 82.

##### Further description.

Penny (1982a); Penny and da Costa (1983); Lambkin (1986a); Wedmann and Makarkin (2007); Lu et al. (2020); Ardila-Camacho et al. (2021a); Li et al. (2023).

##### Taxonomy.

Rehn (1939b); Penny (1982a, b); Penny and da Costa (1983); Ardila-Camacho and García (2015); Ardila-Camacho et al. (2018, 2019).

##### Key to genera.

Navás (1912); Banks (1913); Penny (1982a, b); Penny and da Costa (1983); Hoffman (2002).

##### Phylogeny.

Penny and da Costa (1983) (Symphrasinae sister group of the remaining Mantispidae); Lambkin (1986a) (Symphrasinae sister group of other subfamilies of Mantispidae); Willmann (1990) (Symphrasinae sister group of other subfamilies of Mantispidae); Liu et al. (2015) (Symphrasinae sister group of other subfamilies of Mantispidae); Winterton et al. (2018) (Symphrasinae + Rhachiberothinae sister of Berothidae + (Mantispinae + (Calomantispinae + Drepanicinae)); Shi et al. (2019) (Symphrasinae sister of Mesomantispinae); Shi et al. (2020) (Symphrasinae sister of *Doratomantispa* Poinar); Lu et al. (2020) (Symphrasinae sister of Doratomantispinae + (Drepanicinae + (Calomantispinae + Mantispinae)); Ardila-Camacho et al. (2021a) (Symphrasinae + Paraberothinae sister of Rhachiberothinae).

##### Diagnosis.

This subfamily is distinguished from other mantispoid subfamilies by the presence of hypostomal bridge, the undeveloped laminatentorium and straight ocular plane. The procoxa are inserted approximately at mid-length of the prothorax, the pronotum is shield-shaped, and the postfurcasternum is well-developed and plate-shaped. On the foreleg, the femoral closing surface is proximally curved and presents two rows of thickened setae with globular base adjacent to the rows of processes; the foretarsus is four-segmented in both sexes with basitarsus equipped with a patch of clavate seta on anterior surface, a row of prostrate setae on the closing surface, and a long, lanceolate process set with an apical plug-shaped Stitz organ. On the wings, there are more than two trichosors on the posterior wing margin between the apex of two longitudinal veins, the Sc vein is fused to RA at pterostigma level, and two crossveins in the radial space of the forewing are present. Additionally, the CuP of the forewing is proximally bent, approaching or touching the A1. On the hind wing, the C and Sc veins are fused at or slightly beyond the level of R fork, the M vein is tuning fork-shaped, the 1r-m is sigmoid, and the CuP is developed and free. Male genitalia characteristics of Symphrasinae include gonocoxites IX set with apical, digitiform processes, paired gonapophyses X not fused to gonocoxites X, and gonostyli X remarkably long, incurved or forming loops. Moreover, on the female genitalia, the medial part of the gonapophyses VIII is enlarged and elaborated, the gonocoxites IX remarkably elongated forming an ovipositor, and the tergite IX and ectoproct are fused.

##### Biology.

Summarized in Ardila-Camacho et al. (2021b).

### ﻿﻿Key to Symphrasinae genera

**Table:** 

1	Foretrochanter with blunt process on anterior surface (Fig. 1B); HW with first branch of CuA simple or forked (Fig. 4B); male sternites VIII and IX fused (Fig. 5B); distal portion of spermatheca noticeably expanded, sac-like (Fig. 15H)	***Anchieta* Navás 1909**
–	Foretrochanter without blunt process on anterior surface; HW with first branch of CuA candelabrum-shaped (Fig. 2B); male sternites VIII and IX as separate units; distal portion of spermatheca narrow, generally with a diverticulum or invagination (Figs 27I, 82I)	**2**
2	Third labial palpomere expanded, with broadly ovoid palpimacula (Fig. 83C); forefemur with rows of thickened setae with globular base extending nearly over the entire closing surface; posteroventral processes row uniform, without spine-shaped processes (Fig. 89E, F); HW with 1r-m distally connected to M stem through a short crossvein (Fig. 81B); distal section of spermatheca with conical or trumpet-shaped invagination (Fig. 84I)	***Trichoscelia* Westwood, 1852**
–	Third labial palpomere narrow, with sulcate or narrowly ovoid palpimacula; forefemur with rows of thickened setae with globular base with some degree of reduction to distal portions (Fig. 1D); posteroventral processes row with two primary, spine-shaped processes on proximal 1/2 (Fig. 1E); HW with 1r-m not distally connected to M stem through a short crossvein; distal section of spermatheca with or without a diverticulum (Figs 43I, 78B)	***Plega* Navás, 1928**

#### ﻿﻿Genus Anchieta

Taxon classificationAnimaliaNeuropteraRhachiberothidae

Navás, 1909

Anisoptera Schneider, 1843: 32. Type species: Mantispanotha Erichson, 1839: 170 (now in Anchieta), by monotypy. Junior homonym of Anisoptera Berthold, 1827: 409 (in Orthoptera) and Anisoptera Herrich-Schäffer, 1840: 57, 69 (in Hymenoptera). Replaced by Platymantispa Rehn, 1939 and Anisopterana Strand, 1942. Synonymized with Anchieta by Penny (1982b): 216.
Anchieta Navás, 1909: 483. Type species: Anchietanobilis Navás, 1909: 484 (= Anchietafumosella (Westwood, 1867: 504)), by monotypy.
Platymantispa Rehn, 1939: 82. Name replacement for Anisoptera Schneider, 1843.
Anisopterana Strand, 1942: 389. Unnecessary name replacement for Anisoptera Schneider, 1843, previously replaced by Rehn (1939a).
Anchieta Bechyné, 1954: 176 (in Coleoptera). Junior homonym of Anchieta Navás, 1909.

##### Further description.

Gerstaecker (1888): 117; Enderlein (1910): 375 (as *Anisoptera*); Navás (1912): 201; Penny (1982b): 417; Penny and da Costa (1983): 610.

##### Taxonomy.

Gerstaecker (1888): 117 (*Anisoptera* = *Trichoscelia*); Enderlein (1910): 376 (*Anchieta* = *Trichoscelia*); Navás (1912): 201 (*Anchieta* valid genus); Banks (1913): 205, 206 (*Anchieta* and *Trichoscelia* = *Anisoptera*).

##### Key to species.

Banks (1913): 207–208 (Westwood’s species); Penny (1982a): 418; Penny and da Costa (1983): 611.

##### List of species.

Penny (1977): 37 (as *Trichoscelia*).

##### Diagnosis.

Compound eyes are relatively small, generally ½ the interocular distance at toruli level. The pronotum is as long as wide, unlike the other genera it presents only a slight outgrowth on the posterior margin. The forecoxa is slightly expanded at the apex, the trochanter is unique due to the presence of a blunt process on the anterior surface. The forefemur is setose; on the closing surface, the tubercle-shaped processes are noticeably thickened, with a spine-shaped sub-basal process on the posteroventral row. The anteroventral row is more reduced than in *Plega*, although like this, it presents the basal, primary, spine-shaped process. The foretibia is glabrous. On the hind leg, the tibia is generally markedly expanded. The pterostigma of the forewing is rectangular, and the *rarp2* is straight to gently curved; the hind wing is often noticeably shorter and narrower than the forewing, with the gradate series of crossveins absent or reduced to a single crossvein, and the first branch of the CuA is simple or forked. On the abdomen, the tergites of segments III–VII often present posteroventral keeled processes in both sexes. As in *Mucroberotha*, sternites VIII and IX of the male are fused, the sternite IX forms a wide canal, and the ectoproct presents a posteroventral patch of conical and thickened setae. The gonostyli X are short and recurved, and the gonocoxites XI have a flattened medial lobe. On the spermatheca, the distal section is expanded and sac-like.

##### Description.

***Head*.** Diamond-shaped in frontal view, smooth to rugose, region of vertex domed over compound eyes, paraocular area concave; coronal suture discrete. Antenna moniliform, flagellomeres discoidal on most of the flagellum. Compound eye hemispheric, as wide as 0.5–0.7 of the interocular distance at toruli. ***Thorax*.** Pronotum nearly as long as wide, with a groove adjacent to lateral and distal margins; posterior margin with slightly raised, entire surface with abundant, thickened setae arising flush the pronotal surface; postfurcasternum quadrangular, paired. Mesonotum wider than long, with abundant long, thickened setae, metanotum ~ 3 × as wide as long, glabrous. ***Foreleg*.** Coxa as long as femur, cylindrical, slightly distally expanded, densely setose. Trochanter semi-triangular, with a blunt process on anterior surface. Femur robust, densely setose; closing surface covered with fine and sinuous trichoid setae; posteroventral row processes fully developed, slightly carinated on distal ½, composed of thickened tubercle-shaped specializations with conical Stitz organs; proximally with a more developed, spine-shaped, sub-basal process; adjacent rows of thickened setae with globular base reduced to distal ¾ of the closing surface to a single apical seta; anteroventral row of processes reduced to the proximal region and apex, composed of tubercle-shaped integumentary specializations; basal primary process present, curved; adjacent row of thickened setae with globular base present in distal 4/5. Tibia nearly as long as femur, glabrous, curved, ventrally keel, with a row of prostrate setae; anterior surface with a patch of clavate setae at apex. Basitarsus with long lanceolate process surpassing distal margin of third tarsomere, equipped with a plug-shaped Stitz organ at apex; basal ½ with a row of prostrate setae on ventral surface, and patch of clavate setae on anterior surface; second tarsomere articulated in basal ½ of basitarsus on anterior surface, longer than third and fourth tarsomeres together; pretarsal claws simple. ***Mid- and hind leg*.** Mid-leg with thin to slightly widened tibia; hind leg thin to noticeably expanded, and laterally flattened, oar-shaped or fusiform. ***Wings*.** Forewing oval, trichosors present along wing margin, except at base; costal space medially narrow to slightly widened, humeral vein simple or forked, subcostal veinlets simple; pterostigma rectangular; Sc vein abruptly bent posteriad at proximal margin of pterostigma to merge with the RA; radial space with two crossveins; *rarp2* straight to gently curved; RP base located near separation of M and R, M fork near such separation; 1r-m between RP base and M fork forming a small trapezoidal cell; RP with single gradate series present; CuP basally angled, approaching A1. Hind wing oval, notably smaller and narrower than forewing; costal space narrow and reduced, C and Sc fused at proximal 1/3 of wing length; Sc vein abruptly curved posteriad at proximal margin of pterostigma to merge the RA; pterostigma elongated, narrow to slightly widened distally; radial space widened with single crossvein, straight to sinuous; RP with gradate series absent or reduced to a single crossvein. M forked at or slightly beyond R fork; Cu deeply forked, CuA ending at posterior margin at level of 1ra-rp, distally simple or forked, first branch simple or forked; intracubital vein generally reclined; CuP distally anteriorly curved or bent near posterior wing, pectinate. Cubitoanal space without crossveins. ***Abdomen*.** Tergites III and IV, or III–VI, or III-VII with poorly developed to prominent, posteromedial, keeled processes, present in both sexes.

***Male genitalia*.** Sternites VIII and IX fused, with fusion line weakly marked or absent; Sternite IX U-shaped, transversely curved to form a wide canal. Gonocoxites IX short and sinuous, or almost completely reduced, with or without apical processes. Ectoproct ovoid, with a posteroventral patch of conical, thickened setae. Gonocoxites X unpaired, forming a triangular or hourglass-shaped sclerite, concave or canaliculate on ventral surface, anterior portion spatulate, straight or dorsally bent; posterior apex with paired dorsal and lateral processes, articulated to gonostyli X and gonapophyses X, respectively; gonostyli X with thickened, concave base, set with curved lateral processes; the rest of the structure whip-like, short, recurved, with apex posteriorly curved, sometimes forming a loop. Gonapophyses X paired, rod-shaped, straight, thin, forming a V-shaped structure, joined by membranes; posterior apex with surrounding membrane set with minute granules. Gonocoxites XI thin, U-shaped, medial lobe dorsoventrally flattened, weakly sclerotized. Hypandrium internum concave, keeled, with two lateral fins.

***Female genitalia*.** Sternite VII (gonocoxites VII) trapezoidal or rectangular, sometimes as medially joined, lateral trapezoidal plates. Tergite VIII narrower medially than laterally, enclosing the spiracle of the segment VIII. Gonocoxites VIII forming a narrow, concave plate; gonapophyses VIII medial part canal-shaped, with or without a tubular process; lateral part as an enlarged plate, hidden under tergite IX + ectoproct, sometimes dorsally fused to form a covering. Tergite IX + ectoproct triangular, elongated. Gonocoxites IX long, straight and narrow; gonapophyses sometimes present as tiny sclerites located basally on inner surface of gonocoxites IX. Bursa copulatrix funnel-shaped, unsclerotized, short to long. Spermatheca short and irregularly entangled or long and spiral-shaped, proximal section short to long and thin; medial section thicker than proximal section, entangled or coiled; distal section expanded, wider than medial section, sac-shaped; fertilization canal duct long, thin, spiral-shaped; fertilization canal short to elongated, J-shaped, covered with microfilaments.

##### Included species.

1. *A.apiculasaeva* Thouvenot, 2009 (Brazil, French Guiana)

2. *A.bellus* (Westwood, 1867) (Brazil, Colombia, French Guiana, Suriname)

= *A.eurydella* (Westwood, 1867), new synonym

3. *A.fasciatellus* (Westwood, 1867) (Colombia, Panama)

4. *A.fumosellus* (Westwood, 1867) (Brazil)

= *Anchietanobilis* Navás, 1909

5. *A.nebulosus* Ardila-Camacho & Machado, sp. nov. (Brazil)

6. *A.nothus* (Erichson, 1830) (Brazil)

7. *A.partheniellus* (Westwood, 1867) (Brazil, Colombia, Ecuador, Suriname, Venezuela)

8. *A.remipes* (Gerstaecker, 1888) (Colombia)

9. *A.romani* (Esben-Petersen, 1817) (Brazil, Peru)

10. *A.sophiae* Ardila-Camacho & Contreras-Ramos, sp. nov. (French Guiana)

11. *A.tinctus* Ardila-Camacho & Contreras-Ramos, sp. nov. (Costa Rica)

##### Biology.

Summarized in Ardila-Camacho et al. (2021b).

##### Etymology.

Genus named in honor of the Jesuit missionary San José de Anchieta, evangelizer in Brazil between 1553 and 1597. Masculine gender as the name was erected after a man by Navás (1909).

### ﻿﻿Key to the species of *Anchieta*

**Table:** 

1	Hind tibia narrow and unmodified or only slightly widened (Fig. 10A)	**2**
–	Hind tibia noticeably expanded and flattened, fusiform or oar-shaped (Fig. 4F)	**4**
2	Body bright orange with dark brown bands (Fig. 8A); male gonocoxite IX with 8–13 apical processes arranged as a brush (Fig. 9C)	***Anchietafasciatellus* (Westwood, 1867)**
–	Body either yellow with dark brown stripes or dark brown with few orangish areas; male gonocoxite IX with different number and arrangement of the apical processes	**3**
3	Male gonocoxite IX with posterior apex blade-shaped, sometimes slightly lanceolate, or with 2 or 3 small, preapical processes (Fig. 11C–E); female gonapophyses VIII forming a tubular projection (Fig. 11H)	***Anchietafumosellus* (Westwood, 1867)**
–	Male gonocoxite IX filiform, with posterior apex sharply pointed (Fig. 23C–E); female gonapophyses VIII forming a chamber-shaped structure (Fig. 23H)	***Anchietasophiae* Ardila-Camacho & Contreras-Ramos, sp. nov.**
4	Body nearly completely dark brown (Fig. 14A)	**5**
–	Body mostly pale orange, with brown marks (Fig. 4A)	**7**
5	Male gonocoxite IX reduced, fusiform and helical, ventrally attached to lateral arms of gonocoxites XI (Fig. 25C–E); female gonapophyses VIII medial part composed by quadrangular plates forming a canal (Fig. 25G)	***Anchietatinctus* Ardila-Camacho & Contreras-Ramos, sp. nov.**
–	Male gonocoxite IX well-developed, filiform or thickened with digitiform processes (Figs 13D, 15C); female gonapophyses VIII medial part box-shaped (Fig. 13H)	**6**
6	Male gonocoxite IX with posterior apex ventrally curved, and set with three short processes (Fig. 15C–E)	***Anchietanothus* (Erichson, 1830)**
–	Male gonocoxite IX filiform, lacking processes (Fig. 13 C–E)	***Anchietanebulosus* Ardila-Camacho & Machado, sp. nov.**
7	Forewing costal space narrow (Fig. 6B); hind tibia fusiform (Fig. 6F); male gonocoxite IX with posterior apex pointed and set with four short, preapical, spine-shaped processes, located on the ventral surface (Fig. 7C–E)	***Anchietabellus* (Westwood, 1867)**
–	Forewing costal space medially expanded (Fig. 4B); hind tibia noticeably expanded, oar-shaped (Fig. 4F); processes on male gonocoxite IX with different arrangement	**8**
8	Male sternite IX with broad posteromedial, quadrangular lobe (Fig. 19B); male gonocoxite IX reduced and fusiform (Fig. 19A); male gonostyli X dorsally recurved and anteroventrally projected (Fig. 19A)	***Anchietaremipes* (Gerstaecker, 1888)**
–	Male sternite IX approximately rhomboid, with broad, rounded, posteromedial canal (Fig. 17E); male gonocoxite IX well-developed, bearing digitiform processes (Fig. 17C); male gonostyli X anteroventrally recurved, forming and apical incurvation in lateral view (Fig. 17C)	**9**
9	Male gonocoxite IX with posterior apex strongly lateroventrally recurved, set with two apical and four preapical, short processes (Fig. 17C–E)	***Anchietapartheniellus* (Westwood, 1867)**
–	Male gonocoxite IX with posterior apex laterally or ventrally curved, set with one or two short, preapical processes	**10**
10	Male gonocoxite IX apex laterally curved, set with one or two short preapical processes (Fig. 21C–E); male gonocoxites X anterior apex not expanded, laterally flattened (Fig. 21C, D)	***Anchietaromani* (Esben-Petersen, 1817)**
–	Male gonocoxite IX apex ventrally curved, set with one short, preapical process (Fig. 5C); male gonocoxites X anterior apex noticeably expanded, dorsally recurved (Fig. 5C, D)	***Anchietaapiculasaeva* Thouvenot, 2009**

#### ﻿﻿ Achieta apiculasaeva

Taxon classificationAnimaliaNeuropteraRhachiberothidae

Thouvenot, 2009

Figs 4 , 5

Anchieta apiculasaeva Thouvenot, 2009: 226. Holotype: male, French Guiana, [Régina] Piste de Kaw (MHNL), high resolution images examined.

##### Material examined.

***Holotype*.** French Guiana • ♂; [**Régina**] Route de KAW pk 3; 03 Mar. 1981; G. Tavakilian leg.; MHNL.

##### Other material.

Brazil • 1 ♂; abdomen cleared and stored in a microvial; INPA. – **Mato Grosso** • 1 ♂; Novo Mundo, Pq. Est. do Cristalino; 9°27'6.12"S, 55°50'22.56"W; 21–25 Jun. 2007; R.R. Cavichioli and A.C. Domahovski leg.; light trap; DZUP. French Guiana • 1 ♀; **Matoury**; Mont Matoury, Mont Grand Matoury; 4°51'31.00"N, 52°22'36.00"W; 28 Sep. 2014; flight interception trap, Colline entourée de savanes et zones dégradées; CSCA.

##### Diagnosis.

This species exhibits the same general body color pattern as *A.bellus*, *A.partheniellus*, *A.remipes*, and *A.romani*. It can be separated from *A.bellus* due to the expanded sub-basal region of the costal space in the forewing and the expanded and oar-shaped hind tibia. Moreover, the gonocoxite IX is short, sinuous, with posterior apex ventromedially gently curved, with two processes, one apical, and a shorter preapical, sometimes a third, more proximal, and shorter is present which separates this species from *A.partheniellus*. Meanwhile, the gonocoxites X are thickened, with the anterior ½ expanded, which allows to differentiate *A.apiculasaeva* from *A.romani*.

##### Description.

***Measurements*.** Male (*n* = 1). Forewing length: 9.3 mm; Hind wing length: 5.6 mm. Female (*n* = 1): Forewing length: 8.5 mm; Hind wing length: 5.3 mm.

**Figure 4.**
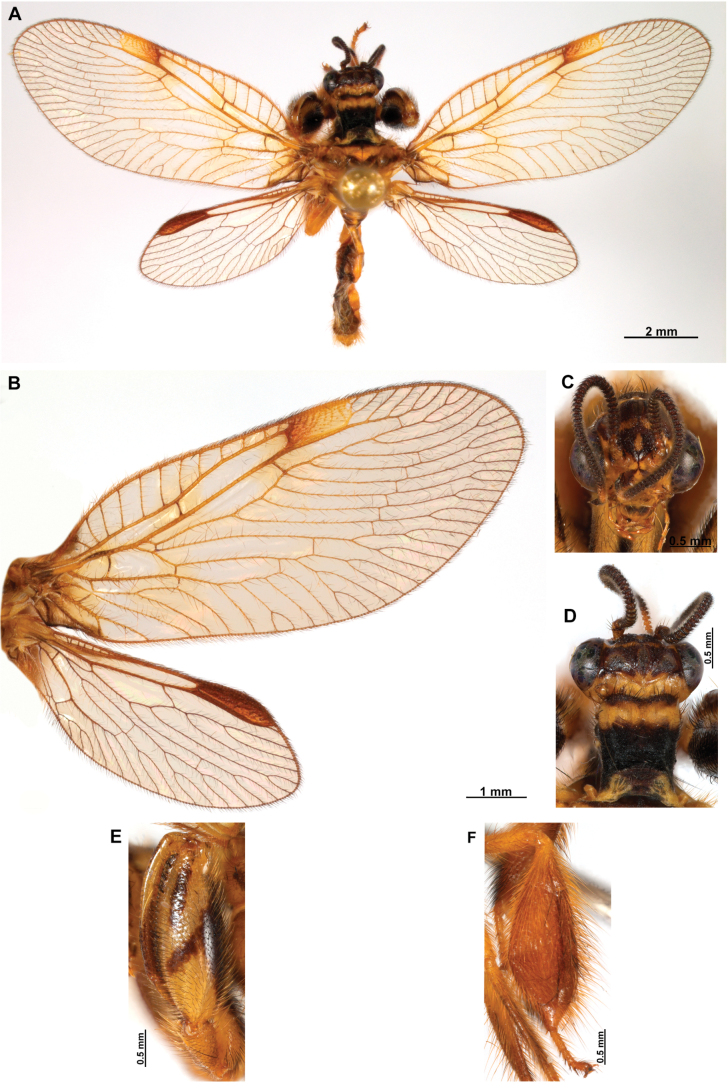
*Anchietaapiculasaeva* Thouvenot, 2009 **A** male habitus, dorsal **B** wings **C** head, frontal **D** pronotum, dorsal **E** forefemur, posterior surface **F** hind tibia, outer surface.

***Coloration* (Fig. 4). *Head*.** Mostly orange, vertexal region with broad, dark brown, transverse band, laterally with rows of dark brown setae; supraantennal area with lateral brown triangular markings located above the toruli, such areas connected to the frontal markings through the interantennal area; frons with brown lateral semi-triangular markings located below the toruli, forming an inverted V-shaped pattern. Antennal scape orange ventrally, brown dorsally, pedicel brown, flagellum brown, except for four or five yellow preapical flagellomeres. Clypeus yellow with brown suffusions, labrum with brown central region; mandible dark amber; maxillary palpus pale brown; labium orange; labial palpus with first and fourth palpomeres orange, second, third and fifth brown, palpimacula pale brown. ***Thorax*.** Pronotum dark brown with an orange band on distal 1/3, anterior margin dark brown, with concolorous setae. Meso- and metanotum mostly orange, with brown markings on area adjacent to parapsidal sutures, and on scutellum, setae pale brown; episternum dark brown; postfurcasternum dark brown with small, posterior, yellow area. Pteropleura mainly orange with some pale brown areas, setation orange. ***Foreleg*.** Coxa yellow at base, apex, and anterior surface; posterior surface with an extensive dark brown mark; setae mostly concolorous, except on anterior surface with interspersed orange and dark brown setae; trochanter bicolor, dorsally pale brown, ventrally orange, with interspersed brown and orange setae. Femur mainly orange with extensive, dark brown, dorsal mark, extending to anterior surface; posterior surface with two elongated brown markings, one located at apex, the other at middle; with interspersed orange and brown setae; tibia bicolor, basal ½ orange, with brown mark on dorsal surface; distal ½ brown, clavate setae orange; basitarsus pale brown with pale amber apex, remaining tarsomeres pale brown. ***Mid and hind legs*.** Mid-leg with predominantly yellow coxa, trochanter brown, femur orange; tibia predominantly pale brown; tarsus orange with dark brown setae on ventral surface. Hind leg coxa orange with pale brown suffusions, trochanter pale brown, femur orange with pale brown, medial suffusion; tibia orange with pale brown posterior margin; tarsus orange with dark brown setae on ventral surface. ***Wings*.** Forewing hyaline with orange areas base and area adjacent to proximal region of pterostigma; an orange transverse band located at R fork; pterostigma bicolor, pale brown on proximal ½, orange on distal ½; venation predominantly orange with brown suffusions at wing base and base and middle of R +M; apex of RP and M branches pale brown. Hind wing hyaline, base of subcostal space orange; pterostigma orange with blackish setation; venation orange with blackish suffusions at wing base. ***Abdomen*.** Tergites yellow with brown, irregular areas, tergite IV completely yellow; sternites yellow with orange setae; pleural membrane yellow on proximal 1/3 of abdomen, brown in the rest.

***Morphology*** (Fig. 4). ***Head*.** Diamond-shaped in frontal view, vertexal region domed above compound eyes, rugose, laterally with rows of short, reclined setae; paraocular area concave; coronal suture distinct. Compound eye hemispherical, as wide as ½ the interocular distance at toruli level. Antenna moniliform, flagellum slightly dorsoventrally flattened, with 50 flagellomeres, discoidal in shape, except for those of apex, narrower; all flagellomeres covered with medial ring of short setae. ***Thorax*.** Pronotum nearly as long as wide, with groove contiguous to lateral and distal margins; posterior margin with slight outgrowth in lateral view; entire surface with abundant, thick setae arising flush the pronotal surface; episternum with short, thin setae; postfurcasternum quadrangular. Mesonotum slightly wider than long, with abundant long, thickened setae on the medial region; metanotum ~ 3× as wide as long, glabrous. Pteropleura covered with abundant long, thin setae. ***Foreleg*.** Coxa as long as femur, cylindrical, slightly expanded at preapical region, with abundant fine and long setae; trochanter semi-triangular, densely setose, dorsally with long, thickened setae, anterior surface with blunt process. Robust femur, with abundant long and fine setae; closing surface with posteroventral row of integumentary specializations fully developed, composed of tubercle-shaped processes, with conical Stitz organs (process/seta ratio 1:1 or 2:1), proximally with a more developed, sub-basal, spine-shaped process (process/seta ratio 4:1); adjacent row of thickened setae with globular base present on distal ½; anteroventral row of integumentary specializations reduced to proximal region and apex, composed of tubercle-shaped processes with conical setae; primary process present, spine-shaped, curved; adjacent row of thickened setae with globular base present on distal 4/5. Tibia almost as long as femur, curved, glabrous, with a row of prostrate setae on the closing surface, and patch of clavate setae apically on anterior surface. Basitarsus with long lanceolate process, proximal region with clavate setae on anterior surface, and a row of prostrate setae ventrally. ***Mid and hind legs*.** Mid-leg covered with abundant fine and long setae, tibia slightly widened. Hind leg densely covered with long, fine setae, except on the tarsus, where they are shorter, tibia notably widened and laterally flattened, oar-shaped. ***Wings*.** Forewing oval, venation densely setose, trichosors present along wing margin; costal space slightly widened medially, humeral vein simple, 10–12 veinlets present; pterostigma rectangular; subcostal space with a single medially located crossvein; Sc vein abruptly bent posteriad at proximal margin of pterostigma to merge with the RA; *rarp2* straight with three veins arising from it, three or four veins from *rarp1*; M fused basally to R; RP base located near separation of M and RA, M fork near such separation; 1r-m located between RP base and M fork, forming a small trapezoidal cell; five or six gradate crossveins present; Cu deeply forked, CuP basally angled, forked; A1 simple, A2 distally forked. Hind wing oval, notably smaller and narrower than forewing; costal space narrow and reduced, with seven veinlets; C and Sc fused at proximal 1/3 of wing length, subcostal space without crossveins; Sc vein abruptly curved posteriad at proximal margin of pterostigma to merge with RA; pterostigma elongated, slightly widened distally; radial area widened with single, sinuous crossvein; two veins arising from *rarp1*, two from *rarp2*. M vein forked at level of R fork; Cu deeply forked, CuA sinuous, distally forked; CuP distally, anteriorly bent, with three branches; A1 arched, A2 short, simple. ***Abdomen*.** Medially widened, tergites III and IV with short, keeled processes, tergites V–IX with abundant long, thin setae; sternites densely setose.

**Figure 5.**
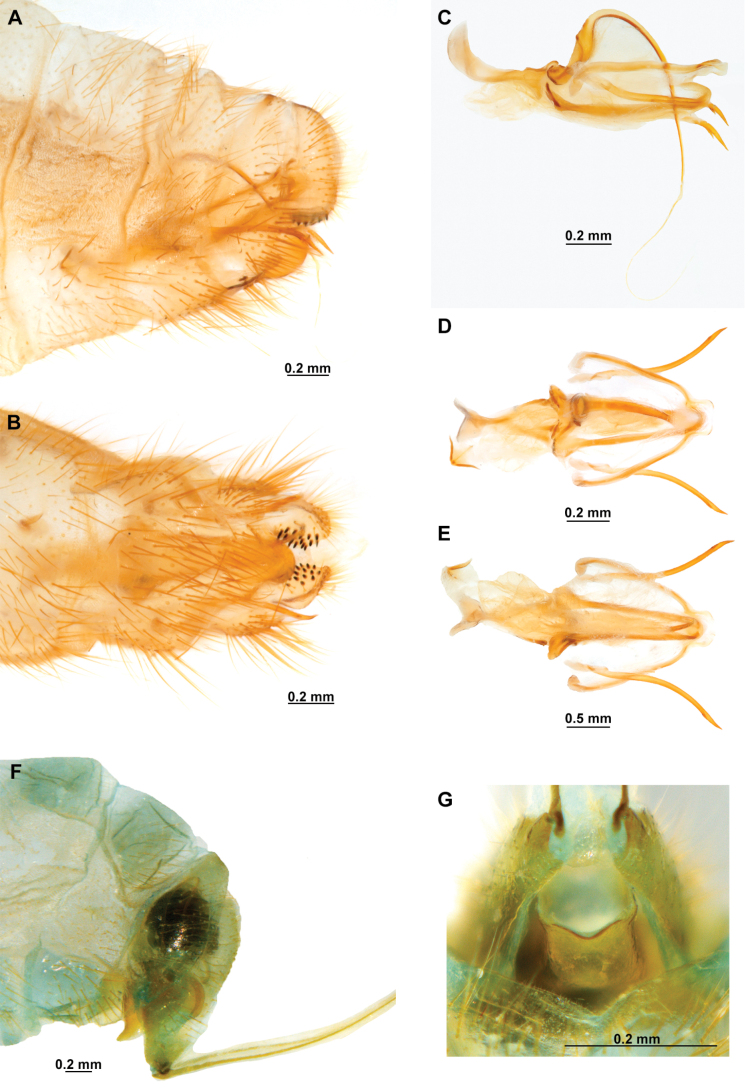
*Anchietaapiculasaeva* Thouvenot, 2009 **A** male terminalia, lateral **B** same, ventral **C** male genitalia, lateral **D** same, dorsal **E** same, ventral **F** female terminalia, lateral **G** gonapophyses VIII of female, ventral.

***Male genitalia*** (Fig. 5A–E). Tergite IX medially narrower than laterally, lateral region covered with abundant long and thickened setae. Sternites VIII and IX fused, with weakly marked fusion line; sternite VIII subrectangular with abundant long and thickened setae; sternite IX U-shaped, with slightly concave lateral margins, and abundant long and thickened setae anteriorly and laterally; posteromedially with convex and glabrous area. Gonocoxite IX short, sinuous, posterior apex ventromedially gently curved, with two processes, one apical, and a shorter preapical, sometimes a third, more proximal, and shorter is present. Ectoproct ovoid, setose, posteroventrally with an area covered with 17 or 18 stout setae; outer surface with smooth, glabrous, bulging area; inner surface with sclerotized ventral area, set with a patch of microtrichia. Gonocoxites X thickened, medially slightly constricted, hourglass-shaped, ventrally canaliculated; anterior ½ expanded, gently curved dorsally, posterior ½ triangular, with paired lateral and dorsal processes. Gonostyli X base triangular, thickened, with curved lateral processes, dorsally concave, the rest of the structure whip-shaped, short, distal portion curved. Gonapophyses X rod-shaped, straight, narrow, anterior apex spatulate, posterior apex curved dorsally; gonapophyses forming a V-shaped structure joined by membranes. Gonocoxites XI thin, U-shaped, medial lobe flattened, weakly sclerotized at center, posterior margin almost straight, lateral borders blunt; lateral arms of gonocoxites straight, apex ventromedially curved.

***Female genitalia*** (Fig. 5F, G). Sternum VII (gonocoxites VII) composed of two medially fused trapezoidal lateral plates, posterior margin obtuse. Tergite VIII narrower medially than laterally, enclosing the segment spiracle, lateral margin triangular. Gonocoxites VIII as two concave, medially fused, trapezoidal plates; gonapophyses VIII medial part ventrally projected forming a broad canal, distal margin rounded, ventrally curved; lateral part of gonapophyses VIII a trapezoidal plate, forming a chamber-shaped plate, dorsally with two convex areas, located behind the genital pore. Tergite IX + ectoproct semi-triangular. Gonocoxite IX long, straight, narrow. Bursa copulatrix unsclerotized, long, conical; spermatheca spiral-shaped; proximal section long, thin, progressively thickened, spiral-shaped, forming three coils; medial section slightly narrower than thickest part of proximal section, entangled, forming several convolutions; distal section thicker than proximal and medial sections, progressively wider towards the apex. Fertilization canal duct long, spiral-shaped; fertilization canal elongated, narrow, J-shaped, covered with microfilaments.

##### Distribution.

Brazil (Mato Grosso), French Guiana (Matoury, Régina).

##### Remarks.

This species was previously known solely from the holotype male collected at Régina, French Guiana and was deposited in the private collection of the author of the species (Thouvenot 2009). After looking for loan of the holotype in the context of the present revision, M. Thouvenot informed that the type was deposited in the Musée des Confluences at Lyon, France (MHNL) (M. Thouvenot, C. Audibert, pers. comm., 18 June 2019). Unfortunately, because the holotype is the only specimen known of this species, a loan from the Musée des Confluences was not possible, so only high-resolution images of the type were studied. Herein several additional specimens were identified, and the distribution range of this species is extended to Matoury in French Guiana, and to Matto Grosso in Brazil.

This species exhibits the same coloration pattern of other species of the genus mimicking stingless bees of the genus *Ptilotrigona* Moure, 1951 (Rasmussen and Ardila-Camacho 2021). Based on the wing shape and venation, hind tibia shape, and morphology of the male genitalia, this species appears to be closely related to *A.romani*. In the phylogeny of the subfamily, this species was recovered within the clade composing all the species exhibiting Batesian mimicry with stingless bees (i.e., *A.bellus*, *A.apiculasaeva*, *A.romani*, *A.remipes*, *A.tinctus*, *A.partheniellus*, *A.nebulosus*, and *A.nothus*).

#### ﻿﻿ Anchieta bellus

Taxon classificationAnimaliaNeuropteraRhachiberothidae

(Westwood, 1867)

Figs 6 , 7

Mantispa (Trichoscelia) bella Westwood, 1867: 502. Holotype: female, Amazonia (OUMNH), high resolution photos studied.
Mantispa (Trichoscelia) eurydella Westwood, 1867: 501. Holotype: female, Amazonia (OUMNH), high resolution photos studied. New synonym.
Anisoptera bella (Westwood, 1867). Gerstaecker (1888).
Anisoptera eurydella (Westwood, 1867). Gerstaecker (1888).
Trichoscelia bella (Westwood, 1867). Enderlein (1910).
Trichoscelia eurydella (Westwood, 1867). Enderlein (1910).
Anchieta eurydella (Westwood, 1867). Penny (1982a), Ohl (2004), Ardila-Camacho and García (2015), Ardila-Camacho et al. (2018).

##### Material examined.

***Holotype*** of Mantispa (Trichoscelia) bella.

Brazil • ♀; **Amazonas**; 1861; H.W. Bates leg.; Type Neur.: No. 8, *MantispaTrichosceliabella* Westwood, HOPE Dept. Oxford; Type Westwood, Trans. Ent. Soc. 1867, p. 502, Coll. Hope Oxon; OUMNH.

***Holotype*** of Mantispa (Trichoscelia) eurydella.

Brazil • ♀; **Amazonas**; 1861; H.W. Bates leg.; Type Neur.: No. 4, *MantispaTrichosceliaeurydella* Westwood, HOPE Dept. Oxford; Type Westwood, Trans. Ent. Soc. 1867, p. 501, Coll. Hope Oxon; OUMNH.

##### Other material.

Brazil – **Amazonas** • 1 ♂; Mamiraua, varzea; 03°02'54"S, 64°51'02"W; 22 Sep. 1993; I.S. Gorayeb and O.T. Silveira leg.; MPEG. – **Pará** • 1 ♀; Tucuruí, Rio Tocantins, Saude; 1–3 Jun. 1984; arm. Suspensa; MPEG; • 1 ♀; Belém, Floresta APEG; 13–16 Aug. 1983; I.S. Gorayeb leg.; MPEG. • 1 ♀; same data as for preceding; 10–14 Jan. 1983; I.S. Gorayeb e equipe leg.; arm. Suspensa; MPEG. • 1 ♀; Serra Norte, estrada do Manganes; 12 May. 1984; arm. Suspensa; MPEG. • 1 ♀; same data as for preceding; N3; 26–29 Jun. 1985; arm. Suspensa; MPEG.

Colombia • 1 ♀; 1997; *Anchietaeurydella* det. M. Ohl, 2006; ZMB.

Suriname • 1 ♂; **Brokopondo**; 24 Apr. 1965; G.F. Mees leg.; *A.remipes*, det. L.A. Stange; FSCA.

##### Diagnosis.

This species is distinguished from its congenerics by the narrow costal space of the forewing and the hind tibia moderately expanded and fusiform. On the male genitalia, the gonocoxite IX is narrow, curved, with posterior apex pointed and set with four short, preapical, spine-shaped processes, located on the ventral surface. The gonocoxites X are anteriorly expanded. On the female genitalia, the gonapophyses VIII medial part is ventrally projected, forming a broad, short, blunt canal.

##### Description.

***Measurements*.** Male (*n* = 1). Forewing length: 8.0 mm; Hind wing length: 4.9 mm.

**Figure 6.**
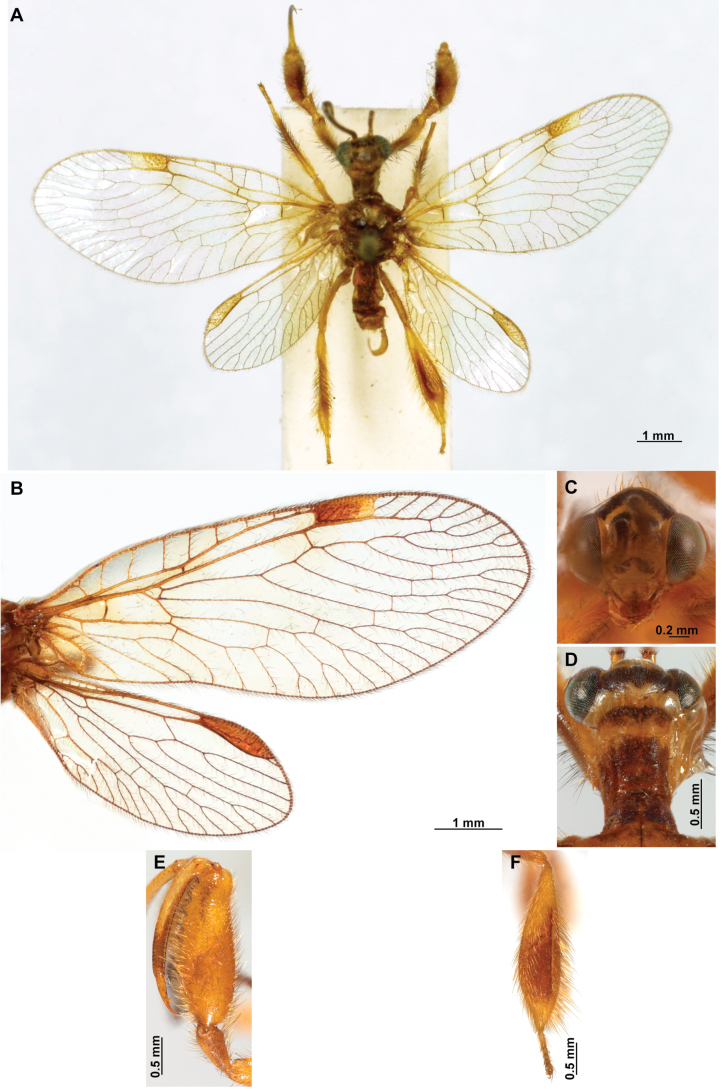
*Anchietabellus* (Westwood, 1867) **A** female habitus, dorsal **B** wings **C** head, frontal **D** pronotum, dorsal **E** forefemur, posterior surface **F** hind tibia, outer surface.

***Coloration*** (Fig. 6). ***Head*.** Mostly orange, vertexal region with transverse brown band connected anteriorly to triangular dark brown mark on supraantennal region through coronal suture, marking of supraantennal area enclosing central orange spot; laterally with orange setae. Antennal scape mainly orange, amber at apex, pedicel pale brown; flagellum brown, except for five orange, preapical flagellomeres. Frons with an inverted, brown, V-shaped marking. Clypeus and labrum with yellowish setae; mandible orange, with amber apex; maxillary palpus orange; labium orange, including palpimacula. ***Thorax*.** Pronotum pale brown with transverse orange band on distal 1/3, setae pale brown; episternum; postfurcasternum orange with brown suffusions. Mesonotum orange with pale brown suffusions, medial region of scutum and scutellum brown, setae orange; metanotum orange with dark brown suffusions mainly at center; pre-episternum brown; mesopleuron with episternum mostly orange, epimeron brown, setation orange; metapleuron with orange episternum, epimeron brown, the entire surface with orange setae. ***Foreleg*.** Coxa mostly orange with a pale brown area externally on basal ½, this area plus the apex with dark brown setae, the rest of the surface with orange setae; trochanter orange on ventral surface, pale brown on dorsal surface, setae mostly orange, with a few dark brown setae on dorsal surface. Femur orange with pale brown areas on dorsal and lateral surfaces, setae mostly orange with some scattered brown setae; tibia with orange basal ½, distal ½ amber, clavate setae orange; basitarsus orange with amber lanceolate process; second to fourth tarsomere orange. ***Mid- and hind leg*.** Mid-leg coxa mostly orange, with pale brown margins and orange setae; trochanter pale brown; femur orange, pale brown on center, with concolorous setae; tibia with basal ½ and tip orange, preapical region pale brown with dark brown setae; tarsomeres orange. Hind leg coxa mostly orange with pale brown margins and orange setae, trochanter pale brown, femur with orange base and apex, pale brown on remaining surface; tibia with orange base and apex, medial region with brown suffusions; tarsus pale brown with orange setae. ***Wings*.** Forewing hyaline, pale amber at base, with transverse pale amber band at R fork, and area surrounding the 1ra-rp; proximal ¾ of pterostigma pale brown, orange on distal ¼; venation uniformly pale brown; hind wing mainly hyaline, pale brown at base; base of subcostal area pale amber; pterostigma pale brown, venation uniformly pale brown, with paler areas on cubital and anal veins. ***Abdomen*.** Orange with pale brown areas.

***Morphology*** (Fig. 6). ***Head*.** Diamond-shaped in frontal view, smooth; vertexal region domed above compound eyes, with lateral rows of short, reclined setae; paraocular area; coronal suture distinct. Antenna moniliform, flagellum with 45 flagellomeres, discoidal in shape, those at the apex narrower; all flagellomeres with medial ring of short setae. Compound eye hemispheric, as wide as ½ the interocular distance at toruli level. ***Thorax*.** Pronotum slightly longer than wide, with groove contiguous to lateral and distal margins; in lateral view, posterior margin slightly elevated, entire surface with abundant long and thickened setae, arising flush the pronotal surface; episternum setose; postfurcasternum quadrangular. Mesonotum slightly wider than long, with abundant long and thick setae on medial region; metanotum ~ 3× as wide as long with. Pteropleura covered with abundant fine, long setae. ***Foreleg*.** Coxa as long as femur, sub-cylindrical, slightly expanded on preapical region, with abundant long and fine setae; trochanter subconical, densely setose, dorsally with a tuft of long and thick setae, anterior surface with blunt process covered with long setae. Femur robust, with abundant fine and long setae; closing surface with posteroventral row of integumentary specializations fully developed, composed of tubercle-shaped processes with conical Stitz organs, proximally with a more developed sub-basal, spine-shaped process, adjacent row of thickened setae with globular base present on distal ½; anteroventral row of specializations reduced to proximal region and apex, composed of tubercle-shaped processes with conical setae; primary, spine-shaped, curved process present; adjacent row of thickened setae with globular base present on distal 4/5. Tibia almost as long as the femur, curved, glabrous, closing surface with a row of prostrate setae; with a patch of clavate setae apically on anterior surface. Basitarsus with long lanceolate process, proximal part with clavate setae on anterior surface, ventrally with a row of prostrate setae. ***Mid and hind legs*.** Mid-leg with coxa, trochanter and femur set with long and fine setae, tibia slightly expanded medially, with abundant long and thickened setae; tarsus covered with short setae, the first tarsomere as long as the next three together, the second as long as the third and fourth together. Hind leg with the coxa covered with long, fine setae, trochanter with few long, fine setae; femur cylindrical, covered with long, fine setae; tibia moderately expanded, laterally flattened, fusiform, densely setose; tarsomeres with short, thin setae, the first tarsomere as long as the next three together, the second as long as the third and fourth together. ***Wings*.** Forewing oval, venation setose, trichosors present along wing margin; costal space narrow, humeral vein forked, 8–10 crossveins present; pterostigma rectangular; subcostal space with single, medially located crossvein, Sc vein abruptly bent posteriad at proximal margin of pterostigma to merge with the RA; rarp2 straight with three veins arising from it, two from *rarp1*; M basally fused to R; RP located near separation of M and R, M fork near such separation; 1r-m located between RP base and M fork; four or five gradate crossveins present; Cu deeply forked, CuP proximally angled, approaching A1, forked; A1 without simple. Hind wing notably smaller and narrower than forewing, oval; narrow and reduced costal space with five crossveins; C and Sc fused at proximal 1/3 of wing length; Sc vein abruptly curved posteriad at proximal margin of pterostigma, to merge with RA; subcostal space without crossveins; pterostigma elongated, with the subapical portion expanded; radial space with single, sinuous crossvein; two veins arising from *rarp1*, one from *rarp2*. M forked at or slightly beyond R fork; 1r-m sigmoid; gradate crossveins absent. Cu vein deeply forked, CuA sinuous, distally forked; CuP distally anteriorly gently curved near wing margin, branched; A1 arched, A2 short, simple. ***Abdomen*.** Medially widened, tergites of abdominal segments III and IV with short keeled posteromedial projections.

**Figure 7.**
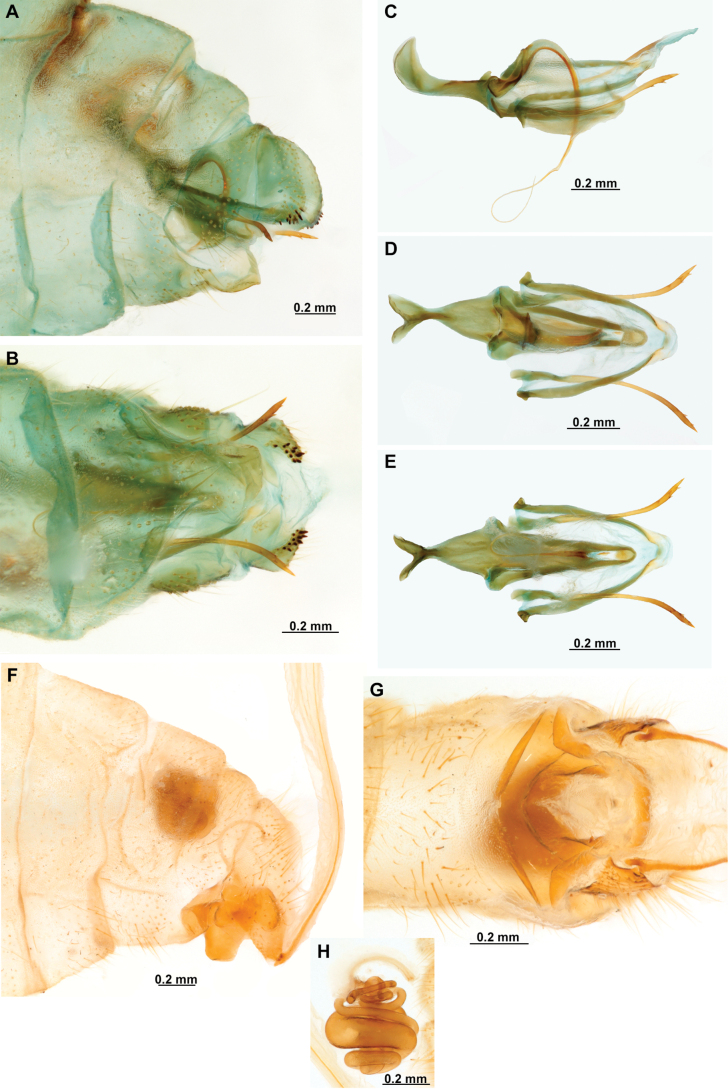
*Anchietabellus* (Westwood, 1867) **A** male terminalia, lateral **B** same, ventral **C** male genitalia, lateral **D** same, dorsal **E** same, ventral **F** female terminalia, lateral **G** same, ventral **H** spermatheca.

***Male genitalia*** (Fig. 7A–E). Tergite IX notably narrower medially than laterally; posterolaterally setose and with small cleft. Sternites VIII and IX fused, with fusion line distinct; sternite VIII ~ 2× as wide as long; sternite IX narrow, ventrally projected, transversely curved, broadly canaliculate, receiving the apex of gonapophyses X. Gonocoxites IX narrow, curved, base spatulate; apex pointed with four short, preapical processes, located on ventral surface. Ectoproct ovoid, lateral surface with smooth protrusion, posteroventral region with ~ 11 short and thick setae; ventral region with moderately sclerotized plate, equipped with few thin and short setae. Gonocoxites X medially constricted, hourglass-shaped, ventrally canaliculate; anterior apex expanded, curved dorsally; posterior apex with dorsal and lateral processes; base of gonostyli X thickened, concave, with lateral rounded lobes, the rest of the structure thin, ventrally curved, whip-shaped, short, reaching anterior margin of sternite VII, with terminal incurvation. Gonapophyses X elongated, straight, narrow, with the anterior apex slightly dorsally bent; gonapophyses arranged in a V-shaped structure, joined by membranes. Gonocoxites XI narrow, U-shaped, medial lobe as a thin, quadrangular plate with blunt corners; lateral arms of gonocoxites XI short with anterior apex curved ventrally.

***Female genitalia*** (Fig. 7F–H). Sternum VII (gonocoxites VII) rectangular with posterior margin concave. Tergite VIII as wide medially as laterally, enclosing the segment, lateral margin rounded. Gonocoxites VIII as a bar-shaped, concave plate, forming and obtuse angle; gonapophyses VIII medial part, ventrally projected, forming a broad, short, blunt canal; lateral part of gonapophyses VIII a narrow, convex plate, forming a chamber-shaped covering dorsally, located behind the genital pore. Tergite IX + ectoproct triangular. Gonocoxite IX long, straight, narrow, as long as the last six abdominal segments together. Bursa copulatrix unsclerotized, short, funnel-shaped; spermatheca spiral-shaped; proximal section long, thin, progressively thickened, spiral-shaped, forming three coils; medial section slightly thicker than proximal section, forming several convolutions; distal section thicker than proximal and medial sections, progressively wider towards the apex, terminal portion expanded. Fertilization canal duct long, spiral-shaped, forming four coils; fertilization canal elongated, narrow, J-shaped, covered with microfilaments.

##### Distribution.

Brazil (Amazonas, Pará), Colombia, French Guiana (Saint-Laurent-du-Maroni) (doubtful record), Suriname (Brokopondo).

##### Remarks.

This species was described based on a female specimen collected by H. Bates. The only collecting data available on the original labels indicate “Amazons” as in many other types of the subfamily. In the present study, further specimens from Suriname, Colombia and Brazil (Amazonas and Pará) were studied and their genital structures and wing venation redescribed. Based on careful comparisons, *A.eurydella* is herein proposed as a synonym of *A.bellus*, as both share the same characteristics in the wing shape, venation and hind tibia shape (i.e., fusiform and narrow). The differences in coloration used by Penny (1982a) and Penny and da Costa (1983) to separate these species are interpreted as intraspecific variation in the intensity of the coloration of the body (darker in *A.eurydella* and paler in *A.bellus*), a rather common attribute in Symphrasinae. *Anchietaeurydella* has also “Amazons” as collecting data, and the type, like *A.bellus* is a female specimen. Penny (1982a) and Penny and da Costa (1983) provided records of *A.bellus* from the Brazilian states of Amazonas and Pará, and from Saint-Laurent-du-Maroni, French Guiana, nevertheless, the taxonomic identification of the specimens from Amazonas and Saint-Laurent-du-Maroni is herein considered as doubtful, primarily the first record which was made from a female. The Brazilian specimen could actually correspond to *A.partheniellus*, whereas the specimen from French Guiana could be *A.apiculasaeva*. Further examination of these specimens will be necessary to corroborate these records.

In the phylogeny of the subfamily, *A.bellus* was recovered as sister of the rest of the species mimicking stingless bees. Like other closely related species, *A.bellus* has a mimicry pattern resembling meliponine bees of the genus *Ptilotrigona* (Rasmussen and Ardila-Camacho 2021).

#### ﻿﻿ Anchieta fasciatellus

Taxon classificationAnimaliaNeuropteraRhachiberothidae

(Westwood, 1867)

Figs 8 , 9

Mantispa (Trichoscelia) fasciatella Westwood 1867: 503. Holotype: female, Santa Martha, Venezuela (OUMNH), high resolution images studied.
Anisoptera fasciatella (Westwood, 1867). Gerstaecker (1888).
Trichoscelia fasciatella Westwood, 1867. Enderlein (1910).
Plega fasciatella (Westwood, 1867). Penny (1982a), Ohl (2004), Ardila-Camacho and García (2015).
Anchieta fasciatella (Westwood, 1867). Ardila-Camacho et al. (2018).

##### Material examined.

***Holotype*** of Mantispa (Trichoscelia) fasciatella. Colombia • ♀; [**Magdalena**] Santa Marta; 1866; Stevens leg.; Type Neur.: No. 10, *MantispaTrichosceliafasciatella* Westw., HOPE Dept. Oxford; Holotype ♀, *Trichosceliafasciatella* Westwood, 1867, ascertained by R.G. Beard, 1968; Type Westwood, Trans. Ent. Soc. 1867, p. 503, Coll. Hope Oxon; OUMNH.

##### Other material.

Colombia – **Bolívar** • 1 ♂; Mompox; 09°14'N, 74°25'W; 33 m; Dec. 1994; MEFLG. • 1 ♂; Zambrano, Hda. Monterrey; 9°45'N, 74°49'W; 70 m; 14 Aug. 1993; F. Fernandez; Malaise No. 6, Lata suelo; ICN. – **Cesar** • 1 ♀; Chiriguana District, lake Sapatoza región; 08 Sep. 1924; C. Allen, BMNH(E) 1201805, Brit Mus. 1925-576, NHMUK 013802797 *Trichosceliafasciatella* Westwood; NHMUK. • 1 ♂; same data collection as for preceding; BMNH(E) 1201814, Brit. Mus. 1925-576, NHMUK 013802798, *Trichosceliafasciatella* Westwood; NHMUK. • 1 ♂; Colombia; Mar. 1968; Saunders leg.; BMNH(E) 1201812, NHMUK 013802800; NHMUK. – **Magdalena** • 1 ♀; Aracataca; 21 Apr.; Darlington leg.; MCZ • 1 ♀; Neguanje, PNN Tayrona; 11°20'N, 74°02'W; 10 m; 21 Feb.–5 Mar. 2001; R. Henriquez leg.; Malaise m–1351; ICN. – **Santander** • 1 ♀; Puerto Parra, Campo Capote; 06°37'08.6"N, 73°54'30"W; 146 m; C. Sarmiento leg.; light trap; ICN.

Panama – **Canal Zone** • 1 ♂; Albrook Forest Site; 30.40 m; 25–26 Jul. 1968; R. S. Hutton leg.; black light trap; UAAM • 1 ♂; same data as for preceding; 13–14 Jul. 1967; Hutton & Llaurudo leg.; UAAM • 2 ♂; same data as for preceding; 24–25 Aug. 1967; Hutton & Llaurudo leg.; black light trap; UAAM • 1 ♂; same data as for preceding 29 Feb.–1 Mar. 1968; R.S. Hutton leg.; UAAM • 2 ♂; same data as for preceding; 3–4 Aug. 1967; Hutton & Llaurudo leg.; UAAM. – **Darién** • 1 ♂; Patino R. Pan; 18 Jul. 1952; F.B. Blanton leg.; det. L.A. Stange; FSCA. – **Panamá** • 1? 1♂; 7–10 Km. N El Llano; 14–22 May. 1993; E. Giesbert leg.; det. L.A. Stange; FSCA • 1 ♂; same data as for preceding; 16–22 May. 1987; E. Giesbert leg.; FSCA • 1 ♂; same data as for preceding; B-10 Km N. El Llano; 26 Apr.–4 May. 1992; E. Giesbert leg.; FSCA • 1 ♂; same data as for preceding; 8–10 km N El Llano; 927.696, -7.896.416; 26 Apr.–04 May. 1992; E. Giesbert leg.; TAMUIC • 2 ♂, 7.5–13 Km El Llano; 21–22 May. 1994; F.T. Hovare leg; CAS • 1 ♂; Panama; 1924; L.E. Cheesman leg.; BMNH(E) 1201813, St. George Exp. B.M. 1925-573, NHMUK 013802799, *Trichosceliafasciatella* Westwood; NHMUK. – **Panamá Oeste** • 1 ♂; Barro Colorado Island; 18 Apr. 1874; Duckworth leg.; USNM • 2♂; Barro Colorado Island; 3–9 Apr. 1985; H. Wolda leg.; Ex. Blacklight; UCD • 1 ♂; Barro Colorado Island; 17–23 Apr. 1985; H. Wolda leg.; UCD.

##### Diagnosis.

This species is easily recognized by its general body color pattern with orange and dark brown or blackish brown. The forefemur is completely orange, and the forewing has medial and apical, broad, dark amber bands which easily separate *A.fasciatellus* from *T.latifascia*. On the hind wing, this species has the intracubital crossvein subparallel to the longitudinal wing axis. On the male genitalia, the gonocoxite IX is long and sinuous, with posterior apex thickened, laterally curved, and equipped with 8–13 apical processes arranged as a brush. On the female genitalia, the gonapophyses VIII medial part is narrow, short, and boat-shaped, with a blunt, dorsally curved process. The spermatheca is short and simple; on the medial section a transparent, digitiform diverticulum is present, and the distal section is progressively expanded and sac-like. The fertilization canal duct is long, spiral-shaped, and the fertilization canal is short, and reniform.

##### Description.

***Measurements*.** Male (*n* = 8). Forewing length: 9.63–13.56 mm; Hind wing length: 7.4–10.65 mm. Female (*n* = 1): Forewing length: 11.43 mm; Hind wing length: 8.75 mm.

**Figure 8.**
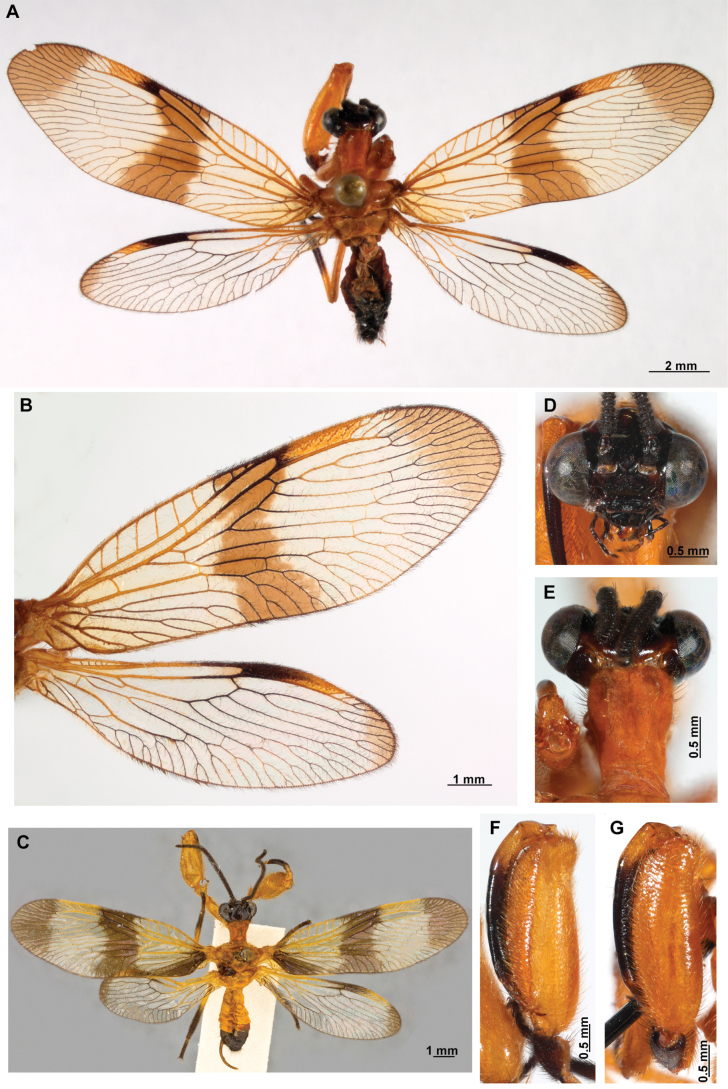
*Anchietafasciatellus* (Westwood, 1867) **A** male habitus, dorsal **B** wings **C** head, dorsal **D** pronotum, dorsal **E** forefemur, anterior surface **F** forefemur, posterior surface.

***Coloration* (Fig. 8). *Head*.** Dark reddish brown to black, vertexal region with lateral rows of black setae; postgena dark reddish brown. Antenna black. Mandible with basal ½ black, distal ½ amber; maxillary palpus black; labium dark reddish brown, palpimacula brown. ***Thorax*.** Pronotum orange, with interspersed black and orange setae. Episternum and postfurcasternum orange. Meso- and metanotum orange, with interspersed orange, brown and black setae; pteropleura completely orange. ***Foreleg*.** Coxa completely orange; trochanter orange or dark reddish brown ventrally and brown dorsally, with concolorous setae. Femur orange; tibia with basal ¼ orange, the rest brown to dark reddish brown, apex generally brown, clavate setae yellowish; basitarsus amber with black tip; second to fourth tarsomere pale brown. ***Mid- and hind legs.*** Mid-leg with coxa, trochanter and femur orange; tibia bicolor, basal ½ orange, distal ½ dark reddish brown to black; tarsus dark reddish brown, ventrally with rows of black setae on the distal margin. Hind leg with coxa, trochanter and femur orange; tibia with basal ¼ orange, the rest dark reddish brown to black; tarsus dark reddish brown, ventrally with rows of black setae. ***Wings*.** Forewing pale orange, dark amber bands at mid length and apex; pterostigma varying with blackish amber from proximal margin to the middle, the rest orange. Venation mainly orange, brown on middle and apical ¼. Hind wing pale orange, subcostal space apex and wingtip amber; pterostigma bicolor, proximal ½ to ¾ blackish amber, orange on the rest. Venation mainly brown, basal ½ of C, Sc, RA, RP veins, M base and basal ½ of Cu orange; apex of C+Sc and RA beyond pterostigma orange. ***Abdomen*.** Basal ½ of the abdomen orange; tergite V bicolor, orange on proximal region, dark brown on region; tergites of abdominal segments VI to ectoproct black; sternites of abdominal segments VI–IX black.

***Morphology* (Fig. 8). *Head*.** Diamond-shaped in frontal view, smooth, vertexal region domed over compound eyes, paraocular area concave; coronal suture discrete. Compound eye hemispheric, as wide as 1/2 of interocular distance at toruli level. Antenna moniliform, flagellum with 58–63 flagellomeres, discoidal in shape, those of the distal ½ wider, except the apical ones, narrow; all flagellomeres with medial ring of short setae. ***Thorax*.** Pronotum slightly longer than wide, with shallow medial concavity; in lateral view, posterior region slightly elevated, entire surface with interspersed thin and thick setae that arise flush the pronotal surface. Postfurcasternum semicircular. Mesonotum ~ 2× as wide as long, with a few fine setae; metanotum ~ 3× as wide as long, mostly glabrous. Pteropleura covered with few fine setae. ***Foreleg*.** Coxa slightly shorter than femur, cylindrical, slightly expanded on preapical region, with fine and short setae; trochanter subtrapezoidal, setose, anterior surface with conspicuous, blunt process; femur robust, densely covered with short and thin setae; closing surface with posteroventral row of integumentary specializations fully developed, composed of thickened tubercle-shaped processes with conical Stitz organs, proximally with a more developed sub-basal, spine-shaped process; adjacent row of thickened setae with globular base reduced to a single apical seta; anteroventral row of integumentary specializations reduced to proximal region and apex, composed of tubercle-shaped processes with conical apical setae; primary process present, spine-shaped, curved; adjacent row of thickened setae with globular base present on distal ¾. Tibia almost as long as femur, curved, glabrous; closing surface with a row of prostrate setae; with a patch of clavate setae apically on anterior surface. Basitarsus with long lanceolate process; proximal ½ with a patch of clavate setae on anterior surface, ventrally with a row of prostrate setae. ***Mid and hind legs*.** Mid-leg covered with abundant fine setae, tibia unmodified, thin. Hind leg covered with fine setae, tibia unmodified, only slightly thickened. ***Wings*.** Forewing elongated and narrow, venation setose, trichosors present along wing margin except at base; costal space slightly widened medially, humeral vein sometimes forked, 7–13 subcostal veinlets, usually simple, a single forked; pterostigma rectangular, 3.5× wider than long; subcostal space with single, medially located crossvein; Sc bent posteriad at proximal margin of pterostigma to merge the RA; *rarp2* straight, with three or four veins arising from it, two or three from *rarp1*; M fused basally to R; RP base near separation of M and R, M forked slightly beyond R fork; 1r-m located between RP base and M base, forming a trapezoidal cell; 4–7 gradate crossveins present; Cu deeply forked, CuP basally angled, approaching A1, forked proximally at level of 1m-cu; A1 distally forked, A2 forked at level of CuP angle. Hind wing smaller and narrower than forewing; costal space narrow and reduced, with 4–9 veinlets; C and Sc fused at proximal 1/3 of wing length, Sc vein strongly curved posteriad at proximal margin of pterostigma to merge the RA; subcostal space without crossveins; pterostigma elongated, narrow; radial space with straight crossvein; three veins arising from *rarp1*, two to three from *rarp2*. M vein forked beyond R fork; Cu deeply forked; CuA sinuous, distally forked; CuP distally gently anteriorly curved, with three or four branches; A1 arched, A2 short, forked. ***Abdomen*.** Medially widened, keeled processes on tergites absent; tergites with abundant setation towards the abdominal apex; sternites setose.

**Figure 9.**
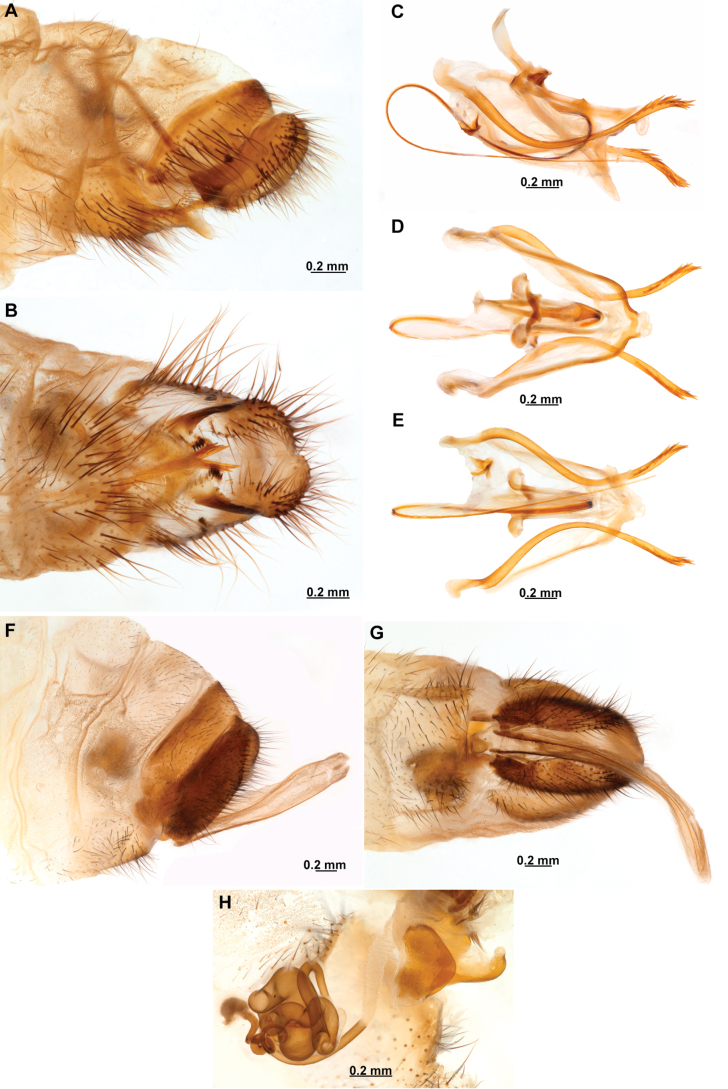
*Anchietafasciatellus* (Westwood, 1867) **A** male terminalia, lateral **B** same, ventral **C** male genitalia, lateral **D** same, dorsal **E** same, ventral **F** female terminalia, lateral **G** same, ventral **H** spermatheca and gonapophyses VIII.

***Male genitalia*** (Fig. 9A–E). Tergite IX slightly narrower medially than laterally, laterally with anterior ½ sclerotized, with abundant, prominent, long, and thick setae, posterior ½ semi-membranous and depigmented. Sternites VIII and IX fused, fusion line distinct, sternite VIII with abundant prominent, long, thick setae. Sternite IX pentagonal in ventral view, with indented lateral margins, with lateral, oblique rows of long, thin setae; in lateral view curved ventrally; gonocoxites IX sinuous, long, base spatulate, curved; gradually thicker towards apex, where it is laterally curved, and equipped with 8–13 apical processes arranged as a brush. Ectoproct, anterior and posterior margins subparallel-sided, posterior ½ covered with prominent, long, and thick setae; ventrally on inner surface a sclerotized region, covered with ~ 15 stout setae, is present. Gonocoxites X thin, anterior apex spatulate, dorsally bent; posterior apex with short lateral and dorsal processes; gonostyli X thin, concave in lateral view, the rest of the structure whip-shaped, recurved. Gonapophyses X straight, narrow, anterior apex spatulate, bent dorsolaterally; gonapophyses subparallel, joined by membrane. Gonocoxites XI V-shaped in ventral view, medial lobe slightly sclerotized, rounded, bent ventrally; anteromedially sclerotized, with two plates fused to form a bridge; lateral arms of gonocoxites XI thin, straight. Hypandrium internum triangular, keeled, with lateral fins.

***Female genitalia*** (Fig. 9F–H). Sternite VII quadrangular, with straight posterior margin; gonocoxites VIII as narrow arc-shaped sclerite; gonapophyses VIII medial part narrow, short, boat-shaped, with a blunt, dorsally curved process; lateral part of gonapophyses as a broad, trapezoidal plate, hidden under the tergite IX + ectoproct. Tergite VIII almost as wide medially as laterally, enclosing the segment VIII spiracle; lateral margin subtrapezoidal. Tergite IX + ectoproct unpaired, forming an elongated, ring-shaped sclerite, laterally wider than medially, lateral margin quadrangular. Gonocoxites IX elongated, curved, narrow, as long as the last four abdominal segments together. Gonapophyses IX as two small, setose sclerites, located behind base of gonocoxites IX. Bursa copulatrix funnel-shaped, short, narrow, membranous, striated. Spermatheca short and simple; proximal section long, thin, with a few convolutions; medial section thicker than proximal section, forming three convolutions, with transparent, digitiform diverticulum; distal section markedly wider than medial section, progressively expanded towards apex, sac-like. Fertilization canal duct long, spiral-shaped, forming four convolutions; fertilization canal short, reniform, covered with microfilaments.

##### Distribution.

Colombia (Bolívar, Cesar, Magdalena, Santander), Panama (Canal Zone, Darien, Panamá, Panamá Oeste).

##### Remarks.

This is a rather striking species of *Anchieta* previously known from Colombia and Panama (Ardila-Camacho et al. 2018). In this study, the first record for the Colombian department of Cesar is presented. In the original description by Westwood (1867), the type locality was specified as Sactam Martham, Venezuela, but the type actually has labels with “Sanctam Martham, Bolivia” as collecting information. This led to a great confusion that was clarified by Ardila-Camacho et al. (2018) and Tauber et al. (2019), who indicated that this locality actually corresponds to the city of Santa Marta (Magdalena), which is located in the Caribbean coast of Colombia.

In the phylogeny of Symphrasinae, this species, which is markedly dissimilar from the rest of the species of the genus, was recovered as sister of all other *Anchieta*. Although it is similar to *T.latifascia*, both can be easily separated by observing the generic characters of each genus, particularly the structure of the foreleg and the wing venation. Additionally, there are marked differences in the mimicry pattern of both species. As discussed by Ardila-Camacho and García (2015) and Ardila-Camacho et al. (2018), this species has a mimicry pattern resembling wasps of the family Braconidae, which have defensive repugnant glands on the abdomen. Furthermore, the striking coloration with orange and black or dark brown bands or spots may serve as a defensive signal against potential predators, as these combinations of colors are typical of aposematic species.

#### ﻿﻿ Anchieta fumosellus

Taxon classificationAnimaliaNeuropteraRhachiberothidae

(Westwood, 1867)

Figs 10 , 11

Mantispa (Trichoscelia) fumosella Westwood, 1867: 504. Holotype: male, Amazonia (OUMNH), specimen examined.
Anisoptera fumosella (Westwood, 1867). Gerstaecker (1888).
Trichoscelia fumosella Westwood, 1867. Enderlein (1910).
Anchieta fumosella (Westwood, 1867). Penny (1982a).
Anchieta nobilis Navás, 1909: 484, male, female. Lectotype, sex not indicated, Brazil (MNHN). Synonymized with Anchietafumosella by Penny (1982a): 4019, High resolution images examined.
Trichoscelia nobilis (Navás, 1909). Enderlein (1910).

##### Material examined.

Holotype of Mantispa (Trichoscelia) fumosella.

[Brazil] • ♂; “*fumosella* Westwood, male”, “*Platymantispafumosella* (Westwood, 1867)”, “Holotype male, *Trichosceliafumosella* Westwood, 1867, R.G. Beard 1968, now belongs in genus *Platymantispa* Rehn”, “Type Westwood, Trans. Entomol. Soc. 1865, p. 504, Coll. Hope Oxon”, “Holotype male, Genitalia of *Trichosceliafumosella* Westwood, 1867, prep. R.G. Beard, 1968”, “over? fumosella Westw.”, “Type Neur: No. 13, *MantispaTrichosceliafumosella* Westw. HOPE Dept. Oxford”; Terminalia cleared and stored in a microvial; OUMNH.

***Lectotype*** of *Anchietanobilis*.

Brazil • ♂; **Goiás**, Jataí; Sep.–Nov. 1897; *Anchietanobilis* Navás, Longin Navas det. 1907; Lectotype ♂ designated by R.G. Beard, 1968; MNHN.

***Paralectotype*** of *Anchietanobilis*.

Brazil • ♀; **Goiás**, Jataí; *Anchietanobilis* Navás, Longin Navas det. 1907; Lectoallotype ♂ designated by R.G. Beard, 1968; MNHN.

##### Other material.

Brazil – **Bahia** • 2 ♂; Encruzilhada; 15°32'25"S, 40°50'12"W; 800 m; 10–12 Dec. 2007; J.A. Rafael, P.C. Grossi, D.R. Parizotto leg.; light trap; *Anchietafumosella* (Westwood, 1867), det. R.J.P. Machado; INPA. • 1 ♂; Encruzilhada; 960 m; Nov. 1972; Seabra and Alvarenga leg.; DZUP. – **Minas Gerais** • 1♀; Feb. 1932; R. Bandens, J. Blaser leg.; MCZ. • 1 ♂; same data as for preceding, Passos; Mar. 1961; C. Elias leg.; det. L.A. Stange; FSCA. – **Paraná** • 1 ♀; São José dos Pinhais; 25°36'18"S, 49°11'37"W; 1–30 Nov. 2019; A.C. Domahovski leg.; malaise; DZUP. • 1 ♀; same data as for preceding; 17–31 Dec. 2016; A.C. Domahovski leg.; sweep; DZUP. • 1 ♂; Campo Largo, Estrada do Cerne, Km 45; 22 Nov. 1979; Expedition Dep. Zoo UFPR leg.; DZUP. • 1 ♀; Ponta Grossa, Pq. Est. Vila Velha; 25°14'S, 49°59'W; 23 Nov. 2001; G.A.R. Melo; DZUP. – **Rio de Janeiro** • 1 ♂; Nova Friburgo, Sans Souci; 1050 m; Nov. 2004; P. Grossi leg.; light; coleçao E. & P. Grossi; *Anchietafumosella* (Westwood, 1867), det. R.J.P. Machado; INPA. • 1 ♂; Itatiaia, P. N. Itatiaia, casa pesquisador; 22°27'21"S, 44°36'30"W; 05 Dec. 2015; A.P.M. Santos and D.M. Takiya leg.; light trap; DZUP. – **Santa Catarina** • 1 ♀; Blumenau; F. Müller leg.; McLachlan Coll. B.M.; NHMUK, 1938-674, BMNH(E)-1241392. • 1 ♀; same data as for preceding; Blumenau, Virgil; 1987; v.d. Weele leg.; det. L.A. Stange; FSCA. • 1 ♂; Nova Teutonia; 27°11'S, 58°23'W; 300–500 m; Jan. 1971; F. Plaumann leg.; INPA. – **São Paulo** • 1 ♀; Jardim Botanico; 18 Dec. 1987; R.L. Jeane leg.; MCZ. • 1 ♂ 1 ♀; Same data as for preceding; Luiz Antonio, Estação Ecológica Jataí; 21°36'47"S, 47°45'04"W; 01 Oct. 2008; R. Lara leg.; light trap; DZUP. • 2 ♀; same data as for preceding; 16 Sep. 2009, Lara and team leg; Malaise; DZUP. • 2 ♂; same data as for preceding; 13 Feb. 2008; R. Lara and team leg.; light trap; DZUP. • 2 ♂; same data as for preceding; 12 Mar. 2008; R. Lara and team leg.; DZUP. • 1 ♂; same data as for preceding; 17 Sep. 2008; R. Lara and team leg.; DZUP. • 3♂ 1♀; same data as for preceding; 01 Oct. 2008; R. Lara and team leg.; DZUP. • 2 ♂; same data as for preceding; 29 Oct.2008; R. Lara and team leg.; DZUP. • 3 ♂; same data as for preceding; 27 Sep. 2008; R. Lara and team leg.; DZUP. • 2 ♂ 3 ♀; same data as for preceding; 28 Jan. 2009; R. Lara and team leg.; DZUP. • 5 ♂; same data as for preceding; 28 Feb. 2009; R. Lara and team leg.; DZUP. • 1 ♂ 1 ♀; same data as for preceding; 18 Mar. 2009; R. Lara and team leg.; DZUP. • 2 ♂; same data as for preceding; 01 Apr. 2009; R. Lara and team leg.; DZUP. • 1 ♀; same data as for preceding; 09 Apr. 2009; R. Lara and team leg.; DZUP. • 1 ♂; same data as for preceding; 15 Apr. 2009; R. Lara and team leg.; DZUP. • 1 ♂; same data as for preceding; 29 Apr. 2009; R. Lara and team leg.; DZUP. • 1 ♂; same data as for preceding; 27 May. 2009; R. Lara and team leg.; DZUP. • 1 ♂; same data as for preceding; 30 Sep. 2009; R. Lara and team leg.; DZUP. • 1 ♂; same data as for preceding; 15 Oct. 2009; R. Lara and team leg.; DZUP. • 2 ♂; same data as for preceding; 29 Oct. 2009; R. Lara and team leg.; DZUP. • 1 ♀; same data as for preceding; 08 Dec. 2009; R. Lara and team leg.; DZUP. • 1 ♂; São Luiz Paraitinga, PESM nucleo St Virginia; 22°19'18"S, 45°05'43"W; 21 Nov. 2011; N.W. Perioto and team leg.; Malaise (7); DZUP.

##### Diagnosis.

The body coloration pattern of this species may be yellow with dark brown stripes or nearly completely dark brown. The forewing membrane is pale amber, with darker area on distal ½ of costal field, subcostal space, radial space, area between R+M and CuP, and area adjacent to RP. The hind wing is short and narrowly oval. On the male genitalia, the tergite IX has a posterolateral tuft of long setae, which surpasses the posterior margin of ectoproct. The sternite IX is blunt, sclerotized, with lateral margins concave. The gonocoxite IX is thin, short, and sinuous, with posterior apex blade-shaped, with sharp tip, sometimes slightly lanceolate, or with two or three tiny, preapical processes. On the female genitalia, the gonapophyses VIII form a posteromedially projected, tubular process with blunt to pointed apex.

##### Description.

***Measurements*.** Male (*n* = 5). Forewing length: 11.0–12.8 mm; Hind wing length: 6.6–8.57 mm. Female (*n* = 3): Forewing length: 7.7–12.15 mm; Hind wing length: 4.6–7.63 mm.

**Figure 10.**
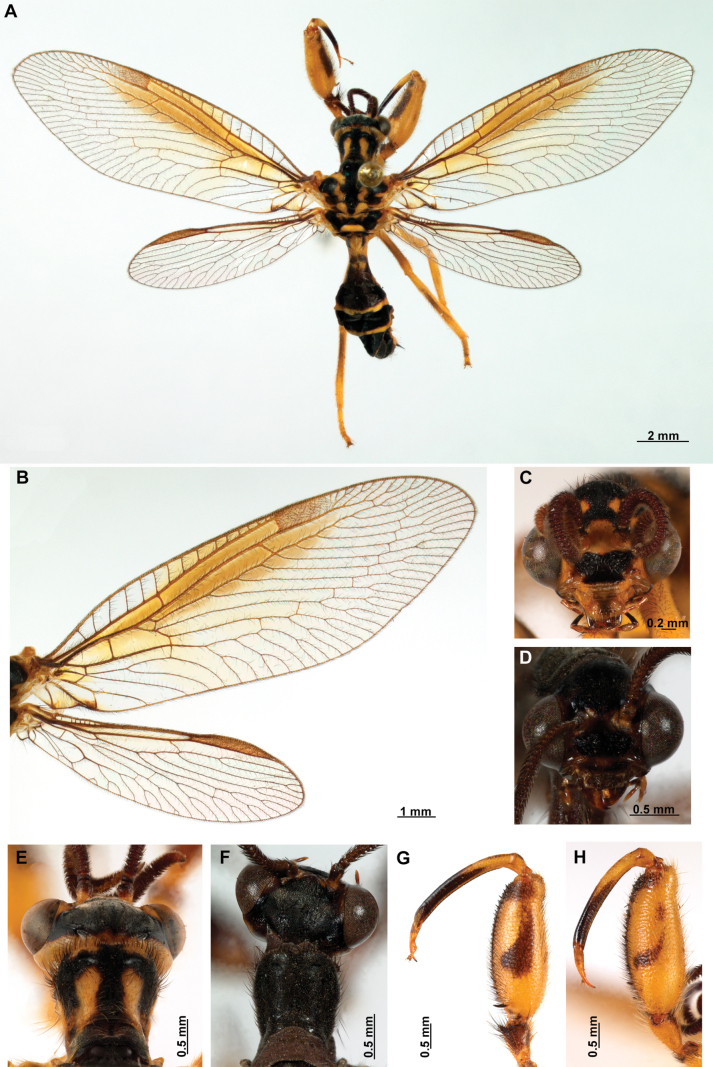
*Anchietafumosellus* (Westwood, 1867) **A** male habitus **B** wings **C** head (yellow morph), frontal **D** head, frontal (dark morph) **E** pronotum (yellow morph), dorsal **F** pronotum (dark morph), dorsal **G** forefemur, anterior surface **H** same, posterior surface.

***Coloration* (Fig. 10). *Head*.** Mostly black, vertexal region with small pale brown mark above compound eye; with lateral rows of dark brown setae. Postgena with the inner ½ dark brown, outer ½ yellowish with dark brown suffusions. Antennal scape pale brown ventrally, dorsally dark brown, pedicel dark brown; flagellum brown to dark brown, except for ~ 8–10 yellow apical flagellomeres, some specimens with flagellum completely dark; toruli surrounded by pale brown. Clypeus with dark amber posterolateral corners; labrum with pale brown margins; mandible dark amber; maxillary palpus brown; labium black except ligula, pale brown, labial palpus brown, palpimacula pale. ***Thorax*.** Pronotum black. Episternum brown, postfurcasternum dark brown. Meso- and metanotum blackish brown, with pale brown setae; pteropleura mostly blackish brown, with orange areas on anepisterna, with brown setae. ***Foreleg*.** Coxa mostly dark reddish brown with yellowish area at apex; trochanter dark brown with pale brown ventral band. Femur mostly brown with paler areas on outer surface and small pale brown area dorsally at level of anteroventral primary process; tibia mostly dark reddish brown, with pale brown basal area, clavate setae pale; basitarsus pale amber with amber apex, second to fourth tarsomere pale brown. ***Mid- and hind legs.*** Mid-leg with brown coxa and trochanter, femur, and tibia pale brown at base and apex, the rest of the surface brown; tarsomeres completely pale brown. Hind leg with coxa, trochanter and femur brown, with interspersed pale and dark brown setae; tibia mostly pale brown with darker median area; tarsus pale brown. ***Wings*.** Forewing membrane pale amber, with darker area on distal ½ of costal field, radial space, subcostal space, area between R+M and CuP, and area adjacent to RP; with small dark amber spot posteriorly on anal region; pterostigma pale brown with pale posterior and distal margins; venation mostly brown with R+M, proximal ½ of CuA and A3 darker. Hind wing pale amber, base of subcostal space and anterior ½ of *ra-rp1* slightly darker; pterostigma amber or pale brown, venation brown. ***Abdomen*.** Dark brown, with pale brown setae.

***Chromatic variation*** (Fig. 10). ***Head*.** Mainly yellow, vertexal region with dark brown posterior transverse band and arc-shaped marking on frontal sutures; frons dark brown. Antennal scape bicolor, yellow on proximal ½, dark brown on distal ½; flagellum yellow to pale brown, setae pale to dark brown. Labrum pale brown, clypeus yellow; mandible yellow with pale amber apex; maxillary palpus amber; labium bicolor, yellow on mentum and brown on ligula, labial palpus amber. ***Thorax*.** Pronotum brown with lateral yellow stripes; episternum pale brown, with yellow margins; postfurcasternum pale brown medially, yellow on margins. Mesonotum dark brown with yellow lateral stripes; metanotum dark brown with medium yellow areas; pteropleura yellow with longitudinal brown bands, setae pale yellow. ***Foreleg*.** Coxa mostly yellow, with brown areas on basal ½; trochanter bicolor, yellow ventrally, brown dorsally. Femur mostly yellow, anterior surface with irregularly shaped brown marking extending from center to apex; posterior surface with a brown J-shaped marking on medial region; tibia yellow on basal ½, brown to dark brown on distal ½; basitarsus pale amber, remaining tarsomeres yellow. ***Mid and hind legs*.** Mid-leg mostly yellow with dark brown spots on trochanter and tibia. Hind leg with coxa bicolor, trochanter brown, femur mostly brown with yellow base and apex; tibia mainly yellow with brown posteromedian region. ***Abdomen*.** Brown, with yellow anterior and posterior margins of sclerites.

***Morphology*** (Fig. 10). ***Head*.** Diamond-shaped in frontal view, smooth, vertexal region domed above compound eyes; coronal suture discrete; paraocular area concave. Antenna moniliform, flagellum with 43–61 flagellomeres, discoidal in shape, those of distal ½ wider, except at apex narrower; all flagellomeres with medial ring of short setae. Compound eye hemispherical, as wide as ½ of interocular distance at toruli level. ***Thorax*.** Pronotum nearly as long as wide, with groove contiguous to lateral and distal margins; in lateral view, posterior margin slightly raised, the rest of the surface straight; entire surface with scattered, thick setae, arising flush the pronotal surface. Postfurcasternum quadrangular. Mesonotum slightly wider than long, with a few scattered fine setae, metanotum ~ 3× as wide as long, mostly glabrous covered with microtrichia. Pteropleura covered with abundant fine and long setae. ***Foreleg*.** Coxa as long as femur, cylindrical, slightly expanded at preapical region, with abundant, fine and long setae; trochanter subtrapezoidal, densely setose, dorsally with a tuft of long and thick setae, anterior surface with blunt process. Femur robust, with abundant fine, long setae; closing surface with posteroventral row of integumentary specializations fully developed, composed of tubercle-shaped processes with conical Stitz organs, proximally with a more developed sub-basal, spine-shaped process; adjacent row of thickened setae with globular base reduced to distal ¾ or ½; anteroventral row of processes reduced to proximal region and apex, composed of tubercle-shaped processes, with conical setae; basal primary process present, spine-shaped, curved; adjacent row of thickened setae with globular base present on distal 4/5. Tibia almost as long as femur, curved, glabrous, closing surface with a row of prostrate setae; apical region of anterior surface with a patch of clavate setae. Basitarsus with long lanceolate process; basal ½ with clavate setae on anterior surface, ventrally with a row of prostrate setae. ***Mid- and hind legs.*** Mid- and hind leg covered with abundant fine setae; tibia unmodified, thin. ***Wings*.** Forewing oval, venation setose, trichosors present along wing margin except at base; costal space slightly widened medially, humeral vein sometimes forked, with 13–18 subcostal veinlets; pterostigma rectangular; subcostal space with single medially located crossvein; Sc vein abruptly bent posteriad at proximal margin of pterostigma to merge the RA; *rarp2* straight with 3–6 veins arising from it, three from *rarp1*; M fused basally with R; RP base located near separation of M and R, M fork near such separation; 1r-m located between RP base and M fork, forming a small trapezoidal cell; 6–8 gradate crossveins present; Cu deeply forked, CuA apically branched; CuP proximally angled, approaching A1, forked slightly beyond 1m-cu; A1 simple, A2 forked. Hind wing notably smaller and narrower than forewing; costal space narrow and reduced, with seven or eight subcostal veinlets; C and Sc fused at 1/3 of wing length; Sc vein abruptly curved posteriad at proximal margin of pterostigma, to merge the RA; subcostal space without crossveins; pterostigma elongated, narrow; radial space with single sinuous crossvein; two or three veins arising from *rarp1*, 1–3 from *rarp2*. M vein forked at level of R fork; Cu deeply forked, CuA gently bent, distally forked, first branch forked; CuP distally gently bent anteriorly, near posterior wing margin, with two or three branches; CuP sometimes fused to posterior margin, and then diverges again forming an arch; A1 arched, A2 short, simple. ***Abdomen*.** Medially widened, tergites without keeled processes.

**Figure 11.**
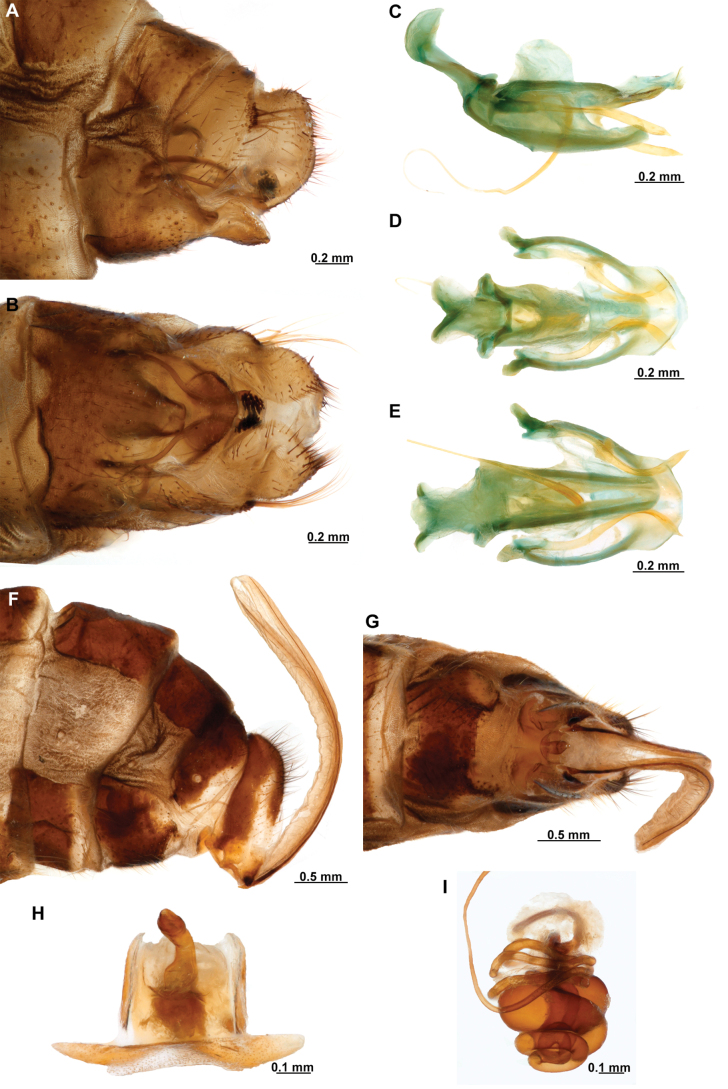
*Anchietafumosellus* (Westwood, 1867) **A** male terminalia, lateral **B** same, ventral **C** male genitalia, lateral **D** same, dorsal **E** same, ventral **F** female terminalia, lateral **G** same, ventral **H** gonapophyses VIII of female, ventral **I** spermatheca.

***Male genitalia*** (Fig. 11A–E). Tergite IX notably narrower medially than laterally, lateral region with posteromedial tuft of long, thick setae, which surpass posterior margin of ectoproct. Sternites VIII and IX fused, fusion line moderately distinct; sternite IX posteroventrally projected, blunt, sclerotized, lateral margins concave. Gonocoxites IX thin, sinuous, as long as gonapophyses X, posterior apex curved laterally, flattened, blade-shaped, with sharp tip, sometimes slightly lanceolate, or with two or three tiny preapical processes. Ectoproct ovoid, with subparallel anterior and posterior margins; ventrally on inner surface with a more sclerotized region covered with 25–29 thick, conical setae on posterior ½ and thin, short setae on anterior ½. Gonocoxites X ventrally canaliculated, with anterior apex expanded and dorsally bent, posterior region with lateral and dorsal processes; base of gonostylus X thickened, triangular, concave in lateral view, the rest of the structure whip-shaped, short, with apical portion recurved. Gonapophyses X straight, narrow, with spatulate and dorsally recurved tips; gonapophyses joined by membrane, forming a V-shaped structure. Gonocoxites XI narrow, U-shaped, medial lobe expanded, composed of two concave plates medially joined by narrow bridge; lateral arms slightly arched, anterior apex curved.

***Female genitalia*** (Fig. 11F–I). Sternite VII subrectangular, with the posterior margin broadly concave, densely setose towards posterior margin. Tergite VIII slightly narrower medially than laterally, enclosing the spiracle of the segment, lateral margin trapezoidal. Gonocoxites VIII as two medially fused, trapezoidal, concave plates; gonapophyses VIII as a convex plate, posteromedially projected, forming a tubular process with blunt to somewhat pointed apex; lateral part of gonapophyses VIII as a trapezoidal plate hidden by the tergite IX + ectoproct. Tergite IX + ectoproct elongated, lateral margin quadrangular. Gonocoxites IX elongated, sinuous, and narrow, as long as the last five abdominal segments together. Gonapophyses IX reduced to a pair of tiny sclerites hidden by membranes, situated behind the gonocoxites IX base. Bursa copulatrix narrow, short, membranous. Spermatheca complex and coiled; proximal section long and thin, forming four coils; medial section thicker than proximal section, short, forming a couple of convolutions; distal section wider than medial section, progressively expanded towards apex, sac-like, forming a convolution; fertilization canal duct, long, spiral-shaped, with four or five convolutions, covered by the proximal section of the spermatheca; fertilization canal elongated, thin, J-shaped, covered with microfilaments.

##### Distribution.

Brazil (Bahía, Distrito Federal, Goiás, Minas Gerais, Rio de Janeiro, Paraná, Santa Catarina, Rio Grande do Sul, São Paulo, Tocantins).

##### Remarks.

This species is so far known only exclusively from Brazil and has been previously recorded in the states of Bahia, Distrito Federal, Goiás, Minas Gerais, São Paulo, Rio de Janeiro, Paraná, Santa Catarina, Tocantins, and Rio Grande do Sul (Penny 1982a; Penny and da Costa 1983; Carvalho and Corseuil 1991; Alvim et al. 2019; Schuster and Machado 2021). Herein, most of these records are confirmed based on the material examined. The holotype of Mantispa (Trichoscelia) fumosella lacks specific collecting data, but Westwood (1867) in the original description of the species indicated “Amazonia” as the collecting site. This information was questioned by Penny (1982a) and herein such idea is corroborated, as this species is restricted to Central and Southwestern Brazil. Araújo et al. (2021) reported several specimens reared from artificial nests of bees in the Amazonian portion of the Brazilian state of Mato Grosso which might be *A.fumosellus*, however detailed examination of such material was not possible at present, so the presence of this species in that state will require corroboration (R.J.P. Machado, pers. observation).

*Anchietafumosellus* expresses a Batesian mimicry pattern with wasps of the family Vespidae (Buys 2008; Rasmussen and Ardila-Camacho 2021). Penny (1982a) realized that this species exhibits a considerable variation in the body color pattern. Some specimens can be completely dark brown, other can be yellow with dark brown stripes, while other can have intermediary colorations, tracing different species of vespid wasps as happens in the mantispid genus *Climaciella* Enderlein, 1910 (Rasmussen and Ardila-Camacho 2021). However, the male genitalia and wing coloration and venation between the yellow and dark forms are identical. This led to the synonym of *A.nobilis* (whose type has yellow body with dark stripes) with *A.fumosella* (in which the type has a dark brown body) (Penny 1982a). Herein that hypothesis is confirmed, after a careful examination of the morphology of several specimens of both morphs. Based on the distribution and coloration pattern and variation of this species, the model for the dark morph is likely the wasp *Agelaiavicina* (de Saussure, 1854), whereas the model species for the yellow morph is probably *Agelaiamultipicta* (Haliday, 1836) (R. Lopes, pers. comm.).

In the phylogeny of Symphrasinae, this species was recovered as an intermediary species between the braconid mimicking *A.fasciatellus* and the clade containing all the bee mimicking species. This evolutionary pattern matches with the phylogeny of Hymenoptera, in which the parasitoid wasps of the family Braconidae are part of a large clade that diverged first in the phylogeny of Hymenoptera, then, within Aculeata the Vespoidea had an early divergence, while the bees or Antophila evolved as a highly specialized group within Apoidea (Peters et al. 2017).

This species can be recognized by the pale amber area and the anterior region of the forewing, which resembles the folding of the forewings of vespid wasps, the short and narrow hind wing, and the male genitalia. The male genitalia of this species are similar to that of *A.nebulosus* as both have a tuft of long setae on the lateral region of the tergite IX, arched goncoxites XI, and narrow and short male gonocoxites IX lacking digitiform processes. By contrast, the female goncoxites+gonapophyses VIII are similar to those of *A.fasciatellus*, but the spermatheca is similar to that of the species included within the bee-mimicking clade.

#### ﻿﻿ Anchieta nebulosus

Taxon classificationAnimaliaNeuropteraRhachiberothidae

Ardila-Camacho & Machado sp. nov.

https://zoobank.org/58E7A5FF-CE29-4F76-8A3D-E578EF84B23E

Figs 12 , 13

##### Type locality.

Brazil, **Espírito Santo**: Linhares, Estrada, Fazenda St. Terezinha, 50 m a.s.l., Oct. 2004, P. Grossi leg.

##### Material examined.

***Holotype*** male, pinned, with genitalia in a separate microvial. Original label: “Brazil, **Espírito Santo**, Linhares, Estrada, Fazenda St. Terezinha, 50 m a.s.l., X.2004, P. Grossi leg. (coleção E. & P. Grossi)”; INPA. ***Paratypes*.** Brazil • 1 ♀; **Bahía**; McLachlan leg. B.M.; NHMUK, 1938-674, BMNH(E)-1241393. • 1 ♂; same data as for preceding; Camacã, Reserva Serra Bonita, Fazenda Santa Barbara; 14 Jan. 2007; A. Raw leg.; DZUP-381880.

##### Etymology.

The specific epithet of this species comes from the Latin *nebula* meaning misty or clouded, in allusion to the wing color pattern of this species. An adjective in the nominative case.

##### Diagnosis.

This species has a general body color pattern very similar to that of *A.nothus*. The body is mostly dark brown; the forewing is dark amber at base, and there are irregular amber areas on membrane surrounding 1m-cu, subcostal veinlets of distal ½ of costal space, medial and apical region of subcostal field, 1ra-rp, and part of M fork, rarp1, and 2m-cu base. The hind wing has a broad, amber, transverse band on proximal 1/3 of wing. The tergites of the abdominal segments III–VI have posteromedial, keeled processes, which are not enlarged. The male tergite IX has posterolateral tuft of long setae, which surpass the posterior margin of the ectoproct. The sternite IX is quadrangular with posterior margin ventrally curved. The gonocoxite IX is short, filiform, with posterior apex pointed. The surrounding membrane of the posterior apex of the gonapophyses X is sclerotized and forms a bilobed structure set with minute granules. On the female genitalia, the gonapophyses VIII form a cubic structure, with concave sides, and with an anteromedial incision.

##### Description.

***Measurements*.** Male (*n* = 1). Forewing length: 9.5 mm; Hind wing length: 5.9 mm. Female (*n* = 1): Forewing length: 13.0 mm; Hind wing length: 7.7 mm.

**Figure 12.**
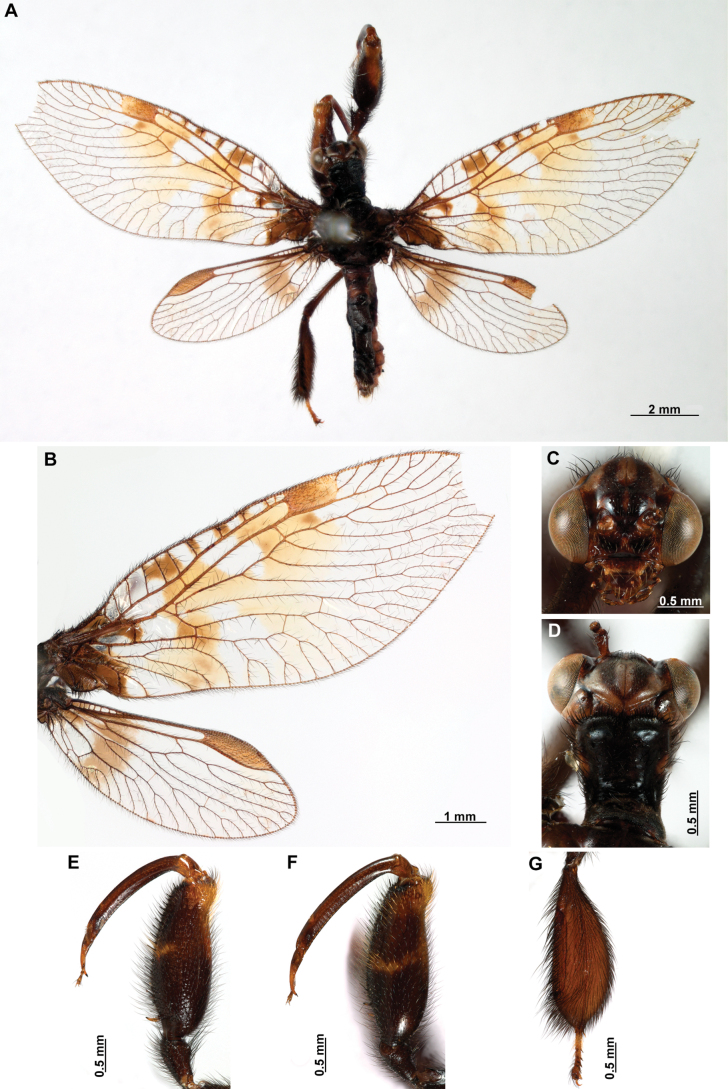
*Anchietanebulosus* Ardila-Camacho & Machado, sp. nov. **A** male habitus **B** wings **C** head, frontal **D** pronotum, dorsal **E** forefemur, anterior surface **F** same, posterior surface **G** hind tibia, inner surface.

***Coloration*** (Fig. 12). ***Head*.** Mostly dark reddish brown, vertex with two dark lateral bands, alternating with paler areas, medially and posterolaterally; with rows of dark brown setae present. Postgena pale brown. Antennal scape and pedicel dark reddish brown, flagellum dark brown, except for three paler preapical flagellomeres. Mandible dark reddish brown; maxillary palpus dark amber; labium brown, palpus dark amber, except on area adjacent to articulations pale brown, palpimacula pale brown. ***Thorax*.** Pronotum dark reddish brown with a pair of pale brown markings on lateral edges, setation black. Episternum and postfurcasternum dark reddish brown. Meso- and metanotum dark reddish brown, mesonotum with black bristles. Pteropleura dark reddish brown. ***Foreleg*.** Coxa dark reddish brown; trochanter dark reddish brown. Femur mostly dark reddish brown with pale brown, medial marking, adjacent to closing surface on anterior and posterior surface; dorsal surface with a large pale brown area at apex; tibia brown, clavate setae pale brown; basitarsus brown, becoming paler towards apex; second to fourth tarsomere brown. ***Mid and hind legs*.** Mid-leg with coxa, trochanter, femur, and tibia dark brown; tarsus pale brown, with dark reddish-brown setae. Hind leg with coxa, trochanter, femur, and tibia brown, with dark brown setae; tarsus pale brown, with brown setae. ***Wings*.** Forewing membrane mainly hyaline, dark amber at base; irregular amber areas on membrane surrounding 1m-cu, subcostal veinlets of distal ½ of costal space, medial and apical region of subcostal field, 1ra-rp, and part of M fork, rarp1 and 2m-cu base; other weakly pigmented areas include posterior radial cells, area between CuP branches, and distal part of cubitoanal space; pterostigma with proximal 2/3 pale brown, distal 1/3 pale yellow; venation mostly brown, becoming paler towards posterior margin. Hind wing mostly hyaline, dark amber at base and with broad, amber, transverse band on proximal 1/3 of wing; basal ½ of subcostal space and *mcu1* amber; pterostigma brown, venation brown, setation dark brown, except on paler apical margin. ***Abdomen*.** Tergites dark reddish brown except on abdominal segments IX and X + ectoproct pale brown, setation dark brown on segments I–VI, pale brown on the rest; sternites pale brown; pleural membrane dark brown.

***Morphology*** (Fig. 12). ***Head*.** Diamond-shaped in frontal view, rugose, vertex domed above compound eyes, paraocular area concave; supra-antennal region with lateral raised, elongate areas; coronal suture distinct. Antenna moniliform, flagellum slightly dorsoventrally flattened, with 47 flagellomeres, discoidal in shape, those of apex narrower; all flagellomeres with medial ring of short setae. Compound eye hemispheric, as wide as 0.5 of the interocular distance at toruli level. ***Thorax*.** Pronotum nearly as long as wide, with groove contiguous to lateral and distal margins; in lateral view, posterior margin slightly raised, the rest of the surface straight; entire surface with abundant long, thick setae arising flush the pronotal surface. Postfurcasternum quadrangular. Mesonotum 1.5× as wide as long, with abundant long, thick setae; metanotum ~ 3× as wide as long, mostly glabrous. Pteropleura covered with abundant long, thin setae. ***Foreleg*.** Coxa as long as femur, cylindrical, slightly distally widened, with abundant fine, long setae; trochanter subconical, densely setose, dorsally with a tuft of long, thick setae, anterior surface with conspicuous, blunt process. Femur robust, with abundant fine, long setae, longer towards ventral surface; closing surface with posteroventral row of integumentary specializations fully developed, composed of tubercle-shaped processes, proximally with a more developed, spine-shaped, sub-basal process, adjacent row of thickened setae with globular base reduced to distal ½; anteroventral row of specializations reduced to proximal region and apex, composed of tubercle-shaped processes, primary, spine-shaped, curved process present, adjacent row of thickened setae with globular base present on distal 4/5. Tibia almost as long as femur, curved, glabrous, closing surface with a row of prostrate setae; anterior surface with patch of clavate setae at apex. Basitarsus with long lanceolate process; anterior surface with patch of clavate setae on basal ½, ventrally with a row of prostrate setae. ***Mid- and hind leg*.** Mid-leg with coxa, trochanter and femur with abundant fine and long setae; tibia slightly medially expanded, with abundant thick, long setae; basitarsus as long as the next three tarsomeres together; all tarsomeres covered with short, thin setae. Hind leg with coxa, trochanter and femur covered with abundant, fine, long setae; tibia notably expanded, laterally flattened, oar-shaped, with abundant long, thin setae; tarsomeres with short, thin setae, basitarsus almost as long as the next three tarsomeres together. ***Wings*.** Forewing oval, venation setose, trichosors present along the wing margin except at base; costal space slightly widened medially, humeral vein sometimes forked, 10–12 subcostal veinlets present, distal veinlets sometimes forked; pterostigma rectangular; subcostal space with single medial crossvein; Sc vein abruptly bent posteriad at proximal margin of pterostigma to merge the RA; *rarp2* straight, with three or four veins arising from it, three or four veins arising from rarp1; M fused basally to R; RP base located near separation of M and R, M fork near such separation; 1r-m located between RP base and M fork forming a small, trapezoidal cell; 4–6 gradate crossveins present; Cu deeply forked, CuP basally angled, approaching A1, forked slightly beyond 1m-cu; A1 simple, A2 forked. Hind wing notably smaller and narrower than forewing, oval; costal space narrow and reduced, 6–10 veinlets; C and Sc fused at proximal 1/3 of wing length; Sc abruptly curved posteriad at proximal pterostigma margin to merge the RA; pterostigma elongated, oval; radial space with single, smoothly sinuous crossvein; two veins arising from *rarp1*, one or two from *rarp2*. M forked oppositely or slightly beyond the level of R fork. Cu vein deeply forked, CuA slightly concave, distally forked, first branch simple; CuP distally, notably, anteriorly bent, near posterior wing margin, with three or four branches; A1 arched, A2 short, simple. ***Abdomen*.** Medially widened, tergites of abdominal segments III–VI with posteromedial, keeled processes, not enlarged. Sternites covered with scattered, long, fine setae.

**Figure 13.**
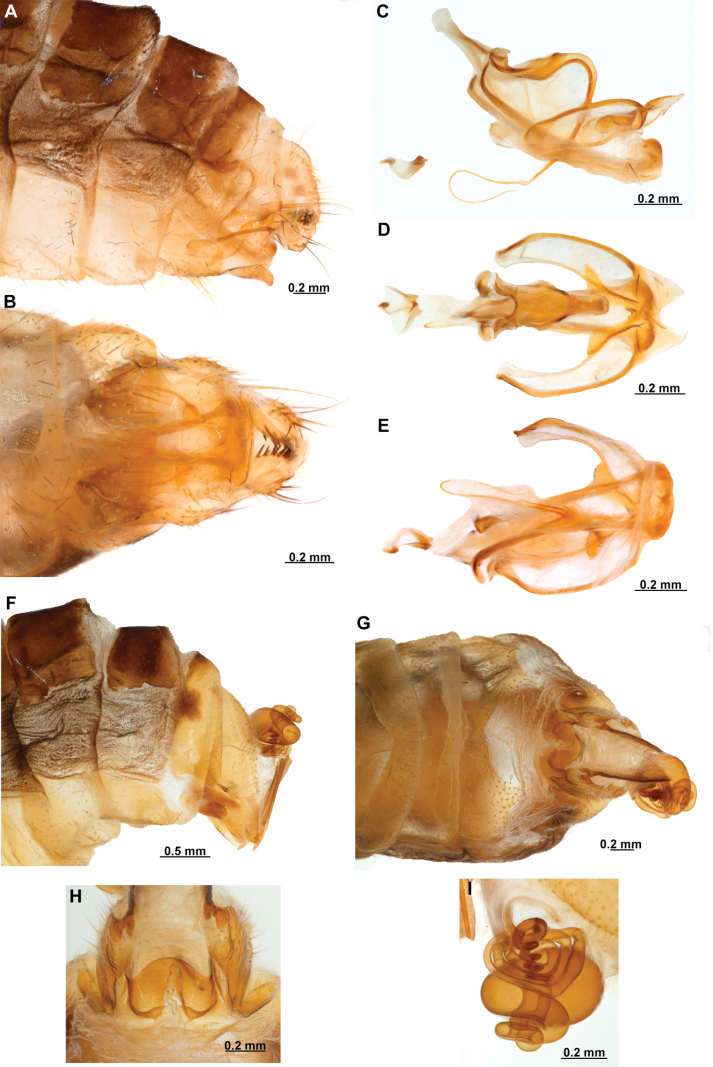
*Anchietanebulosus* Ardila-Camacho & Machado, sp. nov. **A** male terminalia **B** same, ventral **C** male genitalia, lateral **D** same, dorsal **E** same ventral **F** female terminalia, lateral **G** same, ventral **H** gonapophyses VIII of female, ventral **I** spermatheca.

***Male genitalia*** (Fig. 13A–E). Tergite IX notably narrower medially than laterally, lateral margin quadrangular, with posterior, small notch; above this notch a tuft of long, thickened, pedicellate setae, which surpass the posterior margin of ectoproct. Sternites VIII and IX, with fusion line barely perceptible; sternite VIII with abundant long, thin setae on posterior margin; sternite IX quadrangular, lateral margins with anterior slight notch; posterolateral corners rounded with short, thin setae; posterior margin ventrally curved. Gonocoxites IX short, remarkably thin, sinuous, almost as long as lateral arms of gonocoxites XI; base spatulate, rounded; apex pointed, without processes, situated under ectoprocts. Ectoproct ovoid, with short, pedicellate setae, more elongated posteroventrally; ventrally on inner surface with more sclerotized concave region, with patch of short, thickened setae. Gonoxites X forming short, triangular, thickened sclerite; posterior apex with dorsal and lateral processes; gonostyli X with triangular base, with dorsal surface concave, the rest of the structure whip-shaped, short, ventrally curved, with apical portion forming a loop. Gonapophyses X long, parallel, gently curved, with slightly dorsally recurved anterior apex; posterior apex with the surrounding membrane more sclerotized, forming a bilobed structure set with minute granules; gonapophyses joined by membrane. Gonocoxites XI arched, medial lobe composed of two quadrangular, widened plates medially joined; anterior margin with a pair of blunt, curved, projections; lateral arms of gonocoxites XI short, narrow, straight with anterior apex spatulate and rounded. Hypandrium internum arched, keeled.

***Female genitalia*** (Fig. 13F–I). Sternite VII (gonocoxites VII) as two quadrangular, lateral plates, medially fused through a narrow bridge, with concave posterior margin. Tergite VIII slightly narrower medially than laterally, enclosing the segment spiracle, lateral margin quadrangular, notched. Gonocoxites VIII as two lateral, trapezoidal, concave plates; gonapophyses VIII forming a cubic structure, with concave sides, and with an anteromedial incision; lateral part of gonapophyses VIII as a trapezoidal, elongated plate, hidden under tergite IX + ectoproct. Tergite IX + ectoproct triangular. Gonocoxites IX narrow, elongated, as long as the last four abdominal segments. Gonapophyses IX reduced to a pair of tiny sclerites hidden by tergite IX + ectoproct. Bursa copulatrix funnel-shaped membranous, short. Spermatheca tightly coiled; proximal section long, thin, forming three coils on distal part; medial section, short, thicker than proximal section, forming few convolutions; distal section remarkably wider than medial section, strongly expanded towards apex, sac-shaped; fertilization canal duct long, spiral-shaped, with five convolutions, with widened at preapical part; fertilization canal elongated, narrow, J-shaped, covered with microfilaments.

##### Distribution.

Brazil (Bahía, Espírito Santo).

##### Remarks.

This is a new species described from Bahía and Espírito Santo in Brazil. Naked eye, this species could be identified as *Anchietanothus* (Erichson, 1939) as both species have exactly the same general coloration pattern, including the extensive shaded areas on the wings. Nonetheless, the arrangement of the processes on the abdominal terga can be used as a first clue to separate both species. Furthermore, the genital sclerites are completely different, the superficial resemblance between both just represents an additional case of cryptic species, a rather common phenomenon in Symphrasinae. In the phylogeny of the subfamily, this species was recovered as sister of *A.nothus* within the clade of bee mimicking species of *Anchieta*. Regarding the mimicry pattern, this species appears to mimic the genus *Trigona* Jurine, 1807 (Rasmussen and Ardila-Camacho 2021).

#### ﻿﻿ Anchieta nothus

Taxon classificationAnimaliaNeuropteraRhachiberothidae

(Erichson, 1839)

Figs 14 , 15

Mantispa notha Erichson, 1839: 170. Holotype: male, Brazil (ZMB), specimen examined.
Trichoscelia notha (Erichson, 1839). Hagen (1861).
Symphrasis notha (Erichson, 1839). Navás (1909).
Anisoptera notha (Erichson, 1839). Enderlein (1910).
Anchieta notha (Erichson, 1839). Penny (1982b).

##### Material examined.

***Holotype*.** Brazil • ♂; “Virmum Leg., notha Er.”, “Holotype of *Mantispanotha* Erichson 1839, R.G. Beard” [red label], “holotypus Nr.” [red label], “123”, “prep. R.G. Beard, 1968, Holotypus Nr”; [Male genitalia and left forefemur cleared]; ZMB 123.

##### Other material.

Brazil • 1♀; **Bahía**; Hagen, Winthom leg.; MCZ. – **Minas Gerais** • 1 ♂; II.1941, Lopes & Gomes leg., det. L.A. Stange; FSCA.

##### Diagnosis.

This species has the general body color pattern dark brown. The forewing has pale amber, irregular areas at base, area around the 1r-m, proximal ¼ of wing, distal subcostal veinlets, apical area of subcostal field, and area surrounding 1ra-rp. The hind wing exhibits a broad, transverse, pale amber band on proximal 1/3. The tergites of abdominal segments III–VI have prominent, posteromedial, keeled processes. On the male genitalia, the sternite IX is posteromedially produced into a blunt, elongated lobe. The gonocoxite IX is short, narrow, sinuous, with posterior apex ventrally curved, and set with three short apical processes. On the female genitalia, the sternite VII appears as two lateral, broad, subtrapezoidal plates (gonocoxites VII), which are posteromedially fused through a thin bridge. The gonapophyses VIII medial part forms a cubic structure, with concave sides, and with an anteromedial incision.

##### Description.

***Measurements*.** Male (*n* = 2). Forewing length: 10.3–10.7 mm; Hind wing length: 5.9–6.4 mm. Female (*n* = 1): Forewing length: 12.0 mm; Hind wing length: 7.4 mm.

**Figure 14.**
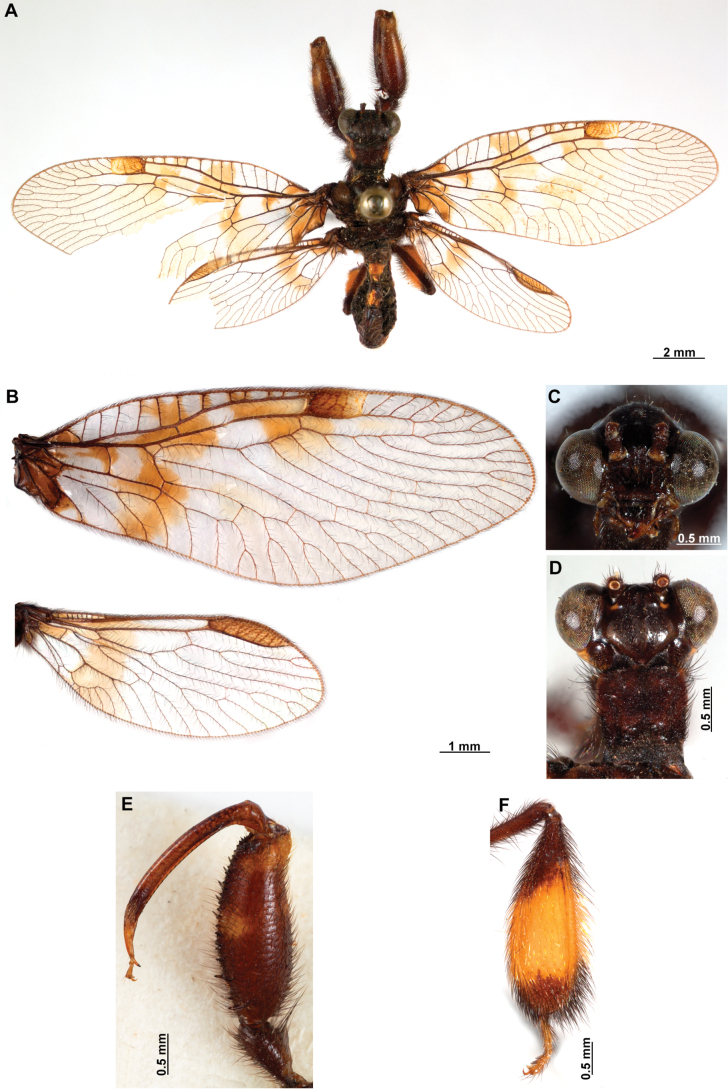
*Anchietanothus* (Erichson, 1830) **A** female habitus, dorsal **B** wings **C** head, frontal **D** pronotum, dorsal **E** forefemur, anterior surface **F** hind tibia, outer surface.

***Coloration*** (Fig. 14). ***Head*.** Mostly dark reddish brown, vertexal region with lateral rows of dark brown setae, supra-antennal region with small yellowish mark above toruli. Postgene yellowish with dark brown spots. Antenna dark reddish brown, except for four pale brown preapical flagellomeres. Mandible pale amber; maxillary palpus amber; labium black except pale brown ligula, labial palpus dark amber, palpimacula amber. ***Thorax*.** Pronotum dark reddish brown with yellowish markings on lateral edges; episternum and postfurcasternum dark reddish brown. Meso- and metanotum dark brown, metanotum with dark reddish brown bristles; pteropleura dark reddish brown. ***Foreleg*.** Coxa dark reddish brown; trochanter dark brown. Femur mostly dark brown with pale brown medial and apical areas. Tibia mostly brown, clavate setae pale brown; basitarsus amber with dark apex, second to fourth tarsomere pale brown tarsomere. ***Mid- and hind leg*.** Mid-leg with coxa, trochanter, femur, and tibia dark reddish brown; tarsomeres yellow with dark reddish brown setae. Hind leg with coxa, trochanter and femur dark brown; tibia with brown base and apex, medial region yellow, with concolorous setae; tarsomeres yellow with interspersed yellow and dark brown setae. ***Wings*.** Forewing membrane mostly hyaline, with pale amber irregular areas at wing base, area around the 1r-m, proximal 1/4 of wing, distal subcostal veinlets, apical area of subcostal field, and area surrounding 1ra-rp; pterostigma with proximal ¾ pale brown, distal ¼ pale yellow; venation mostly brown. Hind wing mostly hyaline with broad transverse pale amber band on proximal 1/3; costal field, basal ½ of subcostal space, and mcua1 pale amber; pterostigma brown, venation brown. ***Abdomen*.** Tergite of abdominal segments III and IX mostly yellow, the rest mostly dark reddish brown; sternites mainly brown, with yellow suffusions; pleural membrane dark brown.

***Morphology* (Fig. 14)**. ***Head*.** Diamond-shaped in front view, rugose; vertexal region domed above compound eyes; coronal suture distinct; paraocular area concave; supra-antennal region with lateral, elongated, raised areas. Antenna moniliform, with 51 flagellomeres, discoidal in shape, those at the apex narrower; all flagellomeres with medial ring of short setae. Compound eye hemispheric, as wide as ¾ of interocular distance at toruli level. ***Thorax*.** Pronotum nearly as long as wide, with groove contiguous lateral and distal margins; in lateral view, posterior margin slightly raised; entire surface with abundant, thick setae arising flush the pronotal surface. Postfurcasternum quadrangular. Mesonotum wider than long, with abundant long and thick setae; metanotum ~ 3× as wide as long, mostly glabrous covered. Pteropleura covered with abundant fine and long setae. ***Foreleg*.** Coxa as long as femur, cylindrical, slightly expanded at preapical region, with abundant fine and long setae; trochanter subtrapezoidal, densely setose, dorsally with a tuft of long, thick setae, inner surface with blunt process. Femur robust, with abundant, long, fine setae; closing surface with posterolateral row of integumentary specializations fully-developed, composed of tubercle-shaped processes, proximally with a more developed, spine-shaped, sub-basal process, adjacent row of thickened setae with globular base reduced to distal ¾ or ½; anteroventral row of specializations reduced to proximal region and apex, composed of tubercle-shaped processes; primary, spine-shaped process present, curved; adjacent row of thickened setae with globular base present on distal 4/5. Tibia almost as long as femur, curved, glabrous, closing surface with a row of prostrate setae; anterior surface with a patch of clavate setae at apex; basitarsus with long lanceolate process; proximal ½ with patch of clavate setae on anterior surface, and single row of prostrate setae ventrally. ***Mid- and hind leg*.** Mid-leg with coxa, trochanter and femur densely covered with fine and long setae; tibia moderately, medially expanded, with abundant long, thick setae; basitarsus as long as the following three together, covered with shorter setae compared to other segments. Hind leg with coxa, trochanter and femur covered with abundant, thin, long setae; tibia conspicuously expanded, laterally flattened, oar-shaped, densely setose, with setae shorter than those of mid tibia; tarsomeres with short, thin setae, basitarsus as long as the following three tarsomeres together. ***Wings*.** Forewing oval, venation setose, trichosors present along wing margin except at base; costal space slightly widened medially, humeral vein sometimes forked, 11–13 subcostal veinlets present; pterostigma rectangular; subcostal space with single, medial crossvein; Sc abruptly bent posteriad at proximal margin of pterostigma to merge the RA; *rarp2* straight with three or four veins arising from it, three from *rarp1*; M basally fused with R; RP base located near separation of M from R, M fork near such separation; 1r-m situated between RP base and M fork, forming a small trapezoidal cell; 4–6 gradate crossveins present; Cu deeply forked, CuP basally angled, approaching A1, forked slightly beyond 1m-cu; A1 simple. Hind wing notably smaller and narrower than fore wing, oval; costal space narrow and reduced, with 7–9 veinlets; C and Sc fused at 1/3 of wing length; subcostal space without crossveins; Sc vein abruptly curved posteriad at proximal margin of pterostigma to merge the RA; pterostigma elongated, oval; radial space with single crossvein, two veins arising from *rarp1*, one or two from *rarp2*. M vein forked slightly beyond R fork. Cu vein deeply forked, CuA straight to slightly concave, distally simple or forked, first branch simple or forked; CuP distally, strongly, anteriorly arched, pectinate; A1 arched, A2 short, simple. ***Abdomen*.** Medially widened, tergites of abdominal segments III–VII with prominent posteromedial keeled processes; processes on tergites IV–VI notably enlarged in both sexes, in male the largest situated on tergite IV, and on tergite V in female.

**Figure 15.**
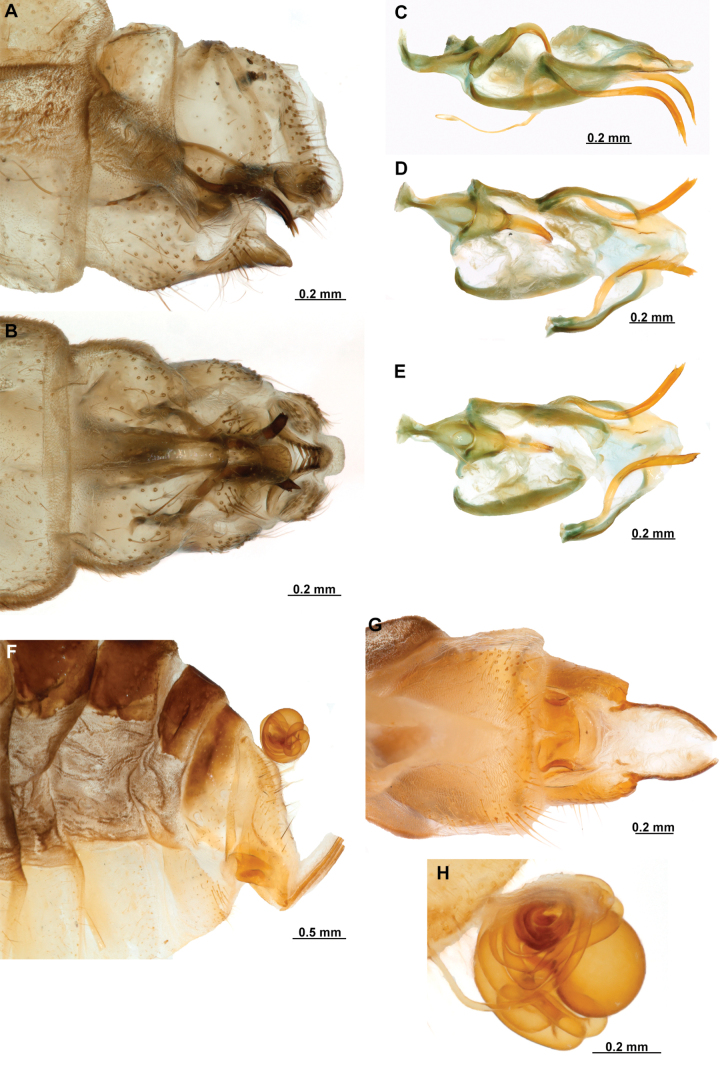
*Anchietanothus* (Erichson, 1830) **A** male terminalia **B** same, ventral **C** male genitalia, lateral **D** same, dorsal **E** same, ventral **F** female terminalia, lateral **G** same, ventral **H** spermatheca.

***Male genitalia*** (Fig. 15A–E). Tergite IX notably narrower medially than laterally, lateral margin rounded, posteriorly with long and thin setae. Sternites VIII and IX fused, fusion line barely perceptible; sternite VIII with abundant long, thin setae; sternite IX quadrangular, posteromedially produced into a blunt, elongated lobe; lateral margins concave, median region with curved, transverse rows of long, thin setae. Gonocoxites IX short and narrow, as long gonostyli X, sinuous, posterior apex ventrally curved, with three short apical processes. Ectoproct elongated and narrow, with subparallel anterior and posterior margins, posteroventrally with short, pedicellate setae; ventrally on inner surface with a more sclerotized region set with ~ 15 short and thick setae on posterior ½ and some fine and short setae on anterior ½. Gonocoxites X constrained at anterior 1/3, base spatulate and bent dorsally; apex with dorsal and lateral processes; base of gonostyli X triangular, ventrally concave; the rest of the structure whip-shaped, short, with apical portion recurved. Gonapophyses X slightly thickened, gently curved, anterior apex spatulate, dorsally recurved; both joined by membranes forming a V-shaped structure. Gonocoxites XI narrow, arched, medial lobe expanded, composed of two oval, widened, concave plates, joined medially by narrow bridge. Lateral arms of gonocoxites XI with short and slender.

***Female genitalia*** (Fig. 15F–H). Sternite VII as two lateral, broad, subtrapezoidal plates (gonocoxites VII), posteromedially fused through thin bridge, posterior margin broadly concave. Tergite VIII slightly narrower medially than laterally, enclosing the spiracle of the segment, lateral margin quadrangular, with a notch. Gonocoxites VIII as two lateral, trapezoidal, concave plates; gonapophyses VIII medial part forming a cubic structure, with concave sides, and with an anteromedial incision; lateral part a trapezoidal, elongated plate, hidden under the tergite IX + ectoproct. Tergite IX + ectoproct triangular. Gonocoxites IX narrow, elongated, as long as the last four abdominal segments. Gonapophyses IX reduced to a pair of tiny sclerites hidden by tergite IX + ectoproct. Bursa copulatrix funnel-shaped, membranous, short. Spermatheca tightly coiled; proximal section long and thin, forming three coils distally; medial section, short, thicker than proximal section, forming a pair of convolutions; distal section markedly wider than medial section, progressively expanded towards apex, sac-like; fertilization canal duct long, spiral-shaped, with five convolutions, widened at preapical part; fertilization canal elongated, narrow, J-shaped, covered with microfilaments.

##### Distribution.

Brazil (Bahía, Minas Gerais).

##### Remarks.

Prior to this work, this species was known from Brazil, although information about its distribution within the country is not available (Machado 2022). The type specimen of *Mantispanotha* deposited in Museum für Naturkunde der Humboldt-Universität (ZMB) has only “Brasilien” as collecting locality. Penny and da Costa (1983) redescribed this species based on a female lacking collecting data. Herein the first specific records of this species in Brazil (i.e., Bahía and Minas Gerais) are presented.

Regarding the phylogenetic relationships of this species, it was recovered as sister to *A.nebulosus* in the clade comprising the bee-mimicking species. As mentioned above for *A.nebulosus*, *A.nothus* has essentially the same coloration pattern which consists of a dark brown body and wings with extensive amber areas. The hind tibia of both species is markedly expanded and flattened, but in *A.nothus* a broad, yellow, medial area is present. The morphology and coloration of this species represents a mimicry pattern whose model is probably the meliponine genus *Trigona* Jurine, 1807 (Rasmussen and Ardila-Camacho 2021).

#### ﻿﻿ Anchieta partheniellus

Taxon classificationAnimaliaNeuropteraRhachiberothidae

(Westwood, 1867)

Figs 16 , 17

Mantispa (Trichoscelia) partheniella Westwood, 1867: 501. Lectotype male, Paralectotype female, Amazonia (OUMNH), specimens examined.
Trichoscelia partheniella (Westwood, 1867). Enderlein (1910).
Anchieta partheniella (Westwood, 1867). Penny (1982a), Ohl (2004).

##### Material examined.

***Lectotype*.** [Brazil] • 1 ♂; **Amazonas**; 1861; Bates leg.; “*Platymantispapartheniella* (Westwood, 1867)”, “Lectotype male, *Trichosceliapartheniella* Westwood, 1867 by R.G. Beard, 1968”, “*partheniella* Westwood, male”, “Type Westwood, Trans. Entomol. Soc. 1865, p. 501, Coll. Hope Oxon”, “Type Neur: No. 61/2, *MantispaTrichosceliapartheniella* Westwood, HOPE Dept. Oxford”; right foreleg and abdomen cleared and stored in a microvial; OUMNH.

##### Other material.

Brazil – **Amazonas** • 1 ♂; Ipixuna, Río Gregorio, com. Lago Grande; 07°10'11.7"S, 70°49'10.3"W; 18–23 May. 2011; J.A. Rafael, J.T. Cámara, R.F. Silva, A. Somavilla, R. Ale-Rocha leg.; Varredura; INPA. – **Pará** • 1 ♀; Tucurú, Río Tocantins; 03 Jun. 1984; Saúde leg.; Malaise trap; *Anchietabella* (Westwood, 1867) det. R.J.P. Machado; INPA. – **Rondônia** • 1 ♀; 62 Km SW Ariquemes, Fazenda Rancho Grande; 11 Oct. 1993; C.W. and L.B. O’Brien leg.; CAS. • 1 ♀; same data as for preceding; UV merc. Vapor light; *Anchietabella*, det. Penny, 2001; CAS. • 1 ♀; same data as for preceding; 9 Nov.1994; CAS.

Colombia • 1 ♀; **Meta**, Cabaña Carrillo, PNN Sierra de la Macarena; 3°21'N, 73°56'W; 460 m, 29 Dec. 2003; W. Villalva leg.; Malaise trap, m-4240; IAvH.

Ecuador • 1 ♀; **Napo**, near Tena Jatun Sacha; 450 m; G. Beccaloni leg.; BMNH(E) 1241394; NHMUK.

Suriname • 1 ♀; Republiek; 13 Nov. 1961; P.H.v. Doesburg leg.; *A.romani*, det. Geisjkes 1970; FSCA.

Venezuela • 1 ♂; **Amazonas**, T.F., Cerro de la Neblina, Basecamp, near Río Baria; 0°50'N, 66°10'W; 140 m; 18 Feb.1985; P.J. and P.M. Spangler, R. Faitoute, W. Steiner leg.; at black light in rainforest clearing; *Anchietabella*, det. O.S. Flint, 1986; USNMENT01541897; USNM. • 1 ♀; same data as for preceding; USNMENT01541896; USNM.

##### Diagnosis.

This species has a general body coloration pattern of bright orange with brown areas, which is also present in *A.apiculasaeva*, *A.bellus*, *A.remipes*, and *A.romani*. The forewing costal space is slightly widened medially. The hind tibia is conspicuously widened and laterally flattened. On the male genitalia, the gonocoxites IX is short, with posterior apex strongly recurved lateroventrally, and set with two apical and four preapical, short processes on outer surface. The gonocoxites X form a hourglass-shaped sclerite, whose anterior ½ is expanded, and dorsally bent. On the female genitalia, the gonapophyses VIII medial part appears as a broad canal, with, ventral, medial depression, and distal margin rounded, and ventrally curved.

##### Description.

***Measurements*.** Male (*n* = 3). Forewing length: 9.3–10.3 mm; Hind wing length: 6.0–6.56 mm. Female (*n* = 3): Forewing length: 9.3–9.7 mm; Hind wing length: 5.6–5.9 mm.

**Figure 16.**
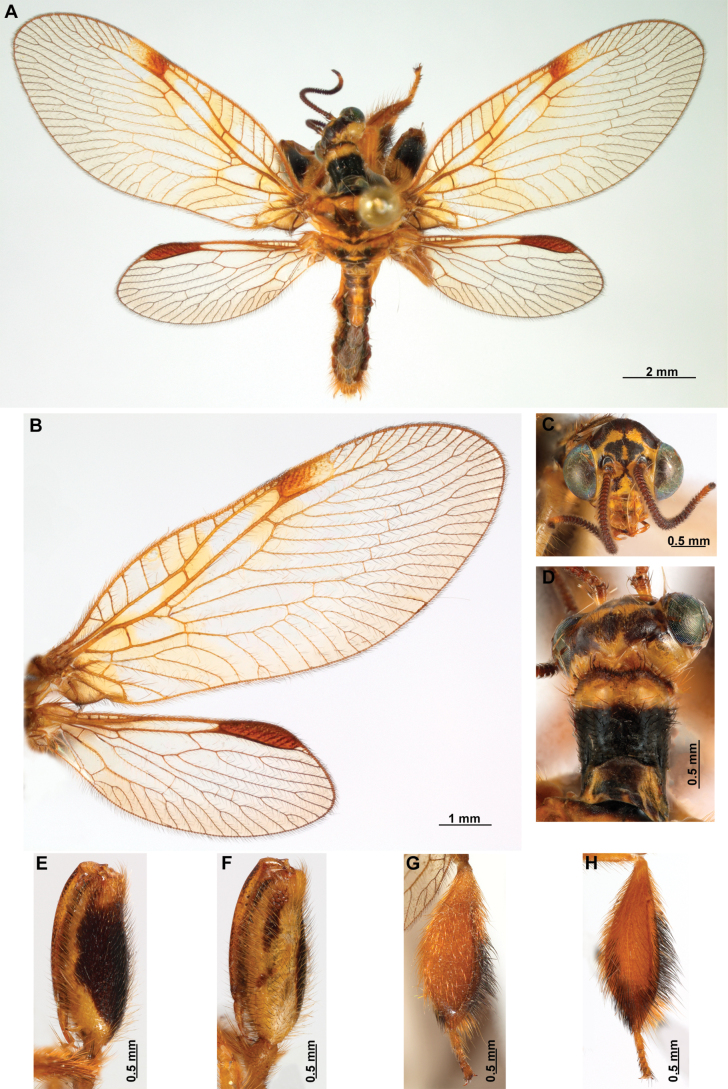
*Anchietapartheniellus* (Westwood, 1867) **A** male habitus, dorsal **B** wings **C** head, frontal **D** pronotum, dorsal **E** forefemur, anterior surface **F** same, posterior surface **G** hind tibia, outer surface **H** same, inner surface.

***Coloration* (Fig. 16)**. ***Head*.** Mostly orange; vertexal region posteriorly with a broad transverse brown band, laterally with rows of dark brown setae; supra-antennal area with dark brown, lateral trapezoidal markings, such markings connected to the frontal markings through interantennal area. Antennal scape ventrally orange, dorsally brown, pedicel brown, flagellum brown, except for four or five yellow preapical flagellomeres. Frons with lateral brown triangular markings forming an inverted, V-shaped pattern. Clypeus with brown posterolateral corners; Labrum with brown medial suffusions; mandible orange with dark amber apex; maxillary palpus brown; labium orange, palpus brown, palpimacula pale brown. ***Thorax*.** Pronotum dark brown, with orange, transverse band on anterior 1/3, anterior margin brown; episternum bicolor, yellow on anterior ¼, dark brown on the rest; postfurcasternum pale to dark brown. Mesonotum orange with brown anterior margin, medial area of scutum with brown suffusions, posterior margin of scutellum dark brown, setae pale brown; metanotum with brown lateral markings, posterior margin of scutellum brown. Pteropleura mainly orange with some brown areas, setae orange. ***Foreleg*.** Coxa mostly orange with extensive dark brown area on posterior surface, apex slightly infuscated; anterior surface with interspersed orange and dark brown setae; trochanter orange except dorsally brown. Femur orange, with enlarged dorsal spot extending towards anterior surface; posterior surface with brown preapical longitudinal mark, setation mostly orange, except on medial region dark brown; tibia bicolor with orange basal ½, brown distal ½; basitarsus pale brown with amber apex, remaining tarsomeres orange. ***Mid- and hind leg*.** Mid-leg predominantly orange, with brown suffusions. Hind leg orange; femur with brown medial suffusion; tibia with posterodistal margin and distal 1/3 of anterior surface black. ***Wings*.** Forewing membrane hyaline, with yellow areas at wing base and area adjacent to proximal region of pterostigma; a yellow transverse band R fork level is present; pterostigma bicolor, orange on proximal ½, yellow on distal ½; venation predominantly orange with dark brown suffusions at base of RA and jugal lobe. Hind wing hyaline, base of subcostal space orange; pterostigma orange with black suffusions; venation orange. ***Abdomen*.** Tergite I yellow with brown area at center, tergite II with lateral brown markings, tergites III and IV yellow with two brown, lateral bands, tergites V–VIII brown; sternites yellow.

***Morphology*** (Fig. 16). ***Head*.** Diamond-shaped in frontal view, smooth; vertexal region domed above compound eyes, laterally with rows of short, reclined setae; paraocular area concave; coronal suture discrete. Antenna moniliform, flagellum with 51 flagellomeres, discoidal in shape, except for those of apex narrower; all flagellomeres with medial ring of short setae. Compound eye hemispherical, as wide as 1/2 of interocular distance at toruli level. ***Thorax*.** Pronotum nearly as long as wide, with groove contiguous lateral and distal margins; posterior region slightly raised; entire surface with scattered, thick setae arising flush the pronotal surface. Episternum with long, thin setae; postfurcasternum quadrangular. Mesonotum slightly wider than long, with few long, fine setae; metanotum ~ 3× as wide as long, glabrous. Pteropleura covered with abundant fine, long setae. ***Foreleg*.** Coxa as long as femur, cylindrical, slightly expanded at preapical region, with abundant fine, long setae; trochanter semi-triangular, densely setose, dorsally with tuft of long, thick setae, anterior surface with blunt process. Femur robust, covered with abundant long, fine setae; closing surface with posteroventral row of integumentary specializations fully developed, composed of tubercle-shaped processes, proximally with sub-basal, spine-shaped process, adjacent row of thickened setae with globular base present on distal ½; anteroventral row of specializations reduced to proximal region and apex, composed of tubercle-shaped processes, with conical setae; primary, spine-shaped, basal process present, curved; adjacent row of thickened setae with globular base present on distal 4/5. Tibia almost as long as femur, curved, glabrous, with a row of prostrate setae on closing surface, anterior surface with apical patch of clavate setae. Basitarsus with long lanceolate process; anterior surface with patch of clavate setae on proximal ½, ventrally with single row of prostrate setae. ***Mid- and hind leg*.** Mid-leg covered with abundant, fine, long setae; tibia slightly widened. Hind leg densely covered with long, fine setae, except on tarsus shorter; tibia conspicuously widened and laterally flattened, oar-shaped. ***Wings*.** Forewing oval, venation densely setose, trichosoros present along wing margin except at base; costal space slightly widened medially, humeral vein forked, 10–12 subcostal veinlets present; pterostigma rectangular; subcostal space with single, medially located crossvein; Sc vein abruptly bent posteriad at proximal margin of pterostigma to merge the RA; *rarp2* straight with four veins arising from it, four from *rarp1*; M fused basally to R; RP base located near separation of M and R, M fork near such separation; 1r-m located between RP base and M fork, forming a small trapezoidal cell; six gradate crossveins present; Cu deeply forked, CuA distally branched, CuP proximally angled, forked; A1 simple. Hind wing oval, notably smaller and narrower than forewing; costal space narrow and reduced, with seven or eight subcostal veinlets; C and Sc fused at 1/3 of wing length, subcostal space without crossveins, Sc vein abruptly curved posteriad at proximal margin of pterostigma to merge the RA; pterostigma elongated, slightly widened distally; radial space widened, with single, sinuous crossveins; two veins arising from *rarp1*, two or three from *rarp2*. M forked at R fork level. Cubitoanal space with distal crossveins. Cu vein deeply forked, CuA gently curved to nearly straight, distally forked, first branch simple; CuP distally anteriorly bent near wing margin, with three branches; A1 arched, A2 short, simple. ***Abdomen*.** Medially widened, without keeled processes, tergites V–IX with abundant long, thin setae; sternites densely setose.

**Figure 17.**
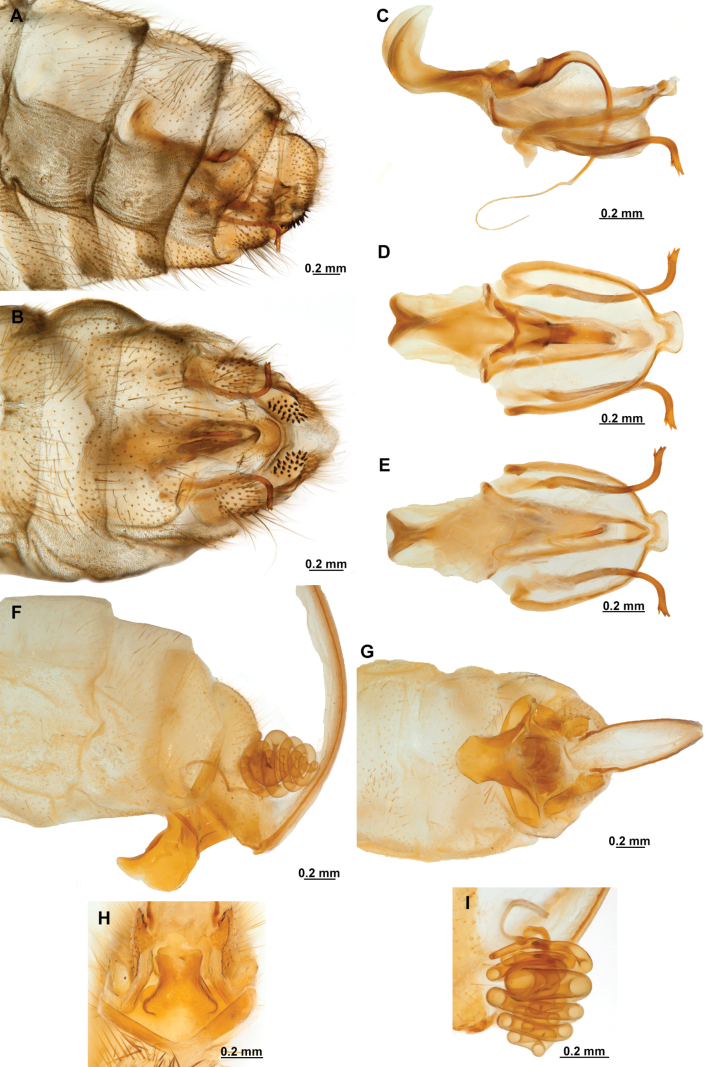
*Anchietapartheniellus* (Westwood, 1867) **A** male terminalia, lateral **B** same, ventral **C** male genitalia, lateral **D** same, dorsal **E** same, ventral **F** female terminalia, lateral **G** same, ventral **H** gonapophyses VIII of female, ventral **I** spermatheca.

***Male genitalia*** (Fig. 17A–E). Tergite IX medially narrower than laterally, posterior corner with abundant long, thick setae. Sternites VIII and IX fused, fusion line weakly marked; sternite IX anteriorly with long, thick setae, medially with short setae, distal ½ glabrous with concave lateral margins; anterior ½ somewhat constricted. Gonocoxites IX short, base spatulate; apex strongly recurved lateroventrally, with two apical and four preapical processes on outer surface. Ectoproct ovoid, posteroventrally with patch of 22 or 23 thickened, dark setae; outer surface with smooth, glabrous, bulging area; inner surface with sclerotized ventral area, with a patch of microtrichia. Gonocoxites X medially constricted, hourglass-shaped; anterior ½ thickened, expanded, dorsally bent, ventrally canaliculated; posterior ½ triangular, with paired dorsal and lateral processes; gonostyli X base triangular, thickened, with curved lateral processes, dorsally concave, the rest of the structure whip-shaped, short, apical portion recurved. Gonapophyses X rod-shaped, straight, narrow, anterior apex spatulate, dorsally curved, posterior apex dorsally curved, surrounding membrane with minute granules; gonapophyses joined by membrane, forming a V-shaped structure. Gonocoxites XI thin, U-shaped, medial lobe flattened, weakly sclerotized at center, posterior margin almost straight, lateral margins emarginate. Lateral arms of gonocoxites XI straight, with anterior apex curved.

***Female genitalia*** (Fig. 17F–I). Sternum VII (gonocoxites VII) composed of two medially fused trapezoidal lateral plates, posterior margin obtuse. Tergite VIII slightly narrower medially than laterally, enclosing spiracle of the segment, lateral margin rounded. Gonocoxites VIII as two concave, medially fused, trapezoidal plates; gonapophyses VIII medial part ventrally projected forming a canal, with medial depression on ventral surface, distal margin rounded, ventrally curved; lateral part of gonapophyses VIII a trapezoidal plate, forming a chamber-shaped plate, dorsally with two convex areas, located behind the genital pore. Tergite IX + ectoproct triangular. Gonocoxite IX long, straight, narrow, as long as the last five abdominal segments together. Bursa copulatrix unsclerotized, long, conical; spermatheca spiral-shaped; proximal section long, thin, progressively thickened, spiral-shaped, forming three coils; medial section slightly narrower than thickest part of proximal section, entangled, forming several convolutions; distal section thicker than proximal and medial sections, progressively wider towards the apex. Fertilization canal duct long, spiral-shaped, forming two coils; fertilization canal elongated, narrow, J-shaped, covered with microfilaments.

##### Distribution.

Brazil (Amazonas, Pará, Rondônia), Colombia (Meta), Ecuador (Napo), Suriname, Venezuela (Amazonas).

##### Remarks.

Lectotypes (male and female) of this species deposited in the Oxford University Museum have only “Amazons” as collecting data. Penny (1982a) and Penny and da Costa (1983) redescribed this species, but no additional records were presented. Although the types of this species were likely collected in Brazil, so far there are no specific state records of this species in the country. Herein, the first records for the states of Amazonas, Pará, and Rondônia (all in the Amazon region) are presented, and the first records from Colombia, Ecuador, Suriname, and Venezuela are provided.

In the phylogeny of the subfamily, *A.partheniellus* was recovered as sister to *A.nebulosus* + *A.nothus*. However, this species exhibits the same mimicry pattern as *A.apiculasaeva*, *A.bellus*, *A.remipes*, and *A.romani* whose model is probably the meliponine genus *Ptilotrigona* Moure, 1951 (Rasmussen and Ardila-Camacho 2021). Considering the relationships between these species, this coloration pattern apparently arose several times within this group of species of *Anchieta*.

The distinction between the species exhibiting this mimicry pattern may be difficult as all of them have a rather similar morphology, although *A.partheniellus* can be separated from other species with the same coloration by the arrangement and number of processes on the male gonocoxites IX, the enlarged and curved gonocoxites X and the complex spermatheca.

#### ﻿﻿ Anchieta remipes

Taxon classificationAnimaliaNeuropteraRhachiberothidae

(Gerstaecker, 1888)

Figs 18 , 19

Anisoptera remipes Gerstaecker, 1888: 120. Holotype: male, Colombia, Bogotá (ZIMG), high resolution images examined.
Trichoscelia remipes (Gerstaecker, 1888). Penny (1977), Hoffman (2002), Ohl (2004).
Anchieta remipes (Gerstaecker, 1888). Ardila-Camacho and García (2015).

##### Material examined.

***Holotype*.** Colombia • ♂; “*remipes* Gerst.* Columb. Thieme”, “*An.remipes* Gerst.* Columb. Thieme”, “Zool. Mus. Greifswald II 27487”, “*Anchietaremipes* det. R. Hall (HNRS) 1988”; Male genitalia cleared and stored in a microvial; ZIMG.

##### Diagnosis.

This species is separated from other of the genus with similar body coloration pattern (*A.apiculasaeva*, *A.bellus*, *A.partheniellus*, and *A.romani*), because the forecoxa has the proximal 2/3 brown and distal 1/3 orange. The forewing costal space is slightly widened medially, and the hind tibia is oar-shaped, notably expanded, and laterally flattened. On the male genitalia, the sternite IX has a broad posteromedial, quadrangular lobe. Like in *A.tinctus*, the gonocoxite IX of this species are fusiform and reduced, and the gonostyli X dorsally recurved and anteroventrally projecting.

##### Description.

***Measurements*.** Male (*n* = 1). Forewing length: 8.9 mm; Hind wing length: 5.0 mm.

**Figure 18.**
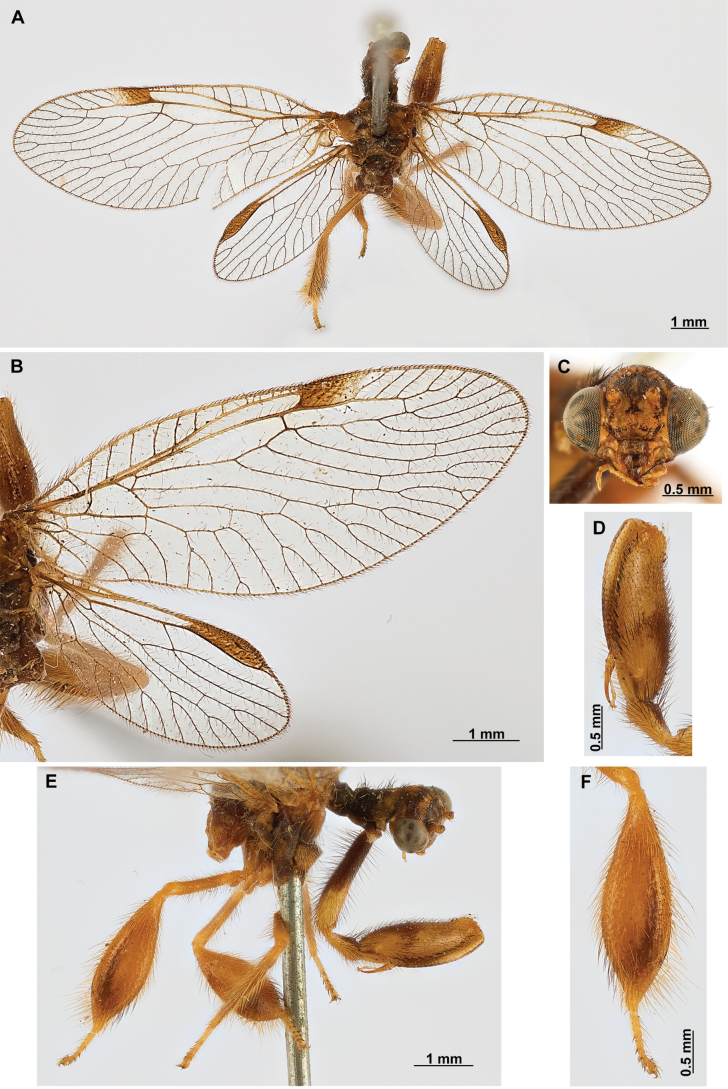
*Anchietaremipes* (Gerstaecker, 1888) **A** male habitus, dorsal **B** wings **C** head, frontal **D** forefemur, anterior surface **E** head and thorax, lateral **F** hind tibia, outer surface.

***Coloration*** (Fig. 18). ***Head*.** Mostly orange, vertexal region with posterior, transverse brown band; supraantennal region with lateral triangular marks; with lateral rows of brown setae. Antennal scape orange. Frons with brown suffusions. Clypeus orange laterally, brown at center; labrum medially brown, orange on edges; mandible orange with amber apex; maxillary palpus orange; labium orange, palpimacula orange. ***Thorax*.** Pronotum dark brown with orange, transverse band on anterior 1/3, anterior margin brown; episternum pale brown with darker margins, postfurcasternum dark brown with poster paler area. Mesonotum orange with brown areas; metanotum mostly brown. Pteropleura with episterna orange with brown areas, epimera brown. ***Foreleg*.** Coxa brown, with distal 1/3 orange, setation brown; trochanter orange except dorsally brown. Femur orange with pale brown, irregular areas on posterior and dorsal surfaces; anterior surface with medial area pale brown; setation mostly orange; basal ½ of tibia orange, distal ½ brown; clavate setae orange; tarsus orange. ***Mid- and hind leg*.** Mid-leg mostly orange. Hind leg mostly orange; tibia with posterior margin and preapical region with brown suffusions. ***Wings*.** Forewing membrane hyaline; pterostigma bicolor, pale brown on proximal ⅔, pale yellow on the rest; venation with orange and brown areas. Hind wing hyaline, pterostigma pale brown, venation mostly brown, most of C and Sc orange. ***Abdomen*.** cleared.

***Morphology*** (Fig. 18). ***Head*.** Diamond-shaped in frontal view; vertexal region domed above compound eyes, smooth, laterally with rows of short, reclined setae; paraocular area concave; coronal suture discrete. Antennal scape 2× as long as wide. Compound eye hemispherical, as wide as ½ the interocular distance at toruli level. ***Thorax*.** Pronotum as long as wide, with groove contiguous to lateral and distal margins; posterior region slightly raised, entire surface with abundant, thick setae arising flush the pronotal surface; episternum with long, thin setae; postfurcasternum trapezoidal. Mesonotum 1.5× as wide as long, with few long, thickened setae; metanotum ~ 3× as wide as long, glabrous. Pteropleura covered with abundant long, thin setae. ***Foreleg*.** Coxa as long as femur, cylindrical, slightly expanded at preapical region, densely covered with abundant fine, long setae; trochanter conical, densely setose, dorsally with tuft of long, thick setae, anterior surface with blunt process. Femur robust, densely set with fine setae; closing surface with posteroventral row of integumentary specializations fully-developed, composed of tubercle-shaped processes, proximally with spine-shaped, sub-basal process, adjacent row of thickened setae with globular base present on distal ½; anteroventral row of specializations reduced to proximal region and apex, composed of tubercle-shaped processes; primary process present, curved; adjacent row of thickened setae with globular base present on distal ⅘. Tibia almost as long as femur, curved, glabrous, with a row of setae prostrate on closing surface, and a patch of clavate setae apically on anterior surface. Basitarsus with lanceolate process reaching middle of fourth tarsomere, proximal ½ with patch of clavate setae on anterior surface, ventrally with single row of prostrate setae. ***Mid- and hind leg*.** Mid-leg covered with abundant fine, long setae, tibia slightly widened. Hind leg densely covered with long, fine setae, except on the tarsi, shorter, tibia notably widened, laterally flattened, oar-shaped. ***Wings*.** Forewing oval, venation densely setose, trichosors present along wing margin except at base; costal space slightly widened medially, humeral vein forked, 11–13 subcostal veinlets present; pterostigma rectangular; subcostal space with single, medially located crossvein; Sc vein abruptly bent posteriad at proximal margin of pterostigma to merge the RA; *rarp2* straight, with three veins arising from it, two from *rarp1*; M basally fused to R; RP base located near separation of M and R, M fork near such separation; 1r-m located between RP base and M fork forming a small trapezoidal cell; three gradate crossveins present; CuA distally branched; CuP angled proximally, touching A1, forked nearly at M fork level; A1 simple, A2 forked. Hind wing oval, notably smaller and narrower than forewing; costal space narrow and reduced, with five veinlets; C and Sc fused at proximal 1/3 of wing length, subcostal space without crossveins; Sc vein abruptly curved posteriad at proximal margin of pterostigma to merge the RA; pterostigma elongated, slightly widened distally; radial space widened with single reclined crossvein; one or two veins arising from *rarp1*, 0–2 from *rarp2*. M vein forked slightly beyond R fork. Cu vein deeply forked, CuA slightly bent at mid-length, distally forked; CuP distally bent anteriorly near posterior wing margin, with three branches; A1 arched, A2 short, simple.

***Abdomen*.** Cylindrical, without keeled processes.

**Figure 19.**
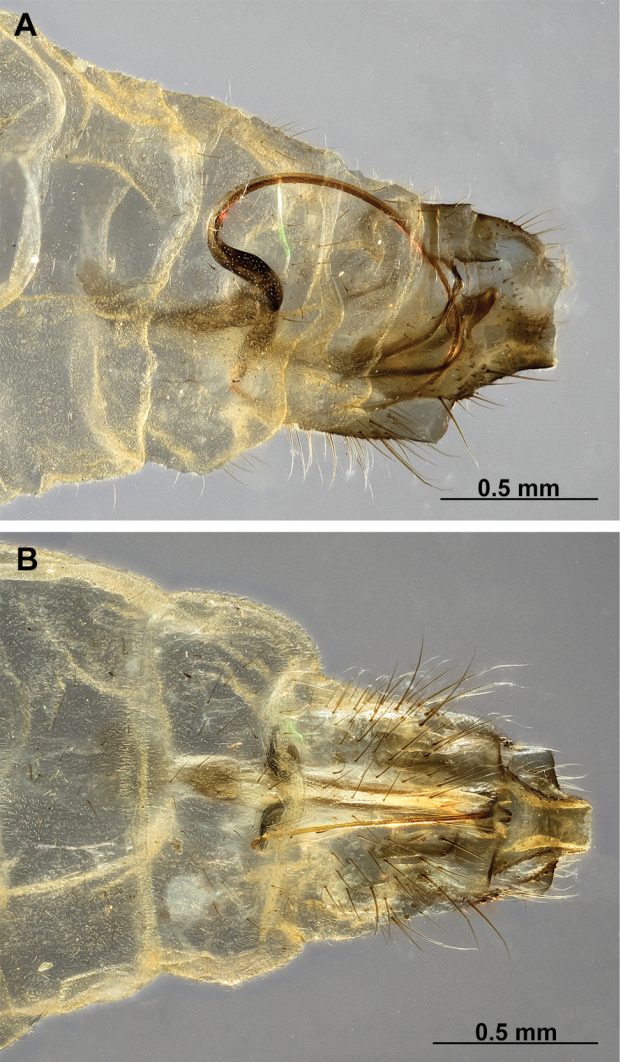
*Anchietaremipes* (Gerstaecker, 1888) **A** male terminalia, lateral **B** same, ventral.

***Male genitalia*** (Fig. 19). Tergite IX medially narrower than laterally, laterally with abundant long, thin setae. Sternites VIII and IX fused, fusion line weakly marked; sternite VIII rectangular, with long, thickened setae; sternite IX trapezoidal, with broad posteromedial, quadrangular lobe. Gonocoxites IX fusiform, reduced. Ectoproct triangular, ventrolaterally with smooth, glabrous, bulging area. Gonocoxites X forming an elongate sclerite; anterior dorsally curved; posterior apex expanded; base of gonostyli X triangular, thickened, with lateral processes; the rest of the structure whip-like, short, dorsally recurved, and anteroventrally projected. Gonapophyses X elongate, narrow; gonapophyses joined by membrane, forming a V-shaped structure, posterior apex of both fused, forming a blunt process. Gonocoxites XI thin, U-shaped, medial lobe flattened, posterior margin rounded; lateral arms of gonocoxites XI straight.

##### Distribution.

Colombia (Cundinamarca?).

##### Remarks.

This species is so far known only from the original description by Gerstaecker (1888). In this description the only collecting data available is “Bogotá”, the main city of Colombia, located in the Cundinamarca department. However, the labels of the holotype only have “Columb.” As collecting information, so presently the distribution of the species in Colombia is unknown.

Regarding the phylogenetic relationships of this species, despite presenting the same mimetic coloration pattern of species like *A.bellus*, *A.apiculasaeva*, *A.partheniella*, and *A.romani*, this species was recovered as sister of *A.tinctus* from Costa Rica. Both species share many characters of the wing shape and venation as well as of the male genitalia. Nonetheless, *A.remipes* can be differentiated from *A.tinctus* by the body coloration and morphology of the sternite IX, while the overall genitalic morphology markedly differs from other species mimicking stingless bees of the genus *Ptilotrigona*.

#### ﻿﻿ Anchieta romani

Taxon classificationAnimaliaNeuropteraRhachiberothidae

(Esben-Petersen, 1917)

Figs 20 , 21

Anisoptera romani Esben-Petersen, 1917: 14. Holotype: male, Brazil, Rio Autaz (NHRS), specimen examined.
Trichoscelia romani (Esben-Petersen, 1917). Penny (1977).
(non) Anchietabella (Westwood, 1867). Penny (1982a).

##### Material examined.

***Holotype*.** Brazil • ♂; **Amazonas**, Río Autaz; Sept.; Roman leg.; “*Anisopteraromani*, male, det. Esben-Petersen (NHRS)”, “Type red label, Rikmuseum Stockholm”, “198, 80”, “190, 57”, “NHRS-KAJO 000000370”; terminalia cleared and stored in a microvial; NHRS.

##### Other material.

Peru • 1 ♀ 2 ♂; **San Martín**, Tarapoto Urku, nr. Boca Toma; 400 m. a.s.l.; 10 Jul. 2012; C. Rasmussen leg.; nest collected; ZMB. • 1 pupa; same data as for preceding; 6 Sep. 2012; ZMB.

##### Diagnosis.

This species is remarkably similar to *A.apiculasaeva* and *A.partheniella* because of the general color pattern of orange with brown marks. Additional similarities include the slightly widened forewing costal space near the medial region, and the notably expanded and laterally flattened hind tibia. On the male genitalia, the sternite IX is U-shaped with posterior ½ glabrous, medially convex, and laterally concave, forming a canal. The gonocoxite IX is short, sinuous, with posterior apex laterally recurved, and set with 1–3 longitudinally arranged, preapical processes on the outer surface. An important distinction from the aforementioned species is the shape of the gonocoxites X, which form a triangular sclerite, with anterior apex laterally flattened and not expanded. On the female genitalia, the gonapophyses VIII medial part forms an enlarged canal, with ventral depression and apex ventrally curved, and rounded.

##### Description.

***Measurements*.** Male (*n* = 2). Forewing length: 9.0–9.2 mm; Hind wing length: 5.2–5.4 mm.

***Coloration*** (Fig. 18). ***Head*.** Mostly orange; vertexal region brown with triangular, yellow areas adjacent to frontal sutures; laterally with rows of dark brown setae; supra-antennal region brown with oval yellow, medial area. Antennal scape orange ventrally, dorsally pale brown on distal ½, pedicel pale brown, flagellum pale brown, except for three or four yellow, preapical flagellomeres. Frons with lateral brown triangular markings, forming an inverted V-shaped pattern. Labrum yellow, clypeus with brown suffusions at center; mandible orange with pale amber apex; maxillary palpus orange with pale brown suffusions; labium orange, palpus with pale brown suffusions, palpimacula orange. ***Thorax*.** Pronotum dark brown, except anterior 1/3 with orange transverse band; episternum brown with yellow anterior margin; postfurcasternum brown. Mesonotum orange with dark brown anterior margin, medial area with broad, brown band, scutellum with brown spot on anterior ½ and brown posterior margin; metanotum yellow with brown lateral markings, and brown medial band and anterior and posterior margins; pteropleura orange with broad brown areas, pale brown or orange setae present. ***Foreleg*.** Coxa mostly orange, with extensive pale brown spot on posterior surface, base and apex brown; trochanter orange on ventral surface, pale brown on dorsal surface. Femur orange with broad dorsal spot extending to most of anterior surface; posterior surface with preapical, longitudinal mark and brown transverse medial mark; tibia bicolor, basal ½ orange, distal ½ pale brown; basitarsus orange on basal 1/2, pale brown on distal 1/2, remaining tarsomeres orange. ***Mid- and hind leg*.** Mid-leg predominantly orange, coxa with basal 1/3 and apex pale brown, trochanter orange with pale brown suffusions, femur medially pale brown; tibia with apical 1/3 pale brown. Hind leg mainly orange, coxa with pale brown suffusions; tibia orange, except at middle of outer surface and distal ¾ of inner surface brown, tip orange. ***Wings*.** Forewing membrane hyaline, with yellow areas at base, area surrounding 1sc-r, 1ra-rp, and area adjacent to proximal region of pterostigma; pterostigma bicolor, orange on proximal ½, pale yellow on distal ½; venation predominantly orange, except wing base, medial region of R + M, subcostal veinlets, 1c-sc, 1ra-rp, 1r-m, and 1m-cu veins brown. Hind wing hyaline, base of subcostal space orange; pterostigma orange with pale brown suffusions, setation blackish; venation mainly pale brown, yellow near wing margin, wing base, and apex of C + Sc. ***Abdomen*.** Tergites I–III yellow with pale brown lateral markings, remaining tergites yellow with brown suffusions; sternites yellow; pleural membrane pale brown.

**Figure 20.**
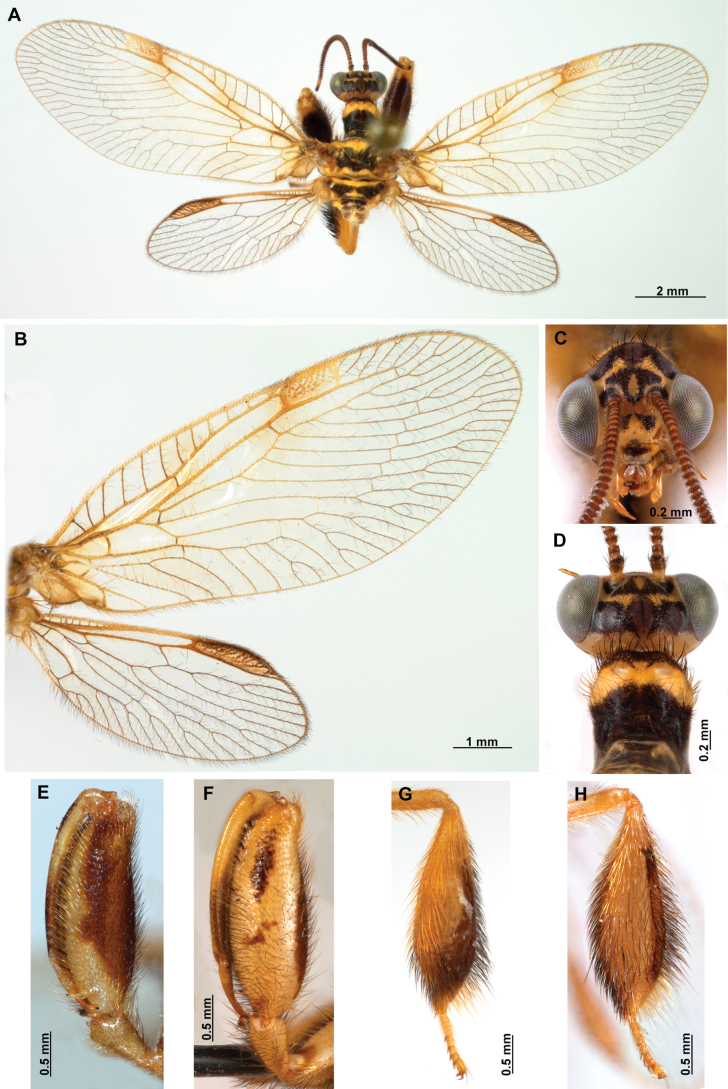
*Anchietaromani* (Esben-Petersen, 1817) **A** male habitus, dorsal (abdomen removed) **B** wings **C** head, frontal **D** pronotum, dorsal **E** forefemur, anterior surface **F** same, posterior surface **G** hind tibia, outer surface **H** same, inner surface.

***Morphology* (Fig. 20). *Head*.** Diamond-shaped in frontal view, smooth; vertexal region domed over compound eyes, laterally with rows of short, reclined setae; paraocular area concave; coronal suture discrete. Antenna moniliform, flagellum with 44–46 flagellomeres, discoidal in shape, except for those of apex narrower; all flagellomeres with medial ring of short setae. Compound eye hemispherical, as wide as ½ of the interocular distance at toruli level. ***Thorax*.** Pronotum nearly as long as wide, with groove contiguous to lateral and distal margins; posterior margin with slight outgrowth; entire surface with abundant, thick setae arising flush the pronotal surface; episternum with interspersed long, thin and short, thickened setae; postfurcasternum quadrangular. Mesonotum wider than long, with abundant long, thickened setae present on medial area; metanotum ~ 3× as wide as long, glabrous. Pteropleura covered with abundant long, thin setae. ***Foreleg*.** Coxa as long as femur, cylindrical, slightly expanded at preapical region, densely covered with fine, long setae; trochanter semi-triangular, densely setose, dorsally with tuft of long, thick setae, anterior surface with blunt process. Femur robust, densely set with long, fine setae; closing surface with posteroventral row of integumentary specializations fully-developed, composed of tubercle-shaped specializations, proximally with spine-shaped, sub-basal process; adjacent row of thickened setae with globular base present on distal ½; anteroventral row of specializations reduced to proximal region and apex, composed of tubercle-shaped specializations; primary process present, curved; adjacent row of thickened setae with globular base present on distal 4/5. Tibia almost as long as femur, curved, glabrous, with a row of prostrate setae on closing surface, and patch of clavate setae apically on anterior surface. Basitarsus with long lanceolate process, with clavate setae on proximal region of anterior surface, ventrally with single row of prostrate setae. ***Mid- and hind leg*.** Mid-leg covered with abundant fine, long setae, tibia slightly widened. Hind leg densely covered with long, fine setae, except on tarsus, shorter; tibia notably widened and laterally flattened, oar-shaped. ***Wings*.** Forewing oval, venation densely setose, trichosors present along wing margin; costal space slightly widened medially, humeral vein simple or forked, 10–12 subcostal veinlets present; pterostigma rectangular; subcostal space with single medially located crossvein; Sc vein abruptly bent posteriad at proximal margin of pterostigma to merge the RA; *rarp2* straight, with four or five veins arising from it, three or four from *rarp1*; M fused basally to R; RP base located near separation of M and R, M fork near such separation; 1r-m located between RP base and M fork forming a small, trapezoidal cell; five or six gradate crossveins present; Cu deeply forked, CuA distally branched; CuP proximally angled, approaching A1, forked; A1 simple, A2 medially forked. Hind wing oval, notably smaller and narrower than forewing; costal space narrow and reduced, with seven or eight veinlets; C and Sc fused at proximal 1/3 of wing length, subcostal space without crossveins; Sc vein abruptly curved posteriad at proximal margin of pterostigma to merge the RA; pterostigma elongated, slightly widened distally; radial space widened with single, straight, crossvein; two or three veins arising from *rarp1*, 1–3 from *rarp2*. M vein forked at R fork level. Cu vein deeply forked, CuA slightly concave, distally forked, first branch simple; CuP distally curved anteriorly near wing margin, with 2–4 branches; A1 arched, A2 short, simple. ***Abdomen*.** Cylindrical, tergites III and IV with short, posteromedial keeled processes in both sexes, tergites V–IX with abundant long, thin setae; sternites densely setose.

**Figure 21.**
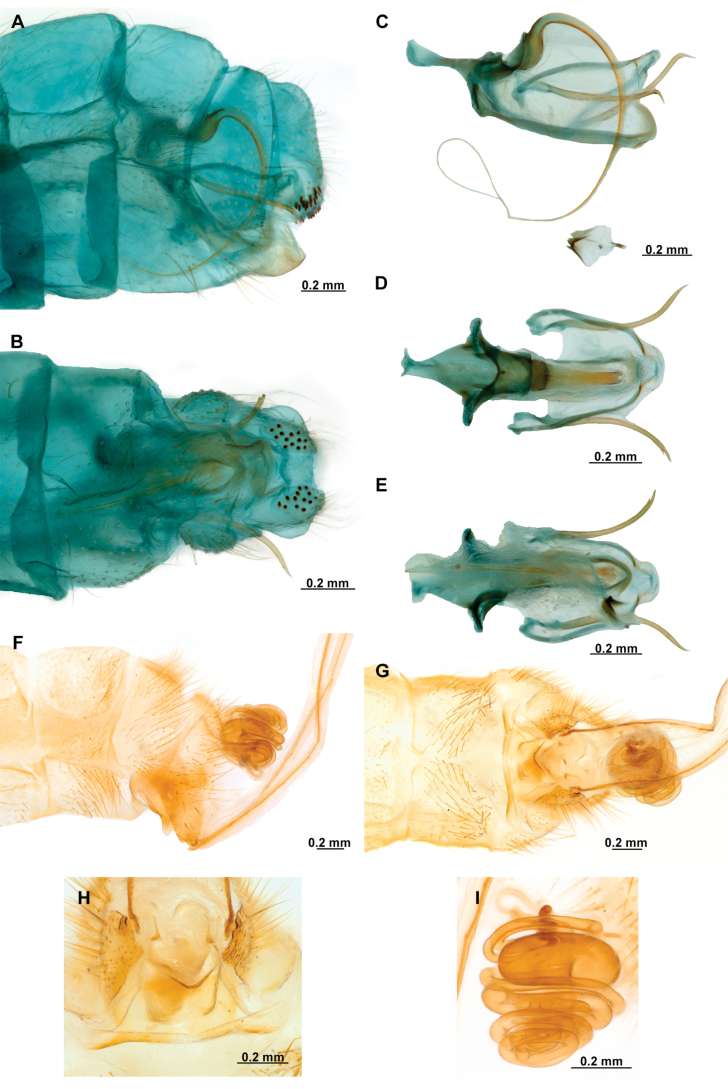
*Anchietaromani* (Esben-Petersen, 1817) **A** male terminalia, lateral **B** same, ventral **C** male genitalia, lateral **D** same dorsal **E** same, ventral **F** female terminalia, lateral **G** same, ventral **H** gonapophyses VIII of female, ventral **I** spermatheca.

***Male genitalia*** (Fig. 21A–E). Tergite IX medially narrower than laterally, posterolaterally with abundant long, thick setae. Sternites VIII and IX fused, fusion line weakly marked; sternite VIII rectangular with abundant long, thickened setae, sternite IX U-shaped, with long, thick setae on anterior ½, posterior ½ glabrous, medially convex, forming a canal, laterally concave. Gonocoxites IX short, sinuous, with base flattened; apex laterally recurved, with two or three longitudinally arranged, preapical processes on outer surface. Ectoproct ovoid, narrow, posteroventrally with patch of 14–16 thick, dark setae; laterally with smooth, glabrous, bulging area; inner area sclerotized, with patch of short, fine setae. Gonocoxites X forming a triangular sclerite; anterior apex laterally flattened, ventrally concave; posterior apex with paired dorsal and lateral processes; base of gonostyli X U-shaped, thickened, concave, set with curved, lateral processes; the rest of the structure whip-like, short, recurved, apical portion forming a loop. Gonapophyses X straight, narrow, anterior apex spatulate, dorsally curved, posterior apex dorsally curved, surrounding membrane set with minute granules; gonapophyses joined by membrane, forming a V-shaped structure. Gonocoxites XI thin, U-shaped, medial lobe flattened, quadrangular, weakly sclerotized at center, anterior margin with U-shaped concavity; lateral arms of gonocoxites XI gently sinuous, anterior apex curved.

***Female genitalia*** (Fig. 21F–I). Sternite VII (gonocoxites VII) formed by two medially fused trapezoidal lateral plates, covered with long and thin setae, posterior margin forming an obtuse angle. Tergite VIII almost as wide medially than laterally, laterally enclosing the spiracle of the segment, lateral margin quadrangular. Gonocoxites VIII forming a narrow trapezoidal plate, with two lateral concavities; gonapophyses VIII medial part forming an enlarged canal, with a depression on ventral surface, apex ventrally curved, rounded; lateral part of gonapophyses VIII forming a chamber-shaped dorsal structure with two lateral concavities. Tergite IX + ectoproct triangular. Gonocoxite IX long, straight, narrow, as long as the last five abdominal segments together. Gonapophyses IX as a pair of tiny sclerites hidden under tergite IX + ectoproct. Bursa copulatrix unsclerotized, long, progressively narrower; spermatheca complex and coiled; proximal section thin, slightly thickened at coiled area, forming three convolutions; medial section thicker than proximal section, long, coiled, forming multiple convolutions; distal section wider than medial section, sac-shaped, forming a loop. Fertilization canal duct long, spiral-shaped, forming three convolutions, slightly preapically expanded; fertilization canal elongated, narrow, J-shaped, covered with microfilaments.

##### Distribution.

Brazil (Amazonas), Peru (San Martín).

##### Remarks.

This species was synonymized with *A.bellus* by Penny (1982a) without examination of the type of *Anisopteraromani* Esben-Petersen, 1917. Both species have basically the same coloration pattern, but the hind tibia of *A.bellus* is narrower than that of *A.romani*, while the shape of the male gonocoxites IX and X are noticeably different. Based on these characters, herein *Anchietaromani* (Esben-Petersen, 1917) is reinstated as a valid species.

Regarding its distribution, the collecting data of the type of *A.romani* appears as “Rio Autaz, Amazon”, locality that was corrected by Penny (1982a) as Autazes, in the Brazilian state of Amazonas. Here, this species is reported from Peru (San Martin) for the first time.

In a recent publication by Rasmussen and Ardila-Camacho (2021) this species was referred as *Anchieta* sp. nov. Since the taxonomic status of *A.romani* was not clarified at that time, the specimens cited in that publication were initially interpreted as belonging to a new species. In that contribution, aspects of the life history of this species like its association with the vespid wasp *Montezumiadimidiata* Saussure, 1852 (Vespidae: Eumeninae) and its mimicry with *Ptilotrigonalurida* (Smith, 1854) were discussed.

Regarding the phylogenetic affinities of this species, it was recovered in a polytomy with *A.apiculasaeva* and a clade containing *A.remipes*, *A.tinctus*, *A.partheniellus*, *A.nebulosus*, and *A.nothus*. Considering the morphology of the male genitalia, this species is closely related with *A.apiculasaeva*. Both species can be separated by the morphology of the male gonocoxites IX and gonocoxites X.

#### ﻿﻿ Anchieta sophiae

Taxon classificationAnimaliaNeuropteraRhachiberothidae

Ardila-Camacho & Contreras-Ramos sp. nov.

https://zoobank.org/F4C36561-8EB8-48D5-8794-4AAC35D49D84

Figs 22 , 23

##### Type locality.

French Guiana, **Camopi**: Mont St-Marcel de la Haute, 2°23'9.816"N, 53°1'29.964"W, 13 Dec. 2014, light trap, J. Touroult leg.

##### Material examined.

***Holotype*** male, pinned. Original label: “French Guiana, **Camopi**, Mont St-Marcel de la Haute, 2°23'9.816"N, 53°1'29.964"W, 13 Dec. 2014, light trap, J. Touroult” MNHN. ***Paratypes*.** French Guiana • 1 ♂; same data as for holotype; CNIN. – **Maripasoula** • 1 ♀; Contreforts du Mitaraka, crique Alama, Fôret vallonnée au pied d’inselbergs; 12 Mar. 2015; light trap; CSCA.

##### Diagnosis.

This species is separated from the yellow morph of *A.fumosellus* because the frons is completely yellow, the pronotum has a dark brown posterior, inverted U-shaped mark, and the pteropleura is nearly entirely yellow. Furthermore, the hind tibia is slightly widened. On the male genitalia, the gonocoxite IX is pointed, and lacks processes. On the female genitalia, the gonocoxites + gonapophyses VIII form an enlarged, chamber-shaped structure.

##### Etymology.

First author dedicates this species after his daughter Lauren Sophia Ardila, born on 29 of December of 2015.

##### Description.

***Measurements*.** Male (*n* = 2). Forewing length: 11.7–12.3 mm; Hind wing length: 6.8–7.0 mm. Female (*n* = 1): Forewing 10.5 length: mm; Hind wing length: 6.1 mm.

**Figure 22.**
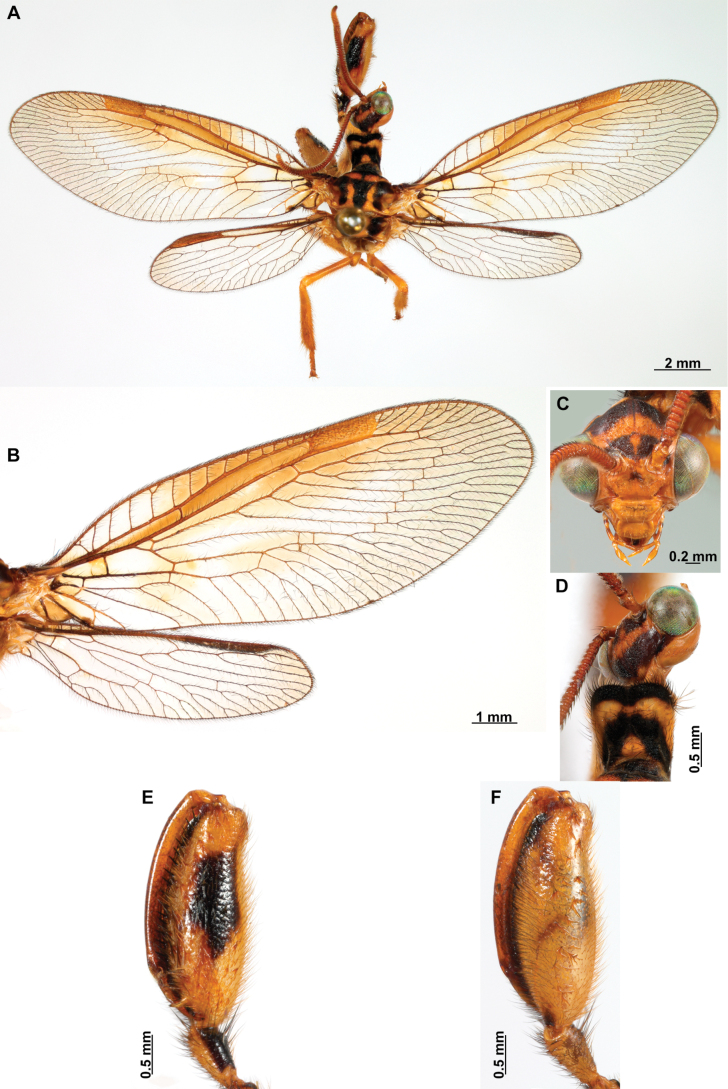
*Anchietasophiae* Ardila-Camacho & Contreras-Ramos, sp. nov. **A** male habitus, dorsal (abdomen removed) **B** wings **C** head, frontal **D** pronotum, dorsal **E** forefemur, anterior surface **F** same, posterior surface.

***Coloration*** (Fig. 22). ***Head*.** Mostly yellow, vertexal region with transversal, dark brown band; with lateral rows of dark brown setae; supra-antennal area with lateral, triangular, dark brown marks. Antennal scape yellow with brown apex, pedicel brown; flagellum pale brown, except apex yellow. Frons with central, dark brown dot; mandible pale brown, with base yellow; maxilla with galea and maxillary palpus pale brown; labium yellow, labial palpus pale brown, palpimacula yellow. ***Thorax*.** Pronotum yellow with dark brown anterior margin and posterior, inverted U-shaped mark. Episternum bicolor, anteriorly dark brown, and posteriorly yellow; postfurcasternum yellow with dorsal, dark brown area. Mesonotum yellow with dark brown anterior margin; lateral regions with C-shaped, dark brown marks; medial region with a triangular, dark brown mark. Metanotum yellow with lateral, dark brown areas; pteropleura yellow, with dark brown suffusions adjacent to pleural sutures. ***Foreleg*.** Coxa yellow with interspersed yellow and black setae; trochanter yellow with brown dorsal surface. Femur posterior surface yellow with small brown areas on base, medial region, and apex; anterior surface yellow with medial, dark brown mark; tibia pale brown with yellow areas at base; basitarsus mostly pale brown, with proximal yellow area on posterior surface; second to fourth tarsomere pale brown. ***Mid- and hind legs.*** Mid-leg with yellow coxa, trochanter, and femur; tibia and tarsus pale brown. Hind leg with yellow coxa, trochanter yellow with pale brown; femur yellow with medial, pale brown ring; tibia yellow with posterior ½ and preapical region pale brown; tarsus pale brown. ***Wings*.** Forewing membrane pale amber, with slightly marked areas on distal ½ of costal field, subcostal, and radial fields, and on the area surrounding 1m-cu, 2m-cu and 1cua-cup, fork of MP, and stems of RP branches; pterostigma pale brown; venation mostly pale brown with dark areas at the base of R+M, Cu, and anal veins. Hind wing hyaline, with costal and subcostal field pale amber; pterostigma pale brown, venation pale brown, with darker areas near wing base. ***Abdomen*.** Tergites pale brown with yellow area adjacent to posterior margins. Sternites yellow, except those of the abdominal segments III-V pale brown.

***Morphology*** (Fig. 22). ***Head*.** Diamond-shaped in frontal view, rugose; vertexal region domed above compound eyes; coronal suture noticeable; paraocular area concave. Compound eye hemispherical, as wide as ½ of interocular distance at toruli level. Antenna moniliform, flagellum with 47–53 flagellomeres, discoidal in shape, those of distal ½ wider, except at apex narrower; all flagellomeres with medial ring of short setae. Maxillary palpus with the first two palpomeres as long as wide, third palpomere 3× as long as wide, fourth slightly shorter than third, fifth as long as fourth; labial palpus with first palpomere 1.5× as long as wide, second 5× as long as wide, third slightly shorter than second palpomere; palpimacula narrowly ovoid. ***Thorax*.** Pronotum as long as wide, with groove contiguous to lateral and distal margins; in lateral view, posterior margin slightly raised, the rest of the surface straight; entire surface with scattered, thick setae, arising flush the pronotal surface. Postfurcasternum quadrangular with anteroventral narrow process. Mesonotum as wide as long with scattered fine setae on medial region; metanotum ~ 3× as wide as long, glabrous. Pteropleura covered with abundant, thin setae. ***Foreleg*.** Coxa slightly shorter than femur, cylindrical, with abundant, fine and long setae; trochanter subtrapezoidal, densely setose, dorsally with a few long, thick setae; anterior surface with blunt process. Femur robust, setose; closing surface with posteroventral row of integumentary specializations fully developed, composed of tubercle-shaped processes, proximally with a more developed sub-basal process; adjacent row of thickened setae with globular base reduced to distal ½; anteroventral row of processes reduced to sub-basal, primary process, and apex; adjacent row of thickened setae with globular base present nearly over the entire ventral surface. Tibia almost as long as femur, curved, glabrous, closing surface with a row of prostrate setae; apical region of anterior surface with a patch of clavate setae. Basitarsus with long lanceolate process; basal ½ with clavate setae on anterior surface, ventrally with a row of prostrate setae. ***Mid- and hind legs.*** Mid- and hind leg covered with abundant fine setae; hindtibia slightly widened; on both legs basitarsus 2.5× as long as wide, second tarsomere slightly longer than wide; third and fourth as long as wide; fifth tarsomere 1.5× as long as wide. ***Wings*.** Forewing oval, venation setose, trichosors present along wing margin except at base; costal space slightly widened medially, humeral vein forked, with 14 or 15 subcostal veinlets; pterostigma rectangular; subcostal space with single, medially located crossvein; Sc vein abruptly bent posteriad at proximal margin of pterostigma to merge the RA; *rarp2* straight with 3–6 veins arising from it, three or four from *rarp1*; M fused basally with R; RP base located near separation of M and R, M fork near such separation; 1r-m located between RP base and M fork, forming a small trapezoidal cell; six or seven gradate crossveins present; Cu deeply forked, CuA apically branched; CuP proximally angled, approaching A1, forked near separation of M and R; A1 simple, A2 forked. Hind wing notably smaller and narrower than forewing; costal space narrow and reduced, with 5–9 subcostal veinlets; C and Sc fused at 1/3 of wing length; Sc vein abruptly curved posteriad at proximal margin of pterostigma, to merge the RA; subcostal space without crossveins; pterostigma elongated, narrow; radial space with single sigmoid crossvein; gradate crossveins absent; two veins arising from each anterior, radial cell. M vein forked at level of R fork; Cu deeply forked, CuA gently bent, distally forked, first branch simple; CuP distally gently bent anteriorly, near posterior wing margin, with two or three branches; A1 simple, A2 short, simple. ***Abdomen*.** Medially widened, tergites quadrangular, without keeled processes; sternites rectangular.

**Figure 23.**
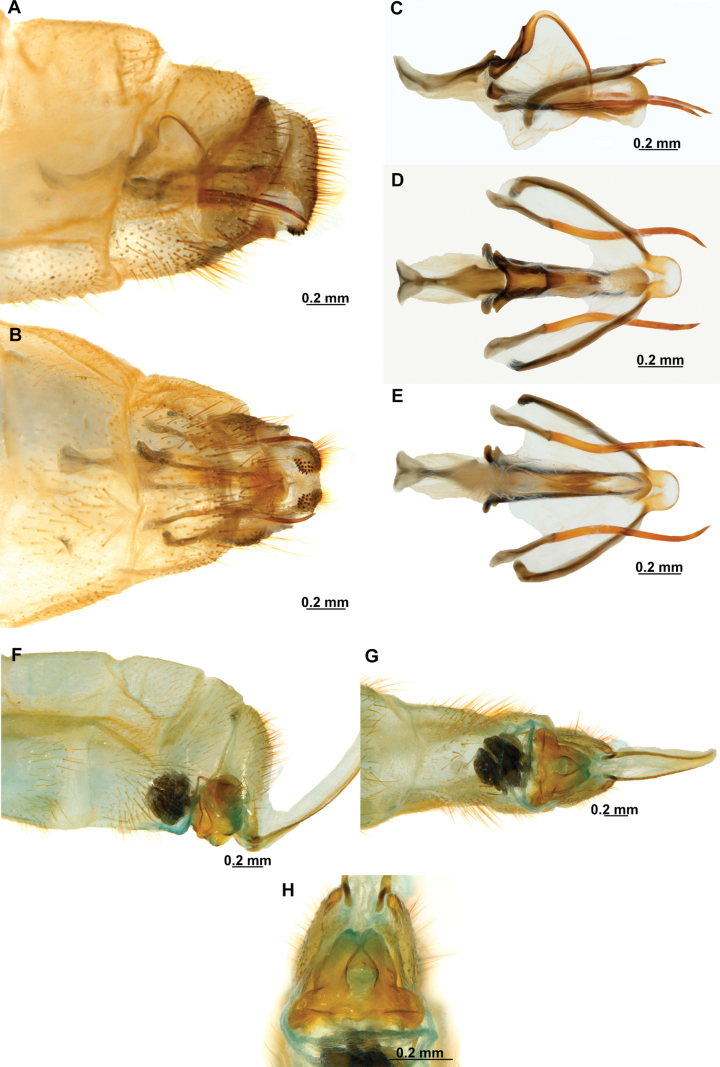
*Anchietasophiae* Ardila-Camacho & Contreras-Ramos, sp. nov. **A** male terminalia, lateral **B** same, ventral **C** male genitalia, lateral **D** same, dorsal **E** same, ventral **F** female terminalia, lateral **G** same, ventral **H** gonapophyses VIII of female, ventral.

***Male genitalia*** (Fig. 23A–E). Tergite IX notably narrower medially than laterally, lateral margin with posterior concavity. Sternites VIII and IX fused, fusion line moderately distinct; sternite IX hourglass-shaped, dorsally forming a canal. Gonocoxite IX sinuous, longer than gonapophyses X, posterior apex pointed, lacking processes. Ectoproct trapezoidal, narrow; lateral apex convex, transparent; posteroventrally set with a patch 16–19 thick, conical setae. Gonocoxites X ventrally canaliculated, hourglass-shaped, curved; posterior ½ with lateral and dorsal processes; base of gonostylus X thickened, triangular with lateral, basal processes; the rest of the structure whip-shaped, short, with apical portion incurved. Gonapophyses X rod-shaped, with dorsally curved tips; both joined by membrane, forming a V-shaped structure. Gonocoxites XI narrow, U-shaped, medial lobe flattened, rounded; lateral arms slightly straight, anterior apex bent ventrally.

***Female genitalia*** (Fig. 23F–H). Sternite VII noticeably wider laterally than medially, with posterior margin broadly concave. Tergite VIII slightly narrower medially than laterally, enclosing the spiracle of the segment, lateral margin quadrangular. Gonocoxites VIII and medial part of gonapophyses VIII completely fused, forming an enlarged, chamber-shaped structure; lateral part of gonapophyses VIII as a concave, narrow plate, hidden by the tergite IX + ectoproct. Tergite IX + ectoproct triangular. Gonocoxites IX elongated, sinuous, and narrow, as long as the last five abdominal segments together. Gonapophyses IX not discernible. Bursa copulatrix narrow, short, membranous. Spermatheca complex and coiled; proximal section long and thin, forming several coils; medial section thicker than proximal section, forming several coils; distal section wider than medial section, progressively expanded towards apex, sac-like, forming a convolution; fertilization canal duct, long, spiral-shaped, forming several convolutions; fertilization canal elongated, thin, J-shaped, covered with microfilaments.

##### Distribution.

French Guiana (Camopi, Maripasoula).

##### Remarks.

This species is known solely from two provinces of French Guiana. Based on its coloration pattern, wing morphology and shape of the hind tibia this species appears to be sister to *A.fumosellus*. However, considering the genitalic morphology this species could be sister of the clade of bee- mimicking species of *Anchieta*. The coloration pattern of *A.sophiae* sp. nov. is markedly similar to that of the yellow form of *A.fumosellus*, although its model is likely *Agelaiamyrmecophila* (Ducke, 1905) (Vespidae: Polistinae) based on overall coloration and distribution of these species.

#### ﻿﻿ Anchieta tinctus

Taxon classificationAnimaliaNeuropteraRhachiberothidae

Ardila-Camacho & Contreras-Ramos sp. nov.

https://zoobank.org/432E07BA-5CB1-4BC3-9D9A-993D067488CA

Figs 24 , 25

##### Type locality.

Costa Rica, **Heredia**: Est. Biol. La Selva, Arboleda, 10°26'N, 84°01'W, 50–150 m, 28 Jan. 1998, R. Vargas C. leg.

##### Material examined.

***Holotype*** male, pinned. Original label: “Costa Rica, **Heredia**, Est. Biol. La Selva, Arboleda, 10°26'N, 84°01'W, 50–150 m, 28 Jan. 1998, R. Vargas C. leg.”, “INBIOCRI002286798”, MNCR. ***Paratypes*.** Costa Rica – **Heredia** • 1 ♀; Est. Biol. La Selva, Arboleda; 10°26'N, 84°01'W; 50–150m; 28 Jan. 1998; R. Vargas C. leg.; INBIOCRI002286797; MNCR. – **Limón** • 1 ♂; Sector Cocori, 30 Km al N. de Cariari; 100 m; Jan. 1993; E. Rojas leg.; L-N 286000, 567500, 1753, CRI001675541; MNCR.

• 1 ♂; Sector Cocori; 150 m; Feb. 1993; E. Rojas leg.; L-N 286000, 567500, CRI001309729; MNCR. • 1 ♂; Manzanillo, RNFS Gandoca y Manzanillo; 0–100 m; 20–30 Nov. 1992; F.A. Quesada leg.; L-S 398100, 610600, CRI000784237; MNCR.

##### Diagnosis.

This species has the body nearly uniformly dark brown, the antennal flagellum has seven yellow preapical flagellomeres. The forewing membrane is mostly pale amber, with darker areas on basal ½ and area adjacent to proximal part of pterostigma; there are hyaline, fenestrated, whitish areas on costal and subcostal fields, anterior radial cells, *mcu2*, *mcu3*, *cuacup1*, and second *a1a2*; wing base, anal region and jugal lobe are dark amber. The hind tibia is conspicuously expanded and laterally flattened, with most of the surface including the apex pale brown to orange. On the male genitalia, the sternite IX is quadrangular, dorsally canaliculate, and curved ventrally. The gonocoxite IX is reduced, appearing as a filiform, thin sclerite, ventrally attached to lateral arms of gonocoxites XI. The gonostyli X are whip-like, dorsally curved and posteroventrally directed, forming an apical loop. The gonapophyses X are posteriorly fused forming a long, blunt process. On the female genitalia, the gonapophyses VIII medial part is composed by quadrangular plates that form a canal, whose ventral surface has a longitudinal depression. The bursa copulatrix is thin, moderately sclerotized, and remarkably long, forming a loop.

##### Etymology.

The specific epithet of this species is a noun in apposition in allusion to the wing coloration pattern of this species.

##### Description.

***Measurements*.** Male (*n* = 4). Forewing length: 10.42–11.85 mm; Hind wing length: 5.81–7.33 mm. Female (*n* = 1): Forewing length: 10.20 mm; Hind wing length: 6.63 mm.

**Figure 24.**
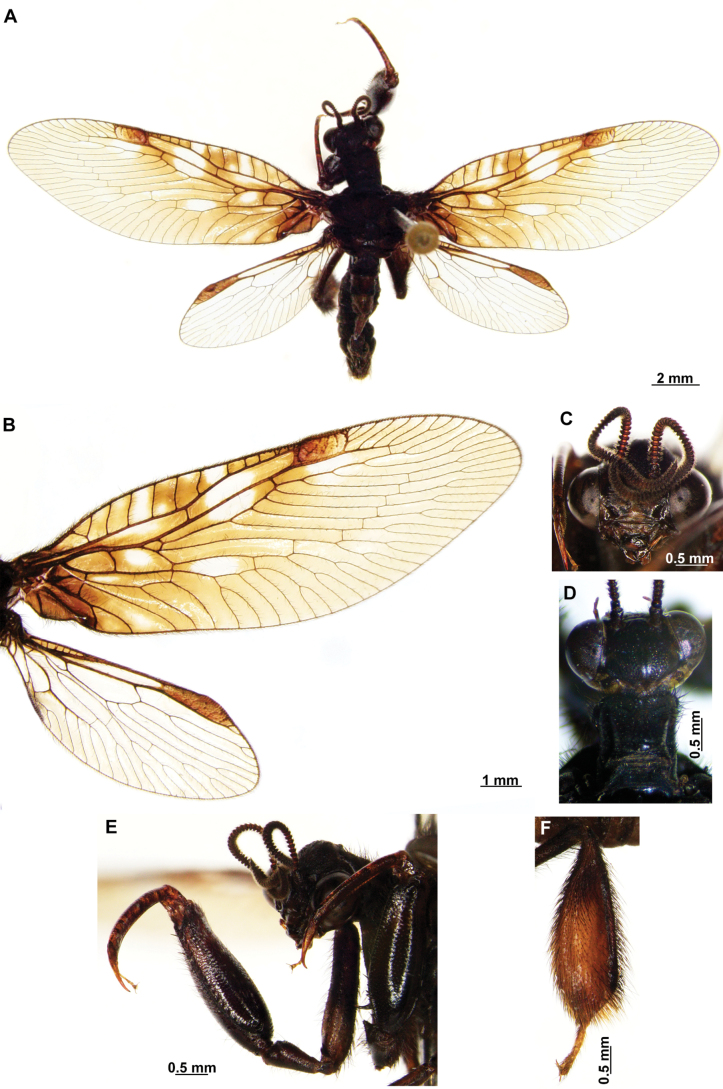
*Anchietatinctus* Ardila-Camacho & Contreras-Ramos, sp. nov. **A** male habitus, dorsal **B** wings **C** head, frontal view **D** pronotum, dorsal **E** forelegs **F** hind tibia, outer surface.

***Coloration*** (Fig. 24). ***Head*.** Mostly dark brown; region of the vertex dark brown with lateral rows of dark brown setae. Postgena pale brown. Antennal scape and pedicel dark brown, flagellum dark brown, except for seven yellow preapical flagellomeres. Frons pale brown. Palpimacula brown. ***Thorax*.** Pronotum dark brown with black bristles; episternum and postfurcasternum dark brown. Meso- and metanotum dark brown, mesonotum with dark brown bristles. Pteropleura dark brown. ***Foreleg*.** Coxa dark brown with paler apex, covered with dark brown to black setae; trochanter dark brown. Femur mostly dark brown; tibia dark brown, clavate setae pale brown; basitarsus pale brown, with amber apex, second to fourth tarsomere pale brown. ***Mid- and hind leg*.** Mid-leg with coxa, trochanter and femur dark brown; tibia mostly dark brown, apex pale brown, entire surface with dark brown to black setae; tarsus pale brown. Hind leg with coxa, trochanter and femur brown, with dark brown setae; outer surface of tibia with base, posterior margin and preapical region dark brown, the rest of the surface including the apex pale brown to orange; inner surface with same pattern as outer surface, sometimes covered with dark brown to black setae; tarsus pale brown. ***Wings*.** Forewing membrane mostly pale amber, with darker areas on basal ½ and area adjacent to proximal part of pterostigma; with hyaline, fenestrated, whitish areas on costal and subcostal fields, anterior radial cells, *mcu2*, *mcu3*, *cuacup1*, and second *a1a2*; wing base, anal region and jugal lobe dark amber; pterostigma brown, becoming paler towards distal region; venation uniformly brown. Hind wing mostly hyaline with area adjacent to posterior and distal margin pale amber, base of wing brown, costal space and base of subcostal field amber; brown pterostigma, uniformly brown venation, dark brown setation. ***Abdomen*.** Brown with dark brown margins of sclerites; pleural membrane dark brown.

***Morphology*** (Fig. 24). ***Head*.** Diamond-shaped in frontal view, rugose; vertexal region domed above compound eyes, with lateral rows of short, reclined setae; coronal suture distinct; paraocular area concave. Antenna moniliform, flagellum with 51 flagellomeres, discoidal in shape, those of apex narrower; all flagellomeres with medial ring of short setae. Compound eye hemispheric, as wide as ½ of the interocular distance at toruli level. ***Thorax*.** Pronotum slightly longer than wide, with groove contiguous to lateral and distal margins of pronotum; in lateral view, posterior margin slightly elevated, the rest of the surface is straight; entire surface with abundant thick setae arising flush the pronotal surface. Mesonotum slightly wider than long, with abundant thick and long setae; metanotum ~ 3× as wide as long. Pteropleura covered with abundant fine, long setae. ***Foreleg*.** Coxa as long as femur, cylindrical, slightly distally widened, with abundant fine, long setae; trochanter subtrapezoidal, densely setose, dorsally with a tuft of long, thick setae, anterior surface with blunt process covered with long setae. Femur robust, with abundant fine, long setae; closing surface with posteroventral row of integumentary specializations fully developed, composed of tubercle-shaped processes with conical Stitz organs (process/seta ratio 1:1 or 2:1), proximally with spine-shaped, sub-basal process (process/seta ratio 4: 1); adjacent row of thickened setae with globular base reduced to distal ¼; anteroventral row of specializations reduced to proximal region and apex, composed of tubercle-shaped processes, with conical setae (process/seta ratio 1:1); basal, primary process present, spine-shaped, curved (process/seta ratio 7:1); adjacent row of thickened setae with globular base present on distal 4/5. Tibia almost as long as femur, curved, glabrous, closing surface with a row of prostrate setae, with patch of clavate setae apically on anterior surface. Basitarsus with long lanceolate process, proximal ½ with clavate setae on anterior surface, ventrally with a row of prostrate setae. ***Mid- and hind leg*.** Mid-leg with coxa, trochanter and femur set with long and fine setae, tibia moderately medially expanded, with abundant long and thick setae, tarsus covered with short setae, basitarsus as long as the next three together. Hind leg with coxa, trochanter and femur covered with long, fine setae; tibia conspicuously expanded and laterally flattened, oar-shaped, densely setose; tarsomeres with short, thin setae, basitarsus as long as the next three tarsomeres together. ***Wings*.** Forewing oval, venation setose, trichosors present along wing margin except at base; costal space slightly widened medially, humeral vein sometimes forked, with 11–14 subcostal veinlets; pterostigma rectangular; subcostal space with single crossvein, medially located; Sc vein abruptly bent posteriad at proximal margin of pterostigma to merge the RA; *rarp2* straight with three or four veins arising from it, three or four from *rarp1*; M fused basally to R; RP base located near separation of M and R, M fork near such separation; 1r-m located between RP base and M fork forming a small trapezoidal cell; seven or eight gradate crossveins present; Cu deeply forked, CuA apically branched, CuP proximally angled, approaching A1, forked slightly before the level of separation of M and R; A1 simple, A2 forked. Hind wing notably smaller and narrower than forewing, oval; costal space narrow and reduced, with 7–9 crossveins; C and Sc fused at 1/3 of wing length, subcostal space without crossveins; Sc vein abruptly curved posteriad at proximal margin of pterostigma to merge the RA; pterostigma elongated, slightly distally expanded; radial space with single crossvein, sinuous; two or three veins arising from *rarp1*, one or two from *rarp2*. M forked at or slightly before R fork. Cubitoanal space with distal crossvein. Cu vein deeply forked, CuA slightly concave, distally forked, first branch simple; CuP distally anteriorly bent near wing margin, with two or three branches; A1 arched A2 short, simple. ***Abdomen*.** Medially widened, tergites of abdominal segments III and IV with posteromedial keeled processes. Sternites covered with long, fine setae, gradually becoming longer towards the last abdominal segments.

**Figure 25.**
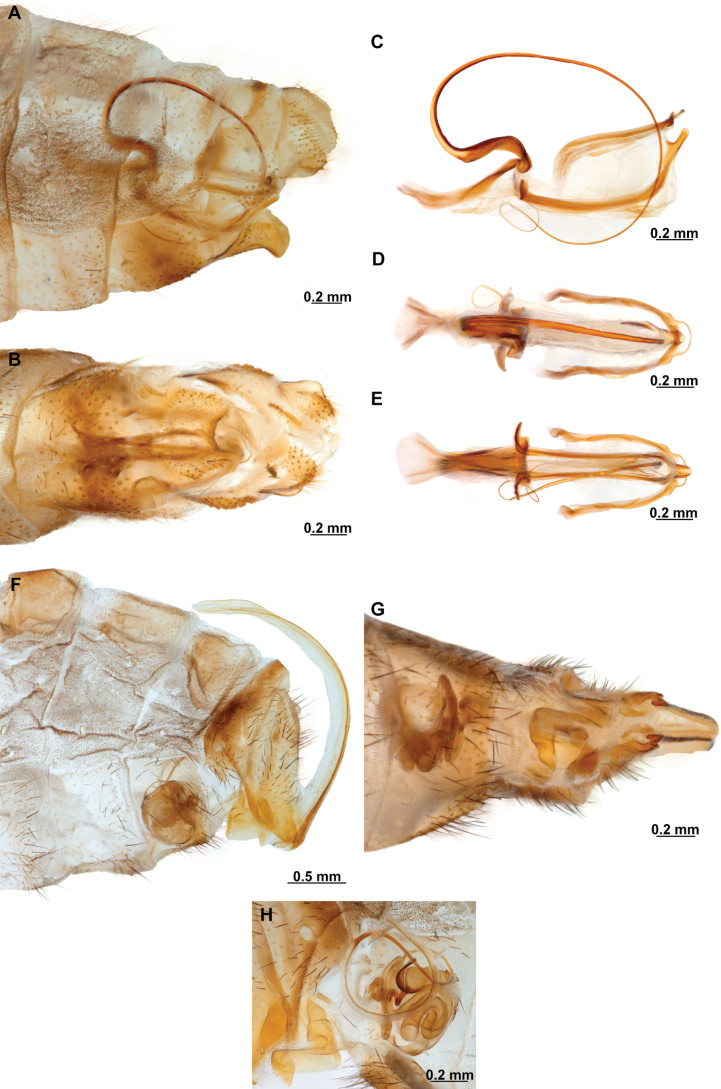
*Anchietatinctus* Ardila-Camacho & Contreras-Ramos, sp. nov. **A** male terminalia, lateral **B** same, ventral **C** male genitalia, lateral **D** same, dorsal **E** same, ventral **F** female terminalia, lateral **G** same, ventral **H** spermatheca.

***Male genitalia*** (Fig. 25A–E). Tergite IX remarkably narrower medially than laterally, lateral region expanded, with blunt process. Sternites VIII and IX fused, with weakly marked fusion line; sternite VIII setose; sternite IX quadrangular with blunt corners, dorsally canaliculate, curved ventrally. Gonocoxites IX reduced, present as fusiform, helical, short and thin sclerites, ventrally attached to lateral arms of gonocoxites XI. Ectoproct trapezoidal, ventrolaterally produced into a rounded, smooth, glabrous outgrowth; ventral surface concave with slightly sclerotized plate on inner side, set with short, thin setae on posterior ½. Gonocoxites X forming a flattened, medially constricted sclerite; anterior ½ expanded, spatulate, posterior ½ triangular, straight; gonostyli X with thickened base, set with short lateral processes articulated to gonocoxites X, medially bent posterodorsally, canaliculated on dorsal surface; the rest of the structure dorsally curved, whip-shaped, forming an apical loop. Gonapophyses X joined by membrane, parallel ventral view; elongated, curved, narrow, with anterior apex spatulate, bent laterally; posterior apex fused to form a long blunt process, dorsally projected. Gonocoxites XI narrow, U-shaped, medial lobe flattened, rounded, medially less sclerotized; lateral arms of gonocoxites XI short, thin, anterior apex bent ventrally.

***Female genitalia*** (Fig. 25F–H). Sternite VII (gonocoxites VII) as two trapezoidal plates joined by posteromedial bridge; posterior margin broadly concave. Tergite VIII medially narrower than laterally, enclosing spiracle of the segment, lateral margin quadrangular. Gonocoxites VIII as subrectangular, concave, sclerotized plate; gonapophyses VIII medial part composed of quadrangular plates forming a canal, whose ventral surface has longitudinal depression; lateral part of gonapophyses VIII a trapezoidal plate. Tergite IX + ectoproct elongated triangular. Gonocoxites IX elongated, curved and narrow, as long as the last four abdominal segments together. Gonapophyses IX as two small, oval sclerites located behind the gonocoxites IX base. Bursa copulatrix is thin, moderately sclerotized, remarkably long, forming a loop. Spermatheca long, complex and coiled; proximal section narrow, forming three convolutions; medial section gradually expanded, wider than proximal section, forming few convolutions; distal section markedly expanded, wider than medial section, sac-shaped. Fertilization canal duct long, spiral-shaped, forming three convolutions, with preapical widening; fertilization canal elongated, narrow, J-shaped, covered with microfilaments.

##### Distribution.

Costa Rica (Heredia, Limón).

##### Remarks.

This species is known only from two provinces of Costa Rica. Regarding its phylogenetic relationships, this species was recovered sister to *A.remipes* as both share many characteristics on the wings and male genitalia. Specifically, the genital sclerites in this pair of species is highly modified, with reduced male gonocoxites IX and fused gonapophyses X at the posterior apex. Besides the coloration pattern, this species can be separated from *A.remipes* by the morphology of the male sternite IX. This species probably mimics *Meliponafuliginosa* Lepeletier, 1836, a species of stingless bee with similar coloration and size found in Costa Rica.

#### ﻿﻿Genus Plega

Taxon classificationAnimaliaNeuropteraRhachiberothidae

Navás, 1928

Plega Navas, 1928: 326. Type species: Symphrasissignata Hagen, by original designation.

##### Further description.

Parker and Stange (1965): 605; Penny (1982a, b): 421; Penny and da Costa (1983): 617.

##### Taxonomy.

Parker and Stange (1965): 605 (divided *Plega* into two groups based on type of antenna and host selection); Werner and Butler (1965): 67 (divided *Plega* into two groups based on type of antennae which corresponds to host selection); Tjeder (1968): 16–17 (*Plega* = *Trichoscelia*).

##### Key to species.

Rehn (1939b): 240–241 (U.S. species); Penny (1982a): 421–422 (Brazilian Amazon); Penny and da Costa (1983): 617 (Brazil).

##### List of species.

Penny (1977): 37; Ardila-Camacho et al. (2019): 352.

##### Diagnosis.

This genus differs from the other Symphrasinae by having a slightly to strongly domed region of the vertex; compound eyes generally are as wide as ½ of the interocular distance at toruli level. The pronotum is typically as long as wide with anterior, medial, and posterior regions unevenly raised, and covered with pedicellate thickened setae; the postfurcasternum is generally ventrally fused, collar-like (unfused in *P.insolita*). On the forefemur, the closing surface presents both rows of processes fully developed in one species, while the rest has the anteroventral row reduced to proximal ½ and apical portion; the anteroventral row of processes generally presents the basal, primary, curved process; the posteroventral row has two primary processes on the proximal ½ of the femur length, and the proximal part of the row has two spine-shaped processes that may be secondary or tertiary; the distal region of the row is slightly keeled and composed mostly of tubercle-shaped specializations; between the cuticular processes, there are stinger-shaped setae. The rows of thickened setae with globular base adjacent to each row of processes are modified, with the posteroventral row reduced to the apical portion, while the anteroventral row is at least present on the distal ½ of the femur. The fore basitarsus has the lanceolate process is generally long, reaching the middle of the fourth tarsomere, although in one species it is short, reaching the middle of the third tarsomere. The distal section of the spermatheca generally has an apical diverticulum, and the proximal part of the fertilization canal duct is triangular and concave.

##### Description.

***Head*.** Diamond-shaped or quadrangular in frontal view, rugose, region of vertex domed over compound eyes, paraocular area concave; coronal suture generally distinct. Antenna moniliform to filiform, basal flagellomeres from discoidal to as long as wide in frontal view. Compound eye hemispheric, as wide as ½ of interocular distance at toruli level. ***Thorax*.** Pronotum either as long as wide or slightly longer than wide, a groove adjacent to lateral and distal margins is generally present; anterior margin, and medial and posterior regions are generally unevenly raised, covered with long, thickened, generally pedicellate setae; postfurcasternum generally unpaired, and ventrally fused. Mesonotum wider than long, with long, thickened, pedicellate setae, metanotum generally 2.5× as wide as long, glabrous. ***Foreleg*.** Coxa slightly shorter than femur, cylindrical, with pedicellate setae or not. Trochanter trapezoidal, generally with protuberance on anterior surface. Femur narrow to robust, closing surface covered generally with microtrichia; posteroventral row processes fully developed, slightly carinated on distal ½ or ⅓, composed of tubercle-shaped specializations and stinger-shaped setae; two primary, spine-shaped specializations are present on proximal ½; secondary or tertiary processes are frequent between the primary ones, and on proximal portion of the row; adjacent row of thickened setae with globular base typically reduced to distal ¼ of femur length; anteroventral row generally reduced to proximal ½ and apex, fully-developed in one species, composed of tubercle-shaped integumentary specializations, and stinger-shaped setae; basal, primary, spine-shaped process is generally present, curved (absent in *P.insolita*); adjacent row of thickened setae with globular base present on distal ½–⅘. Tibia nearly as long as femur, setose, curved or straight, ventrally keeled, with a row of prostrate setae; anterior surface with a patch of clavate setae at apex. Basitarsus generally with long lanceolate process surpassing distal margin of third tarsomere (reaching to the middle of the third tarsomere in *P.insolita*), equipped with a plug-shaped Stitz organ at apex; basal ½ with a row of prostrate setae on ventral surface, and patch of clavate setae on anterior surface; second tarsomere articulated on anterior surface of basal ½ of basitarsus, longer than third and fourth tarsomeres together; pretarsal claws simple. ***Mid- and hind leg*.** Unmodified, basitarsus typically 3.5–4× as long as wide. ***Wings*.** Forewing oval, trichosors present along wing margin, except at base; costal space medially slightly widened, humeral vein generally branched, subcostal veinlets generally simple, sometimes with some forked; pterostigma elongated and narrow; Sc vein abruptly bent posteriad at proximal margin of pterostigma to merge the RA; radial space with two crossveins; *rarp2* curved; RP separated or close to separation of M and R, M fork near this separation; 1r-m between RP base and M fork forming a trapezoidal cell; RP with single series of gradate crossveins; CuP basally angled, approaching A1. Hind wing oval, smaller and narrower than forewing; costal space narrow and reduced, C and Sc fused at proximal ¼ of wing length; Sc vein abruptly curved posteriad at proximal margin of pterostigma to merge the RA; pterostigma elongated, curved, narrow; radial space with single crossvein, generally straight; RP with single gradate series. M forked beyond R fork; Cu deeply forked, CuA distally branched, first branch generally candelabrum-shaped; intracubital vein subparallel to longitudinal wing axis; CuP distally anteriorly bent near posterior wing margin, pectinate. Cubitoanal space generally with two crossveins. ***Abdomen*.** unmodified.

***Male genitalia*.** Sternites VIII and IX not fused; sternite IX pentagonal or trapezoidal, posteromedial lobe dorsally canaliculated, generally triangular. Gonocoxites IX generally long, thin to thickened, with or without apical processes. Ectoproct generally ovoid or trapezoidal, sometimes with pedicellate setae, sometimes developed as prominent spine-shaped structures; ventral surface typically sclerotized, with anterior rounded, flattened lobe that is continuous with ventromedial, curved sulcus. Gonocoxites X unpaired, forming a ventrally canaliculated, bar-shaped sclerite, anterior apex generally slightly expanded, straight or dorsally bent; posterior apex with paired dorsal and lateroventral, preapical processes, articulated to gonostyli X and gonapophyses X, respectively; gonostyli X with thickened base, set with curved, lateral processes; the rest of the structure from short and recurved to long and coiled, forming one or two internal loops, before protruding from abdomen. Gonapophyses X paired, straight, thin, forming a V-shaped structure; gonapophyses joined by membrane that forms a prominent covering over gonostyli X base, which may be medially sclerotized or not. Gonocoxites XI U-shaped, medial lobe elaborated, with two differentiated parts, a dorsal arch and lobe and ventral convex area, continuous with a process; between these parts a less sclerotized is present; lateral arms of gonocoxites XI straight or sinuous, with anterior apex bent or expanded. Hypandrium internum triangular, keeled, with two lateral fins.

***Female genitalia*.** Sternite VII (gonocoxites VII) generally trapezoidal, in a species (*P.drepanicoides*) as two lateral, medially joined, trapezoidal plates. Tergite VIII narrower medially than laterally, enclosing the spiracle of the segment VIII. Gonocoxites VIII forming a narrow plate; gonapophyses VIII medial part keel-shaped, with posteromedial bilobed or bifid process; lateral part as a trapezoidal or oval plate, hidden under tergite IX + ectoproct. Tergite IX + ectoproct triangular, ovoid or D-shaped. Gonocoxites IX long, straight and narrow; gonapophyses IX sometimes present as tiny sclerites, located basally, on inner surface of gonocoxites IX. Bursa copulatrix variable in shape, with sclerotized portion or not, short to long, sometimes with an enlarged chamber-shaped part. Spermatheca short and simple to remarkably long and entangled; proximal section short to long and thin, generally forming several coils; medial section thin, entangled or forming a long spiral; distal section moderately expanded, wider than proximal and medial sections, generally forming a blunt diverticulum at apex; fertilization canal duct short, proximal part triangular and concave; fertilization canal generally elongated, pod-shaped, covered with microfilaments.

##### Included species.

1. *P.acevedoi* sp. nov. (Mexico)

2. *P.banksi* Rehn, 1939 (Mexico, USA)

3. *P.bowlesi* Ardila-Camacho & Contreras-Ramos, sp. nov. (Ecuador)

4. *P.dactylota* Rehn, 1939 (Mexico, USA)

= *P.fratercula* Rehn, 1939, new synonym

5. *P.disrupta* Ardila-Camacho & Contreras-Ramos, sp. nov. (Mexico)

6. *P.drepanicoides* Ardila-Camacho & Contreras-Ramos, sp. nov. (Mexico)

7. *P.duckei* Penny, 1982 (Brazil)

8. *P.flammata* Ardila-Camacho & Contreras-Ramos, sp. nov. (Mexico)

9. *P.fumosa* Linsley & MacSwain, 1955 (Mexico)

10. *P.hagenella* (Westwood, 1867) (Brazil, Colombia, Costa Rica, Ecuador, French Guiana, Guyana, Mexico, Nicaragua, Panama, Trinidad and Tobago, Venezuela)

= *P.beardi* Penny, 1982, new synonym

= *P.melitomae* Linsley & MacSwain, 1955, new synonym

= *Mantispacognatella* Westwood, 1867

11. *P.insolita* Ardila-Camacho & Contreras-Ramos, sp. nov. (Mexico)

12. *P.lachesis* Ardila-Camacho & Contreras-Ramos, sp. nov. (Guatemala)

13. *P.longicornis* Ardila-Camacho & Contreras-Ramos, sp. nov. (Mexico)

14. *P.megaptera* Ardila-Camacho & Contreras-Ramos, sp. nov. (Mexico)

15. *P.mixteca* Ardila et al., 2019 (Guatemala, Mexico)

16. *P.obtusa* Ardila-Camacho & Contreras-Ramos, sp. nov. (Mexico, USA)

17. *P.oswaldi* Ardila-Camacho & Contreras-Ramos, sp. nov. (Mexico)

18. *P.paraensis* Penny, 1982 (Brazil)

19. *P.pseudohagenella* Ardila-Camacho & Contreras-Ramos, sp. nov. (Costa Rica, Guatemala)

20. *P.radicaudata* Ardila-Camacho & Contreras-Ramos, sp. nov. (Bolivia, Peru)

21. *P.signata* (Hagen, 1877) (Mexico, USA)

22. *P.sonorae* Ardila et al., 2019 (Mexico, USA)

23. *P.spinosa* Ardila et al., 2019 (Mexico)

24. *P.stangei* Ardila et al., 2019 (Mexico)

25. *P.vangiersbergenae* Ardila-Camacho, sp. nov. (Guatemala, Mexico)

26. *P.variegata* Navás, 1928 (Mexico), nomen dubium

27. *P.yucatanae* Parker & Stange, 1965 (Guatemala, Honduras, Mexico)

28. *P.zikani* Navás, 1936 (Brazil)

##### Biology.

Summarized in Ardila-Camacho et al. (2021b).

##### Etymology.

Uncertain, and not specified in the original description of the genus by Navás (1928).

### ﻿﻿Key to the species of *Plega*

**Table:** 

1	Forefemur remarkably elongated and narrow in lateral view (Fig. 63F)	**2**
–	Forefemur narrow and short or moderate- to strongly robust in lateral view (Fig. 32F)	**5**
2	Forefemur closing surface with anteroventral row of integumentary specializations fully developed (Fig. 63E, F)	***Plegaradicaudata* Ardila-Camacho & Contreras-Ramos, sp. nov.**
–	Forefemur closing surface with anteroventral row of integumentary specializations reduced to proximal region and apex	**3**
3	Antennal flagellum brown with eight pale, preapical flagellomeres (Fig. 77A)	***Plegazikani* Navás, 1936**
–	Antennal flagellum uniformly brown	**4**
4	Antennal flagellum with basal flagellomeres as wide as long (Fig. 60E)	***Plegaparaensis* Penny, 1982**
–	Antennal flagellum with basal flagellomeres 3× as wide as long and subconical (Fig. 38C)	***Plegaduckei* Penny, 1982**
5	Posterior surface of forefemur pale with numerous brown dots (Fig. 26F)	**6**
–	Posterior surface of forefemur pale with brown areas, which may or may not be interconnected (Fig. 28F)	**17**
6	Pale medial area of forewing pterostigma with anterior notches (Fig. 44B); posteroventral region of male ectoproct with a flattened lobe covered with clear, conical setae (Fig. 45A)	**7**
–	Pale medial area of pterostigma with different pattern, or if notched, additional smaller pale areas are present (Fig. 61B); male ectoproct lacking posteroventral flattened lobe with clear, conical setae	**8**
7	Posterior apex of male gonocoxite IX equipped with long and thin preapical process on inner surface, and 2–4 shorter, apical processes (Fig. 45C–E)	***Plegahagenella* (Westwood, 1867)**
–	Posterior apex of male gonocoxite IX equipped with an elongate preapical process on inner surface and seven or eight short processes on outer surface, arranged in a single plane (Fig. 31C–E)	***Plegabowlesi* Ardila-Camacho & Contreras-Ramos, sp. nov.**
8	Supraantennal region unmodified, without lateral protuberant areas (Fig. 48C, D); male gonocoxite IX posterior apex bifid (49C–E); male ectoproct posterior surface set with abundant, stout, dark setae (Fig. 49A)	***Plegalachesis* Ardila-Camacho & Contreras-Ramos, sp. nov.**
–	Supraantennal region with lateral protuberances covered with reclined setae (Fig. 69C); male gonocoxite lacking digitiform processes or with more than two processes; male ectoproct posterior surface with unmodified setae or with setae arising from protuberant or spiniform bases (Figs 70A, 72A)	**9**
9	Male gonocoxites IX lacking digitiform processes (Fig. 70C–E)	**10**
–	Male gonocoxites IX se with 3–12 digitiform processes	**15**
10	Antennal flagellum completely brown (Fig. 34A); forefemur anterior surface entirely dark brown (Fig. 34E); male ectoproct lacking modified setal bases (Fig. 35A)	**11**
–	Antennal flagellum with 3–5 pale, preapical flagellomeres (Fig. 50D); forefemur anterior surface with base (area adjacent to primary process) pale, brown on the rest (Fig. 50E); male ectoproct bearing modified setal bases on posteroventral surface (Fig. 51A)	**12**
11	Pale area of pterostigmata enlarged, cream colored (Fig. 56B); forewing generally with a dark brown mark extended from 1m-cu to apical part of intracubital space forming an obtuse angle (Fig. 56B); male sternite IX with thickened, black setae on preapical area (Fig. 57B)	***Plegaobtusa* Ardila-Camacho & Contreras-Ramos, sp. nov.**
–	Pale area of pterostigmata small (Fig. 34B); forewing with strong disruptive pattern, with pale wing tips (Fig. 34B); male sternite IX with thin setae over the entire surface (Fig. 35B)	***Plegadisrupta* Ardila-Camacho & Contreras-Ramos, sp. nov.**
12	Forewing hyaline (Fig. 58B); sternite IX broadly rounded (Fig. 59B); male ectoproct with pedicellate setae on posteroventral area (Fig. 59A)	***Plegaoswaldi* Ardila-Camacho & Contreras-Ramos, sp. nov.**
–	Forewing with area adjacent to crossveins and area between apical twigging amber (Fig. 26B); sternite IX trapezoidal (Fig. 27B); male ectoproct with prominent spiniform setal bases on the posteroventral area (Fig. 27A)	**13**
13	Area adjacent to frontal sutures strongly sunken (Fig. 69C); antennal scape short, lacking stout setae (Fig. 69C, D); male genitalia with ventral process of the median lobe of the gonocoxites XI short and blunt (Fig. 69C–F)	***Plegaspinosa* Ardila et al., 2019**
–	Area adjacent to frontal sutures slightly sunken (Fig. 26D); antennal scape elongate set with stout setae in the male (Fig. 26C, D); male genitalia with ventral process of the median lobe of the gonocoxites XI acuminate (Fig. 27F)	**14**
14	Antennal scape 2× as long as wide at middle (Fig. 26D); hind wing without conspicuous apical stripe (Fig. 26B); male genitalia with ventral part of gonocoxites XI median lobe set with ventral triangular process and caudal transversal ridge (Fig. 27F); female gonocoxites + gonapophyses VIII with narrow and elongate medial process (Fig. 27J)	***Plegaacevedoi* Ardila-Camacho & Contreras-Ramos, sp. nov.**
–	Antennal scape 3.5× as long as wide at middle (Fig. 50D); hind wing with conspicuous apical stripe (Fig. 50B); male genitalia with ventral part of gonocoxites XI median lobe set with ventral elongate hook-shaped process and caudal, lateral fins (Fig. 51C–F); female gonocoxites + gonapophyses VIII with vase-shaped medial process (Fig. 51J)	***Plegalongicornis* Ardila-Camacho & Contreras-Ramos, sp. nov.**
15	Forefemur remarkably robust (Fig. 61F); male gonocoxite IX set with three apical digitiform processes, subequal in shape and size, situated on inner surface (Fig. 62C–E); bursa copulatrix entirely membranous (Fig. 62H	***Plegapseudohagenella* Ardila-Camacho & Contreras-Ramos, sp. nov.**
–	Forefemur not remarkably robust (Fig. 71F); male gonocoxite IX set with 4–12 processes; bursa copulatrix with proximal region sclerotized (Fig. 74I)	**16**
16	Head with vertexal region patterned with narrow pale and brown stripes (Fig. 71C); pronotum brown with pale medial areas (Fig. 71D); forefemur anterior surface brown with pale areas including the base (Fig. 71E); male gonocoxite IX with 10–12 subequal processes arranged in a bundle (Fig. 72C–E)	***Plegastangei* Ardila et al. 2019**
–	Head with vertexal region with lateral broad brown stripes (Fig. 73C); pronotum mostly brown (Fig. 73D); forefemur anterior surface uniformly brown (Fig. 73E); male gonocoxite IX with 4–6 subequal processes on distal ⅓ arranged as a row of branches on the outer surface (Fig. 74C–E)	***Plegavangiersbergenae* Ardila-Camacho, sp. nov.**
17	Basal antennal flagellomeres as wide as long (Fig. 36C)	**18**
–	Basal antennal flagellomeres > 2× as wide as long (Fig. 42C)	**19**
18	Antennal flagellum entirely brown (Fig. 36C, D); supra-antennal area entirely brown (Fig. 36C); male gonocoxite IX with three digitiform, apical processes, whose surface has scattered, minute spinules (Fig. 37C–E); male sternite IX without short, posteromedial process (Fig. 37A); female gonocoxites VII as two lateral trapezoidal plates (Fig. 37G)	***Plegadrepanicoides* Ardila-Camacho & Contreras-Ramos, sp. nov.**
–	Antennal flagellum with 3–5 pale preapical flagellomeres (Fig. 75C); supraantennal area with a U-shaped pale area (Fig. 75C); male gonocoxite IX with two digitiform, smooth processes, of which one is slightly longer (a third smaller process is sometimes present) (Fig. 76C–E); male sternite IX with short, posteromedial process (Fig. 76A); female gonocoxites VII unpaired, sternite-like (Fig. 76G)	***Plegayucatanae* Parker & Stange, 1965**
19	Forecoxa with fine and long setae on anterior and posterior surfaces not arising from protuberant bases; forefemur noticeably robust (Fig. 42F)	**20**
–	Forecoxa with thickened setae arising from protuberant bases; forefemur not noticeably robust (Fig. 52F)	**21**
20	Body mostly dark reddish brown (Fig. 42A); forefemur anterior surface uniformly dark-reddish brown colored (Fig. 42E); male gonocoxite IX set with 7–9 long thin processes arranged as a paint-brush (Fig. 43C–E)	***Plegafumosa* Linsley & MacSwain, 1955**
–	Body with a mixture of dark brown and yellow (Fig. 54A); forefemur anterior surface dark brown with base yellow (Fig. 54A); male gonocoxite set with 8–18, short digitiform processes arranged as a paint-brush (Fig. 55C–E)	***Plegamixteca* Ardila et al. 2019**
21	Anteroventral row of integumentary specializations lacking curved, basal, primary process (Fig. 46E); postfurcasternum paired, plate-shaped	***Plegainsolita* Ardila-Camacho & Contreras-Ramos, sp. nov.**
–	Anteroventral row of integumentary specializations with curved, basal, primary process (Fig. 32E); postfurcasternum unpaired, collar-like	**22**
22	Basal antennal flagellomeres > 3× as wide as long (i.e., discoidal) (Fig. 28D)	**23**
–	Basal antennal flagellomeres 2× as wide as long (Fig. 65C)	**24**
23	Body color pattern pale and dark reddish brown (Fig. 28A); mesonotum often with a wide, pale, medial band; sternite IX posteromedially elongated, surpassing posterior the margin of ectoproct (Fig. 29A); male gonocoxite IX equipped with 4–7 processes of different lengths arranged as a paint-brush (Fig. 29C–E); female gonapophyses VIII with chamber-shaped medial part, posteromedially set with an elongated Y-shaped process (Fig. 29H)	***Plegabanksi* Rehn, 1939**
–	Body color pattern pale and dark brown (Fig. 32A); mesonotum nearly completely dark brown; male sternite IX posteromedially short, not surpassing the posterior margin of ectoproct (Fig. 33A); male gonocoxite IX equipped with 4–15 digitiform processes twisted (Fig. 33C–E); female gonapophyses VII medial part keel-shaped, with short posteromedial, bilobed process (Fig. 33H)	***Plegadactylota* Rehn, 1939**
24	Region of the vertex often with a well-defined, arrow-shaped, pale area surrounding the coronal suture (Fig. 52C); pronotum as long as wide and mostly dark brown (Fig. 67D); male sternite IX posteromedially elongated (Fig. 68B)	**25**
–	Region of the vertex with pale area surrounding the coronal suture not arrow-shaped (Fig. 65C); pronotum slightly longer than wide and with conspicuous pale areas and stripes (Fig. 65D); male sternite IX posteromedially barely emarginated (Fig. 66B)	**26**
25	Forewing distinctively enlarged (Fig. 52A); male gonocoxite IX long and thin, equipped with 4–6 processes of different sizes, arranged as a hand (Fig. 53C–E)	***Plegamegaptera* Ardila-Camacho & Contreras-Ramos, sp. nov.**
–	Forewing not distinctively enlarged (Fig. 67A); male gonocoxite IX short and thickened, equipped with two or three processes, of which one is apical and longer and 1 or 2 are preapical and shorter (Fig. 68C–E)	***Plegasonorae* Ardila et al., 2019**
26	Forefemur anterior surface completely dark brown (Fig. 65E); male gonocoxite IX equipped with 3–5 elongated, intertwined processes (Fig. 66C–E)	***Plegasignata* (Hagen, 1877)**
–	Forefemur anterior surface brown with pale base (Fig. 40E); male gonocoxite IX posteriorly, laterally curved, and set with 8–13 processes arranged in a bunch (Fig. 41C–E)	***Plegaflammata* Ardila-Camacho & Contreras-Ramos, sp. nov.**

#### ﻿﻿ Plega acevedoi

Taxon classificationAnimaliaNeuropteraRhachiberothidae

Ardila-Camacho & Contreras-Ramos sp. nov.

https://zoobank.org/D53693B5-4C13-4C97-A8A7-04637B42AA76

Figs 26 , 27

##### Type locality.

Mexico, **Morelos**: Balneareo de los Manantiales, 18°28.059'N, 99°9.315'W, 2253 m, 20.IX.2018, C. Rodríguez Leg., selva baja caducifolia.

##### Material examined.

***Holotype*** male, pinned. Original label: “Mexico, **Morelos**, Balneareo de los Manantiales, 18°28.059'N, 99°9.315'W, 2253 m, 20 Sep. 2018, C. Rodríguez Leg., selva baja caducifolia; CNIN. ***Paratypes*.** Mexico • 1 ♂; **Morelos**, CEAMISH, Sierra de Huautla; 14 Apr. 1996; S. Zaragoza leg.; CNIN. • 1 ♀; Tlaquiltenango, Huautla; 18°23'04.2"N, 99°03'00.03"W; 1028 m; 25 Nov. 2008; V.H. Toledo; selva baja caducifolia; light trap; CNIN-SHM120. • 1 ♀; Tlaquiltenango, Huaxtla; 18°23'4.272"N, 99°3'0.036"W; 1028 m; 25 Nov. 2008; V.H. Toledo leg.; selva baja caducifolia; light trap; CNIN- SHM174. • 1 ♀; Tlaquiltenango, Huaxtla; 18°23'4.272"N, 99°3'0.036"W; 1028 m; 23 Mar. 2009; V.H. Toledo leg.; selva baja caducifolia; light trap; CNIN- SHM111.

##### Other material.

Mexico – **Morelos** • 1 ♂; Tlaquiltenango, Huautla; 18°23'08.4"N, 99°03'04"W, 1023 m, 28–31 Jan. 2009; V.H. Toledo leg.; selva baja caducifolia; light trap; CNIN-SHM227. • 1 ♂; same data as for preceding; CNIN-SHM229. • 1 ♂; same data as for preceding; CNIN- SHM268. • 1 ♂; same data as for preceding; CNIN-SHM261. • 1 ♂; same data as for preceding; CNIN- SHM262. • 1 ♂; same data as for preceding; CNIN- SHM246. • 1 ♂; same data as for preceding; CNIN- SHM249. 1 ♂; same data as for preceding; CNIN-SHM257. • 1 ♂; same data as for preceding; CNIN- SHM261. • 1 ♂; same data as for preceding; CNIN- SHM253. • 1 ♂; same data as for preceding; CNIN- SHM278. • 1 ♂; same data as for preceding; CNIN- SHM251. • 1 ♂; same data as for preceding; CNIN- SHM195. • 1 ♂; same data as for preceding; CNIN-SHM266. • 1 ♂; same data as for preceding; CNIN-SHM242. • 1 ♂; same data as for preceding; CNIN-SHM193. • 1 ♂; same data as for preceding; CNIN-SHM228. • 1 ♂; same data as for preceding; CNIN-SHM232. • 1 ♂; same data as for preceding; CNIN-SHM241. • 1 ♂; Tlaquiltenango, Huautla, 18°23'04.27"N, 99°03'00.03"W; 1028 m; 25 Nov. 2008; V.H. Toledo leg.; selva baja caducifolia; light trap; CNIN-SHM197. • 1 ♂; same data as for preceding; CNIN-SHM208. • 1 ♂; same data as for preceding; CNIN- SHM198. • 1 ♂; same data as for preceding; CNIN-SHM204. • 1 ♂; same data as for preceding; CNIN- SHM265. • 1 ♂; same data as for preceding; CNIN-SHM219. • 1 ♂; same data as for preceding; CNIN-SHM206. • 1 ♂; same data as for preceding; CNIN- SHM272. • 1 ♂; same data as for preceding; CNIN-SHM203. • 1 ♂; same data as for preceding; CNIN-SHM244. • 1 ♂; same data as for preceding CNIN-SHM244. • 1 ♂; same data as for preceding; CNIN-SHM191. • 1 ♂; same data as for preceding; CNIN-SHM269. • 1 ♂; same data as for preceding; CNIN-SHM277. • 1 ♂; same data as for preceding; CNIN-SHM207. • 1 ♂; same data as for preceding; CNIN-SHM209. • 1 ♂; same data as for preceding; CNIN-SHM202. • 1 ♂; same data as for preceding; CNIN-SHM234. • 1 ♂; same data as for preceding; CNIN-SHM210. • 1 ♂; same data as for preceding; CNIN-SHM201. • 1 ♂ same data as for preceding; (CNIN-SHM279). • 1 ♂; same data as for preceding; CNIN-SHM223. • 1 ♂; same data as for preceding; CNIN- SHM199. • 1 ♂; Tlaquiltenango, Huaxtla; 18°23'8.412"N, 99°3'4.068"W; 1023 m; 28–31 Jan. 2009; V.H. Toledo leg.; CNIN-SHM285. • 1 ♂; Tlaquiltenango Huaxtla; 18°23'4.272"N, 99°3'0.036"W; 1028 m; 23 Mar. 2009; V.H. Toledo leg.; selva baja caducifolia; light trap; CNIN-SHM167. • 1 ♀; same data as for preceding; CNIN-SHM177. • 1 ♂; Tlaquiltenango, E. Huaxtla; 18°22'50.556"N, 99°2'39.624"W; 1030 m; 29 Mar. 2009; V.H. Toledo leg.; selva baja caducifolia; light trap; CNIN. • 1 ♀; Tlaquiltenango, Huaxtla; 18°23'4.272"N, 99°3'0.036"W; 1028 m; 25 Nov. 2008; V.H. Toledo leg.; selva baja caducifolia; light trap; CNIN-SHM115. • 1 ♀; same data as for preceding; CNIN- SHM142. • 1 ♀; same data as for preceding; CNIN-SHM144. • 1 ♀; same data as for preceding; CNIN-SHM150. • 1 ♀; same data as for preceding; CNIN-SHM119. • 1 ♀ same data as for preceding; CNIN-SHM152. • 1 ♀; same data as for preceding; CNIN-SHM156. • 1 ♀; same data as for preceding; CNIN-SHM189. • 1 ♀; same data as for preceding; CNIN-SHM166. • 1 ♀; same data as for preceding; CNIN-SHM154. • 1 ♀; same data as for preceding; CNIN- SHM181. • 1 ♀; Tlaquiltenango, Huaxtla; 18°23'8.412"N, 99°3'4.068"W; 1023 m; 28–31 Jan. 2009; V.H. Toledo leg.; selva baja caducifolia; light trap; CNIN-SHM134. • 1 ♀; same data as for preceding; CNIN- SHM176. • 1 ♀; Tlaquiltenango, Coaxitlán; 18°27'29.772"N, 99°13'13.872"W; 842 m; 29 Mar. 2011; V.H. Toledo; selva baja caducifolia; light trap; CNIN-SHM185. • 1 ♀; Tlaquiltenango, 2 Km NW Huixastla; 18°28'52.428"N, 99°8'37.032"W; 767 m; 12 Feb. 2010; N. Hernández leg.; selva baja caducifolia; light trap; CNIN-SHM121. • 1 ♀; same data as for preceding; CNIN-SHM127. • 1 ♀; Tlaquiltenango, Huautla; 18°23'04.2"N, 99°03'00.03"W; 1028 m; 25 Nov. 2008; V.H. Toledo leg.; selva baja caducifolia; light trap; CNIN-SHM168. • 1 ♀; same data as for preceding; CNIN-SHM145. • 1 ♀; same data as for preceding; 18°23'08.4"N, 99°03'04"W; 1023 m; 28–31 Jan. 2009; CNIN- SHM147. • 1 ♀; Tepalcingo, S. El Limón; 18°31'42.6"N, 98°56'24.6"W; 1244 m; 09 Feb. 2013; I.A. Villanueva, V.H. Toledo, R. Reyes, J.G. Martínez leg.; selva baja caducifolia; light trap; CNIN-SHM164. • 1 ♀; Puente de Ixtla, Sur de Coaxitlán, camino antiguo a Quetzalapa; 18°25'46.992"N, 99°10'45.408"W; 1050 m; 03 Mar. 2011; V. Toledo, J.G. Martínez, V. Rendón, F. Hinterholzen leg.; selva baja caducifolia; light trap; CNIN-SHM284. • 1 ♀; same data as for preceding; CNIN- SHM109. • 1 ♀; same data as for preceding; CNIN-SHM108. • 1 ♀; same data as for preceding; CNIN-SHM110. • 1 ♀; same data as for preceding; CNIN-SHM122. • 1 ♀; same data as for preceding; CNIN-SHM132. • 1 ♀; same data as for preceding; CNIN-SHM118. • 1 ♀; Tlaquiltenango, Coaxitlán; 18°25'46.992"N, 99°10'45.408"W; 876 m; 03 Mar. 2011; V.H. Toledo, J.G. Martínez, V. Rendón, F. Hinterholsen leg.; selva baja caducifolia; light trap; CNIN-SHM137. • 1 ♀; Tlaquiltenango, 2 Km N Huaxtla; 18°23'27.024"N, 99°2'54.096"W; 1142 m; 13 Nov. 2009; Campos, Reza & Martínez leg.; selva baja caducifolia; light trap; CNIN-SHM126. • 1 ♀; Tlaquiltenango, 5 Km NW de Huaxtla; 18°24'25.524"N, 99°3'14.544"W; 1246 m; 15 Nov. 2009; E.M. Reza, J.G. Martínez, N. Campos, Y. Viveros leg.; selva baja caducifolia; light trap; CNIN-SHM146. • 1 ♂; Tlaquiltenango, 2.5 Km W Huautla, Estación CEAMISH; 18°27'49.28"N, 99°02'15.05"W; 940 m; 15 Feb. 1996; S. Zaragoza, F. Noguera, E. Ramírez & E. Gonzáles leg.; bosque tropical caducifolio; light trap; CNIN. • 1 ♀; Tlaquiltenango, 2.5 Km W Huautla, Estación CEAMISH; 18°27'47.51"N, 99°02'07.90"W; 940 m; 15 Jan. 1996; S. Zaragoza, F. Noguera, E. Ramírez & E. Gonzáles leg.; bosque tropical caducifolio; light trap; CNIN-MDM0123. • 1 ♂; Villa de Ayala, Rancho El Polvorín; 29 Jan. 1971; H. Pérez R. leg.; nocturnal collection; CNIN-N° Cat 86629. • 1 ♂; same data as for preceding; 22 Jul. 1971; CNIN. • 1 ♂; same data as for preceding; 22 Jul. 1971; CNIN. • 1 ♂; same data as for preceding; 17 Oct. 1971; CNIN. • 1 ♂; Villa de Ayala, Polvorín; 02 Oct. 1972; H. Pérez leg.; CNIN. • 1 ♂; Villa de Ayala, Rancho el Polvorín; 1972; H. Pérez R. leg.; nocturnal collection (23:00 h–01:00 h); CNIN. • 1 ♂; same data as for preceding; CNIN. • 1 ♂; Villa de Ayala, Rancho el Polvorín; H. Pérez R. leg.; nocturnal collection (23:00 h–01:00 h); CNIN) • 1 ♀; Villa de Ayala, Rancho El Polvorín, 22 Jul. 1971, H. Pérez leg.; nocturnal collection; CNIN. • 3 ♀; same data as for preceding; 19 Sep. 1971; CNIN. • 2 ♀; same data as for preceding; 1972; 23:00 h–1:00 h; CNIN. • 1 ♀; same data as for preceding; 22 Jul. 1971; CNIN. • 1 ♀; same data as for preceding; 17 Oct. 1971; CNIN. • 2 ♀; Villa de Ayala, Rancho El Polvorín; H. Pérez leg.; nocturnal collection; CNIN. • 1 ♀; same data as for preceding; 20 Jan.1972; nocturnal collection19:00 h–21:00 h; CNIN. • 2 ♀; same data as for preceding; 06 Nov. 1972; CNIN. • 1 ♂; Xochicalco pyramid; 30 Mar. 1962; F.D. Parker & L.A. Stange leg.; FSCA. – **Puebla** • 1 ♂; Jolalpan, Rancho El Salado, ladera W del cerro Colorado; 18°20'11.61"N, 98°58'55.38"W; 1022 m; 30 Jan. 2014; V.H. Toledo, F. Hinterholzer, J.G. Martínez leg.; selva baja caducifolia; CNIN-SHM226. • 1 ♂; same data as for preceding; CNIN-SHM280. • 1 ♂; same data as for preceding; CNIN-SHM138. • 1 ♂; Jolalpan, Rancho El Salado; 18°20'49.38"N, 98°59'23.02"W; 923 m; 06 Oct. 2010; V.H. Toledo, F. Hinterholzer, J.G. Martínez leg.; selva baja caducifolia; light trap; CNIN- SHM240. • 1 ♂; same data as for preceding; CNIN-SHM238. • 1 ♂; same data as for preceding; CNIN-SHM200. • 1 ♂; same data as for preceding; CNIN-SHM222. • 1 ♂; same data as for preceding; CNIN-SHM180. 1 ♀; Jolalpan, Rancho El Salado, Ladera W de Cerro Colorado; 18°20'11.616"N, 98°58'55.2"W; 1022 m; 05 Oct. 2010; V.H. Toledo, F. Hinterholzer, J.G. Martínez leg.; selva baja caducifolia; light trap; CNIN-SHM170. – **Veracruz** • 1 ♂; E. Biología Los Tuxtlas; 20 May. 1971; *Symphrasis* sp., det. Flint, 1978; CNIN-81206.

##### Diagnosis.

This species is closely related to *P.longicornis* and *P.spinosa* as the area adjacent to frontal sutures is sunken, and the supra-antennal is raised laterally and set with abundant, fine, reclined setae. *Plegaacevedoi* can be separated from them by the antennal scape 2× as long as wide at middle with dorsal surface set with stout setae near the distal margin in males. This species exhibits the femoral posterior surface pale with brown dots, while the anterior surface is dark brown, except basal ¼ pale. On the male genitalia, the gonocoxites IX lack digitiform processes as in *P.longicornis*, *P.oswaldi*, and *P.spinosa*, and the ectoproct is equipped with spine-shaped setal bases on the posteroventral surface; the gonostyli X are posteriorly recurved at mid length before protruding from abdomen, with an abruptly narrowed and pointed apex. The ventral part of the gonocoxites XI medial lobe has a prominent, rounded, transversal ridge and ventrally it forms a pointed, curved process. Moreover, in the female genitalia, the gonocoxites + gonapophyses VIII form two lateral quadrangular plates with a tubular, medial process whose apex is shallowly bifid; the spermatheca is short and simple, with undifferentiated proximal and medial sections; the proximal-most part and the apex form a blunt diverticulum each.

##### Etymology.

This species is dedicated to Fernando Acevedo Ramos, Spanish entomologist and expert in Myrmeleontidae, who supported the first author in many ways. This allowed the development of much of this revision during its first stages.

##### Description.

***Measurements*.** Male (*n* = 6). Forewing length: 6.6–14.9 mm; Hind wing length: 5.3–12.0 mm. Female (*n* = 5). Forewing length: 11.2–14.3 mm; Hind wing length: 9.4–11.4 mm.

**Figure 26.**
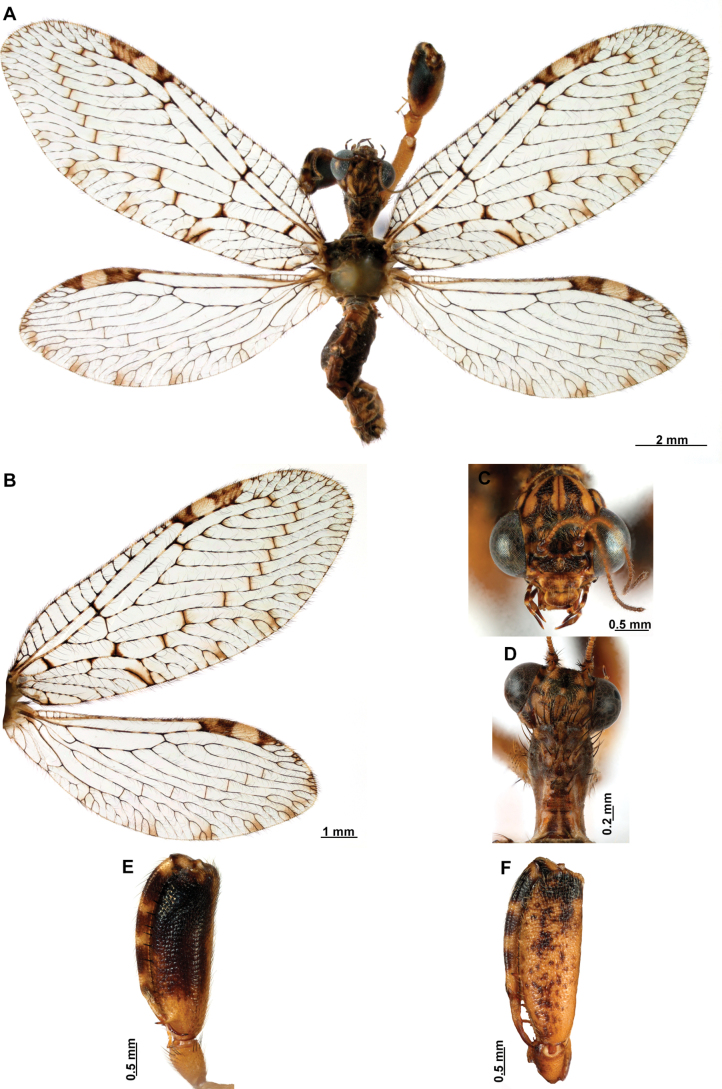
*Plegaacevedoi* Ardila-Camacho & Contreras-Ramos, sp. nov. **A** male habitus, dorsal **B** wings **C** head, frontal **D** pronotum, dorsal **E** forefemur, anterior surface **F** same, posterior surface.

***Coloration*** (Fig. 26). ***Head*.** Vertexal region pale, with lateral brown marks extending from occipital ridge to supra-antennal area, embracing pale areas and forming an inverted V-shaped pattern, with pale brown setae; area adjacent to occipital ridge brown; supra-antennal area brown with pale lateral areas, with pale brown setae; occiput and postgena pale with brown stripes. Antennal scape pale with longitudinal brown stripes, apex of dorsal surface with dark brown setae; pedicel brown; flagellum pale brown changing to brown towards apex, with 1–4 pale, preapical flagellomeres, sometimes completely brown. Frons pale with brown with pale medial area. Clypeus and labrum pale with pale brown areas; mandible pale with dark brown corners, apex dark amber; maxilla pale with brown areas, palpus mostly dark brown; labium pale with brown areas, palpus mostly dark brown, palpimacula pale brown. ***Thorax*.** Pronotum mostly brown, with pale areas on posterior region; episternum brown; postfurcasternum brown with pale areas. Mesonotum dark brown, sometimes with anterolateral pale areas; metanotum brown with cream anteromedial, small area; pre-episternum brown; pteropleura with brown marks and pale areas, pale brown setae present. ***Foreleg*.** Coxa pale to pale brown, with brown mark at apex of anterior surface, with some dark brown setae at basal region; trochanter with same color as coxa, dorsally with some dark brown setae. Femur posterior surface pale with brown dots; anterior surface dark brown, except basal ¼ pale. Tibia dashed with pale and brown. Basitarsus pale at basal ½, changing to amber towards the apex; remaining tarsomeres pale brown. ***Mid- and hind leg*.** Coxa and trochanter pale with brown areas; femora and tibiae pale with brown rings; tarsi pale, distal margins of eutarsal tarsomeres with dark brown setae on plantar surface; remaining surface with pale brown setae. ***Wings*.** Forewing mostly hyaline; membrane surrounding forks of longitudinal veins, crossveins, first branch of CuA and apex of CuP amber; posterior and apical margins with intermittent, amber areas between apical branches of longitudinal veins; pterostigma brown with small, anterior pale areas, and larger cream medial area; major veins, subcostal veinlets, and wing margin alternating pale and brown; crossveins dark brown. Hind wing hyaline, adjacent area to posterior and apical margins with intermittent amber areas between apical branches of longitudinal veins; pterostigma brown with small pale areas, and wide, cream, preapical region; longitudinal veins alternating pale and brown; crossveins brown, except sigmoid 1r-m bicolor; wing margin alternating pale and brown. ***Abdomen*.** Tergites brown. Pleural membrane dark brown, except near dorsal and ventral margins pale. Sternites pale with lateral, brown stripe on each side.

***Morphology*** (Fig. 26). ***Head*.** Diamond-shaped in frontal view, rugose; vertexal region raised above compound eyes, with lateral rows of reclined setae; area surrounding coronal suture glabrous, with muscle insertion mark; coronal suture distinct, area adjacent to frontal sutures sunken; ​​supra-antennal raised laterally, with abundant, fine, reclined setae; paraocular area concave. Antenna filiform, short; scape two times as long as wide at middle, dorsal surface with stout setae near distal margin; pedicel slightly longer than wide; flagellum not dorsoventrally flattened, with 28–33 flagellomeres, those of flagellar base as wide as long, then changing to slightly longer than wide on the rest of flagellum; all articles with medial ring of fine, short setae. Compound eye hemispherical, as wide as ½ of the interocular distance at torulus level. Frons and clypeus narrow. Labrum pentagonal with thin, short setae; maxillary palpus with first palpomere as long as wide, second 1.2× as long as wide, third palpomere 3.5× as long as wide, fourth palpomere 2.5× as long as wide, fifth palpomere slightly longer than third; mentum with long, thin setae; labial palpus with first palpomere 2× as long as wide, second palpomere four 4.5× as long as wide, third slightly shorter than second, palpimacula narrowly ovoid. ***Thorax*.** Pronotum slightly longer than wide, with raised anterior margin, medial and posterior regions; raised regions and anterior and margins covered with pedicellate, thickened setae. Mesonotum 2× as wide as long, with scattered, thick, pedicellate setae on medial area. Metanotum ~ 2× as long as wide, glabrous. Pteropleura with short, thin, setae a few thickened on mesanepisternum. ***Foreleg*.** Coxa nearly as long as femur, cylindrical, posterior surface with pedicellate, thick setae; trochanter trapezoidal, with thin and short setae, except on dorsal and anterior surfaces with some thickened, pedicellate setae near apex; anterior surface with protuberant area. Femur robust, covered with abundant, fine, short setae; closing surface with posteroventral row of processes composed two medially located, primary processes, with or without a tertiary process between them; proximal portion of the row with sub-basal secondary process and basal tertiary process; the rest of the row with numerous tubercle-shaped processes, and stinger-shaped setae; distal ½ slightly carinated, composed of tubercle-shaped specializations and stinger-shaped setae; adjacent row of thickened setae with globular base present on distal ¼. Anteroventral row of processes reduced to proximal ½; it is composed of tubercle-shaped specializations and stinger-shaped setae; the basal-most primary, curved process is present; distal portion composed of a few tubercle-shaped processes; adjacent row of thickened setae with globular base present on distal ½. Tibia almost as long as femur, curved, with thin, short setae; ventral surface keeled with prostrate setae; a patch of clavate setae apically on anterior surface is present. Basitarsus with lanceolate process reaching the middle of fourth tarsomere; clavate setae present proximally on anterior surface; ventrally with single row of prostrate setae; second tarsomere nearly 7× as long as wide; third tarsomere as long as wide, fourth tarsomere two times as long as wide. ***Mid- and hind leg*.** Coxa with short and thin setae; trochanter with short, thin setae; femora with thin setae; tibial spurs short; tibiae with short and thin setae. Hind leg longer than midleg; tibia, 1.5× as long as femur; tarsi with fine and short setae, except on distal margin of plantar surface with lateral groups of 3–5 thickened setae; on both legs, basitarsus 4× as long as wide, second tarsomere 1.2× as long as wide; third and fourth tarsomeres as long as wide; fifth tarsomere two times as long as wide. ***Wings*.** Forewing oval, trichosors present along margin except on wing base; venation setose; costal space proximally expanded, humeral vein branched, 12–19 subcostal veinlets; pterostigma trapezoidal, elongated, narrow, straight, with distinct veinlets; subcostal space with single crossvein, medially located; Sc vein abruptly posteriorly bent at proximal pterostigma margin to merge the RA; radial space with two crossveins; *rarp2* gently curved with 2–4 RP branches; 2–4 veins arising from *rarp1*; M vein basally fused to RA; RP base widely separated from divergence of M and R; M forked opposite to RP origin, 1 r-m connecting RP base and MA stem, forming a trapezoidal cell; 5–7 gradate crossveins present. Cubitus deeply forked; CuP basally angled and approaching A1, distally forked opposite to the level of separation of M and R; A1 apically forked, ending on posterior margin slightly beyond the level of CuP fork, A2 forked slightly beyond to CuP angle level. Hind wing smaller and narrower than forewing, oval; costal space narrow and reduced, with 5–7 veinlets; C and Sc fused at proximal ⅕ of wing length, Sc vein abruptly curved posteriad at proximal margin of pterostigma to merge RA; pterostigma elongated, narrow, straight, composed of well-defined veinlets; radial space with single crossvein, oblique; 2–4 veins arising from *rarp1*, two or three from *rarp2*; 1r-m sigmoid, connecting the stems of M and RP. Media forked slightly beyond R fork. Cubitus deeply forked, intracubital crossvein subparallel to longitudinal wing axis; CuA sinuous, first branch candelabrum-shaped, spur vein absent; CuP not touching A1, strongly anteriorly bent at distal 1/3, pectinate; two crossveins on cubitoanal space present; A1 simple, ending at wing margin at 1r-m stem level; A2 simple, short, and curved. ***Abdomen*.** Tergites quadrangular with anterolateral elongated scars; sternites rectangular.

**Figure 27.**
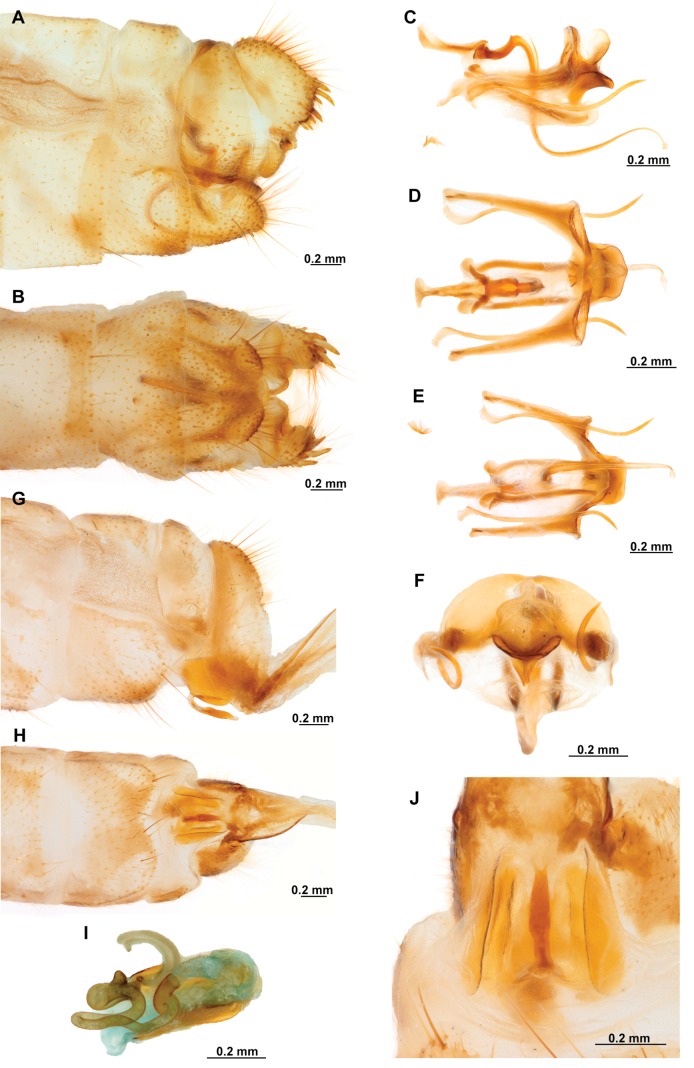
*Plegaacevedoi* Ardila-Camacho & Contreras-Ramos, sp. nov. **A** male terminalia, lateral **B** same, ventral **C** male genitalia, lateral **D** same, dorsal **E** same, ventral **F** same, caudal **G** female terminalia, lateral **H** same, ventral **I** spermatheca **J** gonapophyses VIII of female, ventral.

***Male genitalia*** (Fig. 27A–F). Tergite IX medially narrower than laterally; lateral margin blunt. Sternum VIII rectangular; sternite IX trapezoidal in ventral view; posterior margin with medial lobe not developed, but corresponding area dorsally canaliculated; in lateral view trapezoidal, not reaching posterior margin of ectoproct. Gonocoxites IX thin, sinusoid, short; base spatulated; apex pointed, without processes. Ectoproct ovoid, posteroventral surface set with spine-shaped setal bases, remaining surface with long, pedicellate setae; anteroventrally with enlarged, rounded, sclerotized lobe, continuous with ventromedial sclerotized, curved sulcus. Gonocoxites X forming a short, straight, ventrally canaliculate sclerite; anterior apex, expanded; posterior apex with dorsal processes connected to gonostyli X and ventrolateral processes connected to gonapophyses X with a membrane; gonostyli X with thickened and concave base with two lateral processes, the rest of the structure, ventrally strongly curved, and posteriorly recurved at mid length before protruding from abdomen, apex abruptly narrowed and pointed. Gonapophyses X rod-shaped, gently curved; gonapophyses sub-parallel, joined by a membrane covering the gonostyli X base. Gonocoxites XI U-shaped, medial lobe complex and elaborated with two differentiated parts: dorsal part as an arch, with lateral parts expanded; ventral part with prominent, rounded, transversal ridge; ventrally a pointed, curved process is present; between these parts an irregular, less sclerotized, hyaline area is present, which forms a triangular outgrowth; lateral arms of gonocoxites XI short, thin, sigmoid, with anterior apex bent ventrad. Hypandrium internum triangular, with lateral fins.

***Female genitalia*** (Fig. 27G–J). Sternum VII rectangular with posteromedian concavity. Tergite VIII narrower medially than laterally, encircling the spiracle of the segment; lateral margin quadrangular. Gonocoxites VIII not discernible, forming a complex with the gonocoxites VIII. Gonocoxites + Gonapophyses VIII as two lateral quadrangular plates which form a tubular, medial process whose apex is shallowly bifid; lateral part of gonapophyses VIII as a rounded plate folded beneath tergite IX + ectoproct. Tergite IX + ectoproct triangular. Gonocoxites IX elongated and narrow, nearly as long as the last five abdominal segments; gonapophyses IX as tiny sclerites at the inner base of gonocoxites IX. Bursa copulatrix short, funnel-shaped, with proximal-most part lightly sclerotized, the rest membranous and striated. Spermatheca short and simple; proximal and medial sections undifferentiated, thin; proximal-most forming a blunt diverticulum; distal section progressively slightly expanded, forming a blunt diverticulum; fertilization canal duct with proximal portion triangular, concave; fertilization canal elongated a narrow, C-shaped, covered with microfilaments.

##### Distribution.

Mexico (Morelos, Puebla, Veracruz).

##### Remarks.

This species is closely related to *P.spinosa* and *P.longicornis* based on the sexual dimorphism of the antennae, morphology of the supra-antennal area, coloration of the forefemur, the overall morphology of the male terminalia and genitalia, plus the female genitalia. Furthermore, its distribution in central Mexico (Morelos, Puebla and Veracruz) supports its phylogenetic affinities. This new species, together the aforementioned ones form a complex of species together *P.oswaldi* and *P.obtusa*.

#### ﻿﻿ Plega banksi

Taxon classificationAnimaliaNeuropteraRhachiberothidae

Rehn, 1939

Figs 28 , 29

Plega banksi Rehn, 1939: 248. Holotype: male, USA, Arizona (AMNH).

##### Material examined.

***Paratypes*.** USA • 1 ♂; **Arizona**, Oracle 14 m E.; 27 Jul. 1924; E.P.V. Van Duzee leg.; “*Plegabanksi* Rehn, Paratype”; CAS. • 1 ♀; Baboquivari Mts.; 15 Aug. 1922; O.C. Poling leg.; “Pres. By C.L. Fox”; “*Plegabanksi* Rehn, Paratype”; CAS. • 1 ♂; Boya Thomson, Aboretum, Superior; 1–3 Aug. 1937; MCZ-Paratype 25378.

##### Other material.

Mexico – **Sinaloa** • 2 ♀; 16 mi S Guamuchil; 20 May. 1962; F.D. Parker & L.A. Stange leg.; *Plegafratercula* Rehn det. L. Stange, 1991; FSCA. • 1 ♀; Sinaloa, Guamuchil; 01 Jul. 1963; F.D. Parker & L.A. Stange leg.; FSCA. • 1 ♂ 1 ♀; Sinaloa, 16 miles SO. Guamuchil; 16 Jun. 1961; FSCA. • 1 ♂; Sinaloa, Rio Piaxtla, 1 mi. W. Mex No. 15; E.L. Sleeper & R.C. Anderson leg.; *Plegadactylota*, det. Penny, 1998; CAS. • 1 ♂; Sinaloa, 16 miles SO. Guamuchil; 16 Jun. 1961; F.D. Parker & L.A. Stange leg.; FSCA. – **Sonora** • 1 ♀; 40 mi N. Guaymas; 29 Aug. 1959; L.A. Stange & A.S. Menke leg.; FSCA. • 1 ♀; 100 yds ½ mi E of San Carlos; 05 Apr. 2003; on *Bebbia*, REW; UCD. • 1 ♂; Punta La Manga; 27°58'46"N, 111°07'58"W; 5 m; 1–2 Aug. 2009; E.M. Fisher leg.; MT; CSCA.

USA – **Arizona** • 1 ♂; Madera Canyon; 24 Jul. 1976; B.K. Dozier leg.; FSCA. • 1 ♂; Sta. Catalina Mts.; 01 Jul. 1938; Bryant leg.; FSCA. • 1 ♂; Santa Cruz Co., Peña Blanca, Pajarito Mts.; 07 Jul. 1962; R.H. Arnett Jr., E.R. VanTassell leg.; FSCA. • 1 ♂; Santa Cruz Co., Madera Cyn.; 4880 ft.; 21 Aug. 1963; V.L. Vesterby leg.; UCD. • 1 ♂; same data as for preceding; 15 Aug. 1963; UCD. • 2 ♂; same data as for preceding; 14 Aug. 1963; UCD. • 2 ♂; same data as for preceding; 23 Jul. 1963; UCD. • 2 ♂; same data as for preceding; 15 Aug. 1963; UCD. • 1 ♂; same data as for preceding; 01 Sep. 1963; UCD. • 2 ♂; same data as for preceding; 21 Jul. 1963; UCD. • 1 ♂; same data as for preceding; 12 Sep. 1963; UCD. • 1 ♂; same data as for preceding; 21 Sep. 1963; UCD. • 1 ♂; same data as for preceding; 12 Aug. 1963; UCD. • 7 ♂; same data as for preceding; 21 Jul. 1963; UCD. • 1 ♂; same data as for preceding; 23 Jul. 1963; UCD. • 6♂ 6♀; Tucson, Pima Co.; 02 Aug. 1991; R. & J. Robertson leg.; CAS. • 1 ♂; Pima Co., Bavoquivari Mts., Sabino Can.; 07 Aug. 1951; L.M. Martin leg.; “Phillip Adams Collection 1998 request to Calif. Acad. Sci.”, *Plegabanksi* Rehn”; CAS. • 1 ♀; Globe; 17 Jul. 1933; Parker leg.; *Plegafratercula* Rehn, det. J.W.H. Rehn, A.N.S.P., 1939; CAS-114. • 1 ♀; Pima Co., Alamo Canyon, Ajo Mts.; Jul. 1924; H.B. Leech & J.W. Green leg.; *Plegabanksi*, det. N. Penny, 1985; CAS. • 1 ♂; Pima Co., Sta. Rita Mts., mouth Madera Canyon Procter Ranch Road; 4400’; 15 May. 1966; R.G. Beard leg.; U.V. light; “Phillip Adams Collection 1998 request to Calif. Acad. Sci.”, *Plegabanksi* Rehn, 1939, det. R.G. Beard; CAS. 1 ♂; same data as for preceding; “LDL#0562”; CAS. • 1 ♀; Pima Co., Sta. Rita Mts., mouth Madera Canyon Procter Ranch Road; 17 Jul. 1966; 4400’; R.G. Beard; U.V. light; MCZ-ENT00681784. • 1 ♂; same data as for preceding; 15 Jul. 1966; *Plegabanksi* Rehn, 1939, det. R.G. Beard; MCZ-ENT00681783. • 14 ♂ 12 ♀; Pima Co., Santa Rita Mts Experimental Range, Florida Cyn.; 31°45.42'N, 110°50.43'W; 1646 m; 24 Apr.–03 Jul. 2014; M.I. Erwin, C. O’ Brien, M.J. Sharkey leg.; Malaise on hillside trail; CSCA. • 1 ♂ 2 ♀; same data as for preceding; 3–10 Jul. 2014; CSCA. • 1 ♂; Pima Co., Sabino Cyn.; 04 Sep. 1961; J.S. Buckett leg., UCD. • 1 ♂; same data as for preceding; 04 Sep. 1961; UCD. • 1 ♂; same data as for preceding; 01 Sep. 1963; V.L. Vesterby; UCD. • 3 ♂; same data as for preceding; 19 Aug. 1963; UCD. • 1 ♂; same data as for preceding; 5800’; 11 Jul. 1967; R.C. Gardner, C.R. Kovacic, K. Lorenzen leg.; UCD. • 2 ♂ 1 ♀; Pima Co., Vail, Mountain Creek Ranch; 32°04.99'N, 110°39.56'W; 1100 m; 06–24 Aug. 2014; M.E. Irwin, M.J. Sharkey leg.; Malaise in small dry wash; CSCA. • 1 ♂; Maricopa Co., Sunflower, Jct. Hwy, 87 & Sycamore Ck.; 33°52'8.616"N, 111°27'51.732"W; ± 100 m; 744 m; 15 Aug. 2012; Oswald, Diehl, Machado leg.; MV light; TAMUIC-TAMU-ENTO X0966102. • 1 ♂; Graham Co., Mt Graham, SE side, 3.5 Km W Jet. 191 on hwy 266; 32°34.32'N, 109°43.11'W; 1265 m; 08–15 May. 2015; Malaise in sandy wash; CSCA. • 1 ♂ 1 ♀; Santa Cruz Co., 10 mi NW Nogales; 04 Sep. 1968; T.R. Haig leg.; ex-fluorescent black light; CSCA. • 1 ♂; Santa Cruz Co., Kino Springs; 20 May. 2002; K.A. & E.E. Williams; CSCA.

##### Diagnosis.

Distinguished from other species in the genus by the dark-reddish brown body coloration pattern. The vertexal region has a pale arrow-shaped mark around the coronal suture. The basal antennal flagellomeres are discoidal. The mesonotum often has a wide, pale, medial band. The forewing generally exhibits a zigzagged amber mark connecting MA and MP forks, terminal part of MA and 2r-m and 1ma-mp. The area between CuP and posterior wing margin is often amber. The sternite IX is pentagonal, it is posteromedially elongated, blunt, surpassing posterior margin of ectoproct. The gonocoxite IX is short, thickened, and sinusoid, with posterior apex laterally curved, and equipped with 4–7 processes of different lengths. The dorsal part of the median lobe of gonocoxites XI is rounded and anteriorly curved lobe; the ventral part is convex, and continuous with a ventral, curved, prominent hook-shaped process whose apex is covered with microspinulae. The gonapophyses VIII of female have a chamber-shaped medial part, posteromedially is set with an elongated Y-shaped process. The female gonocoxites IX are nearly as long as the whole abdomen. The bursa copulatrix is short, with the proximal part strongly expanded, ovoid, and sclerotized, continuous with a membranous and striated part, which is abruptly narrowed.

##### Description.

***Measurements*.** Male (*n* = 10). Forewing length: 7.8–14.6 mm; Hind wing length: 6.0–11.5 mm. Female (*n* = 7): Forewing length: 7.3–14.5 mm; Hind wing length: 5.6–11.7 mm.

**Figure 28.**
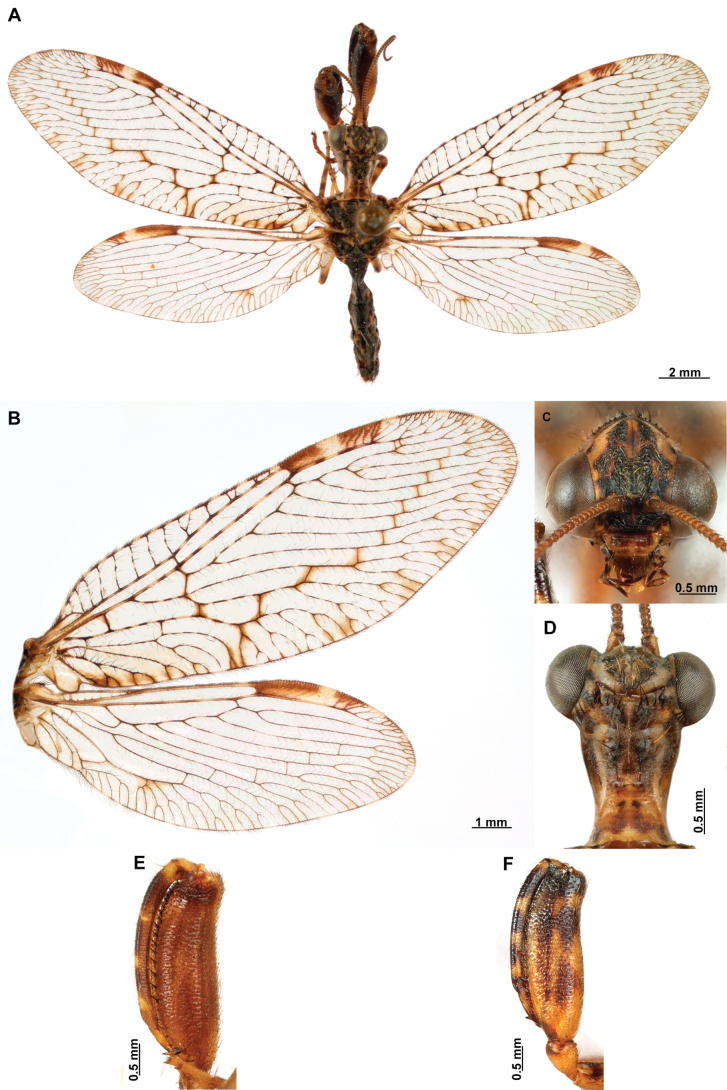
*Plegabanksi* Rehn, 1939 **A** male habitus, dorsal **B** wings **C** head, frontal **D** pronotum, dorsal **E** forefemur, anterior surface **F** same, posterior surface.

***Coloration*** (Fig. 28). ***Head*.** Vertexal mostly dark reddish brown with, small, lateral pale areas; area adjacent to occipital ridge pale; area adjacent to coronal suture with paler, arrow-shaped mark; supra-antennal area dark reddish brown; occiput pale with dark brown mark, postgena pale with brown area. Antennal scape brown with pale, longitudinal band on outer surface, pedicel dark brown; flagellum brown. Frons dark reddish brown, sometimes with medial pale stripe. Clypeus pale with lateral brown areas. Labrum pale brown; mandible yellowish with dark brown corners, dark amber at apex; maxilla pale with brown areas, palpus brown, with apex of the last three palpomeres yellowish; labium pale with dark brown postmentum, palpus with first two palpomeres dark brown, the last pale brown; palpimacula pale brown. ***Thorax*.** Pronotum pale with medial band and posterolateral dark reddish brown areas; episternum dark reddish brown with yellowish, medial area; postfurcasternum pale. Mesonotum, either mostly dark reddish brown with area adjacent to sutures pale brown or dark reddish brown with wide, pale, medial band with a rhomboid dark mark on center. Metanotum dark brown, sometimes with pale anteromedian area; mesopre-episternum dark reddish brown; pteropleura dark reddish brown with pale areas. ***Foreleg*.** Coxa yellowish with reddish brown areas, with interspersed pale and dark brown setae with brown bases; trochanter pale to yellowish with ventral dark reddish brown mark, dorsally with some dark brown setae. Femur posterior surface pale, with widened dark reddish brown areas; anterior surface dark reddish brown, except small, basal, yellowish areas. Tibia posterior surface alternating dark and pale areas, anterior surface mostly dark reddish brown, with preapical yellowish area. Basitarsus with base and lanceolate process dark amber, plus a sub-basal yellowish area; remaining tarsomeres yellowish to pale brown. ***Mid- and hind leg*.** Coxae pale with reddish brown suffusions; trochanter pale with brown suffusions. Femora pale with brown sub-basal, ventral area and apex. Mid tibia dashed with pale and brown, hind tibia pale with brown suffusions, the apex brown. Tarsi pale brown. ***Wings*.** Forewing mostly hyaline; membrane surrounding crossveins, R fork, RP branches stem, first branch of CuA and apex of CuP, and terminal forks of longitudinal veins amber; a zigzagged amber mark connecting MA and MP forks, terminal part of MA and 2r-m and 1ma-mp, plus an amber area between CuP and posterior wing margin are frequent. Pterostigma brown with pale medial area. Major veins, subcostal veinlets and wing margin alternating pale and dark reddish brown; crossveins dark reddish brown. Hind wing hyaline, amber on area adjacent to stem of first branch of CuA; pterostigma brown with pale, preapical area; longitudinal veins alternating pale and dark brown areas; subcostal veinlets nearly transparent; crossveins brown, 1r-m often bicolor; wing margin alternating pale and brown. ***Abdomen*.** Tergites dark reddish brown. Pleural membrane pale with dark reddish brown suffusions. Sternites pale with wide, dark reddish brown, lateral areas, setation mostly pale.

***Morphology*** (Fig. 28). ***Head*.** Diamond-shaped in frontal view, rugose; vertexal region raised above compound eyes, with lateral rows of reclined setae; area surrounding coronal suture glabrous, with muscle insertion mark; coronal suture distinct; medial area of ​​supra-antennal region not raised, with fine, reclined setae; paraocular area concave. Antenna sub-moniliform, nearly as long as head and thorax together; scape as long as wide, cup-shaped, ventrally with a few thickened setae; pedicel as long as wide; flagellum not dorsoventrally flattened, with 45–64 flagellomeres, those of proximal ¼ discoidal, then changing from two times as long as wide to as long as wide; all articles with medial ring of fine, short setae. Compound eye hemispherical, as wide as ½ of the interocular distance at torulus level. Frons and clypeus narrow. Labrum pentagonal; maxillary palpus with first palpomere as long as wide, second 1.2× as long as wide, third palpomere 4× as long as wide, fourth palpomere 2.5× as long as wide, fifth palpomere slightly longer than third; mentum with long, thin setae; labial palpus with first palpomere two times as long as wide, second palpomere 4× as long as wide, third palpomere slightly longer than second, palpimacula ovoid. ***Thorax*.** Pronotum as long as wide, with raised anterior margin, medial and posterior regions; outgrowths covered with pedicellate, thick setae. Mesonotum slightly wider than long, with abundant, thick, pedicellate setae on medial area. Metanotum ~ 2× as wide as long. Pteropleura with short, thin setae, thicker on mesanepisternum and metakatepisternum. ***Foreleg*.** Coxa as long as femur, cylindrical, anterior and posterior surfaces with pedicellate, fine or thick setae of different sizes; trochanter trapezoidal, dorsal surface with some thickened, pedicellate setae near distal margin; anterior surface without protuberant area. Femur robust, closing surface with posteroventral row of processes composed two medially located, primary processes; proximal portion of the row with sub-basal secondary process and basal tertiary process; the rest of the row with abundant tubercle-shaped processes, and stinger-shaped setae; distal portion carinate, composed of tubercle-shaped specializations and stinger-shaped setae; adjacent row of thickened setae with globular base present on distal ¼. Anteroventral row of processes reduced to proximal ½; it is composed of tubercle-shaped specializations and stinger-shaped setae; the basal-most primary, curved process is present; distal portion composed of few tubercle-shaped processes; adjacent row of thickened setae with globular base present on distal ⅘. Tibia almost as long as femur, curved, ventral surface keeled with row of prostrate setae; patch of clavate setae apically on anterior surface present. Basitarsus with lanceolate process reaching base of fourth tarsomere; clavate setae present proximally on anterior surface; ventrally with single row of prostrate setae; second tarsomere nearly 9× as long as wide; third tarsomere 1.5× as long as wide; fourth tarsomere two times as long as wide. ***Mid- and hind leg*.** Coxa and trochanter with short, thin setae; femora with interspersed fine setae of different lengths and few thickened setae; tibiae mostly with short fine setae; hind leg longer than midleg, tibia 1.5× as long as femur; tarsomeres with distal margin of plantar surface with lateral groups of 5–8 thickened setae; on both legs, basitarsus 3.5 times as long as wide, second tarsomere 1.2× as long as wide; third and fourth tarsomeres as long as wide; fifth tarsomere two times as long as wide. ***Wings*.** Forewing narrowly oval, trichosors present along margin except on wing base; costal space proximally expanded, humeral vein branched; 12–15 subcostal veinlets; pterostigma elongated, narrow, straight, with incomplete veinlets; subcostal space with single, medially located crossvein; Sc vein abruptly posteriorly bent at proximal pterostigma margin to merge with RA; radial space with two or three crossveins; *rarp2* gently curved with three or four RP branches; three or four veins arising from *rarp1*; M vein basally fused with RA; RP base widely separated from divergence of M and R; M forked opposite to RP origin; 1 r-m connecting RP base and M fork, forming a trapezoidal cell; 3–6 gradate crossveins present. Cubitus deeply forked; CuP basally angled and approaching A1, distally forked slightly beyond the level of separation of M and R; A1 apically forked, ending on posterior margin at level of CuP fork, A2 forked slightly beyond CuP angle level. Hind wing smaller and narrower than forewing, narrowly oval; costal space narrow and reduced, with 5–9 veinlets; C and Sc fused at ¼ of wing length, Sc vein abruptly curved posteriad at proximal margin of pterostigma to merge RA; pterostigma elongated, narrow, gently curved, composed of incomplete veinlets; radial space with single crossvein, oblique; three or four veins arising from *rarp1*, one or two from *rarp2*. 1r-m sigmoid, connecting the stems of M and RP. Media forked beyond R fork. Cubitus deeply forked, intracubital crossvein subparallel to longitudinal wing axis; CuA sinuous, first branch candelabrum-shaped, spur vein absent or present; CuP not touching A1, strongly anteriorly bent at distal ½, pectinate, sometimes fused to first branch of CuA; two crossveins on cubitoanal space; A1 apically simple or forked, ending on wing margin at 1r-m stem level; A2 simple, short, and curved. ***Abdomen*.** Cylindrical to medially expanded, with subquadrate tergites; tergites III and IV with posteromedian concavity; tergites III-VII with elongated anterolateral scars; sternites rectangular.

**Figure 29.**
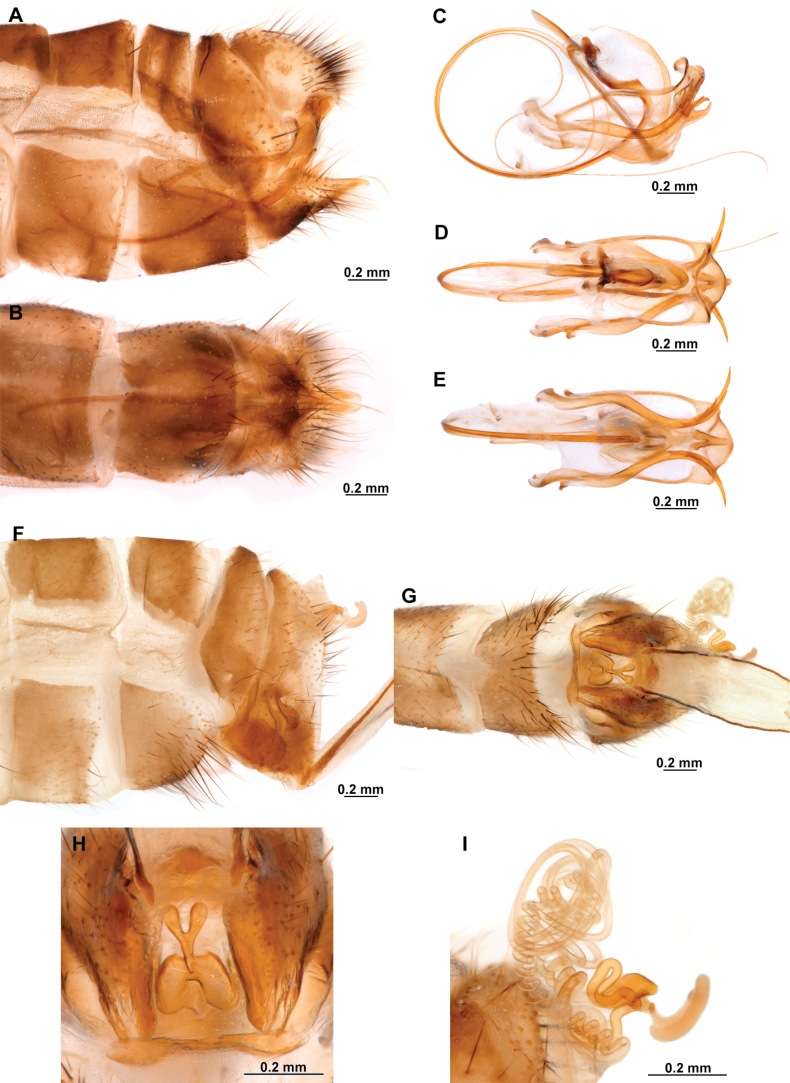
*Plegabanksi* Rehn, 1939 **A** male terminalia, lateral **B** same, ventral **C** male genitalia, lateral **D** same, dorsal **E** same, ventral **F** female terminalia, lateral **G** same, ventral **H** gonapophyses VIII of female, ventral **I** spermatheca.

***Male genitalia*** (Fig. 29A–E). Tergite IX medially narrower than laterally; lateral margin rounded. Sternite VIII rectangular; sternite IX pentagonal in ventral view, with abundant setae; posterior margin with medial, elongated, blunt lobe which is dorsally canaliculated; in lateral view triangular, apex surpassing posterior margin of ectoproct. Gonocoxites IX short, thickened, sinusoid; base flattened, spatulated; apex laterally curved, branched, with 4–7 processes of different lengths. Ectoproct ovoid, covered with abundant, thickened setae on posterior surface, posteroventrally with blunt lobe; anteroventrally with flattened, rounded lobe that is continuous with ventromedial sclerotized, curved sulcus. Gonocoxites X forming a short, ventrally canaliculated sclerite, whose posterior apex has dorsal processes connected to gonostyli X and ventrolateral processes connected to gonapophyses X; gonostyli X with thickened and curved base, equipped with two lateral processes, the rest of the structure, ventrally curved, and anteriorly coiled, forming two loops before protruding from abdomen. Gonapophyses X long, straight, narrow, with posterior apex slightly expanded; gonapophyses arranged in a V-shaped structure, joined by a membrane covering the gonostyli X base. Gonocoxites XI thin, U-shaped, medial lobe complex and elaborated, with two differentiated parts: dorsal part as rounded, anteriorly curved lobe; ventral part with a convex area covered with microspinules, continuous with ventral, curved, prominent hook-shaped process, whose apex is covered with microspinules; between these parts a narrow, less sclerotized, hyaline area is present; lateral arms of gonocoxites XI gently sigmoid, with anterior apex straightly angled. Hypandrium internum triangular.

***Female genitalia*** (Fig. 29F–I). Sternite VII rectangular with broad posteromedian concavity connected with medial, narrow, glabrous, sclerotized area; with long, thickened setae near posteromedian concavity. Tergite VIII conspicuously narrower medially than laterally, encircling the spiracle of the segment; lateral margin D-shaped. Gonocoxites VIII as a sub-rectangular, concave sclerite, posteromedially connected to gonapophyses VIII. Gonapophyses VIII with medial part chamber-shaped, ventral surface with lateral convex areas, and lateral surfaces concave, posteromedially with an elongated, Y-shaped process; lateral part of gonapophyses VIII oval. Tergite IX + ectoproct D-shaped. Gonocoxites IX remarkably elongated, sinuous, and narrow, nearly as long as the whole abdomen. Bursa copulatrix short with proximal part strongly expanded, ovoid, and sclerotized, continuous with a membranous and striated part, which is abruptly narrowed. Spermatheca long, complex and entangled; proximal section thin, long, forming numerous coils; medial section as wide as proximal section, forming a long spiral; distal section entangled, as wide as medial section, terminating in an expanded portion, where a blunt diverticulum is present; fertilization canal duct proximally triangular, concave; fertilization canal elongated, pod-shaped, covered with microfilaments.

##### Distribution.

Mexico (Sinaloa, Sonora), USA (Arizona).

##### Remarks.

This species appears to have a more restricted distribution than the closely related *P.dactylota*, being distributed in northern Mexico (Sinaloa and Sonora) and Arizona. Specimens of Sinaloa are slightly different to those from Arizona, particularly in the overall morphology of male genitalia, although there are not enough differences to consider the specimens from the former location as a different species. Such difference is interpreted as part of intraspecific variation, probably related to size or geographic distribution. Records from the Mexican state of Chihuahua presented by Oswald et al. (2002) and Reynoso-Velasco and Contreras-Ramos (2008) are more likely erroneous and probably represent specimens of the closely related species *P.megaptera*.

Specimens of this species may be difficult to separate from *P.dactylota*, particularly when only females are available. However, the general dark reddish brown body coloration pattern with a cream medial band on the mesonotum, the strongly marked forewing and the longer ovipositor are characters that allow to make a relatively reliable distinction between both.

#### ﻿﻿ Plega bowlesi

Taxon classificationAnimaliaNeuropteraRhachiberothidae

Ardila-Camacho & Contreras-Ramos sp. nov.

https://zoobank.org/2E291328-1E3B-4370-B765-ABA930739309

Figs 30 , 31

##### Type locality.

Ecuador, [**Guayas**]: Guayaquil, Rosemberg leg.

##### Material examined.

***Holotype*** male, pinned, with genitalia in a separate vial. Original label: “Ecuador, Guayaquil, Rosemberg coll., Acq. 1903”; FSCA.

##### Diagnosis.

This species presents the antennal flagellomeres as long as wide at proximal region of flagellum; three preapical flagellomeres are pale. The wings are broadly oval, and the forefemur is narrow with brown dots on the posterior surface. On the male genitalia, the sternite IX is pentagonal in ventral view, with rounded posterolateral lobes. The gonocoxite IX is long, gently sigmoid, with posterior apex straight, thickened, set with elongate preapical process on inner surface and seven or eight short processes on outer surface, arranged in a single plane. Additionally, the ventral part of the gonocoxites XI forms covering with two lateral concavities; such covering is ventrally produced as a straight, trapezoidal process.

##### Etymology.

This species is named after David E. Bowles, a great person, aquatic entomologist and neuropterologist, who has steadily helped AAC in their studies in Neotropical Neuropterida.

##### Description.

***Measurements*.** Male (*n* = 1). Forewing length: 10.4 mm; Hind wing length: 8.3 mm.

**Figure 30.**
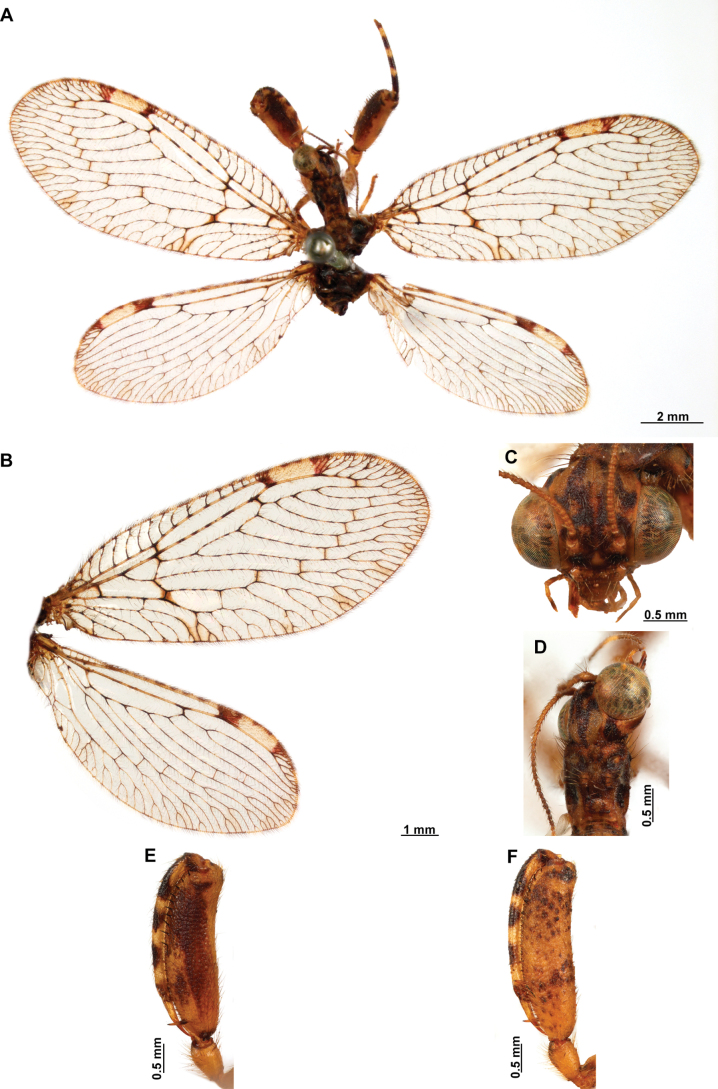
*Plegabowlesi* Ardila-Camacho & Contreras-Ramos, sp. nov. **A** male habitus, dorsal (abdomen removed) **B** wings **C** head, frontal **D** pronotum, dorsal **E** forefemur, anterior Surface **F** same, posterior surface.

***Coloration*** (Fig. 30). ***Head*.** Vertexal region pale, with lateral dark brown markings extending from occiput to toruli; supraantennal area dark brown with lateral, small pale areas; pale brown setae present; occiput pale with sark brown spot, postgena pale with brown area. Antennal scape pale brown with dorsal, darker, longitudinal band, entire surface with brown setae; pedicel brown; flagellum brown with three pale, preapical flagellomeres. Frons with dark brown trapezoidal areas below toruli, medial region with pale triangular area. Clypeus pale. Labrum pale brown; mandible pale brown with dark brown corners, amber at apex; maxilla brown with darker areas, palpus pale brown with darker rings on third and fifth palpomere; labium pale brown with darker postmentum, ligula and first two palpomeres; palpimacula pale brown. ***Thorax*.** Pronotum medially pale with brown areas, area adjacent to lateral margins dark brown, setae brown, with some paler; episternum pale brown; postfurcasternum pale with posterior area brown. Mesonotum pale with brown areas on the medium and laterally dark brown; metanotum mostly dark brown; pre-episternum brown; pteropleura brown with pale on margins of sclerites, setation mostly pale brown. ***Foreleg*.** Coxa pale, with a few, small, brown marks, setae mostly brown, with some paler; trochanter pale with small, brown marks, setation mostly pale brown, with some darker on dorsal and anterior surfaces. Femur posterior surface pale with brown dots, apex brown; anterior surface pale with extensive brown area; processes pale brown. Tibia dashed with pale and dark brown. Basitarsus with base and lanceolate process pale brown, sub-basal area pale, clavate setae pale brown; remaining tarsomeres pale brown. ***Mid- and hind leg*.** Mid- and hind coxae mostly brown, setae pale brown; trochanter pale with brown suffusions. Femur of mid-leg and tibia of both legs dashed with pale and dark brown bands, femur of hind leg pale; tibial spurs pale brown. Tarsi pale brown; first four tarsomeres with brown setae, laterally on distal margin of plantar surface; pretarsal claws pale brown. ***Wings*.** Forewing mostly hyaline; membrane surrounding crossveins, M fork, first branch of CuA and apex of CuP amber; intermittent amber areas between terminal forks of longitudinal veins on distal ½ of wing; pterostigma brown with wide, pale, medial area; major veins, subcostal veinlets and wing margin alternating pale and brown. Hind wing hyaline; pterostigma brown with wide, pale, preapical area; longitudinal veins alternating pale and brown, subcostal veinlets pale brown; crossveins brown, except sigmoid 1r-m, bicolor; wing margin alternating pale and brown. ***Abdomen*.** Cleared.

***Morphology* (Fig. 30)**. ***Head*.** Diamond-shaped in frontal view, rugose; vertexal region raised above compound eyes, with lateral rows of reclined setae; area surrounding coronal suture glabrous, with muscle insertion mark; coronal suture distinct; medial area of ​​supra-antennal region slightly raised, with fine, reclined setae; paraocular area concave. Antenna sub-moniliform, short; scape 1.5× as wide as long, slightly distally expanded, with fine, short setae; pedicel as long as wide; flagellum not dorsoventrally flattened, with 38 flagellomeres, those of proximal ¼ of the flagellum as long as wide; slightly longer than wide on the rest of flagellum; all articles with medial ring of fine, short setae. Compound eye hemispherical, as wide as ½ of the interocular distance at torulus level. Frons and clypeus narrow, with fine and short setae. Labrum pentagonal with thin, short setae; maxillary palpus with first palpomere as long as wide, second 1.2× as long as wide, third palpomere 3.5× as long as wide, fourth palpomere 2.5× as long as wide, fifth as long as third, all with short and thin setae; mentum with long, thin setae; labial palpus with first palpomere 2× as long as wide, second palpomere 4× as long as wide, third palpomere slightly longer than second, palpimacula narrowly ovoid. ***Thorax*.** Pronotum slightly longer than wide, with raised anterior margin, medial and posterior regions; outgrowths covered with pedicellate, thick setae; remaining surface with fine, short setae. Mesonotum slightly wider than long, with scattered, thick, pedicellate setae on medial area. Metanotum ~ 2× as wide as long, with a few fine, short setae. Pteropleura with short, thin setae. ***Foreleg*.** Coxa as long as femur, cylindrical, anterior and posterior surfaces with abundant fine setae of different lengths; trochanter trapezoidal, with thin and short setae, except on dorsal and anterior surfaces with some thickened, pedicellate setae; anterior surface with protuberant area. Femur robust, covered with abundant, fine, short setae; closing surface with posteroventral row of processes composed two medially located, primary processes, with single secondary process between them; proximal portion of the row with two secondary processes; the rest of the row with abundant tubercle-shaped processes, stinger-shaped setae; distal portion raised composed of tubercle-shaped specializations and stinger-shaped setae; adjacent row of thickened setae with globular base present on distal ¼. Anteroventral row of processes reduced to proximal ½; it is composed of tubercle-shaped specializations and stinger-shaped setae; the basal-most primary, curved process is present; distal portion composed of a few tubercle-shaped processes; adjacent row of thickened setae with globular base present on distal ½. Tibia almost as long as femur, curved, with thin, short setae; ventral surface keeled with prostrate setae; a patch of clavate setae apically on anterior surface is present. Basitarsus with lanceolate process reaching the middle of fourth tarsomere; clavate setae present proximally on anterior surface; ventrally with single row of prostrate setae; second tarsomere nearly 7× as long as wide; third tarsomere as long as wide, fourth tarsomere 2.5× as long as wide. ***Mid- and hind leg*.** Coxa and trochanter with thin setae, shorter on trochanter; femora and tibiae with fine setae of different lengths; tibial spurs short; hind leg longer than midleg, tibia, 1.5× as long as femur; tarsi with fine and short setae, except on distal margin of plantar surface with lateral groups of three or four thickened setae; on both legs, basitarsus 4× as long as wide, second tarsomere 1.2× as long as wide; third and fourth tarsomeres as long as wide; fifth tarsomere two times as long as wide. ***Wings*.** Forewing oval, trichosors present along margin except on wing base; venation setose; costal space proximally expanded, humeral vein branched, 15 or 16 subcostal veinlets, where one or two are forked; pterostigma trapezoidal, straight, with incomplete veinlets; subcostal space with single crossvein, nearly opposite to R fork; Sc vein abruptly posteriorly bent at proximal pterostigma margin to merge with RA; radial space with two crossveins; *rarp2* curved with three RP branches; three veins arising from *rarp1*; M vein basally fused with RA; RP base not widely separated from divergence of M and R; M forked slightly before RP origin, 1 r-m connecting RP base and M fork, forming a trapezoidal cell; five or six gradate crossveins present. Cubitus deeply forked; CuP basally angled and approaching A1, distally forked slightly beyond the level of separation of M and R; A1 simple, ending on posterior margin slightly beyond the level of CuP fork, A2 forked slightly beyond CuP angle level. Hind wing smaller and narrower than forewing, oval; costal space narrow and reduced, with 6 veinlets; C and Sc fused at ¼ of wing length, Sc vein abruptly curved posteriad at proximal margin of pterostigma to merge RA; pterostigma elongated, slightly subapically expanded, straight, composed of incomplete veinlets; radial space with single crossvein, oblique; three or four veins arising from *rarp1*, one from *rarp2*. 1r-m sigmoid, connecting the stems of M and RP. Media forked beyond R fork. Cubitus deeply forked, intracubital crossvein subparallel to longitudinal wing axis; CuA gently sinuous, first branch candelabrum-shaped, spur vein absent; CuP not touching A1, strongly anteriorly bent at distal 1/3, pectinate; two crossveins on cubitoanal space; A1 simple, ending on wing margin at 1r-m stem level; A2 simple, short, and curved. ***Abdomen*.** Cylindrical to medially expanded, setae on tergites, scattered, thin, and short, gradually longer towards terminal segments; tergites subquadrate. Sternites rectangular, with abundant, scattered, thin setae.

**Figure 31.**
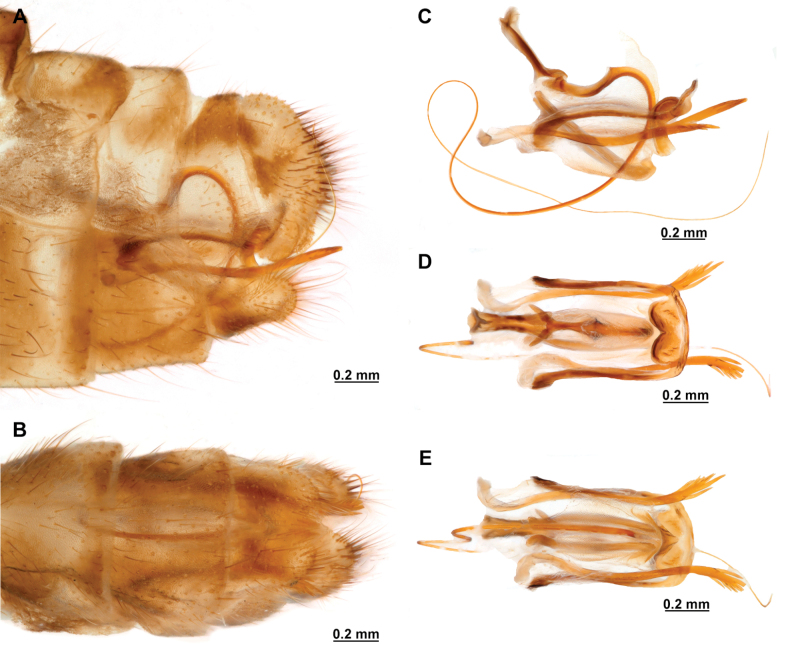
*Plegabowlesi* Ardila-Camacho & Contreras-Ramos, sp. nov. **A** male terminalia, lateral **B** same, ventral **C** male genitalia, lateral **D** same, dorsal **E** same, ventral.

***Male genitalia*** (Fig. 31). Tergite IX medially narrower than laterally; lateral margin rounded. Sternite VIII quadrangular; sternite IX pentagonal in ventral view, with rounded posterolateral lobes covered with abundant, long setae; posterior margin with medial, short, triangular lobe which is dorsally canaliculated; in lateral view bluntly trapezoidal, apex not reaching posterior margin of ectoproct. Gonocoxites IX long, gently sigmoid; base flattened, dorsally curved, connected to gonocoxites XI with a membrane; apex straight, thickened, with elongate preapical process on inner surface and seven or eight short processes on outer surface, arranged in a single plane. Ectoproct ovoid, covered with thickened setae on posterior surface, posteroventrally with rounded lobe covered with fine, pedicellate setae; Gonocoxites X forming a short, thickened, ventrally canaliculated sclerite, with anterior apex dorsally bent, posterior apex with dorsal processes connected to gonostyli X and ventrolateral processes connected to gonapophyses X with a membrane; gonostyli X with thickened, obtuse base with two lateral processes, the rest of the structure, whip-shaped, ventrally curved, and anteriorly coiled, forming a single loop before protruding from abdomen. Gonapophyses X rod-shaped, straight, posterior apex slightly expanded; gonapophyses arranged in a V-shaped structure joined by a membrane covering the gonostyli X base. Gonocoxites XI thin, U-shaped, medial lobe complex and elaborated, with two differentiated parts: dorsal part as a narrow arch; ventral part forming covering with two lateral concavities, ventrally it is produced as a straight, trapezoidal process; between these parts a narrow, less sclerotized, hyaline area is present; lateral arms of gonocoxites XI gently curved, with anterior apex straightly angled, quadrangular.

##### Distribution.

Ecuador (Guayas).

##### Remarks.

This species is solely from its type locality in Guayas, Ecuador. It is remarkably similar to *P.hagenella* in the general coloration pattern and overall morphology, although the shape of the male gonocoxites IX and the gonostyli X rapidly differentiate both species. Another important difference is the shape of the ventral part of the median lobe of the gonocoxites XI.

#### ﻿﻿ Plega dactylota

Taxon classificationAnimaliaNeuropteraRhachiberothidae

Rehn, 1939

Figs 32 , 33

Plega dactylota Rehn, 1982: 423. Holotype: male, USA, Arizona (ANSP).
Plega dactylota lipanica Rehn, 1982: 423. Holotype: male, USA, Texas (ANSP). Rice (1987): 342.
Plega fratercula Rehn, 1939: 247. Holotype: male, USA, Arizona (MCZ), specimen examined. New synonym.

##### Material examined.

***Holotype*** of *Plegafratercula*

USA • ♂; **Arizona**, Capitain Mt.; 8 Aug. 1933; R. Anderson leg.; MCZ.

***Paratype*** of *Plegafratercula*.

USA • 1 ♀; **Arizona**, Capitan Mt.; 24 Jul. 1933; R. Anderson leg.; “Allotype *Plegafratercula* Rehn”; MCZ-ENT 00580916, MCZ type 25377; MCZ.

***Paratypes*** of *Plegadactylota*.

USA • 1 ♂; **Arizona**, Huachuca Mtn. Carr. Cn.; 06 Aug. 1924; J.O. Martin leg.; “*Plegadactylotadactylota* Rehn, Paratype”; CAS. • 1 ♂; Huachuca Mts.; 20 Jul. 1937; D.J. & J.N. Knull leg.; Paratype 25379; MCZ. • 1 ♂; Santa Rita Mts.; Jul.; 5–8000 ft; F.H. Snow leg.; Paratype 23379; MCZ. • 1 ♂; Boya Thomson, Aboretum, Superior; 1–3 Aug. 1937; MCZ Paratype 25378; MCZ. – **Texas** • 1 ♂; Hills W. of Ord Mts Brewster Co.; 22–31 Aug. 1926; O.C. Polling leg.; Paratype 25380, *Plegadactylotalipanica*; MCZ. • 1 ♂; Hills W. of Ord Mts Brewster Co.; 15–22 Aug. 1926; O.C. Polling leg.; Paratype 25380 *Plegadactylotalipanica*; MCZ. • 1 ♂; Big Bend Park, Brewster Co.; 14 Jun. 1937; R.H. Baker leg.; Paratype 25380, *Plegadactylotalipanica*; MCZ. • 1 ♂; Big Bend Park, Brewster Co.; 13 Jun. 1937; R.H. Baker leg.; Paratype 25380, *Plegadactylotalipanica*; MCZ.

##### Other material.

Mexico • 1 ♀; **Chihuahua**, 2 mi W. Matachic; 30 Jul. 1984; D. Thomas leg.; TAMU-ENTOX0285142; TAMUIC. – **Guerrero** • 1 ♀ 1 ?; Grutas de Cacahuamilpa; 02 Nov. 1967; R.E. Woodruff leg.; Blacklight trap; FSCA. – **Jalisco** • 1 ♀; San Buenaventura; 19°97'61.9"N, 104°03'32.4"W; 720 m; 03 Dec. 1996; S. Zaragoza; CNIN. • 1 ♂; San Buenaventura; 19°47.61'N, 104°03.32'W; 720 m; 07 Nov. 1996; light trap; CNIN. • 1 ♀; Chamela; 12 Jan. 1995; H. Brailovsky; CNIN. • 1 ♀; Estación de Biología Chamela; 24 Oct. 1985; R.A. Usela; trampa de luz; CNIN. • 1 ?; Río Cuitzmala; 24 Nov. 1984; E. Ramírez, J. Villa leg.; CNIN. • 2 ♀; El Limón, San Buenaventura; 620 m; 19°46.614'N, 104°03.324'W; 04 Nov. 1996; S. Zaragoza, F. Noguera, E. Ramírez, E. González leg.; 55037; CNIN. – **Morelos** • 1 ♂; Tlaquiltenango, 2.5 Km, 4 Km W Huautla Estación CEAMISH; 18°27'67.1"N, 99°02'47.5"W; 940 m; 16 Nov. 1995; S. Zaragoza, F. Noguera, E. Ramírez, E. González leg.; light trap 3, MDMD125; CNIN. • 1 ♂; Tlaquiltenango, Huautla; 18°23'04.27"N, 99°03'00.03"W; 1028 m; 18 Sep. 2009; V.H. Toledo, J.G. Martínez, E.V. Reza, Y.T. Viveros, N. Hernández leg.; light trap, selva baja caducifolia, SHM224; CNIN. • 1 ♂; Tlaquiltenango, Huautla; 18°22'45.01"N, 99°02'44.91"W; 1001 m; 20 Nov. 2009; V.H. Toledo, J.G. Martínez, E.V. Reza, Y.T. Viveros, N. Hernández leg.; selva baja caducifolia, light trap, SHM215; CNIN. • 1 ♀; Tlaquiltenango, Huaxtla; 18°23'8.736"N, 99°3'4.0068"W; 1023 m; 28~31 Jan. 2009; V.H. Toledo leg.; selva baja caducifolia, light trap, SHM113; CNIN. • 1 ♀; Tlaquiltenango, 3 Km E. Santiopan; 18°25'45.66"N, 98°55'56.136"W; 1381 m; 28 Jan. 2014; V.H. Toledo, R. Reyes, J. Martínez leg.; selva baja caducifolia, light trap, SHM124; CNIN. • 1 ♀; El Limón, atrás de estación; 14 Aug. 2015; C. Pérez, P. Bogadrín, G. Gutiérrez leg.; trampa pit light, luz blanca; CNIN. – **Nayarit** • 1 ♀; P.H. Aguamilpa, Arroyo de los Bueyes; 29~30 Oct. 1991; R. Barba & E. Barrera leg.; CNIN. – **Nuevo León** • 1 ♂; Bustamante, at entrance of Grutas del Palmito; 08 Oct. 1966; D. Riley; TAMU-ENTOX0070934; TAMUIC. • 1 ♂; Nuevo Leon, nr Monterrey, Mesa de Chipinque; 1365 m; 16–18 Jul.; “Cornell Univ. Mexico Field Party 1965”; MCZ-ENT00681790; MCZ. – **Puebla** • 1 ♂; Jolalpan, Rancho El Salado; 18°20'49.38"N, 98°59'23.02"W; 923 m; 06 Oct. 2010; V.H. Toledo, F. Hinterholzer, J.G. Martínez leg.; selva baja caducifolia, light trap, SHM221; CNIN. • 1 ♀; Jolalpan, Rancho El Salado; 18°20'49.38"N, 98°59'23.028"W; 923 m; 06 Oct. 2010; V.H. Toledo, F. Hinterholzer, J.G. Martínez leg.; selva baja caducifolia, light trap, SHM178; CNIN. – **Querétaro** • 1 ♂; Parque Nacional La Joya, La Barreta (cerca a la Monja); 220 m; 06 Aug. 2013; H. Brailovsky, E. Barrera leg.; CNIN. – **Sonora** • 1 ♂; Nacozari de García, 15.9 Km SE. Nacozari de García, Rancho La Zulema, Sierra Juriquipa; 30°17'2.04"N, 109°33'37.08"W; 1987 m; 15 Jul. 2017; Van Devender, J. Palting leg.; rocky mountainside, oak Woodland; USNMENT001541895; USNM. • 2 ♀ 1 ?; Nacozari de García, 15.9 Km SE. Nacozari de García, Rancho La Zulema, Sierra Juriquipa; 30°17'2.04"N, 109°33'37.08"W; 1687 m; 15 Jul. 2017; Van Devender, J. Palting, leg.; rocky mountainside, oak Woodland; CNIN. • 9 ♂ 12 ♀ 2 ?; Nacozari de García, 15.9 Km SE. Nacozari de García, Rancho La Zulema, Sierra Juriquipa; 30°17'2.04"N, 109°33'37.08"W; 1687 m; 14 Aug. 2017; Van Devender, J. Palting leg.; rocky mountain side, oak Woodland; *Plegadactylota* Rehn, det. O.S. Flint, 2017; CNIN. • 3 ♀; same data as for preceding; 15 Jul. 2017; CNIN. • 2 ♂ 6 ♀; same data as for preceding; 30°17'45.24"N, 109°36'44.64"W; 14 Jul. 2017; CNIN. • 2 ♂ 2 ♀; Nacozari de García, 15.9 Km SE. Nacozari de García, Sierra Juriquipa; 30°17'45.24"N, 109°36'44.64"W; 1687 m; 14 Jul. 2017; Van Devender, J. Palting leg.; rocky montain side, oak Woodland; CNIN. • 1 ♂; same data as for preceding; *Plegadactylota* Rehn, det. O.S. Flint, 2017; CNIN. • 2 ♀; Nacozari de García, 15.9 Km SE. Nacozari de García, Sierra Juriquipa; 30°17'1.68"N, 109°40'9.48"W; 1687 m; 15 Jul. 2017; Van Devender, J. Palting leg.; rocky mountain side, oak Woodland; CNIN. • 1 ♀; Nacozari de García, Pilares de Nacozari, 6.5 Km (by air) SE of Nacozari de García, Sierra Nacozari; 30°19'41.988"N, 109°37'46.992"W; 1413 m; 09 Aug. 2015; T.R. Van Devender, A.L. Reina-G leg.; rocky slope; CNIN. • 4 ♂ 3 ♀; Nacozari, rancho El Metate, 7.4 Km SSW Nacozari, E. base of Sierra San José; 31°15'30.96"N, 109°57'33.12"W; 1634 m; 16 Sep. 2017; Van Devender, J. Palting leg.; desert grassland, oak Woodland; CNIN. • 1 ♀; Sonora, Agua Caliente; 25 Aug. 1959; R.E. Ryckman, C.P. Christianson & D. Spencer leg; USNM. • 2 ♂ 4 ♀; Mpio. Álamos, Parque La Colorada, sendero Arroyo alto (Chalatón); 27°00.551'N, 108°57.206'W; 510 m; 17 Sep. 2019; Contreras, Barba, Cancino, Ardila, Marquez leg.; selva baja caducifolia, black and white light trap; CNIN. • 4 ♂ 1 ♀; Mpio. Álamos, Parque La Colorada, sendero Tecolote; 27°00.675'N, 108°56.987'W; 469 m; 16 Sep. 2019; Contreras, Barba, Cancino, Ardila, Marquez leg.; selva baja caducifolia, black and white light trap; CNIN. • 2 ♂; Mpio. Moctezuma, Rancho La Montosa, camino; 29°34.201'N, 109°58.352'W; 930 m; 22 Nov. 2019; Barba, Ardila, Cancino, Marquez, Contreras leg.; black and White light trap, bosque espinoso; CNIN. • 1 ♀; 36.6 Km SE Tecoripa, La Barranca; 28°34'40.1"N, 109°39'54.3"W; 562 m; 11 Sep. 2004; S. Zaragoza, F. Noguera, E. Ramírez, E. González leg.; light trap 1, bosque tropical caducifolio, MDM0233, 2699; CNIN. • 1 ♀; same data as for preceding; MDM0243, 2720; CNIN. • 1 ♀; same data as for preceding; MDM0241, 2719; CNIN. • 1 ♂; same data as for preceding; 28°34'40.1"N, 109°39'54.3"W; 645 m; 16 Aug. 2004; MDM0262, 2722; CNIN. • 1 ♀; same data as for preceding; 28°34'40.1"N, 109°39'54.3"W; 645 m; 16 Aug. 2004; MDM0260, 2717; CNIN. • 3 ♂ 3 ♀; Rancho Los Jarazos, Sierra La Purica; 16 Jul. 2013; T.R. Van Devender, J. Palting leg.; CNIN. – **Tamaulipas** • 1 ♀; 43 km. east 27.V Ciudad Mante; FSCA.

USA – **Arizona** • 1 ♂ 1 ♀; Cochise Co., S.W. Research Sta., nr. Portal; 1982; L.L. Lampert leg.; black light trap; FSCA. • 1 ♂; Chochise County, SW Research Sta.; 27 Jul. 1989; A.M. & N.D. Penny leg.; at lights; *Plegadactylota* Rehn, det. N. Penny; CAS. • 1 ♂ 1 ♀; Cochise Co., Huachuca Mtns., Ash Canyon; 07 Aug. 1991; R. & J. Robertson leg.; *Plegadactylota*, det. Penny, 1991; CAS. • 2 ♀; same data as for preceding; 22 Jul. 1989; CAS. • 1 ♂; Cochise Co., Huachuca Mts., Ash Canyon; 5100’; 09 Aug. 2001; B.D. Valentine leg.; *Plegadactylota* Rehn, det. D.J. Shetlar, 1994 and Penny, 1997; CAS. • 1 ♂; Cochise Co., Huachuca Mtns., Ash Canyon; 27–31 Jul. 1986; D.L. Wagner leg.; black and white lights; CAS. • 2 ♂ 5 ♀; Cochise Co., Ramsey Cyn.; 31°26'57.84"N, 110°18'23.40"W; 1679 m; 22–24 Jul. 2017; Aalbu R.L. leg.; black light; CSCA. • 1 ♀; Cochise Co., 3.2 mi E. SERS; 16 Aug. 1971; D. Carlson leg.; CSCA. • 1 ♀; Gila Co., Carrizo; 4750’; 01 Sep. 1986; A.S. Menke leg.; USNM. • 1 ♂; Portal; 10 Jul. 1969; G.H. Nelson Family leg.; at lite; FSCA. • 1 ♂ Gila Co., Payson; 30 Aug. 2001; P.R. Coleman leg.; UCD. • 1 ♂; Maricopa Co., Jct. Fish, Ck. Cyn & Apache Trail; 01 Aug. 1987; W.B. Warner; U.V. light; FSCA. • 1 ♂; Santa Rita Mts., Madera Canyon; 14 Aug. 1949; 00045; “Phillip A. Adams Collection 1998 request to Calif. Acad. Sci.”; CAS. • 1 ♂; Santa Cruz Co., Duquesne Rd. (For. Ser. Rd. #61), 7.8 mi. E. Jct. Hwy. 82; 4800 ft.; 13 Aug. 1991; L.G. Bezark, D.E. Russell, R.A. Cunningham leg.; MV/BL, Oak/Mesq. Trans; *Plegafratercula*, det. N. Penny, 1997; CAS. • 1 ♀; Santa Cruz Co., Sta. Rita Mts., Madera Canyon, Sta. Rita Lodge; 27 Aug. 1991; R. Wielgus leg.; CAS. • 1 ♂; Santa Cruz Co., Madera Cyn.; 4880’; 15 Jun. 1965; D.N. Harrington leg.; UCD. • 3 ♂; same data as for preceding; 27 Jul. 1965; D.N. Harrington leg.; UCD. • 3 ♂; same data as for preceding; 07 Sep. 1965; D.N. Harrington leg.; UCD. • 1 ♂; same data as for preceding; 26 Aug. 1965; D.N. Harrington leg.; UCD. • 1 ♂; same data as for preceding; 21 Aug. 1965; D.N. Harrington leg.; UCD. • 1 ♂; same data as for preceding; 20 Aug. 1965; D.N. Harrington leg.; UCD. • 1 ♂; same data as for preceding; 15 Aug. 1965; D.N. Harrington leg.; UCD. • 1 ♂; same data as for preceding; 07 Sep. 1965; D.N. Harrington leg.; UCD. • 1 ♂; same data as for preceding; 15 Sep. 1965; D.N. Harrington; UCD. • 1 ♂; same data as for preceding; 01 Oct. 1965; D.N. Harrington leg.; UCD. • 1 ♂; same data as for preceding; 07 Sep. 1965; D.N. Harrington leg.; UCD. 1 ♂; same data as for preceding; 01 Oct. 1965; D.N. Harrington leg.; UCD. • 1 ♂; same data as for preceding: 17 Jul. 1963; V.L. Vesterby; UCD. 1 ♂; same data as for preceding;17 Jul. 1963; V.L. Vesterby; UCD. • 2 ♂; same data as for preceding; 21 Jul. 1963; V.L. Vesterby; UCD. • 5 ♂; same data as for preceding; 15 Aug. 1963; V.L. Vesterby; UCD. • 3 ♂; same data as for preceding; 12 Aug. 1963; V.L. Vesterby; UCD. • 3 ♂; same data as for preceding; 25 Aug. 1963; V.L. Vesterby; UCD. • 1 ♂; same data as for preceding; 08 Sep. 1963; V.L. Vesterby; UCD. • 1 ♂; same data as for preceding; 08 Jul. 1963; V.L. Vesterby; UCD. • 1 ♂; same data as for preceding; 22 Sep. 1963; V.L. Vesterby; UCD. • 1 ♂; same data as for preceding; 12 Sep. 1963; V.L. Vesterby; UCD. • 1 ♂; Maricopa Co., Sunflower, Jct. Hwy, 87 & Sycamore Ck.; 33°52'8.616"N, 111°27'51.732"W ± 100 m; 744 m; 15 Aug. 2012; Oswald, Diehl, Machado leg.; MV light; TAMU-ENTO X0965263; TAMUIC. • 1 ♀; same data as for preceding; TAMU-ENTO X0966388; TAMUIC. • 1 ♂; same data as for preceding; TAMU-ENTO X0966101; TAMUIC. • 1 ♂; same data as for preceding; TAMU-ENTO X0966424; TAMUIC. • 1 ♂; Pima Co., Madera Cyn., Santa Rita Mtns.; 4400 ft.; 1–2. Aug. 1975; Menke & Pulawsky leg.; CAS. • 2 ♂; Pima Co., Santa Rita Mts Experimental Range, Florida Cyn.; 31°45.42'N, 110°50.43'W; 1646 m; 03–10 Jul. 2014; M.I. Erwin, C. O’ Brien, M.J. Sharkey leg.; Malaise on hillside road; CSCA. • 1 ♀; same data as for preceding; 23 Aug. 2014; CSCA. • 2 ♀; same data as for preceding; 10–20 Sep. 2014; CSCA. • 1 ♂; Pima Co., Sabino Cyn.; 03 Sep. 1962; V.L. Vesterby; UCD. • 1 ♂; same data as for preceding; 04 Sep. 1961; UCD. • 1 ♂; same data as for preceding; 14 Sep. 1963; UCD. • 1 ♂; Organ Pipe Cactus Nat. Mon.; 13 Oct. 1963; V.L. Vesterby; UCD. • 1 ♀; Mohave Co., 11 Km E. Kingman on Hualapai Mt. Rd.; 35°58.55'N, 113°55.63'W; 1490 m; 19 Jul–01 Aug. 2018; M.E Irwin leg.; Malaise in narrow wash nr Hualapai Mt. Recreational Park; CSCA. • 1 ♂ 1 ♀; Santa Cruz Co. Sonoita, 3.2 Km Town Center; 31°38'N, 110°39'W; 1524 m; 21 Jul.–06 Aug. 2014; E.E. Grissell leg.; Malaise in juniper-oak-grass land; CSCA. • 1 ♂ 1 ♀; Gila Co., W. side of Pinal Mtn.; 14 Aug. 1977; R.P. Allen leg.; black light; CSCA. • 1 ♂; Gila Co., 5 mi N Payson; 05 Sep. 1961; J.S. Bucket leg.; UCD. • 1 ♂; same data as for preceding; 05 Sep. 1961; UCD. • 1 ♀; 5 mi. Portal Chiricahua Mts.; 17 Aug.1958; G.B. Pitman leg.; UCD. • 1 ♂; 15 mi. W. Portal Chiricahua Mts.; 04 Aug. 1958; R.E. Rice; UCD. – **New Mexico** • 1 ♂; Grat Co., Hwy. #15, 5.5 Km S Jct. #35 3258/10812; 2165 m; 22 Jul. 1983; E.L. & K.W. Sleeper, B.L. leg.; Pine Oak Fst Mdw; *Plegadactylota*, det. N. Penny, 1998; CAS. • 1 ♂; Catron Co., Gila N.F., Whitewater Pub. Camp.; 11–14 Jul. 1970; J.A. Gruwell leg.; *Plegadactylota*, det. N. Penny, 1998; collected at blacklite; CAS. • 1 ♀; Dona Ana Co., Aguirre Sprgs RA; 5600’; 26–27 Aug. 1997; Wappes & Turnbow leg.; TAMU-ENTOX0070958; TAMUIC. • 1 ♀; same data as for preceding; TAMU-ENTOX0070968; TAMUIC. • 1 ♀; same data as for preceding; TAMU-ENTOX0070972; TAMUIC. • 8 ♂; Lincoln Co., HWY 7018-50 Km E Ruidoso; 33°22.1'N, 105°29.9'W; 01 Jul. 2013; C.M. Parker leg.; funnel light trap; UCD. • 10 ♂; same data as for preceding; 01 Jun. 2013; UCD. – **Texas** • 1 ♂; Jeff Davis Co., Davis Mountains Resort; 30°37'42"N, 104°05'01"W; 5800 ft.; 17 May. 2001; D.G. Marqua leg.; FSCA. • 1 ♀; Jeff Davis Co., Davis Mtns. Resort; 5800’; 31 Aug. 1999; D.G. Marqua leg.; TAMU-ENTOX0085828; TAMUIC. • 1 ♂; Jeff Davis Co. rest stop, 9.5 mi. S. jct. hwy 118 on 166; 09 Aug. 1992; W. Godwin & E. Riley leg.; UV light; TAMU-ENTOX0070888; TAMUIC. • 1 ♂; Jeff Davis Co., Davis Mountains Resort; 30°37'42"N, 104°05'01"W; 13 Aug. 2001; 5800 ft.; D. Marqua leg.; UV; UCD. • 1 ♀; same data as for preceding; 15 Aug. 2001; UCD. • 1 ?; Brewster Co., Big Bend Park; 26 Jul. 1937; R.H. Baker; TAMU-ENTOX0070916; TAMUIC. • 1 ?; same data as for preceding; 15 Jul. 1937; TAMU-ENTOX0071037; TAMUIC. • 1 ♂; Brewster Co., BBNP, Basin Area; 29°16'12"N, 103°18'01"W; 18–19 Jul. 2002; E.G. & C.M. Riley leg.; at lights; *Plegadactylota* Rehn, 1930, det. J.D. Oswald, 2004; TAMU-ENTOX0281148; TAMUIC. • 1 ♀; same data as for preceding; TAMU-ENTOX0281153; TAMUIC. • 1 ♂; Brewster Co., Big Bend National Park, Basin Lodge; 5400 ft.; 08 Jul. 2001; D.G. Marqua leg.; *Plegadactylota* Rehn, 1930, det. J.D. Oswald, 2002; TAMU-ENTOX0088735; TAMUIC. – **Utah** • 1 ♂; Juab Co., Hwy. 132 E. of Nephi, 1.5 mi. S. of Nebo Loop Rd.; 21 Mar. 1998; R.C. Mower leg.; *Plegadactylota* Rehn, 1930, de J.D. Oswald, 2003; TAMU-ENTOX0090578; TAMUIC. • 1 ♀; Juab Co., Hwy. 132 E. of Nephi, 1.5 mi. S. of Nebo Loop Rd.; 21 Aug. 1998; R.C. Mower leg.; TAMU-ENTOX0090575; TAMUIC.

##### Diagnosis.

This species is distinguished by the long antennae with discoidal basal flagellomeres. On the pronotum, often lateral pale bands are present. The forefemur posterior surface is either nearly completely brown or pale with two pairs of brown areas near the medial region. On the male genitalia, the gonocoxite IX is sinusoidal, with posterior apex equipped with 4–15 closely adpressed, twisted, digitiform processes. The median lobe of the gonocoxites XI has the dorsal part as an expanded arch with a less sclerotized medial area from which a ventrally incised process protrudes; the ventral part consists of a ventral, curved trapezoidal process. On the female genitalia, the gonocoxites + gonapophyses VIII medial part is keel-shaped, formed by to oval, medially joined plates; such a keel forms a posteromedial curved, bilobed process. The bursa copulatrix is short, conical, and moderately sclerotized. The spermatheca is thin, with the proximal section forming several coils; the medial section is a long spiral; the distal section is slightly expanded forming a D-shaped diverticulum.

##### Description.

***Measurements*.** Male (*n* = 8). Forewing length: 7.3–12.84 mm; Hind wing length: 5.4–9.78 mm. Female (*n* = 12): Forewing length: 10.7–19.5 mm; Hind wing length: 8.7–15.3 mm.

**Figure 32.**
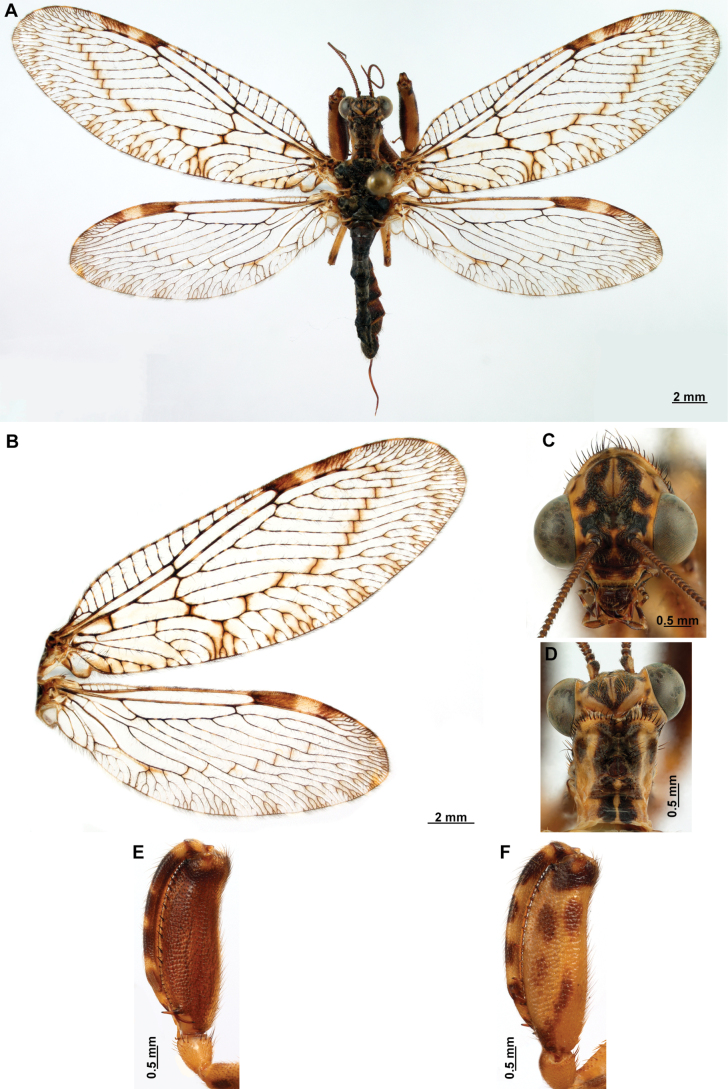
*Plegadactylota* Rehn, 1939 **A** female habitus, dorsal **B** wings **C** head, frontal **D** pronotum, dorsal **E** forefemur, anterior surface **F** same, posterior surface.

***Coloration*** (Fig. 32). ***Head*.** Pale with dark brown marks; vertexal pale with lateral, parallel, dark brown bands extended from occipital suture to supra-antennal area and enclosing lateral pale areas at level of paraocular region; area adjacent to occipital ridge dark brown; area adjacent to coronal suture pale to brown, sometimes arrow-shaped; supra-antennal area dark brown; brown setae present; occiput pale with dark brown mark, postgena pale with brown area. Antennal scape brown with pale outer surface, pedicel dark brown; flagellum pale to dark brown. Frons dark brown, sometimes with medial, pale area. Clypeus dark brown with pale anterior margin. Labrum brown; mandible pale with dark brown corners, dark amber at apex; maxilla pale with dark brown areas, palpus brown; labium pale with brown postmentum, ligula and palpus; palpimacula pale brown. ***Thorax*.** Pronotum pale with broad, brown, medial band, anterolateral areas dark brown; episternum brown; postfurcasternum pale. Mesonotum mostly dark brown, often with pale areas adjacent to sutures; scutellum sometimes with pale medial area. Metanotum dark brown, sometimes with pale anteromedian area; pre-episternum brown; pteropleura dark brown with pale areas. ***Foreleg*.** Coxa from pale with brown areas, to pale or dark brown or reddish brown; with interspersed pale and dark brown setae, sometimes mostly dark brown, setal bases pale to dark brown; trochanter pale with brown suffusions to brown with paler areas, setation as in coxa. Femur posterior surface either nearly completely brown or pale with two pairs of brown areas near medial region of femur; apex of posterior surface brown; anterior surface from brown to dark reddish brown, with pale proximal part of closing surface. Tibia posterior surface alternating pale and brown, anterior surface mostly dark brown, with preapical pale area. Basitarsus with base and lanceolate process dark amber, sub-basal area pale brown area, clavate setae brown; remaining tarsomeres pale brown. ***Mid- and hind leg*.** Coxae brown with paler areas; trochanter pale with brown areas. Femora and tibiae dashed, alternating pale and brown. Tarsi pale, becoming pale brown, towards the last tarsomere. ***Wings*.** Forewing mostly hyaline; membrane surrounding crossveins, R fork, stem of RP branches, M fork, first branch of CuA and apex of CuP, and terminal forks of longitudinal veins amber. Pterostigma brown with pale medial area. Major veins, subcostal veinlets and wing margin alternating pale and brown; crossveins dark brown. Hind wing hyaline, amber on area adjacent to stem first branch of CuA; pterostigma brown with pale preapical area; longitudinal veins mostly brown, with small pale areas; subcostal veinlets pale; crossveins brown, 1r-m bicolor; wing margin alternating pale and brown. ***Abdomen*.** Tergites dark brown. Pleural membrane dark brown with pale areas. Sternites brown, with paler medial and lateral areas.

***Morphology*** (Fig. 32). ***Head*.** Diamond-shaped in frontal view, rugose; vertexal region raised above compound eyes, with lateral rows of reclined setae; area surrounding coronal suture glabrous, with muscle insertion mark; coronal suture distinct; medial area of ​​supra-antennal region not raised, with fine, reclined setae; paraocular area concave. Antenna sub-moniliform; scape as two times as long as wide, ventrally with a few thickened setae; pedicel as long as wide; flagellum slightly dorsoventrally flattened, with 42–56 flagellomeres, those of proximal ¼ discoidal, then changing from two times as long as wide to as long as wide; all articles with medial ring of fine, short setae. Compound eye hemispherical, as wide as ½ of the interocular distance at torulus level. Frons and clypeus narrow. Labrum pentagonal; maxillary palpus with first palpomere as long as wide, second 1.5× as long as wide, third palpomere 4× as long as wide, fourth palpomere 4× as long as wide, fifth palpomere as long as third; mentum with long, thin setae; labial palpus with first palpomere 2.5× as long as wide, second palpomere 5× as long as wide, third palpomere slightly longer than second, palpimacula ovoid. ***Thorax*.** Pronotum as long as wide, with raised anterior margin, medial and posterior regions; outgrowths covered with pedicellate, thick setae. Mesonotum slightly wider than long, with abundant, thick, pedicellate setae on medial area. Metanotum ~ 2× as wide as long. Pteropleura with short, thin setae, thicker on mesepisternum and metakatepisternum. ***Foreleg*.** Coxa as long as femur, cylindrical, anterior and posterior surfaces with pedicellate, fine or thick setae of different sizes; trochanter trapezoidal, dorsal surface with some thickened, pedicellate setae near distal margin; anterior surface without protuberant area. Femur robust, closing surface with posteroventral row of processes composed two medially located, primary processes; proximal portion of the row with sub-basal secondary process and basal tertiary process; the rest of the row with abundant tubercle-shaped processes, and stinger-shaped setae; distal portion carinate, composed of tubercle-shaped specializations and stinger-shaped setae; adjacent row of thickened setae with globular base present on distal ¼. Anteroventral row of processes reduced to proximal ½; composed of tubercle-shaped specializations and stinger-shaped setae; the basal-most primary, curved process is present; distal portion composed of few tubercle-shaped processes; adjacent row of thickened setae with globular base present on distal ⅘. Tibia almost as long as femur, curved, ventral surface keeled with row of prostrate setae; patch of clavate setae apically on anterior surface present. Basitarsus with lanceolate process reaching base of fourth tarsomere; clavate setae present proximally on anterior surface; ventrally with single row of prostrate setae; second tarsomere about 7× as long as wide; third tarsomere as long as wide; fourth tarsomere ~ 3× as long as wide. ***Mid- and hind leg*.** Coxa and trochanter with thin setae, shorter on trochanter; femora with interspersed fine setae of different lengths and few thickened setae; tibiae mostly with short fine setae; hind leg longer than midleg, tibia 1.5× as long as femur; tarsomeres with distal margin of plantar surface with lateral groups of 5–8 thickened setae; on both legs, basitarsus 4× as long as wide, second tarsomere 1.2× as long as wide; third and fourth tarsomeres as long as wide; fifth tarsomere two times as long as wide. ***Wings*.** Forewing narrowly oval, trichosors present along margin except on wing base; costal space proximally expanded, humeral vein branched; 14–18 subcostal veinlets; pterostigma elongated, narrow, straight, with incomplete veinlets; subcostal space with single, medially located crossvein; Sc vein abruptly posteriorly bent at proximal pterostigma margin to merge with RA; radial space with two crossveins; *rarp2* gently curved with three or four RP branches; four veins arising from *rarp1*; M vein basally fused with RA; RP base widely separated from divergence of M and R; M forked opposite to RP origin; 1 r-m connecting RP base and M fork, forming a trapezoidal cell; 5–7 gradate crossveins present. Cubitus deeply forked; CuP basally angled and approaching A1, distally forked oppositely to level of separation of M and R; A1 apically forked, ending on posterior margin at level of CuP fork, A2 forked oppositely to CuP angle level. Hind wing smaller and narrower than forewing, narrowly oval; costal space narrow and reduced, with 6–8 veinlets; C and Sc fused at ¼ of wing length, Sc vein abruptly curved posteriad at proximal margin of pterostigma to merge RA; pterostigma elongated, narrow, gently curved, composed of incomplete veinlets; radial space with single crossvein, oblique; four veins arising from *rarp1*, one or two from *rarp2*. 1r-m sigmoid, connecting the stems of M and RP. Media forked beyond R fork. Cubitus deeply forked, intracubital crossvein subparallel to longitudinal wing axis; CuA sinuous, first branch candelabrum-shaped, spur vein absent or present; CuP strongly anteriorly bent at distal ½, pectinate; two crossveins on cubitoanal space; A1 apically simple, ending on wing margin at 1r-m stem level; A2 generally simple, short. ***Abdomen*.** Cylindrical to medially expanded, with subquadrate tergites; tergites III and IV with posteromedian concavity; tergites III-VII with elongated anterolateral scars; sternites rectangular.

**Figure 33.**
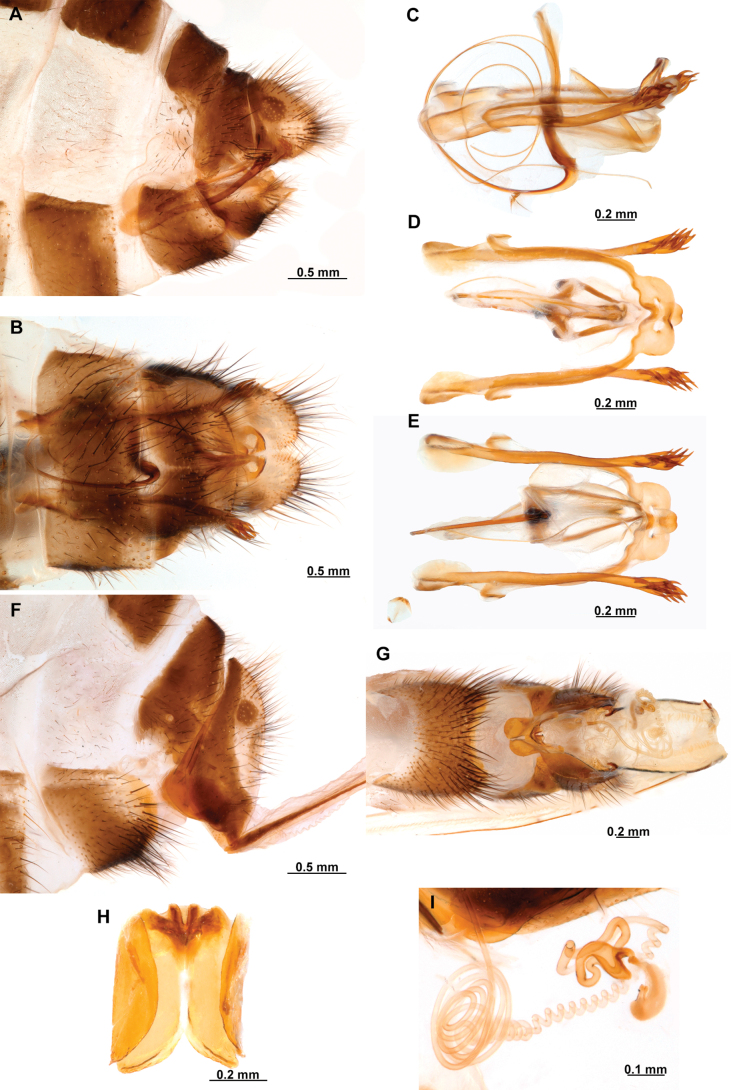
*Plegadactylota* Rehn, 1939 **A** male terminalia, lateral **B** same, ventral **C** male genitalia, lateral **D** same, dorsal **E** same, ventral **F** female terminalia, lateral **G** same, ventral **H** gonapophyses VIII of female, ventral **I** spermatheca.

***Male genitalia*** (Fig. 33A–E). Tergite IX medially narrower than laterally; lateral margin notched. Sternite VIII rectangular; sternite IX pentagonal in ventral view; posterior margin with short, medial, narrow lobe which is dorsally canaliculated; in lateral view triangular, apex not surpassing posterior margin of ectoproct. Gonocoxites IX elongate, thickened, sinusoid; base flattened, spatulated; apex branched, with 5–15 closely adpressed, twisted processes. Ectoproct ovoid, anteroventrally with rounded, flattened lobe that is continuous with ventromedial sclerotized, curved sulcus. Gonocoxites X forming a short, ventrally canaliculated sclerite, whose posterior apex has dorsal processes connected to gonostyli X and ventrolateral processes connected to gonapophyses X; gonostyli X with thickened and curved base, equipped with two lateral processes, the rest of the structure, ventrally curved, and anteriorly coiled, forming two loops before protruding from abdomen. Gonapophyses X long, straight, and narrow, with dorsally curved apexes; gonapophyses arranged in a V-shaped structure, joined by a membrane covering the gonostyli X base. Gonocoxites XI U-shaped, medial lobe complex and elaborated, with two differentiated parts: dorsal part as an expanded arch which has less sclerotized, transparent, medial area from which a ventrally incised process protrudes; ventral part as a curved, short lobe; lateral arms of gonocoxites XI straight, with anterior apex curved posteroventrally. Hypandrium arched and concave.

***Female genitalia*** (Fig. 33F–I). Sternite VII medially narrower than laterally, posterior margin concave, with long, thickened setae. Tergite VIII narrower medially than laterally, encircling the spiracle of the segment; lateral margin bluntly quadrangular. Gonocoxites + gonapophyses VIII medial part keel-shaped, posteromedially produced into a short bilobed process; lateral part an oval plate. Tergite IX + ectoproct ovoid. Gonocoxites IX elongated, sinuous, and narrow, as long as the last seven abdominal segments together. Bursa copulatrix short, conical, and moderately sclerotized. Spermatheca long, complex and entangled; proximal section thin, long, forming several coils; medial section as wide as proximal section, forming a long spiral; distal section forming several convolutions, distally widened, forming a diverticulum; fertilization canal duct proximally triangular, concave; fertilization canal elongated, curved, covered with microfilaments.

##### Distribution.

Mexico (Chihuahua, Coahuila, Guerrero, Jalisco, Morelos, Nayarit, Nuevo León, Puebla, Querétaro, Sinaloa, Sonora, Tamaulipas), USA (Arizona, Colorado?, New Mexico, Texas, Utah).

##### Remarks.

This species is widely distributed in Mexico and the United States. The records from Coahuila and Sinaloa were extracted from Oswald et al. (2002) and Reynoso-Velasco and Contreras-Ramos (2008). The records from Baja California Sur provided by these same authors are more likely erroneous and based on the material studied herein, such reports correspond to *P.flammata*. Here, this species is newly recorded for the Mexican states of Guerrero, Morelos, Nayarit, Nuevo Leon, Puebla, and Tamaulipas. Rehn (1939b) reported this species from Arizona (Cochise and Mohave Counties), Nevada (Charleston), Utah (Stockton and Duchesne) and Texas (Brewster and Jeff Davis Counties). The records from California provided by Penny et al. (1997) are likely doubtful and these probably represent specimens of *P.signata*. Here, this species is newly recorded from New Mexico.

This species is highly variable in coloration, size, and morphology. Rice (1987) studied the variation on the number of processes of the male gonocoxites IX, reporting from seven to twelve processes, leading to the synonymy of *Plegadactylotalipanica* Rehn. Based on the examination of the type specimen of *P.fratercula* and numerous specimens of *P.dactylota*, it was concluded the former represent a synonym of the latter. In the original description by Rehn (1939b), this species was separated from other North American species based on its body size and the presence of five processes on the gonocoxites IX. The overall arrangement of these processes was also used to distinguish this species, but this condition is noticeably similar to that expressed by *P.dactylota*. In this work, several small- and large sized specimens with only four processes were studied, leading to the conclusion that this character is quite plastic, and the variation is even broader to that reported by Rice (1987). Additional support for this synonymy is based on the morphology of the remaining genital sclerites of the male of *P.fratercula* being identical to those of *P.dactylota*. Finally, the distribution of *P.fratercula* from a single locality in Arizona is entirely overlapped with that of *P.dactylota* which is wide in this state.

#### ﻿﻿ Plega disrupta

Taxon classificationAnimaliaNeuropteraRhachiberothidae

Ardila-Camacho & Contreras-Ramos sp. nov.

https://zoobank.org/CB55652B-F1B9-4D25-8283-4154680EE542

Figs 34 , 35

##### Type locality.

Mexico, **Oaxaca**: Distrito de Ixtlán, Ixtlán de Juárez, Universidad de la Sierra Juárez, 17°18'55"N, 96°28'57.8"W, 1956 m, 20.X.2017, J.A. Casasola leg., “sobre pared de habitación”.

##### Material examined.

***Holotype*** male, pinned. Original label: “Mexico, Oaxaca, Distrito de Ixtlán, Ixtlán de Juárez, Universidad de la Sierra Juárez, 17°18'55"N, 96°28'57.8"W, 1956 m, 20.X.2017, J.A. Casasola, “sobre pared de habitación”, CNIN. ***Paratypes*.** Mexico • 1 ♀; same. data as for holotype; 12 Dec. 2016; CNIN.

##### Other material.

Mexico • 1 ♀; Oaxaca, Distrito de Ixtlán, Ixtlán de Juárez, Universidad de la Sierra Juárez; 17°18'55"N, 96°28'57.8"W; 1956 m; 16 Jan. 2017; J.A. Casasola leg.; “sobre pared de habitación”; CNIN. • 1 ♂; same data as for preceding; 12 Jan. 2011; CNIN.

##### Diagnosis.

This species is distinguished because of the presence of a brown flagellum with single preapical paler flagellomere, and the supra-antennal region slightly raised and covered with reclined setae. Furthermore, the antennal flagellomeres are as long as wide on the proximal part of the flagellum. The area adjacent to the posterior and wing margin presents intermittent amber infuscations, yet a pale area at wing tips is remarkable. The forefemur posterior surface is pale with brown dots, whereas the anterior surface is nearly completely brown. On the male genitalia, the sternite IX is trapezoidal with lateral indentation, and the gonocoxite IX is filiform, thin, and sinusoid, with the posterior apex pointed and devoid of processes. The gonocoxite XI medial lobe has a posteroventral U-shaped projection, with lateral rounded fins. The female genitalia have the gonapophyses VIII as two medially joined plates forming a tubular structure; posteromedially two lateral, digitiform lobes are present. The bursa copulatrix is long, with area adjacent to genital pore strongly sclerotized; the spermatheca has the distal section slightly expanded, wider than medial and proximal sections, but abruptly narrowed at apex.

##### Description.

***Measurements*.** Male (*n* = 1). Forewing length: 12 mm; Hind wing length: 9.5 mm. Female (*n* = 1): Forewing length: 12 mm; Hind wing length: 9.6 mm.

**Figure 34.**
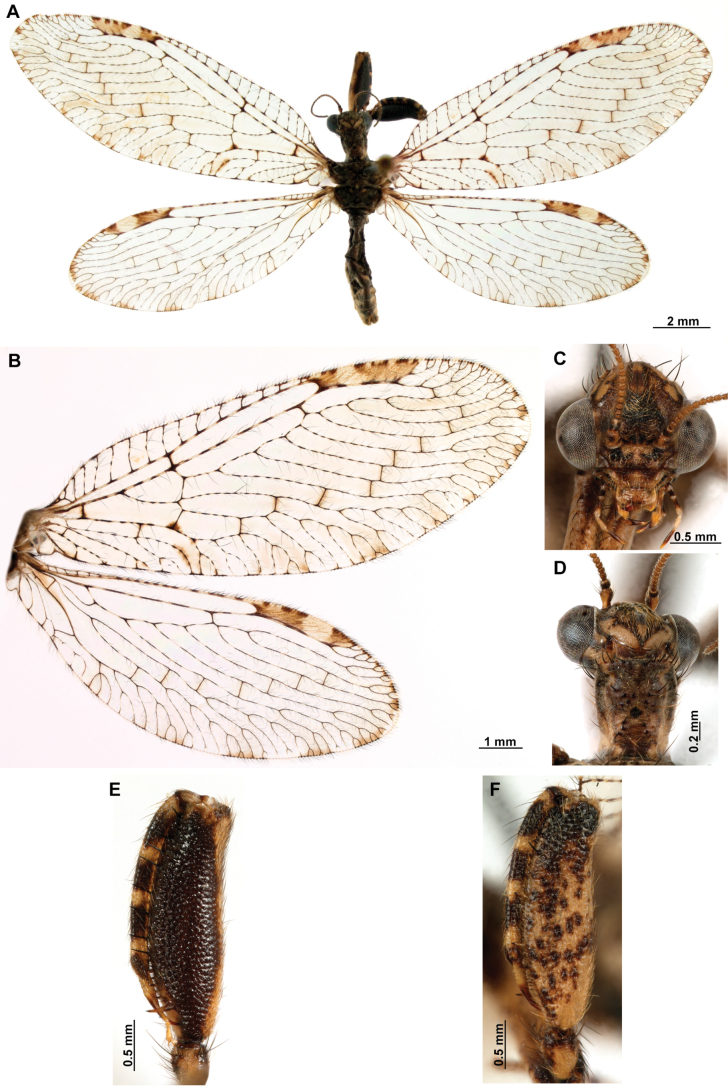
*Plegadisrupta* Ardila-Camacho & Contreras-Ramos, sp. nov. **A** male habitus, dorsal **B** wings **C** head, frontal **D** pronotum, dorsal **E** forefemur, anterior surface **F** same, posterior surface.

***Coloration*** (Fig. 34). ***Head*.** Vertexal region pale with V-shaped inverted mark extended from occiput to supra-antennal region, with pale brown setae. Occiput with dark brown marks near occipital transverse suture, setation pale brown; postgena pale with enlarged dark brown mark. Supra-antennal region mostly dark brown. Antennal scape pale with dark brown, dorsal, longitudinal band, and ventral mark, with dark brown near apex; pedicel dark brown, flagellum brown, with single paler preapical flagellomere. Frons mostly dark brown with medial area pale. Clypeus pale with lateral dark brown marks; labrum pale; mandible pale with dark brown dorsal and ventral corners, apex brown; maxilla pale with dark brown marks, palpus dark brown, except for pale fourth palpomere, and junctions of the remaining palpomeres, setae brown; labium pale with dark areas, setae brown; labial palpus dark brown, with pale junctions, palpimacula pale brown. ***Thorax*.** Pronotum mostly brown with anterolateral and posterolateral small, pale areas, and dark lateral margins; setae mostly dark brown and a few pale brown; episternum mostly dark brown with brown setae; postfurcasternum pale with lateral dark brown area. Mesonotum mostly dark brown with small pale areas, setation dark brown; metanotum dark brown with paler anteromedial area; pre-episternum dark brown mark; pteropleura dark brown with pale areas, with interspersed dark and pale brown setae. ***Foreleg*.** Coxa pale with basal and apical brown marks which are connected through brown suffusions on anterior and posterior surfaces; setal bases dark brown; setae mostly dark brown; trochanter pale with dark brown marks, interspersed pale and dark brown setae present. Femur posterior surface pale with dark brown dots, apex dark brown; anterior surface dark brown; dorsal surface with some suffusions connecting anterior and posterior maculations. Tibia dashed with pale and dark brown. Basitarsus with base and lanceolate process amber, proximal ½ with pale area, clavate setae pale brown; remaining tarsomeres pale with brown areas, setae brown; pretarsal claws pale amber. ***Mid- and hind leg*.** Coxae pale with brown areas, setation mostly pale brown; trochanter pale with dark brown marks. Femora and tibiae pale with dark brown rings. Tarsi pale, except the fifth tarsomere pale brown, setae dark brown; pretarsal claws pale amber. ***Wings*.** Forewing mostly hyaline or slightly amber shaded, dark amber on area surrounding crossveins, and intermittent amber areas on apical twigging near posterior and apical wing margin; major veins and subcostal veinlets alternating pale and dark brown, crossveins dark brown; pterostigma brown with pale, sub-basal area; wing margin alternating pale and brown. Hind wing mostly hyaline, with intermittent amber infuscation on the apical twigging of major veins near the posterior and distal margins; pterostigma dark brown with preapical pale area, and darker marks on anterior margin; longitudinal veins alternating pale and brown, except CuA mostly dark brown; subcostal veinlets and crossveins dark brown; wing margin alternating brown and pale. ***Abdomen*.** Tergites dark brown with pale lateral areas, setae mostly pale brown. Pleural membrane dark brown. Sternites pale with lateral, subtrapezoidal brown marks.

***Morphology* (Fig. 34)**. ***Head*.** Diamond-shaped in frontal view, rugose, vertexal region raised above compound eyes, with lateral rows of reclined setae; area adjacent to coronal suture and occipital ridge, glabrous, with muscle insertion mark; coronal suture distinct, ​​supra-antennal region slightly raised, covered with reclined setae; paraocular area concave. Antenna filiform, short; scape 1.5× as long as wide, slightly distally expanded, with short setae, a few longer and thicker near distal margin; pedicel as long as wide; flagellum not dorsoventrally flattened, with 30–33 or 41 flagellomeres, which are as wide as long on proximal part, and longer than wide on the rest of the flagellum; all articles with medial ring of short setae. Compound eye hemispherical, as wide as ¼ of the interocular distance at torulus level. Frons and clypeus rugose, with fine and short setae. Labrum pentagonal with fine and short setae; maxillary palpus with first palpomere as long as wide, second 1.5× as long as wide, third palpomere 3× as long as wide, fourth palpomere two times as long as wide, fifth palpomere slightly 1.5× as long as third, all the articles with short and thin setae; mentum with thin, long setae. Labial palpus with first palpomere two times as long as wide, second palpomere 4× as long as wide, third palpomere slightly longer than second, palpimacula ovoid. ***Thorax*.** Pronotum slightly longer than wide, with raised anterior margin, medial and posterior regions; outgrowths covered with pedicellate, thick setae, remaining surface with fine, short setae; postfurcasternum ventrally fused, collar-like. Mesonotum 2× as wide as long, with scattered, thick, pedicellate setae and some long, thin setae on medial area. Metanotum ~ 2× as long as wide, mostly glabrous with few fine setae on scutellum. Pteropleura with short, thin setae, and a few thick, longer setae. ***Foreleg*.** Coxa as long as femur, cylindrical, anterior and posterior surfaces with pedicellate, fine or thick setae of different lengths; trochanter subconical, with thin and short setae, except on dorsal and anterior surfaces with some pedicellate, thick, long setae. Femur robust, covered with abundant, fine, short setae which arise from protuberant bases on both surfaces. Closing surface with posteroventral row of processes composed two medially located primary processes and two sub-basal secondary process; between these processes a single tertiary process, and abundant tubercle-shaped processes and stinger-shaped setae are preset; distal portion of posteroventral row raised, composed of proximal tertiary process, tubercle-shaped specializations of different sizes and stinger-shaped setae; adjacent row of thickened setae with globular base present on distal ¼. Anteroventral row of processes reduced to proximal ½ and distal region; it is composed of tubercle-shaped specializations, stinger-shaped setae, and a basal primary, curved process; distal portion composed tubercle-shaped specializations; adjacent row of thickened setae with globular base present on distal ¾. Tibia almost as long as the femur, curved, setose, ventrally keeled with prostrate setae row; a patch of clavate setae is present apically on the anterior surface. Basitarsus with lanceolate process reaching the middle of fourth tarsomere; clavate setae are present proximally on anterior surface; ventrally with single row of prostrate setae; second tarsomere nearly 7× as long as wide; third tarsomere slightly longer than wide, fourth tarsomere 3× as long as wide. ***Mid- and hind leg*.** Coxae with thin, long setae; trochanter with fine and short setae; femora and tibiae with thin setae of different lengths; tibial spurs short; hind leg longer than midleg, tibia, 1.5× as long as femur; tarsi with thin, short setae, except on distal margin of plantar surface with lateral groups of 3–5 thickened setae; on both legs basitarsus 4× as long as wide, second tarsomere 1.5× as long as wide; third and fourth tarsomeres as long as wide; fifth tarsomere two times as long as wide. ***Wings*.** Forewing oval, trichosors present along margin except on wing base; venation setose; costal space proximally expanded, humeral vein branched, 14–17 subcostal veinlets, sometimes with a few apical forked; pterostigma elongated, trapezoidal, with well-defined, mostly forked, entire veinlets; subcostal space with single crossvein, medially located; Sc vein abruptly posteriorly curved at proximal pterostigma margin to merge with RA; radial space with two crossveins; *rarp2* curved with three RP branches arising from it; four veins arising from *rarp1*; M vein fused basally with RA; RP base widely separated from point of divergence of M from R; M forked opposite to R fork, 1 r-m connecting RP base and MA stem, forming a trapezoidal cell; six or seven gradate crossveins present. Cubitus deeply forked; CuP basally angled and approaching A1, distally forked at level of separation of M and R; A1 apically forked, ending on posterior margin at CuP fork level, A2 forked at CuP angle level. Hind wing smaller and narrower than forewing, narrowly oval; costal space narrow and reduced, with five or six veinlets; C and Sc fused at ¼ of wing length, Sc vein abruptly curved posteriorly at proximal margin of pterostigma to merge with RA; pterostigma elongated, narrow, composed of entire, mostly forked veinlets; radial space with single crossvein, oblique; three or four veins arising from *rarp1*, one or two from *rarp2*. 1r-m sigmoid, connecting the stems of M and RP. Media forked beyond R fork. Cubitus deeply forked, intracubital crossvein subparallel to longitudinal wing axis; CuA sinuous, first branch candelabrum-shaped, spur vein absent; CuP not touching A1, strongly anteriorly bent at distal 1/3, with three branches; two crossveins on cubitoanal space; A1 simple ending on wing margin slightly beyond 1m-cu level; A2 simple, short and curved. ***Abdomen*.** Cylindrical to medially expanded, setae on tergites gradually longer and more abundant towards terminal segments; tergites III–VI with elongated anterolateral scars. Sternites rectangular, with abundant, thin, long setae.

**Figure 35.**
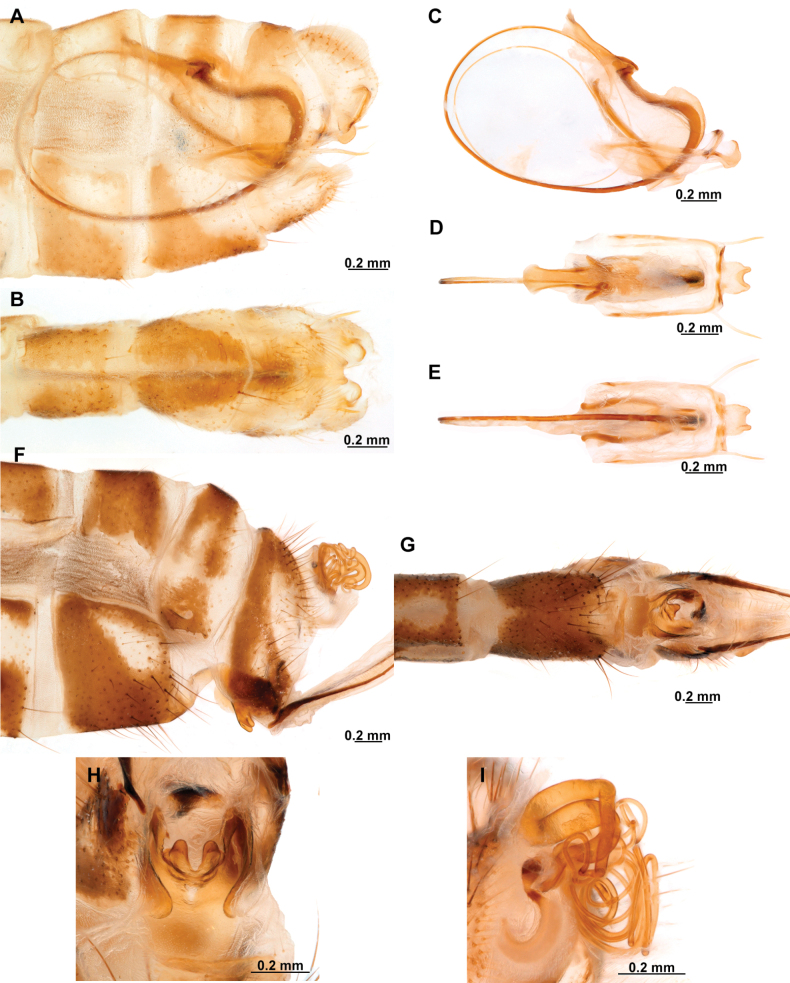
*Plegadisrupta* Ardila-Camacho & Contreras-Ramos, sp. nov. **A** male terminalia, lateral **B** same, ventral **C** male genitalia, lateral **D** same, dorsal **E** same, ventral **F** female terminalia, lateral **G** same, ventral **H** gonapophyses VIII of female, ventral **I** spermatheca.

***Male genitalia*** (Fig. 35A–E). Tergite IX medially narrower than laterally, lateral margin bluntly quadrangular. Sternum VIII rectangular, wider medially than laterally; sternite IX trapezoidal in ventral view, with lateral indentation, covered with abundant thin, short setae, posteromedially produced into a triangular lobe, which is equipped with dorsal canal; in lateral view trapezoidal, apex not reaching posterior margin of ectoproct. Gonocoxites IX filiform, thin, sinusoid; base connected to gonocoxites XI with a membrane; apex pointed, without processes. Ectoproct ovoid, with thin and short setae; ventral surface with anterior rounded lobe, which is continuous with sclerotized, incurved, ventromedial sulcus. Gonocoxites X forming a short, ventrally canaliculate sclerite, whose anterior apex is spatulated; posterior apex with dorsal processes connected to gonostyli X and ventrolateral processes connected to gonapophyses X with a membrane; gonostyli X with thickened, gently curved, with two lateral processes at base; the rest of the structure, whip-shaped, ventrally curved, and anteriorly recurved, forming two loops before protruding from abdomen. Gonapophyses X as two short, thin, rod-shaped, straight sclerites; the gonapophyses are joined by a membrane which is medially sclerotized and covers the gonostyli X base. Gonocoxites XI U-shaped, medial lobe complex and elaborated, with two differentiated parts: a dorsal arch and a posteroventral U-shaped projection, with lateral rounded fins; between these parts a narrow, arched, less sclerotized, hyaline area is present; lateral arms of gonocoxites XI thin, short, straight.

***Female genitalia*** (Fig. 35F–I). Sternum VII rectangular, posteromedially continuous with sclerotized, glabrous area, the rest of the surface with thin and short setae. Tergite VIII slightly narrower medially than laterally, encircling the spiracle of the segment, lateral margin bluntly quadrangular. Gonocoxites VIII as a rounded, concave plate. Gonapophyses VIII as two medially joined plates forming a tubular structure; posteromedially with two lateral, digitiform lobes, and medial U-shaped concavity; median part of gonapophyses VIII connected to narrowly, oval lateral plates, which are folded beneath tergite IX + ectoproct. Tergite IX + ectoproct narrowly ovoid. Gonocoxites IX elongated, sinuous, and narrow, as long as the last five abdominal segments combined. Bursa copulatrix long, funnel-shaped, membranous with area adjacent to genital pore strongly sclerotized. Spermatheca relatively long and complex; proximal section, long, thin, forming numerous coils; medial section thin, as wide as proximal section, forming numerous convolutions; distal section slightly expanded, wider than medial and proximal sections, but abruptly narrowed at apex; fertilization canal duct short, triangular, concave, with distal, thin portion; fertilization canal C-shaped, covered with microfilaments.

##### Etymology.

The name of this species comes from the Latin *disrumpere* which means disrupt(ed), alluding to the discontinuous or disruptive color pattern of the wings of this species. An adjective in the nominative case.

##### Distribution.

Mexico (Oaxaca).

##### Remarks.

This species is known only from the type locality in Oaxaca, Mexico. This is an anomalous species which was recovered in a clade with *P.yucatanae*, *P.hagenella*, and other South American species. The genitalia of both sexes of this species are remarkable, as the male has the gonocoxites XI similar to those of *Trichoscelia*, and the gonocoxites IX are devoid of processes. The female spermatheca is markedly different to other species of the genus, the gonocoxites + gonapophyses VIII have certain similarities with other South America species, for instance *P.zikani*.

#### ﻿﻿ Plega drepanicoides

Taxon classificationAnimaliaNeuropteraRhachiberothidae

Ardila-Camacho & Contreras-Ramos sp. nov.

https://zoobank.org/D9A820B0-7736-4BE0-82DC-2D6F362FD9AB

Figs 36 , 37

##### Type locality.

Mexico, **Jalisco**: Sierra Manatlán, Puerto Los Mazos, 1500 m, 3–7 Aug. 1992, Luz Hg, Encinar, D. Curoe leg.

##### Material examined.

***Holotype*** male, pinned. Original label: “Mexico, Jalisco, Sierra Manatlán, Puerto Los Mazos, 1500 m, 3–7 Aug. 1992, D. Curoe leg., Luz Hg, Encinar”, CAS. ***Paratypes*.** Mexico – **Chihuahua** • 1 ♂; Rta. San Rafael-Cuiteco; 27°28.130'N, 107°56.580'W; 1870 m; 30 Aug. 2005; J. Bueno, R. Barba leg.; CNIN. – **Jalisco** • 1 ♂; San Sebastián del Oeste; 20°46'5.04"N, 104°50'27.67"W; 1620 m, 29.VII.2019, A. Ramírez-Ponce Leg., light trap, mixto de pino-encino 2743 (CNIN).

##### Other material.

Mexico • 1 ?; **Jalisco**, Sierra Manatlán, Puerto Los Mazos; 1500 m; 3–7 Aug. 1992; D. Curoe leg.; Luz Hg, Encinar; CAS. • 1 ♂; Jalisco, Los Mazos; 15 Jul. 1989; R.J. McGinley leg.; USNM. • 1 ♀; Jalisco, La Yerbabuena; 1950 m; 04 Aug. 1994; G. Nogueira; CNIN. • 1 ♂; Volcán de Tequila; 2150 m; 10–11 Aug. 1994; G. Nogueira; CNIN. • 1 ♀; San Sebastián del Oeste; 20°46'5.04"N, 104°50'27.67"W; 1620 m; 29 Jul. 2019; A. Ramírez-Ponce leg.; light trap, mixto de pino-encino; CNIN 2739. • 1 ♂; same data as for preceding; CNIN 2741. • 1 ♂; same data as for preceding; CNIN 2742. • 1 ♀; same data as for preceding; CNIN 2740. • 1 ♂; same data as for preceding; CNIN 2744. • 1 ♂; Volcán de Tequila, 3.7 km S Hwy 15d; 20°49'27"N, 103°51'25"W; 1849 m; 09 Jul. 2017; E. Martínez leg.; CSCA.

##### Diagnosis.

This species presents the antennal flagellum completely brown, and the basal flagellomeres are as wide as long. The ​​supra-antennal region is raised and the area adjacent to frontal sutures is sunken; both areas are covered with reclined setae. The forefemur has the posterior surface pale with discontinuous brown spots on proximal and distal ½; the anterior surface is mainly dark brown with small pale areas. The forewing has the membrane on apex of intracubital area strongly marked. On the male genitalia, the sternite IX is U-shaped; the gonocoxite IX is short, strongly laterally arched, set with three digitiform, apical processes, subequal in shape and size, two of which are dorsally situated; the surface of such processes has scattered, minute spinules. The gonostyli X are anteriorly recurved, forming a loop before protruding from the abdomen. On the female terminalia, the sternum VII appears as two medially joined, trapezoidal plates (gonocoxites VII), with fusion line discernible. The gonapophyses VIII medial part consist of two medially joined, trapezoidal plates forming a keel, which is set with posteromedial bilobed process. The proximal section of the spermatheca is spiral-shaped, thin, with intermittent swellings.

##### Etymology.

The specific epithet of this species is a composite of the mantispid genus name *Drepanicus* and –*oides*, used to refer to a species that resembles other species. This name alludes to the superficial resemblance of this new species with some South American species of Drepanicinae.

##### Description.

***Measurements*.** Male (*n* = 4). Forewing length: 11.9–15.4 mm; Hind wing length: 9.9–13.2 mm.

**Figure 36.**
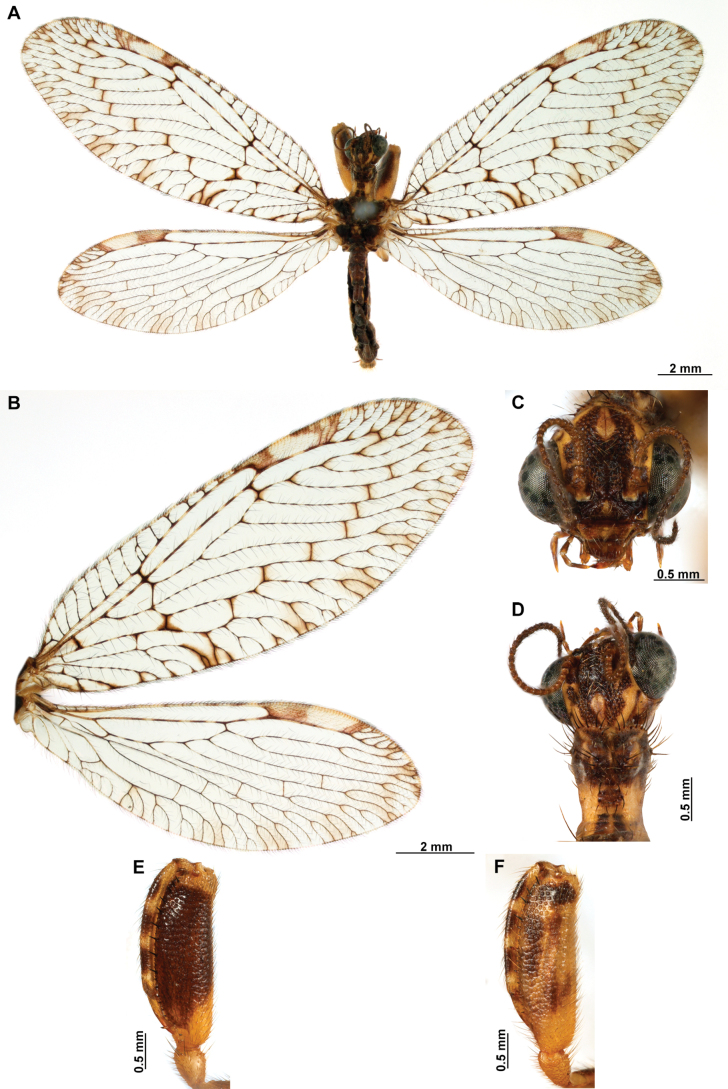
*Plegadrepanicoides* Ardila-Camacho & Contreras-Ramos, sp. nov. **A** male habitus, dorsal **B** wings **C** head, frontal **D** pronotum, dorsal **E** forefemur, anterior surface **F** same, posterior surface.

***Coloration*** (Fig. 36). ***Head*.** Vertexal region pale with dark brown paired irregular markings extending from occiput to toruli, with interspersed pale and dark brown setae. Occiput with dark brown marks near occipital transverse suture, setation brown; postgena pale. Antennal scape pale with dark brown longitudinal ventral and dorsolateral bands, distal margin dark brown, setae dark brown; pedicel dark brown, flagellum brown. Frons pale with dark brown lateral markings which merge anteromedially. Clypeus pale with lateral dark brown markings; labrum pale with anterior ½ brown; mandible pale with dorsal and ventral corners, plus apex dark brown; maxilla pale with dark brown markings, palpus dark brown, except for pale junctions between segments, setae brown; labium pale with pale brown areas, setae dark brown on anterior portion of submentum and anterior area of prementum; labial palpus dark brown, third palpomere paler, palpimacula pale brown. ***Thorax*.** Pronotum pale with dark brown lateral stripes, medial area with irregular markings, setae mostly dark brown and a few pale brown; episternum mostly brown with interspersed yellow, pale- and dark brown setae; postfurcasternum pale. Mesonotum mostly dark brown with small anterolateral and medial pale areas, setation dark brown; metanotum dark brown with pale area on medial region of scutum; pre-episternum pale with dark brown mark; pteropleura pale with brown markings. ***Foreleg*.** Coxa pale with basal and apical brown markings which are connected through brown suffusions; trochanter pale with dark brown marking on anterior surface, setae of dorsal surface dark brown. Femur posterior surface pale with discontinuous brown spots on proximal and distal ½, apex dark brown; anterior surface mainly dark brown with small pale areas, setal bases bordered with dark brown forming a honey-comb pattern. Tibia dashed with pale and dark brown. Basitarsus with base and lanceolate process amber, proximal ½ with pale area, clavate setae pale brown; second tarsomere brown, third and fourth paler with brown setae. ***Mid- and hind leg*.** Coxae pale with small brown areas, pale brown setae present; trochanter pale with dark brown spots. Femora and tibiae pale with brown spots, hind leg with apex of femur and base of tibia dark brown, interspersed pale and dark brown setae present; tibial spurs amber. Basitarsus pale, remaining tarsomeres pale brown, setae brown; distal margins on plantar surface of the first four tarsomeres with dark brown setae; pretarsal claws amber. ***Wings*.** Forewing mostly hyaline, dark brown on area surrounding crossveins, M stem and fork, RP stem and stem of its branches, and intermittently on apical twigging near posterior and apical wing margin; membrane on apex of intracubital area strongly marked; major veins and subcostal veinlets alternating pale and dark brown, crossveins dark brown; pterostigma brown with medial pale area; wing margin alternating pale and brown. Hind wing mostly hyaline, with intermittent amber infuscation on the apical twigging of major veins near the posterior and distal margins; pterostigma dark brown with preapical pale area; C, Sc, and R alternating pale and brown, the remainder longitudinal veins, mostly brown; crossveins dark brown; wing margin alternating brown and pale. ***Abdomen*.** Tergites mostly brown, pale near the lateral margins, setae mostly pale brown. Some specimens with tergites completely brown; pleural membrane mostly brown, with pale, marginal areas. Sternites pale with lateral, subtrapezoidal brown marks.

***Morphology*** (Fig. 36). ***Head*.** Diamond-shaped in frontal view, rugose, vertexal region raised above compound eyes, with lateral rows of reclined setae; area adjacent to coronal suture and occipital ridge, glabrous, with muscle insertion mark; coronal suture distinct; area adjacent to frontal sutures sunken, ​​supra-antennal region raised, both areas covered with reclined setae; paraocular area concave. Antenna filiform, short, scape 1.5× as long as wide, slightly distally expanded, with short setae, a few longer and thicker on ventral surface; pedicel as long as wide; flagellum slightly dorsoventrally flattened, with 40 or 41 flagellomeres, which are as wide as long; all articles with medial ring of short setae. Compound eye hemispherical, as wide as ½ of the interocular distance at torulus level. Frons and clypeus rugose, with fine and short setae. Labrum pentagonal with fine and long setae; maxillary palpus with first palpomere as long as wide, second 1.5× as long as wide, third palpomere 3× as long as wide, fourth palpomeres two times as long as wide, fifth palpomere slightly 1.5× as long as third, with short and thin setae; mentum with thin, long setae. Labial palpus with first palpomere 1.5× as long as wide, second palpomere 4.5 times as long as wide, third palpomere slightly longer than second, palpimacula narrowly ovoid. ***Thorax*.** Pronotum slightly longer than wide, with raised anterior margin, medial and posterior regions; outgrowths covered with pedicellate, thick setae, remaining surface with fine, short setae; postfurcasternum ventrally fused, collar-like. Mesonotum 2× as wide as long, with scattered, thick, pedicellate setae and some long, thin setae on medial area. Metanotum ~ 2× as long as wide, mostly glabrous with few fine setae on scutellum. Pteropleura with short, thin setae; a few thick setae on mesanepisternum. ***Foreleg*.** Coxa as long as femur, cylindrical, anterior and posterior surfaces with pedicellate, fine or thick setae of different lengths; trochanter subconical, with thin and short setae, except dorsally with some thick and longer setae, anterior surface with protuberant area covered with pedicellate, thick setae. Femur robust, covered with abundant, fine, short setae which arise from protuberant bases on both surfaces. Closing surface with posteroventral row of processes composed two medially located primary processes and a sub-basal secondary process; between these processes a single tertiary process, and abundant tubercle-shaped processes and stinger-shaped setae are preset; distal portion of posteroventral row raised, composed of tubercle-shaped specializations of different sizes and stinger-shaped setae; adjacent row of thickened setae with globular base present on distal ¼. Anteroventral row of processes reduced to proximal ½ and distal region; it is composed of tubercle-shaped specializations, stinger-shaped setae, and a basal primary, curved process; distal portion composed tubercle-shaped specializations; adjacent row of thickened setae with globular base present on distal ½. Tibia almost as long as the femur, curved, setose, ventrally keeled with prostrate setae row; a patch of clavate setae is present apically on the anterior surface. Basitarsus with lanceolate process reaching the middle of fourth tarsomere; clavate setae are present proximally on anterior surface; ventrally with single row of prostrate setae; second tarsomere nearly 7× as long as wide; third tarsomere 1.5× as long as wide, fourth tarsomere two times as long as wide. ***Mid- and hind leg*.** Coxae with short, thin setae; trochanter on both legs with fine and short setae; femora and tibiae with fine, short setae; tibial spurs short; hind leg longer than midleg, tibia, 1.5× as long as femur; tarsi with fine and short setae, except on distal margin of plantar surface with lateral groups of 5–7 thickened setae; basitarsus 3.5× as long as wide, second tarsomere 1.5× as long as wide; third and fourth tarsomeres as long as wide; fifth tarsomere two times as long as wide. ***Wings*.** Forewing narrowly oval, trichosors present along margin except on proximal ½ of wing; venation setose; costal space proximally expanded, humeral vein branched, 12–14 subcostal veinlets; pterostigma elongated, curved, with incomplete, mostly simple veinlets; subcostal space with single crossvein, medially located; Sc vein abruptly posteriorly curved at proximal pterostigma margin to merge with RA; radial space with two crossveins; *rarp2* curved with three or four RP branches arising from it; three veins arising from *rarp1*; M vein fused basally with RA; RP base widely separated from point of divergence of M from R; M forked slightly before RP base, 1 r-m connecting RP base and MP stem, forming a trapezoidal cell; five or six gradate crossveins present. Cubitus deeply forked; CuP basally angled and approaching A1, distally forked at level of separation of M and R; A1 apically forked, ending on posterior margin at CuP fork level, A2 forked at CuP angle level. Hind wing smaller and narrower than forewing, narrowly oval; costal space narrow and reduced, with 4–6 veinlets; C and Sc fused at ¼ of wing length, Sc vein abruptly curved posteriorly at proximal margin of pterostigma to merge with RA; pterostigma elongated, curved, narrow, composed of incomplete, mostly simple veinlets; radial space with single crossvein, oblique; three or four veins arising from *rarp1*, 1–3 from *rarp2*. 1r-m sigmoid, connecting the stems of M and RP. Media forked beyond R fork. Cubitus deeply forked, intracubital crossvein subparallel to longitudinal wing axis; CuA sinuous, first branch candelabrum-shaped, spur vein absent; CuP not touching A1, strongly anteriorly bent at distal 1/3, with three branches; two crossveins on cubitoanal space; A1 simple ending on wing margin slightly beyond 1m-cu stem level; A2 simple, short and curved. ***Abdomen*.** Cylindrical to medially expanded, setae on tergites gradually longer and more abundant towards terminal segments; tergites III-VII with elongated anterolateral scars. Sternites with abundant, thin, short setae; sternites III-VII with lateral scars near the posterior margin.

**Figure 37.**
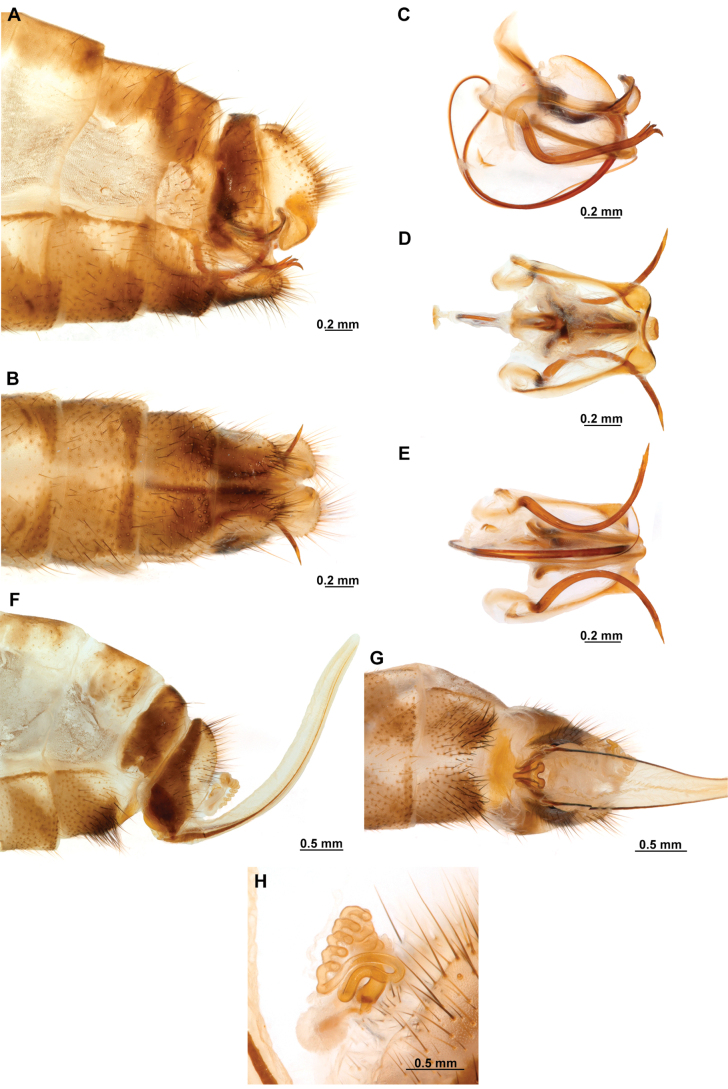
*Plegadrepanicoides* Ardila-Camacho & Contreras-Ramos, sp. nov. **A** male terminalia, lateral **B** same, ventral **C** male genitalia, lateral **D** same, dorsal **E** same, ventral **F** female terminalia, lateral **G** same, ventral **H** spermatheca.

***Male genitalia*** (Fig. 37A–E). Tergite IX medially narrower than laterally, ventrally bluntly quadrangular. Sternum VIII rectangular; sternite IX U-shaped in ventral view, covered with abundant fine and long setae, posteromedially produced into a blunt lobe, which is equipped dorsal canal; in lateral view short and blunt, the apex reaching posterior margin of ectoproct. Gonocoxites IX thickened, short, sinuous; base spatulate, connected to gonocoxites XI with a membrane; the rest of the structure strongly laterally arched, with three digitiform, apical processes, subequal in shape and size, two of which are dorsally situated; the surface of the digitiform processes is set with, minute, scattered spinules. Ectoproct ovoid, covered with abundant, thin setae; ventral surface concave, forming a broad, rounded lobe, entire surface covered with microtrichia; ventromedially the lobe is continued by a curved, sclerotized canal which led to concave plate. Gonocoxites X forming a short, thickened, ventrally canaliculate sclerite, whose anterior apex is expanded; posterior apex with dorsal processes connected to gonostyli X and ventrolateral processes connected to gonapophyses X with a membrane; gonostyli X with thickened, obtuse base, with two lateral processes, the rest of the structure, ribbon-shaped, ventrally curved, and anteriorly recurved, forming a loop before protruding from the abdomen. Gonapophyses X as two short, thin, rod-shaped, straight sclerites arranged in a V-shaped structure; the gonapophyses are joined by a membrane which is medially sclerotized and covers the gonostyli X base. Gonocoxites XI U-shaped, medial lobe complex and elaborated, with two differentiated parts: a dorsal arch and a posteroventral convex area, which is covered with microspinules and medially incised; between these parts a narrow, arched, less sclerotized, hyaline area is present; lateral arms of gonocoxites XI short, sinuous with anterior apex strongly ventrally incurved.

***Female genitalia*** (Fig. 37F–H). Sternum VII as two medially joined, trapezoidal plates (gonocoxites VII), the fusion line discernible, area adjacent to posterior margin with abundant thick and long setae. Tergite VIII slightly narrower medially than laterally, encircling the spiracle of the segment, lateral margin bluntly quadrangular. Gonocoxites VIII as a trapezoidal concave plate. Gonapophyses VIII as two medially joined trapezoidal plates which form a keel; posteromedial margin of this keel bilobed, with medial U-shaped concavity; median par of gonapophyses VIII connected to lateral rounded plates, which are folded beneath tergite IX + ectoproct. Tergite IX + ectoproct narrowly ovoid. Gonocoxites IX elongated, sinuous, and narrow, as long as the last four abdominal segments combined. Bursa copulatrix long, siphon-shaped, semi-membranous, striated. Spermatheca relatively simple; proximal section, spiral-shaped, thin, with intermittent swellings; medial section short, as wide as proximal section, without swellings; distal section slightly expanded, with short and blunt diverticulum; fertilization canal duct short, triangular, concave, with distal, thin portion; fertilization canal J-shaped, covered with microfilaments.

##### Distribution.

Mexico (Chihuahua, Jalisco).

##### Remarks.

This species is known only from the Mexican states of Chihuahua and Jalisco, being probably endemic to the country. This is an anomalous species recovered as intermediary between a clade containing species from South and Mesoamerica and the rest of the species of *Plega*. In the male genitalia, the morphology of the male gonocoxites XI is similar to that of *Trichoscelia*. Furthermore, this species is the unique species of Symphrasinae that has the female gonocoxites VII as paired lateral plates as expressed by several species of *Anchieta* and Rhachiberothinae.

#### ﻿﻿ Plega duckei

Taxon classificationAnimaliaNeuropteraRhachiberothidae

Penny, 1982

Figs 38 , 39

Plega duckei Penny, 1982a: 424. Holotype: male, Brazil, Amazonas (INPA), photographs examined.

##### Material examined.

***Holotype*.** Brazil • 1 ♂; **Amazonas**, Reserva Ducke; 11 Oct. 1977; J.R. Arias leg.; INPA. ***Paratypes***. Brazil • 1 ♂; **Amazonas**, Manaus, Parque das Laranjeiras; 22 Jan. 1981; J.R. Arias leg.; “Paratype, *Plegaduckei* Penny”; USNMENT01541899; USNM.

##### Other material.

Brazil • 1 ♀; Amazonas, Manaus; 22 Oct. 1991; G.A.R. Melo leg.; DZUP. • 1 ♀; same data as for preceding; 17 Aug. 1991; DZUP.

##### Diagnosis.

This species has most of the vertexal region, frons, and clypeus brown. The antennal flagellum has subconical flagellomeres, those of proximal ¼ are 3× as wide as long, and the palpimacula is grooved. The forefemur is remarkably narrow. On the male genitalia, the sternite IX is U-shaped; the gonocoxite IX is short, thin, and straight, with posterior apex set with three or four short processes. The gonostyli X are ribbon-shaped, short, and recurved at middle before protruding from the abdomen.

##### Description.

***Measurements*.** Male (*n* = 1). Forewing length: 8.5 mm; Hind wing length: 6.2 mm.

**Figure 38.**
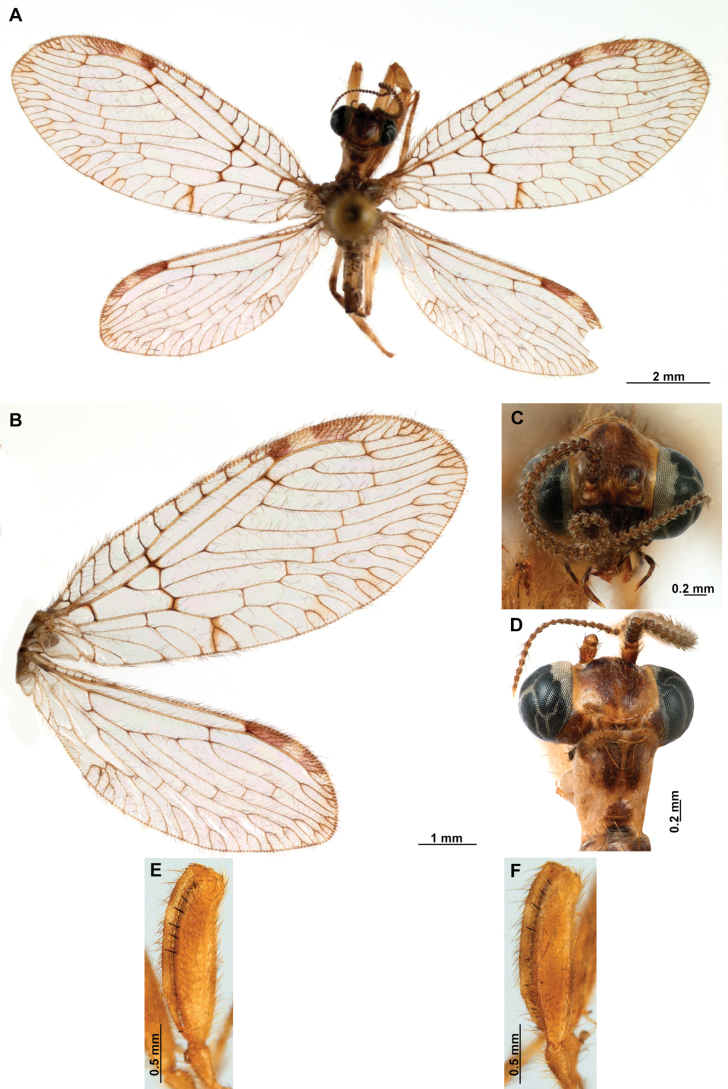
*Plegaduckei* Penny, 1982 **A** male habitus, dorsal **B** wings **C** head, frontal **D** pronotum, dorsal **E** forefemur, anterior surface **F** same, posterior surface.

***Coloration*** (Fig. 38). ***Head*.** Vertexal yellow, with broad medial brown area, extended from occiput to supraantennal region, with pale brown setae; area adjacent to coronal suture paler; supra-antennal area darker with medial yellowish area; occiput and postgena pale brown. Antenna brown, with dark brown setae. Frons dark brown, clypeus brown, labrum pale brown; mandible pale brown, with ventral corner dark brown, amber at apex; maxilla with cardo and stipes yellow, galea brown, palpus dark brown; labium with postmentum yellow, pale brown ligula, palpus dark brown. ***Thorax*.** Pronotum yellow with brown marks on anterior ½ and posteromedian region; episternum yellow; postfurcasternum pale. Mesonotum with brown marks on sclerites on scutum, pale on area adjacent to sutures; scutellum pale with brown mark on the center; setation mostly pale brown. Metanotum with lateral brown areas and pale medial area; pre-episternum brown; pteropleura brown with small pale areas, setation mostly pale brown. ***Foreleg*.** Coxa yellowish with brownish apex; trochanter brownish, setation pale brown. Femur yellowish with pale brown areas; setae mostly pale brown. Tibia dashed with yellowish and pale brown areas, setae pale brown. Basitarsus with yellowish proximal ½ and pale brown lanceolate process; remaining tarsomeres pale brown. ***Mid- and hind leg*.** Coxae brown, with pale brown setae; trochanter of both legs yellowish. Femur and tibia of mid-leg alternating yellowing and pale brown, with pale brown setae present. Hind leg with femur yellowish with pale brown apex, setae mostly yellow, tibia alternating pale brown and yellowish, setation mostly pale brown. Tarsi pale brown; first four tarsomeres with brown setae, laterally on distal margin of plantar surface; remainder surface with pale brown setae; pretarsal claws pale brown. ***Wings*.** Forewing mostly hyaline; membrane surrounding crossveins on subcostal and radial spaces, RP stem, first two crossveins of mediocubital space and first branch of CuA amber; with intermittent amber areas between apical twigging on distal ½ of wing near posterior and apical wing margins. Pterostigma brown with yellow medial area. Major veins alternating yellowish and brown, CuP and anal veins mostly yellow; subcostal veinlets and crossveins brown; wing margin alternating pale yellowish and brown. Hind wing hyaline, amber on area adjacent to stem first branch of CuA; pterostigma brown with yellowish preapical area; longitudinal veins alternating pale yellowish and brown; subcostal veinlets pale brown; crossveins brown, 1r-m bicolor; wing margin alternating pale yellowish and brown. ***Abdomen*.** Cleared.

***Morphology* (Fig. 38). *Head*.** Diamond-shaped in frontal view, rugose; vertexal region raised above compound eyes, with lateral rows of reclined setae; area surrounding coronal suture glabrous, with muscle insertion mark; coronal suture distinct; medial area of ​​supra-antennal region slightly raised, with fine, reclined setae; paraocular area concave. Antenna moniliform, long; scape and pedicel as long as wide, both with fine, short setae; flagellum not dorsoventrally flattened, with 44–52 flagellomeres, subconical, progressively narrowed towards the apex, those of proximal ¼ of the flagellum widened; all articles with medial ring of fine, setae. Compound eye hemispherical, as wide as ¾ of the interocular distance at torulus level. Frons rectangular, with fine and short setae, clypeus trapezoidal, narrow with long, thin setae. Labrum pentagonal with thin, short setae; maxillary palpus with first palpomere as long as wide, second 1.2× as long as wide, third palpomere 3× as long as wide, fourth palpomere 2.5× as long as wide, fifth palpomere as long as third, all with short and thin setae; mentum with long, thin setae; labial palpus with first palpomere 2× as long as wide, second palpomere 3.2× as long as wide, third palpomere as long as second, palpimacula grooved. ***Thorax*.** Pronotum slightly longer than wide, with slightly raised anterior margin, medial and posterior regions; these areas covered with elongated, thickened setae; remaining surface with fine, short setae. Mesonotum wider than long, with abundant, thick, setae on medial area. Metanotum ~ 2× as wide as long, scutellum with few, fine setae. Pteropleura with short, thin setae. ***Foreleg*.** Coxa slightly shorter than femur, cylindrical, anterior and posterior surfaces abundant, thin setae; trochanter narrow, with thin and short setae, except on dorsal surface with some pedicellate, long setae near distal margin; anterior surface without protuberant area covered with pedicellate, long setae. Femur long and narrow, covered with abundant, fine, long setae; closing surface with posteroventral row of processes composed two medially located, primary processes; proximal portion of the row with sub-basal tertiary process; the rest of the row with abundant tubercle-shaped processes, and stinger-shaped setae; distal portion not raised composed of tubercle-shaped specializations and stinger-shaped setae; adjacent row of thickened setae with globular base present on distal 2/5. Anteroventral row of processes reduced to proximal ½; it is composed of tubercle-shaped specializations and stinger-shaped setae; the basal-most primary, curved process is present; distal portion composed of a few tubercle-shaped processes; adjacent row of thickened setae with globular base present on distal ½. Tibia almost as long as femur, curved, with thin, short setae; ventral surface keeled with prostrate setae; a patch of clavate setae apically on anterior surface is present. Basitarsus with lanceolate process reaching the base of fourth tarsomere; clavate setae present proximally on anterior surface; ventrally with single row of prostrate setae; second tarsomere nearly 7× as long as wide; third tarsomere 1.5× as long as wide, fourth tarsomere 2× as long as wide. ***Mid- and hind leg*.** Coxae and trochanter with thin setae, shorter on trochanter; femora with interspersed fine setae of different lengths; hind leg longer than midleg, tibia 1.5× as long as femur, slightly expanded and flattened; tibial spurs short; tarsi with fine and short setae, except on distal margin of plantar surface with lateral groups of 1–3 thickened setae; on both legs, basitarsus 4× as long as wide, second tarsomere 1.5× as long as wide; third and fourth tarsomeres as long as wide; fifth tarsomere 2× as long as wide. ***Wings*.** Forewing oval, trichosors present along margin except on wing base; venation setose; costal space proximally slightly expanded, humeral vein branched, 11 or 12 subcostal veinlets; pterostigma elongated, narrow, gently curved, with incomplete veinlets; subcostal space with single crossvein, medially located; Sc vein abruptly posteriorly bent at proximal pterostigma margin to merge with RA; radial space with two crossveins; *rarp2* gently curved with one or two RP branches; three or four veins arising from *rarp1*; M vein basally fused with RA; RP base close to divergence of M and R; M forked opposite to RP origin, 1 r-m connecting RP base and M fork, forming a trapezoidal cell; three or four gradate crossveins present. Cubitus deeply forked; CuP basally angled and approaching A1, distally forked opposite to the level of separation of M and R; A1 simple, ending on posterior margin at level of CuP fork, A2 forked opposite to CuP angle level. Hind wing smaller and narrower than forewing, narrowly oval; costal space narrow and reduced, with five veinlets; C and Sc fused at ¼ of wing length, Sc vein abruptly curved posteriad at proximal margin of pterostigma to merge RA; pterostigma elongated, narrow, gently curved, composed of entire veinlets; radial space with single crossvein, oblique; three veins arising from *rarp1*, one or no veins arising from *rarp2*. 1r-m sigmoid, connecting the stems of M and RP. Media forked beyond R fork. Cubitus deeply forked, intracubital crossvein subparallel to longitudinal wing axis; CuA sinuous, first branch candelabrum-shaped, spur vein present; CuP not touching A1, anteriorly bent at distal ½, pectinate; two crossveins on cubitoanal space; A1 simple, ending on wing margin at 1r-m stem level; A2 simple, short, and curved. ***Abdomen*.** Cylindrical, setae on tergites, scattered, thin, and short; tergites subquadrate. Sternites rectangular, with abundant fine and short setae, longer near the posterior margin of each sternite.

**Figure 39.**
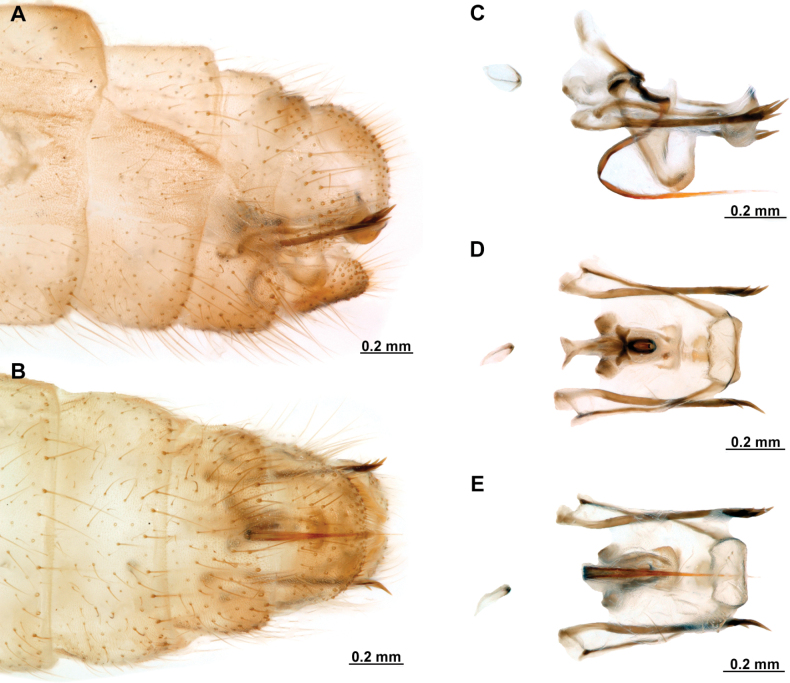
*Plegaduckei* Penny, 1982 **A** male terminalia, lateral **B** same, ventral **C** male genitalia, lateral **D** same, dorsal **E** same, ventral.

***Male genitalia*** (Fig. 39). Tergite IX medially narrower than laterally; lateral margin rounded. Sternum VIII rectangular; sternite IX U-shaped in ventral view, with short and thin setae; posterior margin with medial, small, blunt lobe which is dorsally canaliculated; in lateral view bluntly trapezoidal, apex reaching posterior margin of ectoproct. Gonocoxites IX short, thin, straight; base flattened, spatulated; apex branched, with three or four short processes. Ectoproct ovoid with short, thin setae, ventrally with broad, rounded, sclerotized lobe, which is continuous with ventromedial, curved sulcus. Gonocoxites X forming a short, ventrally canaliculated sclerite, whose anterior apex is expanded; posterior apex has dorsal processes connected to gonostyli X and ventrolateral processes connected to gonapophyses X with a membrane; gonostyli X with thickened, straight base, equipped two lateral processes, the rest of the structure ribbon-shaped, short, recurved at middle before protruding from abdomen. Gonapophyses X short, thin, straight, with anterior apex slightly expanded, posterior apex dorsally bent; gonapophyses subparallel, joined by a membrane covering the gonostyli X base. Gonocoxites XI U-shaped, medial lobe complex and elaborated, with two differentiated parts: dorsal part as a thin arch; ventral part forming a concave covering; between these parts a broad, circular, less sclerotized, hyaline area is present; lateral arms of gonocoxites XI short, thin, with anterior gently curved. Hypandrium internum tiara-shaped.

##### Distribution.

Brazil (Amazonas).

##### Remarks.

This species was described from Amazonas, Brazil and is known solely from its type locality and adjacent areas (Penny 1982a). *Plegaduckei* was recovered within the clade of South American species as sister of *P.paraensis*. This is an anomalous species having enlarged compound eyes, discoidal basal, antennal flagellomeres, sulcate palpimacula, and narrow and elongate forefemur. The male genitalia are markedly distinct from other species of the genus with simple gonocoxites XI and short and recurved gonostyli X. The female genitalia of this species remain unknown.

#### ﻿﻿ Plega flammata

Taxon classificationAnimaliaNeuropteraRhachiberothidae

Ardila-Camacho & Contreras-Ramos sp. nov.

https://zoobank.org/547B37B9-5EE5-4304-B72C-B226756BABDC

Figs 40 , 41

##### Type locality.

Mexico, **Baja California Sur**: San Venancio, 08 Oct. 1941; Rosa & Bohart leg.

##### Material examined.

***Holotype*** male, pinned. Original label: “Mexico, **Baja California Sur**, San Venancio, 08 Oct. 1941, Rosa & Bohart leg.”, CAS. ***Paratypes*.** Mexico • 1 ♀; **Baja California Sur**, 9 mi. N. Cabo San Lucas; 09 Sep. 1988; E.G. Riley leg.; blacklight; TAMU-ENTOX0278312; TAMUIC. • 1 ♂; same data as for preceding; TAMU-ENTOX0278371; TAMUIC. • 1 ♂; same data as for preceding; TAMU-ENTOX0278336; TAMUIC. • 1 ♂; 3 mi. N. San Antonio; 9–10 Oct. 1968; El. 1200’; E.L. Sleeper & F.J. Moore leg.; *Plegadactylota*, det. N. Penny, 1998; CAS. • 1 ♂; Sa. Victoria, Casas Viejas; El. 800’; 27–28 Oct. 1968; E.L. Sleeper & F.J. Moore leg.; collected at blacklite; *Plegadactylota* det. N. Penny, 1998; CAS. • 1 ♂; La Paz, Todos Santos, Km 5 a la Burrera; 23°25'48"N, 110°10'31"W; 145 m; 30 Sep. 2010; C. Mayorga & L. Cervantes leg.; matorral xerófilo, nocturnal collection; CNIN. • 1 ♂; Isla Cerralvo, punta NW; 18 Aug. 1986; F. Arias leg.; CNIN.

##### Other material.

Mexico – **Baja California** • 1 ♂; San Lorenzo [Island]; N. Banks leg.; MCZ-ENT00681782; MCZ. – **Baja California Sur** • 1 ♂; Sa. Victoria, Casas Viejas; El. 800’; 27–28 Oct. 1968; E.L. Sleeper & F.J. Moore leg.; collected at blacklite; *Plegadactylota* det. N. Penny, 1998; CAS. • 2 ♂; 1 mi. SW. Sta. Catarina; 29–30 Jun. 1967; E.L. Sleeper & E.M. Fisher leg.; collected at blacklite; *Plegadactylota*, det. N. Penny, 1998; CAS. • 1 ♂; 2.6 miles E. of San Antonio; 30 Jul. 1971, H.G. Real & R.E. Main leg.; U.V. light; CAS. • 1 ♂; 3 mi. N. San Antonio; 9–10 Oct. 1968; El. 1200’; E.L. Sleeper & F.J. Moore leg.; *Plegadactylota*, det. N. Penny, 1998; CAS. • 1 ♂; Boca de la Sierra; 27–28 Jun. 1967; E.L. Sleeper & E.M. Fisher leg.; collected at blacklite; *Plegadactylota*, det. N. Penny, 1998; CAS. • 2 ♂; 4 Km E. El Triunfo (Km 160); 560 m; 05 Sep. 1977; E. Fisher & R. Westcott leg.; CAS. • 1 ♂; 1 mi. East of Migriño; 19 Jul. 1971; H.G. Real & R.E. Main leg.; U.V. light; CAS. •1 ♂; 1 mi. East of Migriño; 17 Jul. 1971; H.G. Real leg.; at U.V. light; CAS. • 1 ♂; 6 mi. E. San José del Cabo; El. 200’; 26–27 Oct. 1968; E. L. Sleeper & F.J. Moore leg.; collected at blacklite; *Plegadactylota*, det. N. Penny, 1998; CAS. • 2 ♂; Boca de la Sierra; 27–28 Jun. 1967; E.L. Sleeper & E.M. Fisher leg.; collected at blacklite; *Plegadactylota*, det. N. Penny, 1998; CAS. • 2 ♂ 2 ♀; 9 mi SW Hwy 256 on rd to San Isidro; 24°0'55"N, 110°8'0"W; 700 m; 14–15 Jul. 1999; R. Aalbu, K. Brown, I. Stahl & F. Piñero leg.; Blacklite; *Plegadactylota*, det. N. Penny, 1999; #D00750-1502; CAS. • 1 ♀; San Isidro; 24°00.24'N, 110°09.32'W; 533 m; 15 Aug. 2004; D. Robacker, D. Thomas & J. Burne leg.; TAMU-ENTOX0755721; TAMUIC. • 1 ♂ 1 ♀; 8 mi. SE La Paz; El. 1000’; 13 Oct. 1968; E.L. Sleeper & F.J. Moore leg.; collected at blacklite; *Plegadactylota*, det. N. Penny, 1998; CAS. • 1 ♂; 22 mi. W. La Paz; El. 1000’; 07 Oct. 1968; E.L. Sleeper & F.J. Moore leg.; *Plegadactylota*, det. N. Penny, 1998; CAS. • 1 ♂; 9 mi. N San Lucas; El. 1000’; 24–25 Oct. 1968; E.L. Sleeper & F.J. Moore leg.; collected at blacklite; *Plegadactylota*, det. N. Penny, 1998; CAS. • 1 ♂; 6 mi. NE San Lucas; El. 100’; 25 Oct. 1968; E.L. Sleeper & F.J. Moore leg.; collected at blacklite; *Plegadactylota*, det. N. Penny, 1998; CAS. • 1 ♂ 1 ♀; Baja California Sur, 6 mi. E. San José del Cabo; El. 200’; 26–27 Oct. 1968; E.L. Sleeper & F.J. Moore leg.; collected at blacklite; *Plegadactylota*, det. N. Penny, 1998; CAS. • 1 ♀; 12 mi. SW. S. José del Cabo; 03 Jul. 1963; E.M. Fisher leg.; *Plegadactylota*, det. N. Penny, 1998; CAS. • 4 ♂ 16 ♀; Los Cabos, Sierra de la Laguna, Rancho Ecológico Sol de Mayo, Cabañas; 23°29.899'N, 109°47.395'W; 282 m; 15 Aug. 2021; Contreras, Cancino, Luna, Martins & Marquez leg.; light trap; CNIN. •14 ♂; same data as for preceding; 14 Aug. 2021; CNIN. • 10 ♂ 11 ♀; same data as for preceding; Cabañas; 14 Aug. 2021; trampa McPhail/Melaza; CNIN. • 31 ♂ 11 ♀; same data as for preceding; cabaña hacia la cascada; 23°29.890'N, 108°47.415'W; 238 m; 13 Aug. 2021; CNIN. • 4 ♂; same data as for preceding; Vereda arriba cascada en cañón de la zorra; 23°29.910'N, 109°47.720'W; 271 m; 14 Aug. 2021; Luna, Contreras, Barba & Ramírez leg.; light trap; CNIN. • 1 ♂ 3 ♀; same data as for preceding; Vereda arriba cascada en cañón de la zorra; 23°29.910'N, 109°47.720'W; 271 m; 15 Aug. 2021; light trap; CNIN. • 1 ♂ 1 ♀; Valle Perdido, 2 mi N Reserva gate; 23°21'49"N, 110°1'12"W; 500 m; 16–17 Jul. 1999; R. Aalbu, K. Brown, I. Stahl & F. Piñero leg.; *Plegadactylota*, det. N. Penny, 1999; D00750-1502; CAS. 1 ♀; Sa. Victoria, El Chorro; El. 1000’; 29–30 Oct. 1968; E.L. Sleeper & F.J. Moore leg.; collected at blacklite; *Plegadactylota*, det. N. Penny, 1998; CAS. 1 ♀; Todos Santos; 18 Oct. 1941; Ross & Bohart leg.; CAS. • 1 ♀; 13 mi E. hwy 19, E. of Todos Santos; 17–18 Sep. 1988; E. Riley; TAMU-ENTOX0278512; TAMUIC. • 1 ♂; La Paz, Todos Santos, Km 5 a la Burrera; 23°25'48"N, 110°10'31"W; 145 m; 30 Sep. 2010; C. Mayorga & L. Cervantes leg.; matorral xerófilo, nocturnal collection; CNIN. • 6 ♂ 1 ♀; La Paz, 7 mi. SW; 06 Aug. 1966; E. & J. Linsley, J. Chemsak, P.D. Hurd leg.; at light; CSCA. • 1 ♀; Km 119 La Paz-San José del Cabo; 13 Sep. 1977; S. Lorna & G. Gutz leg.; CNIN. • 1 ♂; La Paz-San Antonio, “Microondas”; 23°47'24"N, 110°04'35"W; 677 m; 28 Sep. 2010; C. Mayorga & L. Cervantes leg.; matorral xerófilo, nocturnal collection; CNIN. • 2 ♀; 3 mi. N. San Antonio; 9–10 Oct. 1968; El. 1200’; E.L. Sleeper & F.J. Moore leg.; collected at blacklite; *Plegafratercula*, det. N. Penny, 1998; CAS. • 1 ♀; 3 Km E. La Burrera; 515 m; 02 Sep. 1977; E. Fisher & R. Westcott leg.; CAS. • 1 ♂; 8.4 mi. W. on Ramal a Los Naranjos; 13 Sep. 1988; E.G. Riley leg.; TAMU-ENTOX0278442; TAMUIC. • 1 ♂; same data as for preceding; TAMU-ENTOX0278450; TAMUIC. • 1 ♀; 8.4 mi W on Ramal a Los Naranjos; 13 Sep. 1988; A.J. Gilbert leg.; CSCA. • 1 ♂; 9.4 mi. W. Hwy 1 on Ramal a San Felipe; 10–11 Sep. 1988; E. Riley; TAMU-ENTOX0278355; TAMUIC. • 1 ♂; same data as for preceding; TAMU-ENTOX0278379; TAMUIC. • 1 ♂; same data as for preceding; TAMU-ENTOX0278329; TAMUIC. • 1 ♀; same data as for preceding; TAMU-ENTOX0278349; TAMUIC. • 4 ♀; Isla Cerralvo, punta NW; 18 Aug. 1986; F. Arias leg.; CNIN. • 1 ♀; same data as for preceding; Isla Cerralvo; 04 Aug. 1986; CNIN. • 1 ♀; same data as for preceding; L. Cervantes leg.; CNIN. • 1 ♂ 2 ♀; same data as for preceding; 04 Aug. 1986; L. Cervantes leg.; CNIN. • 1 ♀; Isla San José, punta NW; 17 Aug. 1986; F. Arias; CNIN.

##### Diagnosis.

This species has the antennal flagellum with basal flagellomeres 2× as long as wide. The posterior surface of the forefemur has three brown weakly to well defined spots on the medial area; the anterior surface is dark brown, except the base pale. On the male genitalia, the gonocoxite IX is long, thin, and sigmoid, with posterior apex thickened, laterally curved, and set with 8–13 processes arranged in a group. The ventral part of the median lobe of the gonocoxites XI forms a prominent, curved process with trapezoidal apex, whose surface is rugose and has microspinulae. On the female genitalia, the medial part of the gonapophyses VIII is boat-shaped, lacking processes.

##### Etymology.

The specific name of this species comes from the Latin *flammare*, which means flame. This alludes to the flame-shaped apex of the male gonocoxite IX of this species.

##### Description.

***Measurements*.** Male (*n* = 7). Forewing length: 11.3–14.6 mm; Hind wing length: 8.8–11.3 mm. Female (*n* = 2): Forewing length: 13.2–14.2 mm; Hind wing length: 10.4–11.3 mm.

**Figure 40.**
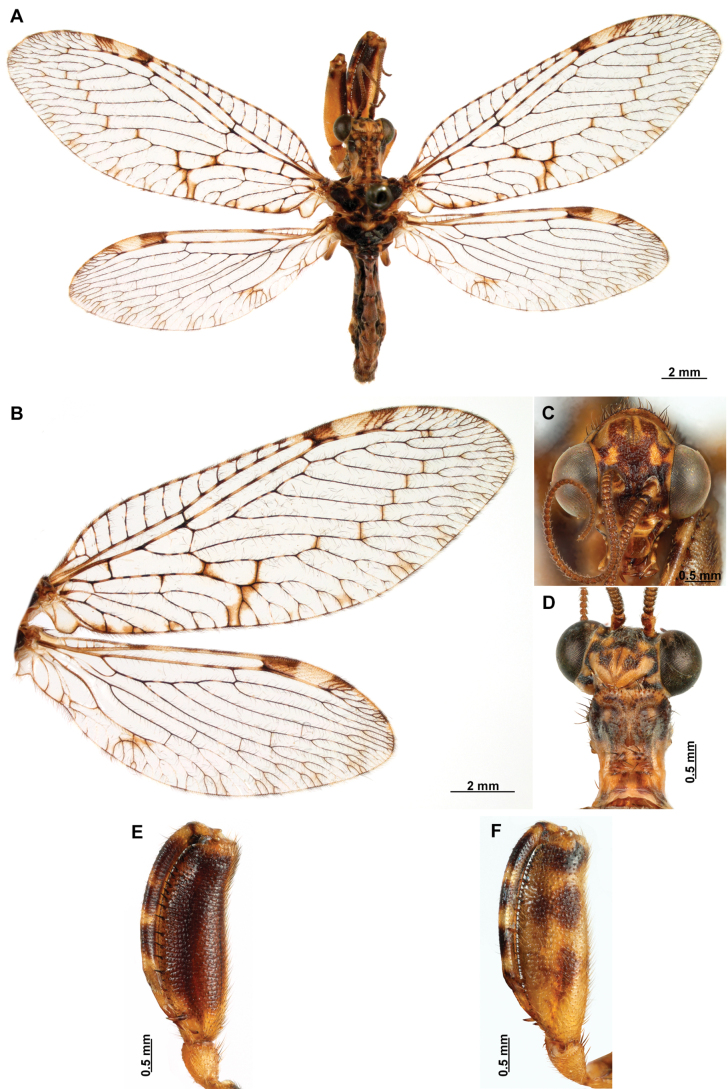
*Plegaflammata* Ardila-Camacho & Contreras-Ramos, sp. nov. **A** male habitus, dorsal **B** wings **C** head, frontal **D** pronotum, dorsal **E** forefemur, anterior surface **F** same, posterior surface.\

***Coloration* (Fig. 40)**. ***Head*.** Vertexal region pale, with lateral dark brown markings extending from occiput to toruli forming a V-shaped pattern, mainly with pale brown setae; area adjacent to occipital ridge dark brown; paraocular area pale with brown areas; supra-antennal area dark brown, with pale brown setae; occiput pale with brown spot, postgena pale sometimes with brown spot. Antennal scape pale, with brown dorsal longitudinal band, entire surface with pale brown setae, and some dark brown setae; pedicel brown, flagellum pale brown with brown setae. Frons either completely brown with or brown with inverted, triangular, pale, medial mark. Clypeus either pale brown or pale with pale brown lateral areas; labrum pale brown; mandible pale with dark brown corners, dark amber at apex; maxilla pale with pale brown areas, palpus brown; labium pale with pale brown areas, setae pale brown; labial palpus brown, pale at junctions, palpimacula pale brown. ***Thorax*.** Pronotum pale with irregular brown markings, anterolateral corners with darker spot; episternum brown, sometimes with pale medial area, postfurcasternum pale. Mesonotum with scutum mainly dark brown, except pale on area surrounding sutures, scutellum pale with median and posterolateral dark brown areas, setation mostly dark brown; metanotum mostly dark brown; pre-episternum pale to dark brown; pteropleura mostly dark brown with pale on margins of sclerites, setation pale brown. ***Foreleg*.** Coxa mostly pale, with brown spots on base and apex, interspersed pale and dark brown setae present; trochanter pale, setation pale brown, except dorsally with some dark brown setae. Femur posterior surface pale with three weakly to well defined brown spots on medial area, apex brown; anterior surface dark brown, except at base, and area surrounding the primary process. Tibia dashed with pale and dark brown. Basitarsus pale brown proximally, changing to amber towards the apex, clavate setae pale brown; remaining tarsomeres pale brown. ***Mid- and hind leg*.** Coxae pale, sometimes with brown areas, setae pale brown; trochanter pale. Femur and tibia of mid-leg dashed with pale and brown, on hind leg with brown at apex of femur and base and apex of tibia. Tarsi pale to pale brown; distal margins on ventral surface with dark brown setae; pretarsal claws brown. ***Wings*.** Forewing mostly hyaline; membrane surrounding crossveins, first branch of CuA and apex of CuP amber; posterior and apical margins on distal ½ of wing with intermittent, amber areas between apical branches of longitudinal veins; pterostigma brown with pale medial area; major veins, subcostal veinlets, and wing margin alternating pale and brown. Hind wing hyaline, amber on area adjacent to first branch of CuA; pterostigma brown with pale preapical area; C, subcostal veinlets and Sc with extensive pale areas, remainder longitudinal veins alternating pale and brown; crossveins brown, except sigmoid 1r-m, bicolor; wing margin alternating pale and brown. ***Abdomen*.** Tergites pale brown with pale areas, and lateral darker marks; setation mostly pale. Pleural membrane mostly dark brown. Sternites pale with pale brown lateral areas; setation mostly pale.

***Morphology*** (Fig. 40). ***Head*.** Diamond-shaped in frontal view, rugose; vertexal region raised above compound eyes, with lateral rows of reclined setae; area surrounding coronal suture glabrous, with muscle insertion mark; coronal suture distinct; medial area of ​​supra-antennal region not raised, with fine, reclined setae; paraocular area concave. Antenna submoniliform, short; scape 1.5× as long as wide, slightly distally expanded, with fine, short setae; pedicel as long as wide; flagellum dorsoventrally slightly flattened, with 50–60 flagellomeres, which are 2× as long as wide on proximal ¼, and as long as wide on the rest of flagellum; all articles with medial ring of fine, short setae. Compound eye hemispherical, as wide as ½ of the interocular distance at torulus level. Frons and clypeus narrow, with fine and short setae. Labrum pentagonal with thin, short setae; maxillary palpus with first palpomere as long as wide, second 1.5× as long as wide, third palpomere 3× as long as wide, fourth palpomeres 2.5× as long as wide, fifth as long as third, all with short and thin setae; mentum with long, thin setae; labial palpus with first palpomere 2.5× as long as wide, second palpomere 4× as long as wide, third palpomere slightly longer than second, palpimacula narrowly ovoid. ***Thorax*.** Pronotum as long as wide, with raised anterior margin, medial and posterior regions; outgrowths covered with pedicellate, thick setae; remaining surface with fine, short setae. Mesonotum 2× as wide as long, with scattered, thick, pedicellate setae on medial area. Metanotum ~ 2× as long as wide, glabrous. Pteropleura with short, thin setae. ***Foreleg*.** Coxa as long as femur, cylindrical, anterior and posterior surfaces with pedicellate fine or thick setae of different sizes; trochanter trapezoidal, with thin and short setae, except on dorsal and anterior surfaces with some thickened setae; anterior surface with protuberant area. Femur robust, covered with abundant, fine, short setae; closing surface with posteroventral row of processes composed two medially located, primary processes, sometimes with tertiary process on the middle; proximal portion of the row with two secondary processes; the rest of the row with abundant tubercle-shaped processes, stinger-shaped setae, and some tertiary processes; distal portion raised composed of tubercle-shaped specializations and stinger-shaped setae; adjacent row of thickened setae with globular base present on distal ¼. Anteroventral row of processes reduced to proximal ½; it is composed of tubercle-shaped specializations and stinger-shaped setae; the basal-most primary, curved process is present; distal portion composed of a few tubercle-shaped processes; adjacent row of thickened setae with globular base present on distal ⅔. Tibia almost as long as femur, curved, with thin, short setae; ventral surface keeled with prostrate setae; a patch of clavate setae apically on anterior surface is present. Basitarsus with lanceolate process reaching the middle of fourth tarsomere; clavate setae present proximally on anterior surface; ventrally with single row of prostrate setae; second tarsomere nearly 6× as long as wide; third tarsomere as long as wide, fourth tarsomere 2× as long as wide. ***Mid- and hind leg*.** Coxae and trochanter with short, thin setae; femora and tibiae with interspersed fine, short setae and a few thickened setae; tibial spurs short; hind leg longer than midleg, tibia, 1.5× as long as femur; tarsi with fine and short setae, except on distal margin of plantar surface with lateral groups of 5–7 thickened setae; on both legs, basitarsus 3.5× as long as wide, second tarsomere 1.2× as long as wide; third and fourth tarsomeres as long as wide; fifth tarsomere 2× as long as wide. ***Wings*.** Forewing narrowly oval, trichosors present along margin except on wing base; venation setose; costal space proximally expanded, humeral vein branched, 9–15 subcostal veinlets; pterostigma elongated, narrow, straight, with distinct veinlets; subcostal space with single crossvein, medially located; Sc vein abruptly posteriorly bent at proximal pterostigma margin to merge with RA; radial space with two crossveins; *rarp2* gently curved with three or four RP branches; three veins arising from *rarp1*; M vein basally fused with RA; RP base, close to divergence of M and R; M forked nearly opposite to RP origin, 1 r-m connecting RP base and M, forming a trapezoidal cell; 3–5 gradate crossveins present. Cubitus deeply forked; CuP basally angled and approaching A1, distally forked slightly beyond the level of separation of M and R; A1 apically forked, ending on posterior margin at level of CuP fork, A2 forked slightly beyond CuP angle level. Hind wing smaller and narrower than forewing, narrowly oval; costal space narrow and reduced, with 5–7 veinlets; C and Sc fused at ¼ of wing length, Sc vein abruptly curved posteriorly at proximal margin of pterostigma to merge RA; pterostigma elongated, narrow, straight, composed of poorly defined veinlets; radial space with single crossvein, oblique; three or four veins arising from *rarp1*, one from *rarp2*. 1r-m sigmoid, connecting the stems of M and RP. Media forked beyond R fork. Cubitus deeply forked, intracubital crossvein subparallel to longitudinal wing axis; CuA sinuous, first branch candelabrum-shaped, spur vein absent or present; CuP not touching A1, strongly anteriorly bent at distal 1/3, pectinate; two crossveins on cubitoanal space; A1 simple ending on wing margin at 1m-cu level; A2 simple, short, and curved. ***Abdomen*.** Cylindrical to medially expanded, setae on tergites, scattered, thin, and short, gradually longer and more abundant towards terminal segments; tergites subquadrate, tergites III-V with elongated anterolateral scars. Sternites rectangular, with abundant fine and short setae.

**Figure 41.**
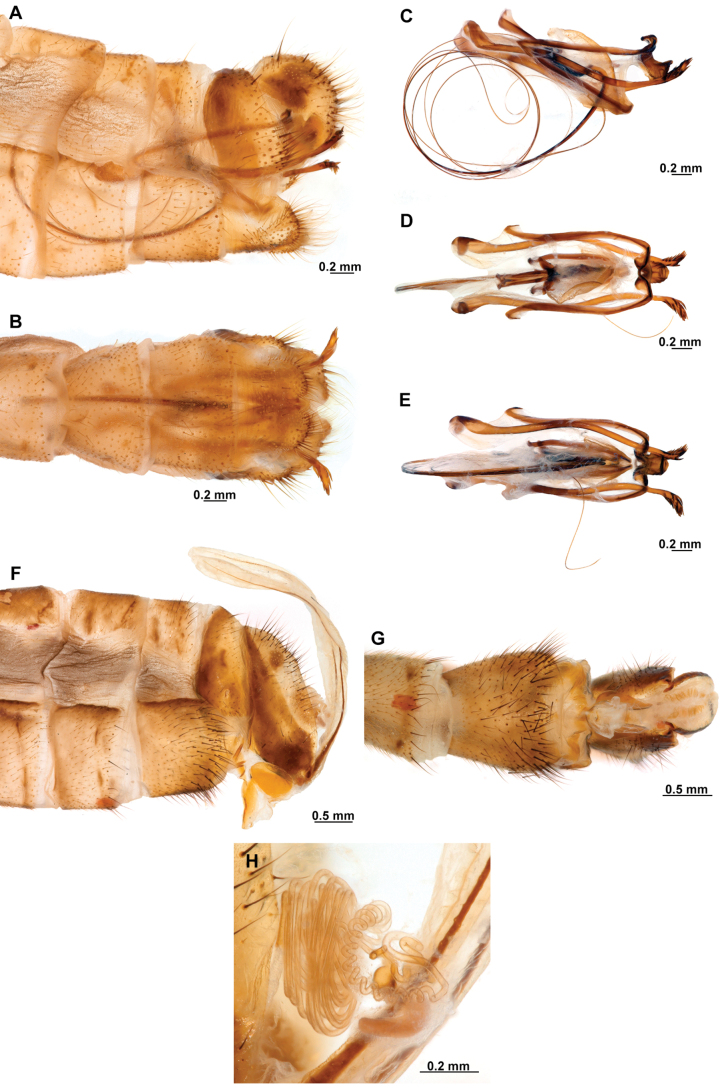
*Plegaflammata* Ardila-Camacho & Contreras-Ramos, sp. nov. **A** male terminalia, lateral **B** same, ventral **C** male genitalia, lateral **D** same, dorsal **E** same, ventral **F** female terminalia, lateral **G** same, ventral **H** spermatheca.

***Male genitalia*** (Fig. 41A–E). Tergite IX medially narrower than laterally; lateral margin quadrangular, posterolateral corner with thickened setae. Sternum VIII quadrangular; sternite IX trapezoidal in ventral view, with rounded posterolateral lobes covered with abundant, long setae; posterior margin with short, medial, blunt lobe, which is dorsally canaliculated; in lateral view blunt, apex not reaching posterior margin of ectoproct. Gonocoxites IX thin, sigmoid, long; base flattened, dorsally bent, connected to gonocoxites XI with a membrane; apex thickened, laterally curved, with 8–13 processes arranged in a bunch. Ectoproct ovoid, covered with abundant, thickened setae on posterior surface, posteroventrally with a convex area covered with thin, pedicellate setae; anteroventrally with a flattened, rounded lobe which is continuous with ventromedial sclerotized, curved sulcus. Gonocoxites X forming a short, thickened, ventrally canaliculate sclerite, with anterior apex expanded, posterior apex with dorsal processes connected to gonostyli X and ventrolateral processes; gonostyli X with thickened and straight base with two lateral processes, the rest of the structure, whip-shaped, ventrally curved, and anteriorly coiled, forming two loops before protruding from abdomen. Gonapophyses X rod-shaped, straight, narrow, arranged in a V-shaped structure; gonapophyses joined by a membrane covering the gonostyli X base; this membrane is medially sclerotized and laterally covered with microspinulae. Gonocoxites XI thin, U-shaped, medial lobe complex and elaborated with two differentiated parts: a dorsal, narrow lobe with medial U-shaped concavity; ventral part as a prominent, curved lobe with trapezoidal apex whose surface appears rugose and with microspinulae; between these parts a small, less sclerotized, hyaline area is present; lateral arms of gonocoxites XI straight with anterior apex incurved. Hypandrium internum tiara-shaped.

***Female genitalia*** (Fig. 41F–H). Sternum VII rectangular with posterior margin concave, setose. Tergite VIII slightly narrower medially than laterally, encircling the spiracle of the segment; lateral margin triangular. Gonocoxites VIII as a narrow sclerite, medially concave. Gonapophyses VIII with medial part boat-shaped, without processes; laterally folded beneath IX + ectoproct, an oval plate is connected to medial part of gonapophyses VIII. Tergite IX-+-ectoproct triangular. Gonocoxites IX elongated, sinuous, and narrow. Bursa copulatrix long, narrow, semi-membranous and striated, except near genital pore, sclerotized. Spermatheca complex and entangled, proximal section, thin, long, forming numerous coils, terminating on a strongly entangled portion; medial section as wide as proximal section, forming a long spiral; distal section as wide as medial section, terminating in an expanded portion, where a blunt diverticulum is present; fertilization canal duct proximally subconical, concave; fertilization canal J-shaped, covered with microfilaments.

##### Distribution.

Mexico (Baja California, Baja California Sur).

##### Remarks.

This species is restricted to the Peninsula of Baja California (Baja California and Baja California Sur) in Mexico. It was recovered within the clade of species of Central Mexico and the Nearctic as sister to *Plegasignata* to which it is noticeably similar. The anterior surface of forefemur with pale base and the numerous short processes on the male gonocoxites IX arranged as a flame easily separate *P.flammata* from *P.signata*. The females may be difficult to separate as there are no clear differences on the genitalia, however they can be separated based on the general color pattern.

#### ﻿﻿ Plega fumosa

Taxon classificationAnimaliaNeuropteraRhachiberothidae

Linsley & MacSwain, 1955

Figs 42 , 43

Plega fumosa Linsley & MacSwain, 1955: 16. Holotype: male, Mexico, Michoacán (CAS), specimen examined.

##### Material examined.

***Holotype*.** Mexico • ♂; **Michoacán**, 11 mi E. Apatzingán; 20 Aug. 1954; E.G. Linsley, J.W. MacSwain, R.F. Smith leg.; Type No. 7202; CAS. ***Paratypes*.** Mexico • 1 ♂; **Michoacán**, 11 mi. E. Apatzingán; 20 Aug. 1954; E.G. Linsley, J.W. MacSwain, R.F. Smith leg.; “Paratype of *Plegafumosa* Linsley & MacSwain”; FSCA. • 2 ♀; same data as for preceding; FSCA. • 1 ♀; 11 mi. E. Apatzingán; 20 Aug. 1954; E.G. Linsley, J.W. Mac Swain, R.F. Smith leg.; “Paratype *Plegafumosa* Linsley & MacSwain”; CAS. • 2 ♀; 11 mi. E. Apatzingán; 20 Aug. 1954; E.G. Linsley, J.W. MacSwain, R.F. Smith leg.; MCZ Paratype 29540, “Paratype *Plegafumosa* Linsley & MacSwain” MCZ-ENT 00029540; MCZ.

##### Other material.

Mexico • 1 ♂; **Michoacán**; 2000 ft.; 13 Aug. 1941; H. Hoogstraal leg.; FSCA. • 1 ♂; Apatzingán; Alt. 1200 ft.; 13 Aug. 1941; Hoogstraal & Haag leg.; *Plegadactylota* Rehn det. N. Banks; FSCA. • 1 ♂; same data as for preceding; “Gift of the Hoogstraal Mexican Biological Expeditions”; FSCA. • 1 ♂; same data as for preceding; H. Hoogstraal leg.; FSCA. • 2 ♀; Apatzingán; Alt. 1200 ft.; 13 Aug. 1941; Hoogstraal & Haag leg.; *Plegafumosa* Linsley & MacSwain, 1955, det. R.G. Beard, 1968; MCZ. • 1 ♀; Km. 169 Uruapan, Playa Azul; 30 Aug. 1988; M. García; CNIN.

##### Diagnosis.

This species has the body uniformly dark reddish brown, and discoidal, basal antennal flagellomeres. In addition, the forefemur is noticeably robust, with posterior surface yellowish with extensive dark reddish brown spots; the anterior surface is completely dark reddish brown. The pterostigma on both wings is dark reddish brown with a small, yellowish area. On the male genitalia, the gonocoxite IX is long, thin, and gently sigmoid, with nine long and thin processes at posterior apex, which are arranged as a paint-brush. The gonostyli X are anteriorly simply recurved, before protruding from the abdomen. On the female genitalia, the medial part of the gonapophyses VIII is boat-shaped, posteromedially set with a narrow, bifid process, whose projections are digitiform. The spermatheca has the proximal section, thin, long, spiral-shaped, and the distal section abruptly expanded and ovoid at apex.

##### Description.

***Measurements*.** Male (*n* = 5). Forewing length: 9.5–13.5 mm; Hind wing length: 7.5–10.8 mm. Female (*n* = 2): Forewing length: 12.1–15 mm; Hind wing length: 9.5–11.7 mm.

**Figure 42.**
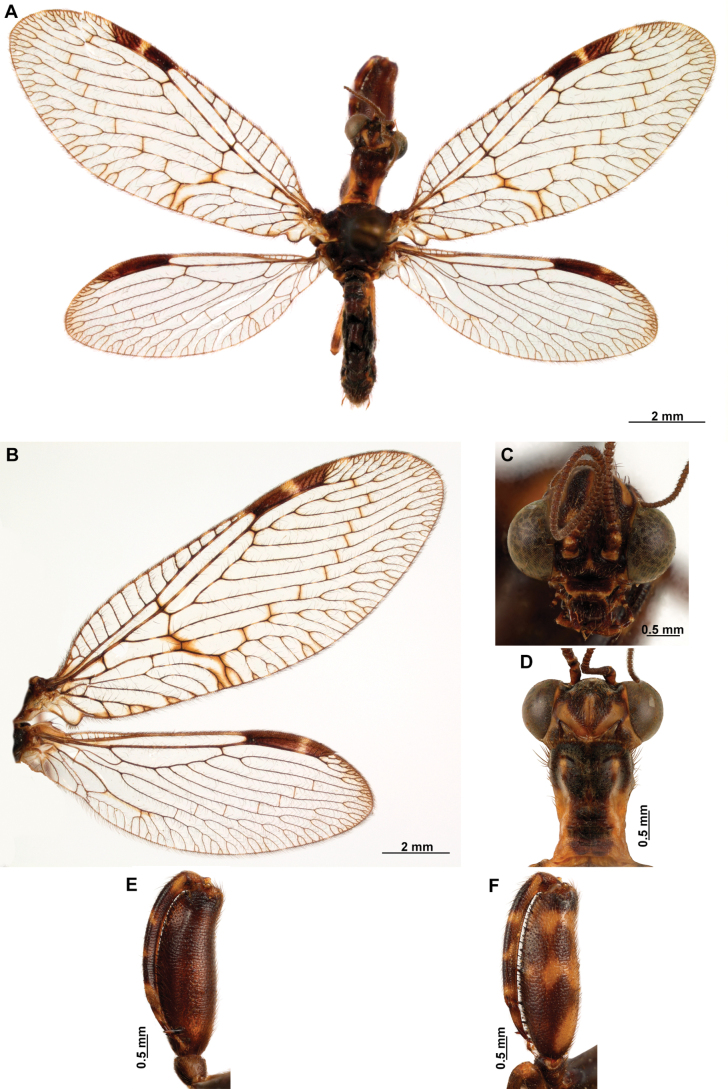
*Plegafumosa* Linsley & MacSwain, 1955 **A** male habitus, dorsal **B** wings **C** head, frontal **D** pronotum, dorsal **E** forefemur, anterior surface **F** same, posterior surface.

***Coloration*** (Fig. 42). ***Head*.** Vertexal region pale brown, with lateral dark reddish brown markings extending from occiput to toruli forming a V-shaped pattern, with brown setae; area adjacent to occipital ridge dark reddish brown; paraocular area alternating pale brown and dark reddish brown; supraantennal area dark reddish brown with lateral semicircular pale brown spots, with brown setae; occiput and postgena pale brown with longitudinal dark reddish brown band. Antennal scape bicolor, pale and reddish brown, entire surface with dark reddish brown setae; pedicel and flagellum dark reddish brown. Frons dark reddish brown with small, triangular, pale brown, medial mark. Clypeus with anterior ½ dark reddish brown, and anterior ½ pale brown; labrum dark reddish brown; mandible dark reddish brown with pale yellow base; maxilla pale with reddish brown areas, palpus reddish brown; labium reddish brown, palpimacula pale brown. ***Thorax*.** Pronotum dark reddish brown, with wide posterolateral yellowish areas, and small, semicircular, anterolateral, yellowish spots; episternum dark reddish brown, sometimes with yellowish, medial area, postfurcasternum yellowish with brown anterior margin. Mesonotum dark reddish brown with anterolateral yellowish spots; metanotum dark reddish brown; pre-episternum dark reddish brown; pteropleura dark reddish brown. ***Foreleg*.** Coxa dark reddish brown; trochanter reddish brown with pale brown areas on dorsal and posterior surface, setae dark reddish brown. Femur posterior surface is yellowish with extensive dark reddish brown spots; anterior surface dark reddish brown; setae mostly brown. Tibia dashed with pale brown and dark reddish brown. Basitarsus mostly reddish brown with pale brown area on proximal ½ of posterior surface; remaining tarsomeres pale brown. ***Mid- and hind leg*.** Coxae dark reddish brown; trochanter of both legs reddish brown with yellowish areas, dark reddish brown setae present. Femur and tibia of mid-leg dashed with pale brown and reddish brown, with reddish brown setae; hind leg with femur pale brown with reddish brown apex, tibia mostly pale brown, with slightly darker base, setae reddish brown; tibial spurs brown. Tarsi pale brown; distal margins on plantar surface with dark brown setae; pretarsal claws brown. ***Wings*.** Forewing mostly hyaline; membrane surrounding crossveins, M fork, medial region and first branch of CuA and apex of CuP, and apical twigging of longitudinal veins amber; pterostigma dark brown with small, yellowish, medial area; major veins, subcostal veinlets, and wing margin alternating yellow and dark reddish brown. Hind wing hyaline; pterostigma dark brown with small, yellowish, preapical area; C, Sc, and RA with pale and dark brown areas, subcostal veinlets pale brown, remaining longitudinal veins dark reddish brown; crossveins brown; wing margin mostly pale brown. ***Abdomen*.** Tergites completely dark reddish brown, except tergite I with yellowish lateral marks and tergites II and III with lateral margins yellowish; setation brown. Pleural membrane mostly dark brown. Sternites mostly dark reddish brown, those of the first abdominal segments medially paler.

***Morphology*** (Fig. 42). ***Head*.** Diamond-shaped in frontal view, rugose; vertexal region raised above compound eyes, with lateral rows of reclined setae; area surrounding coronal suture glabrous, with muscle insertion mark; coronal suture distinct; medial area of ​​supra-antennal region moderately raised, with fine, reclined setae; paraocular area concave. Antenna submoniliform, short; scape slightly longer than wide, slightly distally expanded, mostly with fine, short setae, some thickened ventrally near distal margin; pedicel as long as wide; flagellum slightly dorsoventrally flattened, with 56–63 flagellomeres, which are 2× as long as wide on proximal ¼, and as long as wide on the rest of flagellum; all articles with medial ring of fine, short setae. Compound eye hemispherical, as wide as ½ of the interocular distance at torulus level. Frons and clypeus narrow, with fine and short setae. Labrum pentagonal with thin, short setae; maxillary palpus with first palpomere as long as wide, second 1.2× as long as wide, third palpomere 4× as long as wide, fourth palpomeres 3× as long as wide, fifth slightly shorter than third, all with short and thin setae; mentum with short, thin setae; labial palpus with first palpomere two times as long as wide, second palpomere 4× as long as wide, third palpomere slightly longer than second, palpimacula narrowly ovoid. ***Thorax*.** Pronotum as long as wide, with raised anterior margin, medial and posterior regions; outgrowths covered with thick setae arising flush the pronotal surface; remaining surface with fine, short setae. Mesonotum 1.5× as wide as long, with abundant, thick setae on medial area. Metanotum ~ 2× as long as wide, glabrous. Pteropleura with abundant, long setae. ***Foreleg*.** Coxa as long as femur, cylindrical, anterior and posterior surfaces with fine, long setae; trochanter trapezoidal, with thin and short setae, except on dorsal surface with some thickened, pedicellate setae near distal margin; anterior surface without protuberant area. Femur robust, covered with abundant, fine, short setae; closing surface with posteroventral row of processes composed two medially located, primary processes; proximal portion of the row with two secondary processes; the rest of the row with abundant tubercle-shaped processes, and stinger-shaped setae; distal portion only slightly raised, composed of tubercle-shaped specializations and stinger-shaped setae; adjacent row of thickened setae with globular base present on distal ¼. Anteroventral row of processes reduced to proximal ½; it is composed of tubercle-shaped specializations and stinger-shaped setae; basal-most primary, curved process present; distal portion composed of a few tubercle-shaped processes; adjacent row of thickened setae with globular base present on distal ½. Tibia almost as long as femur, curved, with thin, short setae; ventral surface keeled with prostrate setae; patch of clavate setae apically on anterior surface present. Basitarsus with lanceolate process reaching the middle of fourth tarsomere; clavate setae present proximally on anterior surface; ventrally with single row of prostrate setae; second tarsomere 7× as long as wide; third tarsomere as long as wide, fourth tarsomere two times as long as wide. ***Mid- and hind leg*.** Coxae with long, thin setae; femora and tibiae of both legs with abundant, short, thin setae; tibial spurs short; hind leg longer than midleg, tibia, 1.5× as long as femur; tarsi with fine and short setae, except on distal margin of plantar surface with lateral groups of 5–7 thickened setae; on both legs, basitarsus 3.5 times as long as wide, second tarsomere 1.2× as long as wide; third and fourth tarsomeres as long as wide; fifth tarsomere two times as long as wide. ***Wings*.** Forewing narrowly oval, trichosors present along margin except on wing base; venation setose; costal space proximally expanded, humeral vein branched, 10–17 subcostal veinlets; pterostigma elongated, narrow, straight, with distinct veinlets; subcostal space with single crossvein, medially located; Sc vein abruptly posteriorly bent at proximal pterostigma margin to merge with RA; radial space with two crossveins; *rarp2* gently curved with 2–4 RP branches; three or four veins arising from *rarp1*; M vein basally fused with RA; RP base, close to divergence of M and R; M forked nearly opposite to RP origin, 1 r-m connecting RP base and M, forming a trapezoidal cell; 4–6 gradate crossveins present. Cubitus deeply forked; CuP basally angled and approaching A1, distally forked slightly beyond the level of separation of M and R; A1 apically forked, ending on posterior margin at level or slightly beyond of CuP fork, A2 forked opposite to CuP angle. Hind wing smaller and narrower than forewing, narrowly oval; costal space narrow and reduced, with 6–9 veinlets; C and Sc fused at ¼ of wing length, Sc vein abruptly curved posteriorly at proximal margin of pterostigma to merge RA; pterostigma elongated, narrow, straight, composed of poorly defined veinlets; radial space with single crossvein, oblique; three or four veins arising from *rarp1*, one or two from *rarp2*. 1r-m sigmoid, connecting the stems of M and RP. Media forked beyond R fork. Cubitus deeply forked, intracubital crossvein subparallel to longitudinal wing axis; CuA sinuous, first branch candelabrum-shaped, spur vein absent or present; CuP not touching A1, strongly anteriorly bent at distal 1/3, pectinate; two crossveins on cubitoanal space; A1 simple ending on wing margin slightly beyond 1m-cu level; A2 simple, short, and curved. ***Abdomen*.** Cylindrical to medially expanded, setae on tergites, scattered, thin, and short, gradually longer and more abundant towards terminal segments; tergites subquadrate. Sternites rectangular, with abundant fine and short setae.

**Figure 43.**
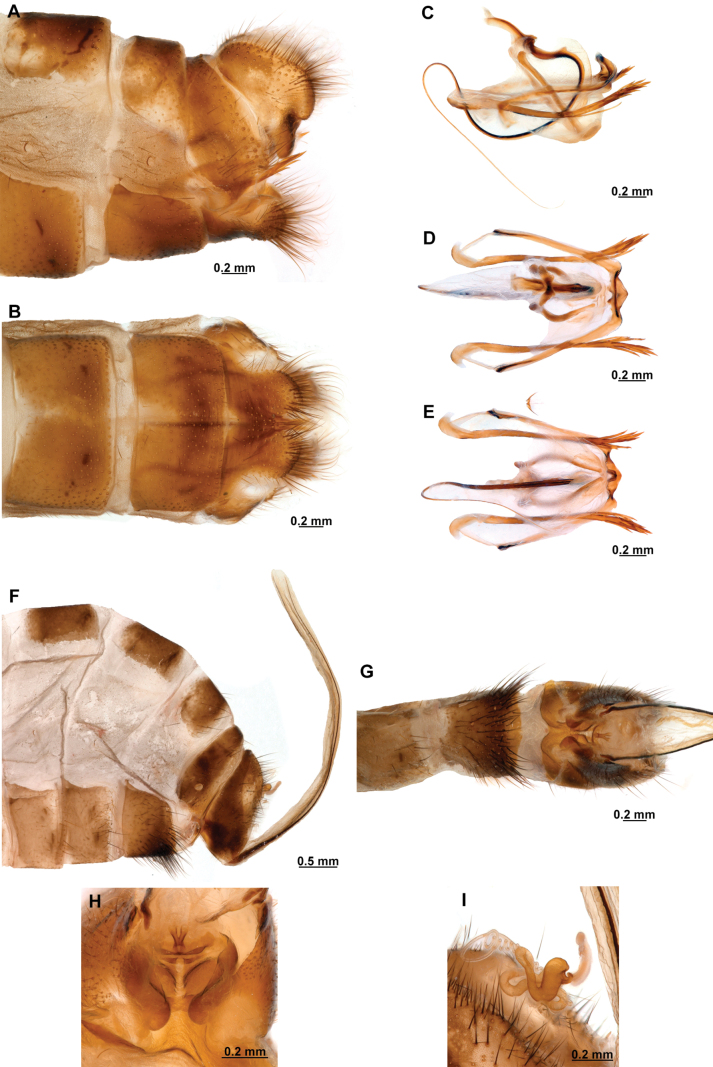
*Plegafumosa* Linsley & MacSwain, 1955 **A** male terminalia, lateral **B** same, ventral **C** male genitalia, lateral **D** same, dorsal **E** same, ventral **F** female terminalia, lateral **G** same, ventral **H** gonapophyses VIII of female, ventral **I** spermatheca.

***Male genitalia*** (Fig. 43A–E). Tergite IX medially narrower than laterally; lateral margin with rounded lobe on posterior corner, and acute process on anterior corner. Sternum VIII rectangular; sternite IX pentagonal in ventral view, with rounded posterolateral lobes covered with abundant, long setae; posterior margin with short, medial, acute lobe, which is dorsally canaliculated; in lateral view triangular, apex reaching posterior margin of ectoproct. Gonocoxites IX thin, gently sigmoid, long; base spatulate, connected to gonocoxites XI with a membrane; apex paint-brush-shaped, with nine, long and thin processes. Ectoproct ovoid, covered with thin setae on posterior surface; anteroventrally with a flattened, rounded lobe which is continuous with ventromedial sclerotized, curved sulcus. Gonocoxites X forming a short, thickened, curved, ventrally canaliculate sclerite; posterior apex with dorsal processes connected to gonostyli X and short ventrolateral processes connected to gonapophyses X with a membrane; gonostyli X with thickened, concave base with two lateral processes, the rest of the structure, thin, whip-shaped, ventrally curved, and anteriorly simply recurved, before protruding from abdomen. Gonapophyses X rod-shaped, narrow, with posterior apex dorsally curved; gonapophyses arranged in a V-shaped structure, joined by a membrane covering the gonostyli X base; this membrane is medially slightly sclerotized. Gonocoxites XI thin, U-shaped, medial lobe complex and elaborated with two differentiated parts: a dorsal, trapezoidal lobe; ventral part as a convex area, which is ventrally continuous with a short ventral process; between these parts a rectangular, less sclerotized, hyaline area is present; lateral arms of gonocoxites XI gently sigmoid, with anterior apex ventrally bent. Hypandrium internum arched.

***Female genitalia*** (Fig. 43F–I). Sternum VII rectangular with abundant, long setae near posterior margin. Tergite VIII narrower medially than laterally, encircling the spiracle of the segment; lateral margin bluntly quadrangular. Gonocoxites VIII as a trapezoidal sclerite with lateral concavities. Gonapophyses VIII with medial part boat-shaped, posteromedially with a narrow, bifid process, whose projections are digitiform; laterally folded beneath IX + ectoproct, a trapezoidal, concave plate is connected to medial part of gonapophyses VIII. Tergite IX + ectoproct narrowly ovoid. Gonocoxites IX elongated, sinuous, and narrow, as long as the last five abdominal segments together. Bursa copulatrix elongated, funnel-shaped, narrow, membranous, and striated, except near genital pore, slightly sclerotized. Spermatheca complex and entangled, proximal section, thin, long, spiral-shaped; medial section wider than proximal section, entangled; distal section abruptly expanded and ovoid at distal portion, a blunt diverticulum is present; fertilization canal duct proximally triangular, concave; fertilization canal C-shaped, covered with microfilaments.

##### Distribution.

Mexico (Michoacán).

##### Remarks.

This is a distinctive species only known from the state of Michoacán, and apparently endemic to Mexico. In the phylogeny of the subfamily, this species was recovered as sister of *P.signata* + *P.flammata*. This species is similar to *P.banksi* in its body color pattern being predominantly dark-reddish brown, although in *P.fumosa* the coloration is more uniform, with few yellowish areas. The forefemur in this species is noticeably robust and the pterostigma is quite distinctive. The overall structure of the male gonocoxite IX is similar to that of *P.banksi*, yet the morphology of the gonocoxites XI is more similar to that of *P.dactylota* or *P.mixteca*, to which this species differs by the simply recurved gonostyli X. In the general structure of the gonapophyses VIII and the spermatheca, this species is similar to *P.dactylota* and *P.mixteca*.

#### ﻿﻿ Plega hagenella

Taxon classificationAnimaliaNeuropteraRhachiberothidae

(Westwood, 1867)

Figs 44 , 45

Mantispa hagenella Westwood, 1867: 504. Holotype: male, Brazil, Amazonas (OUMNH), photographs examined.
Mantispa cognatella Westwood, 1867: 506. Holotype: female, “Bolivia, Sta. Martha” [Colombia, Magdalena, Santa Marta], (OUMNH), photographs examined. Penny (1982a): 425.
Symphrasis cognatella (Westwood,1867). Hagen (1877): 211.
Plega melitomae Linsley & MacSwain, 1955: 15. Holotype: male, Mexico, Chiapas (CAS), specimen examined. New synonym.
Plega beardi Penny, 1982a: 423. Holotype: male, Brazil, Amazonas (INPA), photographs examined. New synonym.

##### Material examined.

***Holotype*** of *Mantispahagenella*.

Brazil • ♂; **Amazonas**; H.W. Bates leg.; 1861; Type Neur: No. 14, *Mantispahagenella* Westwood, HOPE Dept. Oxford; Type Westwood, Trans. Ent. Soc. 1867, P. 504, Coll. Hope Oxon.; Genitalia preparation of *Plegahagenella* (Westwood) 1867 ♂ Holotype made 19.VI.68, Robert G. Beard; OUMNH.

***Holotype*** of *Mantispacognatella*.

Colombia • ♀; [**Magdalena**] Santa Marta; 1866; Stevens leg.; Type Neur: No 17, *Mantispacognatella* Westwood, HOPE Dept. Oxford; *Mantispacognatella* Westwood, 1867 Holotype ♀, ascertained by R.G. Beard, 20.VI.1968; *Mantispacognatella*Westwood 1867 p. 506 is a synonym of *Mantispahagenella*Westwood 1867 p. 504 and also of *Plegamelitomae* L. & McS. 1955, R. Beard 1968; OUMNH.

***Holotype*** of *Plegamelitomae*.

Mexico • ♂; **Chiapas**, Francia, 8 mi N.E. Cintalapa; 03 Apr. 1953; R.C. Bechtel, E.L. Schlinger leg.; Type No. 7203; CAS.

***Paratypes*** of *Plegamelitomae*.

Mexico • 2 ♀; **Chiapas**, Francia, 8 mi. N.E. Cintalapa; 03 Apr. 1953; R.C. Bechtel, E.I. Schlinger leg.; “Paratype ♀ *Plegamelitomae* Linsley & MacSwain”; CAS. • 1 ♂; Francia, 8 m N.E. Cintalapa; 03 Apr. 1953; R.C. Bechtel & E.I. Schlinger leg.; FSCA. • 1 ♀ 1 ♂; same data as for preceding; MCZ.

***Holotype*** of *Plegabeardi*.

Brazil • ♂; **Amazonas**, 15 Km SE of Barcelos; 14 Jan. 1978; N.D. Penny; INPA.

##### Other material.

Brazil – **Goiás** • 1 ♂; Alto Paraíso de Goiás; 14°08'36"S, 47°46'04"W; 17 Jul. 2018; N. Perioto & R. Lara leg.; DZUP. – **Maranhão** • 1 ♂ 1 ♀; Mirador, P E Mirador, Base Geraldina; 06°37'48"S, 45°52'49"W; 2–12 Aug. 2013; F. Limeira de Oliveira, L.L.M. Santos, L.S. Santos leg.; Malaise trap; CZMA. • 1 ♀; same data as for preceding; 06°37'25"S, 45°52'08"W; 14–18 Aug. 2012; F. Limeira de Oliveira, J.S. Pinto Junior, D.W.A. Marques leg.; armadilha suspensa; CZMA. • 1 ♀; same data as for preceding; 7–14 May. 2010; M.M. Abreu & J.S. Pinto Junior leg.; light trap; CZMA. • 1 ♀; Caxias, Res. Ecol. Inhamun; F. Limeira de Oliveira et al. leg.; light trap; CZMA. – **Mato Grosso** • 1 ♀; Cotriguaçu, Faz. São Nicolau, PPBio 1; 09°49'10"S, 58°15'30"W; 01 Nov. 2017; F. Vaz-de-Melo & R.J.P. Machado leg.; light trap; DZUP. • 1 ♀; Chapada dos Guimarães, João Carro; 19 May. 2015; M. Serrano leg.; manual collection; DZUP. – **Pará** • 1 ♂; Serra Norte, est. Manganes; 9–12 Sep. 1983; F.F. Ramos leg.; armadilha suspensa; MPEG. • 1 ♀; same data as for preceding; 19–22 Aug. 1984; MPEG. 1 ?; Óbidos; 18 Aug. 1929; Parish leg.; MCZ – **Piauí** • 1 ♂; Caracol, PN Serra das Confusões, casa do visitante; 09°13'33"S, 43°27'48"W; 23–24 Dec. 2013; J.A. Rafael, F. Limeira de Oliveira, T.T.A. Silva leg.; light trap; CZMA. • 1 ♂; Guaribas, PN Serra das Confusões, Andorinha; 09°08'28"S, 43°33'42"W; 5–8 Jun. 2013; J.A. Rafael, F. Limeira de Oliveira, A.A. Santos leg.; light trap; CZMA. **Rondônia** • 1 ♀; 62 Km SW Ariquemes, nr. Fazenda Rancho Grande; 03–15 Dec. 1996; J.E. Eger leg.; MV & Black lights; FSCA.

Colombia – **Caquetá** • 1 ♂; Solano, Resguardo Kareguaje, Jericó, quebrada Konsaya, La Raya, 10 m, 28.IX.2016, J. Hoyos; CAUD. – **Chocó** • 1 ♂; PNN Los Katíos, Centro Administrativo, Sautatá; 07°51'N, 77°08'W; 30 m; 14 May.–02 Jun. 2004; D. Ramírez leg.; Malaise trap, fuera del bosque; M4987; IavH. – **Cundinamarca** • 1 ♀; Via Guaduas-Honda; 05°04'N, 74°35'W; 300–1000 m; 01 Jun. 2013; G. Bazzani leg.; manual collection; UNAB. • 1 ♂; Supatá; 01 May. 2011; J. del Río leg.; manual collection; CAUD. • 1 ♂; Nimaima, Tóbia; 1220 m; 28 Apr. 2007; N. González leg.; CAUD. • 1 ♀; Nimaima, Tóbia; 05°.07'21.09"N, 74°27'0.81"W; 750 m, D. Arenas; CAUD. • 1 ♂; Nimaima, Tóbia; 750 m; 09 Jun. 2012; A. García; aerial net on Solanacae, 20:00 h; CAUD. • 1 ♀; Nimaima, Tóbia; 28 May. 2007; CAUD. 1 ♀; Tena; 04°39'21"N, 74°23'21"W; 1354 m; 14 Oct. 2017; J. González leg.; CAUD-206; CAUD. – **Huila** • 1 ♂; Tierradentro; 1500 m; 07 Aug. 1971; FSCA. – **Magdalena** • 1 ♀; Neguanje, PNN Tayrona; 11°20'N, 74°02'W; 10 m; 09–27 Sep. 2001; R. Henríquez leg.; Malaise trap, m-2137; IavH. • 1 ♀; Palangana, PNN Tayrona; 11°20'N, 74°02'W; 30 m; 15–30 Nov. 2001; R. Henríquez leg.; Malaise trap, m-2570; IavH. – **Meta** • 1 ♂; Caño Nevera, PNN Tinigua; 02°11'N, 73°48'W; 390 m; 23 Jan.–07 Feb. 2002; C. Sánchez leg.; malaise trap, m-2330; IavH. • 1 ♀; Villavicencio; 450 m; Gassl, N. Banks leg.; MCZ. – **Putumayo** • 1 ♀; Mocoa, vereda Pueblo Viejo, finca Villa Loca; 01°04'28.1"N, 76°38'48.3"W; 690 m; 29 Feb. 2016; R. Romero leg.; manual collection on vegetation; UNAB. –**Santander** • 1 ♀; Barrancabermeja, vereda Las Lajas; 800 m; 02 Oct. 2010; C. Arango leg.; CAUD. • 1 ♀; Chipatá, vereda Ropero, El Hatillo; 1600 m; 18 Jan. 2011; A. Ardila; aerial net, 19:00 h; CAUD. – **Tolima** • 1 ♂; Méndez, Hacienda Bremen; 05°05'N, 74°45'W; 300 m; 11 Jun. 1995; F. Fernández leg.; manual collection; IavH-E 115980. – **Valle del Cauca** • 1 ♂; Cali; 1000 m; FSCA. COSTA RICA – **Heredia** • 1 ♀; Est. Biol. La Selva, Alr. ALAS; 10°26'N, 84°01'W; 50–150 m; 06 Jul. 1995; R. Vargas C. leg.; CRI001247871; MNCR. – **Puntarenas** • 1 ♂; Peninsula Osa; 13–23 Mar. 1978; D.H. Janzen leg.; CRI001688255; MNCR.

Ecuador – **Napo** • 1 ♀; 20 Km E. Puerto Napo, Aliñahui; 1°00'S, 77°25'W; 450 m; Dec. 1995; E.S. Ross leg.; *Plegahagenella*, det. N. Penny, 1999; CAS. • 1 ♂; Napo, Res. Etnica Waorani, 1 Km S. Onkone Gare Camp., Trans. Ent.; 00°38'S, 76°36'W; 220 m; 23 Jan. 1994; T.L. Erwin et al. leg.; insecticidal fogging of mostly bare green leaves, some with covering of lichenous or bryophytic plants, Project MAXUS At x-trans 10, 12 m, Lot 631; USNMENT01541936; USNM.

French Guiana • 2 ♀; **Camopi**, Mont Saint-Marcel, Mont St.-Marcel, de la Huate, Camp mission; 2°23'03.00"N, 53°00'37.00"W; 26 Sep. 2014; Thounrend leg.; automatic light trap (pink); CSCA.

Guyana – [**Essequibo Islands-West Demerara**] • 1 ♀; Essequebo R.; Jul. 1921; A. Busck leg.; USNMENT01541932; USNM. – [**Upper Demerara-Berbice**] • 1 ♂; Dubulay ranch; 5°40.9'N, 57°51.5'W; 9–11 Apr. 1994; O.S. Flint Jr. leg.; USNMENT01541945; USNM. – [**Upper Takutu-Upper Essequibo**] • 1 ♂; Moco-Moco, Lethem, (30 Km east); 3°18.2'N, 59°39.0'W; 3–6 Apr. 1994; W.N. Mathis leg.; USNMENT01541951; USNM.

Mexico – **Chiapas** • 1 ♂; Cacahoatán, Unión Juárez, Río Mixcum; 23 Mar. 1985; M. Velasco leg.; det. D. Reynoso-Velasco, 2005; CNIN. • 1 ♀; Panamerican Hwy. Río de la Venta; 06 Aug. 1956; J.W. MacSwain leg.; FSCA. – **Tabasco** • 1 ♂; Teapa; Dec. 1957; F. Gonzales leg.; FSCA.

Panama – **Canal Zone** • 1 ♂; Close’s Cano Saddle; 15 May. 1923; B.C. Shanon leg.; USNMENT01541933; USNM. • 1 ♀; same data as for preceding; USNMENT01541959; USNM. • 1 ?; Canal Zone, Barro Colorado Island; 02 Jul. 1978; Silberglied & Aiello leg.; at light; USNMENT01541956; USNM. • 1 ♂; same data as for preceding; 10 Apr. 1978; USNMENT01541954; USNM. • 1 ♀; same data as for preceding; 10 Apr. 1978; USNMENT01541946; USNM. • 1 ♀; same data as for preceding; 08 May. 1977; USNMENT01541957; USNM. • 1 ♀; same data as for preceding; 03 Apr. 1978; USNMENT01541952, USNM. • 1 ♀; same data as for preceding; 15 May. 1978; USNMENT01541953; USNM. • 1 ?; Canal Zone, Ft. Clayton; 03 Jul. 1951; F.S. Blanton leg.; USNMENT01541958; USNM. – [**Guna Yala**] • 1 ♂; San Blas, 2 Km S Nusagandi; 03 Mar. 1985; Flint & Louton leg.; USNMENT01541950; USNM. – [**Herrera**] • 1 ♂; Chitré; 08 Aug. 1951; F.S. Blanton; light trap; USNMENT01541955; USNM. – **Panamá** • 1 ♂; 14 Dec. 1968; FSCA. • 1 ♂; Panamá, Las Cumbres; 14 Jan. 1975; L.B. O’Brien; at light; CAS. • 1 ♀; Panamá, Las Cumbres; 17 Dec. 1958; W.J. Hanson leg.; MCZ. • 1 ♂; same data as for preceding; 15 Jun. 1968; MCZ. • 1 ♂; same data as for preceding; 8.VI.1968; MCZ. • 1 ♀; same data as for preceding; 27 Apr. 1966; G.B. Fairchild leg.; MCZ. • 1 ♀; same data as for preceding; 15 Jun. 1968; MCZ. • 1 ♀; same data as for preceding; 23 May. 1968; MCZ. • 1 ♀; same data as for preceding; 16 Jun. 1968; MCZ. • 1 ♀; same data as for preceding; 23 May. 1968; R.G. Beard det.; MCZ. • 1 ♂; Ciudad de Panamá; Feb. 1957; G. Fairchild; MCZ. – [**Panamá Oeste**] • 1 ♀; Cerro Campana, nr. Chica; 18–19 May. 1964; W.D. & S.S. Duckworth leg.; USNMENT01541943; USNM.

Trinidad And Tobago – **Couva-Tabaquite-Talparo** • 1 ♂; 06–22 Jul. 1988; H.L. Dozier leg.; FSCA. – **Tunapuna-Piarco** • 1 ♂; W.I. Brasso Seco; 10°44'45.6"N, 61°17'6"W; 26 Aug. 2008; A.W. Hook; Biol. No. 36-2008; TAMUIC. • 1 ♀; same data as for preceding; 29 Oct. 2008; TAMUIC. • 1 ♂; [Tunapuna-Piarco] St. Agustine; May. 1953; N.L.H. Krauss; USNMENT01541948; USNM. • 1 ♀; Trinidad, Rio Pan; 12 Jun.; A. Busck leg.; USNMENT01541931; *Symphrasiscognatella* West., det. N. Banks, *Plegahagenella* West., det. N. Penny, 1981; USNM. • 1 ♂; Trinidad, Riv. Pan.; 03 Jun. 2012; August & Busck leg.; USNMENT01541963; USNM. • 1 ♂; Trinidad, W.I. Cura Valley; 10°41'16.8"N, 62°22'15.6"W; 26 Jul–9 Aug. 2003; A.W. Hook leg.; TAMUIC. • 1 ♀; Trinidad; 1980; B. Freeman leg.; Ex nest *Trypoxylonmaidli* Richards; NHMUK 013802796; NHMUK. • 1 ♀; same data as for preceding; “Also reared: *Photocryptus* (Ichneum.), *Brachymeria* (Chalcid); NHMUK 013802795; NHMUK.

Venezuela – **Amazonas** • 1 ♀; Cerro La Neblina, Basecamp; 0°50'N, 66°10'W; 140 m; 07 Feb. 1985; W.E. Steiner leg.; at black light on bank of Rio Baria; USNMENT01541934; *Plegahagenella*, det. Flint, 1986; USNM. • 1 ♀; Amazonas, Cerro de la Neblina, Basecamp; 0°50'N, 66°10'W; 140 m; 10–20 Feb. 1985; P.J. & P.M. Spangler, R. Faitoute & W. Steiner leg.; at black light in rainforest clearing near Rio Baria; USNMENT01541935; USNM. • 1 ♀; same data as for preceding; 26–31 Jan. 1985; flight intercept pan trap in rainforest; USNMENT01541937; USNM. – **Guárico** • 1 ♂; 11 mi. W. Dos Caminos; 02 Mar. 1986; R.B. Miller & L.A. Stange leg.; FSCA. • 1 ♂; Guárico, Hato Masaguaral (44 Km S. Calabozo); 11–19 May. 1985; Menke & Carpenter leg.; USNMENT01541962; USNM. • 1 ♀; same data as for preceding; USNMENT01541961; USNM. • 1 ♀; same data as for preceding; 20–28 May. 1985; USNMENT01541960; USNM. – **Miranda** • 1 ?; 2 Sep.; S. Vivas, Berthier leg.; identified as *S.cognatella*; MCZ – **Zulia** • 1 ♀; Tucuco; 23 Apr. 1981; H.K. Townes leg.; FSCA.

##### Diagnosis.

This species has the antennal flagellum brown, with basal flagellomeres as long as wide; 2–6 preapical flagellomeres are pale. The wings are broadly oval, with pterostigma often with pale medial area (or preapical on hind wing) anteriorly notched. The forefemur has the posterior surface pale brown dots; the anterior surface has extensive brown marks and pale areas, including the area adjacent to the anteroventral primary process. On the male genitalia, the gonocoxite IX is long, thin, and sinusoid, with posterior apex equipped with long, thin preapical process on inner surface, and 2–4 shorter, apical processes. The ectoproct has short spinous setal bases posteroventrally. In addition, the median lobe of the gonocoxites XI has the ventral part as a concave covering which forms a ventral, trapezoidal process. On the female genitalia, the gonapophyses VIII medial part is a narrow keel, with two, strongly curved, convergent processes: the ventral one is blunt, and the dorsal one narrow is bilobed at apex; the lateral part is an oval, concave plate which overlaps ventrally with the contralateral. The spermatheca has the proximal section, thin, forming multiple coils, and the rest zigzagged; the medial part forms a long spiral, while the distal section lacks a diverticulum.

##### Description.

***Measurements*.** Male (*n* = 13). Forewing length: 6.9–14.1 mm; Hind wing length: 5.5–11.1 mm. Female (*n* = 9): Forewing length: 8.8–14.8 mm; Hind wing length: 7.3–11.7 mm.

**Figure 44.**
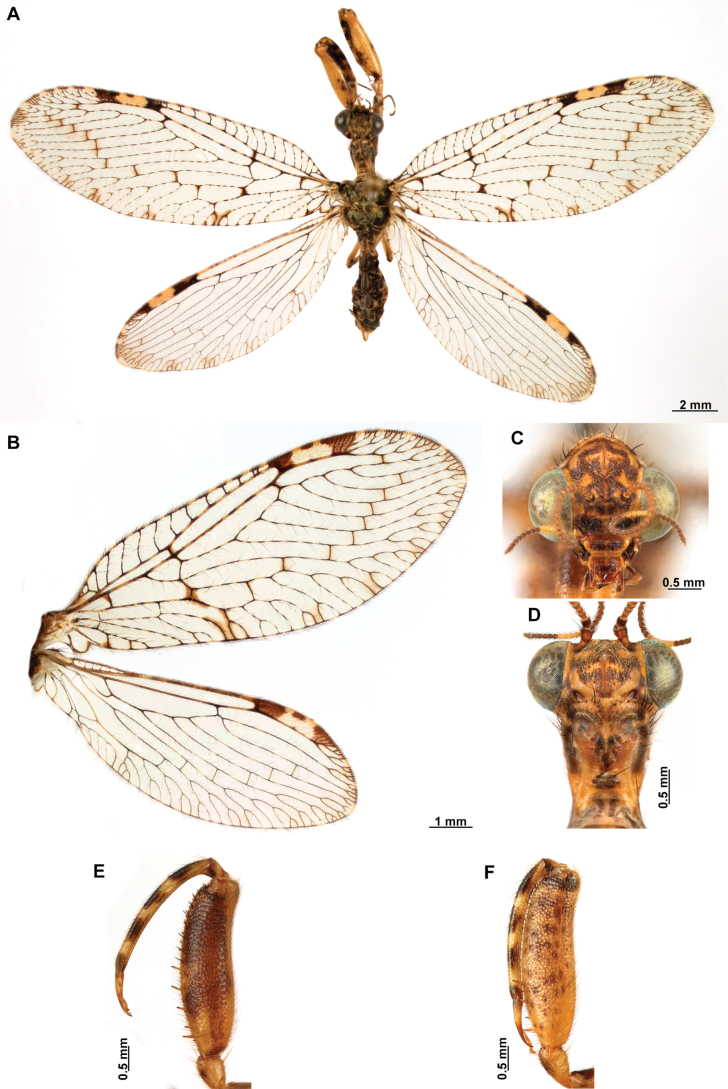
*Plegahagenella* (Westwood, 1867) **A** female habitus, dorsal **B** wings **C** head, frontal **D** pronotum, dorsal **E** forefemur, anterior surface **F** same, posterior surface.

***Coloration*** (Fig. 44). ***Head*.** Vertexal region pale, with irregular, lateral brown marks extending from occiput to supra-antennal area, with brown setae; supra-antennal area brown with small, lateral, pale areas, with pale brown setae; occiput and postgena from pale with brown marks to pale or dark brown. Antennal scape from pale to pale or dark brown, sometimes paler dorsally and darker ventrally; pale to dark brown setae present; pedicel brown, flagellum brown, sometimes proximally paler and becoming darker towards the apex, with 2–6 pale, preapical flagellomeres. Frons either pale with small, lateral, triangular, dark brown marks below the toruli or nearly completely brown. Clypeus brown with pale margins; labrum pale to brown; mandible pale to brown with darker corners, apex amber; maxilla pale, with brown areas on stipes and gale, palpus pale to dark brown; labium from pale to dark brown; labial palpus pale to dark brown, palpimacula pale to brown. ***Thorax*.** Pronotum pale with brown marks on medial region, area adjacent to lateral margins on distal 1/3 brown; episternum either pale to dark brown or bicolor; postfurcasternum pale with broad, brown lateral area. Mesonotum pale on medial region with brown suffusions, dark brown on lateral areas, sometimes nearly completely dark brown with pale areas con scutellum; metanotum pale to dark brown with pale anteromedial area; pre-episternum pale to dark brown; pteropleura pale with brown areas, varying from pale to dark. ***Foreleg*.** Coxa pale with brown areas, sometimes nearly completely dark brown; trochanter pale with brown areas, setation completely pale brown or with some dark brown setae on dorsal and anterior surface. Femur posterior surface pale with brown dots, apical area brown; anterior surface with extensive brown marks and pale areas, area adjacent to anteroventral primary process pale. Tibia dashed with pale and brown. Basitarsus with proximal pale area, lanceolate process pale brown, clavate setae pale; remaining tarsomeres brown. ***Mid- and hind leg*.** Coxae pale with brown marks, setae mostly brown; trochanter pale with brown marks. Femora and tibiae pale with brown rings, setae mostly brown in dark specimens and pale in clearer individuals; tibial spurs pale brown. Tarsi pale to pale brown; distal margins on ventral surface with dark brown setae; pretarsal claws pale brown. ***Wings*.** Forewing mostly hyaline; membrane surrounding crossveins, first branch of CuA and apex of CuP, and sometimes stem of RP an MP amber; posterior and apical margins on distal ½ of wing with intermittent, amber areas between apical branches of longitudinal veins; pterostigma brown with pale medial area with embedded anterior brown mark; major veins, subcostal veinlets, and wing margin alternating pale and brown; crossveins brown. Hind wing hyaline, sometimes with amber intermittent amber areas between apical branches of longitudinal veins adjacent to posterior margin; pterostigma brown with wide, pale, preapical area, sometimes with embedded, anterior, brown mark; venation and wing margin alternating pale and brown, except crossveins brown. ***Abdomen*.** Tergites brown with pale areas. Pleural membrane dark brown with broad pale areas. Sternites brown, sternites III–VI in females IV–VII in males with lateral pale areas.

***Morphology*** (Fig. 44). ***Head*.** Diamond-shaped in frontal view, rugose; vertexal region raised above compound eyes, with lateral rows of reclined, fine setae; area surrounding coronal suture glabrous, with muscle insertion mark; coronal suture distinct; ​​supra-antennal not raised, with fine, reclined setae; paraocular area concave. Antenna filiform, short; scape 1.5× as long as wide, slightly distally expanded, with fine, short setae, some longer and thicker on ventral surface; pedicel slightly longer than wide; flagellum not dorsoventrally flattened, with 33–44 flagellomeres, which are as wide as long on proximal 1/3, then changing to slightly longer than wide; all articles with medial ring of fine, short setae. Compound eye hemispherical, as wide as ½ of the interocular distance at torulus level. Frons and clypeus narrow, with fine and short setae. Labrum pentagonal with thin, short setae; maxillary palpus with first palpomere as long as wide, second 1.5× as long as wide, third palpomere 3.2× as long as wide, fourth palpomere 3× as long as wide, fifth slightly longer than third, all with minute setae; mentum with long, thin setae; labial palpus with first palpomere two times as long as wide, second palpomere 4× as long as wide, third palpomere as long as second, palpimacula narrowly ovoid. ***Thorax*.** Pronotum slightly longer than wide, with raised anterior margin, medial and posterior regions; outgrowths covered with pedicellate, thick setae; remaining surface with fine, short setae. Mesonotum slightly wider than long, with scattered, thick, pedicellate setae on medial area. Metanotum ~ 2× as long as wide, glabrous. Pteropleura with thin, short setae. ***Foreleg*.** Coxa as long as femur, cylindrical, anterior and posterior surfaces with fine setae of different lengths, some of which arise from protuberant bases; trochanter trapezoidal, with thin and short setae, except on dorsal and anterior surfaces with some, pedicellate, thickened setae; anterior surface with protuberant area. Femur robust, elongate, covered with abundant, fine, short setae, those of anterior surface arising from protuberant bases; closing surface with posteroventral row of processes composed two medially located, primary processes, generally without a tertiary process between them; proximal portion with basal tertiary process and sub-basal secondary process, very close to the first primary process; the rest of the row with numerous tubercle-shaped processes, and stinger-shaped setae; distal portion raised, composed of tubercle-shaped specializations and stinger-shaped setae; adjacent row of thickened setae with globular base present on distal ¼. Anteroventral row of processes reduced to proximal ½, composed of tubercle-shaped specializations and stinger-shaped setae; the basal-most primary, curved process is present; adjacent row of thickened setae with globular base present on distal ½. Tibia almost as long as femur, curved, with thin, short setae; ventral surface keeled with prostrate setae; a patch of clavate setae apically on anterior surface is present. Basitarsus with lanceolate process reaching the middle of fourth tarsomere; clavate setae present proximally on anterior surface; ventrally with single row of prostrate setae; second tarsomere nearly 7× as long as wide; third tarsomere as long as wide, fourth tarsomere two times as long as wide. ***Mid- and hind leg*.** Coxae and trochanter with thin setae, shorter on trochanter; femora and tibiae with abundant interspersed fine setae of different lengths; tibial spurs short. Hind leg longer than midleg, tibia nearly two times as long as femur; tarsi with fine and short setae, except on distal margin of plantar surface with lateral groups of 3–6 thickened setae; on both legs, basitarsus 4.5 times as long as wide, second tarsomere 1.2× as long as wide; third and fourth tarsomeres as long as wide; fifth tarsomere two times as long as wide. ***Wings*.** Forewing oval, trichosors present along margin except on wing base; venation setose; costal space proximally moderately expanded, humeral vein branched, 9–14 subcostal veinlets, mostly simple; pterostigma elongated, narrow, trapezoidal, straight, with most of its veinlets incomplete; subcostal space with single crossvein, located at R fork level; Sc vein abruptly posteriorly bent at proximal pterostigma margin to merge with RA; radial space with two crossveins; *rarp2* gently curved with two or three RP branches; 3–5 veins arising from *rarp1*; M vein basally fused with RA; RP base not widely separated from divergence of M and R; M forked opposite to RP origin, 1 r-m connecting RP base and M fork, forming a trapezoidal cell; 4–7 gradate crossveins present. Cubitus deeply forked; CuP basally angled and approaching A1, distally forked opposite to the level of separation of M and R; A1 simple or apically forked, ending on posterior margin at level of CuP fork, A2 forked opposite to CuP angle level. Hind wing smaller and narrower than forewing, narrowly oval; costal space narrow and reduced, with four or five veinlets; C and Sc fused at ¼ of wing length, Sc vein abruptly curved posteriorly at proximal margin of pterostigma to merge RA; pterostigma elongated, narrow, gently curved, composed of weakly defined veinlets; radial space with single crossvein, oblique; 3–5 veins arising from *rarp1*, one or two from *rarp2*. 1r-m sigmoid, connecting the stems of M and RP. Media forked slightly beyond R fork. Cubitus deeply forked, intracubital crossvein subparallel to longitudinal wing axis; CuA sinuous, first branch candelabrum-shaped, spur vein absent or present; CuP not touching A1, strongly anteriorly bent at distal ½, pectinate; two crossveins on cubitoanal space; A1 simple, ending on wing margin at 1r-m stem level; A2 simple, short, and curved. ***Abdomen*.** Cylindrical to medially expanded, setae on tergites, scattered, thin, and short; tergites subquadrate. Sternites rectangular, with scattered fine and short setae.

**Figure 45.**
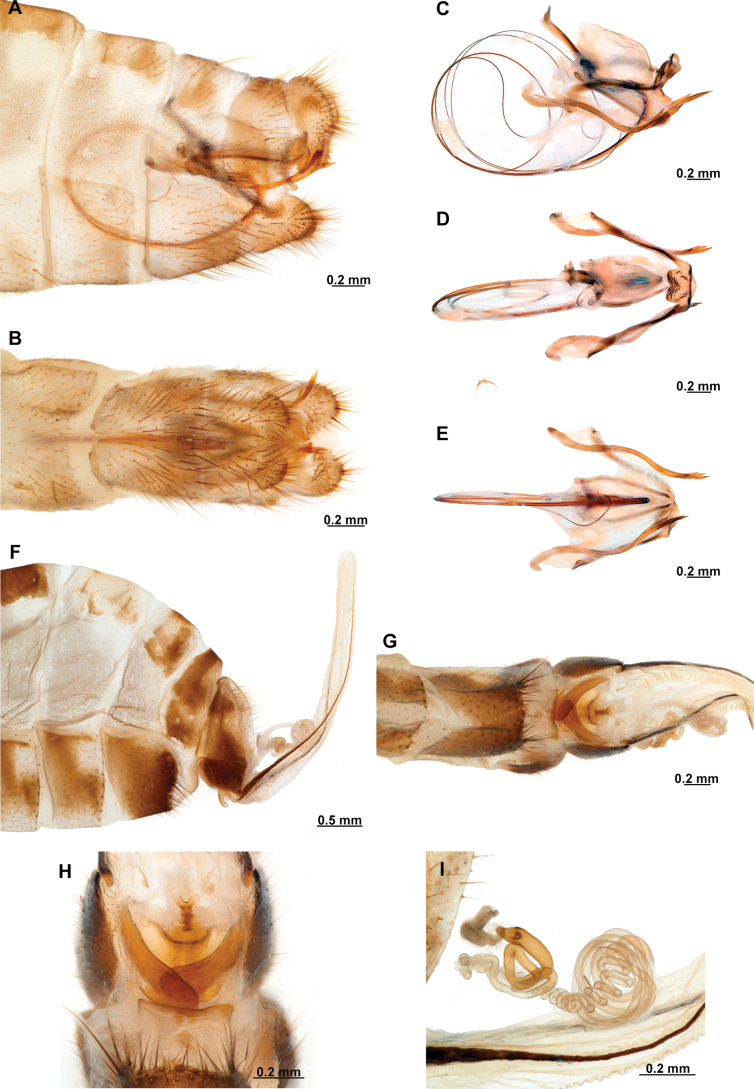
*Plegahagenella* (Westwood, 1867) **A** male terminalia, lateral **B** same, ventral **C** male genitalia, lateral **D** same, dorsal **E** same, ventral **F** female terminalia, lateral **G** same, ventral **H** gonapophyses VIII of female, ventral **I** spermatheca.

***Male genitalia*** (Fig. 45A–E). Tergite IX medially narrower than laterally; lateral margin rounded. Sternum VIII rectangular; sternite IX pentagonal in ventral view, with rounded posterolateral corners rounded, covered with fine, short and long setae; posterior margin with medial lobe short, dorsally canaliculated; in lateral view triangular, apex approaching posterior margin of ectoproct. Gonocoxites IX thin, sinusoid, long; base dorsally curved; apex with long and thin preapical process on inner surface, and 2–4 apical shorter processes. Ectoproct as an obtuse triangle, with thickened setae on posterior surface, callus cerci vestigial, posteroventrally with short spines; ventral margin forming a rounded lobe covered with microtrichia. Gonocoxites X forming a slightly elongate, thickened, ventrally canaliculate sclerite, anterior apex expanded and dorsally bent, posterior apex with dorsal processes connected to gonostyli X and ventrolateral processes connected to gonapophyses X with a membrane; gonostyli X with thickened and curved base with two lateral processes; the rest of the structure, whip-shaped, ventrally curved, and anteriorly forming single two loops before protruding from abdomen. Gonapophyses X long, narrow with posterior apex dorsally curved; gonapophyses arranged in a V-shaped structure, joined by a membrane covering the gonostyli X base. Gonocoxites XI U-shaped, medial lobe complex and elaborated with two differentiated parts: dorsal part as narrow arch; ventral part, forming a concave covering which forms a ventral, trapezoidal process; between these parts a narrow, arched or ellipsoid, less sclerotized, hyaline area is present; lateral arms of gonocoxites XI long, gently curved, with anterior apex bent ventrad. Hypandrium internum tiara-shaped.

***Female genitalia*** (Fig. 45F–I). Sternum VII rectangular, with posterior margin concave. Tergite VIII medially narrower than laterally, encircling the spiracle of the segment; lateral margin D-shaped. Gonocoxites VIII as a rounded, concave sclerite. Gonapophyses VIII with medial part as a narrow keel, with two, strongly curved, convergent processes: ventral process, blunt, dorsal process narrow with bilobed apex; lateral part as an oval, concave plate folded beneath IX + ectoproct. Tergite IX + ectoproct narrowly ovoid. Gonocoxites IX elongated, and narrow, as long as the last four abdominal segments together. Bursa copulatrix long, funnel-shaped, sclerotized near genital pore, the rest membranous and striated. Spermatheca entangled and long; proximal section, thin, forming multiple coils, the rest zigzagged; medial section as wide as proximal section, forming a long spiral; distal section slightly, progressively expanded, without diverticulum; fertilization canal duct long, with proximal portion concave, progressively narrowed; fertilization canal long, curved, covered with microfilaments.

##### Distribution.

Brazil (Acre, Amazonas, Ceará, Goiás, Maranhão, Mato Grosso, Minas Gerais, Pará, Piauí, Rondônia, Roraima, Tocantins, Rio Grande do Norte), Bolivia, Colombia (Antioquia, Caquetá, Chocó, Cundinamarca, Huila, Magdalena, Meta, Putumayo, Santander, Tolima, Valle del Cauca), Costa Rica (Heredia, Puntarenas), Ecuador (Napo), French Guiana (Camopi), Guyana (Essequibo Islands-West Demerara, Upper Demerara-Berbice, Upper Takutu-Upper Essequibo), Mexico (Chiapas, Tabasco), Nicaragua, Panama (Canal Zone, Guna Yala, Herrera, Panamá, Panamá Oeste), Trinidad and Tobago (Couva-Tabaquite-Talparo, Tunapuna-Piarco), Venezuela (Amazonas, Guárico, Miranda, Zulia).

##### Remarks.

This is a widely distributed and common species in Northern South America and Panamá, although it reaches Central America and Southern Mexico.

Here, two new synonyms of this species are proposed, i.e., *P.melitomae* and *P.beardi*. The former species has all the genital sclerites of both sexes indistinguishable from *P.hagenella*, and the general color pattern is basically the same, despite that it is highly variable. In the case of *P.beardi*, it was separated from other Amazonian species by Penny (1982a) because of the number of processes on the male gonocoxites IX and the uniformly dark antennal flagellum. After examining numerous specimens from different locations and of different species, it was concluded that: 1) the number of processes in *P.hagenella* is variable, with two to five processes. It is worth mentioning that *P.beardi* has the same general arrangement on the processes and the overall shape of the gonocoxites IX and other genital sclerites is basically the same as in *P.hagenella*. *Plegabeardi* was diagnosed based on the completely brown antennal flagellum, yet this is a variable character since in other species the antennal flagellum may have pale preapical flagellomeres or be completely brown (i.e., *P.spinosa*). The presence of pale preapical flagellomeres in *P.hagenella* and the uniformly brown flagellum in *P.beardi* could be explained as phenotypic variation, since the coloration of the rest of the body is basically the same in both species. *Plegahagenella* was recovered within the clade of South American species of *Plega* as sister to *P.bowlesi* + *P.radicaudata*, to which it is remarkably similar.

#### ﻿﻿ Plega insolita

Taxon classificationAnimaliaNeuropteraRhachiberothidae

Ardila-Camacho & Contreras-Ramos sp. nov.

https://zoobank.org/B836957E-B6CC-4E60-89AE-8ECC08F916AE

Figs 46 , 47

##### Type locality.

Mexico, **Sonora**: 28.5 Km SE Tecoripa, Cerro Verde, 28°33'09.5"N, 109°43'34"W, 532 m, 19 Apr. 2004, light trap, S. Zaragoza leg.

##### Material examined.

***Holotype*** male, pinned, with genitalia in a separate microvial. Original label: “Mexico, **Sonora**, 28.5 Km SE Tecoripa, Cerro Verde, 28°33'09.5"N, 109°43'34"W, 532 m, 19 Apr. 2004, trampa de luz, S. Zaragoza leg.”, USNM. ***Paratypes***. Mexico – **Sonora** • 1 ♂; 8 mi. SE. Álamos; 28 Mar. 1961; *Plega* sp. det. N. Penny, 1998; CAS. – **Sinaloa** • 3 ♂; 16 mi. S. Guamúchil; 20 May. 1962; F.D. Parker & L.A. Stange leg; FSCA. • 2 ♂; 16 mi. S. Guamúchil; 16 Mar. 1962; F.D. Parker & L.A. Stange leg.; FSCA.

##### Other material.

Mexico – **Sonora** • 1 ♂; 36.6 Km SE Tecoripa, La Barranca; 562 m; 28°34'40.1"N, 109°39'54.3"W; 20 Apr. 2004; S. Zaragoza, F. Noguera, E. Ramirez & E. Gonzales leg.; light trap; CNIN. – **Sinaloa** • 1 ♂; 16 mi. S. Guaymuchil [Guamúchil]; 16 Mar. 1962; L. Stange leg.; FSCA. • 1 ♂; same data as for preceding; 16 mi. S. Guamúchil; 16 Mar. 1962; F.D. Parker & L.A. Stange leg.; FSCA. • 2 ♂; same data as for preceding; 16 mi. S. Guamúchil; 20 May. 1962; F.D. Parker & L.A. Stange leg.; FSCA. • 1 ♂; 20 mi. S. Guamúchil; 05 May. 1962; F.D. Parker & L.A. Stange leg.; FSCA.

##### Diagnosis.

This species is distinguished by the quadrangular head in frontal view, with vertexal region strongly domed over the compound eyes. The compound eye appears relatively small in relation to head size. The postocular region is markedly widened and bulging in lateral view. The pronotum is slightly wider than long, where the posterior margin has a transverse ridge, the medial region has two lateral protrusions and a triangular muscle attachment mark at the center. The foreleg is inserted at posterior end of the prothorax; the moderately thickened femur lacks a primary, curved process on the anteroventral row of integumentary specializations. The tibia is nearly straight, and the forebasitarsus has a short lanceolate process, which reaches the middle of the third tarsomere. The costal field of the forewing presents simple and forked subcostal veinlets.

##### Etymology.

The specific epithet of this species *insolita* alludes to the peculiar morphology of this species. An adjective in the nominative case.

##### Description.

***Measurements*.** Male (*n* = 9). Forewing length: 12.5–16.7 mm; Hind wing length: 10.0–14.2 mm.

**Figure 46.**
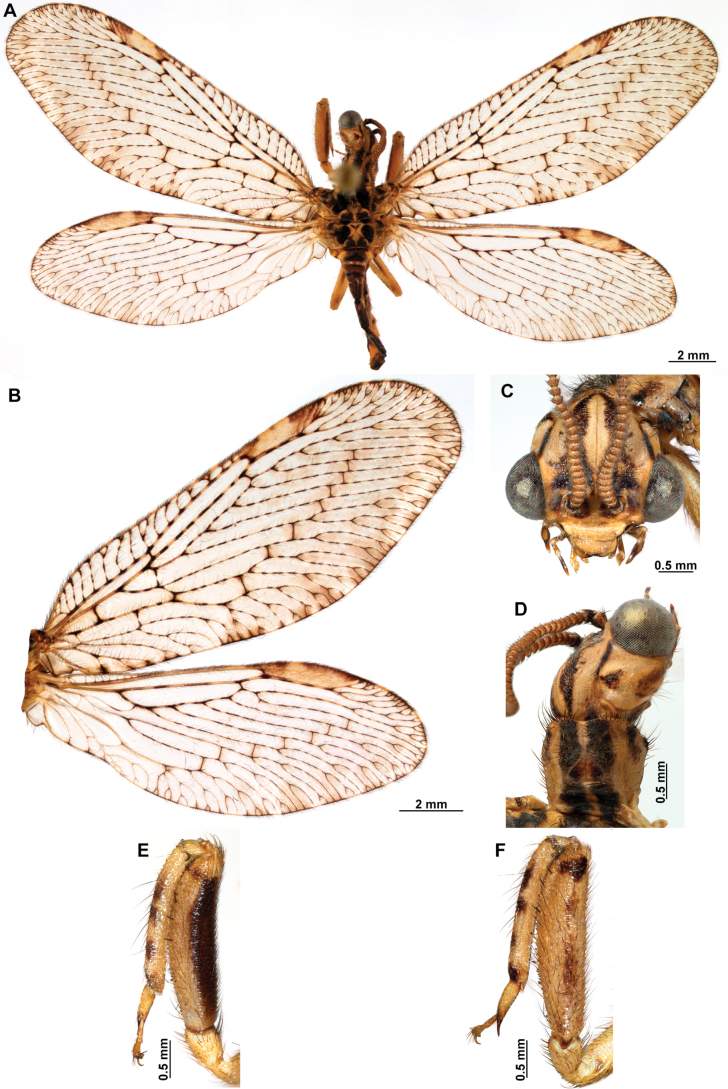
*Plegainsolita* Ardila-Camacho & Contreras-Ramos, sp. nov. **A** male habitus, dorsal **B** wings **C** head, frontal **D** pronotum, dorsal **E** forefemur, anterior surface **F** same, posterior surface.

***Coloration*** (Fig. 46). ***Head*.** Mainly pale ocher, with two dark brown bands with paler areas extending from occipital ridge to toruli; area adjacent to occipital transverse ridge with dark brown stripe. Postgena pale ocher with brown markings; hypostomal bridge pale to dark amber. Antennal scape dark brown, with pale ocher dorsolateral band, pedicel dark brown; flagellomeres pale brown with darker setae. Frons mainly pale ocher; clypeus and labrum pale ocher; mandible mainly pale ocher, dark brown dorsal- and ventrally on basal ½, apex amber. Labium with pale ocher submentum with two brown lateral markings; prementum pale brown, labial palpus with first two palpomeres mainly brown, pale ocher near joints, last palpomere completely brown except for pale ocher palpimacula; maxilla with pale ocher cardo and stipes, with dark brown markings, galea and lacinia pale brown, palpus with first four palpomeres brown, ocher near the joints, fifth palpomere completely brown. ***Thorax*.** Pronotum pale ocher, with a pair of wide, dark brown, medial bands, posteromedially with pale to dark brown triangle; anterolaterally with a dark brown stripe extending to mid-length of pronotum. Episternum pale ocher with dark brown areas, postfurcasternum pale ocher. Mesonotum pale ocher with two broad dark brown medial bands on scutum that extend posterolaterally; area adjacent to wing bases with broad, dark brown spots; scutellum pale ocher with two broad, dark brown, lateral spots. Metanotum with broad, dark brown, lateral spots, with paler areas on scutum, pale ocher medially; scutellum pale ocher with broad, dark brown lateral areas, connecting at posterior margin. Pre-episternum with a broad dark brown band. Pteropleura with epimera presenting broad, dark brown spots, episterna having from faint suffusions to well-marked dark brown spots, particularly on the metathorax. ***Foreleg*.** Coxa pale ocher, with brown preapical mark on ventral surface, interspersed pale to dark brown setae present; trochanter pale ocher. Femur pale ocher, sometimes with medial, dashed line on posterior surface, with brown apical marking; anterior surface with weak to strongly marked brown medial band. Tibia pale ocher with brown spots on dorsal surface, prostrate setae pale. Basitarsus mainly pale ocher, with base and lanceolate process varying from pale to dark amber; clavate setae pale ocher; second to fourth tarsomere pale brown. ***Mid- and hind leg*.** Mid-leg mostly pale ocher, coxa with brown at junction with thorax; trochanter bicolor, brown posteriorly; femur with medial, brown suffusions, more marked on posterior surface; tibia dorsally alternating brown and pale ocher; tarsomeres sometimes pale brown towards the apex. Hind leg mostly pale ocher, trochanter, femur, and tibia with brown suffusions, weak to well-marked; tarsomeres as in mid-leg. ***Wings*.** Forewing with pale amber membrane, with darker areas surrounding crossveins, and forks of longitudinal veins; intermittent darker areas along wing margin also present; pterostigma either completely pale brown, or dark brown, with pale medial area; longitudinal veins and subcostal veinlets alternating pale ocher and pale to dark brown; crossveins pale to dark brown; posterior and distal margin alternating brown and pale ocher. Hind wing membrane hyaline, with amber areas adjacent to crossveins, terminal branches of longitudinal veins and base RP branches; pterostigma either completely pale to brown, with pale medial area; venation mostly alternating brown and pale, except Cu and anal veins mostly pale; posterior and distal margin alternating pale and brown. ***Abdomen*.** Tergites brown, pale ocher on area adjacent to lateral margins, dark brown on area adjacent to posterior margin. Sternites mostly ocher, with brown markings on area adjacent to anterolateral corners. Pleural membrane pale ocher with extensive dark brown areas medially.

***Morphology*** (Fig. 46). ***Head*.** Sub-quadrate in frontal view, rugose, vertexal region strongly domed above compound eyes; paraocular area concave; coronal suture noticeable. Antenna moniliform, scape and pedicel as long as wide, both with short, thin setae; flagellum slightly dorsoventrally compressed, with 52–60 flagellomeres, ~ 4× as wide as long at flagellar base, progressively narrower, becoming trapezoidal towards the apex, apical flagellomere elongated and acuminate; all flagellomeres covered with short, thin setae, not arranged in a ring. Compound eye hemispherical, as wide as ½ of interocular distance at toruli level; postocular region markedly expanded, as wide as ocular diameter. Postgena broad, hypostomal bridge completely fused with it. Maxillary palpus with first palpomere as long as wide, second 1.5× as long as wide, third palpomere 3× as long as wide, fourth 3× as long as wide, fifth palpomere slightly shorter than third. Submentum U-shaped, with long, thin setae; labial palpus, with first palpomere 2.5× as long as wide, the second 4× as long as wide, third slightly shorter than the second, basally expanded, with ovoid palpimacula. ***Thorax*.** Pronotum slightly wider than long, with furrow contiguous to lateral and anterior margins; posterior margin with transverse ridge; medial region with two lateral protrusions, and triangular, central muscle attachment mark, anterior margin raised; raised areas with long, thin, pedicellate setae. Episternum with long, fine setae; postfurcasternum trapezoidal. Mesonotum wider than long, with abundant, long, thickened, pedicellate setae on medial area. Metanotum 3× as wide as long, scutellum with long, thin setae. Pteropleura with abundant short, thin setae on mesepisternum and metapleura. ***Foreleg*.** Coxa as long as femur, cylindrical, with abundant long, thin setae; trochanter trapezoidal, covered with short, thin setae, thicker and elongated on dorsal surface; femur moderately thickened, with abundant long, thin setae; closing surface glabrous; posteroventral row of integumentary processes fully developed, slightly carinated on distal 1/3, composed of tubercle-shaped specializations (process/seta ratio 1:1 or 2:1) of different sizes, and with some stinger-shaped setae; with two primary spine-shaped processes (process/seta ratio 4: 1) located at middle and on proximal 1/3 of femur length, between these, a secondary process is present; proximal 1/3 of the row with some secondary and tertiary processes; adjacent row of thickened setae with globular base present on distal 1/3 to l ¼ of femur length; anteroventral row of processes reduced to proximal 1/3 and apex, composed of tubercle-shaped processes, with one or two more developed processes at base; adjacent row of thickened setae with globular base present on distal ½. Tibia slightly shorter than femur, gently curved, setose, ventrally keeled, with a row of prostrate setae; anterior surface with patch of clavate setae at apex, distal edge forming a blade-shaped process. Basitarsus with short lanceolate process reaching the middle of third tarsomere, equipped with apical plug-shaped Stitz organ; ventral surface keeled, with a row of prostrate setae, anterior surface with patch of clavate setae on basal ½; second tarsomere articulated at basal ½ of basitarsus on anterior surface, 3× as long as wide; third tarsomere as long as wide, fourth tarsomere as long as second, thickened; pretarsal claws simple. ***Mid- and hind leg*.** Mid-leg with coxa, trochanter and femur covered with long, thin setae; tibia slightly longer than femur, with shorter and thickened setae compared to proximal podomeres; tibial spurs short and thickened; basitarsus 2× as long as wide, second tarsomere slightly longer than wide, third tarsomere as long as wide, fourth tarsomere slightly wider than long, fifth tarsomere 2.5× as long as wide, all covered with short, thin setae; the first four tarsomeres with groups of 7–9 thickened setae laterally on distal margin of plantar surface. Hind leg longer than mid-leg, tibia 1.5× as long as femur; setae on the coxa, trochanter and femur long and thin, shorter on the tibia; tarsus as in mid-leg. ***Wings*.** Forewing oval, trichosors present along wing margin except at base (single trichosor between apex of two veins on apical margin and 1–4 on the posterior margin); venation setose; costal space moderately widened proximally, humeral vein generally branched, 14–19 subcostal veinlets, simple, forked, and branched randomly arranged; pterostigma trapezoidal, composed of numerous veinlets; subcostal space with single, medially located crossvein; Sc vein bent posteriad at proximal margin of pterostigma to merge the RA; *rarp2* curved, with 2–4 veins arising from it, four or five from *rarp1*; M fused basally to R; RP base located near separation of M and R, M fork also near such separation, 1 r-m situated between RP base and MA base, forming a small trapezoidal cell; five or six gradate crossveins present; M fork opposite to R fork. Cu vein deeply forked, CuA distally branched; CuP basally angled, approaching A1, medially forked, opposite to separation of M and R. A1 apically forked, reaching the level of separation of M and R; A2 medially forked, at level of CuP, branched. Hind wing smaller and narrower than forewing; costal space narrow and reduced, 8–10 veinlets present; Sc and C fused at proximal ¼ of wing length; Sc curved posteriad, at proximal margin of pterostigma to merge with RA; pterostigma subtrapezoidal, distally widened, composed of poorly marked veinlets; radial space with a single crossvein, oblique; four or five veins arising from *rarp1*, one or two from *rarp2*. M forked slightly beyond R fork; 1 r-m sigmoid, connecting M base and RP base. Cu deeply forked, CuA long and sinuous, distally branched, first branch candelabrum-shaped; CuP long, distally branched, ending at posterior margin slightly beyond R fork level; intracubital vein subparallel to longitudinal wing axis. Cubitoanal space with single distal crossvein. A1 distally forked, reaching R fork level. A2 distally forked, ending on posterior margin at 1m-cu level. ***Abdomen*.** Cylindrical, tergites quadrangular, tergites III–V with posteromedial concavity; sternites rectangular.

**Figure 47.**
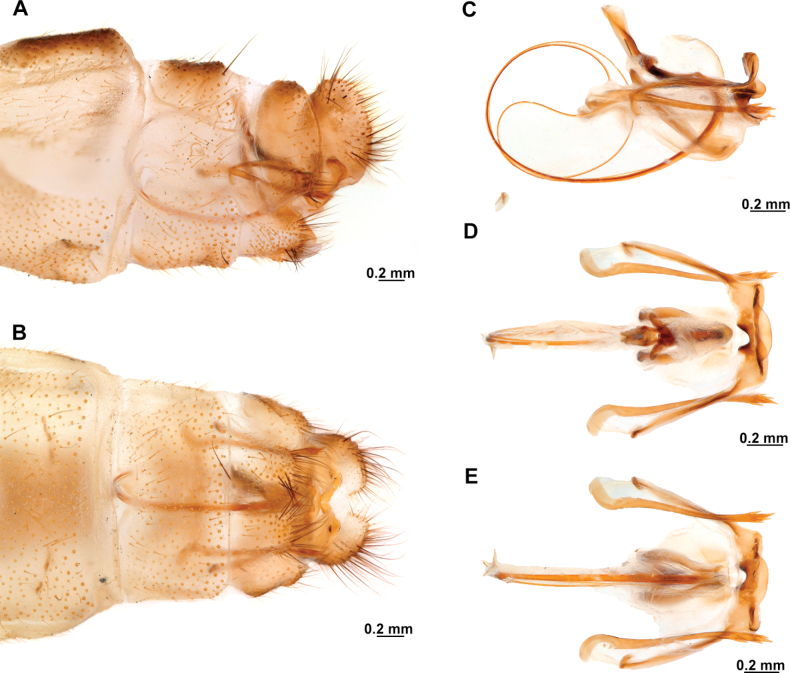
*Plegainsolita* Ardila-Camacho & Contreras-Ramos, sp. nov. **A** male terminalia, lateral **B** same, ventral **C** male genitalia, lateral **D** same, dorsal **E** same, ventral.

***Male genitalia*** (Fig. 47). Tergite IX setose, medially narrower than laterally, lateral margin rounded. Sternite VIII subrectangular; sternite IX pentagonal with rounded and convex posterolateral corners, posteromedially forming an acute, dorsally canaliculated lobe, glabrous, the rest of the surface with abundant long, thin setae; in lateral view acuminate, not reaching the posterior margin of ectoproct. Gonocoxite IX short and thin, base spatulate; apex with 6–12 apical, subequal processes, arranged like a brush. Ectoproct oval; ventral surface with flattened anterior lobe covered with microtrichia, continued posteriorly by an arched, sclerotized groove. Gonocoxites X forming a short, ventrally canaliculated, subrectangular sclerite; posterior apex bilobed connected to gonostyli X, two preapical, ventrolateral processes are connected to gonapophyses X with a membrane. Gonostyli X with thin, concave base, set with two lateral processes, the rest of the structure long, curved ventrally and coiled anteriorly, forming two internal loops before protruding from abdomen. Gonapophyses X straight, thin, posterior apex curved dorsally with surrounding membrane set with minute granules; gonapophyses arranged in a V-shaped structure, joined by moderately sclerotized membrane covering gonostyli X base. Gonocoxites XI U-shaped, medial lobe expanded and elaborated, with two differentiated parts: dorsal one as an arched lobe; ventral part a convex structure, dorsoventrally flattened, with granular texture ventrally continued by curved, rectangular process; between these parts a quadrangular, less sclerotized region is present; lateral arms of gonapophyses XI, slightly sinuous, anterior apex ventrally curved. Hypandrium internum concave, keeled, arrowhead-shaped.

***Female genitalia*.** Unknown.

##### Distribution.

Mexico (Sinaloa, Sonora).

##### Remarks.

This is a striking species of *Plega* until now solely known from the Mexican states of Sonora and Sinaloa, and probably endemic to the country. This species expresses numerous plesiomorphisms shared with *Trichoscelia* and *Anchieta*, yet it was recovered as sister to the clade *P.banksi* (*P.megaptera* + *P.sonorae*), a group of species distributed in the same area in Northern Mexico and Southern United States. Among the species of the genus, *P.insolita* has a narrow forefemur lacking a primary basal process on the anteroventral processes row of forefemur, which is a unique condition within the genus and expressed by *Trichoscelia*. However, this row is reduced to the proximal region and the apex of the femoral closing surface as in the majority of species of *Plega*. On the posteroventral row of processes, two primary processes on the proximal ½ of the femur are present, which represent synapomorphy of *Plega*. Additionally, this species has a straight foretibia and a short lanceolate process of the foretarsus, conditions also observed in Rhachiberothinae and *Trichoscelia*, respectively.

The remarkable short prothorax is shared with other species of *Plega* (i.e., *P.sonorae*) and Rhachiberothinae, and the paired and plate-shaped postfurcasterna are also present in *Anchieta* and *Trichoscelia*. On the head, the compound eyes are noticeably small and the vertexal region is conspicuously enlarged, a unique condition among *Plega* species. Moreover, the male terminalia and the gonocoxites XI express a similar morphology to that of *P.sonorae* and *P.megaptera*.

#### ﻿﻿ Plega lachesis

Taxon classificationAnimaliaNeuropteraRhachiberothidae

Ardila-Camacho & Contreras-Ramos sp. nov.

https://zoobank.org/BF2FD4C6-D74B-42F1-AA86-ACB02162B2BE

Figs 48 , 49

##### Type locality.

Guatemala, **Guatemala**: Santa Fé, 07 Mar. 1992, P. Hunzker leg.

##### Material examined.

***Holotype*** male, pinned, with genitalia in a separate microvial. Original label: “Guatemala, **Guatemala**, Santa Fé, 07 Mar. 1992, P. Hunzker leg.” FSCA.

##### Diagnosis.

This species has the basal antennal flagellomeres as wide as long; two preapical articles are pale. The general body coloration is similar to that of *P.spinosa*, yet the lack of a sunken area around the coronal sutures and raised, lateral areas on supra-antennal region covered with thickened, reclined setae in *P.lachesis* separates both species. The pterostigma on both wings is brown with pale areas. On the male genitalia, the gonocoxite IX is thin, straight, and long, with posterior apex bifid. The gonostyli X are short and anteriorly incurved. The ventral of the gonocoxites XI median lobe is composed of a convex area, whose ventral surface forms a concave covering.

##### Etymology.

The name of this species refers to the genus *Lachesis* Daudin, 1803 (Serpentes: Viperidae), which includes the largest vipers of the American continent. The bifid male gonocoxite IX of this species resembles the viper’s tongue.

##### Description.

***Measurements*.** Male (*n* = 1). Forewing length: 12.6 mm; Hind wing length: 10.0 mm.

**Figure 48.**
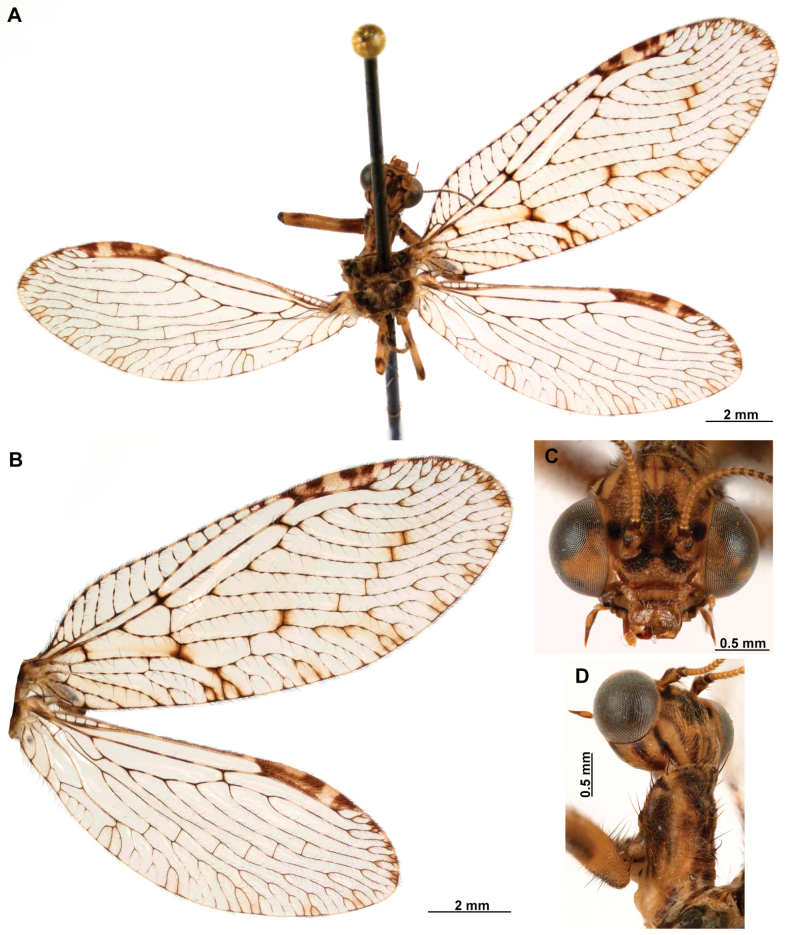
*Plegalachesis* Ardila-Camacho & Contreras-Ramos, sp. nov. **A** male habitus, dorsal **B** wings **C** head, frontal **D** pronotum, lateral.

***Coloration*** (Fig. 48). ***Head*.** Vertexal region pale brown, with lateral dark brown markings extending from occiput to toruli forming a V-shaped pattern, with brown setae; area adjacent to occipital ridge dark brown; supra-antennal area dark brown with lateral pale brown spots, with brown setae; occiput and postgena pale brown with dark brown stripe each. Antennal scape pale brown with longitudinal dark bands, entire surface with pale brown setae; pedicel brown, flagellum pale brown on proximal 1/3, gradually becoming darker towards the apex, except two preapical flagellomeres pale. Frons pale brown with dark brown marks, adjacent to toruli; clypeus pale brown; labrum pale brown; mandible pale brown with amber apex; maxilla pale with brown areas, palpus with first two palpomeres brown, third with basal ½ brown and distal ½ pale, fourth palpomere pale, fifth palpomere brown; labium pale, except prementum pale brown and palpus brown, palpimacula pale brown. ***Thorax*.** Pronotum pale brown with darker areas on medial region, area adjacent to lateral margins brown; episternum pale brown with dark brown marks, postfurcasternum pale with posterolateral corners brown. Mesonotum brown with pale areas; metanotum brown with anterolateral pale marks; pre-episternum brown; pteropleura pale with brown areas, primarily on episternum, setae mostly pale brown. ***Foreleg*.** Coxa pale brown with brown marks on base and apex of anterior surface, setae mostly pale brown, but posterior surface with dark brown setae on proximal region. ***Mid- and hind leg*.** Coxae pale with brown marks, setae mostly pale brown; trochanter pale with dark brown mark on anterior surface. Femora and tibiae of both legs pale with brown rings; tibial spurs brown. Tarsi pale brown with slightly darker setae; pretarsal claws brown. ***Wings*.** Forewing mostly hyaline; membrane surrounding crossveins, first branch of CuA and apex of CuP, and apical twigging of longitudinal veins amber; pterostigma brown with pale areas; major veins, subcostal veinlets, and wing margin alternating pale and brown. Hind wing hyaline; pterostigma brown with pale areas; C, Sc, and subcostal veinlets mostly pale brown, remaining longitudinal veins, and wing margin alternating pale and brown; crossveins brown. ***Abdomen*.** Cleared.

***Morphology*** (Fig. 48). ***Head*.** Diamond-shaped in frontal view, rugose; vertexal region raised above compound eyes, with lateral rows of reclined setae; area surrounding coronal suture glabrous, with muscle insertion mark; coronal suture distinct; medial area of ​​supra-antennal region moderately raised, with fine, reclined setae; paraocular area concave. Antenna submoniliform, short; scape nearly two times as long as wide, slightly distally expanded, mostly with fine, short setae, some thickened dorsally near distal margin; pedicel slightly longer than wide; flagellum not dorsoventrally flattened, with 35 flagellomeres, which are as wide as long on proximal ¼, and slightly longer than wide on the rest of flagellum; all articles with medial ring of fine, short setae. Compound eye hemispherical, as wide as ½ of the interocular distance at torulus level. Frons and clypeus narrow, with fine and short setae. Labrum pentagonal with thin, short setae; maxillary palpus with first palpomere as long as wide, second 1.2× as long as wide, third palpomere 3× as long as wide, fourth palpomeres 2.5× as long as wide, fifth slightly longer than third, all with short and thin setae; mentum with long, thin setae; labial palpus with first palpomere two times as long as wide, second palpomere 4× as long as wide, third palpomere as long as second, palpimacula narrowly ovoid. ***Thorax*.** Pronotum as long as wide, with raised anterior margin, medial and posterior regions; outgrowths covered with thick setae arising flush the pronotal surface; remaining surface with fine, short setae. Mesonotum 1.5× as wide as long, with scattered, thick setae on medial area. Metanotum ~ 2× as long as wide, glabrous. Pteropleura with thin, short setae, a few thickened on mesanepisternum. ***Foreleg*.** Coxa as long as femur, cylindrical, posterior surface with intersperse fine, short setae and thickened, long setae; anterior surface mostly with fine, short setae. ***Mid- and hind leg*.** Coxa with long, thin setae; femora and tibiae with abundant, short, thin setae; tibial spurs short; hind leg longer than midleg, tibia, 1.5× as long as femur; tarsi with fine and short setae, except on distal margin of plantar surface with lateral groups of 4–6 thickened setae; on both legs, basitarsus 3.5 times as long as wide, second tarsomere 1.2× as long as wide; third and fourth tarsomeres as long as wide; fifth tarsomere two times as long as wide. ***Wings*.** Forewing oval, trichosors present along margin except on wing base; venation setose; costal space proximally expanded, humeral vein branched, 16 subcostal veinlets; pterostigma elongated, narrow, veinlets not distinct; subcostal space with single crossvein, medially located; Sc vein abruptly posteriorly bent at proximal pterostigma margin to merge with RA; radial space with two crossveins; *rarp2* curved with four RP branches; three veins arising from *rarp1*; M vein basally fused with RA; RP base, close to divergence of M and R; M forked nearly opposite to RP origin, 1 r-m connecting RP base and M fork, forming a trapezoidal cell; four gradate crossveins present. Cubitus deeply forked; CuP basally angled and approaching A1, distally forked slightly beyond the level of separation of M and R; A1 simple, ending on posterior margin at level or slightly beyond CuP fork, A2 forked slightly beyond CuP angle. Hind wing smaller and narrower than forewing, oval; costal space narrow and reduced, with six veinlets; C and Sc fused at ¼ of wing length, Sc vein abruptly curved posteriorly at proximal margin of pterostigma to merge RA; pterostigma elongated, narrow, straight, composed of poorly defined veinlets; radial space with single crossvein, oblique; three veins arising from *rarp1*, two from *rarp2*. 1r-m sigmoid, connecting the stems of M and RP. Media forked beyond R fork. Cubitus deeply forked, intracubital crossvein subparallel to longitudinal wing axis; CuA sinuous, first branch candelabrum-shaped, spur vein absent; CuP not touching A1, strongly anteriorly bent at distal 1/3, pectinate; single crossvein on cubitoanal space; A1 simple ending on wing margin at 1r-m level; A2 simple, short, and curved. ***Abdomen*.** Medially expanded, setae on tergites, scattered, thin, and short; tergites subquadrate. Sternites rectangular, with abundant fine and short setae.

**Figure 49.**
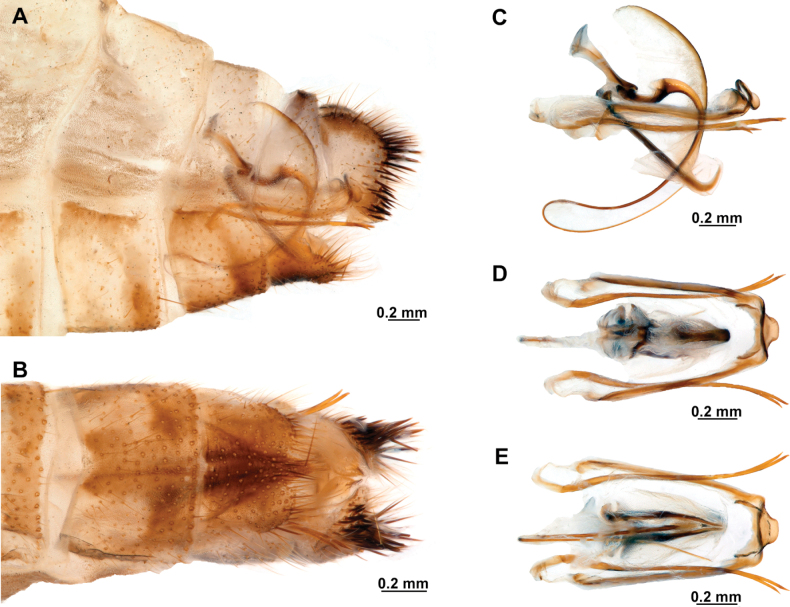
*Plegalachesis* Ardila-Camacho & Contreras-Ramos, sp. nov. **A** male terminalia, lateral **B** same, ventral **C** male genitalia, lateral **D** same, dorsal **E** same, ventral.

***Male genitalia*** (Fig. 49). Tergite IX medially narrower than laterally; lateral margin bluntly quadrangular. Sternum VIII rectangular; sternite IX trapezoidal in ventral view, covered with abundant, thin, long setae near posterior margin; posteromedially with elongate, narrow, acuminate process, which is dorsally canaliculated; in lateral view trapezoidal, apex reaching posterior margin of ectoproct. Gonocoxites IX thin, straight, long; base laterally curved, connected to gonocoxites XI with a membrane; apex bifid, with two thin processes. Ectoproct trapezoidal, covered with prominent, stout setae on posterior surface; ventrally with lightly sclerotized, ovoid lobe, which is continuous with ventromedial sclerotized, short sulcus and covered with microtrichia. Gonocoxites X forming a short, thickened, ventrally canaliculate sclerite, anterior apex expanded; posterior apex with dorsal processes connected to gonostyli X and short ventrolateral processes connected to gonapophyses X with a membrane; gonostyli X with thickened base forming a right angle and with two lateral processes, the rest of the structure, thin, whip-shaped, ventrally curved, and anteriorly incurved, not protruding from abdomen. Gonapophyses X rod-shaped, narrow, with posterior apex dorsally curved; gonapophyses arranged in a V-shaped structure, joined by an enlarged membrane covering the gonostyli X base; this membrane is medially slightly sclerotized. Gonocoxites XI thin, U-shaped, medial lobe complex and elaborated with two differentiated parts: a dorsal, curved lobe; ventral part as a convex area, ventral surface forming a concave covering; between these parts narrow, less sclerotized, hyaline area is present; lateral arms of gonocoxites XI straight, with anterior apex ventrally curved. Hypandrium internum arched.

##### Distribution.

Guatemala (Guatemala).

##### Remarks.

This remarkable species is solely known from its type locality in Guatemala (Guatemala). In the phylogeny of the subfamily, this species was recovered within a clade of species restricted to Central America and Mexico as sister to *P.obtusa* + (*P.oswaldi + P.spinosa*). The wing coloration pattern of this species is similar to that of *P.spinosa*, yet the lack of the sunken supra-antennal area with protuberant, setose regions as in *P.spinosa* rapidly separate both species. The configuration of the male genitalia of *P.Lachesis* is remarkably similar to that of *P.stangei* and *P.vangiersbergenae* yet the ectoproct set with abundant stout setae on the posterior surface and the bifid male gonocoxites IX distinguish this species.

#### ﻿﻿ Plega longicornis

Taxon classificationAnimaliaNeuropteraRhachiberothidae

Ardila-Camacho & Contreras-Ramos sp. nov.

https://zoobank.org/EC58BFA2-A8E5-4719-AF26-F36A5569D028

Figs 50 , 51

##### Type locality.

Mexico, **Jalisco**: Reserva de la Biósfera Chamela-Cuixmala, Estación de Biología Chamela, sendero Tejón (850 m), 19°30.288'N, 105°02.812'W, 58 m, 23 Nov. 2021, aerial net, A. Gómez, J. Almaráz & H. Juárez leg.

##### Material examined.

***Holotype*** male, pinned. Original label: “Mexico, **Jalisco**, Reserva de la Biósfera Chamela-Cuixmala, Estación de Biología Chamela, sendero Tejón (850 m), 19°30.288'N, 105°02.812'W, 58 m, 23 Nov. 2021, red aérea, A. Gómez, J. Almaráz & H. Juárez leg.” CNIN. ***Paratypes*.** Mexico • 1 ♂; **Jalisco**, Reserva de la Biósfera Chamela-Cuixmala, Estación de Biología Chamela; 19°29.905'N, 105°02.667'W; 100 m; 23 Nov. 2021; A. Gómez, J. Almaráz & H. Juárez leg.; red aérea, atraídos a la luz; CNIN. • 1 ♂; same data as for preceding; EBCh, Sendero Chachalaca; 19°29.959'N, 105°02.311'W; 52 m; A. Ardila & A. Gómez-Jácome leg.; trampa de luz, 21:00 h; CNIN.

##### Other material.

Mexico – **Colima** • 1 ♀; 5.8 Km NO Ixtlahuacán; 19°01'15.7"N, 103°46'37.8"W; 354 m; 26 Apr. 2006; S. Zaragoza leg.; CNIN. • 1 ♀; 5.1 Km NW Ixtlahuacán; 19°.01'18.9"N, 103°46'19.4"W; 400 m; 17 Feb. 2007; S. Zaragoza, F.A. Noguera, E. González, E. Ramírez, L. Salas leg.; bosque tropical caducifolio; CNIN. – **Jalisco** • 1 ♂; Reserva de la Biósfera Chamela-Cuixmala, Estación de Biología Chamela, sendero Ardilla (800 m); 19°30.425'N, 105°02.676'W; 50 m; 23 Nov. 2021; A. Gómez, J. Almaráz & H. Juárez leg.; red aérea; CNIN. • 1 ♂ 1 ♀; Plan de Barrancas; 24 Mar. 1962; F.D. Parker & L.A. Stange leg.; FSCA. • 1 ♂; Chamela; 28 Mar. 1988; E. Ramírez leg.; CNIN. • 1 ♀; Estación de Biología Chamela; 25 Apr. 1984; E. Ramírez leg.; light trap; CNIN. – **Nayarit** • 1 ♂; P.H. Aguamilpa, San Rafael, Arroyo de la Virgen; 01 Nov. 1991; R. Barba & E. Barrera leg.; CNIN. • 1 ♀; Estación Microondas, 8.3 Km W de la presa hidroeléctrica “El Cajón”; 21°25'40"N, 104°31'43"W, 902 m; 16 Nov. 2009; S. Zaragoza, F. Noguera, E. Ramírez leg.; bosque tropical caducifolio, trampa de luz 4; CNIN.

##### Diagnosis.

This species is separated from its closer relatives, i.e., *P.spinosa*, *P.acevedoi*, and *P.oswaldi* based on the elongated antennal scape, which is 3.5 times as long as wide at middle, with dorsal surface set with stout setae near distal margin. Like in *P.acevedoi*, the area adjacent to frontal sutures is slightly sunken and the ​​supra-antennal is moderately raised laterally, with abundant, fine, reclined setae. In addition, the antennal flagellum has 2–4 pale, preapical flagellomeres. The presence of a fuscous stripe before a whitish apical margin on the hind wing apex is a distinctive feature of this species. On the forefemur the posterior surface is pale with brown dots, while the anterior surface is dark brown on distal ½, but progressively changes to pale towards base. On the male genitalia, the gonocoxite IX lacks digitiform processes, and the ectoproct is set with spine-shaped setal bases on the posteroventral surface, the gonostyli X are posteriorly recurved at mid length before protruding from the abdomen, with the apex abruptly narrowed and pointed; the ventral part of the medial lobe of the gonocoxites XI forms a ventrally projected, curved, pointed process, set with two lateral, fin-shaped processes on its base. On the female genitalia, the gonocoxites + gonapophyses VIII form a tubular, medial process whose apex is bifid, the spermatheca is short and simple, with undifferentiated proximal and medial sections, and there are blunt diverticula on the proximal-most part and on the apex.

##### Etymology.

The specific epithet of this species is a composite of the Latin words *longus* (extended or prolonged) and *cornus* meaning horn, in reference to the noticeably elongated antennal scape of this species.

##### Description.

***Measurements*.** Male (*n* = 5). Forewing length: 7.9–12.6 mm; Hind wing length: 6.3–10.6 mm. Female (*n* = 1). Forewing length: 8.4 mm; Hind wing length: 6.9 mm.

**Figure 50.**
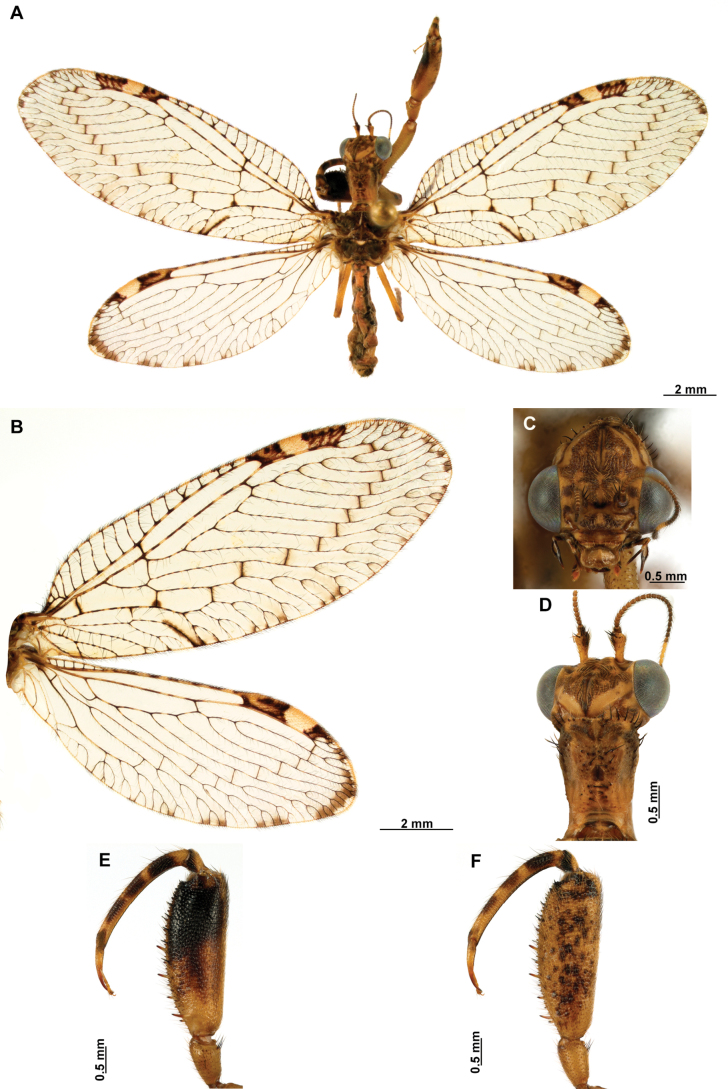
*Plegalongicornis* Ardila-Camacho & Contreras-Ramos, sp. nov. **A** male habitus, dorsal **B** wings **C** head, frontal **D** pronotum, dorsal **E** forefemur, anterior surface **F** same, posterior surface.

***Coloration*** (Fig. 50). ***Head*.** Vertexal region pale, with lateral brown marks extending from occipital ridge to supra-antennal area, embracing pale areas and forming an inverted V-shaped pattern, with pale brown setae; area adjacent to occipital ridge brown; supra-antennal area brown with pale lateral areas, with pale brown setae; occiput and postgena brown with pale stripes. Antennal scape pale, with dark brown areas on inner and ventral surfaces, apex with dark brown setae; pedicel brown; flagellum brown, with 2–4 pale, preapical flagellomeres. Frons pale with brown areas. Clypeus and labrum pale with pale brown areas; mandible pale with dark brown corners, apex dark amber; maxilla pale with brown areas, palpus mostly dark brown; labium pale with brown areas, palpus mostly dark brown, palpimacula pale brown. ***Thorax*.** Pronotum pale brown, with darker anterolateral areas, medial region and anterior margin with small pale areas; a dark brown muscle attachment mark is present on the center; episternum brown; postfurcasternum brown with brown suffusions. Mesonotum mostly brown; metanotum brown with cream anteromedial, small area; pre-episternum brown; pteropleura with brown marks and pale areas, pale brown setae present. ***Foreleg*.** Coxa tan to pale greenish brown, with brown mark at apex of anterior surface, with some dark brown setae at basal region; trochanter with same color as coxa, dorsally with some dark brown setae. Femur posterior surface pale with brown dots; anterior surface dark brown on distal ½, progressively changing to pale towards base. Tibia dashed with pale and brown. Basitarsus pale at basal ½, changing to amber towards the apex; remaining tarsomeres pale brown. ***Mid- and hind leg*.** Coxae and trochanter pale with brown marks; femora and tibiae pale with brown rings; tarsi pale, distal margins of eutarsal tarsomeres with dark brown setae on plantar surface; remaining surface with pale brown setae. ***Wings*.** Forewing mostly hyaline; membrane surrounding forks of longitudinal veins, crossveins, first branch of CuA and apex of CuP fuscous; posterior and apical margins with intermittent, fuscous areas between apical branches of longitudinal veins; pterostigma brown with small, anterior pale areas, and larger cream medial area; major veins, subcostal veinlets, and wing margin alternating pale and brown; crossveins dark brown. Hind wing hyaline, adjacent area to posterior margin with intermittent fuscous areas between apical branches of longitudinal veins; wing apex with a fuscous stripe before whitish apical margin; pterostigma brown with small pale areas, and wide, cream, preapical region; longitudinal veins alternating pale and brown; crossveins brown, except sigmoid 1r-m bicolor; wing margin alternating pale and brown. ***Abdomen*.** Tergites pale brown. Pleural membrane dark brown, except near dorsal and ventral margins pale. Sternites pale with lateral, brown stripe on each side.

***Morphology*** (Fig. 50). ***Head*.** Diamond-shaped in frontal view, rugose; vertexal region raised above compound eyes, with lateral rows of reclined setae; area surrounding coronal suture glabrous, with muscle insertion mark; coronal suture distinct, area adjacent to frontal sutures slightly sunken; ​​supra-antennal moderately raised laterally, with abundant, fine, reclined setae; paraocular area concave. Antenna filiform, short; scape conspicuously elongated and conical, 3.5 times as long as wide at middle, dorsal surface with stout setae near distal margin; pedicel slightly longer than wide; flagellum not dorsoventrally flattened, with 27–36 flagellomeres, those of flagellar base as wide as long, then changing to slightly longer than wide on the rest of flagellum; all articles with medial ring of fine, short setae. Compound eye hemispherical, as wide as ½ of the interocular distance at torulus level. Frons and clypeus narrow. Labrum pentagonal with thin, short setae; maxillary palpus with first palpomere as long as wide, second 1.2× as long as wide, third palpomere 4× as long as wide, fourth palpomere 3× as long as wide, fifth palpomere slightly longer than third; mentum with long, thin setae; labial palpus with first palpomere two times as long as wide, second palpomere 4× as long as wide, third slightly shorter than second, palpimacula narrowly ovoid. ***Thorax*.** Pronotum slightly longer than wide, with raised anterior margin, medial and posterior regions; a muscle attachment mark is present in the middle of the pronotum; raised regions and anterior and margins covered with pedicellate, thickened setae. Mesonotum two times as wide as long, with scattered, thick, pedicellate setae on medial area. Metanotum ~ 2× as long as wide, glabrous. Pteropleura with short, thin, setae a few thickened on mesanepisternum. ***Foreleg*.** Coxa nearly as long as femur, cylindrical, anterior and posterior surfaces with pedicellate, thick setae on basal region; trochanter trapezoidal, with thin and short setae, except on dorsal and anterior surfaces with some thickened, pedicellate setae; anterior surface with protuberant area. Femur robust, covered with abundant, fine, short setae arising from protuberant bases; closing surface with posteroventral row of processes composed two medially located, primary processes, and one tertiary process between them; proximal portion of the row with sub-basal secondary or tertiary process and basal secondary or tertiary process; the rest of the row with numerous tubercle-shaped processes, and stinger-shaped setae; distal ½ slightly carinated, composed of tubercle-shaped specializations and stinger-shaped setae; adjacent row of thickened setae with globular base present on distal ¼. Anteroventral row of processes reduced to proximal ½; it is composed of tubercle-shaped specializations and stinger-shaped setae; the basal-most primary, curved process is present; distal portion composed of a few tubercle-shaped processes; adjacent row of thickened setae with globular base present on distal ½. Tibia almost as long as femur, curved, with thin, short setae; ventral surface keeled with prostrate setae; a patch of clavate setae apically on anterior surface is present. Basitarsus with lanceolate process reaching the middle of fourth tarsomere; clavate setae present proximally on anterior surface; ventrally with single row of prostrate setae; second tarsomere nearly 7× as long as wide; third tarsomere as long as wide, fourth tarsomere two times as long as wide. ***Mid- and hind leg*.** Coxae with short and thin setae, some thickened; trochanter with short, thin setae; femora with interspersed fine, long, verticillate setae, and fine, short, reclined setae; tibial spurs short; tibiae with short and thin setae. Hind leg longer than midleg; tibia, 1.5× as long as femur; tarsi with fine and short setae, except on distal margin of plantar surface with lateral groups of 3–5 thickened setae; on both legs, basitarsus 4× as long as wide, second tarsomere 1.2× as long as wide; third and fourth tarsomeres as long as wide; fifth tarsomere two times as long as wide. ***Wings*.** Forewing oval, trichosors present along margin except on wing base; venation setose; costal space proximally expanded, humeral vein branched, 8–16 subcostal veinlets; pterostigma trapezoidal, elongated, narrow, straight, with distinct veinlets; subcostal space with single crossvein, medially located; Sc vein abruptly posteriorly bent at proximal pterostigma margin to merge the RA; radial space with two crossveins; *rarp2* gently curved with three or four RP branches; four veins arising from *rarp1*; M vein basally fused to RA; RP base widely separated from divergence of M and R; M forked nearly opposite to RP origin, 1 r-m connecting RP base and MA stem, forming a trapezoidal cell; six or seven gradate crossveins present. Cubitus deeply forked; CuP basally angled and approaching A1, distally forked opposite to the level of separation of M and R; A1 apically forked, ending on posterior margin slightly beyond the level of CuP fork, A2 forked slightly beyond to CuP angle level. Hind wing smaller and narrower than forewing, oval; costal space narrow and reduced, with six veinlets; C and Sc fused at proximal ⅕ of wing length, Sc vein abruptly curved posteriad at proximal margin of pterostigma to merge RA; pterostigma elongated, narrow, straight, composed of well-defined veinlets; radial space with single crossvein, oblique; three veins arising from *rarp1*, one or two from *rarp2*. 1r-m sigmoid, connecting the stems of M and RP. Media forked slightly beyond R fork. Cubitus deeply forked, intracubital crossvein subparallel to longitudinal wing axis; CuA sinuous, first branch candelabrum-shaped, spur vein absent; CuP not touching A1, strongly anteriorly bent at distal 1/3, pectinate; two crossveins on cubitoanal space present; A1 simple, ending at wing margin at 1r-m stem level; A2 simple, short, and curved. ***Abdomen*.** Tergites quadrangular with anterolateral elongated scars; sternites rectangular.

**Figure 51.**
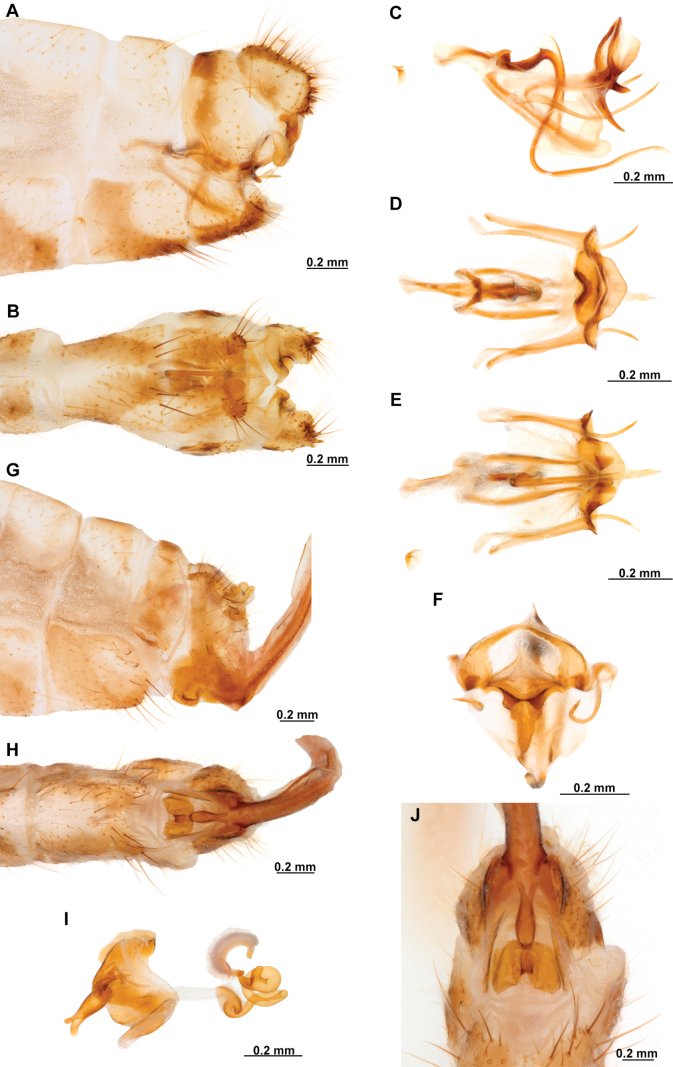
*Plegalongicornis* Ardila-Camacho & Contreras-Ramos, sp. nov. **A** male terminalia, lateral **B** same, ventral **C** male genitalia, lateral **D** same, dorsal **E** same, ventral **F** same, caudal **G** female terminalia, lateral **H** same, ventral **I** spermatheca **J** gonapophyses VIII of female, ventral.

***Male genitalia*** (Fig. 51A–F). Tergite IX medially narrower than laterally; lateral margin rounded. Sternum VIII rectangular; sternite IX trapezoidal in ventral view; posterior margin with medial lobe not developed, but corresponding area dorsally canaliculated; in lateral view trapezoidal, not reaching posterior margin of ectoproct. Gonocoxites IX thin, sinusoid, short; base spatulated; apex pointed, without processes. Ectoproct trapezoidal, posteroventral surface set with spine-shaped setal bases, remaining surface with long, pedicellate setae; anteroventrally with enlarged, rounded, sclerotized lobe, continuous with ventromedial sclerotized, curved sulcus. Gonocoxites X forming a short, straight, ventrally canaliculate sclerite; anterior apex, expanded; posterior apex with dorsal processes connected to gonostyli X and ventrolateral processes connected to gonapophyses X with a membrane; gonostyli X with thickened and concave base with two lateral processes, the rest of the structure, ventrally curved, and posteriorly recurved at mid length before protruding from abdomen, apex abruptly narrowed and pointed. Gonapophyses X rod-shaped, gently curved; gonapophyses sub-parallel, joined by a membrane covering the gonostyli X base. Gonocoxites XI U-shaped, medial lobe complex and elaborated with two differentiated parts: dorsal part as an arch, with lateral parts expanded; ventral part with prominent, ventrally projected, curved, pointed process, set with two lateral, fin-shaped processes on its base; between these parts a pentagonal, less sclerotized, hyaline area is present, which forms a triangular outgrowth; lateral arms of gonocoxites XI short, thin, sigmoid, with anterior apex bent ventrad. Hypandrium internum triangular, with lateral fins.

***Female genitalia*** (Fig. 51G–J). Sternum VII subquadrate with posteromedian concavity. Tergite VIII narrower medially than laterally, encircling the spiracle of the segment; lateral margin quadrangular. Gonocoxites VIII not discernible, forming a complex with the gonocoxites VIII. Gonocoxites + gonapophyses VIII as two lateral oval plates which form a tubular, medial process whose apex is bifid; lateral part of gonapophyses VIII as a rounded plate folded beneath tergite IX + ectoproct. Tergite IX + ectoproct D-shaped. Gonocoxites IX elongated and narrow, nearly as long as the last five abdominal segments; gonapophyses IX not discernible. Bursa copulatrix short, funnel-shaped, with proximal-most part sclerotized, the rest membranous and striated. Spermatheca short and simple; proximal and medial sections undifferentiated, thin; proximal-most forming a blunt diverticulum; distal section only progressively slightly expanded, forming a blunt diverticulum; fertilization canal duct with proximal portion triangular, concave; fertilization canal elongated a narrow, C-shaped, covered with microfilaments.

##### Distribution.

Mexico (Colima, Jalisco, Nayarit).

##### Remarks.

This species is closely related to *P.spinosa* and *P.acevedoi* from central Mexico due to modifications of the antennal scape and supra-antennal area, coloration pattern of forefemur and wings, as well as configuration of male and female genitalia. *Plegalongicornis* is geographically isolated from the aforementioned species, being apparently restricted towards the western coast of central Mexico (Jalisco, Nayarit and Colima). This new species belongs to a group of species together with *P.oswaldi* and *P.obtusa*.

#### ﻿﻿ Plega megaptera

Taxon classificationAnimaliaNeuropteraRhachiberothidae

Ardila-Camacho & Contreras-Ramos sp. nov.

https://zoobank.org/3E523FFC-6121-4DE2-8B93-D7195E55D47E

Figs 52 , 53

##### Type locality.

Mexico, **Chihuahua**: 25 mi. SW. Tejab[a]n along Arique [Urique] River, 20–23 May. 1991, R.E. Stecker leg.

##### Material examined.

***Holotype*** male, pinned. Original label: “Mexico, **Chihuahua**, 25 mi. SW. Tejabon along Arique [Urique] River, 20–23 May. 1991, R.E. Stecker leg.” CAS. ***Paratypes*.** Mexico • 1 ♂; **Chihuahua**, 25 mi. SW. Tejab[a]n along Arique [Urique] River; 20–23 May. 1991; R.E. Stecker leg.; *Plegafratercula*, det. N. Penny, 1991; CAS. • 3 ♂; 25 mi. SW. Tejab[a]n along Arique [Urique] River; 20–23 May. 1991; R.E. Stecker leg.; *Plegafratercula*, det. N. Penny, 1991; UCD. • 1 ♂; 10 mi. SW. Tejab[a]n along Urique River; 11–15 May. 1991; R.E. Stecker leg.; UCD. • 1 ♂; Sierra Mts., La Bufa; 900 m; 07 Jul. 1972; D. Giuliani leg.; *Plegabanksi*, det. N. Penny, 1985; CAS.

##### Diagnosis.

This species has the basal antennal flagellomeres two times as long as wide. It is similar to *P.sonorae*, but the remarkably enlarged, oval wings of *P.megaptera* separate it from the former. Furthermore, this species has the gonocoxite IX long, thin, and sigmoid, with the posterior apex equipped with 4–6 processes of different sizes, arranged as a hand.

##### Etymology.

The specific epithet of this species is a composite of the Greek *μέγας* (*mégas*), meaning great or large and *πτερόν* (*pterón*) meaning wing, in allusion to the distinctly enlarged wings of this species.

##### Description.

***Measurements*.** Male (*n* = 3). Forewing length: 11.2–19.5 mm; Hind wing length: 8.8–15.6 mm.

**Figure 52.**
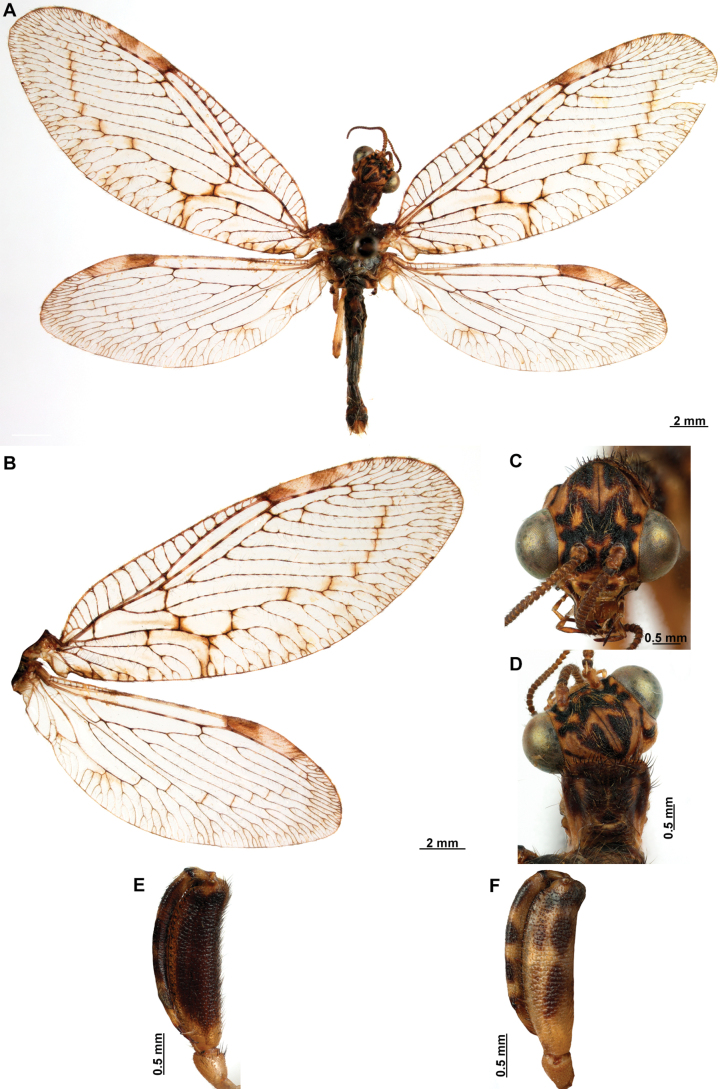
*Plegamegaptera* Ardila-Camacho & Contreras-Ramos, sp. nov. **A** male habitus, dorsal **B** wings **C** head, frontal **D** pronotum, dorsal **E** forefemur, anterior surface **F** same, posterior surface.

***Coloration*** (Fig. 52). ***Head*.** Vertexal region pale, with lateral dark brown markings extending from occiput to toruli, area adjacent to occipital ridge dark brown, area adjacent to coronal suture with an arrow-shaped pale area; supra-antennal area dark brown with lateral oval, pale areas; pale brown setae present; occiput pale with brown spot, postgena pale to pale brown. Antennal scape pale brown with darker apex and dorsal surface, entire surface with pale brown setae; pedicel dark brown; flagellum brown with darker setae. Frons brown with darker areas below toruli. Clypeus pale brown with darker lateral areas; labrum pale brown; mandible pale brown with dark brown corners, dark amber at apex; maxilla brown, palpus darker, with brown setae; labium brown with darker palpus, brown setae present; palpimacula brown. ***Thorax*.** Pronotum with extensive brown marks with pale areas, most of the setae dark brown; episternum pale brown with dark margins; postfurcasternum pale with posterodorsal corner dark brown. Mesonotum mostly dark brown, area adjacent to sutures pale brown, setation mostly dark brown; metanotum mostly dark brown with pale anteromedian area; pre-episternum dark brown; pteropleura mostly dark brown with pale on margins of sclerites, setation mostly pale brown, except on anepisternum dark brown. ***Foreleg*.** Coxa mostly pale, with brown spots on base and apex, interspersed pale, and dark brown setae with brown base; trochanter pale, setation pale brown, except dorsally with some dark brown setae. Femur posterior surface pale with three weakly to well defined brown spots on medial area, apex brown; anterior surface mostly dark brown, except, base and apex pale; major process pale with brown apex. Tibia dashed with pale and dark brown, except on anterior surface nearly completely dark brown. Basitarsus pale brown proximally, changing to amber towards the apex, clavate setae pale brown; second tarsomere brown, third and fourth tarsomere paler. ***Mid- and hind leg*.** Coxae pale with brown areas, setae pale brown; trochanter of both legs pale with brown suffusion. Femur of mid-leg pale with sub-basal brown area and dark brown apex, tibia pale with medial an apical parts brown, interspersed pale and dark brown setae present; femur of hind leg pale with brown apex, tibia pale with brown suffusion on base and apex, interspersed pale and dark brown setae present; tibial spurs brown. Tarsi pale brown, except fifth tarsomere darker; first four tarsomeres with dark brown setae, laterally on distal margin of plantar surface; remainder surface with interspersed pale and dark brown setae; pretarsal claws brown. ***Wings*.** Forewing mostly hyaline; membrane surrounding crossveins, first branch of CuA, and terminal forks of longitudinal veins amber; pterostigma brown with pale medial area; major veins and wing margin alternating pale and brown, subcostal veinlets mostly brown. Hind wing hyaline, amber on area adjacent to first branch of CuA; pterostigma brown with pale preapical area; C, subcostal veinlets and Sc with extensive pale areas, remainder longitudinal veins alternating pale and brown; crossveins brown, except sigmoid 1r-m, bicolor; wing margin alternating pale and brown. ***Abdomen*.** Tergites and sternites brown with pale areas; setation mostly brown.

***Morphology*** (Fig. 52). ***Head*.** Diamond-shaped in frontal view, rugose; vertexal region raised above compound eyes, with lateral rows of reclined setae; area surrounding coronal suture glabrous, with muscle insertion mark; coronal suture distinct; medial area of ​​supra-antennal region slightly raised, with fine, reclined setae; paraocular area concave. Antenna submoniliform, short; scape 1.5× as long as wide, slightly distally expanded, with fine, short setae; pedicel as long as wide; flagellum dorsoventrally slightly flattened, with 49–56 flagellomeres, those of proximal ¼ of the flagellum two times as wide as long; as long as wide on the rest of flagellum; all articles with medial ring of fine, short setae. Compound eye hemispherical, as wide as ½ of the interocular distance at torulus level. Frons and clypeus narrow, with fine and short setae. Labrum pentagonal with thin, short setae; maxillary palpus with first palpomere as long as wide, second 1.2× as long as wide, third palpomere 3.5 times as long as wide, fourth palpomere 2.5× as long as wide, fifth as long as third, all with short and thin setae; mentum with long, thin setae; labial palpus with first palpomere 2.5× as long as wide, second palpomere 4× as long as wide, third palpomere slightly longer than second, palpimacula narrowly ovoid. ***Thorax*.** Pronotum as long as wide, with raised anterior margin, medial and posterior regions; outgrowths covered with pedicellate, thick setae; remaining surface with fine, short setae. Mesonotum slightly wider than long, with abundant, thick, pedicellate setae on medial area. Metanotum ~ 2× as wide as long, scutellum with pedicellate, thickened setae. Pteropleura with short, thin setae, thicker on anepisternum. ***Foreleg*.** Coxa as long as femur, cylindrical, anterior and posterior surfaces with pedicellate fine or thick setae of different sizes; trochanter trapezoidal, with thin and short setae, except on dorsal and anterior surfaces with some thickened setae; anterior surface with protuberant area. Femur robust, covered with abundant, fine, short setae; closing surface with posteroventral row of processes composed two medially located, primary processes, with tertiary process between the primary ones; proximal portion of the row with two secondary processes; the rest of the row with abundant tubercle-shaped processes, stinger-shaped setae, and some tertiary processes; distal portion raised composed of tubercle-shaped specializations and stinger-shaped setae; adjacent row of thickened setae with globular base present on distal ¼–⅕. Anteroventral row of processes reduced to proximal ½; it is composed of tubercle-shaped specializations and stinger-shaped setae; the basal-most primary, curved process is present; distal portion composed of a few tubercle-shaped processes; adjacent row of thickened setae with globular base present on distal ⅘. Tibia almost as long as femur, curved, with thin, short setae; ventral surface keeled with prostrate setae; a patch of clavate setae apically on anterior surface is present. Basitarsus with lanceolate process reaching the middle of fourth tarsomere; clavate setae present proximally on anterior surface; ventrally with single row of prostrate setae; second tarsomere nearly 6× as long as wide; third tarsomere as long as wide, fourth tarsomere 2.5× as long as wide. ***Mid- and hind leg*.** Coxae and trochanter with short, thin setae; femora and tibiae with interspersed fine, short setae and a few thickened setae; tibial spurs short; hind leg longer than midleg, tibia, 1.5× as long as femur; tarsi with fine and short setae, except on distal margin of plantar surface with lateral groups of 5–7 thickened setae; on both legs, basitarsus 3.5 times as long as wide, second tarsomere 1.2× as long as wide; third and fourth tarsomeres as long as wide; fifth tarsomere two times as long as wide. ***Wings*.** Forewing narrowly oval, trichosors present along margin except on wing base; venation setose; costal space proximally expanded, humeral vein branched, 16–20 subcostal veinlets; pterostigma elongated, narrow, straight, with distinct veinlets; subcostal space with single crossvein, medially located; Sc vein abruptly posteriorly bent at proximal pterostigma margin to merge with RA; radial space with two or three crossveins; *rarp2* gently curved with three or four RP branches; three or four veins arising from *rarp1*; M vein basally fused with RA; RP base, widely separated from divergence of M and R; M forked nearly opposite to RP origin, 1 r-m connecting RP base and M fork, forming a trapezoidal cell; 4–7 gradate crossveins present. Cubitus deeply forked; CuP basally angled and approaching A1, distally forked slightly beyond the level of separation of M and R; A1 apically forked, ending on posterior margin at level of CuP fork, A2 forked slightly beyond CuP angle level. Hind wing smaller and narrower than forewing, narrowly oval; costal space narrow and reduced, with 7–9 veinlets; C and Sc fused at ¼ of wing length, Sc vein abruptly curved posteriad at proximal margin of pterostigma to merge RA; pterostigma elongated, slightly subapically expanded, straight, composed of well-defined veinlets; radial space with single crossvein, oblique; three or four veins arising from *rarp1*, one or two from *rarp2*. 1r-m sigmoid, connecting the stems of M and RP. Media forked beyond R fork. Cubitus deeply forked, intracubital crossvein subparallel to longitudinal wing axis; CuA sinuous, first branch candelabrum-shaped, spur vein absent or present; CuP not touching A1, strongly anteriorly bent at distal 1/3, pectinate; two crossveins on cubitoanal space; A1 apically forked, ending on wing margin at 1m-cu level; A2 apically forked, short, and curved. ***Abdomen*.** Cylindrical to medially expanded, setae on tergites, scattered, thin, and short, gradually longer and more abundant towards terminal segments; tergites subquadrate, tergites III-VII with elongated anterolateral scars. Sternites rectangular, those of segments III–VII with posterolateral narrow scars; with abundant fine and short setae.

**Figure 53.**
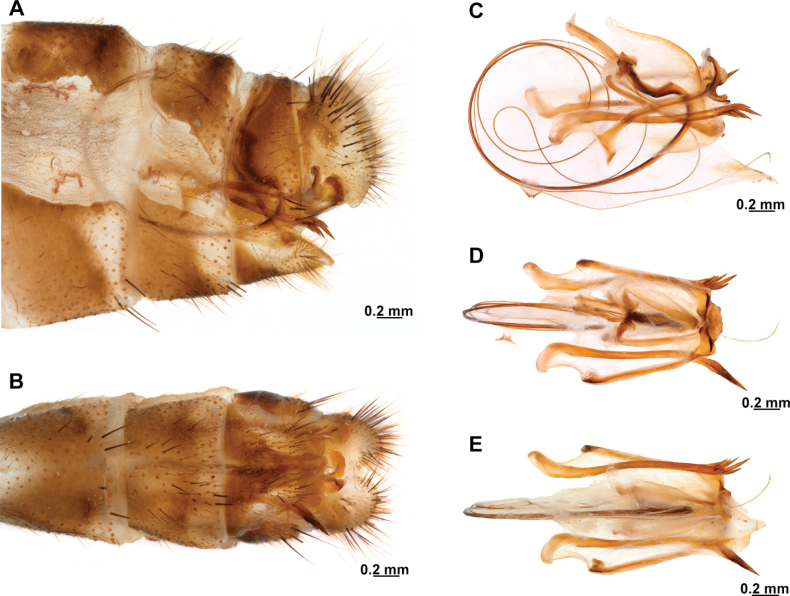
*Plegamegaptera* Ardila-Camacho & Contreras-Ramos, sp. nov. **A** male terminalia, lateral **B** same, ventral **C** male genitalia, lateral **D** same, dorsal **E** same, ventral.

***Male genitalia*** (Fig. 53). Tergite IX medially narrower than laterally; lateral margin rounded, posterolateral corner with thickened setae. Sternum VIII quadrangular; sternite IX pentagonal in ventral view, with rounded posterolateral lobes covered with abundant, long setae; posterior margin with medial, short, blunt lobe which is dorsally canaliculated; in lateral view triangular, apex not reaching posterior margin of ectoproct. Gonocoxites IX long, thin, sigmoid; base flattened, dorsally curved, connected to gonocoxites XI with a membrane; apex laterally gently curved, with 4–6 processes of different sizes, arranged as a hand. Ectoproct ovoid, covered with abundant, thickened setae on posterior surface, posteroventrally with blunt lobe; anteroventrally with flattened, rounded lobe which is continuous with ventromedial sclerotized, curved sulcus. Gonocoxites X forming a short, thickened, ventrally canaliculated sclerite, with anterior apex slightly expanded, posterior apex with dorsal processes connected to gonostyli X and ventrolateral processes connected to gonapophyses X with a membrane; gonostyli X with thickened and straight base with two lateral processes, the rest of the structure, whip-shaped, ventrally curved, and anteriorly coiled, forming several coils and a loop before protruding from abdomen. Gonapophyses X rod-shaped, straight, narrow, arranged in a V-shaped structure; gonapophyses joined by a membrane covering the gonostyli X base; this membrane is medially sclerotized. Gonocoxites XI thin, U-shaped, medial lobe complex and elaborated, with two differentiated parts: dorsal part as bluntly quadrangular lobe; ventral part with a convex area continuous with ventral, curved, bilobed process; between these parts a semicircular, less sclerotized, hyaline area is present; lateral arms of gonocoxites XI gently curved, with anterior apex straightly angled. Hypandrium internum triangular.

##### Distribution.

Mexico (Chihuahua).

##### Remarks.

This species is known only from its type locality in the Mexican state of Chihuahua, being probably endemic to the country. *Plegamegaptera* was recovered as sister to *P.sonorae*, to which is remarkably resemblant. The enlarged wings and the number and arrangement of processes of the male gonocoxites IX rapidly separate both species. Other genital structures are markedly similar between both species, although the female of *P.megaptera* remains unknown.

#### ﻿﻿ Plega mixteca

Taxon classificationAnimaliaNeuropteraRhachiberothidae

Ardila et al., 2019

Figs 54 , 55

Plega mixteca Ardila et al. 2019: 353. Holotype: male, Mexico, Jalisco (CNIN), specimen examined.

##### Material examined.

***Holotype***. Mexico • ♂; Jalisco, Chamela; 05 Jul. 1985; R. Ayala leg.; CNIN. ***Paratypes*.** Mexico • 1 ♂; **Oaxaca**, Parque Nacional Huatulco, Estación “EL Sabanal”; 15°48'10.7"N, 96°11'39.4"W; 109 m; 02 Jun. 2005; S. Zaragoza, F. Noguera, E. Ramírez, E. González leg.; bosque tropical caducifolio; MDM0134; CNIN. • 1 ♂; Parque Nacional Huatulco, Est. Sabanal; 109 m; 02 Jun. 2005; light trap; MDMO128; CNIN.

##### Other material.

Guatemala • 1 ♂; **San Marcos**, 17.3 Km SE Talisman, Rio Cabuz at Hwy CA2; 14°51'N, 92°04'W; 200 m; 23 May. 1973; Erwin & Hevel leg.; Central American Expedition, 1973; USNM.

Mexico – **Jalisco** • 1 ♂; Instituto Biol. Chamela; 25 Sep. 1986; R. Miller & L. Stange leg.; FSCA. • 1 ♂; Chamela station; 10–11 Jul. 1989; R.J. McGinley leg.; USNM. • 1 ♂; Estac. Biol. Chamela, Camino Chachalaca; 27–31 Jul. 1996; Gonzales & Woolley leg.; Selva Baja, Malaise trap 96/036; TAMU-ENTOX0085449; TAMUIC. • 1 ♀; Mpio. La Huerta, Chamela Biol. Sta.; 28 Jul. 1996; Wm. Godwin leg.; BL/UV light trap; TAMU-ENTOX0070927; TAMUIC. • 1 ♀; Estación de Biología Chamela; 06 Jul. 1988; E. Ramirez leg.; CNIN. • 1 ♀; Estación de Biología Chamela; 05 Jul. 1993; F.A. Noguera leg.; CNIN. • 1 ♀; Estación de Biología Chamela; 12–15 Aug. 2021; I. Garzón, F. Joele, J. Jasso, A. Zaldívar leg.; CNIN. • 1 ♂; Chamela Research Station; 19°29.873'N, 105°02.674'W; 375’; 08 Jul. 2003; F.G. Andrews leg.; at lights; CSCA. – **Oaxaca** • 1 ♀; Parque Nacional Huatulco, Estación “EL Sabanal”; 15°48'10.7"N, 96°11'39.4"W; 109 m; 02 Jun. 2005; S. Zaragoza, F. Noguera, E. Ramírez, E. González leg.; bosque tropical caducifolio; MDMO133; CNIN. • 1 ♀; same data as for preceding; MDM0131, 2721; CNIN. • 1 ♀; same data as for preceding; light trap 1; MDM0132, 2707; CNIN. • 1 ♀; same data as for preceding; light trap 1; MDM0130, 2724; CNIN. • 1 ♀; same data as for preceding; light trap 1; MDM0129, 2698; CNIN. • 1 ♂; Bahía de la Media Luna, Playa Boquilla; 15°40'42.996"N, 96°27'15.984"W; 2 m; 15 Aug. 2018; F. Acevedo, A. Ramírez, D. Curoe leg.; CNIN. 1 ♂; Mpio. Sta. María Huatulco, predio FONATUR 2B; 15°43'48.68"N, 96°9'43.91"W; 05 Aug. 2021; A. Zaldívar-Riverón, P. Benites leg.; trampa HUAT02; CNIN.

##### Diagnosis.

This species has the basal antennal flagellomeres 3× as long as wide. The pronotum is pale with brown medial band and anterolateral areas. The pterostigma of both wings is dark brown with a small yellow area. The forefemur is remarkably robust and expanded, with the posterior surface pale with four brown markings near the medial region which may be interconnected; the anterior surface is dark brown, except on proximal 1/5 pale. On the male genitalia, the gonocoxite IX is thickened, gently sigmoid, and long, with posterior apex paintbrush-shaped, set with 8–18, short digitiform processes. On the female genitalia, the gonapophyses VIII medial part is keel-shaped, posteromedially forming a short, bifid process, whose projections are short and thin. The spermatheca has the proximal and medial sections, thin, while the distal section is abruptly expanded and ovoid at distal portion, with a blunt diverticulum.

##### Description.

***Measurements*.** Male (*n* = 4). Forewing length: 10.1–15.8 mm; Hind wing length: 7.2–12.0 mm.

**Figure 54.**
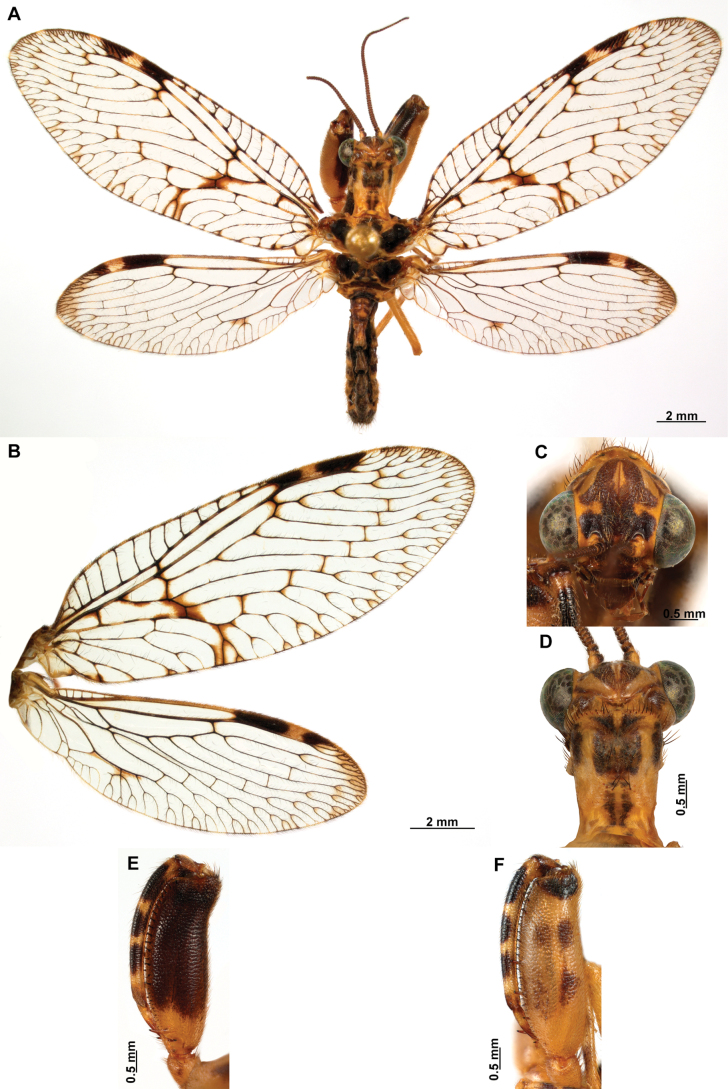
*Plegamixteca* Ardila et al., 2019 **A** male habitus, dorsal **B** wings **C** head, frontal **D** pronotum, dorsal **E** forefemur, anterior surface **F** same, posterior surface.

***Coloration*** (Fig. 54). ***Head*.** Vertexal pale, with lateral dark markings extending from occiput to supra-antennal area, enclosing anterior, lateral yellowish areas, with brown setae; area adjacent to occipital ridge brown; paraocular and supra-antennal arear dark brown, the latter with brown setae; occiput and postgena pale brown with darker areas. Antennal scape pale with brown band on inner surface, with interspersed pale and dark brown setae; pedicel dark brown with pale area on outer surface; flagellum uniformly brown. Frons mostly brown, with lateral triangular darker marks and medial, triangular, pale area. Clypeus with anterior ½ yellowish, and posterior ½ dark brown; labrum brown; mandible amber with pale base; maxilla pale with brown distal ½ of stipes, palpus, galea, and lacinia; labium brown, with proximal area of prementum paler, palpimacula brown. ***Thorax*.** Pronotum pale with brown medial band and anterolateral areas; episternum brown with pale medial area; postfurcasternum pale to brown. Mesonotum dark brown, with pale on area adjacent to sutures; metanotum dark brown with pale medial areas; pre-episternum dark brown; pteropleura dark brown with pale on area adjacent to sutures. ***Foreleg*.** Coxa either completely brown or mostly brown with yellowish anterodistal area; trochanter yellowish to brown. Femur posterior surface pale with four brown markings near medial region which may be interconnected, apex brown; anterior surface dark brown, except on proximal 1/5 pale. Tibia dashed, with yellowish and brown. Basitarsus with base and lanceolate process brown, sub-basal area yellowish; remaining tarsomeres pale brown. ***Mid- and hind leg*.** Coxae brown, sometimes with yellowish areas; trochanter of both legs either completely pale or with brown marking. Femora either entirely pale or with medial brown ring; tibiae pale with diffuse brown rings. Tarsi pale with pale brown setae; distal margins of plantar surface of first four tarsomeres with dark brown setae. ***Wings*.** Forewing mostly hyaline; membrane surrounding crossveins, M stem and fork, medial region and first branch of CuA, apex of CuP, and forks of longitudinal veins amber; pterostigma dark brown with, small, yellow, medial area; major veins, subcostal veinlets, and wing margin alternating yellow and dark brown; crossveins dark brown. Hind wing hyaline, area surrounding first branch of CuA and wing apex amber; pterostigma dark brown with small, yellow, preapical area; longitudinal veins mostly dark brown, with small yellow areas; crossveins brown; wing margin alternating yellow and dark brown. ***Abdomen*.** Tergites brown with lateral pale areas. Pleural membrane mostly dark brown. Sternites mostly brown, with small, lateral pale areas.

***Morphology*** (Fig. 54). ***Head*.** Diamond-shaped in frontal view, rugose; vertexal region raised above compound eyes, with lateral rows of reclined setae; area surrounding coronal suture glabrous, with muscle insertion mark; coronal suture distinct; medial area of ​​supra-antennal region moderately raised, with fine, reclined setae; paraocular area concave. Antenna submoniliform, elongated; scape 1.5× as long as wide, slightly distally expanded, mostly with fine, short setae; pedicel as long as wide; flagellum slightly dorsoventrally flattened, with 54–67 flagellomeres, those of proximal 1/3 discoidal, then progressively changing from slightly wider than long to as long as wide on the rest of flagellum; all articles with medial ring of fine, short setae. Compound eye hemispherical, as wide as ½ of the interocular distance at torulus level. Frons and clypeus narrow. Labrum pentagonal; maxillary palpus with first palpomere as long as wide, second 1.5× as long as wide, third palpomere 4× as long as wide, fourth palpomere 3.5 times as long as wide, fifth as long as third; mentum with thin setae; labial palpus with first palpomere two times as long as wide, second palpomere 4.5 times as long as wide, third palpomere as long as second, palpimacula narrowly ovoid. ***Thorax*.** Pronotum as long as wide, with raised anterior margin, medial and posterior regions; outgrowths covered with thick setae arising flush the pronotal surface. Mesonotum 1.5× as wide as long, with abundant, thick setae on medial area. Metanotum ~ 2× as long as wide, glabrous. Pteropleura with abundant, thin setae. ***Foreleg*.** Coxa as long as femur, cylindrical, anterior and posterior surfaces with abundant, thin setae; trochanter trapezoidal, with thin and short setae, except on dorsal surface with some thickened, pedicellate setae near distal margin; anterior surface with protuberant area. Femur remarkably robust and expanded, covered with abundant, fine, short setae; closing surface with posteroventral row of processes composed two medially located, primary processes; proximal portion of the row with sub-basal, secondary process and basal, tertiary process; the rest of the row with abundant tubercle-shaped processes, and stinger-shaped setae; distal portion only slightly raised, composed of tubercle-shaped specializations and stinger-shaped setae; adjacent row of thickened setae with globular base present on distal ¼. Anteroventral row of processes reduced to proximal ½; it is composed of tubercle-shaped specializations and stinger-shaped setae; basal-most primary, curved process present; distal portion composed of a few tubercle-shaped processes; adjacent row of thickened setae with globular base present on distal 2/3. Tibia almost as long as femur, curved, with short, thin setae; ventral surface keeled with prostrate setae; patch of clavate setae apically on anterior surface present. Basitarsus with lanceolate process reaching the middle of fourth tarsomere; clavate setae present proximally on anterior surface; ventrally with single row of prostrate setae; second tarsomere 7× as long as wide; third tarsomere as long as wide, fourth tarsomere two times as long as wide. ***Mid- and hind leg*** with abundant, short, thin setae; tibial spurs short; hind leg longer than midleg, tibia, 1.5× as long as femur; distal margin of plantar surface of first four tarsomeres with lateral groups of four or five thickened setae; on both legs, basitarsus 3.5 times as long as wide, second tarsomere 1.2× as long as wide; third and fourth tarsomeres as long as wide; fifth tarsomere two times as long as wide. ***Wings*.** Forewing narrowly oval, trichosors present along margin except on wing base; venation setose; costal space proximally expanded, humeral vein branched, ~ 13 subcostal veinlets present; pterostigma elongated, narrow, straight, with distinct veinlets; subcostal space with single crossvein, medially located; Sc vein abruptly posteriorly bent at proximal pterostigma margin to merge the RA; radial space with two crossveins; *rarp2* gently curved with two or three RP branches; three veins arising from *rarp1*; M vein basally fused with RA; RP base, close to divergence of M and R; M forked nearly opposite to RP origin, 1 r-m connecting RP base and M, forming a trapezoidal cell; four or five gradate crossveins present. Cubitus deeply forked; CuP basally angled and approaching A1, distally forked opposite to separation of M and R; A1 apically forked, ending on posterior margin slightly beyond CuP fork, A2 forked opposite to CuP angle. Hind wing smaller and narrower than forewing, narrowly oval; costal space narrow and reduced, with 4–6 veinlets; C and Sc fused at ¼ of wing length, Sc vein abruptly curved posteriorly at proximal margin of pterostigma to merge RA; pterostigma elongated, narrow, straight, composed of well-defined veinlets; radial space with single crossvein, oblique; three or four veins arising from *rarp1*, one from *rarp2*. 1r-m sigmoid, connecting the stems of M and RP. Media forked beyond R fork. Cubitus deeply forked, intracubital crossvein subparallel to longitudinal wing axis; CuA sinuous, first branch candelabrum-shaped, spur vein generally absent; CuP not touching A1, strongly anteriorly bent at distal 1/3, pectinate; two crossveins on cubitoanal space; A1 simple ending on wing margin slightly beyond 1m-cu level; A2 simple, short, and curved. ***Abdomen*.** Cylindrical to medially expanded; tergites subquadrate; sternites rectangular.

**Figure 55.**
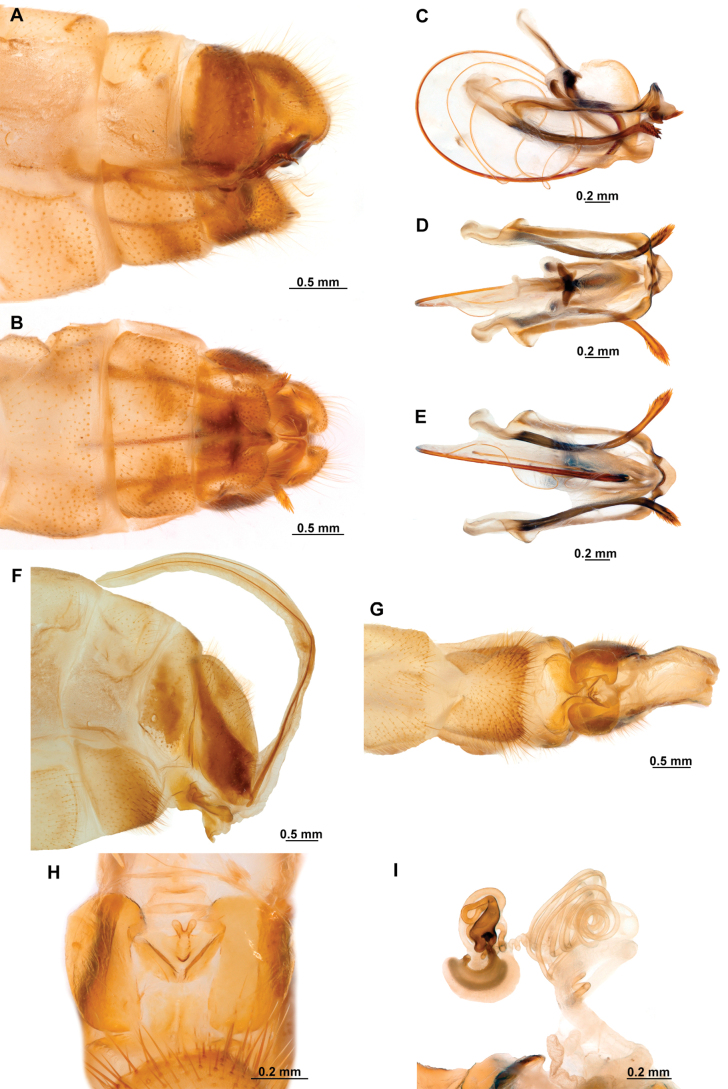
*Plegamixteca* Ardila et al., 2019 **A** male terminalia, lateral **B** same, ventral **C** male genitalia, lateral **D** same, dorsal **E** same, ventral **F** female terminalia, lateral **G** same, ventral **H** gonapophyses VIII of female, ventral **I** spermatheca.

***Male genitalia*** (Fig. 55A–E). Tergite IX medially narrower than laterally; lateral margin rounded. Sternum VIII rectangular; sternite IX pentagonal in ventral view, with rounded posterolateral lobes covered with abundant setae; posterior margin with short, medial, obtuse lobe, which is dorsally canaliculated; in lateral view trapezoidal, apex reaching posterior margin of ectoproct. Gonocoxites IX thickened, gently sigmoid, long; base spatulate, elongated; apex paint-brush-shaped, with 8–18, short digitiform processes. Ectoproct bluntly trapezoidal; ventral area sclerotized, convex, with anterior margin rounded, ventromedially is continuous with sclerotized, narrow, incurved projection. Gonocoxites X forming a short, thickened, straight, ventrally canaliculate sclerite; posterior apex with dorsal processes connected to gonostyli X and short ventrolateral processes connected to gonapophyses X with a membrane; gonostyli X with thickened, curved base with two lateral processes, the rest of the structure, ventrally curved, and anteriorly coiled, forming two loops before protruding from abdomen. Gonapophyses X rod-shaped, narrow, with dorsally curved apexes; gonapophyses arranged in a V-shaped structure, joined by a membrane covering the gonostyli X base; this membrane is medially slightly sclerotized. Gonocoxites XI U-shaped, medial lobe complex and elaborated with two differentiated parts: dorsal part as a rounded lobe; ventral part as a convex area covered with minute granules, which is ventrally continuous with a blunt, short, curved process; between these parts a rectangular, less sclerotized, ellipsoid, hyaline area is present; lateral arms of gonocoxites XI long, thin, straight, with anterior apex expanded and ventrally curved. Hypandrium internum triangular, concave.

***Female genitalia*** (Fig. 55F–I). Sternum VII trapezoidal, transversely narrowed, posteromedially broadly concave. Tergite VIII narrower medially than laterally, encircling the spiracle of the segment; lateral margin D-shaped. Gonocoxites VIII as a transversely narrowed sclerite with lateral concavities. Gonapophyses VIII with medial part keel-shaped, posteromedially with a short, bifid process, whose projections are short and thin; laterally folded beneath tergite IX + ectoproct, a broadly oval plate constitutes the lateral part of the gonapophyses VIII. Tergite IX + ectoproct narrowly ovoid. Gonocoxites IX elongated, sinuous, and narrow, as long as the last five abdominal segments together. Bursa copulatrix long, funnel-shaped, membranous, and striated. Spermatheca complex and entangled; proximal section, thin, long, forming several coils; medial section as wide as proximal section, forming a long spiral; distal section abruptly expanded and ovoid at distal portion, with a blunt diverticulum; fertilization canal duct proximally triangular, concave; fertilization canal curved, covered with microfilaments.

##### Distribution.

Guatemala (San Marcos), Mexico (Jalisco, Oaxaca).

##### Remarks.

This species was previously known solely from Mexico (Jalisco and Oaxaca) (Ardila-Camacho et al. 2019), and here its distribution range is extended to Guatemala (San Marcos). The striking color pattern with brown and yellow areas of this species is remarkable. This is particularly noticeable on the wings since the pterostigma exhibits a bright yellow area, and the membrane has strong amber maculation. On the forefemur, the anterior surface has a distinctive coloration with yellow base and brown on the rest of the surface. This species was recovered as sister to the remaining species of *Plega* comprising the clade of Mexican and Nearctic species. Morphologically, this species has discoidal, basal antennal flagellomeres and robust forefemur resembling *P.fumosa*. The genital sclerites of both sexes are similar to those of *P.fumosa* and *P.dactylota*, yet the shape of the sternite and the male gonocoxites IX easily distinguish *P.mixteca*.

#### ﻿﻿ Plega obtusa

Taxon classificationAnimaliaNeuropteraRhachiberothidae

Ardila-Camacho & Contreras-Ramos sp. nov.

https://zoobank.org/03B9E831-B948-4449-A923-5E1DA89BF46E

Figs 56 , 57

##### Type locality.

Mexico, **Sonora**: Mpio. Álamos, Parque La Colorada, Sendero Tecolote, 27°00.675‘N, 108°56.987'W, 469 m, 16 Sep. 2019, selva baja caducifolia, luz negra y blanca, Contreras, Barba, Cancino, Ardila, Marquez leg.

##### Material examined.

***Holotype*** male, pinned. Original label: “Mexico, **Sonora**, Mpio. Álamos, Parque La Colorada, Sendero Tecolote, 27°00.675'N, 108°56.987'W, 469 m, 16 Sep. 2019, Contreras, Barba, Cancino, Ardila, Marquez leg., selva baja caducifolia, luz negra y blanca” CNIN. ***Paratypes*.** Mexico • 3 ♂ 15 ♀; same data as for holotype; CNIN. • 5 ♂ 1 ♀; same data as for holotype; sendero arroyo alto (Chalatón); 27°00.551'N, 108°57.206'W; 510 m; 17 Sep. 2019; Contreras, Barba, Cancino, Ardila, Marquez leg.; selva baja caducifolia, luz negra y blanca; CNIN.

##### Other material.

USA • 1 ♀; **Arizona**, 1 mile N. Nogales, Mariposa Canyon; 19 Aug. 1971; R.L. Jacques leg.; FSCA. • 1 ♂; Atascosa Mountains; 16 Aug. 1987; FSCA. • 1 ♂; Santa Cruz Co., Patagonia Mtns., Mt. Washington; 4300’; 13 Aug. 1991; L.G. Bezark, R.A. Cunningham, D.E. Russell leg.; Hg vapor and U.V. light; CAS.

Mexico • 1 ♀; **Sonora**, Álamos, Acosta Trailer Pk.; 19 Sep. 1980; D. DeeWilder leg.; CAS. • 1 ♀; Sonora, Private La Púrica Mtn. Site Camp, Site #4; 30°31'51.1"N, 109°43'21.8"W; 1644 m; 09 Sep. 2013; Stevens & Ledbetter leg.; NEU130051; USNM. • 1 ♂; same data as for preceding; NEU130052; USNM. • 1 ♂; same data as for preceding; NEU130058; Hg vapor light; USNM. • 1 ♂; same data as for preceding; NEW130057; USNM. • 1 ♂; Sonora, Agua Prieta, Rancho Los Ojos Calientes, 48.6 Km ESEA Agua Prieta; 31.2783°N, 109.001°W; 1291 m; 23 Apr. 2017: Van Devender leg.; Paiting Riparian vegetation, Oak woodland on slopes; USNMENT01541893; USNM. • 1 ♂; same data as for preceding; USNMENT01541894; USNM. • 1 ♂ 1 ♀; Mpio. Álamos, Parque la Colorada, sendero Tecolote; 27°00.675'N, 108°56.987'W; 469 m; 16 Sep. 2019; Contreras, Barba, Cancino, Ardila, Marquez leg.; selva baja caducifolia, black and white light trap; CNIN. • 1 ♂; same data as for preceding; 2738; CNIN. • 1 ♂ 5 ♀; Mpio. Álamos, Parque La Colorada, Sendero Puente; 27°00.803'N, 108°57.133'W; 462 m; 20 Sep. 2019; A. Ardila, Y. Marquez, R. Cancino leg.; black and white light trap, selva baja caducifolia; CNIN. • 2 ♂; Mpio. Álamos, Parque La Colorada, Río Chalatón; 27°00.552'N, 108°57.241'W; 506 m; 17 Nov. 2019; R. Cancino, Y. Marquez, A. Ardila leg.; direct collection, selva baja caducifolia; CNIN. • 8 ♂ 4 ♀; Mpio. Álamos, Parque la Colorada, sendero arroyo alto (Chalatón); 27°00.551'N, 108°57.206'W; 510 m; 17 Sep. 2019; Contreras, Barba, Cancino, Ardila, Marquez leg.; black and white light trap, selva baja caducifolia; CNIN. • 1 ♀; Nacozari de García, 15.9 Km SE. Nacozari de García, Rancho La Zulema, Sierra Juriquipa; 30°17'2.04"N, 109°33'37.08"W; 1687 m; 14 Mar. 2017; Van Devender, J. Palting leg.; rocky mountain side, oak Woodland; CNIN.

##### Diagnosis.

This species is closely related to *P.spinosa*, as both has the area surrounding frontal sutures sunken, and the supra-antennal region with lateral outgrowths covered with thickened, reclined setae. However, the antennal flagellum in this species is completely brown, with basal flagellomeres as wide as long. The femur posterior surface is pale with numerous dark brown dots; the anterior surface is dark brown. The forewing may be nearly completely hyaline or have a blackish brown stripe extended from base of mediocubital space to first branch of CuA and apex of CuP forming an obtuse angle at M fork level; the pterostigmata are dark brown with wide, cream medial area. On the male genitalia, the gonocoxite IX is thin, gently curved, and elongated, with posterior apex straight and sharply pointed. The ventral part of the gonocoxites XI medial lobe appears as a pentagonal lobe equipped with a posteroventral, hook-shaped process. On the female genitalia, the gonapophyses VIII medial part is boat-shaped, and posteromedially set with two short, blunt lobes. The spermatheca has the proximal section spiral-shaped; the medial section is entangled; and the distal section is S-shaped with blunt diverticulum.

##### Etymology.

The specific epithet of this species *obtusa* refers to the pigmentation pattern on the forewing of this species, in which a dark brown stripe forms an obtuse angle. An adjective in the nominative case.

##### Description.

***Measurements*.** Male (*n* = 5). Forewing length: 12.6–17.6 mm; Hind wing length: 9.8–14.5 mm. Female (*n* = 8): Forewing length: 13.0–19.4 mm; Hind wing length: 10.6–15.0 mm.

**Figure 56.**
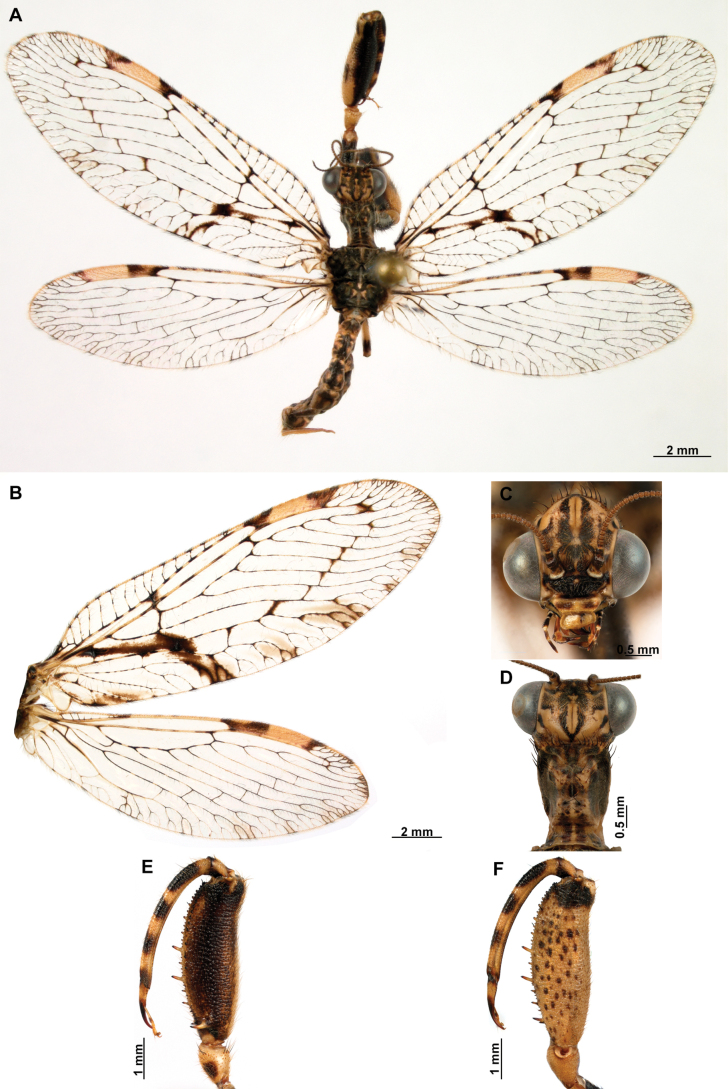
*Plegaobtusa* Ardila-Camacho & Contreras-Ramos, sp. nov. **A** female habitus, dorsal **B** wings **C** head, frontal **D** pronotum, dorsal **E** forefemur, anterior surface **F** same, posterior surface.

***Coloration*** (Fig. 56). ***Head*.** Vertexal region pale, with lateral dark brown markings extending from occiput to toruli forming a V-shaped pattern, mainly with pale brown setae; area adjacent to occipital ridge dark brown; supra-antennal area dark brown with lateral pale areas, with pale brown setae and some dark brown setae; postgena pale with dark brown markings. Antennal scape brown, with pale dorsolateral longitudinal band, entire surface with pale brown setae, and some dark brown setae dorsally; pedicel brown, flagellum pale brown with brown setae. Frons dark brown with pale medial inverted V-shaped pale mark. Clypeus pale with brown lateral markings; labrum pale; mandible pale with dark brown corners, dark amber at apex; maxilla pale with dark brown markings, palpus with first three segments dark brown, remaining palpomeres paler; labium pale with brown suffusions, setae pale brown; labial palpus dark brown, pale at junctions, palpimacula pale brown. ***Thorax*.** Pronotum pale with extensive irregular brown markings, lateral areas darker; episternum dark brown, postfurcasternum pale brown darker posterior areas. Mesonotum dark brown with median longitudinal pale stripe and lateral pale marks adjacent to sutures, setation dark brown on medial region, pale on the rest; metanotum dark brown with cream anteromedian area; median region adjacent to scutoscutellar suture pale; pre-episternum dark brown; pteropleura pale with brown markings, setation pale brown. ***Foreleg*.** Coxa mostly dark brown, with longitudinal pale stripe on dorsal surface, apex pale brown, setation dark brown; trochanter pale with brown spots on anterior surface, setation pale brown, except dorsally dark brown. Femur posterior surface pale with numerous dark brown spots and dark brown apex; anterior surface dark brown. Tibia dashed with pale and dark brown. Basitarsus pale on proximal region, changing to brown towards the apex, clavate setae pale brown; remaining tarsomeres pale with pale brown suffusions. ***Mid- and hind leg*.** Coxae pale with brown areas, pale and pale or dark brown setae present; trochanter pale with brown area on inner surface. Femora and tibiae pale with brown spots, apex of femur and base of tibia darker, interspersed pale, pale, and dark brown setae present; tibial spurs brown. Basitarsus on both legs pale, remainder tarsomeres pale brown, pale brown setae present; distal margins on ventral surface with dark brown setae; pretarsal claws amber. ***Wings*.** Forewing either nearly completely hyaline or conspicuously patterned; if patterned, membrane surrounding crossveins and marginal twigging blackish brown; posterior and apical margins on distal ½ of wing with intermittent, smoky areas between apical branches of longitudinal veins; area surrounding A2 fork blackish brown; a blackish brown stripe extended from base of mediocubital space to first branch of CuA and apex of CuP forming an obtuse angle at M fork is present; pterostigma dark brown at base and apex, and a wide cream medial area; major veins and subcostal veinlets alternating pale and dark brown, most of the crossveins dark brown; wing margin alternating pale and dark brown. Hind wing either completely hyaline or with smoky areas on membrane adjacent to CuA base and between apical branches of longitudinal veins on distal ½ of wing; pterostigma with preapical, wide, and sub-basal, small cream areas, apex and part of proximal ½ dark brown; longitudinal veins with extensive pale areas and dark brown regions; crossveins and subcostal veinlets, either pale, bicolor or dark brown; wing margin alternating pale and dark brown. ***Abdomen*.** Tergite I and anterotergite II mostly dark brown; posterotergite II and tergites III–VIII brown with pale anterior, medial, and posterior lateral areas; tergites II–VIII with pale anterolateral scars; setation pale. Pleural membrane mostly dark brown with area adjacent to tergites and sternites pale. Sternites pale with wide lateral areas, lateral margins brown. gonostyli X with thickened and concave base with two lateral processes, the rest of the structure, ribbon-shaped, ventrally curved, and anteriorly recurved forming a single loop before reach posterodorsal canal of sternite IX

***Morphology*** (Fig. 56). ***Head*.** Diamond-shaped in frontal view, rugose; vertexal region raised above compound eyes, with lateral rows of reclined setae; area surrounding coronal suture glabrous, with muscle insertion mark; coronal suture distinct; area surrounding frontal sutures sunken; medial area of ​​supra-antennal region with lateral outgrowths located above toruli, covered with thickened, reclined setae; paraocular area concave. Antenna filiform, short; scape 1.5× as long as wide, slightly distally expanded, with short setae, dorsally longer and thicker; pedicel as long as wide; flagellum with 42–48 flagellomeres, which are as wide as long on proximal ½, slightly longer than wide on distal ½; all articles with medial ring of fine, short setae. Compound eye hemispherical, as wide as ½ of the interocular distance at torulus level. Frons and clypeus narrow, with fine and short setae. Labrum pentagonal with thin, short setae; maxillary palpus with first palpomere as long as wide, second 1.5× as long as wide, third palpomere 3× as long as wide, fourth palpomeres 2.5× as long as wide, fifth palpomere slightly longer than third, all with short and thin setae; mentum with thin, long setae; labial palpus with first palpomere 1.2× as long as wide, second palpomere 4× as long as wide, third palpomere slightly longer than second, palpimacula narrowly ovoid. ***Thorax*.** Pronotum slightly longer than wide, subquadrate, with raised anterior margin, medial and posterior regions; outgrowths covered with pedicellate, thick setae; remaining surface with fine, short setae. Mesonotum 2× as wide as long, with scattered, thick, pedicellate setae on medial area, most of the surface with scattered, thin, short setae. Metanotum ~ 2× as long as wide, mostly glabrous. Pteropleura with short, thin setae. ***Foreleg*.** Coxa as long as femur, cylindrical, anterior and posterior surfaces with pedicellate fine or thick setae of different sizes; trochanter trapezoidal, with thin and short setae, except on dorsal and anterior surfaces with some thickened setae; anterior surface with protuberant area. Femur robust, covered with abundant, fine, short setae which arise from protuberant bases on both surfaces. Closing surface with posteroventral row of processes composed two medially located, primary processes and medial secondary or tertiary process; proximal portion of the row with two secondary processes; the rest of the row with abundant tubercle-shaped processes, stinger-shaped setae, and some tertiary processes; distal portion raised composed of tubercle-shaped specializations and stinger-shaped setae; adjacent row of thickened setae with globular base present on distal ⅕. Anteroventral row of processes reduced to proximal ⅓; it is composed of tubercle-shaped specializations, stinger-shaped setae, and few tertiary processes; the basal-most primary, curved process is present; distal portion composed of a few tubercle-shaped processes; adjacent row of thickened setae with globular base present on distal ⅔. Tibia almost as long as femur, curved, with thin, short setae; ventral surface keeled with prostrate setae; a patch of clavate setae apically on anterior surface is present. Basitarsus with lanceolate process reaching the middle of fourth tarsomere; clavate setae present proximally on anterior surface; ventrally with single row of prostrate setae; second tarsomere nearly 6× as long as wide; third tarsomere as long as wide, fourth tarsomere two times as long as wide. ***Mid- and hind leg*.** Coxae and trochanter with short, thin setae; femora and tibiae of both legs with interspersed fine, short setae and a few thickened setae; tibial spurs short; hind leg longer than midleg, tibia, 1.5× as long as femur; tarsi with fine and short setae, except on distal margin of plantar surface with lateral groups of 5–7 thickened setae; on both legs, basitarsus 3.5 times as long as wide, second tarsomere 1.5× as long as wide; third and fourth tarsomeres as long as wide; fifth tarsomere two times as long as wide. ***Wings*.** Forewing narrowly oval, trichosors present along margin except on proximal ½ of wing; venation setose; costal space proximally expanded, humeral vein branched, 11–15 subcostal veinlets; pterostigma elongated, narrow, curved, with barely perceptible veinlets; subcostal space with single crossvein, medially located; Sc vein abruptly posteriorly bent at proximal pterostigma margin to merge with RA; radial space with two crossveins; *rarp2* curved with three or four RP branches; three veins arising from *rarp1*; M vein basally fused with RA; RP base, widely separated from divergence of M and R; M forked nearly opposite to RP origin, 1 r-m connecting RP base and MA base, forming a trapezoidal cell; four or five gradate crossveins present. Cubitus deeply forked; CuP basally angled and approaching A1, distally forked slightly beyond the level of separation of M and R; A1 apically forked, ending on posterior margin, slightly beyond CuP fork level, A2 forked slightly beyond CuP angle level. Hind wing smaller and narrower than forewing, narrowly oval; costal space narrow and reduced, with 7–9 veinlets; C and Sc fused at ¼ of wing length, Sc vein abruptly curved posteriorly at proximal margin of pterostigma to merge RA; pterostigma elongated, curved, narrow, composed of poorly defined veinlets; radial space with single crossvein, oblique; four veins arising from *rarp1*, one or two from *rarp2*. 1r-m sigmoid, connecting the stems of M and RP. Media forked beyond R fork. Cubitus deeply forked, intracubital crossvein subparallel to longitudinal wing axis; CuA sinuous, first branch candelabrum-shaped, spur vein generally absent; CuP not touching A1, strongly anteriorly bent at distal 1/3, pectinate; two crossveins on cubitoanal space; A1 simple ending on wing margin slightly beyond 1m-cu level; A2 simple, short, and curved. ***Abdomen*.** Cylindrical to medially expanded, setae on tergites, scattered, thin, and short, gradually longer and more abundant towards terminal segments; tergites subquadrate, tergites III-VII with elongated anterolateral scars. Sternites rectangular, with abundant fine and short setae; sternites III–VII with posterolateral elongate scars.

**Figure 57.**
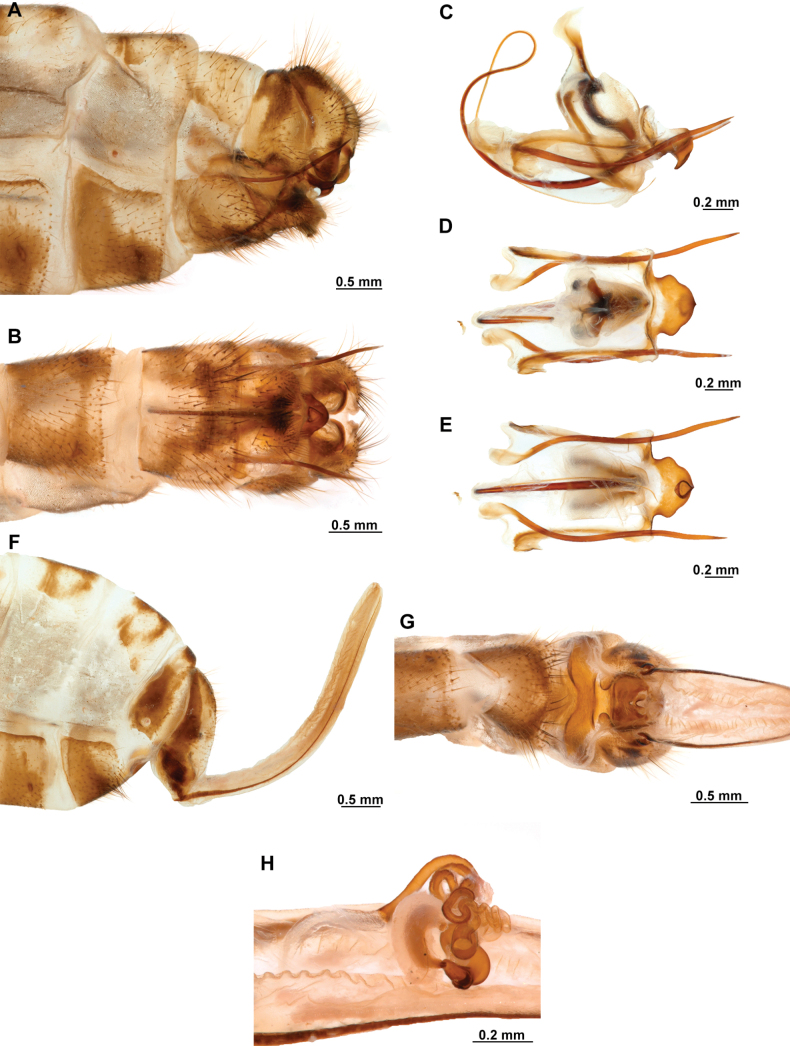
*Plegaobtusa* Ardila-Camacho & Contreras-Ramos, sp. nov. **A** male terminalia, lateral **B** same, ventral **C** male genitalia, lateral **D** same, dorsal **E** same, ventral **F** female terminalia, lateral **G** same, ventral **H** spermatheca.

***Male genitalia*** (Fig. 57A–E). Tergite IX medially narrower than laterally; lateral margin rounded. Sternum VIII quadrangular, setose; sternite IX pentagonal in ventral view, with rounded posterolateral lobes; medial region with prominent, thickened setae, remainder surface with long and thin setae, posteromedially produced into a blunt lobe with canaliculate dorsal surface; in lateral view triangular, apex not reaching posterior margin of ectoproct. Gonocoxites IX thin, gently curved, elongated; base flattened, dorsally curved, connected to gonocoxites XI with a membrane; apex straight, sharply pointed, without processes. Ectoproct ovoid, covered with abundant, thin, long setae; anteroventrally a more sclerotized, triangular, convex area, which is continuous with ventromedial sclerotized, curved sulcus. Gonocoxites X forming a short, thickened, ventrally canaliculate sclerite, with anterior apex expanded, posterior apex with dorsal processes connected to gonostyli X and ventrolateral processes connected to gonapophyses X with a membrane; gonostyli X with thickened and concave base with two lateral processes, the rest of the structure, ribbon-shaped, ventrally curved, and anteriorly recurved forming a single loop before reach posterodorsal canal of sternite IX. Gonapophyses X rod-shaped, straight, narrow, arranged in a V-shaped structure with apexes dorsally bent; gonapophyses are joined by a membrane covering the gonostyli X base; this membrane is medially sclerotized and laterally covered with microspinulae. Gonocoxites XI thin, U-shaped, medial lobe complex and elaborated with two differentiated parts: a dorsal, rounded, concave lobe, and a ventral, pentagonal lobe equipped with posteroventral hook-shaped process; between these parts a less sclerotized, hyaline area is present; lateral arms of gonocoxites XI straight with anterior apex expanded and curved ventrad. Hypandrium internum with lateral fins.

***Female genitalia*** (Fig. 57F–H). Sternum VII rectangular with posterior margin concave, setose, posteromedially continuous with a sclerotized, glabrous area. Tergite VIII slightly narrower medially than laterally, encircling the spiracle of the segment. Gonocoxites VIII as a trapezoidal plate with lateral concavities. Gonapophyses VIII with boat-shaped medial part, posteromedially with two short, blunt lobes; lateral part triangular, concave, folded beneath tergite IX + ectoproct. Tergite IX + ectoproct triangular. Gonocoxites IX elongated, sinuous, and narrow, as long as the last five abdominal segments together. Bursa copulatrix short, siphon-shaped, area adjacent to genital pore, expanded, flattened, and sclerotized; the rest semi-membranous and striated. Spermatheca complex and entangled, proximal section, thin, spiral-shaped forming several coils; medial section narrower than proximal section, entangled; distal section thicker than proximal section, S-shaped, with blunt diverticulum; fertilization canal duct proximally subconical, concave; fertilization canal pod-shaped, covered with microfilaments.

##### Distribution.

Mexico (Sonora), USA (Arizona).

##### Remarks.

This is a rather distinctive and striking species distributed in Northern Mexico (Sonora) and Arizona. It was recovered sister to *P.oswaldi* + *P.spinosa*, to which it is markedly similar. As in these species, *P.obtusa* has a sunken area and setose protuberances on the supra-antennal region, and the male gonocoxites IX lack digitiform processes. The cream colored medial (or preapical) area on the pterostigmata and a dark amber stripe forming an obtuse angle on the forewing easily distinguish this species. Moreover, the gonostyli X forming a single loop and the hook-shaped process on the medial lobe of the gonocoxites XI are also distinctive attributes of this species.

The egg and primary larva of this species were described and compared in a context of Mantispoidea in a recent publication by Ardila-Camacho et al. (2021b).

#### ﻿﻿ Plega oswaldi

Taxon classificationAnimaliaNeuropteraRhachiberothidae

Ardila-Camacho & Contreras-Ramos sp. nov.

https://zoobank.org/8ED565CD-C968-4CCF-9203-EC9D21796DB8

Figs 58 , 59

##### Type locality.

Mexico, **Guerrero**: San Luis Acatlán, Km 38 San Luis-Tlapa El Mango, 17°01'33"N, 98°44'15"W, 701 m, 21 Mar. 2006, L. Cervantes leg.

##### Material examined.

***Holotype*** male, pinned, with genitalia in a separate microvial. Original label: “Mexico, **Guerrero**, San Luis Acatlán, Km 38 San Luis-Tlapa El Mango, 17°01'33"N, 98°44'15"W, 701 m, 21 Mar. 2006, L. Cervantes leg.” CNIN. ***Paratype***. Mexico • 1 ♂; **Guerrero**, Acahuizotla; 17 Oct. 2009; S. Zaragoza, F. Noguera, E. Ramírez, E. González leg.; bosque tropical caducifolio; MDM0102; CNIN.

##### Diagnosis.

This species is closely related to *P.spinosa*; it has the area adjacent to frontal sutures slightly sunken, whereas the ​​supra-antennal has lateral outgrowths, covered with reclined setae. The antennal flagellum has the basal flagellomeres as long as wide, and three pale, preapical flagellomeres are present. The forefemur posterior surface is pale with numerous, irregular brown markings; the anterior surface is brown with pale base. The wings are completely hyaline. On the male genitalia, the gonocoxite IX is thin, short, and sigmoid with posterior apex pointed, and without digitiform processes. The ectoproct has the posteroventral surface covered with short, conical setal bases. The gonostyli X is short, ventrally curved, and posteriorly recurved at middle, before protruding from abdomen. The ventral part of the gonocoxites XI median lobe has a medial process appearing as a T-shaped ridge covered with microspinulae; ventrally a caudally curved, rounded process is present.

##### Etymology.

This species is named after John D. Oswald, American Neuropterologist who has devoted much of his life to the advance in the knowledge of neuropteroid insects.

##### Description.

***Measurements*.** Male (*n* = 1). Forewing length: 12.8 mm; Hind wing length: 9.8 mm.

**Figure 58.**
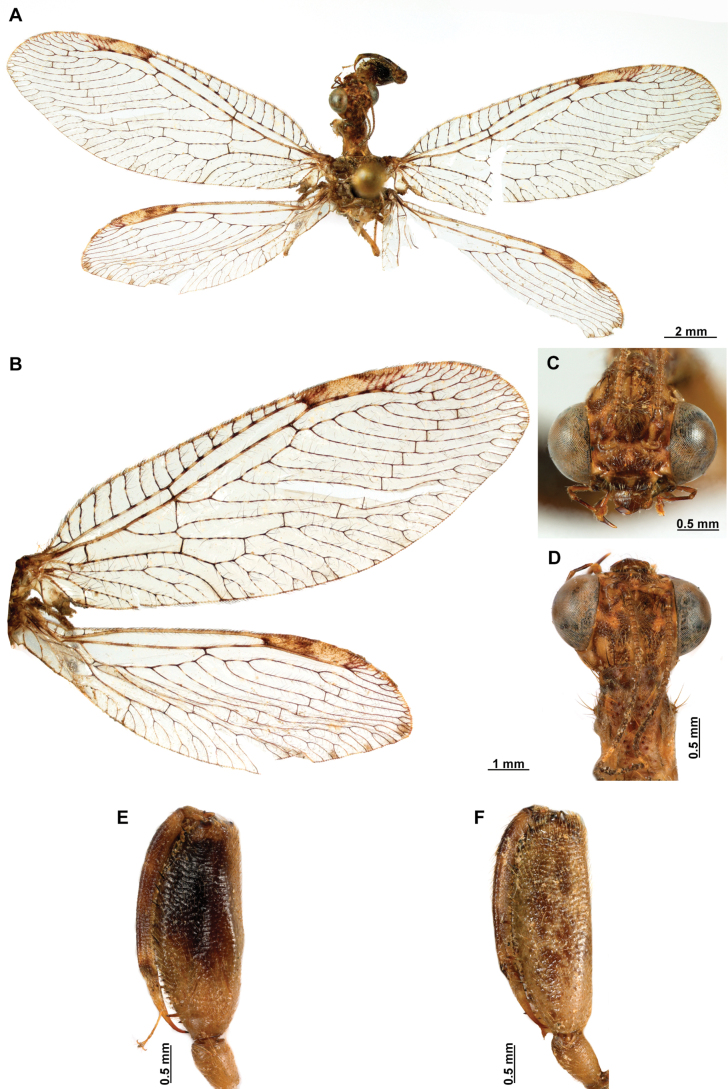
*Plegaoswaldi* Ardila-Camacho & Contreras-Ramos, sp. nov. **A** male habitus, dorsal **B** wings **C** head, frontal **D** pronotum, dorsal **E** forefemur, anterior surface **F** same, posterior surface.

***Coloration*** (Fig. 58). ***Head*.** Vertexal region pale with brown lateral markings extending from occipital ridge to toruli, enclosing small pale areas; pale brown setae on brown marks present; area adjacent to lateral parts of occipital ridge dark brown; occiput and postgena pale brown, with longitudinal darker stripe. Antennal scape pale with longitudinal, brown stripes on dorsal and ventral surfaces; pedicel brown, flagellum brown with three pale, preapical flagellomeres. Frons brown with pale margins. Clypeus pale with brown lateral markings; labrum brown; mandible pale with dark brown corners, amber at apex; maxilla brown; labium with pale postmentum, brown prementum, ligula, and palpus, palpimacula pale brown. ***Thorax*.** Pronotum brown with paler areas on anteromedial region, pale posterolateral areas, darker anterolateral areas; episternum brown, postfurcasternum brown. Mesonotum brown, with paler areas on medial region, and darker lateral regions, pale anterolateral, small areas; metanotum dark brown laterally, paler medially; pre-episternum pale brown; pteropleura brown with pale areas. ***Foreleg*.** Coxa pale, with pale brown apical areas; trochanter pale with apex brown. Femur posterior surface pale with numerous, irregular brown markings; anterior surface brown, except pale base. Tibia brown, alternating darker and paler areas. Basitarsus pale brown; remaining tarsomeres pale brown. ***Mid- and hind leg*.** Coxae brown with pale areas; trochanter pale with brown apex. Femora pale with brown suffusions at middle and apex, tibiae pale with brown areas. Tarsi pale to pale brown with pale brown setae. ***Wings*.** Forewing hyaline; pterostigma brown with pale medial area; major veins and subcostal veinlets alternating pale and brown, crossveins dark brown. Hind wing hyaline; pterostigma brown with pale preapical area; longitudinal veins alternating pale and brown; crossveins brown. ***Abdomen*.** cleared.

***Morphology* (Fig. 58)**. ***Head*.** Diamond-shaped in frontal view, rugose, vertexal region raised above compound eyes, with lateral rows of reclined setae; coronal suture distinct, with adjacent area glabrous, with muscle insertion mark; area adjacent to frontal sutures slightly sunken; medial area of ​​supra-antennal region with lateral outgrowths, covered with reclined setae; paraocular area concave. Antenna filiform, short; scape two times as long as wide, slightly distally expanded, dorsal surface with thickened setae, near distal margin; pedicel as long as wide; flagellum with 36 flagellomeres, as wide as long on basal region, slightly longer than wide on the rest, all flagellomeres with medial ring of short setae. Compound eye hemispherical, as wide as ½ of the interocular distance at torulus level. Frons and clypeus narrow, rugose, with fine and short setae. Labrum pentagonal with fine and long setae; maxillary palpus with first palpomere as long as wide, second 1.2× as long as wide, third palpomere 5× as long as wide, fourth palpomeres 2.5× as long as wide, fifth palpomere as long as third; mentum with fine, long setae; labial palpus with first palpomere two times as long as wide, second palpomere 5× as long as wide, third palpomere slightly longer than second, palpimacula narrowly ovoid. ***Thorax*.** Pronotum 1.5× as long as wide, with raised anterior margin, medial and posterior regions; raised regions covered with pedicellate, thickened setae. Mesonotum 1.5× as wide as long, with scattered, thickened, pedicellate setae on medial area. Metanotum ~ 2× as long as wide, mostly glabrous. Pteropleura with short, thin setae. ***Foreleg*.** Coxa as long as femur, cylindrical, anterior and posterior surfaces with pedicellate, fine setae; trochanter subtrapezoidal, dorsally with some pedicellate, thick setae; anterior surface with protuberant area covered with pedicellate, thick setae. Femur robust, covered with abundant, fine, short setae, those of posterior surface arising from protuberant bases; closing surface with posteroventral row of integumentary specializations composed two medially located primary processes; proximal region with basal and sub-basal tertiary processes; the rest of the with abundant tubercle-shaped processes and stinger-shaped setae; distal ½ of the row slightly carinated, composed of tubercle-shaped specializations of different sizes and stinger-shaped setae; adjacent row of thickened setae with globular base present on distal ¼. Anteroventral row of processes reduced to proximal ½, it is composed of tubercle-shaped specializations and stinger-shaped setae; basal, spine-shaped, primary process present, curved; apical portion of anteroventral row composed tubercle-shaped specializations; adjacent row of thickened setae with globular base present on distal ⅔. Tibia almost as long as femur, curved, moderately setose, ventrally keeled with row of prostrate setae; a patch of clavate setae at apex of anterior surface is present. Basitarsus with lanceolate process reaching the middle of fourth tarsomere, equipped with apical, conical Stitz organ; proximal ½ with patch of clavate setae on anterior surface, and single row of prostrate setae on ventral surface; second tarsomere nearly 7× as long as wide; third tarsomere as long as wide, fourth tarsomere two times as long as wide. ***Mid- and hind leg*.** Coxae and trochanter with fine setae, shorter on trochanter; femora and tibiae on both legs covered with fine setae, shorter on tibia; hind leg longer than mid-leg, tibia, 1.5× as long as femur; tarsi with fine and short setae, except on distal margin of plantar surface of the first four tarsomeres with lateral groups of thickened setae; on both legs basitarsus 4× as long as wide, second tarsomere 1.2× as long as wide; third and fourth tarsomeres as long as wide; fifth tarsomere two times as long as wide. ***Wings*.** Forewing narrowly oval, trichosors present along margin except on wing base; venation setose; costal space proximally expanded, humeral vein forked, 17 subcostal veinlets; pterostigma elongated, narrow, curved, with barely perceptible veinlets; subcostal space with single crossvein, medially located; Sc vein abruptly bent posteriad at proximal margin of pterostigma to merge the RA; radial space with two crossveins; *rarp2* curved with five veins arising from it, four veins arising from *rarp1*; M fused basally to RA; RP base widely separated from divergence of M and R; M forked slightly before RP base, 1 r-m connecting RP base and M fork, forming a trapezoidal cell; eight gradate crossveins present. Cu deeply forked; CuP basally angled and approaching A1, forked at level of separation of M and R; A1 apically forked, ending on posterior margin at CuP fork level, A2 forked at CuP angle level. Hind wing smaller and narrower than forewing, narrowly oval; costal space narrow and reduced, with five veinlets; C and Sc fused at ¼ of wing length, Sc vein abruptly curved posteriad at proximal margin of pterostigma to merge the RA; pterostigma elongated, curved, narrow, composed poorly defined veinlets; radial space with single crossvein, oblique; four veins arising from *rarp1*, three from *rarp2*. 1r-m sigmoid, connecting the stems of M and RP. Media forked beyond R fork. Cubitus deeply forked, intracubital crossvein subparallel to longitudinal wing axis; CuA sinuous, first branch candelabrum-shaped, spur vein absent; CuP not touching A1, anteriorly bent at distal 1/3, pectinate; two crossveins on cubitoanal space; A1 simple ending on wing margin slightly beyond 1m-cu level; A2 simple, short, and curved. ***Abdomen*.** Cylindrical, slightly medially expanded; tergites III-VII with elongated anterolateral scars. Sternites rectangular.

**Figure 59.**
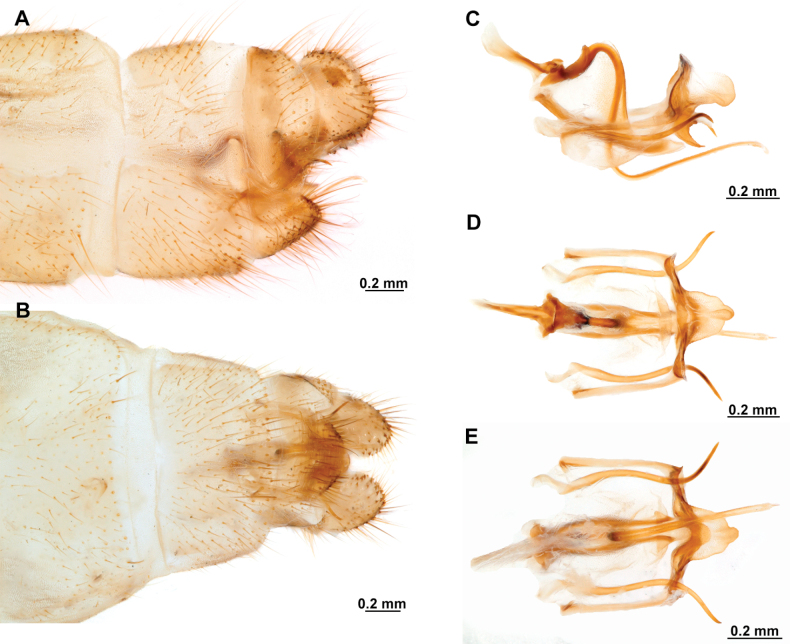
*Plegaoswaldi* Ardila-Camacho & Contreras-Ramos, sp. nov. **A** male terminalia, lateral **B** same, ventral **C** male genitalia, lateral **D** same, dorsal **E** same, ventral.

***Male genitalia*** (Fig. 59). Tergite IX medially narrower than laterally, lateral margin rounded. Sternum VIII rectangular, setose; sternite IX pentagonal in ventral view, setose, posteromedial lobe not developed; in lateral view trapezoidal, apex not reaching posterior margin of ectoproct. Gonocoxites IX thin, short, sigmoid; base curved, connected to gonocoxites XI with a membrane; apex pointed, without digitiform processes. Ectoproct ovoid, covered with abundant, thin setae; posteroventral surface with fine, short, pedicellate setae; anteroventrally with sclerotized, rounded lobe, which is continuous with sclerotized, ventromedial, curved sulcus. Gonocoxites X forming a short, thickened, ventrally canaliculate sclerite, anterior apex slightly expanded, laterally flattened, dorsally curved; posterior apex with dorsal processes connected to gonostyli X, ventrolateral processes connected to gonapophyses X with a membrane; gonostyli X with thickened and short base, with two lateral processes, the rest of the structure, short, ventrally curved and posteriorly recurved at middle, before protruding from abdomen. Gonapophyses X rod-shaped, gently curved, narrow, subparallel; the gonapophyses are joined by a membrane covering the gonostyli X base. Gonocoxites XI, quadrangular, medial lobe complex and elaborated with two differentiated parts: dorsal part as a rounded lobe; ventral part with medial process appearing as a T-shaped ridge covered with microspinulae, ventrally a caudally curved, rounded process is present; between these parts a small, less sclerotized, hyaline area is present; lateral arms of gonocoxites XI straight, short, anterior bent ventrad. Hypandrium internum triangular, concave with medial keel.

##### Distribution.

Mexico (Guerrero).

##### Remarks.

This species is known only from the Mexican state of Guerrero. It was recovered as sister to *P.spinosa* to which is markedly similar. *Plegaoswaldi* can be separated from *P.spinosa* based on its hyaline wings and the lack of prominent spiniform setal bases on the male ectoproct. The morphology of the median lobe of the gonocoxites XI also allows a reliable separation of both species. The female of this species remains unknown.

#### ﻿﻿ Plega paraensis

Taxon classificationAnimaliaNeuropteraRhachiberothidae

Penny, 1982

Fig. 60

Plega paraense Penny, 1982a: 426. Holotype: female, Brazil, Pará (MPEG), photographs examined.
Plega paraensis Penny, 1982a. Ohl (2004): 148.

##### Material examined.

***Holotype*.** Brazil • ♀; **Pará**, Belem, Mocambo; 19 Jan. 1978; Mata de terra firma, Armadilio de malaise; MPEG 30000001; Holotipo *Plegaparaense* Penny; MPEG.

##### Other material.

Brazil • 1 ♂; **Pará**, Canaã dos Carajás, Projeto Serra do Tarzan; 9°18'3.078"S, 59°51'15.84"W; 19 Jan. 2016; Bioespeleo cons. Amb. leg.; DZUP. • 1 ♂ 1 ♀; same data as for preceding; Projeto S11C; 9°17'41.8488"S, 56°51'31.32"W; 21 Apr. 2016; DZUP.

##### Diagnosis.

This species is closely related to *P.hagenella*; the basal antennal flagellomeres are as wide as long on most of the flagellum; the flagellum is completely brown. Additionally, the wings are broadly oval and the forewing pterostigma has a medial pale mark with an anterior notch. The forefemur is remarkably elongated and narrow.

##### Description.

***Measurements*.** Female (*n* = 1): Forewing length: 11.5 mm; Hind wing length: 8.7 mm.

**Figure 60.**
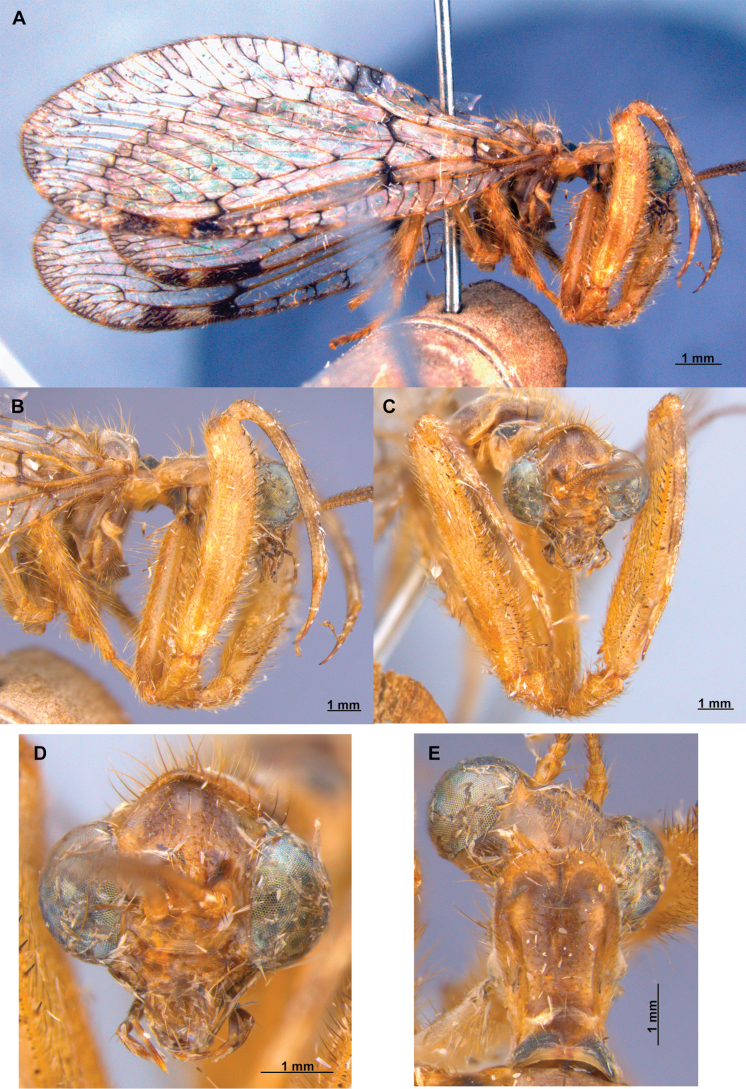
*Plegaparaensis* Penny, 1982 **A** habitus of holotype female **B** detail of thorax and forelegs, lateral **C** detail of head and forelegs, frontolateral **D** head, frontal **E** pronotum, dorsal.

***Coloration*** (Fig. 60). ***Head*.** Vertexal region pale, with irregular, lateral brown marks extending from occiput to supra-antennal area, with pale brown setae; supra-antennal pale with irregular brown marks, and pale brown setae; occiput and postgena pale with brown areas. Antennal scape pale with brown areas, pale to dark brown setae present; pedicel pale, flagellum pale brown. Frons and clypeus pale with brown areas; labrum brown; mandible brown; maxillary and labial palpi brown. ***Thorax*.** Pronotum brown with pale areas on medial region, anterior and lateral margins, pale brown setae present; episternum pale brown, postfurcasternum pale. Mesonotum brown, with area adjacent to sutures pale, setation pale brown; metanotum brown; pre-episternum brown; pteropleura brown with area adjacent to sutures pale, setation mostly pale brown. ***Foreleg*.** Coxa and trochanter pale brown. Femur posterior surface pale with pale brown areas on proximal and distal ⅓; anterior surface mostly pale, with pale brown suffusions on medial area. Tibia dashed with pale and pale brown. Tarsus pale brown. ***Mid- and hind leg*.** Coxae brown; trochanter pale brown. Femora and tibiae pale with brown rings; tarsi pale. ***Wings*.** Forewing mostly hyaline; membrane surrounding crossveins, RP and MP stem, and first branch of CuA and apex of CuP amber; posterior and apical margins on distal ½ of wing with intermittent, amber areas between apical branches of longitudinal veins; pterostigma brown with pale, medial area; major veins, subcostal veinlets, and wing margin alternating pale and brown. Hind wing hyaline, with amber intermittent amber areas between apical branches of longitudinal veins; wing apex amber; pterostigma brown with pale, preapical area; longitudinal veins alternating pale and brown, crossveins brown; wing margin alternating pale and brown. ***Abdomen*.** Cleared.

***Morphology*** (Fig. 60). ***Head*.** Diamond-shaped in frontal view, rugose; vertexal region raised above compound eyes, with lateral rows of reclined, fine setae; area surrounding the distinct coronal suture glabrous, with muscle insertion mark; medial area of ​​supra-antennal not raised, with fine, reclined setae; paraocular area concave. Compound eye hemispherical, as wide as ½ of the interocular distance at torulus level. Antenna filiform; scape slightly longer than wide, slightly distally expanded, with fine, short setae; pedicel as long as wide; flagellum with 58 or 59 flagellomeres, which are as wide as long on most of the flagellum, and slightly longer than wide near the apex; all articles with medial ring of fine, short setae. Frons and clypeus narrow; labrum pentagonal; maxillary palpus with first palpomere as long as wide, second 1.5× as long as wide, third palpomere 3.5 times as long as wide, fourth palpomere 3× as long as wide, fifth slightly longer than third; labial palpus with first palpomere two times as long as wide, second palpomere 4× as long as wide, third palpomere as long as second, palpimacula narrowly ovoid. ***Thorax*.** Pronotum slightly longer than wide, with raised anterior margin, medial and posterior regions; outgrowths covered with thick setae, arising flush with the pronotal surface. Mesonotum slightly wider than long, with scattered, thick, setae on medial area. Metanotum ~ 2× as long as wide. Pteropleura with interspersed short and long, thin setae. ***Foreleg*.** Coxa shorter than femur, cylindrical, anterior and posterior surfaces with fine setae of different lengths; trochanter conical, with thin, short setae, except on dorsal and anterior surfaces with some thickened setae; anterior surface with protuberant area. Femur elongate and narrow, distally slightly curved dorsad, covered with abundant, fine, short setae; closing surface with posteroventral row of processes composed two medially located, primary processes, with a tertiary process between them; proximal portion with basal and sub-basal tertiary processes; the rest of the row with numerous tubercle-shaped processes, and stinger-shaped setae; distal portion raised, composed of tubercle-shaped specializations and stinger-shaped setae; adjacent row of thickened setae with globular base present on distal ¼. Anteroventral row of processes reduced to proximal ⅓ and apex, composed of tubercle-shaped specializations and stinger-shaped setae; the basal-most primary, curved process is present; adjacent row of thickened setae with globular base present on distal ½. Tibia shorter than femur, curved, with thin, short setae; ventral surface keeled, with row of prostrate setae; a patch of clavate setae apically on anterior surface is present. Basitarsus with lanceolate process reaching the middle of fourth tarsomere; clavate setae present proximally on anterior surface; ventrally with single row of prostrate setae. ***Mid- and hind leg*.** Coxae and trochanter with thin setae; femora and tibiae with abundant interspersed fine setae of different lengths. Hind leg longer than midleg, tibia nearly two times as long as femur; tarsi with fine and short setae, except on distal margin of plantar surface with lateral groups of thickened setae. ***Wings*.** Forewing oval, trichosors present along margin except on wing base; venation setose; costal space proximally moderately expanded, humeral vein branched, 14 subcostal veinlets; pterostigma elongated, narrow, gently curved; subcostal space with single crossvein, located at R fork level; Sc vein abruptly posteriorly bent at proximal pterostigma margin to merge with RA; radial space with two crossveins; *rarp2* curved with three RP branches; four veins arising from *rarp1*; M vein basally fused with RA; RP base not widely separated from divergence of M and R; M forked at RP origin level, 1 r-m connecting RP base and M fork, forming a trapezoidal cell; six gradate crossveins present. Cubitus deeply forked; CuP basally angled and approaching A1, distally forked opposite to the level of separation of M and R; A1 simple, ending on posterior margin at level of CuP fork, A2 forked opposite to CuP angle level. Hind wing smaller and narrower than forewing, narrowly oval; costal space narrow and reduced; C and Sc fused at ¼ of wing length, Sc vein abruptly curved posteriorly at proximal margin of pterostigma to merge RA; pterostigma elongated, narrow, curved; radial space with single crossvein, oblique. 1r-m sigmoid, connecting the stems of M and RP. Media forked slightly beyond R fork. Cubitus deeply forked, intracubital crossvein subparallel to longitudinal wing axis; CuA sinuous, first branch candelabrum-shaped, spur vein present; CuP not touching A1, strongly anteriorly bent at distal 1/3, pectinate.

##### Distribution.

Brazil (Pará).

##### Remarks.

Since its original description, this species is known only by the female holotype which was collected in the Brazilian state of Pará. Despite that many morphological features of this species are unknown; it is clear that it is closely related to other South American species. *Plegaparaensis* was recovered sister to *P.duckei*, which is distributed in the neighboring state of Amazonas. Both species share the remarkable narrow forefemur, yet the genital structures of this species are still in urgent need of a detailed description in order to corroborate its phylogenetic affinities.

#### ﻿﻿ Plega pseudohagenella

Taxon classificationAnimaliaNeuropteraRhachiberothidae

Ardila-Camacho & Contreras-Ramos sp. nov.

https://zoobank.org/8D2F6191-34AF-4E94-BE0B-77CDD7E1BE37

Figs 61 , 62

##### Type locality.

Costa Rica, **Guanacaste**: Quebrada el Pedregal, 10°58'58.8"N, 85°32'20.4"W, 5 Feb. 1988, R.M. Strand leg.

##### Material examined.

***Holotype*** male, pinned. Original label: “Costa Rica, **Guanacaste**, Quebrada el Pedregal, 10°58'58.8"N, 85°32'20.4"W, 5 Feb. 1988, R.M. Strand leg., *Plegahagenella* det. Penny, 92” CAS. ***Paratypes*.** Costa Rica • 1 ♀; **Guanacaste**, 5 Km NW Canas, Hac. La Pacífica; 50 m; 15 Mar. 1976; Opler leg.; USNMENT01541941; USNM.

Guatemala • 1 ♂; **Izabal**, Pto. Barrios; 09 May. 1931; L. Bequaert leg.; *Plegavariegata* Navás det. N. Banks; FSCA. • 1 ♂; **Zacapa**, 12–14 Km S. San Lorenzo; 03 Jun. 1989; J.E. Wappes leg.; TAMU-ENTOX0285348; TAMUIC.

##### Other material.

Costa Rica – **Guanacaste** • 6 ♂ 10 ♀; Quebrada el Pedregal; 10°58'58.8"N, 85°32'20.4"W; 5 Feb. 1988; R.M. Strand leg.; *Plegahagenella* det. N. Penny, 92; UMSP. • 1 ♀; Quebrada Alcornoque; 11°0'32.4"N, 85°34'37.2"W; 21 Jan. 1988; R.M. Strand leg.; UMSP. • 1 ♂; Río Tempisquito; 10°57'28.8"N, 85°29'49.2"W; 15 Jan. 1988; R.M. Strand leg.; *Plegahagenella* det. N. Penny, 92; UMSP. • 1 ♀; Agua Buena, P.N. Guanacaste; 220 m; Feb. 1992; L-N 334800, 364100, INBIO CRI000904925; MNCR. • 1 ♀; same data as for preceding; L-N 334800, 364100, INBIO CRI000904930; MNCR. • 1 ♂; same data as for preceding; L-N 334800, 364100, INBIO CRI000904932; MNCR. • 1 ♂; same data as for preceding; L-N 334800, 364100, INBIO CRI000904937; MNCR. • 1 ♂; same data as for preceding; L-N 334800, 364100, INBIO CRI000904926; MNCR. • 1 ♂; same data as for preceding; L-N 334800, 364100, INBIO CRI000904939; MNCR. • 1 ♂; same data as for preceding; 21–24 Mar. 1993; E. López leg.; L-N-334800, 364100, INBIO CRI001196363; MNCR. • 1 ♂; same data as for preceding; 21–24 Mar. 1993; E. López leg.; L-N-334800, 364100, INBIO CRI001196364; MNCR. • 1 ♀; same data as for preceding; 21–24 Mar. 1993; E. López leg.; L-N-334800, 364100, INBIO CRI001196365; MNCR. • 1 ♂; same data as for preceding; 21–24 Mar. 1993; E. López leg.; L-N-334800, 364100, INBIO CRI001196366; MNCR. • 1 ♂; same data as for preceding; 8–23 Apr. 1993; E. López leg.; L-N-334800, 364100, INBIO CRI001331897; MNCR. • 1 ♀; same data as for preceding; 8–23 Apr. 1993; E. López leg.; L-N-334800, 364100, INBIO CRI001331898; MNCR. • 1 ♂; same data as for preceding; 240 m; 7–12 Feb. 1994; E. López; L-N-334800, 364100, INBIO CRI001747084; MNCR. 1 ♂; same data as for preceding; 240 m; 7–12 Feb. 1994; E. López; L-N-334800, 364100, INBIO CRI001747085; MNCR. • 1 ♀; Fca. Jenny, 30 Km N. de Liberia P.N. Guanacaste; Mar. 1991; R. Espinoza leg.; L-N-316200, 364400, INBIO CRI001326186; MNCR. • 1 ♀; same data as for preceding; L-N-316200, 364400, INBIO CRI001692729; MNCR. • 1 ♂; Est. Las Pailas, P.N. Ricón de la Vieja; 9–25 Feb. 1993; D. García leg.; L-N-306300, 388600, INBIO CRI001215244; MNCR. • 1 ♂; same data as for preceding; 7–19 Feb. 1994; 800 m; G. García leg.; L-N-306300, 388600, INBIO CRI002116224; MNCR. 1 ♀; same data as for preceding; 4–10 Apr. 1995; E. Navarro leg.; L-N-259150, 388500, INBIO CRI002219124; MNCR. • 1 ♀; Los Mesones, P.N. Barra Honda; 100 m; Mar. 1995; M. Reyes leg.; L-N-239200, 388500, INBIO CRI002179845; MNCR. • 1 ♀; same data as for preceding; L-N-239200, 388500, INBIO CRI002179844; MNCR. • 1 ♀; P.N. Barra Honda, de la oficina 900 m O.; 900 m; Feb. 1995; M. Reyes leg.; light trap; L-N-237200, 387800, INBIO CRI002178614; MNCR. • 1 ♀; Est. Maritza, lado O. Vol. Orosi; 600 m; Feb. 1992; F. Araya leg.; L-N-326900, 373000, INBIO CRI000737236; MNCR. • 1 ♀; same data as for preceding; L-N-326900, 373000, INBIO CRI000737237; MNCR. • 1 ♀; same data as for preceding; L-N-326900, 373000, INBIO CRI000737238; MNCR. • 1 ♀; same data as for preceding; L-N-326900, 373000, INBIO CRI000737239; MNCR. • 1 ♀; same data as for preceding; L-N-326900, 373000, INBIO CRI000737240; MNCR. • 1 ♂; same data as for preceding; L-N-326900, 373000, INBIO CRI000737241; MNCR. • 1 ♂; same data as for preceding; P. Campos leg.; L-N 326900, 373000, INBIO CRI000888332; MNCR. • 1 ♂; same data as for preceding; P. Campos leg.; L-N 326900, 373000, INBIO CRI000888333; MNCR. • 1 ♀; same data as for preceding; Feb. 1988; P. Campos leg.; Malaise trap; L-N-326900, 373000, INBIO CRI000294265; MNCR. • 1 ♀; same data as for preceding; 27 Feb.– 10 Mar. 1992; R. Vargas leg.; L-N-326900, 373000, INBIO CRI000468653; MNCR. • 1 ♀; same data as for preceding; 27 Feb.– 10 Mar. 1992; R. Vargas leg.; L-N-326900, 373000, INBIO CRI000468651; MNCR. • 1 ♀; same data as for preceding; 27 Feb.– 10 Mar. 1992; R. Vargas leg.; L-N-326900, 373000, INBIO CRI000468652; MNCR. • 1 ♀; same data as for preceding; same data as for preceding; 27 Feb.– 10 Mar. 1992; F.A. Quesada leg.; L-N-326900, 373000, INBIO CRI000888973; MNCR. • 1 ♂; same data as for preceding; Feb. 1992; F. Araya leg.; L-N-326900, 373000, INBIO CRI000737242; MNCR. • 1 ♀; same data as for preceding; 28 Feb.– 10 Mar. 1992; G. Gallardo leg.; L-N-326900, 373000, INBIO CRI000724745; MNCR. • 1 ♀; same data as for preceding; 27 Feb.– 10 Mar. 1992; R. Vargas leg.; L-N-326900, 373000, INBIO CRI000468649; MNCR. • 1 ♀; same data as for preceding; 27 Feb.– 10 Mar. 1992; R. Vargas leg.; L-N-326900, 373000, INBIO CRI000468650; MNCR. • 1 ♂; same data as for preceding; 27 Feb.– 10 Mar. 1992; R. Vargas leg.; L-N-326900, 373000, INBIO CRI000468553; MNCR. • 1 ♀; same data as for preceding; 27 Feb.– 10 Mar. 1992; F.A. Quesada leg.; L-N-326900, 373000, INBIO CRI000889003; MNCR. • 1 ♀; same data as for preceding; 27 Feb.– 10 Mar. 1992; A. Marín leg.; L-N-326900, 373000, INBIO CRI000735000; MNCR. • 1 ♀; same data as for preceding; 27 Feb.– 10 Mar. 1992; A. Marín leg.; L-N-326900, 373000, INBIO CRI000735021; MNCR. • 1 ♀; same data as for preceding; 27 Feb.– 10 Mar. 1992; A. Marín leg.; L-N-326900, 373000, INBIO CRI000734989; MNCR. • 1 ♂; same data as for preceding; 27 Feb.– 10 Mar. 1992; A. Marín leg.; L-N-326900, 373000, INBIO CRI000734990; MNCR. • 1 ♀; same data as for preceding; 27 Feb.– 10 Mar. 1992; A. Marín leg.; L-N-326900, 373000, INBIO CRI000734991; MNCR. • 1 ♂; same data as for preceding; 27 Feb.– 10 Mar. 1992; A. Marín leg.; L-N-326900, 373000, INBIO CRI000734993; MNCR. • 1 ♂; same data as for preceding; 27 Feb.– 10 Mar. 1992; M. Segura leg.; L-N-326900, 373000, INBIO CRI000789757; MNCR. • 1 ♀; same data as for preceding; 27 Feb.– 10 Mar. 1992; A. Marín leg.; L-N-326900, 373000, INBIO CRI000734999; MNCR. • 1 ♀; same data as for preceding; 27 Feb.– 10 Mar. 1992; A. Marín leg.; L-N-326900, 373000, INBIO CRI000734998; MNCR. • 1 ♀; same data as for preceding; 27 Feb.– 10 Mar. 1992; A. Marín leg.; L-N-326900, 373000, INBIO CRI000734994; MNCR. • 1 ♀; same data as for preceding; 27 Feb.– 10 Mar. 1992; M. Segura leg.; L-N-326900, 373000, INBIO CRI000789760; MNCR. 1 ♀; same data as for preceding; 28 Feb.– 10 Mar. 1992; C. Cano leg.; L-N-326900, 373000, INBIO CRI000750668; MNCR. 1 ♀; same data as for preceding; 27 Feb.– 10 Mar. 1992; A. Marín leg.; L-N-326900, 373000, INBIO CRI000734995; MNCR. • 1 ♀; same data as for preceding; 27 Feb.– 10 Mar. 1992; M. Segura leg.; L-N-326900, 373000, INBIO CRI000789756; MNCR. • 1 ♀; same data as for preceding; 27 Feb.– 10 Mar. 1992; M. Segura leg.; L-N-326900, 373000, INBIO CRI000789758; MNCR. • 1 ♂; same data as for preceding; 27 Feb.– 10 Mar. 1992; M. Segura leg.; L-N-326900, 373000, INBIO CRI000789761; MNCR. • 1 ♀; same data as for preceding; 27 Feb.– 10 Mar. 1992; K. Flores leg.; L-N-326900, 373000, INBIO CRI000751084; MNCR. • 1 ♀; same data as for preceding; 27 Feb.– 10 Mar. 1992; K. Flores leg.; L-N-326900, 373000, INBIO CRI000751085; MNCR. • 1 ♀; same data as for preceding; 27 Feb.– 10 Mar. 1992; K. Flores leg.; L-N-326900, 373000, INBIO CRI000751086; MNCR. • 1 ♀; same data as for preceding; 27 Feb.– 10 Mar. 1992; M. Segura leg.; L-N-326900, 373000, INBIO CRI000789755; MNCR. • 1 ♀; same data as for preceding; 27 Feb.– 10 Mar. 1992; M. Segura leg.; L-N-326900, 373000, INBIO CRI000789762; MNCR. • 1 ♀; same data as for preceding; 27 Feb.– 10 Mar. 1992; K. Flores leg.; L-N-326900, 373000, INBIO CRI000751083; MNCR. • 1 ♂; same data as for preceding; 27 Feb.– 10 Mar. 1992; C. Cano leg.; L-N-326900, 373000, INBIO CRI000750667; MNCR. • 1 ♂; same data as for preceding; 27 Feb.– 10 Mar. 1992; K. Flores leg.; L-N-326900, 373000, INBIO CRI000751087; MNCR. • 1 ♀; same data as for preceding; 28 Feb.– 10 Mar. 1992; C. Cano leg.; L-N-326900, 373000, INBIO CRI000750666; MNCR. • 1 ♀; same data as for preceding; 28 Feb.– 10 Mar. 1992; C. Cano leg.; L-N-326900, 373000, INBIO CRI000750665; MNCR. • 1 ♀; same data as for preceding; 27 Feb.– 10 Mar. 1992; M. Segura leg.; L-N-326900, 373000, INBIO CRI000789764; MNCR. • 1 ♀; same data as for preceding; 28 Feb.– 10 Mar. 1992; K. Taylor leg.; L-N-326900, 373000, INBIO CRI000394922; MNCR. • 1 ♀; same data as for preceding; 28 Feb.– 10 Mar. 1992; K. Taylor leg.; L-N-326900, 373000, INBIO CRI000394921; MNCR. • 1 ♀; same data as for preceding; 28 Feb.– 10 Mar. 1992; F.A. Quesada leg.; L-N-326900, 373000, INBIO CRI000889023; MNCR. • 1 ♀; same data as for preceding; 28 Feb.– 10 Mar. 1992; D. García leg.; L-N-326900, 373000, INBIO CRI000535627; MNCR. • 1 ♀; same data as for preceding; 28 Feb.– 10 Mar. 1992; R. Guzmán leg.; L-N-326900, 373000, INBIO CRI000441323; MNCR. • 1 ♀; same data as for preceding; 28 Feb.– 10 Mar. 1992; M. Reyes leg.; L-N-326900, 373000, INBIO CRI000439924; MNCR. • 1 ♀; same data as for preceding; 27 Feb.– 10 Mar. 1992; F.A. Quesada leg.; L-N-326900, 373000, INBIO CRI000889051; MNCR. • 1 ♀; Est. Maritza, Quebrada Yeguitas, A.C.G.; 600 m; 21 Feb. 1994; L-N-326900, 373000, INBIO CRI001804019; MNCR. • 1 ♂; Est. Maritza, Tempisquito Sur, A.C.G.; 600 m; 20–23 Feb. 1994; L-N-325800, 373700, INBIO CRI001682886; MNCR. • 1 ♂; same data as for preceding; L-N-325800, 373700, INBIO CRI001682885; MNCR. • 1 ♀; same data as for preceding; L-N-325800, 373700, INBIO CRI001682884; MNCR. • 1 ♀; same data as for preceding; L-N-325800, 373700, INBIO CRI001682883; MNCR. • 1 ♂; same data as for preceding; L-N-325800, 373700, INBIO CRI001682882; MNCR. • 1 ♂; same data as for preceding; L-N-325800, 373700, INBIO CRI001682881; MNCR. • 1 ♂; same data as for preceding; L-N-325800, 373700, INBIO CRI001682880; MNCR. • 1 ♀; same data as for preceding; L-N-325800, 373700, INBIO CRI001682876; MNCR. • 1 ♀; same data as for preceding; L-N-325800, 373700, INBIO CRI001682875; MNCR. • 1 ♂; same data as for preceding; L-N-325800, 373700, INBIO CRI001682877; MNCR. • 1 ♀; same data as for preceding; L-N-325800, 373700, INBIO CRI001682878; MNCR. • 1 ♂; Playa Naranjo, Sta. Rosa; Dec. 1990; E. Alcazar leg.; L-N-309300, 353300, INBIO CRI000486473; MNCR. • 1 ♂; same data as for preceding; L-N-309300, 353300, INBIO CRI000486471; MNCR. • 1 ♂; La Cruz, P.N. Guanacaste, Casa Oeste, Cerro el Hacha; 400–500 m; 4 Feb. 1988; A. Solís leg.; L-N-331700, 365400, INB 0003353138; MNCR. • 1 ♀; La Cruz, P.N. Guanacaste, Santa Cruz, Vista del Mar, Torre CoCENSA; 927 m; 10 Feb. 2003; W. Porras leg.; L-N-235350, 357500, INB 0003703247; MNCR. • 1 ♀; Est. Palo Verde; 20–23 Feb. 1994; 10 m; R. Chavarría leg.; L-N-259000, 388400, INBIO CRI001739708; MNCR. • 1 ♂; Est. Palo Verde; 25 Mar.–21 Apr. 1992; 10 m; A. Gutierrez leg.; L-N-259000, 388400, INBIO CRI000438906; MNCR. • 1 ♀; Est. Quebrada Bonita. 50 m R.B. Carara; 800 m; Mar. 1993; R. Guzmán leg.; L-N-194500, 469850, INBIO CRI000379570; MNCR. • 1 ♀; Santa Rosa National Park; Apr. 1985; 300 m; D.H. Janzen & W. Hallwachs leg.; INBIO CRI000688706; MNCR. • 1 ♂; same data as for preceding; INBIO CRI001688702; MNCR. • 1 ♀; same data as for preceding; INBIO CRI001688703; MNCR. • 1 ♂; same data as for preceding; INBIO CRI001688698; MNCR. • 1 ♀; Est. Murcielago, A.C.G.; 600 m; 9–19 Feb. 1994; F. Quesada leg.; L-N-320300, 347200, INBIO CRI000744649; MNCR. • 1 ♀; same data as for preceding; 100 m; L-N-320300, 347200, INBIO CRI000744648; MNCR. • 1 ♂; same data as for preceding; 100 m; L-N-320300, 347200, INBIO CRI001744652; MNCR. • 1 ♀; same data as for preceding; 100 m; L-N-320300, 347200, INBIO CRI001744651; MNCR. • 1 ♀; Finca Jenny, 30Km N de Liberia; 240 m; 8–15 Mar. 1994; E. Araya leg.; L-N-317150, 363700, INBIO CRI001737006; MNCR. – **Limón** • 1 ♂; Cuatro Esquinas, P.N. Tortuguero; 0 m; Sep. 1989; J. Solano leg.; 280000, 590500, INBIO CRI000052838; MNCR. – **Puntarenas** • 1 ♀; Est. Quebrada Bonita. R.B. Carara; 50 m; Mar. 1994; J. Saborio leg.; L-N-194500, 469850, INBIO CRI000681096; MNCR.

##### Diagnosis.

This species has a brown flagellum, with basal flagellomeres as long as wide. The femur posterior surface is pale with numerous dark brown dots; the anterior surface is mainly dark brown with small pale areas. The pterostigma of both wings is brown with a large, medial, irregular whitish area, and additional surrounding pale areas. On the male genitalia, the gonocoxite IX is thin, gently curved, and sinusoid in ventral view, with posterior apex straight, and set with three apical, digitiform processes, subequal in shape and size, situated on the inner surface. The gonocoxites XI medial lobe, has the ventral part as a lobe with a keel on the center, which is covered with minute granules. On the female genitalia, the gonapophyses VIII medial part is boat-shaped, posteromedially set with a small, bilobed process. The bursa copulatrix is long, semi-membranous, and striated, while the spermatheca has the distal section slightly expanded, and with a rounded diverticulum.

##### Etymology.

The specific epithet *pseudohagenella* is a composite of the Greek *ψευδής* (*pseudḗs*) meaning false, and *hagenella*, the specific name of a closely related species of the genus *Plega*. This name refers to the resemblance between both species and the difficulty of recognizing them by previous researchers, who always identified this species as *P.hagenella*.

##### Description.

***Measurements*.** Male (*n* = 6). Forewing length: 8.37–16.70 mm; Hind wing length: 6.72–13.97 mm. Female (*n* = 4): Forewing length: 7.22–16.29 mm; Hind wing length: 6.29–13.04 mm.

**Figure 61.**
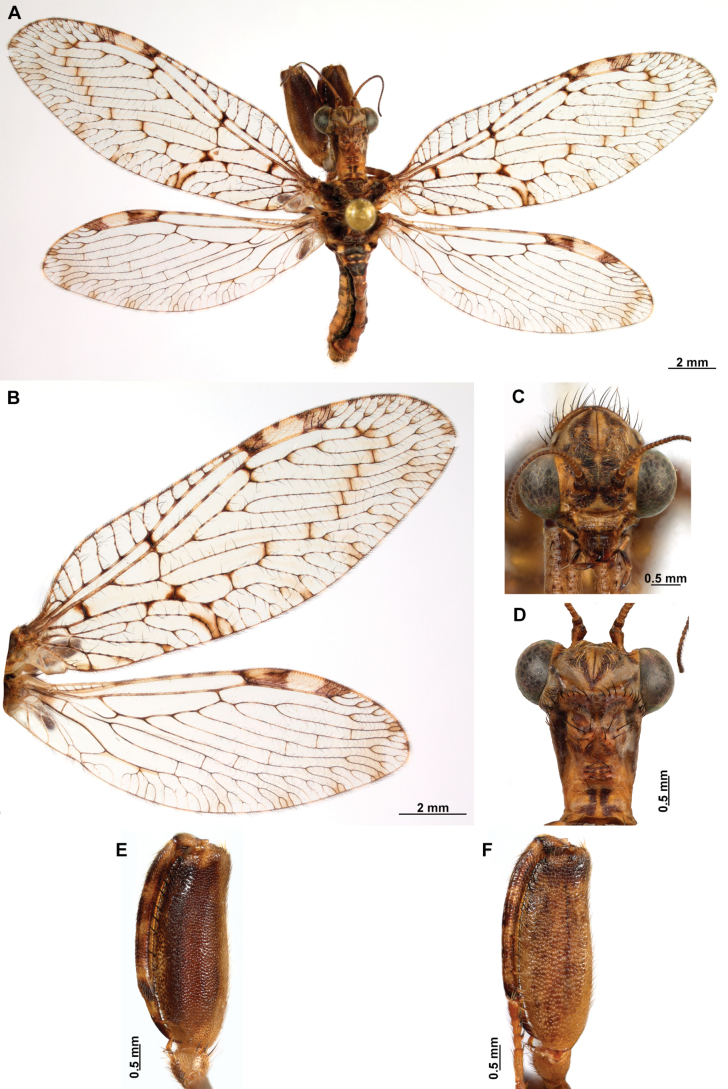
*Plegapseudohagenella* Ardila-Camacho & Contreras-Ramos, sp. nov. **A** male habitus **B** wings **C** head, frontal **D** pronotum, dorsal **E** forefemur, anterior surface **F** same, posterior surface.

***Coloration*** (Fig. 61). ***Head*.** Vertexal region pale with brown lateral markings extending from occiput to toruli, forming a V-shaped pattern; pale brown setae on brown marks present; area adjacent to occipital ridge dark brown; paraocular area bicolor; postgena either pale with brown marks or almost completely brown. Antennal scape mostly brown, with a pale, longitudinal, dorsolateral band, dark brown setae present; pedicel dark brown, flagellum brown with dark brown setae. Frons pale with dark brown markings on center and below toruli. Clypeus pale with dark brown lateral markings; labrum pale brown with dark brown anterior margin; mandible pale brown at base, dark amber at apex; maxilla pale with dark brown markings, palpus dark brown, except for third article pale brown, and pale junctions; labium dark brown with pale margins, labial palpus dark brown, pale at junctions, palpimacula brown. ***Thorax*.** Pronotum brown with dark brown lateral stripes, pale posterolateral areas, dark brown setae, sternal surface pale; episternum brown, postfurcasternum pale. Mesonotum with broad, pale, median band and dark brown lateral stripes, setation dark brown; scutellum with some pale brown setae; metanotum dark brown laterally, paler medially; pre-episternum pale with brown suffusions; pteropleura pale with brown markings, interspersed pale and brown setae present. ***Foreleg*.** Coxa brown with interspersed pale and dark brown setae; trochanter either pale or brown with pale areas, most of the surface with pale brown setae, dark brown on dorsal surface. Femur posterior surface pale with numerous dark brown spots and dark brown apex; anterior surface mainly dark brown with small pale areas. Tibia dashed with pale and dark brown, with pale brown setae. Basitarsus brown on proximal region, with broad pale area on basal ½, clavate setae pale brown; lanceolate process dark amber; remaining tarsomeres pale brown. ***Mid- and hind leg*.** Coxae dark brown with small pale areas, almost completely pale in some specimens, pale brown setae present; mid-trochanter pale with brown spot on the posterior surface; hind trochanter pale with brown spot on the anterior surface. Femora and tibiae dashed, alternating pale and brown, setae pale brown on pale areas and brown on dark areas. Tarsomeres pale to pale brown with pale brown setae, distal margins on ventral surface with dark brown setae; pretarsal claws amber. ***Wings*.** Forewing mostly hyaline, pale amber in some specimens; membrane surrounding crossveins, marginal twigging, first branch of CuA and apex of CuP dark amber; pterostigma brown with a large, medial, irregular pale area; major veins alternating pale and dark brown, crossveins dark brown. Hind wing mostly hyaline, with pale amber infuscation near the posterior and distal margins; base of subcostal space and *mcu1* amber; pterostigma dark brown with two pale areas, one wide and medially located, and another proximal and smaller, which sometimes are fused; longitudinal veins and crossveins alternating pale and brown. ***Abdomen*.** Tergite I dark brown with pale lateral areas, tergites II-IX brown with pale margins; tergites III-VII with pale lateral scars, with longer one anterolaterally on each side. Tergite IX pale with brown markings. All segments with pale setae. Some specimens with tergites completely brown; pleural membrane dark brown with ventral pale areas. Sternites with broad, pale medial and lateral areas; lateral bands extended from anterior margin to merge with narrow, transverse stripe near posterior margin. Sternite VIII with pale posterior 1/2 with medial brown mark, anterior ½ brown. Sternite laterally pale, dark brown on center, setae pale brown, darker on dark brown areas. Female tergites brown with pale areas, pale brown setae present; sternites with pale posterolateral areas, medial region pale to dark brown, with semi-triangular lateral markings on each side, pale brown setae present; sternite VII pale medial and posterolateral areas, laterally with dark brown setae.

***Morphology*** (Fig. 61). ***Head*.** Diamond-shaped in frontal view, rugose, vertexal region raised above compound eyes, with lateral rows of reclined setae on brown areas; area between coronal suture glabrous, with muscle insertion mark; coronal suture distinct; medial area of ​​supra-antennal region with lateral outgrowths located above toruli, covered with reclined setae; paraocular area shallowly concave. Antenna filiform, short; scape 1.5× as long as wide, slightly distally expanded, with short setae, except near distal margin thicker; pedicel as long as wide; flagellum with 29–42 flagellomeres, as wide as long on proximal ½, slightly longer than wide on distal ½; all articles with medial ring of short setae, except the most basal with scattered setae. Compound eye hemispherical, as wide as ½ of the interocular distance at torulus level. Frons and clypeus rugose, with fine and short setae. Labrum pentagonal with fine and long setae; maxillary palpus with first palpomere as long as wide, second 1.5× as long as wide, third palpomere 3× as long as wide, fourth palpomeres 2.5× as long as wide, fifth palpomere slightly longer than third, with short and thin setae; mentum with fine, long setae; labial palpus with first palpomere two times as long as wide, second palpomere 4× as long as wide, third palpomere slightly shorter than second, palpimacula elongate and ovoid. ***Thorax*.** Pronotum as long as wide, subtrapezoidal, with raised anterior margin, medial and posterior regions; outgrowths covered with pedicellate, thickened setae, remaining surface with fine, short setae. Mesonotum 2× as wide as long, with scattered, thick, pedicellate setae and some long, thin setae on medial area. Metanotum ~ 2× as long as wide, mostly glabrous with few fine setae on scutellum. Pteropleura with short, thin setae and thick setae on mesanepisternum and mesokatepisternum and on metakatepisternum. ***Foreleg*.** Coxa as long as femur, cylindrical, anterior and posterior surfaces with pedicellate fine or thick setae of different sizes; trochanter subtrapezoidal, with thin and short setae, except dorsally with some thick and longer setae, anterior surface with protuberant area covered with pedicellate, thick setae. Femur robust, covered with abundant, fine, short setae which arise from protuberant bases on both surfaces. Closing surface with posteroventral row of processes composed two medially located primary processes and a sub-basal secondary process; between these processes a single tertiary process, and abundant tubercle-shaped processes and stinger-shaped setae are preset; distal portion of posteroventral row raised, composed of tubercle-shaped specializations of different sizes and stinger-shaped setae; adjacent row of thickened setae with globular base present on distal ½. Anteroventral row of processes reduced to proximal ⅓ which is composed of tubercle-shaped specializations, and stinger-shaped setae; the basal-most process, a primary, curved process; distal portion of anteroventral row composed tubercle-shaped specializations; adjacent row of thickened setae with globular base present on distal ¼–½. Tibia almost as long as the femur, curved, moderately setose, ventrally keeled with prostrate setae; a patch of clavate setae is present apically on the anterior surface. Basitarsus with lanceolate process reaching the middle of fourth tarsomere; clavate setae are present proximally on anterior surface; ventrally with single row of prostrate setae; second tarsomere ~ 6× as long as wide; third tarsomere as long as wide, fourth tarsomere two times as long as wide. ***Mid- and hind leg*.** Coxae with long, thick setae; trochanter with fine and short setae; femora and tibiae on both legs with interspersed fine, short and long setae; tibial spurs short; hind leg longer than midleg, tibia, 1.5× as long as femur; tarsi with fine and short setae, except on distal margin of plantar surface with lateral groups of 5–7 thickened setae; on both legs basitarsus 3.5 times as long as wide, second tarsomere 1.5× as long as wide; third and fourth tarsomeres as long as wide; fifth tarsomere two times as long as wide. ***Wings*.** Forewing narrowly oval, trichosors present along margin except on wing base; venation setose; costal space proximally expanded, humeral vein branched, 11–15 subcostal veinlets; pterostigma elongated, curved, with barely perceptible veinlets; subcostal space with single crossvein, medially located; Sc vein abruptly posteriorly bent at proximal pterostigma margin to merge with RA; radial space with two crossveins; *rarp2* curved with 2–4 RP branches arising from it, 2–4 veins arising from *rarp1*; M vein fused basally with RA; RP base widely separated from point of divergence of M from R; M forked slightly before RP base, 1 r-m connecting RP base and M fork, forming a trapezoidal cell; 4–8 gradate crossveins present. Cubitus deeply forked; CuP basally angled and approaching A1, distally forked at level of separation of M and R; A1 apically forked, ending on posterior margin at CuP fork level, A2 forked at CuP angle level. Hind wing smaller and narrower than forewing, narrowly oval; costal space narrow and reduced, with 4–8 veinlets; C and Sc fused at ¼ of wing length, Sc vein abruptly curved posteriorly at proximal margin of pterostigma to merge with RA; pterostigma elongated, curved, slightly expanded basally, composed poorly defined veinlets; radial space with single crossvein, oblique; three or four veins arising from *rarp1*, two or three from *rarp2*. 1r-m sigmoid, connecting the stems of M and RP. Media forked beyond R fork. Cubitus deeply forked, intracubital crossvein subparallel to longitudinal wing axis; CuA sinuous, first branch candelabrum-shaped, spur vein sometimes present; CuP not touching A1, strongly anteriorly bent at distal 1/3, sometimes touching posterior wing margin, with three or four branches; two crossveins on cubitoanal space; A1 simple ending on wing margin slightly beyond 1m-cu level; A2 simple, short, and curved. ***Abdomen*.** Cylindrical to medially expanded, setae on tergites gradually longer and more abundant towards terminal segments; tergites I–III with few, scattered, fine setae, tergites III-VII with elongated anterolateral scars. Sternites with abundant fine and long setae, sternites III-VII preapically with an elongated, transverse scar.

**Figure 62.**
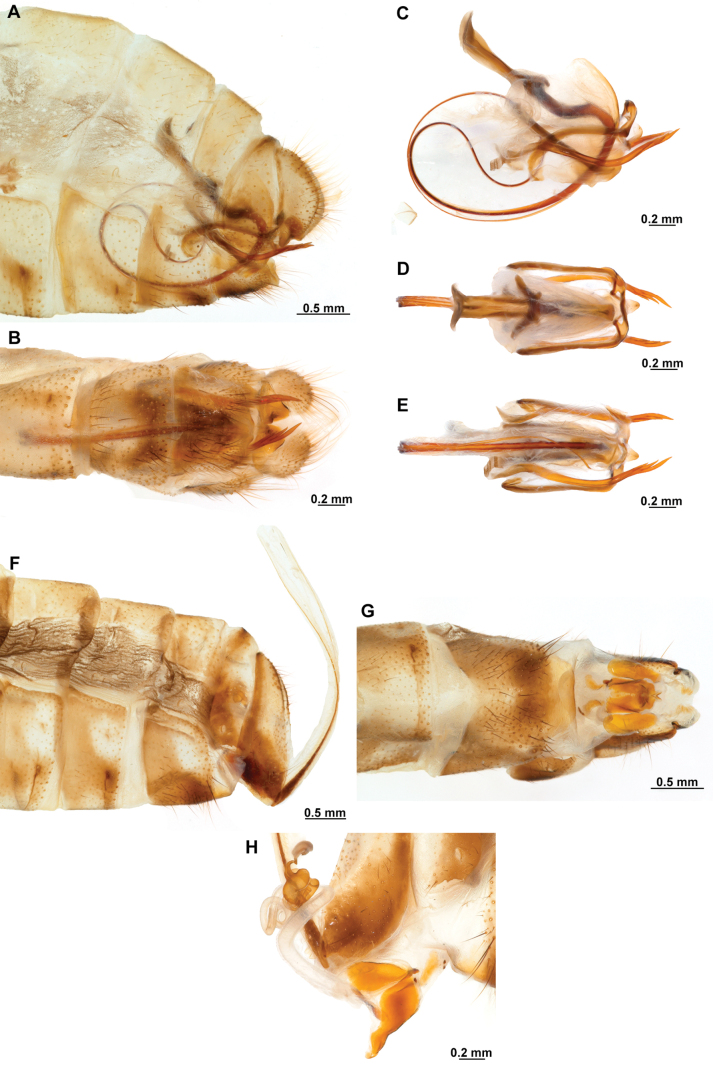
*Plegapseudohagenella* Ardila-Camacho & Contreras-Ramos, sp. nov. **A** male terminalia, lateral **B** same, ventral **C** male genitalia, lateral **D** same, dorsal **E** same, ventral **F** female terminalia, lateral **G** same, ventral **H** gonapophyses VIII and spermatheca, lateral.

***Male genitalia*** (Fig. 62A–E). Tergite IX medially remarkably narrower than laterally, lateral margin rounded. Sternum VIII rectangular, setose; sternite IX subtrapezoidal in ventral view, setose, with rounded posterolateral lobes, covered with abundant fine and long setae, posteromedially produced into a blunt lobe with canaliculate dorsal surface; in lateral view trapezoidal, apex not reaching posterior margin of ectoproct; posteromedial lobe ventrally curved. Gonocoxites IX thin, gently curved, sinusoid in ventral view; base dorsally curved, connected to gonocoxites XI with a membrane; posterior apex straight, with three apical digitiform processes, subequal in shape and size, situated on inner surface. Ectoproct ovoid, covered with abundant, thin setae; anteroventrally with sclerotized, rounded lobe, which is continuous with sclerotized, ventromedial, curved sulcus; entire surface covered with microtrichia. Gonocoxites X forming a short, thickened, ventrally canaliculate sclerite, anterior apex expanded and dorsally curved, posterior apex with dorsal processes connected to gonostyli X, ventrolateral processes connected to gonapophyses X with a membrane; gonostyli X with thickened and concave base with two lateral processes, the rest of the structure, ribbon-shaped, ventrally curved and anteriorly coiled, forming two loops before protruding from abdomen. Gonapophyses X rod-shaped, straight, narrow, arranged in a V-shaped structure with apexes dorsally bent; the gonapophyses are joined by a sclerotized membrane covering the gonostyli X base, whose lateral surface is covered with microspinules. Gonocoxites XI, U-shaped, medial lobe complex and elaborated with two differentiated parts: a dorsal trapezoidal lobe, and a ventral lobe with a keel on the center, which is covered with minute granules; between these parts a quadrangular, less sclerotized, hyaline area is present; lateral arms of gonocoxites XI gently curved, anterior apex expanded, and curved ventrad. Hypandrium internum triangular, concave with medial keel.

***Female genitalia*** (Fig. 62F–H). Sternum VII trapezoidal, slightly narrower medially than laterally, setose, posteromedially continuous with a sclerotized, glabrous area. Tergite VIII slightly narrower medially than laterally, encircling the spiracle of the segment. Gonocoxites VIII as a small, rounded, moderately sclerotized plate. Gonapophyses VIII as two medially joined trapezoidal plates forming a boat-shaped structure, posteromedially with a small, bilobed process; laterally to medial plates an oval plate folded beneath tergite IX + ectoproct is present. Tergite IX + ectoproct triangular. Gonocoxites IX elongated, narrow, as long as the last four abdominal segments together. Bursa copulatrix long, funnel-shaped, semi-membranous, striated. Spermatheca complexly entangled, proximal section, spiral-shaped forming several coils; medial section narrower than proximal section, entangled; distal section slightly expanded, with rounded diverticulum; fertilization canal duct proximally expanded, subconical, concave; fertilization canal J-shaped, covered with microfilaments.

##### Distribution.

Costa Rica (El Limón, Guanacaste, Puntarenas), Guatemala (Izabal, Zacapa).

##### Remarks.

This species is probably restricted to Central America and until now only known from Costa Rica and Guatemala. This species is superficially similar to *P.hagenella* and indeed both species overlap their distribution in Central America. For this reason, this species was always identified as *P.hagenella* (Hoffman 2002) and basically in all museums and collections where there is material all the specimens have identification labels of *P.hagenella* (e.g., Museo Nacional de Costa Rica-MNCR, California Academy of Sciences-CAS, and University of Minnesota-UMSP). *Plegapseudohagenella* was recovered as an intermediary species between the South American clade and a large clade containing Central American, Mexican and Nearctic species. It is remarkably similar to *P.stangei* and *P.vangiersbergenae* in the general coloration pattern and genital morphology, although it can be differentiated by the noticeably robust forefemur. Despite the male gonocoxites IX are similar to those of *P.hagenella*, this species lacks the long, preapical process and the morphology of other genital sclerites is completely different. Additionally, the antennal flagellum is uniformly brown whereas in *P.hagenella* it has pale preapical flagellomeres.

#### ﻿﻿ Plega radicaudata

Taxon classificationAnimaliaNeuropteraRhachiberothidae

Ardila-Camacho & Contreras-Ramos sp. nov.

https://zoobank.org/C3E7B5C2-DE19-4A27-9A8C-52381D8DB2D5

Figs 63 , 64

##### Type locality.

Peru, **Madre de Dios**: Manu, Pakitza, 12°7'S, 70°58'W; 250 m; 9–23 Sep. 1988; O. Flint & N. Adams leg.

##### Material examined.

***Holotype*** male, pinned. Original label: “Peru, **Madre de Dios**, Manu, Pakitza, 12°7'S, 70°58'W, 250 m, 9–23 Sep 1988, O. Flint & N. Adams leg., USNMENT01541942” USNM. ***Paratypes***. Peru • 1 ♂; **Madre de Dios**, Manu, Pakitza; 12°7'S, 70°58'W; 250 m; 9–23 Sep. 1988; O. Flint & N. Adams leg.; USNMENT01541949; USNM. • 1 ♀; same data as for preceding; USNMENT01541944; USNM. • 1 ♂; same data as for preceding; USNMENT01541940; USNM. • 1 ♀; Madre de Dios, Manu Biosphere Res, Aguajal; 250 m; 12°07'S, 70°58'W; 12 Sep. 1988; M.G. Pogue leg.; blacklight trap; USNMENT01541939; USNM. • 1 ♀; same data as for preceding; USNMENT01541938; USNM.

##### Other material.

Bolivia • 1 ♂; [**Santa Cruz**] Prov. Del Sara; 450 m; J. Steinbach leg.; FSCA.

##### Diagnosis.

This species has the basal antennal flagellomeres as wide as long; five pale, preapical flagellomeres are present. The forefemur is elongated and narrow, with both rows of integumentary specializations fully developed; the posterior surface is pale with brown dots, while the anterior surface is pale with extensive brown areas. The forewing is oval, with the medial pale area of the pterostigma anteriorly notched; the apex of the hind wing is amber. On the male genitalia, the gonocoxite IX is thin, sinusoid, and long; the posterior apex is set with long and thin preapical process on inner surface, and 2–4 apical shorter, thin processes. The membrane adjacent to posterior apexes of gonapophyses X has a pair of sclerites covered with microspinules. The medial lobe of gonocoxites XI has the ventral part concave, continuous with a ventral, caudally curved, trapezoidal process, whose apex is covered with microspinules. On the female genitalia, the gonapophyses VIII medial part is boat-shaped with posteromedial, dorsally curved, elongated process whose apex is bilobed. The spermatheca is not strongly elongated; the proximal section is thin, proximally forming few loops, while the rest is zigzagged; the distal section lacks a diverticulum.

##### Etymology.

The specific epithet of this species is combination of the Latin words *radix* meaning root and *cauda* meaning tail, in reference to the root-shaped apex of the male gonocoxite IX of this species. An adjective in the nominative case.

##### Description.

***Measurements*.** Male (*n* = 4). Forewing length: 11.3–14.3 mm; Hind wing length: 8.7–11.2 mm. Female (*n* = 3): Forewing length: 11.29–13.2 mm; Hind wing length: 9.2–10.4 mm.

**Figure 63.**
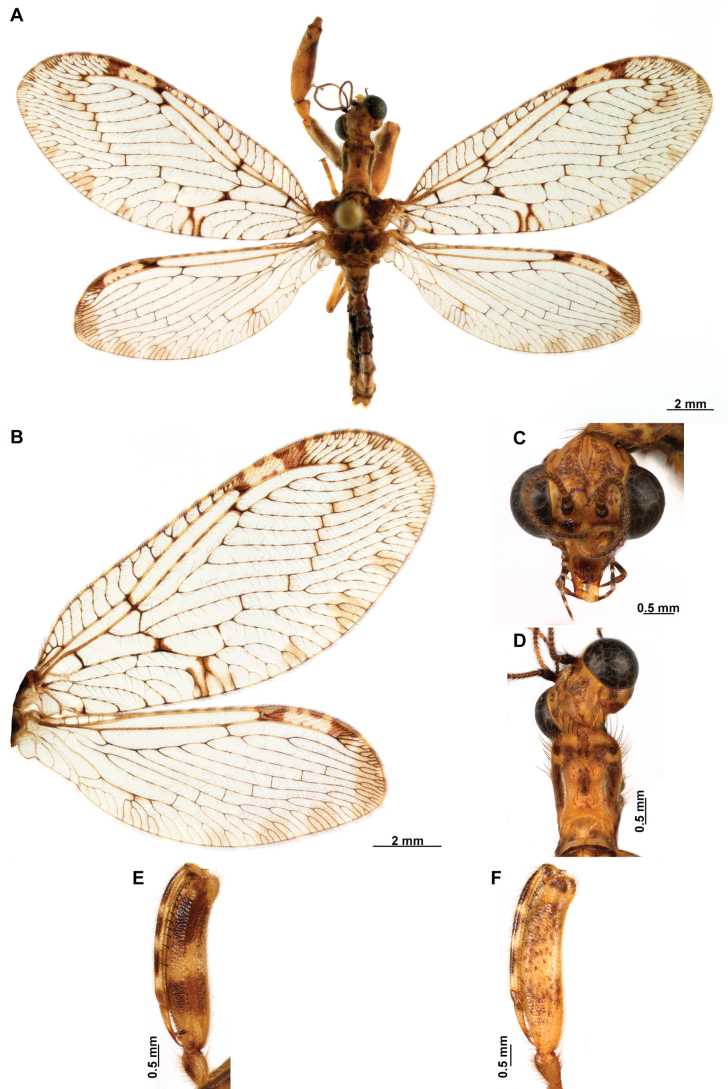
*Plegaradicaudata* Ardila-Camacho & Contreras-Ramos, sp. nov. **A** male habitus **B** wings **C** head, frontal **D** pronotum, dorsal **E** forefemur, anterior surface **F** same, posterior surface.

***Coloration*** (Fig. 63). ***Head*.** Vertexal region pale, with irregular, lateral brown marks extending from occiput to supra-antennal area, with pale brown setae; supra-antennal pale with irregular brown marks, with pale brown setae; occiput and postgena brown with paler areas. Antennal scape either dark brown or brown with pale dorsal longitudinal band, pale to dark brown setae present; pedicel dark brown, flagellum pale to dark brown, with five pale, preapical flagellomeres. Frons pale with lateral triangular dark brown marks below the toruli. Clypeus pale or with brown, medial suffusions; labrum pale brown; mandible pale with dark brown corners, apex amber; maxilla pale, palpus dark brown; labium pale with brown postmentum and pale brown ligula; labial palpus dark brown, palpimacula pale brown. ***Thorax*.** Pronotum mostly pale with brown medial areas and lateral bands, interspersed pale and dark brown setae present; episternum brown, postfurcasternum with broad brown lateral area. Mesonotum mostly brown with pale medial and anterolateral areas, setation brown; metanotum brown with small, medial, pale areas; pre-episternum brown; pteropleura brown with pale areas, setation mostly pale brown. ***Foreleg*.** Coxa pale brown; trochanter brown darker, anterior area, setation mostly pale brown. Femur posterior surface pale with brown dots, sometimes with small, brown area on apex; anterior surface with extensive brown marks and pale areas. Tibia dashed with pale and brown, setae pale brown. Basitarsus with proximal pale area, base and lanceolate process pale brown, clavate setae pale brown; remaining tarsomeres pale brown. ***Mid- and hind leg*.** Coxae brown with pale areas, setae pale brown; trochanter pale with brown mark. Femora and tibiae of both legs pale with brown rings, setae mostly and tibial spurs pale brown. Tarsi pale brown; distal margins on ventral surface with dark brown setae; pretarsal claws brown. ***Wings*.** Forewing mostly hyaline; membrane surrounding crossveins, first branch of CuA and apex of CuP amber; posterior and apical margins on distal ½ of wing with intermittent, amber areas between apical branches of longitudinal veins; pterostigma brown with enlarged, pale medial area, which has medial brown spots on anterior and posterior margins; major veins, subcostal veinlets, and wing margin alternating pale and brown. Hind wing hyaline, with amber intermittent amber areas between apical branches of longitudinal veins adjacent to posterior margin; wing apex amber; pterostigma brown with wide, pale, medial area which has brown spots on anterior and posterior margins; longitudinal veins alternating pale and brown, proximal ½ of Cu and anal veins pale; subcostal veinlets pale brown, crossveins brown, except sigmoid 1r-m, bicolor; wing margin alternating pale and brown. ***Abdomen*.** Tergites pale with pale reddish brown suffusions. Pleural membrane dark brown. Sternites nearly completely brown.

***Morphology*** (Fig. 63). ***Head*.** Diamond-shaped in frontal view, rugose; vertexal region raised above

compound eyes, with lateral rows of reclined, fine setae; area surrounding coronal suture glabrous, with muscle insertion mark; coronal suture distinct; medial area of ​​supra-antennal moderately raised, with fine, reclined setae; paraocular area concave. Antenna submoniliform, short; scape two times as long as wide, slightly distally expanded, with fine, short setae; pedicel slightly longer than wide; flagellum not dorsoventrally flattened, with 37–43 flagellomeres, which are as wide as long on most of the flagellum, and slightly longer than wide near the apex; all articles with medial ring of fine, short setae. Compound eye hemispherical, as wide as ½ of the interocular distance at torulus level. Frons and clypeus narrow, with fine and short setae. Labrum pentagonal with thin, short setae; maxillary palpus with first palpomere as long as wide, second 1.5× as long as wide, third palpomere 3.5 times as long as wide, fourth palpomere 3× as long as wide, fifth slightly longer than third, all with minute setae; mentum with long, thin setae; labial palpus with first palpomere two times as long as wide, second palpomere 4× as long as wide, third palpomere as long as second, palpimacula ovoid. ***Thorax*.** Pronotum slightly longer than wide, with raised anterior margin, medial and posterior regions; outgrowths covered with pedicellate, thick setae; remaining surface with fine, short setae. Mesonotum slightly wider than long, with scattered, thick, pedicellate setae on medial area. Metanotum ~ 2× as long as wide, with a few fine, short setae. Pteropleura with interspersed short and long, thin setae. ***Foreleg*.** Coxa as long as femur, cylindrical, anterior and posterior surfaces with fine setae of different lengths; trochanter trapezoidal, with thin and short setae, except on dorsal and anterior surfaces with some thickened setae; anterior surface with protuberant area. Femur robust, elongate, and narrow, covered with abundant, fine, short setae, those of anterior surface arising from protuberant bases; closing surface with posteroventral row of processes composed two medially located, primary processes, with or without a tertiary process between them; proximal portion with basal tertiary process and sub-basal secondary process; the rest of the row with numerous tubercle-shaped processes, and stinger-shaped setae; distal portion raised, composed of tubercle-shaped specializations and stinger-shaped setae; adjacent row of thickened setae with globular base present on distal ¼. Anteroventral row of processes complete, composed of tubercle-shaped specializations and stinger-shaped setae; the basal-most primary, curved process is present; adjacent row of thickened setae with globular base present on distal ½. Tibia almost as long as femur, curved, with thin, short setae; ventral surface keeled with prostrate setae; a patch of clavate setae apically on anterior surface is present. Basitarsus with lanceolate process reaching the middle of fourth tarsomere; clavate setae present proximally on anterior surface; ventrally with single row of prostrate setae; second tarsomere nearly 6× as long as wide; third tarsomere 1.5× as long as wide, fourth tarsomere 2.5× as long as wide. ***Mid- and hind leg*.** Coxae and trochanter with long, thin setae; femora and tibiae of both legs with abundant interspersed fine setae of different lengths; tibial spurs short. Hind leg longer than midleg, tibia nearly two times as long as femur; tarsi with fine and short setae, except on distal margin of plantar surface with lateral groups of three or four thickened setae; on both legs, basitarsus 4× as long as wide, second tarsomere 1.2× as long as wide; third and fourth tarsomeres as long as wide; fifth tarsomere two times as long as wide. ***Wings*.** Forewing oval, trichosors present along margin except on wing base; venation setose; costal space proximally moderately expanded, humeral vein branched, 12–16 subcostal veinlets which may be simple or forked; pterostigma elongated, narrow, trapezoidal, gently curved, with most of its veinlets incomplete; subcostal space with single crossvein, located at R fork level; Sc vein abruptly posteriorly bent at proximal pterostigma margin to merge with RA; radial space with two crossveins; *rarp2* curved with three or four RP branches; three or four veins arising from *rarp1*; M vein basally fused with RA; RP base not widely separated from divergence of M and R; M forked slightly before RP origin, 1 r-m connecting RP base and M fork, forming a trapezoidal cell; 5–7 gradate crossveins present. Cubitus deeply forked; CuP basally angled and approaching A1, distally forked opposite to the level of separation of M and R; A1 simple or apically forked, ending on posterior margin at level of CuP fork, A2 forked opposite to CuP angle level. Hind wing smaller and narrower than forewing, narrowly oval; costal space narrow and reduced, with five or six veinlets; C and Sc fused at ¼ of wing length, Sc vein abruptly curved posteriorly at proximal margin of pterostigma to merge RA; pterostigma elongated, narrow, curved, composed of weakly defined veinlets; radial space with single crossvein, oblique; four veins arising from *rarp1*, one or two from *rarp2*. 1r-m sigmoid, connecting the stems of M and RP. Media forked slightly beyond R fork. Cubitus deeply forked, intracubital crossvein subparallel to longitudinal wing axis; CuA sinuous, first branch candelabrum-shaped, spur vein absent or present; CuP not touching A1, strongly anteriorly bent at distal 1/3, pectinate; two crossveins on cubitoanal space; A1 simple, ending on wing margin at 1r-m stem level; A2 simple, short, and curved. ***Abdomen*.** Cylindrical to medially expanded, setae on tergites, scattered, thin, and short; tergites subquadrate, tergites IV-VII with elongated anterolateral scars. Sternites rectangular, with scattered fine and short setae.

**Figure 64.**
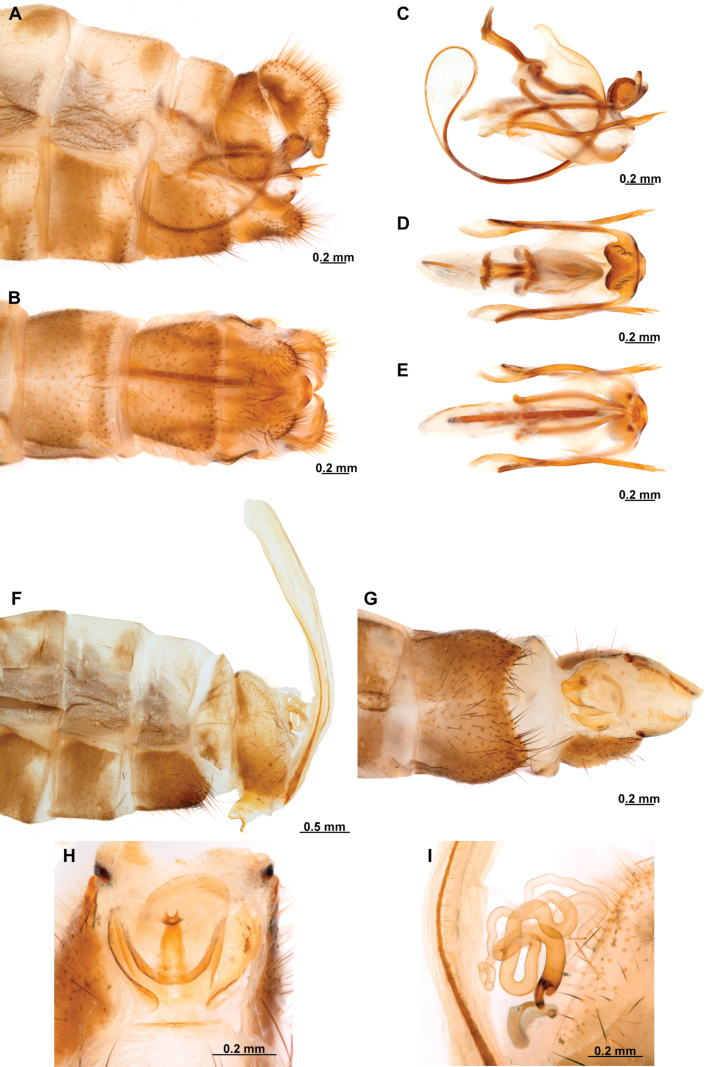
*Plegaradicaudata* Ardila-Camacho & Contreras-Ramos, sp. nov. **A** male terminalia, lateral **B** same, ventral **C** male genitalia, lateral **D** same, dorsal **E** same, ventral **F** female terminalia, lateral **G** same, ventral **H** gonapophyses VIII ventral **I** spermatheca.

***Male genitalia*** (Fig. 64A–E). Tergite IX medially narrower than laterally; lateral margin rounded with posterior tuft of setae. Sternum VIII rectangular; sternite IX subtrapezoidal in ventral view, with rounded posterolateral corners, covered with fine, short and long setae; posterior margin with medial lobe poorly developed, dorsally canaliculated; in lateral view bluntly triangular, apex approaching posterior margin of ectoproct. Gonocoxites IX thin, sinusoid, long; base, fusiform, narrow, connected to gonocoxites XI with a membrane; posterior apex thin, straight, with long and thin preapical process on inner surface, and 2–4 apical shorter, thin processes. Ectoproct ovoid, covered with thin setae, callus cerci vestigial, posteroventrally with short spines; posteroventral margin forming a rounded lobe covered with microtrichia. Gonocoxites X forming a short, thickened, ventrally canaliculate sclerite, anterior apex expanded and dorsally bent, posterior apex with dorsal processes connected to gonostyli X and ventrolateral processes connected to gonapophyses X with a membrane; gonostyli X with thickened and concave base with two lateral processes, the rest of the structure, whip-shaped, ventrally curved, and anteriorly forming single loop before protruding from abdomen. Gonapophyses X rod-shaped, narrow with posterior apex dorsally curved, arranged in a V-shaped structure; gonapophyses joined by a membrane covering the gonostyli X base; this membrane is medially sclerotized, posterolaterally adjacent to gonapophyses X apexes with a sclerite covered with microspinules. Gonocoxites XI thin, U-shaped, medial lobe complex and elaborated with two differentiated parts: a dorsal, narrow arch, and ventral concave structure that forms a ventral, caudally curved, trapezoidal process whose apex is covered with microspinules; between these parts a narrow, less sclerotized, hyaline area is present; lateral arms of gonocoxites XI long, gently curved, with anterior apex incurved. Hypandrium internum tiara-shaped.

***Female genitalia*** (Fig. 64F–I). Sternum VII trapezoidal with posterior margin concave. Tergite VIII medially strongly constrained, encircling the spiracle of the segment; lateral margin D-shaped. Gonocoxites VIII as an ellipsoid, concave sclerite. Gonapophyses VIII with medial part boat-shaped with posteromedial, dorsally curved, elongated process whose apex is bilobed; lateral part as an oval, concave plate folded beneath IX + ectoproct. Tergite IX + ectoproct D-shaped. Gonocoxites IX elongated, and narrow, as long as the last five abdominal segments together. Bursa copulatrix short, funnel-shaped, sclerotized near genital pore, the rest membranous and striated. Spermatheca entangled, not strongly elongated; proximal section, thin, proximally forming few loops, the rest zigzagged; medial section slightly wider than proximal section, forming several convolutions; distal section slightly, progressively expanded, without diverticulum; fertilization canal duct sigmoid, progressively narrowed; fertilization canal long, forming a straight angle, covered with microfilaments.

##### Distribution.

Bolivia (Santa Cruz), Peru (Madre de Dios).

##### Remarks.

This is a rather distinctive species of *Plega* known from Bolivia and Peru. It was recovered as sister to *P.bowlesi*, being part of the clade of South American species closely related to *P.hagenella*. This unique species of *Plega* has the anteroventral row of processes of the forefemur fully developed, a condition only observed in *Trichoscelia*. Its position in *Plega* is warranted because it has the basal primary process of the anteroventral row of processes, two primary processes on the posteroventral row of processes on the proximal ½ of the femur and the ventrally fused and collar-like postfurcasterna. Additionally, this species has a genitalic morphology noticeably similar to that of *P.hagenella* yet can be differentiated by the shape of the apex of the male gonocoxites IX which resembles plant roots and the gonostyli X which is simply recurved. This species, together with *P.insolita*, express a series of plesiomorphisms, making them important species in understanding the evolution of the genus and the Symphrasinae.

#### ﻿﻿ Plega signata

Taxon classificationAnimaliaNeuropteraRhachiberothidae

(Hagen, 1877)

Figs 65 , 66

Symphrasis signata Hagen, 1877: 208. Holotype: female, USA, California (MCZ), specimen examined.
Plega signata (Hagen, 1877). Navás (1928): 326.

##### Material examined.

***Holotype*.** USA • ♀; **California**, Kern, Fort Tejon; 1875; Brown leg.; “10429, no. 810”; “*Symphrasissignata* Hagen”; MCZ.

##### Other material.

Mexico – **Baja California** • 2 ♀; Mexicali, Cañón de Guadalupe; 32°09'11"N, 115°47'25"W; 355 m; 20 May. 2009; nocturnal collection, “palmar”; CNIN. • 1 ♀; Sierra Calamajué, 11 km e Chapala; 29°31'N, 114°42'W; 23 Aug. 1994; S.L. Heydon; UCD. • 1 ♂; 2 mi. SW San Francisquito, box canyon with caves; 26–27 Jul. 1986; R.L. Aalbu leg.; BL; CSCA. • 1 ♀; Ensenada, Carr. 1, Hotel Misión Cataviñá, predio detrás; 29°43.266'N, 114°43.194'W; 558 m; 07 Aug. 2021; Contreras, Cancino, Luna, Martins & Marquez leg.; light trap; CNIN. – **Baja California Sur** • 2 ♂; 0.5 mi N. of Miraflores; 06 Jan. 1959; H.B. Leech leg.; FSCA. • 1 ♂; 6 mi. S. of Miraflores; 18 Jan. 1959; H.B. Leach leg.; CAS. • 2 ♂ 4 ♀; 1.8 Km E. La Burrera, 23110Bc, (CapeThorFor.); 490 m; 03 Jun. 1973; E.L. Sleeper leg.; collected at blacklite; *Plegafratercula*, det. N. Penny, 1998; CAS. • 1 ♂ 1 ♀; Sierra Lagunas, 1.7 mi. E. Summit, Los Naranjos rd.; 23°13'4"N, 109°50'8"W; 500 m; 15–16 Jul. 1999; R. Aalbu, K. Brown, I. Stahl & F. Piñero leg.; B. Lite; #D00750-502; CAS. • 1 ♂ 2 ♀; 3 mi. N. San Antonio; 9–10 Oct. 1968; El. 1200’; E.L. Sleeper & F.J. Moore leg.; collected at blacklite; *Plegafratercula*, det. Penny, 1998; CAS. • 1 ♀; same data as for preceding; *Plegadactylota*, det. N. Penny, 1998 (CAS). • 1 ♂; 22 mi. W. la Paz; 25 Jun. 1967; E.L. Sleeper & E.M. Fisher leg.; collected at blacklite; *Plegadactylota*, det. N. Penny, 1998; CAS. • 2 ♂ 2 ♀; 8 mi. SE. La Paz; El. 1000’; 13 Oct. 1968; E.L. Sleeper & F.J. Moore leg.; collected at blacklite; *Plegadactylota*, det. N.D. Penny, 1998; CAS. 22 mi. • 1 ♂; W. La Paz; 25 Jun. 1967; E.L. Sleeper & E.M. Fisher leg.; collected at blacklite; *Plegasignata*, det. N. Penny, 1998; CAS. • 1 ♂; 4 Km NE. San Lucas del Cabo; 15 m; 11–12 Jun. 1973; E.L. Sleeper leg.; BL; 22109Aa, (CpThrn.); *Plegadactylota*, det. N. Penny, 1998; CAS. • 1 ♂; 2 mi. SW. S. José del Cabo; 03 Jul. 1967; E.M. Fisher leg.; *Plegadactylota*, det. N.D. Penny, 1996; CAS. • 1 ♂; Valle Perdido, 2 mi. N. Reserva gate; 23°21'49"N, 110°1'12"W; 500 m; 16–17 Jul. 1999; R. Aalbu, K. Brown, I. Stahl & F. Piñero leg.; #D00750-1502; *Plegadactylota*, det. N. Penny, 1999; CAS. • 1 ♂; 3.8 Km SE Valle Perdido; 08 Jun. 1973; 23°40'44.4"N, 110°5'31.2"W; E.L. Sleeper leg.; CSCA. • 1 ♀; 28–29. Km N. Todos los Santos (Km 23–24); 250 m; 26 Aug. 1977; E. Fisher & R. Westcott leg.; collected at black light; CAS. • 1 ♀; Baja California Sur, 1.3 mi. N. San José Comondú; 21 Jun. 1967; E.L. Sleeper & E.M. Fisher leg.; *Plegadactylota*, det. N. Penny, 1998; CAS. • 1 ♀; 5 Km S. Último Agua; 25°33.65'N, 111°16.07'W; 288 m; 14 Aug. 2004; Tomas, Burne, Robacker leg.; TAMU-ENTOX0756149; TAMUIC. • 1 ♂; Isla Cerralvo; 04 Aug. 1986; F. Arias leg.; CNIN. • 1 ♂ Santa Rita, 13 Km S., 15 Km W.; 24°28'42.4272"N, 111°36'48.7332"W; 2 m; 04 Sep. 2013; E. Ríos leg.; CNIN. • 1 ♀; El Pescadero, Playa Los Cerritos; 16 Apr. 1979; M. Wasbauer leg.; cerial bowl pit trap; CSCA. • 22 ♂ 32 ♀; Loreto, Misión de San Fco. Javier de Viggé-Biaundó, Arroyo San Javier; 25°51.347'N, 111°33.005'W; 393 m; 11 Aug. 2021; Luna, Contreras, Barba & Ramírez leg.; light trap; CNIN.

USA – **Arizona** • 1 ♂; 18 Sep. 2006; C. Porter & L. Stange leg.; FSCA. • 1 ♀; Phoenix; 06 May. 1962; G. Deal leg.; FSCA. • 2 ♀; Pima C., Alamo Canyon, Ajo Mountains; Jul. 1924; H.B. Leech & J.W. Green leg.; *Plegabanksi*, det. N. Penny, 1985; CAS. • 1 ♂; Oslar, Prescott; 01 Feb.; N. Banks leg.; Identified as *Symphrasissignata*; det. Rehn, 1939; MCZ. – **California** • 1 ♀; Fresno Co., Piedra; 06 Aug. 1982; R.F. Gill leg.; “Donald J. Burdick Collection, Donated to CAS”; CAS. • 1 ♂; JTNM Plsnt. Vlly Fried Liver Wsh; 30 Jun. 1965; E.L. Sleeper & S.L. Jenkins leg.; blacklite; CASENT8140616; *Plegasignata*, det. N. Penny, 1998; CAS. • 1 ♂; Joshua Tree N.M., Pinyon Wells 5; 25 Sep. 1965; E.L. Sleeper & S.L. Jenkins leg.; CASENT8140589; *Plegasignata*, det. N. Penny, 1998; CAS. • 1 ♂; Joshua Tree N.M. Pinyon Wells 2; 28 Jul. 1967; E.L. Sleeper & S.L. Jenkins leg.; CASENT8140674; *Plegasignata*, det. N. Penny, 1998; CAS. • 1 ♀; Joshua Tree N.M., Pinyon Wells; 15 May. 1969; E.L. Sleeper leg.; collected at blacklite; CASENT8140579; *Plegasignata*, det. N. Penny, 1998; CAS. • 1 ♀; Joshua Tree NM, Pleasant Vlly. 1; 16 Jul. 1965; E.L. Sleeper & S.L. Jenkins leg.; Blacklite; CASENT8140576; *Plegasignata*, det. N. Penny, 1998; CAS). • 1 ♀; same data as for preceding; Pleasant Vlly. 3; 27 Aug. 1965; CASENT8140548; CAS. • 1 ♀; same data as for preceding; CASENT8140546; CAS. • 1 ♀; Joshua Tree N.M.L. Covington Flat; R.E. Somerby leg.; CASENT8140596; *Plegasignata*, det. N. Penny, 1998; CAS. • 1 ♂; same data as for preceding; CASENT8140598; CAS. • 1 ♂; same data as for preceding; CASENT8140632; CAS. • 1 ♂; same data as for preceding; CASENT8140928; CAS. • 1 ♀; same data as for preceding; CASENT8140931; CAS. • 1 ♀; Joshua Tree N.M., Squaw Tank; 16 Jun. 1960; J. Geest & W. Schilling leg.; CASENT8140553; *Plegasignata*, det. R.G. Beard; CAS. • 1 ♀; Joshua Tree N.M., Squaw Tank; 06 Aug. 1959; E.L. Sleeper leg.; CASENT8140874; *Plegasignata*, det. N. Penny, 1998; CAS. • 1 ♂; Joshua Tree N. M., Smithwater Wash; R.E. Somerby leg.; CASENT8140930; *Plegasignata*, det. N. Penny, 1998; CAS. • 1 ♂; Riverside Co., Phillip L Boyd Deep Canyon Desert Research Ctr at Marker 0; 33°30.0'N, 116°23.5'W; 380 m; 2–3 Jun. 2002; M.E. Irwin, F.D. Parker leg.; malaise nr water in canyon wash; CAS. • 1 ♂; Joshua Tree N. M., L. Covington Flat; E.L. Sleeper leg.; MCZ-ENT00681786; *Plegasignata* (Hagen), det. R.G. Beard; MCZ. • 1 ♀; Joshua Tree N. M., L. Covington Flat; M. Knox & E. Sleeper leg.; MCZ-ENT00681787; *Plegasignata* (Hagen), det. R.G. Beard; MCZ. • 1 ♀; Joshua Tree, Bells Cmp., N.M.; 04 Aug. 1959; F.C. Raney; UCD. • 1 ♀; San Diego County, 6 Km E. Jacumba, nr. Desert View Tower; 32°39.1'N, 116°06.1'W, 30 May.–05 Jun. 2002; M.E. Irwin & F.D. Parker leg.; Malaise in small wash; CAS. • 16 ♂ 5♀; San Diego Co., Tecate lookout station; 21 Aug. 1963; B. Reed leg.; CSCA. • 1 ♂; S.D. Co., San Isidro; 24 Jun. 1969; ex light trap, plant quarantine, Riv. USDA; CSCA. • 1 ♂; Mendocino County, 7.5 Km NE. of Covelo; 39°50'N, 123°10.5'W; 21 Aug. 2012; J. Vindum leg.; CASENT8230400; CAS. • 1 ♀; San Bernardino Co., 29 Palms Marine Base, Powerline Road; 31 May. 2003; G. Pratt & C. Pierce leg.; night collect; TAMU-ENTOX0280778; *Plegasignata* (Hagen, 1877), det. J.D. Oswald, 2004; TAMUIC. • 1 ♂; same data as for preceding; TAMU-ENTOX280779; TAMUIC. • 1 ♀; San Bernardino Co., Gold Valley, 1.3 mi. S. Mid Hills Campg.; 08 May–11 Aug. 1981; R.L Aalbu leg.; Antifreeze pit trap; CSCA. • 1 ♂ 1 ♀; San Bernardino, Co. 6 mi. SE Hesperia; 11 Jul. 1981; A.R. Hardy leg.; at blacklight; CSCA. • 1 ♀; Inyo Co., China Lake Naval Weapons Ctr.; 36°05.16'N, 117°29.66'W; 17 Jul–16 Aug. 2014; M.E. Irwin Pratt leg.; Malaise at Old House Spr.; CSCA. • 1 ♀; Inyo co., China Lake, Mt. Spring Cyn.; 35°56.91'N, 117°33.40'W; 1220 m; 17 Sep. 2014; M.E. Irwin leg.; Malaise nr creek with *Salix*; CSCA. • 1 ♀; Inyo Co., Saline Valley, Grapevine Canyon; 5000’; 25 Jan. 1986–29 Nov. 1986; D. Giuliani leg.; Antifreeze pit trap; CSCA. • 1 ♂; Anza Riv. Co.; 15 Aug. 1966; R. Gill leg.; black light; CSCA. • 1 ♂; Tulare Co., Yisalia; 24 Aug. 1960; A. Blant; CSCA. • 1 ♂; Inyo Co., Inyo Mts., Long John Cyn.; 5500’; 14 Jul–26 Dec. 1982; D. Giuliani leg.; antifreeze pit trap; CSCA. • 1 ♂; L.A. Co., Glendale; 09 Aug. 1952; W.M. Schlinger leg.; UCD. • 1 ♂; same data as for preceding; IX.1952; UCD. • 1 ♂; same data as for preceding; 09 Aug. 1952; UCD. • 1 ♂; Glendale; 20 Aug. 1949; E.I. Schlinger leg.; UCD. • 1 ♂; L.A. Co., Tambark Flat; 20 Aug. 1950; E.B. Goodwin; UCD. • 1 ♂; Bakersfield Cal.; 25 Jul. 1951; L.W. Isaak leg.; UCD. • 1 ♂; Kern Co., Keene; 23 Jul. 1951; L.W. Isaak leg.; UCD. • 1 ♂; Kern Co., Woody Cal.; 15 Jul. 1951; LW Isaak leg.; UCD. • 1 ♂; Solano Co., Cold Canyon Rerv., 11. Km W Winters; 28 Jul. 1992; S.L. Heydon leg.; UV; UCD. • 1 ♂; Yolo Co., 8 mi. NW Winters Cal.; 18 Aug. 1959; J. Fowler leg.; light trap; UCD. • 1 ♂; Tulare Co., Hot Springs; 13 Aug. 1966; R.E. Stecker leg.; black light; UCD. • 1 ♂; Calaveras Co., Gwin Mine Rd., 1 mi. N., Paloma Rd; 16 Jul. 2009; R.M. & S. Brown leg.; UCD. • 1 ♀; Orange Co., Santiage Cyn; 27 Aug. 1962; N.W. Fleming leg.; UCD. – **Nevada** • 1 ♀; Clark Co., Aravada Ranch, 7.3 Km E. Whitney Pocket on AZ Rd.; 36°31.35'N, 114°02.60'W; 1317 m; 06 May–08 Jun. 2018; M.E. Irwin, G.R. Ballmer leg.; Malaise in narrow rocky gully; CSCA.

##### Diagnosis.

This species has the antennal flagellum generally pale brown, with proximal flagellomeres two times as long as wide. The forefemur posterior surface is pale with small to widened brown areas near the middle; the anterior surface is dark brown. The wing venation on both wings has a conspicuous alternating color pattern of brown and pale. On the male genitalia, the gonocoxite IX is thin, long, and straight, with the posterior apex set with 3–5 elongated, intertwined processes. The ventral part of gonocoxites XI has a prominent curved, rugose process covered with granules. On the female genitalia, the gonapophyses VIII medial part is keel-shaped, lacking posteromedial process. The spermatheca is long and entangled, with proximal section thin, forming numerous coils; the medial section forms irregular convolutions and terminates in a long spiral; the distal section terminates in an abruptly expanded portion, where a blunt diverticulum is present.

##### Description.

***Measurements*.** Male (*n* = 8). Forewing length: 7.6–16.5 mm; Hind wing length: 6.0–12.6 mm. Female (*n* = 10): Forewing length: 6.6–16.5 mm; Hind wing length: 5.3–12.8 mm.

**Figure 65.**
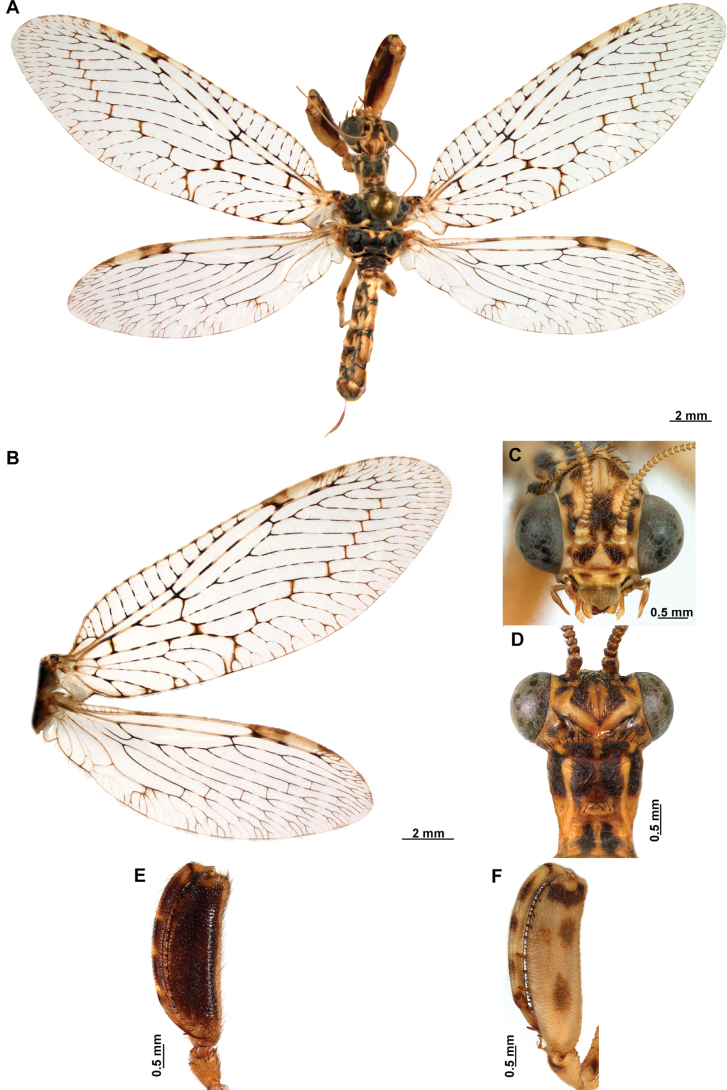
*Plegasignata* (Hagen, 1877) **A** female habitus, dorsal **B** wings **C** head, frontal **D** pronotum, dorsal **E** forefemur, anterior surface **F** same, posterior surface.

***Coloration*** (Fig. 65). ***Head*.** Vertexal pale with lateral brown stripes extending from occiput to supra-antennal area, an encircling a pale area, near paraocular region; these marks with pale brown setae; area adjacent to occipital ridge pale, area adjacent to coronal suture pale; supra-antennal area dark brown; pale brown setae present; occiput pale with dark brown mark, postgena pale, sometimes with diffuse brown area. Antennal scape pale ventrally and brown dorsally, pedicel brown; flagellum generally pale brown. Frons generally with semi-triangular, brown marks beneath toruli and pale, triangular area on middle. Clypeus pale with lateral brown areas; labrum pale to pale brown; mandible pale dark brown corners, dark amber at apex; maxilla pale with pale brown galea, palpus brown, with pale brown setae; labium pale with pale brown ligula, palpus brown, setae pale to pale brown; palpimacula pale brown. ***Thorax*.** Pronotum pale with two pairs or dark brown marks on anterior 2/3, the medial ones connected on the middle of pronotum, with interspersed pale and dark brown setae; episternum pale to pale brown with ventral margin dark brown, with interspersed pale and brown setae; postfurcasternum pale. Mesonotum mostly dark brown, scutum with pale areas anterolaterally, posteromedially, and on area adjacent area to ecdysial suture, scutellum pale lateral areas; interspersed pale and dark brown setae present. Metanotum dark brown, with pale anteromedian area with dark brown mark con the center; pre-episternum brown with medial pale stripe; pteropleura dark brown with pale areas, setation mostly pale brown. ***Foreleg*.** Coxa pale brown with dark, apical areas or nearly completely brown, with interspersed pale brown setae and dark brown setae; trochanter pale, sometimes with brown marks, setation mostly pale brown, except dorsally with some dark brown setae. Femur posterior surface pale with small to widened brown areas near the middle, a dark mark near the apex, and sometimes with brown areas near distal portion of closing surface and base; anterior surface dark brown, except small pale area on the base of closing surface. Tibia posterior surface alternating pale and dark brown areas, sometimes anterior surface mostly dark brown. Basitarsus with base and lanceolate process amber, and a sub-basal pale area, clavate setae pale to pale brown; remaining tarsomeres pale to pale brown. ***Mid- and hind leg*.** Coxae pale with brown areas, with pale or pale brown setae; trochanter pale. Femora and tibiae pale with brown areas, with interspersed pale and dark brown setae. Tarsi pale brown; first four tarsomeres with dark brown setae, laterally on distal margin of plantar surface; remainder surface with setae mostly pale brown; pretarsal claws pale brown. ***Wings*.** Forewing mostly hyaline; membrane surrounding crossveins, forks of longitudinal veins, first branch of CuA and apex of CuP, with subtle or strong amber maculation. Pterostigma either brown with pale medial area, or pale with brown areas. Major veins, subcostal veinlets and wing margin alternating pale and dark brown; crossveins dark brown. Hind wing hyaline, amber on area adjacent to stem first branch of CuA, and intermittently on apical branches of longitudinal veins adjacent to posterior margin; pterostigma brown with pale preapical area; longitudinal veins alternating pale and dark brown areas; subcostal veinlets nearly transparent; crossveins brown, 1r-m often bicolor; wing margin alternating pale and brown. ***Abdomen*.** Tergites mostly pale brown, with pale brown setae. Pleural membrane brown with pale areas. Sternites mostly pale brown.

***Morphology*** (Fig. 65). ***Head*.** Diamond-shaped in frontal view, rugose; vertexal region raised above compound eyes, with lateral rows of reclined setae; area surrounding coronal suture glabrous, with muscle insertion mark; coronal suture distinct; medial area of ​​supra-antennal region not raised, with fine, reclined setae; paraocular area concave. Antenna submoniliform, slightly longer than head and prothorax together; scape slightly longer than wide, cup-shaped, with fine, short setae; pedicel as long as wide; flagellum dorsoventrally flattened, with 45–58 flagellomeres, those of proximal ¼ of the flagellum two times as wide as long, then changing from as long as wide to slightly longer than wide; all articles with medial ring of fine, short setae. Compound eye hemispherical, as wide as ½ of the interocular distance at torulus level. Frons and clypeus narrow, with fine and short setae. Labrum pentagonal with thin, short setae; maxillary palpus with first palpomere as long as wide, second 1.2× as long as wide, third palpomere 3× as long as wide, fourth palpomere 2.2× as long as wide, fifth palpomere slightly longer than third, all with short and thin setae; mentum with long, thin setae; labial palpus with first palpomere two times as long as wide, second palpomere 4× as long as wide, third palpomere slightly longer than second, palpimacula narrowly ovoid. ***Thorax*.** Pronotum as long as wide, with raised anterior margin, medial and posterior regions; outgrowths covered with pedicellate, thick setae; remaining surface with fine, short setae. Mesonotum slightly wider than long, with abundant, thick, pedicellate setae on medial area. Metanotum ~ 2× as wide as long, scutellum with few, fine setae. Pteropleura with long, thin setae, some thicker on mesanepisternum. ***Foreleg*.** Coxa as long as femur, cylindrical, anterior and posterior surfaces with pedicellate fine or thick setae of different lengths; trochanter trapezoidal, with thin and short setae, except on dorsal surface with some thickened, pedicellate setae near distal margin; anterior surface with protuberant area covered with pedicellate thickened setae. Femur robust, finely rugose, covered with abundant, fine, short setae; closing surface with posteroventral row of processes composed two medially located, primary processes, sometimes with a tertiary process at the middle; proximal portion of the row with secondary or tertiary basal process and sub-basal secondary process; the rest of the row with abundant tubercle-shaped processes and stinger-shaped setae; distal portion raised composed of tubercle-shaped specializations and stinger-shaped setae; adjacent row of thickened setae with globular base present on distal ¼. Anteroventral row of processes reduced to proximal ½; it is composed of tubercle-shaped specializations and stinger-shaped setae; the basal-most primary, curved process is present; distal portion composed of a few tubercle-shaped processes; adjacent row of thickened setae with globular base present on distal ⅘. Tibia almost as long as femur, curved, with thin, short setae; ventral surface keeled with prostrate setae; a patch of clavate setae apically on anterior surface is present. Basitarsus with lanceolate process reaching the middle of fourth tarsomere; clavate setae present proximally on anterior surface; ventrally with single row of prostrate setae; second tarsomere nearly 7× as long as wide; third tarsomere as long as wide, fourth tarsomere two times as long as wide. ***Mid- and hind leg*.** Coxae and trochanter with thin setae, which are shorter on trochanter; femora with interspersed fine setae of different lengths, and with a few thickened setae; tibiae mostly with short fine setae; tibial spurs short; hind leg longer than midleg, tibia 1.5× as long as femur; tarsi with fine and short setae, except on distal margin of plantar surface with lateral groups of 3–9 thickened setae; on both legs, basitarsus 4× as long as wide, second tarsomere 1.5× as long as wide; third and fourth tarsomeres as long as wide; fifth tarsomere 2.5× as long as wide. ***Wings*.** Forewing narrowly oval, trichosors present along margin except on wing base; venation setose; costal space proximally expanded, humeral vein branched, 13–15 subcostal veinlets; pterostigma elongated, narrow, straight, with incomplete veinlets; subcostal space with single crossvein, medially located; Sc vein abruptly posteriorly bent at proximal pterostigma margin to merge with RA; radial space with two crossveins; *rarp2* gently curved with three or four RP branches; three or four veins arising from *rarp1*; M vein basally fused with RA; RP base, widely separated from divergence of M and R; M forked opposite to slightly before to RP origin, 1 r-m connecting RP base and M fork, forming a trapezoidal cell; 3–5 gradate crossveins present. Cubitus deeply forked; CuP basally angled and approaching A1, distally forked to separation of M and R; A1 apically forked, ending on posterior margin at level of CuP fork, A2 forked opposite to CuP angle level. Hind wing smaller and narrower than forewing, narrowly oval; costal space narrow and reduced, with 6–8 veinlets; C and Sc fused at ¼ of wing length, Sc vein abruptly curved posteriad at proximal margin of pterostigma to merge RA; pterostigma elongated, narrow, gently curved, composed of incomplete veinlets; radial space with single crossvein, oblique; three veins arising from *rarp1*, 0–2 from *rarp2*. 1r-m sigmoid, connecting the stems of M and RP. Media forked beyond R fork. Cubitus deeply forked, intracubital crossvein subparallel to longitudinal wing axis; CuA sinuous, first branch candelabrum-shaped, spur vein absent or present; CuP not touching A1, strongly anteriorly bent at distal ½, pectinate, sometimes fused to first branch of CuA; two crossveins on cubitoanal space; A1 simple, ending on wing margin at 1r-m stem level; A2 simple, short, and curved. ***Abdomen*.** Cylindrical, setae on tergites, scattered, thin, and short; tergites subquadrate, tergites III and IV with posteromedian concavity, tergites IV-VI with elongated, anterolateral scars. Sternites rectangular, with abundant fine and short setae, longer towards terminal segments.

**Figure 66.**
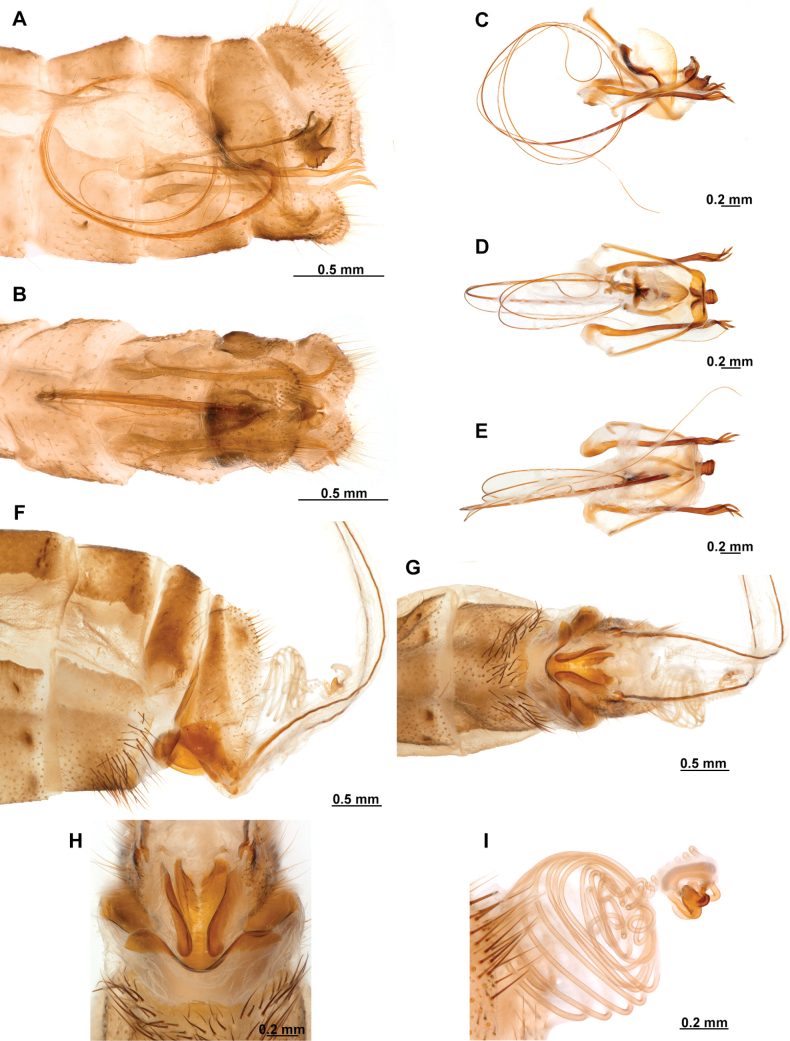
*Plegasignata* (Hagen, 1877) **A** male terminalia, lateral **B** same, ventral **C** male genitalia, lateral **D** same, dorsal **E** same, ventral **F** female terminalia, lateral **G** same, ventral **H** gonapophyses VIII ventral **I** spermatheca.

***Male genitalia*** (Fig. 66A–E). Tergite IX medially narrower than laterally; lateral margin bluntly trapezoidal. Sternum VIII rectangular; sternite IX pentagonal in ventral view, posterolaterally rounded and convex, with abundant, thin, long setae; posterior margin with medial, short lobe which is dorsally canaliculated; in lateral view blunt, apex nearly reaching posterior margin of ectoproct. Gonocoxites IX thin, long, straight; base flattened, spatulated, dorsally arched; posterior with 3–5 elongated, intertwined processes. Ectoproct ovoid, covered with abundant, thin, long setae on posterior surface, posteroventrally with blunt lobe, covered with fine, shirt, pedicellate setae; anteroventrally with flattened, rounded lobe that is continuous with ventromedial, sclerotized concave area. Gonocoxites X forming an elongated, straight, ventrally canaliculated sclerite, whose anterior apex is slightly expanded; posterior apex has dorsal processes connected to gonostyli X and ventrolateral processes connected to gonapophyses X with a membrane; gonostyli X with thickened and arched base, equipped two lateral processes; the rest of the structure, whip-shaped, ventrally curved, and anteriorly coiled, forming two loops before protruding from abdomen. Gonapophyses X long, straight, narrow, with posterior apex slightly expanded; gonapophyses arranged in a V-shaped structure, joined by a membrane covering the gonostyli X base. Gonocoxites XI thin, U-shaped, medial lobe complex and elaborated, with two differentiated parts: dorsal part as narrow arch; ventral part with medial are covered with granules and lateral concavities and with prominent ventral, curved, rugose process; between these parts a narrow, arched or rectangular, less sclerotized, hyaline area is present; lateral arms of gonocoxites XI straight, with anterior apex straightly angled. Hypandrium tiara-shaped.

***Female genitalia*** (Fig. 66F–I). Sternum VII rectangular with broad posteromedian concavity; with long, thickened setae near such concavity. Tergite VIII slightly narrower medially than laterally, encircling the spiracle of the segment; lateral region narrow, margin rounded. Gonocoxites VIII with medial concave area, laterally continuous with narrow, flat projections; posteromedially connected to gonapophyses VIII. Gonapophyses VIII with medial part keel-shaped, without posteromedial process; laterally folded beneath IX + ectoproct, a broadly oval plate is connected to medial part of gonapophyses VIII. Tergite IX + ectoproct D-shaped. Gonocoxites IX remarkably elongated, sinuous, and narrow, as long as the last seven segments together. Bursa copulatrix long with proximal part slightly sclerotized, the rest membranous and striated. Spermatheca long, complex, and entangled; proximal section, thin, long, forming numerous coils; medial section as wide as proximal section, forming numerous irregular convolutions and terminating in a long spiral; distal section entangled, as wide as proximal section, terminating in an abruptly expanded portion, where a blunt diverticulum is present; fertilization canal duct with proximal section triangular and concave; fertilization canal elongated, pod-shaped, covered with microfilaments.

##### Distribution.

Mexico (Baja California, Baja California Sur), USA (Arizona, California, Nevada).

##### Remarks.

This species is distributed from Baja California Sur in Mexico to California, Nevada, and Arizona in the United States (Rehn 1939b; Penny et al. 1997; Oswald et al. 2002; Reynoso-Velasco and Contreras-Ramos 2008). The records of this species from Nevada provided herein represent the first ones from that state. It was also previously reported from Costa Rica (Reynoso-Velasco and Contreras-Ramos 2008), but this record is erroneous. *Plegasignata* was recovered as sister to *P.flammata*, and both species may be difficult to differentiate, particularly in the Peninsula of Baja California where both overlap their distribution ranges. *Plegasignata* can be differentiated from *P.flammata* because the male gonocoxite IX has only 3–5 intertwined digitiform processes and the anterior surface of the forefemur is completely brown.

#### ﻿﻿ Plega sonorae

Taxon classificationAnimaliaNeuropteraRhachiberothidae

Ardila-Camacho et al., 2019

Figs 67 , 68

Plega sonorae Ardila et al., 2019: 357. Holotype: male, Mexico, Sonora (CNIN), specimen examined.

##### Material examined.

***Holotype*.** Mexico • ♂; **Sonora**, Rosario de Tesopaco 11 Km E carretera a la Estrella; 27°49'57"N, 109°15'10"W; 482 m; 03 May. 2005; L. Cervantes, M Schwartz leg.; CNIN. ***Paratypes*.** Mexico – **Sonora** • 1 ♂; Rosario de Tesopaco, 11 Km E carretera a la Estrella; 482 m; 27°49'57"N, 109°15'10"W; 03 May. 2005; L. Cervantes & M. Schwartz leg.; CNIN. • 1 ♂; 36.6 Km SE Tecoripa, La Barranca; 28°34'40.1"N, 109°39'54.3"W; 562 m; 19 Jul. 2004; S. Zaragoza, F. Noguera, E. Ramírez, E. González leg.; bosque tropical caducifolio, light trap 1, MDM0227, 2723; CNIN. • 1 ♂; same data as for preceding; 645 m; MDM0276, 2700; CNIN. • 1 ♂; 24.4 Km SE de Tecoripa, Rancho Lo de Campa, 2 Km S. El Cajón; 28°32'13.2"N, 109°44'37.7"W; 483 m; 18 Jul. 2004; S. Zaragoza, F. Noguera, E. Ramírez, E. González leg.; bosque tropical caducifolio, light trap, MDM0253; CNIN. • 1 ♂; same data as for preceding; MDM0252; CNIN. • 1 ♂; 29 Km SE Tecoripa and 3 Km SW Rancho Las Peñitas; 28°32'21.7"N, 109°41'31.5"W; 645 m; 22 Apr. 2004; S. Zaragoza, F. Noguera, E. González, E. Ramírez leg.; light trap 2; MDM0254, 2705; CNIN.

##### Other material.

Mexico – **Colima** • 1 ♀; 5.8 Km NW Ixtlahuacan; 19°01'15.7"N, 103°46'37.8"W; 345 m; 25 Jun. 2006; S. Zaragoza, F. Noguera, E. González, E. Ramírez leg.; bosque tropical caducifolio, light trap; CNIN. – **Sinaloa** • 1 ♂; 16 miles SO. Guamuchil; 16 Jun. 1961; F.D. Parker leg.; FSCA. – **Sonora** • 1 ♀; 36.6 Km SE Tecoripa, La Barranca; 28°34'40.1"N, 109°39'54.3"W; 645 m; 16 Aug. 2004; S. Zaragoza, F. Noguera, E. Ramírez, E. González leg.; bosque tropical caducifolio, light trap 1, MDM0299; CNIN. • 1 ♀; same data as for preceding; 562 m; 19 Jul. 2004; MDM0229, 2696; CNIN. • 1 ♀; same data as for preceding; MDM0298; CNIN. • 1 ♀; same data as for preceding; MDM0268; CNIN. • 1 ♀; same data as for preceding; MDM0; CNIN. • 1 ♀; same data as for preceding; 19 Jul. 2004; MDM0230; CNIN. • 1 ♀; same data as for preceding; 19 Jul. 2004; MDM0228; CNIN. • 1 ♀; 36.6 Km SE Tecoripa, La Barranca; 28°34'40.1"N, 109°39'54.3"W; 562 m; 20 Apr. 2004; S. Zaragoza leg.; light trap; CNIN. • 1 ♀; 28.5 Km SE Tecoripa, Cerro Verde; 28°33'09.5"N, 109°43'34"W; 532 m; 19 Apr. 2004; S. Zaragoza leg.; light trap; CNIN. • 1 ♂ 2 ♀; Rosario de Tesopaco, 11 Km E carretera a la Estrella; 482 m; 27°49'57"N, 109°15'10"W; 03 May. 2005; L. Cervantes & M. Schwartz leg.; CNIN. • 2 ♂ 1 ♀; 40 mi N Guaymas; 29 Aug. 1959; L.A. Stange & A.S. Menke leg.; FSCA. • 1 ♀; 2 Km S. San Javier; 28°34'53"N, 109°44'51.5"W; 795 m; 18 Jul. 2004; S. Zaragoza, F. Noguera, E. Ramírez, E. González leg.; bosque tropical caducifolio, light trap; MDM0225; CNIN. • 1 ♀; same data as for preceding; MDM0226; CNIN. • 1 ♂; unknown collecting data; FSCA.

USA – **Arizona** • 5 ♂ 1 ♀; Pima Co., Brown Cyn., Baboquivari Mts.; 08 Jun. 1952; M. Cazier, E. Gertsch, R. Schrammel leg.; FSCA.

##### Diagnosis.

This species presents a pale, arrow-shaped mark on the area adjacent to coronal suture with, like in *P.megaptera*. The antennal flagellum is brown, with proximal flagellomeres two times as wide as long. The pronotum is as long as wide. The forefemur posterior surface is pale brown with widened dark brown areas which are interconnected; the anterior surface is dark brown. The pterostigma is dark brown with irregular, pale, medial area. On the male genitalia, the gonocoxite IX is short, thickened, and sinusoid; the posterior apex is laterally curved, and set with two or three processes, of which one is apical and longer and one or two are preapical and shorter. The ventral part of the gonocoxites XI median lobe has a convex area covered with microspinules, continuous with ventral, caudally curved process, whose apex is bilobed. On the female genitalia, the gonapophyses VIII medial part is trapezoidal and keeled, produced posteromedially, forming a Y-shaped process which is caudally bent. The bursa copulatrix is strongly sclerotized at the proximal part. The spermatheca has the proximal section, thin, forming numerous coils; the medial section forms a long spiral, while the distal section terminates in an expanded portion, where a blunt diverticulum is present.

##### Description.

***Measurements*.** Male (*n* = 7). Forewing length: 10.0–17.6 mm; Hind wing length: 7.8–13.8 mm.

**Figure 67.**
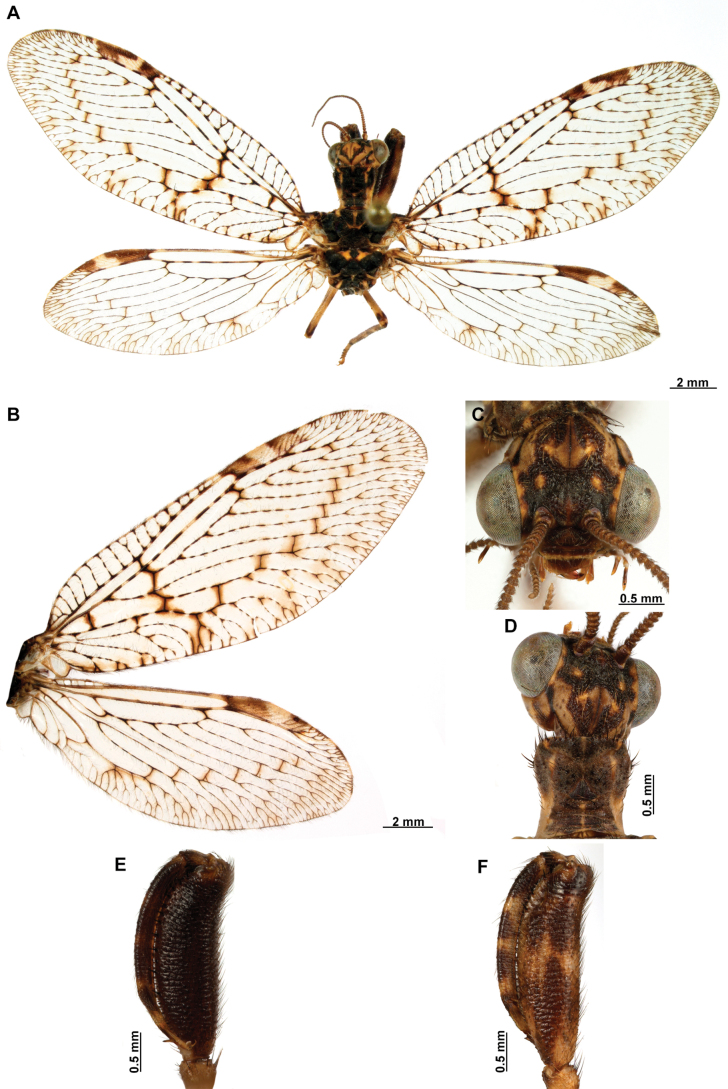
*Plegasonorae* Ardila et al., 2019 **A** male habitus, dorsal (abdomen removed) **B** wings **C** head, frontal **D** pronotum, dorsal **E** forefemur, anterior surface **F** same, posterior surface.

***Coloration*** (Fig. 67). ***Head*.** Vertexal pale brown with dark brown marks extending from occiput to supra-antennal area, anterolaterally encircling two lateral, small pale areas; area adjacent to occipital ridge dark brown, area adjacent to coronal suture with paler, arrow-shaped mark; supra-antennal area dark brown; brown setae present; occiput pale with dark brown mark, postgena pale with brown area. Antennal scape brown with pale, dorsal, and ventral areas, pedicel brown; flagellum brown with darker setae. Frons either dark brown or with lateral dark brown triangles and medial paler triangle. Clypeus pale brown with lateral darker marks; labrum pale to dark brown; mandible pale with dark brown corners, amber at apex; maxilla pale with brown areas on stipes and galea, palpus brown with paler junctions; labium including palpus brown; palpimacula pale brown. ***Thorax*.** Pronotum dark brown with small pale areas; episternum dark brown, sometimes with paler area; postfurcasternum pale. Mesonotum dark brown, sometimes with pale areas adjacent to sutures. Metanotum dark brown, with pale anteromedian area; pre-episternum dark; pteropleura dark brown with pale areas. ***Foreleg*.** Coxa brown, sometimes with paler and darker areas, interspersed pale, and dark brown setae present, darker ones with brown base; trochanter brown with paler and darker areas, setation mostly pale brown, except dorsally with some dark brown setae. Femur posterior surface pale brown with widened dark brown areas which are connected; anterior surface dark brown. Tibia posterior surface alternating dark and pale areas, anterior surface mostly dark brown; with pale brown setae. Basitarsus with base and lanceolate process dark amber, and pale brown sub-basal area, clavate setae pale brown; remaining tarsomeres pale brown. ***Mid- and hind leg*.** Coxae dark brown, sometimes with pale areas, with interspersed pale and dark brown setae; trochanter pale with pale brown suffusions. Femora and tibiae pale with brown rings, mainly with dark brown setae; tibial spurs brown. Tarsi pale, except fifth tarsomere pale brown; first four tarsomeres with dark brown setae, laterally on distal margin of plantar surface; remainder surface with pale brown setae; pretarsal claws brown. ***Wings*.** Forewing mostly hyaline; membrane with extensive maculation on area surrounding crossveins and forks of longitudinal veins dark amber; area adjacent to first branch of CuA and apex of CuP strongly marked, from this point to wing apex intermittent dark amber areas between apical forks of longitudinal veins are present. Pterostigma dark brown with irregular, pale, medial area. Major veins, subcostal veinlets and wing margin alternating pale and dark brown; crossveins dark brown. Hind wing hyaline, amber on area adjacent to stem of first branch of CuA and crossveins on the remigium; pterostigma dark brown with pale preapical area; longitudinal veins and subcostal veinlets alternating pale and dark brown; crossveins brown; wing margin alternating pale and brown. ***Abdomen*.** Tergites brown; sternites mostly pale brown.

***Morphology* (Fig. 67)**. ***Head*.** Diamond-shaped in frontal view, rugose; vertexal region raised above compound eyes, with lateral rows of reclined setae; area surrounding coronal suture glabrous, with muscle insertion mark; coronal suture distinct; medial area of ​​supra-antennal region not raised, with fine, reclined setae; paraocular area concave. Antenna submoniliform, nearly as long as head and prothorax together; scape as long as wide, cup-shaped, ventrally with a few thickened setae; pedicel as long as wide; flagellum slightly dorsoventrally flattened, with 48–55 flagellomeres, those of proximal 1/3 of the flagellum two times as wide as long, then changing to as long as wide; all articles with medial ring of fine, short setae. Compound eye hemispherical, as wide as ½ of the interocular distance at torulus level. Frons and clypeus narrow, with fine and short setae. Labrum pentagonal with thin, short setae; maxillary palpus with first palpomere as long as wide, second 1.2× as long as wide, third palpomere 3× as long as wide, fourth palpomere 3× as long as wide, fifth palpomere as long as third, all with short and thin setae; mentum with long, thin setae; labial palpus with first palpomere 1.5× as long as wide, second palpomere 3.5 times as long as wide, third palpomere slightly shorter than second, palpimacula ovoid. ***Thorax*.** Pronotum as long as wide, with raised anterior margin, medial and posterior regions; outgrowths covered with pedicellate, thick setae; remaining surface with fine, short setae. Mesonotum slightly wider than long, with abundant, thick, pedicellate setae on medial area. Metanotum ~ 2× as wide as long, scutellum with fine setae. Pteropleura with short, thin setae. ***Foreleg*.** Coxa as long as femur, cylindrical, anterior and posterior surfaces with interspersed fine setae and pedicellate, thickened setae of different lengths; trochanter trapezoidal, with thin and short setae, except on dorsal surface with some thickened, pedicellate setae near distal margin; anterior surface without protuberant area. Femur robust, covered with abundant, fine, short setae; closing surface with posteroventral row of processes composed two medially located, primary processes; proximal portion of the row with sub-basal secondary process; the rest of the row with abundant tubercle-shaped processes and stinger-shaped setae; distal portion raised composed of tubercle-shaped specializations and stinger-shaped setae; adjacent row of thickened setae with globular base present on distal ¼. Anteroventral row of processes reduced to proximal ½; it is composed of tubercle-shaped specializations and stinger-shaped setae; the basal-most primary, curved process is present; distal portion composed of a few tubercle-shaped processes; adjacent row of thickened setae with globular base present on distal 2/3. Tibia almost as long as femur, curved, with thin, short setae; ventral surface keeled with prostrate setae; a patch of clavate setae apically on anterior surface is present. Basitarsus with lanceolate process reaching the middle of fourth tarsomere; clavate setae present proximally on anterior surface; ventrally with single row of prostrate setae; second tarsomere nearly 7× as long as wide; third tarsomere as long as wide, fourth tarsomere 2.5× as long as wide. ***Mid- and hind leg*.** Coxae with short, thin setae, a few thickened present; trochanter with short, thin setae; femora with interspersed fine setae of different lengths and a few thickened setae; tibiae mostly with short, fine setae; tibial spurs short; hind leg longer than midleg, tibia 1.5× as long as femur; tarsi with fine and short setae, except on distal margin of plantar surface with lateral groups of 3–8 thickened setae; on both legs, basitarsus 3.5 times as long as wide, second tarsomere 1.2× as long as wide; third and fourth tarsomeres as long as wide; fifth tarsomere two times as long as wide. ***Wings*.** Forewing oval, trichosors present along margin except on wing base; venation setose; costal space proximally expanded, humeral vein branched, 11–18 subcostal veinlets; pterostigma elongated, narrow, curved, with complete veinlets; subcostal space with single crossvein, medially located; Sc vein abruptly posteriorly bent at proximal pterostigma margin to merge with RA; radial space with two crossveins; *rarp2* gently curved with two or three RP branches; three or four veins arising from *rarp1*; M vein basally fused with R; RP base, widely separated from divergence of M and R; M forked opposite to RP origin, 1 r-m connecting RP base and M fork, forming a trapezoidal cell; four or five gradate crossveins present. Cubitus deeply forked; CuP basally angled and approaching A1, distally forked slightly beyond the level of separation of M and R; A1 apically forked, ending on posterior margin at level of CuP fork, A2 forked slightly beyond CuP angle level. Hind wing smaller and narrower than forewing, narrowly oval; costal space narrow and reduced, with 7–9 veinlets; C and Sc fused at ¼ of wing length, Sc vein abruptly curved posteriad at proximal margin of pterostigma to merge RA; pterostigma elongated, narrow, gently curved, composed complete veinlets; radial space with single crossvein, oblique; three or four veins arising from *rarp1*, one from *rarp2*. 1r-m sigmoid, connecting the stems of M and RP. Media forked slightly beyond R fork. Cubitus deeply forked, intracubital crossvein subparallel to longitudinal wing axis; CuA sinuous, first branch candelabrum-shaped, spur vein absent; CuP not touching A1, strongly anteriorly bent at distal ½, pectinate; two crossveins on cubitoanal space; A1 simple, ending on wing margin at 1r-m stem level; A2 simple, short, and curved. ***Abdomen*.** Cylindrical to medially expanded, tergites subquadrate, setae scattered, thin, long. Sternites rectangular, with abundant long and thin setae.

**Figure 68.**
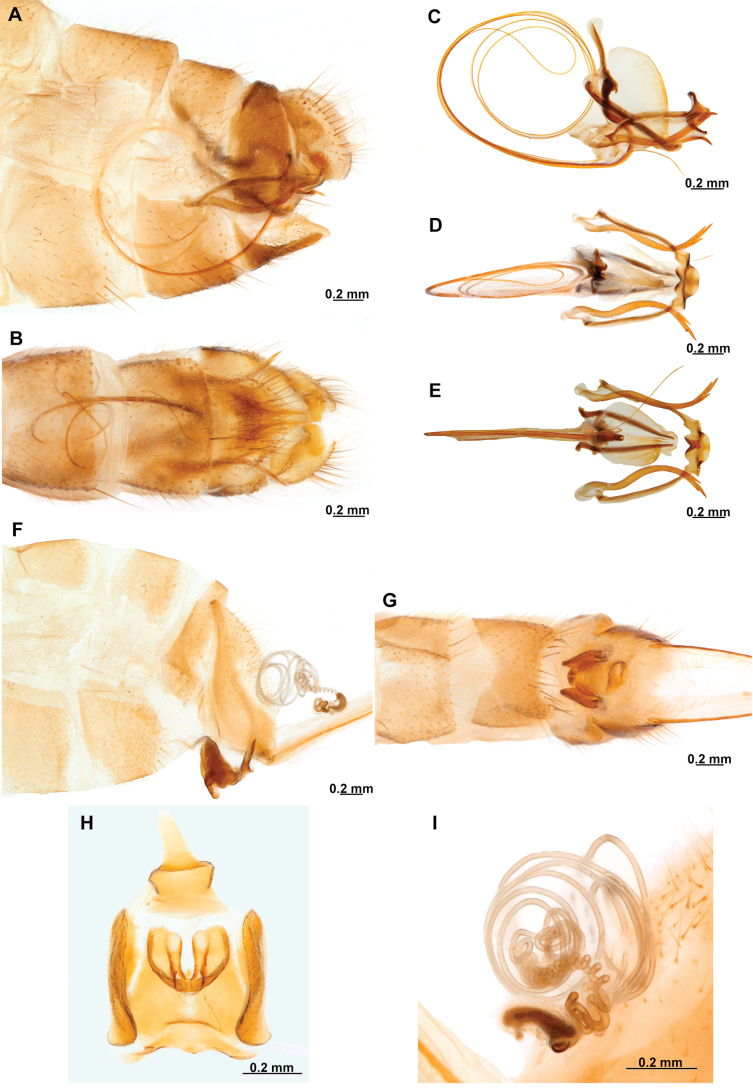
*Plegasonorae* Ardila et al., 2019 **A** male terminalia, lateral **B** same, ventral **C** male genitalia, lateral **D** same, dorsal **E** same, ventral **F** female terminalia, lateral **G** same, ventral **H** gonapophyses VIII ventral **I** spermatheca.

***Male genitalia*** (Fig. 68A–E). Tergite IX medially narrower than laterally; lateral margin rounded. Sternum VIII rectangular; sternite IX pentagonal in ventral view; posterior margin with medial lobe elongate, blunt, dorsally canaliculated; in lateral view triangular, apex reaching posterior margin of ectoproct. Gonocoxites IX short, thickened, sinusoid; posterior apex laterally curved, with two or three processes, of which one is apical and longer and one or two are preapical and shorter. Ectoproct ovoid, covered with long and thin setae on posterior surface; callus cerci vestigial; anteroventrally with flattened, rounded lobe that is continuous with ventromedial sclerotized, incurved, concave area. Gonocoxites X forming a slightly elongated, ventrally canaliculated sclerite, whose posterior apex has dorsal processes connected to gonostyli X and ventrolateral processes connected to gonapophyses X with a membrane; anterior apex expanded and dorsally bent; gonostyli X with thickened, straight base, equipped two lateral processes, the rest of the structure, whip-shaped, ventrally curved, and anteriorly coiled, forming two loops before protruding from abdomen. Gonapophyses X long, straight, narrow, with posterior apex curved dorsad; gonapophyses arranged in a V-shaped structure, joined by a membrane covering the gonostyli X base; this membrane is slightly medially sclerotized. Gonocoxites U-shaped, medial lobe complex and elaborated, with two differentiated parts: dorsal part as a rounded arch, anteriorly curved lobe; ventral part with a convex area covered with microspinules, continuous with ventral, caudally curved process, whose apex is bilobed; between these parts a semicircular, less sclerotized, hyaline area is present; lateral arms of gonocoxites XI gently straight and thin, with anterior apex straightly angled. Hypandrium internum triangular.

***Female genitalia*** (Fig. 68F–I). Sternum VII trapezoidal with broad posteromedian concavity with long setae near posterior margin. Tergite VIII narrower medially than laterally, encircling the spiracle of the segment; lateral margin rounded. Gonocoxites VIII a narrow sclerite fused to gonapophyses VIII. Gonapophyses VIII as a trapezoidal sclerite with medial keel, produced posteromedially and forming a medial Y-shaped process which is caudally bent; laterally folded beneath IX + ectoproct, an oval, convex plate is connected to medial part of gonapophyses VIII. Tergite IX + ectoproct narrowly ovoid. Gonocoxites IX remarkably elongated, sinuous, and narrow, nearly as long as the last seven abdominal segments. Bursa copulatrix long, funnel-shaped, with proximal part strongly sclerotized, the rest membranous and striated. Spermatheca long, complex, and entangled; proximal section, thin, long, forming numerous coils; medial section as wide as proximal section, forming a long spiral; distal section entangled, as wide as medial section, terminating in an expanded portion, where a blunt diverticulum is present; fertilization canal duct proximally triangular, concave; fertilization canal elongated, curved, covered with microfilaments.

##### Distribution.

Mexico (Colima, Sinaloa, Sonora), USA (Arizona).

##### Remarks.

This species is known from Northwestern Mexico and Arizona, being relatively common in xeric environments. It was recovered as part of the Nearctic species as sister of *P.megaptera*. The short prothorax, the wing venation and the male gonocoxites XI of this species are markedly similar to *P.insolita*. The shape of the female gonapophyses VIII of this species is quite distinctive, but the spermatheca is marked similar to other closely related species like *P.dactylota* or *P.signata*. It can be differentiated from *P.megaptera* by the short male gonocoxites IX set with two or three digitiform processes.

#### ﻿﻿ Plega spinosa

Taxon classificationAnimaliaNeuropteraRhachiberothidae

Ardila-Camacho et al., 2019

Figs 69 , 70

Plega spinosa Ardila et al., 2019: 360. Holotype: male, Mexico, Oaxaca (CNIN), specimen examined.

##### Material examined.

***Holotype*.** Mexico • ♂; **Oaxaca**, San Juan B. Cuicatlán, Dominguillo; 17°38'90.7"N, 96°54'70.3"W; 760 m; 25 Jan. 1998; S. Zaragoza, F. Noguera, E. Ramírez, E. González leg.; Bosque tropical caducifolio; trampa de luz, MDM0180; CNIN. ***Paratypes*.** Mexico – **Guerrero** • 1 ♂; Atoyac-Nueva Delhi, El Ranchito; 20 Apr. 1988; A. Cadena, M. García, L. Cervantes leg.; CNIN. – **Oaxaca** • 1 ♂; San Juan B, Cuicatlán, Dominguillo; 17°38'90.7"N, 96°54'70.3"W; 760 m; 22 Jan. 1998; S. Zaragoza, F. Noguera, E. Ramírez, E. González leg.; light trap 3, bosque tropical caducifolio; MDM0175, 2725; CNIN. • 1 ♂; same data as for preceding; MDM0174, 2718; CNIN. • 1 ♂; same data as for preceding; 25 Mar. 1998; MDM0167, 2728; CNIN. • 1 ♂; San Juan B., Cuicatlán, Dominguillo; 24 Apr. 1998; S. Zaragoza leg.; light trap 2, bosque tropical caducifolio; MDM0147, 2713; CNIN. • 1 ♂; same data as for preceding; MDM0143, 2701; CNIN. • 1 ♂; same data as for preceding; MDM0145, 2709; CNIN. • 1 ♂; same data as for preceding; 24 Mar. 2004; MDM0135, 2702; CNIN. – **Puebla** • 1 ♂; Jolalpan, Rancho el Salado; 18°20'49.3"N, 98°59'23"W; 923 m; 06 Oct. 2010; V. Toledo, F. Hinterholzer, J.G. Martinez leg.; selva baja caducifolia, light trap; SHM222; CNIN. • 1 ♂; Zapotitlán de las Salinas, Jardín Botánico Helia Bravo Hollis; 18°19'57.06"N, 97°27'30.06"W; 1488 m; 23–24 Mar. 2017; P. Abad, F. Acevedo, R. López leg.; matorral xerófilo, light trap; PAPIIT00015; CNIN. • 1 ♂; same data as for preceding; PAPIIT00045; CNIN. • 1 ♂; same data as for preceding; PAPIIT00005; CNIN. • 1 ♂; same data as for preceding; PAPIIT00008; CNIN. • 1 ♂; same data as for preceding PAPIIT00048; CNIN. • 1 ♂; same data as for preceding; PAPIIT00059; CNIN. • 1 ♂; same data as for preceding; PAPIIT00034; CNIN. • 1 ♂; same data as for preceding; PAPIIT00046; CNIN. • 1 ♂; same data as for preceding; PAPIIT00043; CNIN. • 1 ♂; same data as for preceding; PAPIIT00037; CNIN. • 1 ♂; same data as for preceding; PAPIIT00032; CNIN. • 1 ♂; same data as for preceding; PAPIIT00013; CNIN. • 1 ♂; same data as for preceding; PAPIIT00038; CNIN. • 1 ♂; same data as for preceding; PAPIIT00009; CNIN. • 1 ♂; same data as for preceding; PAPIIT00025 (CNIN).

##### Other material.

Mexico – **Estado de México** • 1 ♀; Km 72 carr. Toluca-Ixtapan de la Sal, Puente Nenetzingo; 03 Oct. 2000; E. Barrera, H. Brailovsky leg.; CNIN. – **Guerrero** • 1 ♀; Zihuaquito; 15 Apr. 1988; L. Cervantes, M. García, A. Cadena leg.; CNIN. • 3 ♂ 3 ♀; Ixcateopan de Cuauhtémoc, La Barranca; 18°30'17.37"N, 99°47'15.6"W; 2136 m; 28 Aug. 2018–17 Oct. 2018; E. González-Gaona leg.; emerged from pupae of *Monoctenus* sp.; CNIN. – **Oaxaca** • 1 ♀; Dominguillo; 17°38'90.7"N, 96°54'70.3"W; 760 m; 22 Feb. 1998; S. Zaragoza; CNIN. • 1 ♀; same data as for preceding; 22 Mar. 1998; CNIN. • 1 ♂; 23.5 Km SSE de Cuicatlán; 17°37.582'N, 96°55.121'W; 940 m; 21 Aug. 1998; M.A. Morales; CNIN. • 1 ♀; San Juan B, Cuicatlán, Dominguillo; 17°38'90.7"N, 96°54'70.3"W; 760 m; 22 Jan. 1998; S. Zaragoza, F. Noguera, E. Ramírez, E. González leg.; light trap 3, bosque tropical caducifolio, MDM0178, 2706; CNIN. • 1 ♀; same data as for preceding; 25 Mar. 1998; MDM0166, 2716; CNIN. • 1 ♀; same data as for preceding; 22 Jan. 1998; MDM0177, 2714; CNIN. • 1 ♀; San Juan B, Cuicatlán, 23.5 Km SSE de Cuicatlán; 17°37'58.2"N, 96°55'12.1"W; 940 m; 25 Jan. 1998; S. Zaragoza, F. Noguera, E. Ramírez, E. González leg.; light trap 2, MDM0185, 2727; CNIN. • 1 ♀; San Juan B., Cuicatlán, Dominguillo; 24 Feb. 2004; S. Zaragoza leg.; light trap 2, bosque tropical caducifolio; MDM0139, 2712; CNIN. • 1 ♀; same data as for preceding; 24 Apr. 1998; MDM0153, 2711; CNIN. • 1 ♀; same data as for preceding; 24 Apr. 1998; MDM0152, 2708; CNIN. • 1 ♀; same data as for preceding; 24 Apr. 1998; MDM0151; CNIN. • 1 ♀; same data as for preceding; 24 Mar. 2004; MDM0141, 2715; CNIN. • 2 ♂ 1 ♀; Ixtlán de Juárez, Río Grande; 2627; CNIN. • 1 ♂; Pochutla, Candelaria, Loxichá, Santa Rita; 15°55'48.972"N, 96°32'4.992"W; 541 m; 12 Aug. 2018; F. Acevedo, A. Ramírez, D. Curoe leg.; CNIN. • 1 ♂; Reserva Tehuacán-Cuicatlán, Barranca afluente del Río Calapa; 18°9'15.984"N, 97°16'32.988"W; 1118 m; 18 Aug. 2018; F. Acevedo, A. Ramírez, D. Curoe leg.; 2734; CNIN. • 2 ♀; same data as for preceding; 2735; CNIN. – **Puebla** • 1 ♀; S. Teotlalco; 18°24'48.168"N, 98°47'37.86"W; 1013 m; 04 May. 2012; Hernández, Rendón, Toledo, Prado leg.; selva baja caducifolia, light trap; SHM140; CNIN. • 1 ♂; 3 mi. S. Petlalcingo; 06 Oct. 1986; R. Miller & L. Stange leg.; FSCA. • 1 ♀; 3 mi. North Petlalcingo; 03 Aug. 1963; F.D. Parker & L.A. Stange leg.; FSCA. • 1 ♂; 3 mi north Petlalcingo; 21 Aug. 1963; F.D. Parker & L.A. Stange leg.; FSCA. • 1 ♀; 3 mi. E. Izúcar de Matamoros; 25 Apr. 1962; F.D. Parker & L.A. Stange leg.; FSCA. • 1 ♀; Puebla, 7 mi NE. Zapotitlán; 01 Mar. 1972; F. Parker & D. Miller leg.; USNM. • 1 ♀; Jolalpan, Rancho el Salado; 18°20'49.3"N, 98°59'23"W; 923 m; 06 Oct. 2010; V. Toledo, F. Hinterholzer, J.G. Martinez leg.; selva baja caducifolia, light trap; SHM160; CNIN. • 1 ♀; Jolalpan, Rancho el Salado, Ladera W. del cerro Colorado; 18°20'11.6"N, 98°58'55.3"W; 1022 m; 05 Oct. 2010; V. Toledo, F. Hinterholzer, J.G. Martínez leg.; selva baja caducifolia, light trap; SHM138; CNIN. • 1 ♀; Jolalpan, Rancho el Salado, Ladera W de Cerro Colorado; 18°20'11.616"N, 98°58'55.2"W; 1022 m; 05 Oct. 2010; V.H. Toledo, F. Hinterholzer, J.G. Martínez leg.; selva baja caducifolia, light trap; SHM170; CNIN. • 1 ♀; same data as for preceding; SHM161; CNIN. • 1 ♀; same data as for preceding; SHM169; CNIN. • 1 ♀; same data as for preceding; SHM172; CNIN. • 1 ♀; same data as for preceding; SHM159; CNIN. • 1 ♀; Jolalpan, Rancho el Salado; 18°20'49.38"N, 98°59'23.028"W; 923 m; 06 Oct. 2010; V.H. Toledo, F. Hinterholzer, J.G. Martínez leg.; selva baja caducifolia, light trap; SHM183; CNIN. • 1 ♀; same data as for preceding; SHM190 CNIN. • 1 ♀; same data as for preceding; SHM149; CNIN. • 1 ♀; same data as for preceding; SHM186; CNIN. • 1 ♀; same data as for preceding; SHM129; CNIN. • 1 ♀; Zapotitlán de las Salinas, Jardín Botánico Helia Bravo Hollis; 18°19'57.06"N, 97°27'30.06"W; 1488 m; 23–24 Mar. 2017; P. Abad, F. Acevedo, R. López leg.; matorral xerófilo, light trap; PAPIIT00069; CNIN. • 1 ♀; same data as for preceding; PAPIIT00070; CNIN. 1 ♀; same data as for preceding; PAPIIT00063; CNIN. • 1 ♀; same data as for preceding PAPIIT00053; CNIN. • 1 ♀; same data as for preceding; PAPIIT00062; CNIN. • 1 ♀; same data as for preceding; PAPIIT00056; CNIN. • 1 ♀; same data as for preceding; PAPIIT00057; CNIN. • 1 ♀; same data as for preceding; PAPIIT00047; CNIN. • 1 ♀; same data as for preceding; PAPIIT00007; CNIN. • 1 ♀; same data as for preceding; CNIN. • 1 ♀; same data as for preceding; PAPIIT00004; CNIN. • 1 ♀; same data as for preceding; PAPIIT; CNIN. • 1 ♀; same data as for preceding; PAPIIT00031; CNIN. • 1 ♀; same data as for preceding; PAPIIT00033; CNIN. • 1 ♀; same data as for preceding; PAPIIT00041; CNIN. • 1 ♀; same data as for preceding; PAPIIT00049; CNIN. • 1 ♀; same data as for preceding; PAPIIT00024; CNIN. • 1 ♀; same data as for preceding; PAPIIT00019; CNIN. • 1 ♀; same data as for preceding; CNIN. • 1 ♀; same data as for preceding; PAPIIT00026; CNIN. • 1 ♀; same data as for preceding; PAPIIT00022; CNIN. • 1 ♀; same data as for preceding; PAPIIT00018; CNIN. • 1 ♀; same data as for preceding; PAPIIT00052; CNIN. • 1 ♀; same data as for preceding; PAPIIT00018; CNIN. • 1 ♀; same data as for preceding PAPIIT00036; CNIN. • 1 ♀; same data as for preceding; PAPIIT00027; CNIN. • 1 ♀; same data as for preceding; PAPIIT00017; CNIN. • 1 ♀; same data as for preceding; PAPIIT00035; CNIN. • 1 ♀; same data as for preceding; PAPIIT00060; CNIN. • 1 ♀; same data as for preceding; PAPIIT00021; CNIN. • 1 ♀; same data as for preceding; PAPIIT00068; CNIN. • 1 ♀; same data as for preceding; PAPIIT00054; CNIN. • 1 ♀; same data as for preceding; PAPIIT00067; CNIN. • 1 ♀; same data as for preceding; PAPIIT00042; CNIN. • 1 ♀; same data as for preceding; PAPIIT00044; CNIN. • 1 ♀; same data as for preceding; PAPIIT00055; CNIN. • 1 ♀; same data as for preceding; PAPIIT00058; CNIN. • 1 ♀; same data as for preceding; PAPIIT00066; CNIN. • 1 ♀; same data as for preceding; PAPII00003; CNIN. • 2 ♀; Zapotitlán de las Salinas, Jardín Botánico; 18°19'57"N, 97°27'30"W; 24 Feb. 2017; F. Acevedo, P. Abad, R. López leg.; light trap, “matorral xerófilo, cactus y mezquite”; CNIN. • 1 ♂; same data as for preceding; recepción; PAPIIT00099; CNIN. • 1 ♂; same data as for preceding; recepción; PAPIIT00093; CNIN. • 8 ♀; Zapotitlán de las Salinas, J.B. Helia Bravo Hollis; recepción; 18°19'57"N, 97°27'30"W; 24 Feb. 2017; light trap; “matorral xerófilo dominado por cactus y mezquite”; CNIN. • 2 ♀; same data as for preceding; 2737; CNIN. • 1 ♀; Zapotitlán de las Salinas, J.B. Helia Bravo Hollis; 18°19'37"N, 97°27'13"W; 25 Feb. 2017; H. Cabañas; light trap; CNIN. • 1 ♀; Jardín Botánico Helia Bravo Hollis; 09 Jun. 2019; I. Garzón leg.; CNIN.

##### Diagnosis.

This species has the antennal flagellum brown, generally with 2–5 pale, preapical flagellomeres; the proximal flagellomeres are as long as wide. The area adjacent to frontal sutures is sunken, and the ​​supra-antennal is laterally raised, set with abundant, reclined setae. The forefemur posterior surface is pale with brown dots; the anterior surface is dark brown, with pale base. The pterostigma of both wings is brown with palea areas. On the male genitalia, the gonocoxite IX is thin, sinusoid, and short; the posterior apex is pointed, and without processes. The posteroventral surface of the ectoproct is covered with prominent, spine-shaped setal bases. The gonostyli X is short, and posteriorly recurved at midlength. The ventral part of gonocoxites XI median lobe is caudally projected with a Y-shaped outgrowth, which is continuous with ventral, blunt process, set with medial ridge. On the female genitalia, the gonapophyses VIII medial part is chamber-shaped, caudally projected into a Y-shaped process. The spermatheca is short, proximally forming a blunt diverticulum; the distal section terminates in an expanded portion, where a short and blunt diverticulum is present.

##### Description.

***Measurements*.** Male (*n* = 10). Forewing length: 5.9–14.3 mm; Hind wing length: 4.5–10.7 mm.

**Figure 69.**
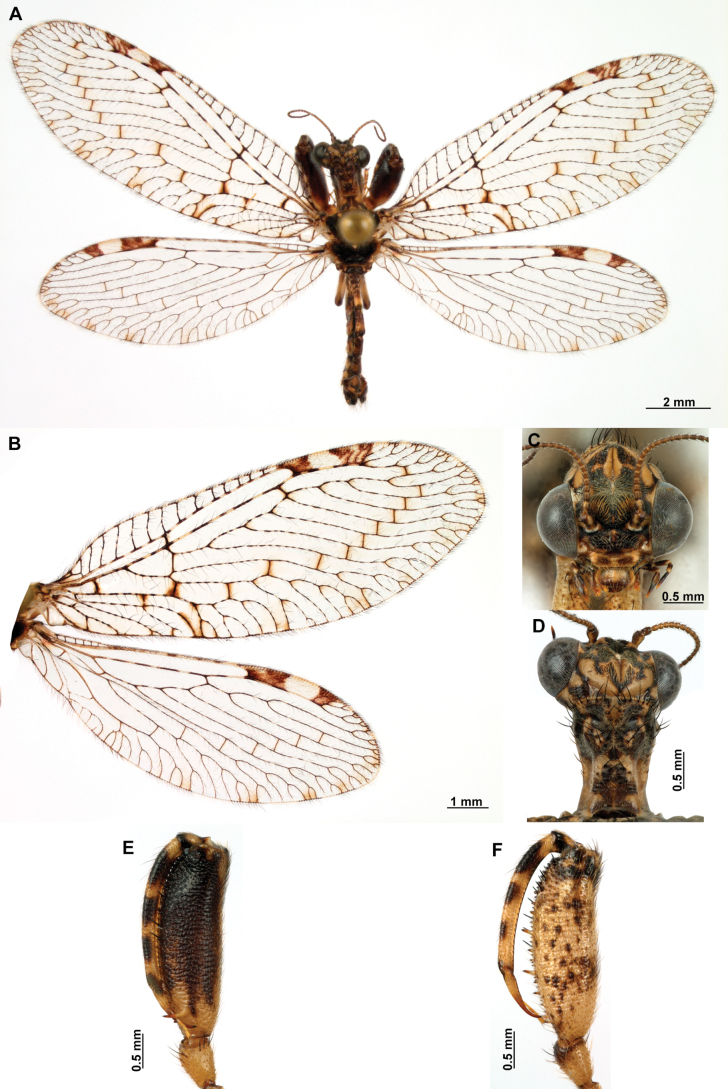
*Plegaspinosa* Ardila et al. 2019 **A** male habitus, dorsal **B** wings **C** head, frontal **D** pronotum, dorsal **E** forefemur, anterior surface **F** same, posterior surface.

***Coloration*** (Fig. 69). ***Head*.** Vertexal region pale, with lateral dark brown marks extending from occipital ridge to supra-antennal area, being broken or embracing pale areas, with pale brown setae; area adjacent to occipital ridge dark brown; supra-antennal area dark brown with pale lateral areas, with pale brown setae; occiput and postgena pale with dark brown areas. Antennal scape dark brown, with pale, longitudinal, dorsal and ventral bands, with interspersed pale and dark brown setae; pedicel brown with ventral paler stripe; flagellum brown, generally with 2–5 pale, preapical flagellomeres. Frons dark brown. Clypeus pale with lateral brown marks; labrum brown with pale anteromedial area; mandible pale with dark brown corners, apex amber; maxilla pale with brown areas, palpus dark brown; labium brown with paler and darker areas, palpus dark brown, palpimacula pale brown. ***Thorax*.** Pronotum pale brown, with darker anterolateral areas, medial region and anterior margin with small pale; episternum dark brown, with pale medial area; postfurcasternum brown with dark brown suffusions near margins. Mesonotum dark brown, with pale areas adjacent to sutures; metanotum dark brown with pale anteromedial area; pre-episternum dark brown; pteropleura with pale and dark brown areas and pale regions, pale, and dark brown setae present. ***Foreleg*.** Coxa pale with dark brown marks at apex, interspersed pale and dark brown setae; trochanter pale, setation mostly pale brown, dorsally with some dark brown setae. Femur posterior surface pale with brown dots, apex dark brown; anterior surface mostly dark brown, pale at base. Tibia dashed with pale and brown. Basitarsus pale at basal ½, lanceolate process brown, clavate setae pale brown; remaining tarsomeres pale brown. ***Mid- and hind leg*.** Coxae pale with brown marks, with interspersed pale and dark brown setae; trochanter of both legs pale with brown marks. Femora and tibiae pale with brown rings, with pale and dark brown setae; in both legs, basitarsus pale, eutarsus pale brown; distal margins on ventral surface with dark brown setae; remaining surface with pale and dark brown setae. ***Wings*.** Forewing mostly hyaline; membrane surrounding forks of longitudinal veins, crossveins, first branch of CuA and apex of CuP amber; posterior and apical margins with intermittent, amber areas between apical branches of longitudinal veins; pterostigma brown with medial, pale area; major veins, subcostal veinlets, and wing margin alternating pale and brown; crossveins brown. Hind wing hyaline, adjacent area to posterior and apical margins with intermittent amber areas between apical branches of longitudinal veins; pterostigma brown with paler areas, with wide, pale, preapical area; longitudinal veins alternating pale and brown, most of CuA brown; crossveins brown, except sigmoid 1r-m bicolor; wing margin alternating pale and brown. ***Abdomen*.** Tergites either pale with pale brown suffusions or completely brown. Pleural membrane brown, pale on dorsal and ventral margins. Sternites pale with lateral, brown marks.

***Morphology*** (Fig. 69). ***Head*.** Diamond-shaped in frontal view, rugose; vertexal region raised above compound eyes, with lateral rows of reclined setae; area surrounding coronal suture glabrous, with muscle insertion mark; coronal suture distinct, area adjacent to frontal sutures sunken; supra-antennal conspicuously raised laterally, with abundant, fine, reclined setae; paraocular area concave. Antenna filiform, short; scape two times as long as wide, slightly distally expanded, with some thickened setae near distal margin; pedicel slightly longer than wide; flagellum not dorsoventrally flattened, with 26–36 flagellomeres, those of flagellar base as wide as long, then changing to slightly longer than wide on the rest of flagellum; all articles with medial ring of fine, short setae. Compound eye hemispherical, as wide as ½ of the interocular distance at torulus level. Frons and clypeus narrow. Labrum pentagonal with thin, short setae; maxillary palpus with first palpomere as long as wide, second 1.2× as long as wide, third palpomere 4× as long as wide, fourth palpomere 3× as long as wide, fifth palpomere slightly longer than third; mentum with long, thin setae; labial palpus with first palpomere two times as long as wide, second palpomere 4× as long as wide, third palpomere as long as second, palpimacula narrowly ovoid. ***Thorax*.** Pronotum slightly longer than wide, with raised anterior margin, medial and posterior regions; raised regions and anterior and lateral margins covered with pedicellate, thickened setae. Mesonotum two times as wide as long, with scattered, thick, pedicellate setae on medial area. Metanotum ~ 2× as long as wide, glabrous. Pteropleura with short, thin, setae a few thickened on mesanepisternum. ***Foreleg*.** Coxa nearly as long as femur, cylindrical, anterior and posterior surfaces with pedicellate fine or thick setae of different sizes; trochanter trapezoidal, with thin and short setae, except on dorsal and anterior surfaces with some thickened, pedicellate setae; anterior surface with protuberant area. Femur robust, covered with abundant, fine, short setae arising from protuberant bases; closing surface with posteroventral row of processes composed two medially located, primary processes, and one tertiary process between them; proximal portion of the row with sub-basal secondary or tertiary process and basal tertiary process; the rest of the row with numerous tubercle-shaped processes, and stinger-shaped setae; distal ½ raised, composed of tubercle-shaped specializations and stinger-shaped setae; adjacent row of thickened setae with globular base present on distal ¼. Anteroventral row of processes reduced to proximal ½; it is composed of tubercle-shaped specializations and stinger-shaped setae; the basal-most primary, curved process is present; distal portion composed of a few tubercle-shaped processes; adjacent row of thickened setae with globular base present on distal ½. Tibia almost as long as femur, curved, with thin, short setae; ventral surface keeled with prostrate setae; a patch of clavate setae apically on anterior surface is present. Basitarsus with lanceolate process reaching the middle of fourth tarsomere; clavate setae present proximally on anterior surface; ventrally with single row of prostrate setae; second tarsomere nearly 7× as long as wide; third tarsomere as long as wide, fourth tarsomere two times as long as wide. ***Mid- and hind leg*.** Coxae with long and thin setae, some thickened; trochanter with short, thin setae; femora with interspersed fine, long setae; tibiae with short and thin setae; tibial spurs short. Hind leg longer than mid-leg; tibia, 1.5× as long as femur; tarsi with fine and short setae, except on distal margin of plantar surface with lateral groups of 3–5 thickened setae; on both legs, basitarsus 4× as long as wide, second tarsomere 1.2× as long as wide; third and fourth tarsomeres as long as wide; fifth tarsomere two times as long as wide. ***Wings*.** Forewing narrowly oval, trichosors present along margin except on wing base; venation setose; costal space proximally expanded, humeral vein branched, 11–21 subcostal veinlets; pterostigma trapezoidal, elongated, narrow, straight, with distinct veinlets; subcostal space with single crossvein, medially located; Sc vein abruptly posteriorly bent at proximal pterostigma margin to merge the RA; radial space with two crossveins; *rarp2* gently curved with two or three RP branches; three or four veins arising from *rarp1*; M vein basally fused to RA; RP base widely separated from divergence of M and R; M forked nearly opposite to RP origin, 1 r-m connecting RP base and M fork, forming a trapezoidal cell; 4–6 gradate crossveins present. Cubitus deeply forked; CuP basally angled and approaching A1, distally forked opposite to the level of separation of M and R; A1 apically forked, ending on posterior margin at level of CuP fork, A2 forked opposite to CuP angle level. Hind wing smaller and narrower than forewing, narrowly oval; costal space narrow and reduced, with four or five veinlets; C and Sc fused at ¼ of wing length, Sc vein abruptly curved posteriad at proximal margin of pterostigma to merge RA; pterostigma elongated, narrow, straight, composed of well-defined veinlets; radial space with single crossvein, oblique; two or three veins arising from *rarp1*, one or two from *rarp2*. 1r-m sigmoid, connecting the stems of M and RP. Media forked slightly beyond R fork. Cubitus deeply forked, intracubital crossvein subparallel to longitudinal wing axis; CuA sinuous, first branch candelabrum-shaped, spur vein absent; CuP not touching A1, strongly anteriorly bent at distal 1/3, pectinate; two crossveins on cubitoanal space; A1 simple, ending at wing margin at 1r-m stem level; A2 simple, short, and curved. ***Abdomen*.** Tergites quadrangular with anterolateral elongated scars; sternites rectangular.

**Figure 70.**
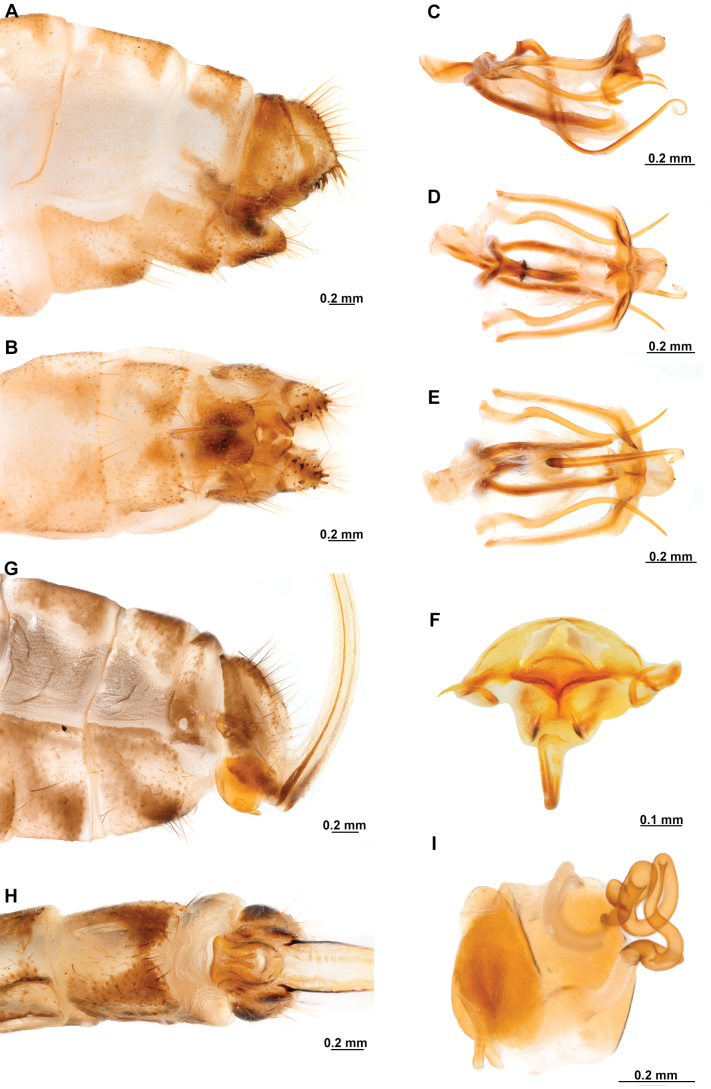
*Plegaspinosa* Ardila et al. 2019 **A** male terminalia, lateral **B** same, ventral **C** male genitalia, lateral **D** same, dorsal **E** same, ventral **F** same, caudal **G** female terminalia, lateral **H** same, ventral **I** gonapophyses VIII and spermatheca.

***Male genitalia*** (Fig. 70A–E). Tergite IX medially narrower than laterally; lateral margin rounded. Sternum VIII rectangular; sternite IX trapezoidal in ventral view, with rounded posterolateral corners, covered with long setae; posterior margin with medial lobe not developed, but corresponding area dorsally canaliculated; in lateral view trapezoidal, not reaching posterior margin of ectoproct. Gonocoxites IX thin, sinusoid, short; base spatulated; apex pointed, without processes. Ectoproct trapezoidal, posteroventral surface covered with prominent spine-shaped setal bases, remaining surface with long, pedicellate setae; anteroventrally with enlarged, rounded, sclerotized lobe, continuous with ventromedial sclerotized, curved sulcus. Gonocoxites X forming a short, straight, ventrally canaliculate sclerite; anterior apex, expanded; posterior apex with dorsal processes connected to gonostyli X and ventrolateral processes connected to gonapophyses X with a membrane; gonostyli X with thickened and curved base with two lateral processes, the rest of the structure, ventrally curved, and posteriorly recurved at mid length before protruding from abdomen, apex sometimes incurved or forming a loop. Gonapophyses X rod-shaped, gently curved; gonapophyses sub-parallel, joined by a membrane covering the gonostyli X base. Gonocoxites XI U-shaped, medial lobe complex and elaborated with two differentiated parts: dorsal part as an arch, with lateral parts expanded; ventral part with a caudally projected, Y-shaped outgrowth, which is continuous with ventral, blunt process, set with medial ridge; between these parts a semicircular, less sclerotized, hyaline area is present; lateral arms of gonocoxites XI short, thin, sigmoid, with anterior apex bent ventrad. Hypandrium internum arrowhead-shaped.

***Female genitalia*** (Fig. 70F–H). Sternum VII trapezoidal with broad posteromedian concavity. Tergite VIII narrower medially than laterally, encircling the spiracle of the segment; lateral margin quadrangular. Gonocoxites VIII as a narrow sclerite. Gonapophyses VIII with medial part chamber-shaped, with caudally projected, Y-shaped process arising at mid length; lateral part of gonapophyses VIII as a rounded, concave plate folded beneath tergite IX + ectoproct. Tergite IX + ectoproct D-shaped. Gonocoxites IX elongated and narrow, nearly as long as the last five abdominal segments; gonapophyses IX as two tiny sclerites located basally on inner side of gonocoxites IX. Bursa copulatrix short, funnel-shaped, with proximal-most part sclerotized, the rest membranous and striated. Spermatheca short and simple; proximal and medial sections undifferentiated, thin, proximal-most part conical, forming a blunt diverticulum; distal section progressively expanded, terminating in an expanded portion, where a short, blunt diverticulum is present; fertilization canal duct with proximal portion triangular, concave; fertilization canal elongated a narrow, C-shaped, covered with microfilaments.

##### Distribution.

Mexico (Estado de México, Guerrero, Oaxaca, Puebla).

##### Remarks.

This is a rather distinctive species distributed in Central Mexico. It was previously reported from the Mexican states of Jalisco, Morelos, Nayarit, and Veracruz by Ardila-Camacho et al. (2019); however, these records constitute misidentified specimens published prior to this revision. Consequently, only the records presented herein are considered as valid. This species is so far known to occur only in Estado de Mexico (first record), Guerrero, Oaxaca, and Puebla.

This species was recovered as sister to *P.oswaldi* inside a clade of species mostly distributed in Central America and Mexico. *Plegaspinosa* can be distinguished by the presence of a conspicuous sunken area on the vertexal region, followed anteriorly by prominent setose areas on the supra-antennal region. The antennal scape is only slightly longer than wide and devoid of thickened setae. On the male terminalia, the ectoproct is set with prominent spiniform setal bases, the male gonocoxites IX are devoid of processes and the gonostyli X are short and simply recurved. The morphology of the medial lobe of the male gonocoxites XI is the best character to accurately recognize this species.

The association between this species with larvae of sawflies of the genus *Monoctenus* Dahlbom, 1835 (Hymenoptera: Symphyta: Diprionidae) as well as some aspects of its biology were recently documented by De Lira-Ramos et al. (2022).

#### ﻿﻿ Plega stangei

Taxon classificationAnimaliaNeuropteraRhachiberothidae

Ardila-Camacho et al., 2019

Figs 71 , 72

Plega stangei Ardila-Camacho et al., 2019: 364. Holotype: male, Mexico, Oaxaca (CNIN), specimen examined.

##### Material examined.

***Holotype*.** Mexico • ♂; **Oaxaca**, Parque Nacional Huatulco, 0.5 Km S. Estación “El Sabanal”; 15°46'0.09"N, 96°11'31.1"W; 107 m; 04 Sep. 2005; S. Zaragoza, F. Noguera, E. Ramírez, E. González leg.; Trampa de luz 5; MDMO127; CNIN. ***Paratypes*.** Mexico – **Morelos** • 1 ♂; Tlaquiltenango, Estación CEAMISH; 940 m; Feb. 1996; S. Zaragoza leg.; bosque tropical caducifolio; MDM0121, 2704; CNIN. • 1 ♂; same data as for preceding; MDM0122, 2710; CNIN.

##### Other material.

Mexico – **Campeche** • 1 ♀; Zona Arqueológica Calakmul, Aguada Grande; 15 Feb. 1998; Contreras leg.; CNIN. – **Morelos** • 1 ♀; Tlaquiltenango, Estación CEAMISH; 940 m; Feb. 1996; S. Zaragoza leg.; bosque tropical caducifolio; MDM0120; CNIN. • 1 ♀; same data as for preceding; MDM0118; CNIN. • 1 ♀; same data as for preceding; MDM0119; CNIN.

##### Diagnosis.

The antennal flagellum is pale brown, with proximal flagellomeres slightly wider than long. The area adjacent to frontal sutures is slightly sunken, and the ​​supra-antennal area is moderately raised laterally, covered with fine, reclined setae. The pterostigma of both wings is brown with a wide pale area; additional paler areas are present. The forefemur posterior surface is pale with brown dots, whereas the anterior surface is brown with pale, small areas. On the male genitalia, the gonocoxite IX is thin, sinusoid, and long; the posterior apex is set with 10–12 subequal processes arranged in a bundle. The ventral part of the gonocoxites XI median lobe forms a triangular, caudally projected process, which is ventrally connected to a narrow arch. On the female genitalia, the gonapophyses VIII medial part is keel-shaped, with distal portion dilated, and produced posteromedially into a short, dorsally bent, bilobed process. The bursa copulatrix is long, with the proximal ½ strongly sclerotized. The spermatheca is long and complex, with proximal and medial sections undifferentiated; the distal section terminates into an expanded portion, where a blunt diverticulum is present.

##### Description.

***Measurements*.** Male (*n* = 3). Forewing length: 10.0–10.6 mm; Hind wing length: 8.0–8.9 mm.

**Figure 71.**
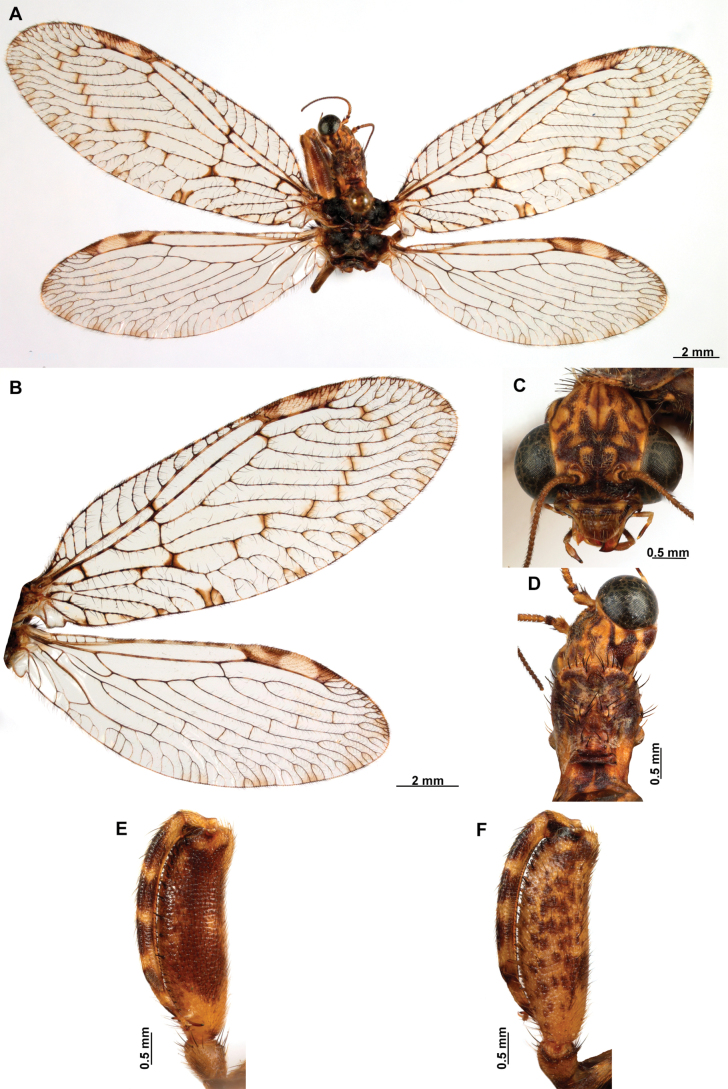
*Plegastangei* Ardila et al., 2019 **A** male habitus, dorsal **B** wings **C** head, frontal **D** pronotum, dorsal **E** forefemur, anterior surface **F** same, posterior surface.

***Coloration* (Fig. 71)**. ***Head*.** Vertexal region pale to pale brown, with lateral brown marks extending from posterior end of coronal suture to supraantennal area being broken near the middle, with pale brown setae; area adjacent to occipital ridge brown; supraantennal area brown with pale lateral areas, with pale brown setae; occiput and postgena pale with pale brown areas. Antennal scape pale brown, with pale, longitudinal, ventral band, entire surface with pale brown setae; pedicel brown, flagellum pale brown with brown setae. Frons pale with lateral pale brown marks. Clypeus pale brown; labrum pale brown with pale margins; mandible pale brown with dark corners, apex amber; maxilla pale with pale brown areas, palpus pale brown; labium pale brown with palpus slightly darker, palpimacula pale brown. ***Thorax*.** Pronotum pale brown with pale areas on medial area, slightly darker on lateral areas; episternum pale brown, with paler medial area; postfurcasternum pale brown. Mesonotum pale with pale brown areas on medial region, lateral regions pale brown, setation mostly pale brown; metanotum with scutum pale on medial regions and pale brown on lateral areas, scutellum mostly pale brown; pre-episternum pale brown; pteropleura pale with pale brown areas, setation mostly pale brown. ***Foreleg*.** Coxa brown; trochanter brown with paler areas, setation mostly pale brown. Femur posterior surface pale with brown dots, apex brown; anterior surface brown with small, pale areas. Tibia dashed with pale and pale brown. Basitarsus pale at basal ½, lanceolate process pale brown, clavate setae pale brown; remaining tarsomeres pale brown. ***Mid- and hind leg*.** Coxae and trochanter pale brown, setae on coxa pale brown, pale on trochanter. Femora and tibiae pale with pale brown rings; tibial spurs pale brown; basitarsus pale, eutarsus pale brown; distal margins on ventral surface with dark brown setae; remaining surface with pale setae; pretarsal claws pale brown. ***Wings*.** Forewing mostly hyaline; membrane surrounding forks of longitudinal veins, crossveins, first branch of CuA and apex of CuP amber; posterior and apical margins with intermittent, amber areas between apical branches of longitudinal veins; pterostigma brown with paler areas, with widened pale, medial area; major veins, subcostal veinlets, and wing margin alternating pale and brown; crossveins brown. Hind wing hyaline, with intermittent amber areas between apical branches of longitudinal veins; pterostigma brown with paler areas, with wide, pale, preapical area; longitudinal veins alternating pale and brown, most of CuA brown; crossveins brown, except sigmoid 1r-m bicolor; wing margin alternating pale and brown. ***Abdomen*.** Tergites pale with pale brown suffusions. Pleural membrane dark brown, pale on dorsal and ventral margins. Sternites pale with pale brown suffusions.

***Morphology* (Fig. 71)**. ***Head*.** Diamond-shaped in frontal view, rugose; vertexal region raised above compound eyes, with lateral rows of reclined setae; area surrounding coronal suture glabrous, with muscle insertion mark; coronal suture distinct, area adjacent to frontal sutures slightly sunken; ​​supra-antennal moderately raised laterally, with fine, reclined setae; paraocular area concave. Antenna filiform, short; scape two times as long as wide, slightly distally expanded, with fine, short setae; pedicel slightly longer than wide; flagellum not dorsoventrally flattened, with 36–44 flagellomeres, which are slightly wider than long at the flagellar base, and then change to as long as wide to slightly longer than wide on the rest of flagellum; all articles with medial ring of fine, short setae. Compound eye hemispherical, as wide as ½ of the interocular distance at torulus level. Frons and clypeus narrow. Labrum pentagonal with thin, short setae; maxillary palpus with first palpomere as long as wide, second 1.2× as long as wide, third palpomere 4× as long as wide, fourth palpomeres 4× as long as wide, fifth palpomere as long as third; mentum with long, thin setae; labial palpus with first palpomere two times as long as wide, second palpomere 5× as long as wide, third palpomere as long as second, palpimacula ovoid. ***Thorax*.** Pronotum as long as wide, with raised anterior margin, medial and posterior regions; outgrowths covered with pedicellate, thick setae; remaining surface with fine, short setae. Mesonotum two times as wide as long, with scattered, thick, pedicellate setae on medial area. Metanotum ~ 2× as long as wide, nearly glabrous. Pteropleura with short, thin, setae a few thickened on episternum. ***Foreleg*.** Coxa as long as femur, cylindrical, anterior and posterior surfaces with pedicellate fine or thick setae of different sizes; trochanter trapezoidal, with thin and short setae, except on dorsal and anterior surfaces with some thickened, pedicellate setae; anterior surface with protuberant area. Femur robust, covered with abundant, fine, short setae arising from protuberant bases; closing surface with posteroventral row of processes composed two medially located, primary processes; proximal portion of the row with sub-basal secondary or tertiary process; the rest of the row with numerous tubercle-shaped processes, and stinger-shaped setae; distal ½ raised, composed of tubercle-shaped specializations and stinger-shaped setae; adjacent row of thickened setae with globular base present on distal ¼. Anteroventral row of processes reduced to proximal ½; it is composed of tubercle-shaped specializations and stinger-shaped setae; the basal-most primary, curved process is present; distal portion composed of a few tubercle-shaped processes; adjacent row of thickened setae with globular base present on distal ⅔–½. Tibia almost as long as femur, curved, with thin, short setae; ventral surface keeled with prostrate setae; a patch of clavate setae apically on anterior surface is present. Basitarsus with lanceolate process reaching the middle of fourth tarsomere; clavate setae present proximally on anterior surface; ventrally with single row of prostrate setae; second tarsomere nearly 8× as long as wide; third tarsomere as long as wide, fourth tarsomere two times as long as wide. ***Mid- and hind leg*.** Coxae and trochanter with short, thin setae; femora with interspersed fine, long setae; tibiae with short and thin setae; tibial spurs short. Hind leg longer than midleg, tibia, 1.5× as long as femur; tarsi with fine and short setae, except on distal margin of plantar surface with lateral groups of 4–6 thickened setae; on both legs, basitarsus 3.5 times as long as wide, second tarsomere 1.2× as long as wide; third and fourth tarsomeres as long as wide; fifth tarsomere two times as long as wide. ***Wings*.** Forewing narrowly oval, trichosors present along margin except on wing base; venation setose; costal space proximally expanded, humeral vein branched, 11–14 subcostal veinlets; pterostigma trapezoidal, elongated, narrow, straight, with distinct veinlets; subcostal space with single crossvein, medially located; Sc vein abruptly posteriorly bent at proximal pterostigma margin to merge with RA; radial space with two crossveins; *rarp2* gently curved with three or four RP branches; three or four veins arising from *rarp1*; M vein basally fused to RA; RP base widely separated from divergence of M and R; M forked nearly opposite to RP origin, 1 r-m slightly sigmoid, connecting RP base and M fork, forming a trapezoidal cell; 5–7 gradate crossveins present. Cubitus deeply forked; CuP basally angled and approaching A1, distally forked opposite to the level of separation of M and R; A1 apically forked, ending on posterior margin at level of CuP fork, A2 forked opposite to CuP angle level. Hind wing smaller and narrower than forewing, narrowly oval; costal space narrow and reduced, with 4–6 veinlets; C and Sc fused at ¼ of wing length, Sc vein abruptly curved posteriad at proximal margin of pterostigma to merge RA; pterostigma elongated, narrow, gently curved, composed of well-defined veinlets; radial space with single crossvein, oblique; three or four veins arising from *rarp1*, 0–2 from *rarp2*. 1r-m sigmoid, connecting the stems of M and RP. Media forked slightly beyond R fork. Cubitus deeply forked, intracubital crossvein subparallel to longitudinal wing axis; CuA sinuous, first branch candelabrum-shaped, spur vein absent or present; CuP not touching A1, strongly anteriorly bent at distal 1/3, pectinate; two crossveins on cubitoanal space; A1 simple, ending on wing margin at 1m-cu level; A2 simple, short, and curved. ***Abdomen*.** Dissected.

**Figure 72.**
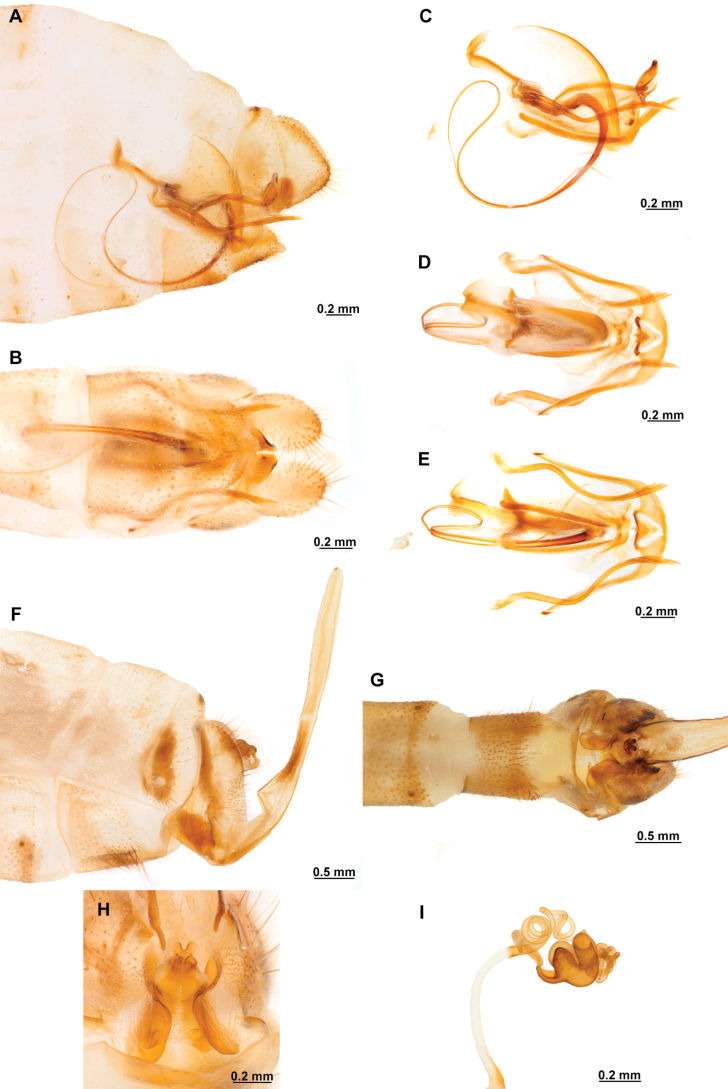
*Plegastangei* Ardila et al., 2019 **A** male terminalia, lateral **B** same, ventral **C** male genitalia, lateral **D** same, dorsal **E** same, ventral **F** female terminalia, lateral **G** same, ventral **H** gonapophyses VIII ventral **I** spermatheca.

***Male genitalia*** (Fig. 72A–E). Tergite IX medially narrower than laterally; lateral margin rounded. Sternum VIII rectangular; sternite IX pentagonal in ventral view, with rounded posterolateral corners, covered with fine, short setae; posterior margin with medial, blunt lobe, which is dorsally canaliculated; in lateral view trapezoidal, apex not reaching posterior margin of ectoproct. Gonocoxites IX thin, sinusoid, long; base spatulated, dorsally curved; apex branched, with 10–12 subequal processes arranged in a bundle. Ectoproct ovoid, covered with thin setae; anteroventrally with rounded, sclerotized lobe, continuous with ventromedial sclerotized, curved sulcus. Gonocoxites X forming an elongate, thickened, ventrally canaliculate sclerite, with anterior apex, expanded, dorsally bent, posterior apex with dorsal processes connected to gonostyli X and ventrolateral processes connected to gonapophyses X with a membrane; gonostyli X with thickened and curved base with two lateral processes, the rest of the structure, whip-shaped, ventrally curved, and anteriorly forming single loop before protruding from abdomen. Gonapophyses X rod-shaped, gently curved; gonapophyses arranged in a V-shaped structure, joined by a membrane covering the gonostyli X base, medial region of this membrane sclerotized. Gonocoxites XI U-shaped, medial lobe complex and elaborated with two differentiated parts: dorsal part as an arch, with lateral parts expanded; ventral part as a triangular caudally projected process, ventrally connected to a narrow arch; between these parts an inverted heart-shaped, less sclerotized, hyaline area is present; lateral arms of gonocoxites XI short, thin, gently curved, with anterior apex incurved. Hypandrium internum tiara-shaped.

***Female genitalia*** (Fig. 72F–I). Sternum VII rectangular with broad posteromedian concavity, posteromedially connected to rounded, sclerotized, glabrous plate. Tergite VIII narrower medially than laterally, encircling the spiracle of the segment; lateral margin trapezoidal. Gonocoxites VIII as lateral sclerotized, trapezoidal plates, medial part less sclerotized. Gonapophyses VIII with medial part keel-shaped, distal portion dilated, produced posteromedially into a short, dorsally bent, bilobed process; lateral part of gonapophyses VIII folded beneath IX + ectoproct, oval, convex. Tergite IX + ectoproct D-shaped. Gonocoxites IX elongated and narrow, nearly as long as the last five abdominal segments. Bursa copulatrix long, funnel-shaped, with proximal ½ strongly sclerotized, the rest membranous and striated. Spermatheca long, complex, and entangled; proximal and medial sections undifferentiated, thin, entangled, not extremely elongated; distal section progressively expanded, forming several loops, terminating in an expanded portion, where a blunt diverticulum is present; fertilization canal duct with proximal portion elongated, triangular, concave; fertilization canal elongated, curved, covered with microfilaments.

##### Distribution.

Mexico (Campeche, Morelos, Oaxaca).

##### Remarks.

This species is distributed in Southern Mexico and was previously known from the states of Morelos and Oaxaca. Here, the first record from Campeche is presented. *Plegastangei* is closely related to *P.vangiersbergenae* and other species from Central America and Mexico (e.g., *P.spinosa*, *P.lachesis*). The body coloration pattern makes this species difficult to separate from *P.pseudohagenella* and *P.vangiersbergenae*, but the male gonocoxites IX and gonostyli X are quite distinctive and allow to distinguish it from the aforementioned species. Conversely, the female genitalia are noticeably similar between the three species, so a careful examination of the color pattern and the degree of sclerotization of the bursa copulatrix can help to distinguish this species.

#### ﻿﻿ Plega vangiersbergenae

Taxon classificationAnimaliaNeuropteraRhachiberothidae

Ardila-Camacho sp. nov.

https://zoobank.org/F5D27A52-13D0-4216-991D-A2741BC7204D

Figs 73 , 74

##### Type locality.

Mexico, **Chiapas**: 25 mi. W Cintalapa, 08 Apr. 1962, L.A. Stange leg.

##### Material examined.

***Holotype*** male, pinned. Original label: “Mexico, **Chiapas**, 25 mi. W Cintalapa, 08 Apr. 1962, L.A. Stange leg.”, FSCA. ***Paratypes***. Mexico • 1 ♂; **Chiapas**, 23 mi. S. Matias Romero; 04 Mar. 1985; L. Stange & R. Miller leg.; FSCA. • 3 ♂ 3 ♀; 6 mi. NW. Villaflores; 10 Apr. 1962; F.D. Parker & L.A. Stange leg.; FSCA. • 1 ♂; **Veracruz**, Tinajas, 23 Apr. 1962; F.D. Parker & L.A. Stange leg.; FSCA. • 1 ♂; Paso de Ovejas; 30 Apr. 1962; F.D. Parker & L.A. Stange leg.; FSCA.

##### Other material.

Guatemala • 1 ♂; **San Marcos**, 17.3 Km SE Talisman, Río Cabaz at Hwy CA2; 14°51'N, 92°04'W; 200 m; 23 May. 1973; Erwin & Hevel leg.; Central American Expedition, 1973; USNM.

Mexico – **Chiapas** • 2 ♀; 6 mi. NW. Villaflores; 10 Apr. 1962; F.D. Parker & L.A. Stange leg.; FSCA. • 1 ♀; 42 mi. W. Cintalapa; 04 Mar. 1985; L. Stange & R. Miller leg.; FSCA. • 1 ♂ 2 ♀; 23 mi. S. Matias Romero; 04 Mar. 1985; L. Stange & R. Miller leg.; FSCA. • 1 ♂; Chorreadera St. Pk.; 26 May. 1987; D.B. Thomas, D.A. Rider, E.G. & T.J. Riley leg.; TAMU-ENTOX0070959; TAMUIC. • 1 ♀; Tuxtla Gutiérrez, Rizo de Oro; 08 May. 1994; C.R. Beutelspacher; CNIN. • 2 ♂ 4 ♀; Rizo de Oro; 11 Apr. 1994; C. Beutelspacher; CNIN. 1 ♀; same data as for preceding; 08 May. 1994; CNIN. – **Oaxaca** • 1 ♂; Gamboa, Crawford leg.; *Symphrasissignata* H.; 7404; CAS. – **Veracruz** • 1 ♀; Apazapan, Km 5 Carrizal-Apazapan; 19°19'29"N, 96°42'11"W; 453 m; 19 Nov. 2008; L. Cervantes leg.; 2726; CNIN.

##### Diagnosis.

This species is recognized because the antennal flagellum is pale brown, with proximal flagellomeres as wide as long. The forefemur posterior surface is pale with brown dots, and the anterior surface is brown. The pterostigma on both wings is brown with pale areas. On the male genitalia, the gonocoxite IX is thin, sinusoid, and long; the posterior apex is thin, with 4–6 subequal processes located on distal ⅓ and arranged as a row on the outer surface. The gonostyli X are coiled, anteriorly forming two loops before protruding from the abdomen. On the female genitalia, the gonapophyses VIII medial part is boat-shaped, posteromedially set with dorsally bent, bilobed process. The bursa copulatrix is short, with proximal ½ strongly sclerotized. The spermatheca has the proximal section spiral-shaped, and the distal section is progressively expanded, terminating in blunt and wide diverticulum.

##### Etymology.

This species is named after the female artist Anneke van Giersbergen, Dutch singer and songwriter in the metal music context.

##### Description.

***Measurements*.** Male (*n* = 16). Forewing length: 9.23–13.76 mm; Hind wing length: 7.8–10.41 mm. Female (*n* = 20): Forewing length: 7.95–13.23 mm; Hind wing length: 6.66–11.43mm.

**Figure 73.**
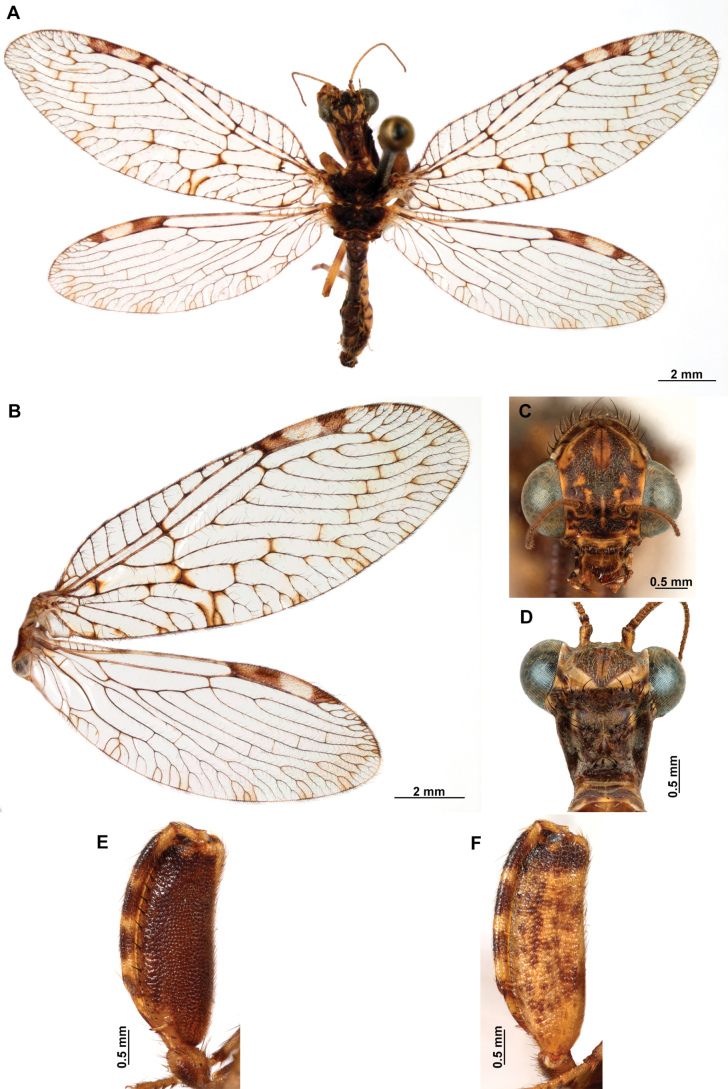
*Plegavangiersbergenae* Ardila-Camacho, sp. nov. **A** male habitus, dorsal **B** wings **C** head, frontal **D** pronotum, dorsal **E** forefemur, anterior surface **F** same, posterior surface.

***Coloration* (Fig. 73)**. ***Head*.** Vertexal region pale to pale brown, with lateral dark brown markings extending from occiput to toruli forming a V-shaped pattern, mainly with pale brown setae; area adjacent to occipital ridge dark brown; supraantennal area dark brown with pale lateral areas, with pale brown setae; occiput pale with brown spot; postgena pale with wide brown area. Antennal scape mostly brown, with pale dorsal longitudinal band, entire surface with pale brown setae; pedicel brown, flagellum pale brown with brown setae. Frons either completely brown or pale with lateral brown marks. Clypeus pale with lateral brown spots; labrum brown with pale lateral margins; mandible pale with dark brown corners, apex amber; maxilla pale with pale brown areas, palpus brown; labium pale with brown areas, setae pale brown; labial palpus brown, palpimacula pale brown. ***Thorax*.** Pronotum mostly brown with pale areas, interspersed pale and dark brown setae present; episternum dark brown, sometimes with pale medial area, postfurcasternum pale brown. Mesonotum mostly brown sometimes with pale areas adjacent to sutures or only two small anterolateral pale areas, setation mostly dark brown; metanotum either entirely dark brown or with anteromedial pale area; pre-episternum brown; pteropleura brown with pale areas, setation mostly pale brown, dark on mesanepisternum. ***Foreleg*.** Coxa brown; trochanter brown with paler and darker areas, setation mostly brown, dorsally with some dark brown setae. Femur posterior surface pale with brown dots, apex brown; anterior surface brown, except area surrounding primary process and apex pale. Tibia dashed with pale and dark brown. Basitarsus pale brown proximally, changing to amber towards the apex, clavate setae pale brown; remaining tarsomeres pale brown. ***Mid- and hind leg*.** Mid- and hind coxa brown with pale areas, setae pale brown; trochanter of both legs pale with brown spots. Femur and tibia of both legs pale with brown rings. Tarsi pale to pale brown with brown setae; distal margins on ventral surface with dark brown setae; pretarsal claws brown. ***Wings*.** Forewing mostly hyaline; membrane surrounding crossveins, first branch of CuA and apex of CuP amber; posterior and apical margins on distal ½ of wing with intermittent, amber areas between apical branches of longitudinal veins; pterostigma brown with pale medial area, sometimes with small, proximal, pale spot; major veins, subcostal veinlets, and wing margin alternating pale and brown. Hind wing hyaline, with amber intermittent amber areas between apical branches of longitudinal veins; pterostigma brown with wide, pale, preapical area; longitudinal veins alternating pale and brown, sometimes mostly brown; crossveins brown, except sigmoid 1r-m, bicolor; wing margin alternating pale and brown. ***Abdomen*.** Tergites brown with pale areas. Pleural membrane dark brown with pale dorsal in ventral margins. Sternites pale with pale brown lateral areas, becoming wider towards the terminal segments.

***Morphology* (Fig. 73)**. ***Head*.** Diamond-shaped in frontal view, rugose; vertexal region raised above compound eyes, with lateral rows of reclined setae; area surrounding coronal suture glabrous, with muscle insertion mark; coronal suture distinct; medial area of ​​supra-antennal moderately raised, with fine, reclined setae; paraocular area concave. Antenna submoniliform, short; scape two times as long as wide, slightly distally expanded, with fine, short setae; pedicel as long as wide; flagellum slightly dorsoventrally flattened, with 33–40 flagellomeres, which are as wide as long on proximal ¼, and slightly longer than wide on the rest of flagellum; all articles with medial ring of fine, short setae. Compound eye hemispherical, as wide as ½ of the interocular distance at torulus level. Frons and clypeus narrow, with fine and short setae. Labrum pentagonal with thin, short setae; maxillary palpus with first palpomere as long as wide, second 1.2× as long as wide, third palpomere 3× as long as wide, fourth palpomeres 2.5× as long as wide, fifth slightly longer than third, all with minute setae; mentum with long, thin setae; labial palpus with first palpomere two times as long as wide, second palpomere 3× as long as wide, third palpomere slightly longer than second, palpimacula narrowly ovoid. ***Thorax*.** Pronotum as long as wide, with raised anterior margin, medial and posterior regions; outgrowths covered with pedicellate, thick setae; remaining surface with fine, short setae. Mesonotum 1.5× as wide as long, with scattered, thick, pedicellate setae on medial area. Metanotum ~ 2× as long as wide, nearly glabrous. Pteropleura with short, thin setae. ***Foreleg*.** Coxa as long as femur, cylindrical, anterior and posterior surfaces with pedicellate fine or thick setae of different sizes; trochanter trapezoidal, with thin and short setae, except on dorsal and anterior surfaces with some thickened setae; anterior surface with protuberant area. Femur robust, covered with abundant, fine, short setae arising from protuberant bases; closing surface with posteroventral row of processes composed two medially located, primary processes, and a tertiary process between the primary ones; proximal portion of the row with two secondary processes; the rest of the row with numerous tubercle-shaped processes, and stinger-shaped setae; distal portion raised, composed of tubercle-shaped specializations and stinger-shaped setae; adjacent row of thickened setae with globular base present on distal ¼. Anteroventral row of processes reduced to proximal ½; it is composed of tubercle-shaped specializations and stinger-shaped setae; the basal-most primary, curved process is present; distal portion composed of a few tubercle-shaped processes; adjacent row of thickened setae with globular base present on distal ½. Tibia almost as long as femur, curved, with thin, short setae; ventral surface keeled with prostrate setae; a patch of clavate setae apically on anterior surface is present. Basitarsus with lanceolate process reaching the middle of fourth tarsomere; clavate setae present proximally on anterior surface; ventrally with single row of prostrate setae; second tarsomere nearly 6× as long as wide; third tarsomere as long as wide, fourth tarsomere two times as long as wide. ***Mid- and hind leg*.** Coxae and trochanter with long, thin setae; femur and tibia of midleg with interspersed fine, long setae and a few thickened setae; tibial spurs short; femur and tibia of hind leg with short and thin setae. Hind leg longer than midleg, tibia, 1.5× as long as femur; tarsi with fine and short setae, except on distal margin of plantar surface with lateral groups of 5–7 thickened setae; on both legs, basitarsus 3.5 times as long as wide, second tarsomere 1.2× as long as wide; third and fourth tarsomeres as long as wide; fifth tarsomere 2.5× as long as wide. ***Wings*.** Forewing narrowly oval, trichosors present along margin except on wing base; venation setose; costal space proximally expanded, humeral vein branched, 9–13 subcostal veinlets; pterostigma elongated, narrow, gently curved, with distinct veinlets; subcostal space with single crossvein, medially located; Sc vein abruptly posteriorly bent at proximal pterostigma margin to merge with RA; radial space with two crossveins; *rarp2* gently curved with 2–4 RP branches; three or four veins arising from *rarp1*; M vein basally fused with RA; RP base widely separated from divergence of M and R; M forked nearly opposite to RP origin, 1 r-m connecting RP base and M fork, forming a trapezoidal cell; 4–6 gradate crossveins present. Cubitus deeply forked; CuP basally angled and approaching A1, distally forked opposite to the level of separation of M and R; A1 apically forked, ending on posterior margin at level of CuP fork, A2 forked opposite to CuP angle level. Hind wing smaller and narrower than forewing, narrowly oval; costal space narrow and reduced, with 5–7 veinlets; C and Sc fused at ¼ of wing length, Sc vein abruptly curved posteriorly at proximal margin of pterostigma to merge RA; pterostigma elongated, narrow, straight, composed of poorly defined veinlets; radial space with single crossvein, oblique; three veins arising from *rarp1*, one or two from *rarp2*. 1r-m sigmoid, connecting the stems of M and RP. Media forked slightly beyond R fork. Cubitus deeply forked, intracubital crossvein subparallel to longitudinal wing axis; CuA sinuous, first branch candelabrum-shaped, spur vein generally absent; CuP not touching A1, strongly anteriorly bent at distal 1/3, pectinate; two crossveins on cubitoanal space; A1 simple, ending on wing margin at 1m-cu level; A2 simple, short, and curved. ***Abdomen*.** Cylindrical to medially expanded, setae on tergites, scattered, thin, and short; tergites subquadrate, tergites III-VI with elongated anterolateral scars. Sternites rectangular, with scattered fine and short setae.

**Figure 74.**
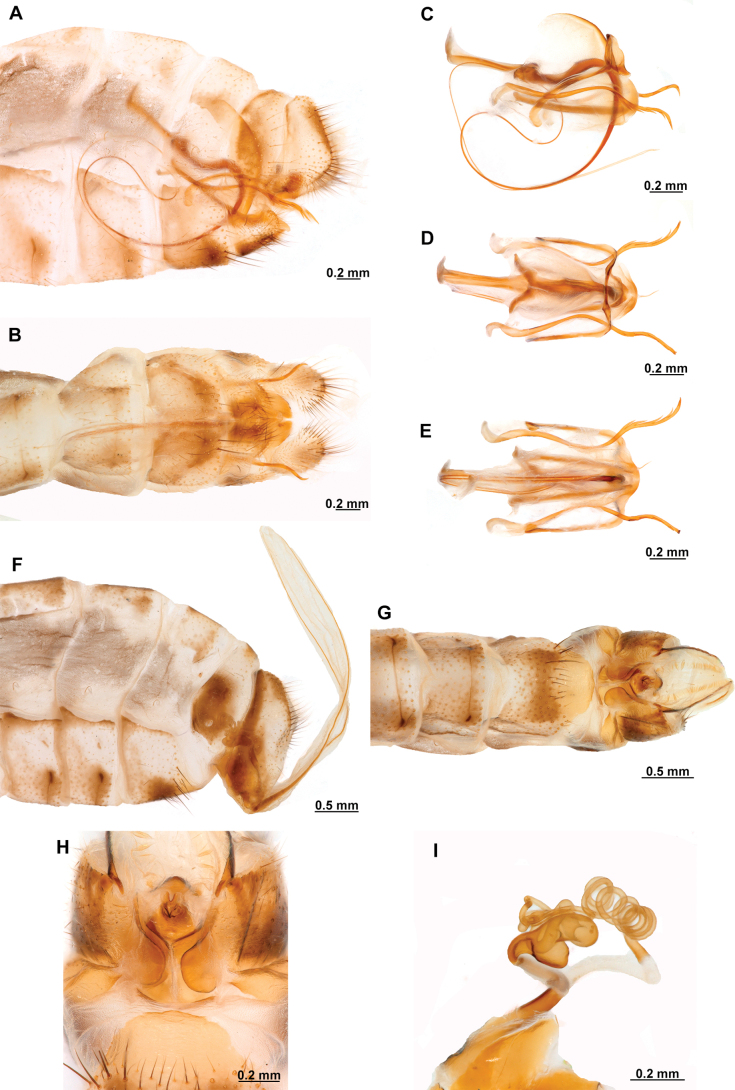
*Plegavangiersbergenae* Ardila-Camacho, sp. nov. **A** male terminalia, lateral **B** same, ventral **C** male genitalia, lateral **D** same, dorsal **E** same, ventral **F** female terminalia, lateral **G** same, ventral **H** gonapophyses VIII ventral **I** spermatheca.

***Male genitalia*** (Fig. 74A–E). Tergite IX medially narrower than laterally; lateral margin rounded. Sternum VIII rectangular; sternite IX subtrapezoidal in ventral view, with rounded posterolateral corners, covered with fine, short and long setae; posterior margin with medial, blunt lobe, which is dorsally canaliculated; in lateral view trapezoidal, apex not reaching posterior margin of ectoproct. Gonocoxites IX thin, sinusoid, long; anterior apex, narrow, sigmoid, connected to gonocoxites XI with a membrane; posterior apex thin, sometimes ventrally curved, with 4–6 subequal processes on distal ⅓ arranged as a row of branches on the outer surface. Ectoproct subtrapezoidal, covered with thin setae; anteroventrally with a fusiform, slightly concave, sclerotized plate. Gonocoxites X forming an elongate, thickened, ventrally canaliculate sclerite, with anterior apex dorsally bent, posterior apex with dorsal processes connected to gonostyli X and ventrolateral processes connected to gonapophyses X with a membrane; gonostyli X with thickened and concave base with two lateral processes, the rest of the structure, whip-shaped, ventrally curved, and anteriorly forming two loops before protruding from abdomen. Gonapophyses X rod-shaped, straight, narrow, arranged in a V-shaped structure; gonapophyses joined by a membrane covering the gonostyli X base; this membrane is medially sclerotized. Gonocoxites XI thin, U-shaped, medial lobe complex and elaborated with two differentiated parts: a dorsal, subtrapezoidal lobe; ventral part as a short process with a triangular convex area; between these parts a heart-shaped, less sclerotized, hyaline area is present; lateral arms of gonocoxites XI short, sigmoid, with anterior apex ventrally bent. Hypandrium internum tiara-shaped.

***Female genitalia*** (Fig. 74F–I). Sternum VII rectangular with posterior margin concave, and continuous with a rounded, sclerotized, glabrous plate. Tergite VIII slightly narrower medially than laterally, encircling the spiracle of the segment; lateral margin blade-shaped. Gonocoxites VIII as a narrow sclerite with lateral concavities. Gonapophyses VIII with medial part boat-shaped with posteromedial, dorsally bent, bilobed process; lateral part as an oval, concave plate folded beneath tergite IX + ectoproct. Tergite IX + ectoproct triangular. Gonocoxites IX elongated, and narrow, as long as the last four abdominal segments together. Bursa copulatrix short, funnel-shaped, proximal ½ strongly sclerotized, the rest membranous and striated. Spermatheca complex and entangled, proximal section, thin, spiral-shaped; medial section as wide as proximal section, forming several convolutions; distal section progressively expanded, terminating in blunt and wide diverticulum; fertilization canal duct proximally triangular, expanded, and concave; fertilization canal C-shaped, covered with microfilaments.

##### Distribution.

Guatemala (San Marcos), Mexico (Chiapas, Oaxaca, Veracruz).

##### Remarks.

This species is known from Southern Mexico, from the states of Chiapas, Oaxaca, and Veracruz. This species is closely related and very similar to *P.stangei*, but the marks on the head, the darker pronotum and the uniformly dark anterior surface of the forefemur can help to separate both species. Additionally, the male genitalia of this species are quite distinctive, with male gonocoxites resembling the spinous stem of a rose, whereas the gonostyli X form two loops. On the female genitalia, the proximal region of the bursa copulatrix is strongly sclerotized.

#### ﻿﻿ Plega yucatanae

Taxon classificationAnimaliaNeuropteraRhachiberothidae

Parker & Stange, 1965

Figs 75 , 76

Plega yucatanae Parker & Stange, 1965: 606. Holotype: male, Mexico, Yucatán (CAS), specimen examined.

##### Material examined.

***Holotype*.** Mexico • ♂; **Yucatán**, Chichen Itzá, Xtoloc cave; 18 Apr. 1962; Parker & L.A. Stange leg.; Type No. 9471; CAS. ***Paratypes*.** Mexico • 1 ♂; **Yucatán**, Chichén Itzá, Xtoloc cave, 18 Apr. 1962, F.D. Parker & L.A. Stange leg.; “Paratype of *Plegayucatanae* F. Parker & L. Stange”; UCD. • 1 ♂; same data as for preceding; CAS. • 1 ♀; same data as for preceding; “62-12-18a”; FSCA. • 3 ♂; same data as for preceding; FSCA. • 1 ♂; same data as for preceding; “EMGD, June 15, 1962”, “62-12-18b”; FSCA.

##### Other material.

Guatemala – **El Progreso** • 1 ♂; Est. de la Virgen; 11–12 Aug. 1965; Flint & Ortiz leg.; USNM. – **Zacapa** • 1 ♂; 12–14 K S. San Lorenzo; 03 Jun. 1989; J.E. Wappes leg.; TAMU-ENTOX0285259; TAMUIC. • 1 ♀; same data as for preceding; TAMU-ENTOX0285133; TAMUIC.

Honduras – **El Paraíso** • 5 ♂ 2 ♀; Yuscarán (Río Aguacote); 2800’; 12 May. 1993; L.Stange & R. Miller leg.; (H-1); FSCA. – **Ocotepeque** • 1 ♀; 32 Km N.E. Ocotepeque; 16 May. 1993; L. Stange & R. Miller leg.; FSCA. – **Francisco Morazán** • 1 ♀; Fac. Agr Panameric El Zamorano; F. Weisson leg.; “captura en un terreno valdío en la maleza”; FSCA.

Mexico – **Chiapas** • 1 ♂; Suchiapa; 18 Jul. 1957; Hard & Durham leg.; FSCA. • 1 ♂; Chiapas, Sta. Isabel, 28 Km S Jct 190/195; 15 Oct. 1988; J.E. Wappes leg.; TAMU-ENTOX0285297; TAMUIC. • 1 ♂; Chorreadera St. Pk.; 26 May. 1987; D.B. Thomas, D.A. Rider, E.G. & T.J. Riley leg.; TAMU-ENTOX0755110; TAMUIC. • 1 ♀; Ocozocoautla, Reserva El Ocote, nr. Campo El Ocote; 17°02'34"N, 93°48'28"W; 900’; 26–29 Jul. 1997; Wooley, González & Galdámez leg.; 97/059 Malaise trap; TAMU-ENTOX0071171; TAMUIC. – **Jalisco** • 1 ♀; Chamela; 29 Sep. 1977; H. Brailovsky leg.; CNIN. • 1 ♀; Estación de Biología Chamela; 02 Oct. 1985; R.A. Usela leg.; light trap; CNIN. – **Yucatán** • 1 ♂; Chichén Itzá; 10–11 Jun. 1983; E.G. Riley leg.; TAMU-ENTOX0085515; TAMUIC.

##### Diagnosis.

This species is distinguished by the presence of a M-shaped dark brown mark, with median U-shaped pale area on the supraantennal area. The antennal flagellum has the proximal ½ pale brown, with flagellomeres as long as wide, the distal ½ is darker with 3–5 pale, preapical articles. The posterior surface of the forefemur is pale with irregular dark brown marks on proximal 1/3 and on distal ½, which may be connected; the anterior surface is either nearly completely dark brown or pale with narrow dark brown marks. The pterostigma on both wings is reddish brown with pale area. The male sternite VIII has a posteromedial, blunt process. The gonocoxite IX is thickened, short, and straight; the posterior apex, generally has two lateral- or ventrally curved, digitiform processes, of which one is slightly longer; a third smaller process may be present. On the female genitalia, the gonapophyses VIII medial part is boat-shaped, with posteromedian thickened, bilobed process. The spermatheca has the proximal and medial sections thin and extremely coiled; the distal section lacks a diverticulum.

##### Description.

***Measurements*.** Male (*n* = 8). Forewing length: 9.3–12.3 mm; Hind wing length: 7.3–9.5 mm. Female (*n* = 5): Forewing length: 7.7–13.0 mm; Hind wing length: 6.3–10.5 mm.

**Figure 75.**
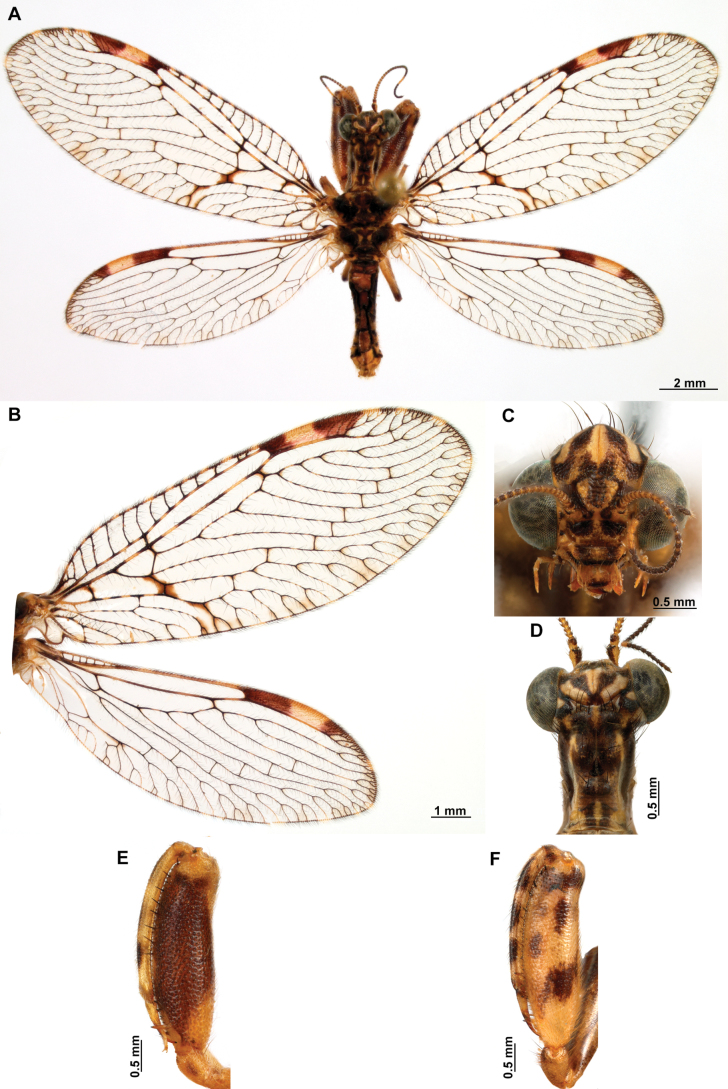
*Plegayucatanae* Parker & Stange, 1965 **A** male habitus, dorsal **B** wings **C** head, frontal **D** pronotum, dorsal **E** forefemur, anterior surface **F** same, posterior surface.

***Coloration* (Fig. 75)**. ***Head*.** Vertexal region pale with dark brown V-shaped inverted mark, sometimes anteriorly connected to markings of the supraantennal area, with interspersed pale and dark brown setae. Occiput with dark brown marks near occipital transverse suture; postgena pale. Supraantennal area with M-shaped dark brown mark, with median U-shaped pale area. Antennal scape pale with brown longitudinal dorsal band; pedicel dark brown with ventral pale area, flagellum with proximal ½ pale brown, distal ½ darker, except for 3–5 pale preapical flagellomeres. Frons pale with dark brown lateral markings. Clypeus pale, sometimes with lateral brown marks, anterior margin brown; labrum pale with anteromedian brown mark; mandible pale with dorsal and ventral corners dark amber, apex amber; maxilla pale with pale brown areas, palpus brown, except fourth palpomere and distal ½ of third palpomere pale, setae pale brown; labium pale with pale brown areas; labial palpus brown, palpimacula pale brown. ***Thorax*.** Pronotum with extensive dark brown marks, with lateral pale stripes, sometimes restricted to anterolateral regions; episternum dark brown with wide, medial pale area, pale brown setae present; postfurcasternum pale with posterolateral area brown. Mesonotum dark brown on lateral areas, medial region pale with irregular brown marks, setation mostly dark brown; metanotum mostly dark brown with pale medial areas; pre-episternum brown with pale areas; pteropleura pale with brown marks. ***Foreleg*.** Coxa dark brown, with medial, small, paler area; trochanter pale with dark brown marks on anterior and ventral surfaces, some dark brown setae dorsally near distal margin. Femur posterior surface pale with irregular dark brown marks on proximal 1/3 and on distal ½, which are sometimes connected; anterior surface either nearly completely dark brown or pale with narrow dark brown marks. Tibia dashed with pale and dark brown. Basitarsus proximal ½ pale brown, lanceolate process dark amber, clavate setae pale brown; remaining tarsomeres pale brown. ***Mid- and hind leg*.** Coxae pale with small brown areas, pale setae present; trochanter pale. Femur of mid-leg pale with medial and apical dark brown rings, on hind leg pale to pale brown with dark apex; tibia of both legs pale with dark brown rings; tibial spurs amber. Tarsi pale brown, with interspersed pale and dark brown setae; distal margins on plantar surface of the first four tarsomeres with dark brown setae; pretarsal claws brown. ***Wings*.** Forewing mostly hyaline, dark brown in area surrounding crossveins, MP stem, first branch of CuA and apex of CuP, and intermittently on apical twigging near posterior and apical wing margin; major veins and subcostal veinlets alternating pale and dark brown, crossveins dark brown; pterostigma reddish brown with medial pale area; wing margin alternating pale and brown. Hind wing mostly hyaline, sometimes with intermittent amber infuscations on the apical twigging of major veins near the posterior and distal margins; pterostigma reddish brown with preapical pale area; C and Sc veins mostly pale brown, remaining longitudinal veins alternating brown and pale, Cu and anal veins mostly pale; crossveins dark brown; wing margin alternating brown and pale. ***Abdomen*.** Tergites mostly brown, pale near the lateral margins, setae mostly pale. Pleural membrane mostly dark brown on abdominal segments IV–VII, pale on remaining segments. Sternites pale with lateral, brown marks; setae mostly pale.

***Morphology* (Fig. 75)**. ***Head*.** Diamond-shaped in frontal view, rugose, vertexal region raised above compound eyes, with lateral rows of reclined setae; area adjacent to coronal suture and occipital ridge, glabrous, with muscle insertion mark; coronal suture distinct; ​​supra-antennal region raised, covered with fine, reclined setae; paraocular area concave. Antenna filiform, short, scape 1.5× as long as wide, slightly distally expanded, with short fine, short setae, a few longer and thicker on dorsal surface; pedicel slightly longer than wide; flagellum not dorsoventrally flattened, with 35–38 flagellomeres, which are as wide as long on proximal portion, slightly longer than wide towards the flagellar apex; all articles with medial ring of short setae. Compound eye hemispherical, as wide as ½ of the interocular distance at torulus level. Frons and clypeus rugose, with fine and short setae. Labrum pentagonal with fine and short setae; maxillary palpus with short and thin setae, first two palpomeres as long as wide, third palpomere 3× as long as wide, fourth palpomere two times as long as wide, fifth palpomere slightly longer than third; mentum with thin, long setae. Labial palpus with first palpomere 1.2× as long as wide, second palpomere 3× as long as wide, third palpomere as long as second, palpimacula narrowly ovoid. ***Thorax*.** Pronotum 1.5× as long as wide, with raised anterior margin, medial and posterior regions; outgrowths covered with pedicellate, thick setae; remaining surface with fine, short setae; postfurcasternum ventrally fused, collar-like. Mesonotum 1.5× as wide as long, with scattered, thick, pedicellate setae and some thin setae on medial area. Metanotum ~ 2× as wide as long, with few, scattered, fine setae. Pteropleura with abundant, short, thin setae. ***Foreleg*.** Coxa as long as femur, cylindrical, anterior and posterior surfaces with abundant, thin setae; trochanter subconical, with thin and short setae, except dorsally with some thick, pedicellate, and longer setae, anterior surface with protuberant area covered with pedicellate, thick setae. Femur robust, covered with abundant, fine, short setae. Closing surface with posteroventral row of processes composed two medially located primary processes and a sub-basal secondary process; tertiary processes are present between the primary processes and the base, the rest of the row with abundant tubercle-shaped processes and stinger-shaped setae; distal portion of posteroventral row slightly raised; adjacent row of thickened setae with globular base present on distal ¼. Anteroventral row of processes reduced to proximal ½ and distal region; it is composed of tubercle-shaped specializations, stinger-shaped setae, and a basal primary, curved process; distal portion composed of a few tubercle-shaped specializations; adjacent row of thickened setae with globular base present on distal ½. Tibia almost as long as the femur, curved, setose, ventrally keeled with prostrate setae row; a patch of clavate setae is present apically on the anterior surface. Basitarsus with lanceolate process reaching the base of fourth tarsomere; clavate setae are present proximally on anterior surface; ventrally with single row of prostrate setae; second tarsomere nearly 7× as long as wide; third tarsomere 1.5× as long as wide, fourth tarsomere 2.5× as long as wide. ***Mid- and hind leg*.** Coxae with abundant, thin setae; trochanter on both legs with abundant fine and short setae; femora and tibiae of both legs with interspersed long and short, thin setae; tibial spurs short; hind leg longer than midleg, tibia, 1.5× as long as femur; tarsi with short, thin setae, except on distal margin of plantar surface with lateral groups of 3–5 thickened setae; on both legs basitarsus 5× as long as wide, second tarsomere 1.2× as long as wide; third and fourth tarsomeres as long as wide; fifth tarsomere 1.5× as long as wide. ***Wings*.** Forewing oval, trichosors present along margin except on wing base; venation setose; costal space proximally slightly expanded, humeral vein forked or branched, 10–13 subcostal veinlets; pterostigma elongated, gently curved, distally wider than proximally, with numerous, branched veinlets; subcostal space with single crossvein, medially located; Sc vein abruptly posteriorly curved at proximal pterostigma margin to merge with RA; radial space with two crossveins; *rarp2* curved with 3–5 RP branches arising from it; two or three veins arising from *rarp1*; M vein fused basally with R; RP base not widely separated from point of divergence of M from R; M forked slightly before RP base, 1 r-m connecting RP base and MA stem, forming a trapezoidal cell; 3–6 gradate crossveins present. Cubitus deeply forked; CuP basally angled and approaching A1, distally forked at level of separation of M and R; A1 simple or apically forked, ending on posterior margin at CuP fork level, A2 forked at CuP angle level. Hind wing smaller and narrower than forewing, narrowly oval; costal space narrow and reduced, with 5–7 veinlets; C and Sc fused at ¼ of wing length, Sc vein abruptly curved posteriorly at proximal margin of pterostigma to merge with RA; pterostigma elongated, curved, narrow, composed of incomplete, mostly simple veinlets; radial space with single crossvein, oblique; two or three veins arising from *rarp1*, 1–3 from *rarp2*. 1r-m sigmoid, connecting the stem of M and RP. Media forked beyond R fork. Cubitus deeply forked, intracubital crossvein subparallel to longitudinal wing axis; CuA sinuous, first branch candelabrum-shaped, spur vein absent; CuP not touching A1, strongly anteriorly bent at distal 1/3, pectinate; two crossveins on cubitoanal space; A1 simple ending on wing margin slightly beyond 1m-cu stem level; A2 simple, short, and curved. ***Abdomen*.** Cylindrical to medially expanded, setae on tergites gradually longer and more abundant towards terminal segments; tergites III–VII with elongated anterolateral scars. Sternites with scattered, thin, short setae, becoming longer towards terminal segments.

**Figure 76.**
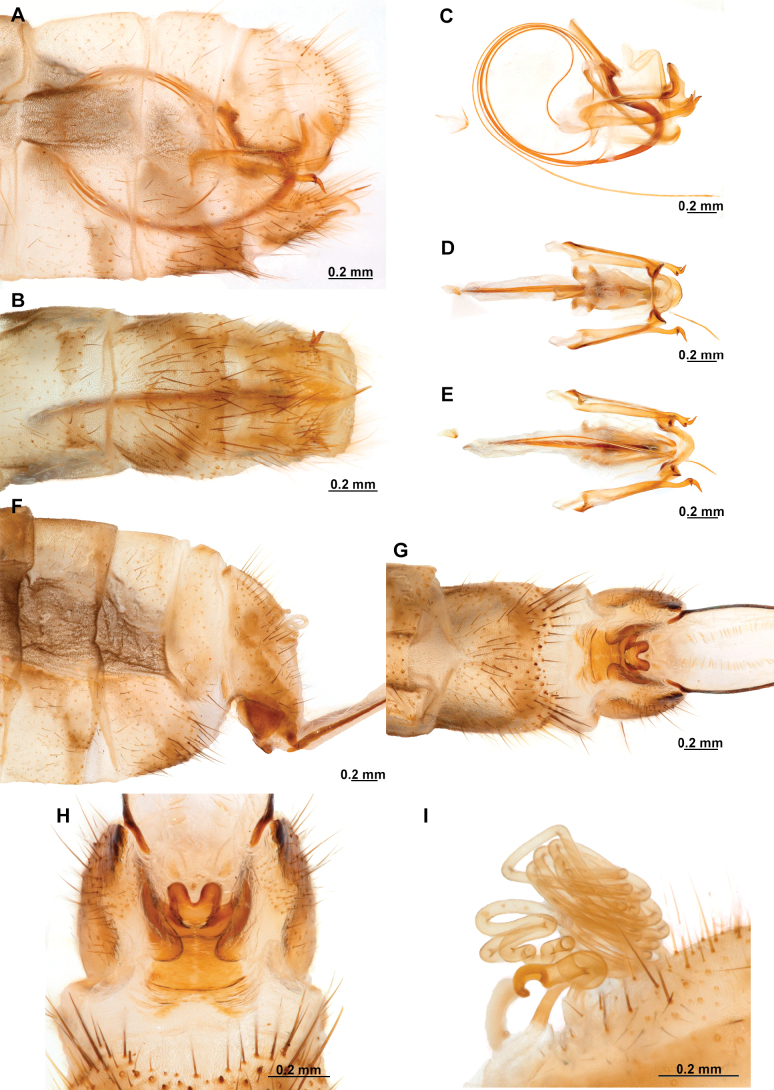
*Plegayucatanae* Parker & Stange, 1965 **A** male terminalia, lateral **B** same, ventral **C** male genitalia, lateral **D** same, dorsal **E** same, ventral **F** female terminalia, lateral **G** same, ventral **H** gonapophyses VIII ventral **I** spermatheca.

***Male genitalia*** (Fig. 76A–E). Tergite IX narrower medially than laterally, lateral margin rounded. Sternite VIII rectangular with posteromedial blunt process; sternite IX trapezoidal ventral view with posteromedial elongate, U-shaped process which is dorsally canaliculated; entire surface of sternite IX covered with abundant fine and long setae; in lateral view triangular, the apex reaching posterior margin of ectoproct. Gonocoxites IX thickened, short, straight; base connected to gonocoxites XI with a membrane; apex, generally with two digitiform processes lateral- or ventrally curved, of which one is slightly longer, a third smaller process is sometimes present. Ectoproct ovoid, covered with abundant, short, thin setae; ventral surface with anterior rounded lobe, which is continuous with ventromedial sclerotized, curved canal. Gonocoxites X forming a short, straight, ventrally canaliculate sclerite, whose posterior apex has two dorsal and two ventrolateral, short processes; gonostyli X with thickened, obtuse base, with two lateral processes at base, the rest of the structure, whip-shaped, ventrally curved, and anteriorly coiled, forming two loops before protruding from abdomen. Gonapophyses X as two thin, rod-shaped, straight sclerites whose posterior apex is dorsally curved; gonapophyses X arranged in a V-shaped structure, joined by a membrane which is medially sclerotized and covers the gonostyli X base. Gonocoxites XI U-shaped, medial lobe complex and elaborated, with two differentiated parts: a dorsal rounded lobe, and a ventral curved, hook-shaped process; between these parts a semicircular, less sclerotized, hyaline area is present; ventral margin V-shaped; lateral arms of gonocoxites XI short, straight, with anterior apex bent ventrad. Hypandrium internum triangular.

***Female genitalia*** (Fig. 76F–I). Sternum VII rectangular, with posterior margin concave, with thickened, long setae near posterior margin. Tergite VIII slightly narrower medially than laterally, encircling the spiracle of the segment, lateral margin triangular. Gonocoxites VIII as an oval, concave plate, whit V-shaped anterior margin. Gonapophyses VIII as two medially joined plates forming a boat-shaped structure with posteromedian thickened, bilobed process; median part of gonapophyses VIII connected laterally to triangular, concave plates, which are folded beneath tergite IX + ectoproct. Tergite IX + ectoproct narrowly ovoid. Gonocoxites IX elongated, narrow, as long as the last five abdominal segments together. Bursa copulatrix short, siphon-shaped, semimembranous, striated, with sclerotized proximal portion. Spermatheca complex and entangled; proximal and medial sections long thin, extremely coiled; distal section slightly wider than proximal and medial sections, diverticulum absent; fertilization canal duct with proximal portion triangular, concave; distal portion thin; fertilization canal pod-shaped, covered with microfilaments.

##### Distribution.

Guatemala (El Progreso, Zacapa), Honduras (El Paraíso, Francisco Morazán, Ocotepeque), Mexico (Chiapas, Jalisco, Yucatán).

##### Remarks.

This species is distributed in Central America and Southern Mexico. Since its original description, *P.yucatanae* was known only from the Mexican states of Chiapas and Yucatán (Oswald et al. 2002; Reynoso-Velasco and Contreras-Ramos 2008), here the first record from Jalisco is provided. Furthermore, the records from Guatemala and Honduras are the first for these countries. In the phylogeny of Symphrasinae, this species was recovered as sister of a clade mostly composed of South American species. This species has a striking coloration pattern and can be easily distinguished based on the markings of the head, pronotum and forefemur. This species is unique within the genus, presenting a tubercle-like process posteromedially on the sternite VIII. The overall structure of the male gonostyli X resembles that of *P.hagenella*, but the median lobe of the gonocoxites XI is markedly distinct from other species of the genus. The spermatheca is interesting as it is similar to that of *P.hagenella*, thus confirming its phylogenetic affinities.

#### ﻿﻿ Plega zikani

Taxon classificationAnimaliaNeuropteraRhachiberothidae

Navás, 1936

Figs 77 , 78

Plega zikani Navás, 1936: 722. Holotype: female, Brazil, Rio de Janeiro (SDEI), specimen examined. Ohl (2004): 148.
Trichoscelia zikani (Navás, 1936). Penny (1982a): 438.

##### Material examined.

***Holotype***. Brazil • ♀; **Rio de Janeiro**, Itatiaya Berg.; 11 Apr. 1929; Zikan leg.; no. 26; “*Plegazikani* ♀ Nav., P. Navás S.J. det.”, “Syntypus”, “*Plegazikani* Navás det. R. Hall (HNRS) 1984”, “DEI Münchberg Lep-00409”; SDEI.

##### Diagnosis.

This species has the antennal flagellum dark reddish brown with eight pale, preapical flagellomeres; the proximal flagellomeres are as long as wide. The femur is brown, with pale and dark areas. The pterostigma of both wings is dark reddish brown, with small, yellowish area. On the hind wing, the first branch of the CuA is trident-shaped. On the female genitalia, the gonapophyses VIII medial part is keel-shaped, posteromedially set with a dorsally bent, bilobed, process. The spermatheca has the proximal section zigzagged, and the distal section lacks a diverticulum.

##### Description.

***Measurements*.** Female (*n* = 1): Forewing length: 14.5 mm; Hind wing length: 11.2 mm.

**Figure 77.**
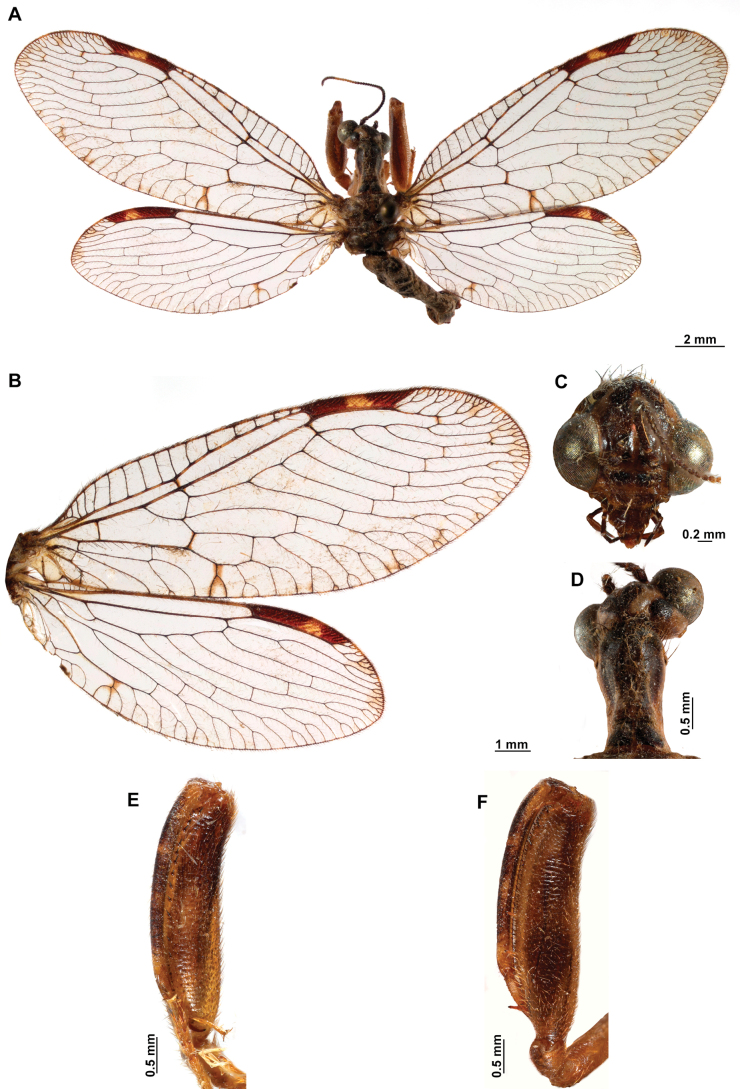
*Plegazikani* Navás, 1936 **A** female holotype habitus, dorsal **B** wings **C** head, frontal **D** pronotum, dorsal **E** forefemur, anterior surface **F** same, posterior surface.

***Coloration* (Fig. 77)**. ***Head*.** Vertexal region pale brown, with lateral dark brown marks extending from occiput to supraantennal area forming an inverted V-shaped pattern, with brown setae; supraantennal pale brown with lateral dark brown marks; occiput pale brown with dark mark adjacent to occipital ridge; postgena pale brown. Antennal scape and pedicel dark reddish brown, flagellum dark reddish brown with eight preapical pale flagellomeres. Frons pale brown with dark reddish-brown mark, clypeus mostly dark reddish brown; labrum brown; mandible dark reddish brown; maxilla mostly pale brown, palpus dark reddish brown; labium mostly pale brown, labial with first palpomere pale brown, second and third dark reddish brown, palpimacula brown. ***Thorax*.** Pronotum dark brown with pale brown lateral margins; episternum brown, postfurcasternum pale. Mesonotum mostly brown, with pale areas, setation brown; metanotum dark brown; pre-episternum brown; pteropleura brown with pale area on area adjacent to anapleural cleft. ***Foreleg*.** Coxa brown, with pale brown setae; trochanter brown with dark areas. Femur brown, with pale and dark areas. Tibia pale brown with broadened, darker bands, with pale brown setae. Basitarsus with pale, proximal area, base, and lanceolate process pale amber, clavate setae pale brown; remaining tarsomeres pale brown. ***Mid- and hind leg*.** Mid-leg brown with darker suffusions on trochanter, femur, and tibia. Hind leg pale brown with darker areas on apex of femur, and proximal ⅓ of femur. ***Wings*.** Forewing hyaline; membrane surrounding first branch of CuA and crossveins of the subcostal, radial, and mediocubital fields amber; posterior and apical margins on distal ½ of wing with intermittent, small, amber areas between apical branches of longitudinal veins; pterostigma dark reddish brown, with small, yellowish, medial area; longitudinal veins mostly dark brown, Sc, RA, and CuA alternating dark brown and yellowish; crossveins and subcostal veinlets dark brown; wing margin alternating yellow and dark reddish brown. Hind wing hyaline, with amber surrounding base of first branch of CuA, and wing apex; pterostigma dark reddish brown with small, yellowish, preapical area; most of longitudinal veins dark brown, C pale brown, C + Sc alternating pale and dark brown; subcostal veinlets pale brown, crossveins dark brown, except sigmoid 1r-m, mostly pale brown; wing margin alternating pale brown and dark reddish brown. ***Abdomen*.** Cleared.

***Morphology* (Fig. 77)**. ***Head*.** Diamond-shaped in frontal view, smooth; vertexal region raised above compound eyes, with lateral rows of fine, reclined setae; area surrounding coronal suture glabrous, with muscle insertion mark; coronal suture distinct; medial area of ​​supra-antennal moderately raised, with fine, reclined setae; paraocular area concave. Antenna submoniliform, short; scape 1.5× as long as wide, slightly distally expanded, with fine, short setae; pedicel as long as wide; flagellum not dorsoventrally flattened, with 44 flagellomeres, which are as wide as long; all articles with medial ring of fine, short setae. Compound eye hemispherical, as wide as ½ of the interocular distance at torulus level. Frons and clypeus narrow, with fine and short setae. Labrum pentagonal with thin, short setae; maxillary palpus with first palpomere as long as wide, second two times as long as wide, third palpomere 4× as long as wide, fourth palpomere 3× as long as wide, fifth as long as third, all with minute setae; mentum with long, thin setae; labial palpus with first palpomere two times as long as wide, second palpomere 4× as long as wide, third palpomere slightly longer than second, palpimacula narrowly ovoid. ***Thorax*.** Pronotum slightly longer than wide, with raised anterior margin, medial and posterior regions; outgrowths covered with pedicellate, thick setae; remaining surface with fine, short setae. Mesonotum nearly as long as wide, with scattered, thick, pedicellate setae on medial area. Metanotum ~ 2× as long as wide, nearly glabrous. Pteropleura with short, thin setae. ***Foreleg*.** Coxa as long as femur, cylindrical, anterior and posterior surfaces with fine setae of different lengths; trochanter trapezoidal, with thin and short setae, except on dorsal and anterior surfaces with some long setae; anterior surface with protuberant area. Femur robust, elongate, narrow, covered with abundant, fine, short setae arising from protuberant bases; closing surface with posteroventral row of processes composed two medially located, primary processes; proximal portion of the row with two tertiary processes; the rest of the row with numerous tubercle-shaped processes, and stinger-shaped setae; distal portion raised, composed of tubercle-shaped specializations and stinger-shaped setae; adjacent row of thickened setae with globular base present on distal ¼. Anteroventral row of processes reduced to proximal ½; it is composed of tubercle-shaped specializations and stinger-shaped setae; the basal-most primary, curved process is present; distal portion composed of a few tubercle-shaped processes; adjacent row of thickened setae with globular base present on distal ⅔. Tibia almost as long as femur, curved, with thin, short setae; ventral surface keeled with prostrate setae; a patch of clavate setae apically on anterior surface is present. Basitarsus with lanceolate process reaching the middle of fourth tarsomere; clavate setae present proximally on anterior surface; ventrally with single row of prostrate setae; second tarsomere nearly 7× as long as wide; third tarsomere 1.2× as long as wide, fourth tarsomere 2.5× as long as wide. ***Mid- and hind leg*.** Coxa and trochanter of both legs with short, thin setae; femur and tibia of midleg with thin setae of different lengths; tibial spurs short; femur and tibia of hind leg with short and thin setae. Hind leg longer than midleg, tibia, 1.5× as long as femur; tarsi with fine and short setae, except on distal margin of plantar surface with lateral groups of 3–5 thickened setae; on both legs, basitarsus 4× as long as wide, second tarsomere 1.2 × as long as wide; third and fourth tarsomeres as long as wide; fifth tarsomere two times as long as wide. ***Wings*.** Forewing oval, trichosors present along margin except on wing base; venation setose; costal space proximally expanded, humeral vein branched, 13 or 14 subcostal veinlets; pterostigma elongated, narrow, trapezoidal, with incomplete veinlets; subcostal space with single crossvein, medially located; Sc vein abruptly posteriorly bent at proximal pterostigma margin to merge with RA; radial space with two crossveins; *rarp2* gently curved with three RP branches; four veins arising from *rarp1*; M vein basally fused with RA; RP base not widely separated from divergence of M and R; M forked nearly opposite to RP origin, 1 r-m connecting RP base and MA stem, forming a trapezoidal cell; five gradate crossveins present. Cubitus deeply forked; CuP basally angled and approaching A1, distally forked opposite to the level of separation of M and R; A1 simple, ending on posterior margin at level of CuP fork, A2 forked opposite to CuP angle level. Hind wing smaller and narrower than forewing, narrowly oval; costal space narrow and reduced, with five veinlets; C and Sc fused at ¼ of wing length, Sc vein abruptly curved posteriorly at proximal margin of pterostigma to merge RA; pterostigma elongated, narrow, straight, composed of well-defined, entire veinlets; radial space with single crossvein, oblique; four veins arising from *rarp1*, one or two from *rarp2*. 1r-m sigmoid, connecting the stems of M and RP. Media forked slightly beyond R fork. Cubitus deeply forked, intracubital crossvein subparallel to longitudinal wing axis; CuA sinuous, first branch candelabrum-shaped, spur vein absent; CuP not touching A1, strongly anteriorly bent at distal 1/3, pectinate; single crossvein on cubitoanal space; A1 simple, ending on wing margin at 1r-m stem level; A2 simple, short, and curved. ***Abdomen*.** Subcylindrical, medially expanded, tergites subquadrate, setae on tergites, scattered, thin, and short. Sternites rectangular, with scattered fine and short setae.

**Figure 78.**
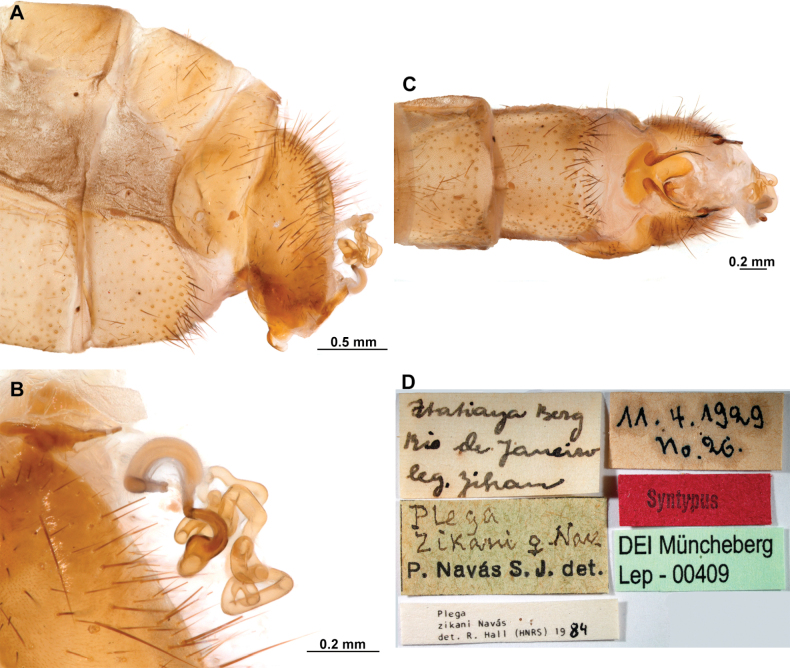
*Plegazikani* Navás, 1936 **A** female terminalia, lateral **B** same, ventral **C** spermatheca **D** holotype labels.

***Female genitalia*** (Fig. 78). Sternum VII rectangular with posterior margin concave. Tergite VIII slightly narrower medially than laterally, encircling the spiracle of the segment; lateral margin D-shaped. Gonocoxites VIII as an oval concave sclerite. Gonapophyses VIII with medial part as two medially fused plates forming a keeled structure with posteromedial, dorsally bent, bilobed, process; lateral part as an oval, concave plate folded beneath IX + ectoproct. Tergite IX + ectoproct narrowly ovoid. Gonocoxites IX elongated and narrow. Bursa copulatrix elongate, funnel-shaped, membranous, and striated. Spermatheca long, entangled; proximal section, thin, zigzagged; medial section as wide as proximal section, forming several convolutions; distal section slightly distally expanded, diverticulum absent; fertilization canal duct strongly curved, progressively narrowed; fertilization canal C-shaped, covered with microfilaments.

##### Distribution.

Brazil (Rio de Janeiro).

##### Remarks.

This species is known only from the female holotype which was collected in the state of Rio de Janeiro in Brazil. Despite that Navás (1936) described this species under the genus *Plega*, Penny (1982a) wrongly treated this species under the genus *Trichoscelia* without having seen the holotype of *P.zikani*. Then, Penny and da Costa (1983) provided a redescription for the species, although they were unaware that were actually redescribing *T.varia*. After that study, confusion arose about the identity of *T.zikani*, which was separated from *T.varia* by Penny (1982a) and Penny and da Costa (1983) based on the extensive pigmentation on the apex of both wings, although at the same time mentioned the former species could be a dark form of the latter as both shared a similar morphology of the male gonocoxites IX. In the world catalog of Mantispidae by Ohl (2004), the generic combination of the name *zikani* was revised based on the examination of the holotype, and it was corroborated as belonging to the genus *Plega*, as originally proposed by Navás (1936). In a recent publication by Silva et al. (2016), *Plegazikani* was recorded from Minas Gerais, Brazil, although the authors misidentified a dark specimen of *T.varia*, following the redescription of Penny and da Costa (1983). Here, the confusing identity of this species is further clarified based on the examination and redescription of the holotype of *P.zikani*.

In the phylogeny of the subfamily, *P.zikani* was recovered as sister to the rest of species of *Plega*, although unfortunately the male is still unknown. This species has a narrow forefemur, a distinctive coloration of the pterostigma and a simple and short spermatheca lacking diverticulae.

#### ﻿﻿Genus Trichoscelia

Taxon classificationAnimaliaNeuropteraRhachiberothidae

Westwood, 1852

Trichoscelia Westwood, 1852: 270. Type species: Mantispa (Trichoscelia) fenella Westwood, 1852: 269, subsequent designation by Enderlein (1910): 376. Trichoscelia was originally proposed as a subgenus of Mantispa Illiger in Kugelann.
Trichoscelis ; Hagen, 1861: 323. Incorrect spelling of Trichoscelia.
Symphrasis Hagen, 1877: 208. Type species: Raphidiavaria Walker, 1853: 212 (as “S.varia Walker, 1853”), subsequent designation by Enderlein (1910): 374. Symphrasis was considered as a synonym of Trichoscelia by Gerstaecker (1888): 117, Parker and Stange (1965): 605 and Penny (1982b): 214.
Symphasis ; Banks, 1903: 329. Incorrect spelling of Symphrasis.

##### Further description.

Brauer (1887): 214; Enderlein (1910): 374 (as *Symphrasis*); Tjeder (1959): 275, figs 244–253 (wings; mouth parts; foreleg; female terminalia of *T.varia*); Parker and Stange (1965): 605; Penny (1982a, b): 426; Penny and da Costa (1983): 622; Makarkin (2015): 339–340, fig. 8c (drawing of foretarsus).

##### Taxonomy.

Gerstaecker (1888): 117 (as *Anisoptera*; *Symphrasis* = *Plega*); Enderlein (1910): 376 (*Anchieta* = *Trichoscelia*); Navás (1912): 201 (*Anchieta* not = *Trichoscelia*); Tjeder (1959): 276 (*Symphrasis* = *Plega*); Tjeder (1968): 16–17 (*Symphrasis* = *Plega*); Parker and Stange (1965): 605 (*Symphrasis* = *Trichoscelia*).

##### Key to species.

Banks (1913): 207–208 (species described by Westwood); Penny (1982a): 427 (Brazilian Amazonia); Penny and da Costa (1983): 622–623 (Brazil); Machado (2018): 190 (South America).

##### Lists of species.

Walker (1853): 117 (as *Mantispa*); Hagen (1866): 426, 461; Brauer (1887): 213–214; Penny (1977): 37; Machado (2018): 191.

##### Diagnosis.

This genus differs from the other Symphrasinae by having a slightly domed vertexal region above compound eyes; compound eyes are as wide as ¾ of the interocular distance at toruli level. The labial palpus has the third palpomere generally remarkably expanded. The pronotum is generally narrow, 1.5× as long as wide. On the forefemur, the closing surface presents both rows of processes fully developed, the specializations are tubercle-shaped, with a more developed sub-basal process on each row; primary process of the anteroventral row of processes is absent; the rows of thickened setae with globular base adjacent to each row of processes extend over almost the entire closing surface on both sides. On the mid- and hind leg, the first four tarsomeres have a couple of thickened setae, laterally on the distal margin of the plantar surface. In the hind wing, the 1 r-m is distally connected to M stem by a short cross vein. The bursa copulatrix has the proximal region sclerotized and concave; the spermatheca generally has a trumpet-shaped invagination on apex of distal section, adjacent to the fertilization canal duct.

##### Description.

***Head*.** Semi-triangular or diamond-shaped in frontal view, vertexal region slightly domed above compound eyes, with short, reclined rows of setae; paraocular area strongly concave; coronal suture discrete or not discernible. Antenna moniliform, scape slightly longer to ~ 2× as long as wide; flagellomeres as wide as long or discoidal, narrower towards the flagellar apex, with medial ring of setae. Frons trapezoidal, narrow; labrum rounded. Compound eyes hemispheric, as wide as ¾ of the interocular distance at torulus level. Postocular region narrow. Labial palpus 3-articulated, third palpomere moderate- to markedly expanded. ***Thorax*.** Pronotum as long as wide or 1.5× as long as wide, with a groove contiguous to the lateral and distal margins; in lateral view anterior margin, medial region and posterior margin slightly raised, with or without thickened, pedicellate setae; postfurcasternum trapezoidal, paired. Mesonotum as or ~ 2× as wide as long, with abundant long and thickened setae on medial area, metanotum ~ 2× as wide as long, glabrous. Pteropleura setose. ***Foreleg*.** Coxa slightly shorter than femur, setose; trochanter subtrapezoidal; femur thickened, setose; closing surface with double row of tubercle-shaped processes, both rows with a more developed sub-basal process; rows of thickened setae with globular base adjacent to processes rows present, extending over almost the entire closing surface. Tibia almost as long as femur, curved, setose, closing surface keeled with prostrate, flattened setae; with a patch of clavate setae distally on anterior surface. Basitarsus elongated, ventrally keeled, with a row of prostrate, flattened setae on the proximal ½, anterior surface with a patch of clavate setae proximally; lanceolate process short, reaching the distal margin of third tarsomere, with a plug-shaped Stitz organ at the apex; second tarsomere 4× as long as wide. Pretarsus with two simple claws and an arolium. ***Mid- and hind leg*.** Tibiae thin to slightly thickened; basitarsus three to 4× as long as wide, the first four tarsomeres with a pair of thickened setae laterally on distal margin of plantar surface. ***Wings*.** Forewing oval, trichosors present along margin except at wing base; costal field narrow, humeral vein simple, forked or branched; pterostigma elongated and narrow; subcostal space with single, medially located crossvein; Sc vein bent posteriorly at the proximal margin of pterostigma to merge with the RA; *rarp2* curved; M basally fused to R; RP base located near separation of M and R, M fork near such separation, opposite to R fork; 1r-m located between RP base and MA base, forming a small trapezoidal cell; single gradate series present. Cubitus deeply forked, CuA ending on the margin posterior at or slightly before the level of 1ra-rp; CuP basally angulated, approaching A1, distally forked. A1 simple, reaching the level of separation of M and R; A2 distally forked, at the level of the CuP angle. Hind wing smaller and narrower than forewing; Sc and C fused at 1/5 of wing length; Sc diverging from C at proximal pterostigma margin, curving posteriorly to merge RA; pterostigma elongated, narrow, curved; radial space with single, oblique crossvein. Media forked beyond R fork; 1 r-m sigmoid, connecting M base and RP base, distally connected again to M stem through a short crossvein. Cubitus deeply forked, CuA long and sinuous, approaching the level of 1ra-rp, first branch candelabrum-shaped, situated beyond the level of M-fork; CuP distally, anteriorly curved, pectinate, ending at posterior margin slightly beyond M fork. Cubitoanal space with two crossveins. A1 arched, A2 short and arched. ***Abdomen*.** Subcylindrical without apparent modifications.

***Male genitalia*.** Tergite IX generally medially narrower than laterally, lateral margin often with a posteroventral notch. Sternite IX semi-triangular to pentagonal, posteromedially generally forming an obtuse angle, with a dorsal canal; in lateral view approaching posterior margin of the ectoproct. Gonocoxite IX short to long, straight to curved, thin to thickened, base generally spatulate, connected to gonocoxites XI by membranes; apex with digitiform processes. Ectoproct ovoid to trapezoidal; ventral surface with an anterior bulging area, followed posteriorly by a concave area, both generally sclerotized. Gonocoxites X forming a generally short, straight, thickened, ventrally canaliculate sclerite; anterior apex sometimes expanded, posterior apex bilobed, with two preapical processes connected to gonapophyses X with a membrane. Gonostyli X with two lateral processes at base, the rest whip-shaped, long, curved ventrally and coiled anteriorly, forming internal loops before protruding from the abdomen. Gonapophyses X rod-shaped, arranged in a V-shaped structure, joined by a membrane covering the base of gonostyli X. Gonocoxites XI U-shaped, with straight to sinuous lateral arms; median lobe expanded and elaborate, with two distinct parts: a dorsal lobe, and a convex ventral part, whose ventral margin generally incised; between both parts an unsclerotized region present.

***Female genitalia*.** Sternite VII rectangular to trapezoidal. Tergite VIII enclosing the spiracle of eighth abdominal segment. Membrane between tergite VIII and tergite IX + ectoproct sclerotized, forming a concave triangular plate. Gonocoxites VIII forming a trapezoidal plate, sometimes with two lateral concave areas where the lateral plates of gonapophyses VIII and tergite IX + ectoproct rest; gonapophyses VIII with medial part composed of two trapezoidal plates medially joined forming a keel, posteromedially a bilobed process is present; laterally medial plates are connected to convex plates which in turns are connected to tergite IX + ectoproct. Tergite IX and ectoproct fused. Gonocoxite IX elongated and narrow. Bursa copulatrix short funnel-shaped, proximal region sclerotized, concave, the rest membranous; spermatheca complexly entangled, long, proximal section thin, medial section sometimes thicker than proximal section, complexly entangled; distal section progressively broadened towards the apex, distally with a trumpet-shaped invagination adjacent to fertilization canal duct. Fertilization canal duct short, thin, sigmoid; fertilization canal short, thickened, J- or pod-shaped, covered with microfilaments.

##### Included species.

1. *T.anae* Penny, 1982 (Brazil, Peru)

2. *T.andina* Ardila-Camacho, 2015 (Colombia, Ecuador, Panama, Venezuela)

3. *T.banksi* Enderlein, 1910 (Costa Rica, Guatemala, Honduras, Mexico, Nicaragua)

=*T.santareni* (Navás, 1914), new synonym

4. *T.basella* (Westwood, 1867) (Bolivia, Brazil)

5. *T.fenella* (Westwood, 1852) (Brazil, Colombia, French Guiana, Suriname)

= *T.egella* (Westwood, 1867), new synonym

=*Anisopteraamoenula*Gerstaecker 1888

6. *T.flavomaculata* Ardila-Camacho & Contreras-Ramos, sp. nov. (Costa Rica, Panama)

7. *T.geraldoi* Machado, 2018 (Brazil, French Guiana)

8. *T.gorgonensis* Ardila-Camacho, 2015 (Colombia)

9. *T.involuta* Ardila-Camacho & Contreras-Ramos, sp. nov. (Brazil, Colombia)

10. *T.iridella* (Westwood, 1867) (Brazil)

11. *T.karijona* Ardila-Camacho, 2015 (Colombia, French Guiana, Guyana)

12. *T.larizae* Ardila-Camacho & Contreras-Ramos, sp. nov. (Panama)

13. *T.latifascia* McLachlan, 1867 (Brazil, Ecuador, Peru)

=*Anisopterajocosa*Gerstaecker 1888

=*Symphrasisthaumasta*Navás 1915

14. *T.nassonovi* (Navás, 1912) (Bolivia, Brazil, Ecuador, French Guiana, Peru, Trinidad and Tobago)

= *T.trifasciata* (Stitz, 1913), new synonym

15. *T.pennyi* Ardila-Camacho & Contreras-Ramos, sp. nov. (Colombia, Ecuador)

16. *T.sequella* (Westwood, 1867) (Brazil, Colombia)

17. *T.tobari* (Navás, 1914) (Mexico)

18. *T.umbrata* Ardila-Camacho, 2018 (Colombia)

19. *T.varia* (Walker, 1853) (Argentina, Bolivia, Brazil, Uruguay)

=*Mantispamyrapetrella*Westwood 1867

20. *T.vespiformis* Ardila-Camacho & Contreras-Ramos, sp. nov. (Brazil)

21. *T.wintertoni* Ardila-Camacho & Contreras-Ramos, sp. nov. (Brazil)

##### Biology.

Summarized in Ardila-Camacho et al. (2021b).

##### Etymology.

The generic name was proposed by Westwood (1852) in reference to the setose condition of the hind tibia.

### ﻿﻿Key to the species of *Trichoscelia*

**Table:** 

1	Costal space proximally noticeably expanded (Fig. 79B); antennal flagellum with four pale, preapical flagellomeres (Fig. 79C)	***Trichosceliaanae* Penny, 1982**
–	Costal space narrow with C and Sc veins subparallel-sided (Fig. 81B); antennal flagellum completely dark or with yellowish apex	**2**
2	Body color pattern mostly orange; forewing patterned, with amber bands and/or spots; pterostigma with single color or transversely divided into two colors	**3**
–	Body color pattern with a mixture of yellow and brown or mostly blackish brown; forewing mostly hyaline or with amber areas surrounding crossveins and apical forks of longitudinal veins; pterostigma brown with pale medial area	**4**
3	Forefemur posterior surface completely orange (Fig. 87F); forewing with an amber spot on the area adjacent to proximal margin of pterostigma and 1ra-rp, a second spot on the MP fork may be present (Fig. 87B); male gonocoxite IX with six or seven digitiform processes, arranged as a brush (Fig. 88C–E)	***Trichosceliafenella* (Westwood, 1852)**
–	Forefemur posterior surface orange with dark brown base (Fig. 101F); forewing with a broad, amber, transverse band at the pterostigma level, two amber spots near anterior and posterior wing margins on proximal 1/3 of wing length (Fig. 101B); male gonocoxite IX with four or five apical processes, an additional longer, preapical process is also present (Fig. 102C–E	***Trichoscelialatifascia* McLachlan, 1867**
4	Body coloration pattern mostly blackish brown (Fig. 115A); basal abdominal segments cream colored	**5**
–	Body coloration pattern with a mixture of yellow and brown (Fig. 81A); basal abdominal segments with different color	**6**
5	Male sternite IX pentagonal in ventral view (Fig. 92B); male gonocoxite IX equipped with five or six digitiform processes arranged as a hand (Fig. 92C–E); female gonocoxites VIII forming a trapezoidal plate, with two lateral concavities in ventral view (Fig. 92H)	***Trichosceliageraldoi* Machado, 2018**
–	Male sternite IX semi-triangular in ventral view (Fig. 116B); male gonocoxite IX equipped with 10 or 11 digitiform processes arranged in a cluster (Fig. 116C–E); female gonocoxites VIII not discernible (Fig. 116H)	***Trichosceliavespiformis* Ardila-Camacho & Contreras-Ramos, sp. nov.**
6	Basal antennal flagellomeres as wide as long (Fig. 113C); wings often patterned with faint to well-marked brown maculation surrounding crossveins, forks of longitudinal veins, and wing tips (Fig. 113B); forefemur remarkably narrow (Fig. 113F); male gonocoxite IX with two to four digitiform processes, of which, when present two are preapical, dorsally situated and widely separated (Fig. 114C–E)	***Trichosceliavaria* (Walker, 1853)**
–	Basal antennal flagellomeres 1.5–2.0 times as wide as long; wings mostly hyaline; forefemur narrow or noticeably robust; processes on male gonocoxites with different arrangement	**7**
7	Forefemur noticeably robust (Fig. 117F); wing venation mostly yellow on medial area of C, Sc, RA and on proximal ½ of RP branches (Fig. 117B); male gonocoxite IX with three apical digitiform processes, two lateral and long, and one medially located, and shorter (Fig. 118C–E)	***Trichosceliawintertoni* Ardila-Camacho & Contreras-Ramos, sp. nov.**
–	Forefemur not noticeably robust; wing venation alternating brown and yellow or mostly brown; processes of male gonocoxite IX with different arrangement	**8**
8	Male gonocoxite IX with four digitiform processes two apical, medially situated, and two preapical, laterally located (Fig. 100C–E)	**9**
–	Male gonocoxite IX processes with different arrangement	**12**
9	Gonostyli X strongly coiled, forming several convolutions (Fig. 94C)	**10**
–	Gonostyli X not strongly coiled, forming one or two convolutions (Fig. 112C)	**11**
10	Forefemur entirely yellow (Fig. 99E, F); male gonocoxite IX short and straight (Fig. 100C–E)	***Trichoscelialarizae* Ardila-Camacho & Contreras-Ramos, sp. nov.**
–	Forefemur yellow with honeycomb-shaped brown marks (Fig. 93E, F); male gonocoxite IX elongate and curved (Fig. 94C–E)	***Trichosceliainvoluta* Ardila-Camacho & Contreras-Ramos, sp. nov.**
11	Antennal flagellum dark with yellowish apex (Fig. 109D); posterior surface of forefemur with a broad, brown band on the area adjacent to closing surface (Fig. 109F); forewing with faint areas surrounding crossveins and forks of longitudinal veins (Fig. 109B)	***Trichosceliatobari* (Navás, 1914)**
–	Antennal flagellum completely dark (Fig. 111A); posterior surface of forefemur yellow, with a narrow, brown, medial mark (Fig. 111F); forewing with shaded intermittent, shaded areas adjacent to posterior wing margin (Fig. 111B)	***Trichosceliaumbrata* Ardila-Camacho, 2018**
12	Male gonocoxite IX bifid (a third short process may be present)	**13**
–	Male gonocoxite IX processes with different arrangement	**14**
13	Forewing hyaline (Fig. 97B); male gonocoxite IX deeply forked, with long preapical process (Fig. 98C–E)	***Trichosceliakarijona* Ardila-Camacho, 2015**
–	Forewing with faint amber areas adjacent to crossveins and forks of longitudinal veins (Fig. 103A); male gonocoxite IX apically bifid, with short preapical process (Fig. 104C–E)	***Trichoscelianassonovi* (Navás, 1912)**
14	Male gonocoxites IX noticeably short (as long as the lateral arms of the gonocoxite XI) (Fig. 108C–E)	**15**
–	Male gonocoxites IX elongate (longer than lateral arms of gonocoxites XI) (Fig. 96C–E)	**17**
15	Wing venation and pterostigma completely dark brown (Fig. 107B); male gonocoxite IX with three or four subequal, digitiform processes, hand-like arranged (Fig. 108C–E)	***Trichosceliasequella* (Westwood, 1867)**
–	Wing venation alternating dark brown and yellow, pterostigma brown with medial, yellow area (Fig. 83B); male gonocoxite IX with three digitiform, recurved processes (Fig. 84C–E)	**16**
16	Pronotum yellow with dark brown medial band (Fig. 83D); male gonocoxite IX with two apical, subequal and laterally curved processes and a third longer, preapical being twisted or recurved laterally (Fig. 84C–E)	***Trichosceliabanksi* Enderlein, 1910**
–	Pronotum yellow with a dark brown Y-shaped, inverted mark (Fig. 105D); male gonocoxite IX with the three processes apically located and dorsally recurved (Fig. 106C–E)	***Trichosceliapennyi* Ardila-Camacho & Contreras-Ramos, sp. nov.**
17	Male gonocoxite IX equipped with 11 or 12 processes arranged in a cluster (Fig. 96C–E); dorsal part of the gonocoxites XI medial lobe anteriorly curved and acuminate in dorsal view (Fig. 96D)	***Trichosceliairidella* (Westwood, 1867)**
–	Male gonocoxite IX equipped with 5–8 short processes; dorsal part of the gonocoxites XI medial lobe straight or concave in dorsal view (Fig. 82D, 86D)	**18**
18	Both wings with most of the CuA, CuP, and anal veins yellow, with the membrane between them yellow (Ardila-Camacho and García 2015, fig. 11); male sternite IX with rounded lateral margins (Ardila-Camacho and García 2015, fig. 13a)	***Trichosceliagorgonensis* Ardila-Camacho, 2015**
–	Both wings with CuA, CuP, and anal veins alternating brown and yellow, with the membrane between them hyaline; male sternite IX with lateral margins angulate or straight in ventral view (Fig. 82B)	**19**
19	Median lobe of male gonocoxites XI with ventral part forming a prominent, trapezoidal process with blunt lateroventral outgrowths (Fig. 82C–E)	***Trichosceliaandina* Ardila-Camacho, 2015**
–	Median lobe of male gonocoxites XI with ventral part convex (Fig. 85C)	**20**
20	Forewing pterostigma with small medial, yellow area (Fig. 89B); male gonocoxite IX gently curved in lateral view, with straight base, apex with six or seven short processes (Fig. 90C–E)	***Trichosceliaflavomaculata* Ardila-Camacho & Contreras-Ramos, sp. nov.**
–	Forewing pterostigma with wide, orangish medial area (Fig. 85B); male gonocoxite IX strongly curved in lateral view with base dorsally curved inwards, apex with five short processes (Fig. 86C–E)	***Trichosceliabasella* (Westwood, 1867)**

#### ﻿﻿ Trichoscelia anae

Taxon classificationAnimaliaNeuropteraRhachiberothidae

Penny, 1982

Figs 79 , 80

Trichoscelia anae Penny, 1982a: 427. Holotype: male, Brazil, Rondônia (INPA), photographs examined.

##### Material examined.

***Holotype***. Brazil • ♂; **Rondônia**, 364, Km 50; 01 Nov. 1979; J. Arias leg.; armadilha de malaise, 6 m; Neurop 065; Holotipo *Trichosceliaanae* Penny; INPA.

##### Other material.

PERU – **Madre de Dios** • 2 ♂; Río Tambopata Res., 30 Km (air) S.W. Pto. Maldonado; 290 m; 12°50'S, 69°20'W; 31 Oct. 1982; Canopy Fogging Project, La Torre trail Pt. Secondary floodplain forest, fogged tree #73; FSCA. • 1 ♀; **San Martín**, Moyabamba, vic. Ecológico “Rumipata”; 06°04'32"S, 76°58'7.5"W; 970 m; 13–18 Oct. 2012; J.E. Eger leg.; MV and UV light; FSCA.

##### Diagnosis.

This species is easily distinguished from all its congeners because the wings are distinctively expanded and broadly oval, with the costal field expanded proximally. The antennal flagellum is dark brown with four yellowish preapical flagellomeres. On the male genitalia the gonocoxite IX is short, thin, and sinuous, with the posterior apex lateroventrally curved, possessing four long, subequal apical digitiform processes. The gonocoxites XI are V-shaped; the medial lobe has the dorsal part as a quadrangular lobe with thickened margins; the ventral part is composed of a caudally curved, acuminate process with thickened margins. The female genitalia have a short and conical invagination on the distal section of the spermatheca, and the fertilization canal is remarkably long and S-shaped.

##### Description.

***Measurements*.** Male (*n* = 2). Forewing length: 8.0–9.0 mm; Hind wing length: 6.3–7.4 mm. Female (*n* = 1): Forewing length: 9.3 mm; Hind wing length: 7.5 mm.

**Figure 79.**
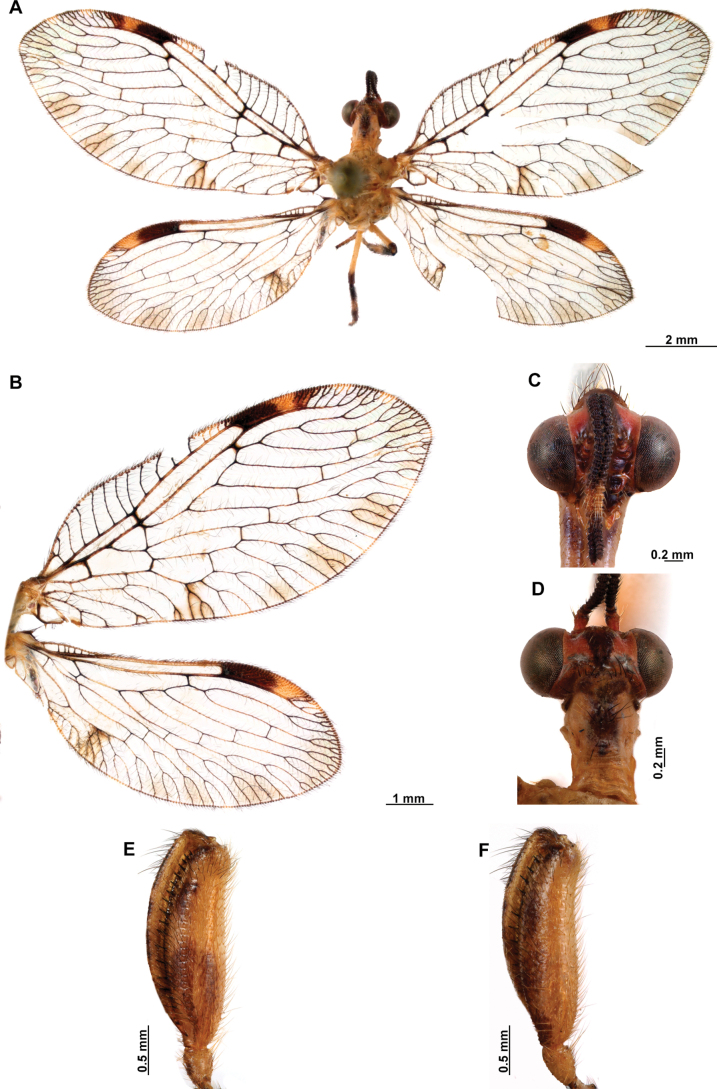
*Trichosceliaanae* Penny, 1982 **A** male habitus, dorsal (abdomen removed) **B** wings **C** head, frontal **D** pronotum, dorsal **E** forefemur, anterior surface **F** same, posterior surface.

***Coloration* (Fig. 79)**. ***Head*.** Mainly yellow with a longitudinal brown band extending from occiput to supraantennal region where it is forked. Vertexal region covered with dark brown setae on medial band. Antennal scape yellow, with distal margin infuscate; pedicel brown; flagellomeres dark brown, except 4–6 preapical yellow. Frons with two lateral, brown areas; clypeus brown with yellow margins, pale brown setae present. Labrum brown; mandible brown; maxilla pale brown with dark areas, palpus brown. Labium yellow, palpus brown with pale and dark areas, palpimacula pale brown. ***Thorax*.** Pronotum yellow with the medial area and the anterior margin brown, setae generally with same color as cuticle, on anterior margin interspersed pale and dark setae present; episternum yellow; postfurcasternum yellow. Mesonotum yellow, with brown markings on central area of sclerites, setae on these markings dark brown; metanotum either completely yellow or with brown lateral markings on the scutum, plus medial mark on scutellum. Pre-episternum yellow with brown medial mark. Pteropleura yellow with brown markings on sclerites. ***Foreleg*.** Coxa yellow with brown marks, with interspersed yellow and brown setae; trochanter yellow with brown mark on anterior surface, interspersed yellow and brown setae present. Femur yellow, anterior surface with broad mark on proximal ½, plus smaller preapical mark; posterior surface with adjacent ½ to closing surface brown; setae mostly yellow except on adjacent ½ to ventral surface dark brown. Tibia yellow with discontinuous brown band on the dorsal surface, clavate setae yellow; tarsus brown. ***Mid- and hind leg*.** Mid-leg with coxa yellow with brown marks at base and apex, interspersed yellow and brown setae present; trochanter mainly brown, yellow at base and apex, interspersed yellow and brown setae present; femur yellow with brown apex, setae with same color as cuticle, except for some brown setae near the base; tibia yellow with brown base and fuscous apex, setae mainly brown; tarsus brown with pale areas, interspersed yellow and brown setae; plantar surface of first four tarsomeres distal, brown setae, laterally on each side. Hind leg similar to mid-leg, except trochanter completely yellow. ***Wings*.** Forewing hyaline, with intermittent smoky areas along posterior and distal margin; area adjacent to crossveins amber. Pterostigma, dark brown, with a yellow preapical area. Longitudinal veins and posterior and distal wing margin alternating yellow and dark brown; subcostal veinlets and crossveins mainly dark brown, except at the apex of the costal field, yellow; setae mainly dark brown, in some areas with same color as cuticle. Hind wing hyaline with smoky areas at the base of the subcostal field and along the posterior and distal wing margin; area adjacent to first CuA branch strongly marked. Pterostigma brown with small, preapical yellow area. Longitudinal veins alternating brown and yellow, Sc + C yellow; crossveins brown; setae mainly dark brown. ***Abdomen*.** Tergites yellow with brown mark adjacent to posterior margin; setae generally corresponding to cuticle color. Sternites yellow with setae mainly yellow. Pleural membrane yellow with intermittent brown areas.

***Morphology* (Fig. 79)**. ***Head*.** Semi-triangular in frontal view, moderately setose, smooth; vertexal region slightly domed above compound eyes, with rows of reclined setae; paraocular area strongly concave; coronal suture discrete. Compound eye hemispheric, large in relation to head size, as wide as ¾ interocular distance at toruli level. Antenna moniliform, scape slightly longer than wide, subconical; pedicel slightly longer than wide, both with thickened setae; flagellum slightly dorsoventrally compressed, with 29–33 flagellomeres; flagellomeres on basal ½ of flagellum 1.5× as wide as long, the rest almost as wide as long, progressively smaller towards the apex; all articles with medial row of thickened setae. Postgena narrow in lateral view; hypostomal bridge not completely fused to postgena. Maxillary palpus with first two palpomeres as long as wide, third palpomere 3× as long as wide, fourth ~ 2× as long as wide, fifth palpomere pointed, as long as third. Submentum oval with long setae; first palpomere 1.5× as long as wide, second 2× as long as wide, third palpomere slightly longer than second, proximally expanded; palpimacula ovoid. ***Thorax*.** Pronotum slightly longer than wide, with sulcus contiguous to lateral and anterior margins; in lateral view anterior margin, medial region and posterior margin raised, with pedicellate, thickened setae; the rest of the surface with fine and short setae; episternum with long fine setae; postfurcasternum trapezoidal. Mesonotum slightly wider than long, with thickened, pedicellate, erect setae in the medial area of the scutum and on scutellum. Metanotum ~ 2× as long as wide, mostly glabrous; scutellum with few short, thin setae. Pteropleura covered with abundant long, thin setae. ***Foreleg*.** Coxa slightly shorter than femur, cylindrical, with abundant long, thin setae; trochanter subconical, covered with long, thin setae, dorsal surface with thickened, pedicellate near distal margin; femur incrassate, with abundant short, thin setae; closing surface with double row of tubercle-shaped specializations, both rows with a more developed sub-basal process (process/ seta ratio 1:1); rows of thickened setae with globular base adjacent to each row of processes present, extending over almost the entire closure surface. Tibia almost as long as femur, curved, with abundant long, thin setae; with a patch of clavate setae apically on anterior surface; lanceolate process of basitarsus reaching the base of fourth tarsomere; second tarsomere 3× as long as wide, third tarsomere as long as wide, fourth tarsomere 2× as long as wide, with thickened setae. ***Mid- and hind leg*.** Mid-leg with coxa and trochanter covered with long, thin setae; tibia slightly longer than femur, with long, thickened setae, thinner and shorter on femur; tibial spurs short; basitarsus 3× as long as wide, second tarsomere slightly longer than wide, third and fourth as long as broad, fifth tarsomere 2× as long as wide, all covered with long, thin setae; first four tarsomeres with a pair of thickened setae laterally on distal margin of the plantar surface. Hind leg longer than mid-leg, densely pubescent, tibia 1.5× as long as femur, setae as in midleg; tarsomeres similar in shape and proportion to those of the midleg. ***Wings*.** Forewing broadly oval, trichosors present along wing margin except at base; costal space proximally widened, humeral vein forked; 12 or 13 subcostal veinlets present. Pterostigma elongated and narrow, composed of numerous veinlets, mainly simple and incomplete; subcostal space with a single, medially located crossvein. Sc vein bent posteriad at the proximal margin of pterostigma to merge RA; *rarp2* curved, with two or three veins arising from it, three from *rarp1*. M vein fused basally to R; base of RP located near separation of M and R, M fork near that separation, opposite to R fork; 1r-m located between RP base and MA base, forming a small, trapezoidal cell; four or five gradate crossveins present. Cu vein deeply forked; CuP basally angled, approaching A1, forked opposite to separation of M and R. A1 vein simple, reaching the level of separation of M and R, apically forked; A2 apically forked, at CuP angle level. Hind wing smaller and narrower than anterior; costal space narrow and reduced, with seven veinlets; Sc and C fused at proximal ¼ of wing length; Sc curved posteriad at proximal margin of pterostigma to merge RA. Pterostigma elongated, narrow, curved, composed of numerous incomplete veinlets; radial space with single crossvein; three veins arising from *rarp1*, one from *rarp2*. M vein forked beyond the R fork; 1 r-m sigmoid, connecting M base and RP base, distally connected again to M stem through short crossvein. Cu deeply forked, CuA long and sinuous, distally branched, first branch candelabrum-shaped; CuP with the distal portion anteriorly curved, pectinate. Cubitoanal space with two crossveins. A1 arched, A2 short and arched. ***Abdomen*.** Cylindrical to medially expanded; tergites quadrangular with scattered, thin setae. Sternites rectangular with scattered, short, thin setae.

**Figure 80.**
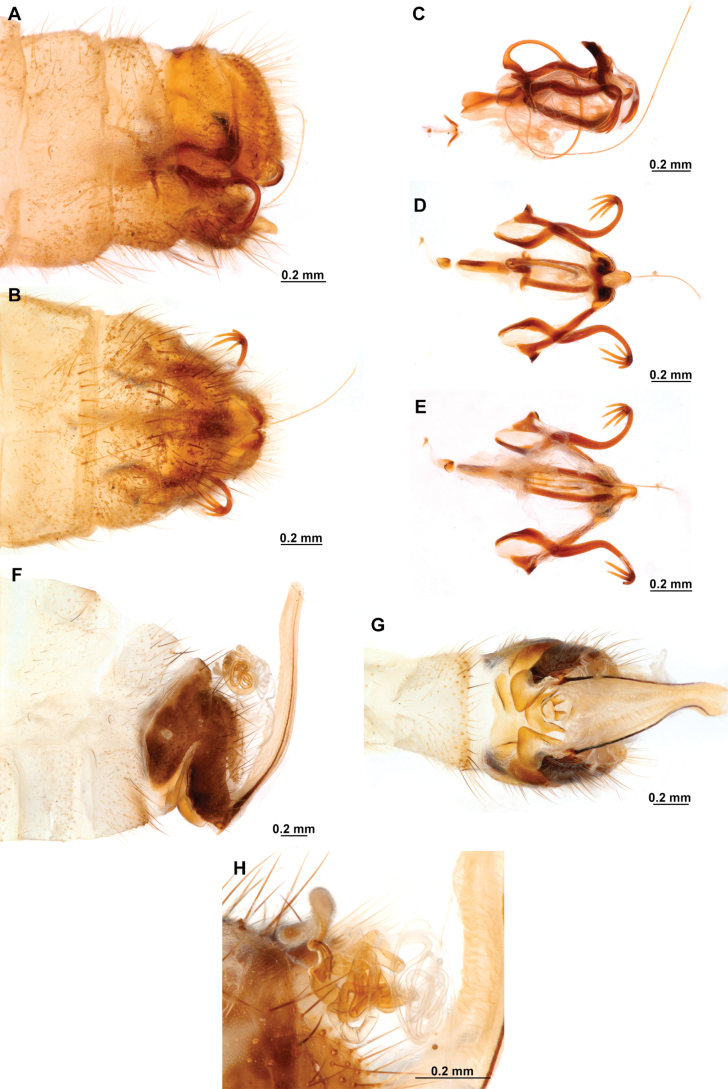
*Trichosceliaanae* Penny, 1982 **A** male terminalia, lateral **B** same, ventral **C** male genitalia, lateral **D** same, dorsal **E** same, ventral **F** female terminalia, lateral **G** same, ventral **H** spermatheca.

***Male genitalia*** (Fig. 80A–E). Tergite IX dorsally slightly narrower than laterally, lateral margin quadrangular, with a posteroventral notch. Sternite VIII rectangular; sternite IX triangular, posteromedially forming an acute angle, with tiny setae, dorsal surface canaliculate; the rest of the surface with abundant, long, thin setae; in lateral view acuminate, with apex reaching posterior margin of ectoproct. Gonocoxite IX short, thin and sinuous, base spatulate; apex lateroventrally curved, with four long, subequal apical digitiform processes. Ectoproct ovoid, setose; ventral surface with anterior region convex, followed posteriorly by a strongly curved, keeled structure. Gonocoxites X forming as a short, thickened, ventrally canaliculate sclerite, anterior apex expanded, posterior apex bent with two short lateral preapical processes. Gonostyli X thin and curved at base, with two lateral processes, the rest of the structure whip-shaped, long, curved ventrally and coiled anteriorly, forming single loop before protruding from the abdomen. Gonapophyses X subparallel, rod-shaped, straight, joined by membrane covering gonostyli X base. Gonocoxites XI V-shaped, medial lobe elaborated, with two differentiated parts: dorsal part as a quadrangular lobe with thickened margins; ventral part composed of a caudally curved, acuminate process with thickened margins; between these parts a narrow, diamond-shaped, less sclerotized region is present; lateral arms short and sinuous, with pointed process laterally projected at the anterior apex.

***Female genitalia*** (Fig. 80F–H). Sternite VII rectangular. Tergite VIII narrower dorsally than laterally, enclosing spiracle of eighth abdominal segment, lateral margin triangular. Membrane between tergite VIII and tergite IX + ectoproct sclerotized, forming a concave, triangular plate. Gonocoxites VIII forming a trapezoidal plate, with two lateral concave areas, where lateral plates of the gonapophyses VIII and the tergite IX + ectoproct rest; gonapophyses VIII with quadrangular, convex medial part; such a part is posteromedially produced into a bilobed process, whose base is convex and lobes digitiform; lateral part of gonapophyses VIII quadrangular, convex, covered with microspinules, connected to tergite IX+ ectoproct. Tergite IX + ectoproct ovoid. Gonocoxite IX long and narrow, as long as the last four abdominal segments together. Bursa copulatrix short, funnel-shaped, with the proximal region sclerotized and concave, the rest thin and membranous. Spermatheca long, entangled; proximal section thin forming three coils, ending in a more internally entangled part; medial section slightly thicker than proximal section, complexly entangled and forming multiple convolutions. Distal section slightly thicker than the medial section, forming several convolutions, progressively wider towards the apex, distally with a short and conical invagination, adjacent to fertilization canal duct. Fertilization canal duct short, thickened, sigmoid; fertilization canal long, thickened, S-shaped, covered with microfilaments.

##### Distribution.

Brazil (Rondônia), Peru (Madre de Dios, San Martín).

##### Remarks.

This is a rather distinctive species of *Trichoscelia* previously known only from its original description (Penny 1982a). It was known from Rondônia (Brazil) and herein its distribution range is extended to Peru (Madre de Dios and San Martín). This is the only species of the genus that has pale preapical flagellomeres and an expanded costal space of forewing. The male genitalia exhibit a distinctive morphology with relatively short gonostyli X forming a single loop and a well-developed median lobe on the gonocoxites XI. The latter condition is only shared with *Trichosceliaandina.* The spermatheca is striking, probably expressing plesiomorphic character states, for instance the apical invagination is short and conical, while the fertilization canal duct and the fertilization canal are noticeably elongated, conditions only expressed by certain species of Rhachiberothinae.

#### ﻿﻿ Trichoscelia andina

Taxon classificationAnimaliaNeuropteraRhachiberothidae

Ardila-Camacho, 2015

Figs 81 , 82

Trichoscelia andina Ardila-Camacho, 2015: 420. Holotype: male, Colombia, Cundinamarca (MPUJ), specimen examined.

##### Material examined.

***Holotype***. Colombia • ♂; **Cundinamarca**, Albán, vereda Santana; 1800 m; 22 Nov. 1981; H. Schmit leg.; MPUJ.

##### Other material.

Colombia – **Cundinamarca** • 1 ♂ 1 ♀; Guaduas, Vda. Las Lajitas, Finca Buenos Aires; 23 May. 1992; B. Tellez leg; UPN. – **Huila** • 1 ♂; Pitalito; 10 Mar. 1973; W.P. Mackay leg.; *Trichosceliavaria* det. N. Penny, 1999; CAS. • 1 ♀; Unknown locality [probably Colombia] Benavides; Jul; H. Smith leg.; MCZ-ENT 006811791; MCZ.

Ecuador • 1 ♀; **Pichincha**, Tinalandia; 10–17 Jul. 1980; H.V. Weems Jr. leg.; FSCA. • 1 ♂; Pichincha, Tinalandia; 21 Jan. 2002; F.T. Hovore leg.; *Trichosceliaegella* det. N. Penny, 2003; CAS.

Panama • 1 ♀; **Canal Zone**, Barro Colorado Island; 22 Apr. 1978; Silberglied, Aiello leg.; at light; USNMENT01541904; USNM.

Venezuela • 1 ♂; [**Carabobo**] San Esteban; Anduge leg.; *Trichosceliabanksi* End., det. N. Banks; FSCA.

##### Diagnosis.

This species is similar in the general color pattern and wing venation to *T.banksi* and *T.flavomaculata*, however, the wings are narrower and more elongate than in the latter, and the male and female genitalia are remarkably distinct from the former. The posterior apex of the gonocoxite IX of *T.andina* possesses 5–8 digitiform processes, arranged in a cluster, while only three are present in *T.banksi*. Furthermore, the median lobe of gonocoxites XI of *T.andina* has a ventral part forming a trapezoidal process with two blunt lateroventral outgrowths, which is absent in *T.flavomaculata*.

##### Description.

***Measurements*.** Male (*n* = 3). Forewing length: 7.6–9.4 mm; Hind wing length: 5.8–6.2 mm. Female (*n* = 2): Forewing length: 8.1–9.3 mm; Hind wing length: 6.1–7.3 mm.

**Figure 81.**
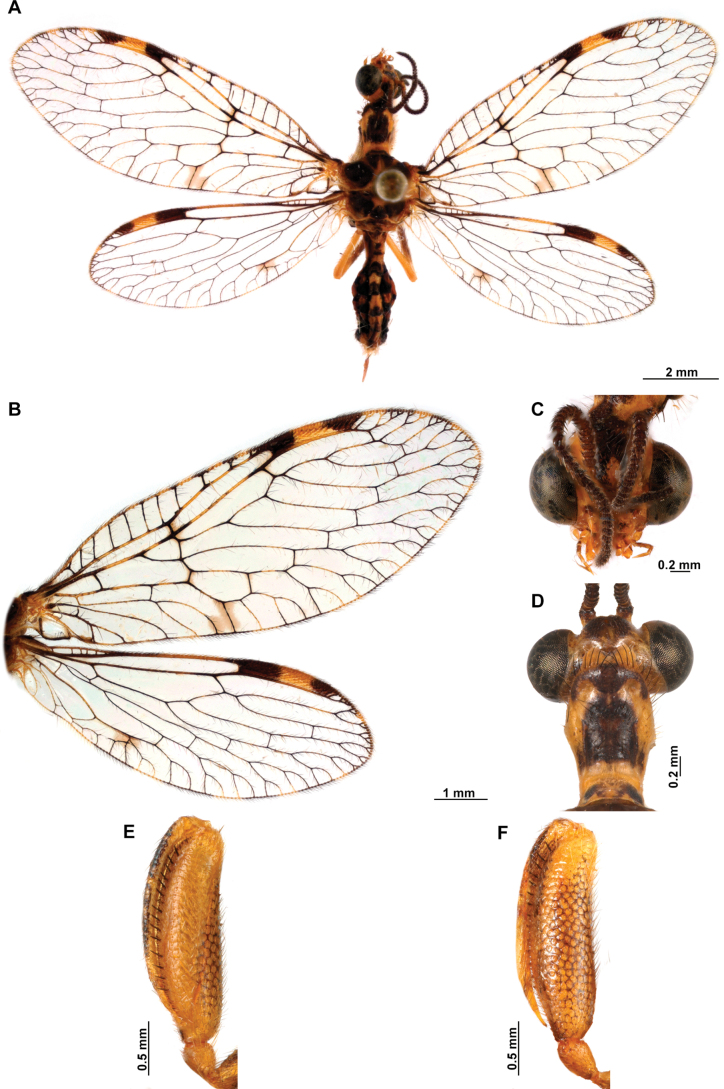
*Trichosceliaandina* Ardila-Camacho, 2015 **A** female habitus, dorsal **B** wings **C** head, frontal **D** pronotum, dorsal **E** forefemur, anterior surface **F** same, posterior surface.

***Coloration* (Fig. 81)**. ***Head*.** Mostly yellow, vertex with a brown band extended from occiput to supraantennal area. Antennal scape yellow, except dorsally on distal ½ brown, setae dark brown; pedicel brown; flagellum dark brown. Frons with transverse, brown band; hypostomal bridge pale brown; clypeus with a brown transverse band and yellow margins, setae yellow. Labrum pale brown with yellow setae; mandible pale amber; maxilla yellow with pale brown palp, the first three palpomeres darker, all with yellow apex; submentum yellow with pale brown setae, prementum and ligula yellow, labial palpus brown with yellow ring at base and apex of each palpomere, palpimacula pale brown. ***Thorax*.** Pronotum yellow with brown broad medial area extending posterolaterally, anterior margin brown, setae brown; episternum bicolor, with pale brown setae; postfurcasternum yellow with brown suffusions. Mesoscutum with sclerites brown with yellow margins, scutellum with medial brown marking and yellow lateral areas, dark brown setae. Meso-pre-episternum mostly brown. Pteropleuron with sclerites mainly brown with yellow margins; setation with same color as cuticle. ***Foreleg*.** Coxa mainly dark brown with yellow areas on the anterior surface, with concolorous setae; trochanter yellow with dark brown suffusions on posterior surface, setation mainly yellow. Femur yellow, with brown area extending on dorsal, anterior and posterior surfaces, it consists of pentagonal brown markings with yellow center; distal portion adjacent to closure surface brown. Tibia yellow with brown areas, setation mostly brown, clavate yellow. Basitarsus yellow, gradually changing to pale brown towards apex, setae mainly brown, clavate setae yellow; remaining tarsomeres pale brown, setae brown. ***Mid- and hind leg*.** Mid-leg with brown coxa, trochanter yellow with brown spot; femur yellow; tibia yellow with brown spot dorsally at base; tarsus yellow. Hind leg with coxa mainly brown, trochanter yellow with brown suffusions, femur yellow, tibia yellow with broad sub-basal band; setation mostly yellow, yellow areas sometimes with brown setae; tibial spurs pale amber, pretarsal claws brown. ***Wings*.** Forewing hyaline; longitudinal veins alternating brown and yellow, subcostal veinlets, and crossveins brown; pterostigma brown with yellow medial area; wing margin alternating yellow and brown. Hind wing hyaline; C + Sc, medial area of RA, base of CuP and anal veins yellow, remaining longitudinal veins mostly brown; subcostal veinlets and crossveins brown; pterostigma brown with yellow preapical area; wing margin alternating yellow and brown. ***Abdomen*.** Cleared.

***Morphology* (Fig. 81)**. ***Head*.** Semi-triangular in frontal view, smooth, vertexal region slightly domed above compound eyes, with rows of short, reclined setae; paraocular area concave; coronal suture discernible. Compound eye hemispheric, enlarged, as wide as ¾ of interocular distance at toruli level. Antenna moniliform, scape 1.5× as long as wide, slightly distally expanded, with short, thin setae; pedicel as long as wide; flagellum slightly dorsoventrally flattened, basal flagellomeres 1.5× as wide as long, progressively narrower towards the apex, all articles with a medial ring of short and thin setae. Frons narrow, trapezoidal, with thin and short setae. Postgena broad; hypostomal bridge not entirely fused with postgena. Clypeus rectangular, narrow. Labrum rounded, with fine, long setae; maxillary palpus with first palpomere as long as wide, second 1.5× as long as wide, third 3× as long as wide, fourth 2× as long as wide, fifth as long as the third. Submentum rounded with long, thin setae; labial palpus with first palpomere slightly longer than wide, second 2× as long as wide, third slightly longer than second, not noticeably expanded, with ovoid palpimacula. ***Thorax*.** Pronotum 1.5× as long as wide, with a groove contiguous to lateral and distal margins; posterior margin, medial area and anterior margin slightly raised with pedicellate, long, thickened setae, remaining surface short and thin; episternum with long, thin setae; postfurcasternum trapezoidal. Mesonotum ~ 2× as wide as long, mesoscutum with some pedicellate, thickened, erect setae on medial area; scutellum with a few pedicelled, thickened, long setae and thin, short setae. Metanotum 2× as wide as long, scutum with lateral cusps and a medial depression; scutellum semi-triangular, with rounded posterior margin. Pteropleura covered with abundant short, thin setae. ***Foreleg*.** Coxa cylindrical, almost as long as femur, with abundant, fine, long setae; trochanter trapezoidal, setose, dorsally with thickened setae near distal margin. Femur robust, sub-cylindrical, setose; closing surface both rows of processes fully developed, composed of tubercle-shaped, specializations; posteroventral row with single more developed basal process (process/seta ratio 1:1), anteroventral row with single more developed sub-basal process (process/seta ratio 1:1); rows of thickened setae with globular base fully developed, extended along the entire closing surface. Tibia curved, slightly shorter than femur, setose; anterior surface with apical patch of clavate setae; closing surface keeled, with row of flattened, prostrate setae. Basitarsus elongated, basal ½ with clavate setae on anterior surface, ventrally keeled with row of prostrate setae; lanceolate process short, reaching the base of fourth tarsomere, with plug-shaped Stitz organ at apex; second tarsomere 4× as long as wide, third tarsomere slightly longer than wide, fourth tarsomere 2× as long as wide. ***Mid- and hind leg*.** Mid-leg covered with abundant, long, thin setae, shorter on tarsus. Tibia slightly flattened, thin; basitarsus ~ 3× as long as wide, second tarsomere slightly longer than wide, third and fourth tarsomeres as long as wide, fifth tarsomere 1.5× as long as wide; first four tarsomeres with a pair of lateral, thickened setae at distal margin on plantar surface. Hind leg densely covered with long, thin setae, shorter on tarsus; tibia slightly thickened; tibial spurs short; tarsomeres similar to those of mid-leg. ***Wings*.** Forewing oval, trichosors present along wing margin except at wing base; venation setose; costal space narrow, with simple or forked humeral vein; 10–12 subcostal simple veinlets; pterostigma rectangular, narrow, gently curved, with numerous, incomplete, simple veinlets; subcostal space with single, medially located crossvein; Sc vein abruptly bent posteriad at the proximal margin of pterostigma to merge RA; *rarp2* gently curved, two RP branches arising from each anterior radial cell; M fused basally to RA; base of the RP located near separation of M and RA, M fork near such separation; 1r-m located between base of RP and MA fork, forming a small, trapezoidal cell; four gradate crossveins present. Cu deeply forked; CuP proximally angled, touching A1, distally forked at level of divergence of M and R. 1A simple ending at the level of separation of M and R; 2A distally forked, slightly beyond the level of CuP angle. Jugal lobe with abundant setae on the posterior margin. Hind wing oval, smaller and narrower than forewing; venation densely setose; costal space narrow and reduced, with four or five subcostal veinlets; C and Sc fused at proximal ¼ of wing length, subcostal space without crossveins, gradually widening towards apex; Sc vein abruptly curved posteriad at proximal pterostigma margin to merge RA; pterostigma elongated and narrow, straight; radial space widened with a single crossvein, straight; two veins arising from *rarp1*, one from *rarp2*. Vein 1r-m sigmoid, connecting base of M and base of RP, distally connected again to M stem through short crossvein. M vein forked beyond the level of R fork. Cu deeply forked, CuA long, sinuous, distally forked, first branch candelabrum-shaped; CuP distally anteriorly curved, pectinate. A1 long and arched; A2 simple, short, and arched. ***Abdomen*.** Cylindrical, scarcely setose; tergites quadrangular; intertergal membrane between abdominal segments III–V broad, setose, with glabrous lateral areas; tergites V and VI with two lateral scars near posterior margin. Sternites rectangular, with short, thin setae.

**Figure 82.**
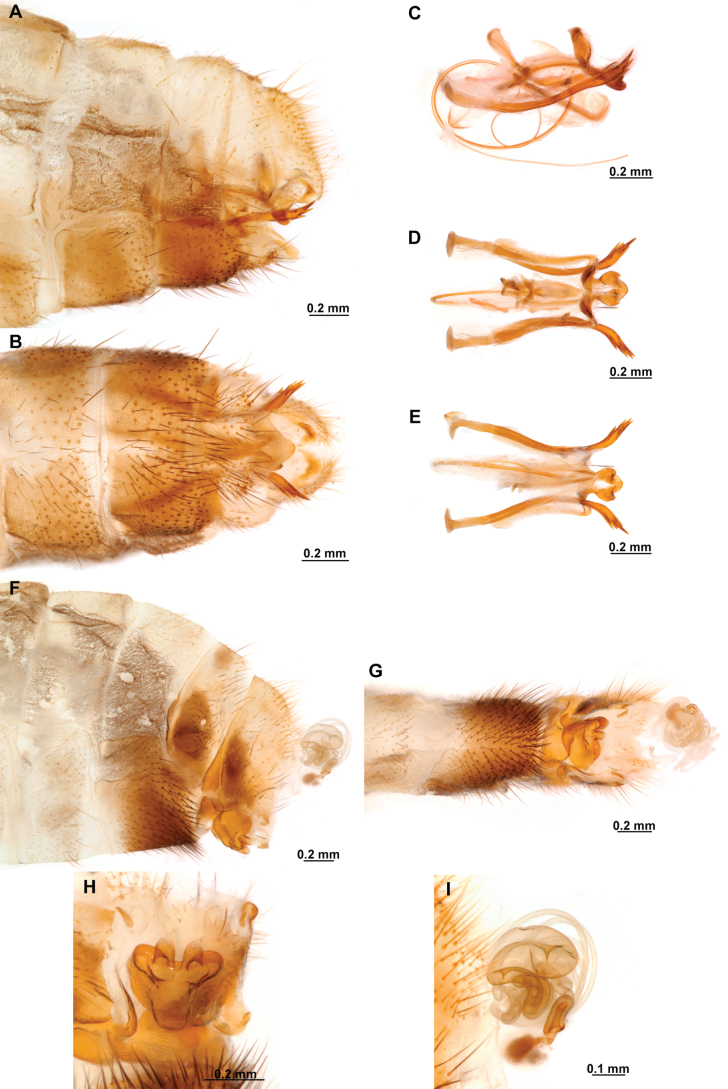
*Trichosceliaandina* Ardila-Camacho, 2015 **A** male terminalia, lateral **B** same, ventral **C** male genitalia, lateral **D** same, dorsal **E** same, ventral **F** female terminalia, lateral **G** same, ventral **H** gonapophyses VIII ventral **I** spermatheca.

***Male genitalia*** (Fig. 82A–E). Tergite IX narrower dorsally than laterally, posteroventrally notched. Sternite VIII rectangular with concave posterior margin. Sternite IX pentagonal, setose, apex acuminate and slightly curved ventrad, dorsally canaliculate; in lateral view slightly surpassing posterior margin of ectoproct. Gonocoxite IX short and sinuous, base spatulate and dorsally bent; apex laterally recurved, with 5–8 digitiform processes, arranged in a cluster. Ectoproct triangular, setose, ventrally broadly concave, with posterior margin sclerotized. Gonocoxites X forming a short, straight, ventrally canaliculate sclerite, anterior apex expanded, posterior apex with dorsal processes connected to gonostyli X, and two short, preapical, lateroventral processes connected to gonapophyses X. Gonostyli X not thickened, curved, with two lateral processes at base; the rest of the structure long, abruptly anteroventrally curved, anteriorly coiled, forming two internal loops after protruding from abdomen. Gonapophyses X rod-shaped, long, straight; posterior apex dorsally bent, with surrounding membrane set with microspinules; gonapophyses joined by a membrane covering the base of the gonostyli X. Gonocoxites XI U-shaped, with medial lobe complex and elaborated composed of two differentiated parts: dorsal part as a broad, quadrangular, concave lobe; ventral part as a trapezoidal process with two blunt lateroventral outgrowths; ventral margin deeply incised; area between these parts less sclerotized; lateral arms of gonocoxites XI short, gently curved;

***Female genitalia*** (Fig. 82F–I). Sternite VII subtrapezoidal. Tergite VIII slightly narrower dorsally than laterally, enclosing spiracle of eighth abdominal segment, lateral margin quadrangular. Membrane between tergite VIII and tergite IX + ectoproct sclerotized, forming a concave triangular plate. Gonocoxites VIII forming a trapezoidal, concave plate; gonapophyses VIII with thickened, convex, keel-shaped medial part, posteromedially with two blunt processes; lateral part of gonapophyses VIII triangular, connected to tergite IX + ectoproct. Tergite IX + ectoproct semi-triangular, setose. Gonocoxite IX long and narrow, as long as the last four abdominal segments together. Bursa copulatrix long, funnel-shaped, proximal part sclerotized, concave, the rest of the structure thin and membranous. Spermatheca long, complex and entangle; proximal section forming four coils, ending in a more internal spiral; medial section thicker than proximal and distal section, with broader S-shaped part which is continuous with entangled part that forms multiple convolutions. Distal section thin forming several convolutions, progressively wider towards the apex, distally with a trumpet-shaped invagination. Fertilization canal duct short, thin, and sigmoidal; fertilization canal short, thickened, L-shaped, covered with microfilaments.

##### Distribution.

Colombia (Cundinamarca, Huila), Ecuador (Pichincha), Panama (Canal Zone), Venezuela (Carabobo).

##### Remarks.

This species was previously known only from its original description in the department of Cundinamarca in Colombia. Here the distribution of this species is extended to the department of Huila, and new records from Ecuador (Pichincha), Panama (Canal Zone), and Venezuela (Carabobo) are provided. *Trichosceliaandina* has a body coloration pattern markedly similar to *T.banksi* from Mexico and Central America and *T.flavomaculata* from Central America, but it can be distinguished to them by the enlarged ventral process of the male gonocoxites XI, the shape of the medial part of the female gonapophyses VIII and the spermatheca.

#### ﻿﻿ Trichoscelia banksi

Taxon classificationAnimaliaNeuropteraRhachiberothidae

Enderlein, 1910

Figs 83 , 84

Trichoscelia banksi Enderlein, 1910: 337. Holotype: male, Mexico, Chiapas (MZPW), photographs examined.
Trichoscelia santareni (Navás, 1914). Penny (1977): 37. New synonym.
Symphrasis santareni Navás, 1914: 25. Syntype: male, Mexico, Tabasco (NHMUK), specimen examined.

##### Material examined.

***Holotype*** of *Trichosceliabanksi*.

Mexico • ♂; **Chiapas**; 02 Nov. 1907; L. Conradt S. leg.; *Trichosceliabanksi* Enderlein ♀, det. Dr. Enderlin, Type; MZPW.

***Lectotype*** of *Trichosceliasantareni*.

Mexico • ♂; **Tabasco**, Teapa; Apr.; H.H.S., Godman-Salvin collection 1913–214; “*Symphrasissantareni* Nav. Navás S.J. det., Typus, Type H.T.” red label; NHMUK 013802767; NHMUK. Note: Lectotype and paralectotype of *T.santareni* are herein designated since Navás (1914), did not explicitly designated a specimen as the holotype. The specimens deposited in the Natural History Museum, London and cited here are considered as syntypes.

***Paralectotype*** of *Trichosceliasantareni*.

Mexico • ♂; **Tabasco**, Teapa; Feb.; Godman-Salvin collection 1913-214; “Syntype” blue label; NHMUK 013802766; NHMUK.

##### Other material.

Costa Rica – **Cartago** • 1 ♂; Turrialba; 1–6 Mar. 1965; SS & D. Duckworth leg.; USNMENT01541901; USNM. • 1 ♂; P.N. Tapantí; 1650 m; Feb. 1994; G. Mora leg.; L-N, 194000, 559800, CRI001913997; MNCR. • 1 ♀; Dulce Nombre, Tajo La Chilena; 1400–1500 m; 23 Apr. 2009; B. Hernandez, M. Moraga leg.; Trampa de luz de Mercurio; INBIO0004206465; MNCR. –**Guanacaste** • 1 ♀; Estación Pitilla, 9Km. S de Santa Cecilia, P.N. Guanacaste; 700 m; Aug. 1991; P. Ríos leg.; L-N 330200, 380200, CRI000608208; MNCR. • 1 ♂; Nicoya Ostional Río Montana; 0–100 m; 15 Jun. 2004; B. Gamboa, D. Briceño, M. Moraga, Y. Cárdenas leg.; L-N, 218850, 352050, 77315, IMB0003853250; MNCR. • 1 ♀; Derumbe est. Cacao, lado oeste del V[olcán] Cacao; 1400 m; 21–29 May. 1992; L-N 323700, 376700, INBIOCRI000409761; MNCR. – **Heredia** • 1 ♂; Finca Naranjo Valenciana, 2 Km S Pueblo Nuevo, Sarapiquí; 90 m; 24 Jul–22 Aug. 1992; M. Ortiz leg.; L-N 271800, 523750, CRI000877267; MNCR. • 1 ♀; 11 km SE la virgen; 10°20'N, 84°04'W; 450–550 m; 24 Feb. 2003; “INBIO-OET-ALAS transect 8–18 Apr. 2003-RVC-008 Transect”; MNCR. • 1 ♂; Est. Biol. La Selva; 10°26'N, 84°01'W; 50–150 m; Mar.1999; “INBIO-OET, 8–25 Mar. 1999, área laboratorios I/00/594”, CRI002739378; MNCR. – **Puntarenas** • 1 ♀; Finca Cafrosa, Embalse, 800 m N.O. de Tigra; 1280 m; 13–21 May. 1996; E. Navarro leg.; L-N 317800, 596200, 7494, CRI002375011; MNCR.

Guatemala • 1 ♀; **Suchitepequez**, Fca. Moca; 12 Jun. 1966; Flint & Ortiz leg.; USNMENT01541903; USNM.

Honduras • 1 ♂; **El Paraíso**, Dept. Yuscaran; 25 May. 1993; F.W. Skillman Jr. leg.; MV light; FSCA.

Mexico – **Chiapas** • 1 ♀; Cacahoatán, Ejido Benito Juarez, El Plan; 15°05'27.66"N, 92°08'50.5"W; 1487 m; 07 Jun. 2018; R. Cancino & M. Luna leg.; UV and white light trap; det. A. Ardila; CNIN. • 1 ♂ 2 ♀; Cacahoatán, Ejido el Águila; 15°05'33.24"N, 92°10'50.64"W; 1194 m; 07 Sep. 2018; R. Cancino & M. Luna leg.; UV and white light trap; CNIN. • 1 ♂; same data as for preceding; 10 Aug. 2018; CNIN. • 1 ♂; Cacahoatán, Finca Alianza; 15°02'28.74"N, 92°10'9.48"W; 661 m; 01 Nov. 2018; R. Cancino & M. Luna leg.; UV and white light trap; CNIN. • 1 ♀; Cacahoatán, Finca Alianza; 15°03'35.82"N, 92°10'34.68"W; 749 m; 02 Dec. 2018; R. Cancino leg.; aerial net; CNIN. • 1 ♂; Union Juarez, Finca Monteperla; 15°02'40.8"N, 92°05'17.4"W; 926 m; 08 Aug. 2018; R. Cancino leg.; UV and white light trap; det. A. Ardila; CNIN. – **Oaxaca** • 1 ♂; Distrito de Ixtlán, Ixtlán de Juárez, Universidad de la Sierra de Juárez; 17°18'55"N, 96°28'57.8"W; 1956 m; 22 May. 2015; J.A. Casasola leg.; CNIN. – **Querétaro** • 1 ♂; 18 mi E. Landa de Matamoros 6500; 26 May. 1974; C. & L. O’ Brien & Marshall leg.; CAS. – **Tabasco** • 1 ♂; Municipio Tacotalpa, Río de la Sierra; 17°28.04'N, 92°46.52'W; 09 Feb. 1998; J. Bueno & R. Barba leg.; CNIN. – **Veracruz** • 1 ♂; Estación de Biología Los Tuxtlas; 160 m; 20 May. 1985; A. Ibarra leg.; 03372; CNIN. • 1 ♂; Xalapa; 08 Jun. 1985; J. Peña leg.; CNIN.

Nicaragua • 2 ♀; **Río San Juan** Monte Cristo Resort, 3 Km SE Boca de Sábalos; 03–10 May. 2005; N.J. Smith leg.; UCD.

##### Diagnosis.

This species is distinguished from *T.flavomaculata* by a forefemur more markedly pigmented, with wide brown area on anterior and posterior surfaces, forming a honeycomb pattern, in which setal bases are yellow. Moreover, *T.banksi* is separated of the remaining species of the genus because the gonocoxite IX has the posterior apex equipped with three digitiform processes, of which two are apical, subequal and laterally curved, and the third is longer, preapical situated, being twisted or recurved laterally. On the female genitalia, the medial part of gonapophyses VIII is broad, keel-shaped, posteromedially with a bilobed process.

##### Description.

***Measurements*.** Male (*n* = 4). Forewing length: 7.9–8.3 mm; Hind wing length: 6.2–6.6 mm. Female (*n* = 1): Forewing length: 8.7 mm; Hind wing length: 6.8 mm.

**Figure 83.**
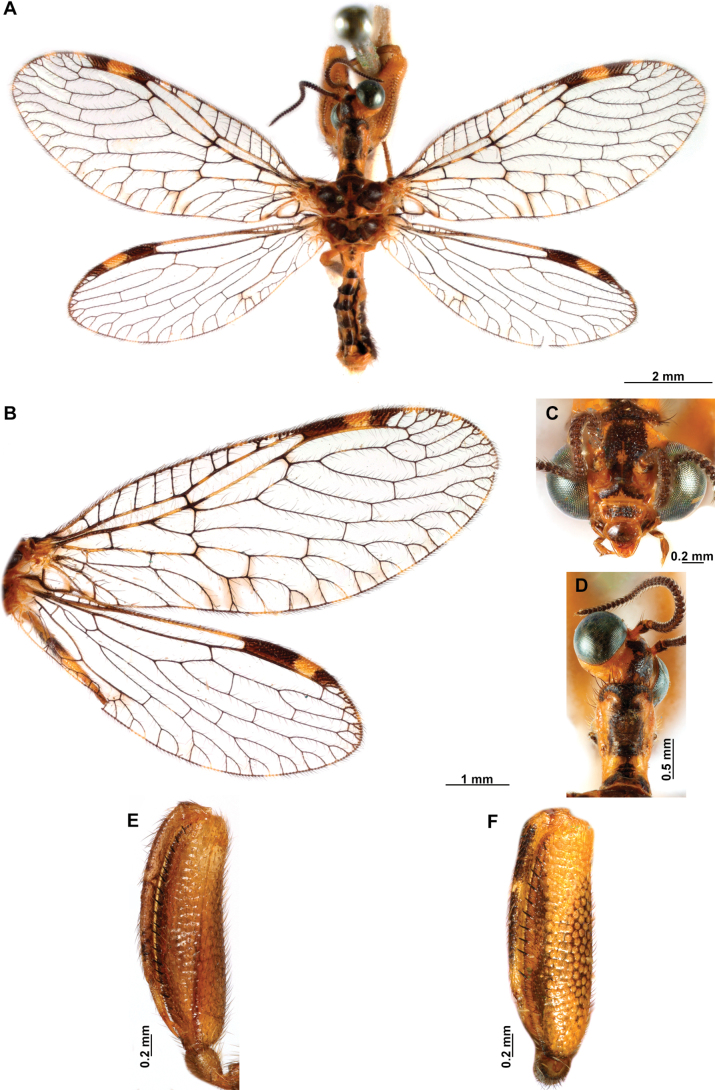
*Trichosceliabanksi* Enderlein, 1910 **A** male habitus, dorsal **B** wings **C** head, frontal **D** pronotum, dorsal **E** forefemur, anterior surface **F** same, posterior surface.

***Coloration* (Fig. 83)**. ***Head*.** Mainly yellow, vertexal region with longitudinal brown band extended from occipital ridge to supraantennal area where it forks, with brown setae. Antennal scape yellow with brown distal margin on dorsal surface; pedicel bicolor with yellow and brown; flagellomeres brown. Frons with brown suffusions; clypeus brown with yellow margins, labrum yellow with brown suffusions at center; mandible yellow, with brown suffusions at base, apex pale amber. Maxilla yellow, with pale brown palpus. Labium yellow with pale brown labial palpus, palpimacula pale brown. ***Thorax*.** Pronotum yellow with brown longitudinal, medial, broad band which is extended laterally on anterior margin; episternum bicolor, brown on anterior ½, yellow on posterior ½; postfurcasternum yellow. Mesonotum predominantly brown, area adjacent to sutures yellow, scutellum yellow laterally and brown at center; metanotum brown with pale yellow central area. Pre-episternum brown; Pteropleura yellow with broad brown areas. ***Foreleg*.** Coxa yellow with brown areas; trochanter brown ventrally, yellow dorsally. Femur yellow, with wide brown area on anterior and posterior surfaces, forming a honeycomb pattern, in which setal bases are yellow; area adjacent to closing surface on both surfaces with large brown marks; setae mostly brown, yellow on the femoral apex. Tibia yellow with two brown bands on the dorsal surface, anterior surface with brown area adjacent to closing surface at base; setae mostly brown, pale clavate setae brown. Basitarsus with medial area yellow with dark brown suffusions; basal region and lanceolate process pale amber, clavate setae yellow; second to fourth tarsomere pale brown. ***Mid- and hind leg*.** Mid-leg with brown coxa, yellow trochanter with brown infuscation on posterior surface; femur yellow, tibia yellow with brown ring near middle; first four tarsomeres yellow with pale amber apex, fifth tarsomere pale brown. Hind leg with coxa mainly brown with yellow area at center, trochanter, and femur yellow; tibia yellow with extensive brown sub-basal area, tarsomeres as in mid-leg. ***Wings*.** Forewing hyaline, sometimes with amber on area adjacent to first branch of CuA and apex of CuP; pterostigma brown with yellow medial area; longitudinal veins alternating dark brown and yellow, crossveins and subcostal veinlets dark brown; posterior and distal margin alternating brown and yellow. Hind wing hyaline, sometimes with amber on area adjacent to stem of first branch of CuA; pterostigma brown with small, yellow, preapical area; venation mostly brown, except yellow C + Sc; posterior and distal margin alternating yellow and brown. ***Abdomen*.** Tergites of abdominal segments I and II yellow with brown medial spot, tergites III-VI with wide brown posteromedial area, with a dark brown medial band on remaining segments. Sternites of segments I–III yellow, sternites IV–VI with yellow area at center, laterally brown, remaining sternites brown. Pleural membrane yellow with extensive brown areas.

***Morphology* (Fig. 83)**. ***Head*.** Diamond-shaped in frontal view, moderately setose, smooth, vertexal region domed above compound eyes; paraocular area concave; coronal suture discrete. Compound eye hemispheric, as wide as ¾ interocular distance at torulus level. Broad postgena, hypostomal bridge not completely fused with it. Antenna moniliform, scape ~ 2× as long as wide, slightly distally expanded; pedicel as long as wide; flagellum slightly dorsoventrally compressed, with 35 flagellomeres, those of basal ½ 1.5× as wide as long, the rest almost as wide as long, progressively smaller towards apex; all flagellomeres with a medial ring of thickened setae. Maxillary palpus the first two palpomeres as long as wide, third palpomere 3× as long as wide, fourth ~ 2× as long as wide, fifth palpomere tapering, slightly shorter than the third. Postmentum oval with long setae, labial palpus with first palpomere 1.5× as long as wide, second 3× as long as wide, third as long as the second, widely expanded, palpimacula widely ovoid. ***Thorax*.** Pronotum 1.5× as long as wide, with furrow contiguous to lateral and anterior margins; in lateral view anterior margin, medial region and posterior margin slightly elevated, with thickened, pedicellate setae; episternum with long fine setae, postfurcasternum trapezoidal. Mesonotum 1.5× wider than long, with thick, pedicellate on medial area. Metanotum ~ 2× as long as wide, mostly glabrous. Pteropleura covered with abundant short, thin setae. ***Foreleg*.** Coxa slightly shorter than femur, cylindrical, with abundant long and thin setae; trochanter subtrapezoidal; femur robust, with short and thin setae; closing surface with double row of tubercle-shaped specializations fully developed, both with a more developed sub-basal process (process/seta ratio 1:1); rows of thickened setae with globular base adjacent to each row of processes present, extending over almost the entire closing surface. Tibia almost as long as femur, curved, with short and thin setae; closing surface with prostrate setae; anterior surface with a patch of clavate setae at apex; basitarsus elongated, ventrally keeled and with a row of prostrate setae on proximal ½, anterior surface with a patch of clavate setae proximally; lanceolate process, reaching the base of fourth tarsomere, with plug-shaped Stitz organ at apex; second tarsomere 4× as long as wide, third tarsomere as long as wide, fourth tarsomere 2× as long as wide. ***Mid- and hind leg*.** Mid-leg with coxa and trochanter covered with long, thin setae, tibia slightly longer than femur, both with short, thin setae; basitarsus 3× as long as wide, second tarsomere slightly longer than wide, third and fourth as long as wide, fifth tarsomere as long as wide, all covered with short, thin setae; the first four tarsomeres with a pair of thickened setae laterally on distal margin of plantar surface. Hind leg longer than mid-leg, densely setose, tibia 1.5× as long as femur; tarsomeres similar to those of mid-leg. ***Wings*.** Forewing oval, trichosors present along wing margin except at wing base, venation setose; costal space slightly widened medially, humeral vein simple, forked or branched, 9–11 subcostal veinlets; pterostigma elongated and narrow, composed of numerous veinlets, mainly incomplete; subcostal space with a single medially located crossvein; Sc vein bent posteriad at proximal margin of pterostigma to merge the RA; *rarp2* curved, with two or three veins arising from it, two or three from *rarp1*; M fused basally to R; RP base located near separation of M and R, M fork near such separation, opposite to R fork; 1r-m located between RP base and MA base, forming a small trapezoidal cell; three or four gradate crossveins present. Cu vein deeply forked; CuP basally angled, approaching A1, distally forked, opposite to separation of M and R. 1A simple, reaching the level of separation of M and R; A1 distally forked, at level of CuP angle. Hind wing smaller and narrower than forewing; costal space narrow and reduced, with 4–6 veinlets; Sc and C fused at 1/5 of wing length, Sc abruptly curved posteriad at proximal margin of pterostigma, to merge the RA; pterostigma elongated, narrow, curved, composed of numerous incomplete veinlets; radial space with a single crossvein, oblique; two veins arising from *rarp1*, one from *rarp2*. M forked beyond R fork; 1 r-m sigmoid, connecting M base and RP base, distally connected again with M stem through short crossvein. Cu deeply forked, CuA long and sinuous, distally forked, first branch candelabrum-shaped; CuP distally bifurcated, distally anteriorly curved, pectinate. Cubitoanal space with two crossveins. A1 arched, A2 short and arched. ***Abdomen*.** Cylindrical, tergites quadrangular; intertergal membranes between segments III–VI in male and III–V in female broad, covered with microtrichia, and with two glabrous lateral areas; sternites rectangular.

**Figure 84.**
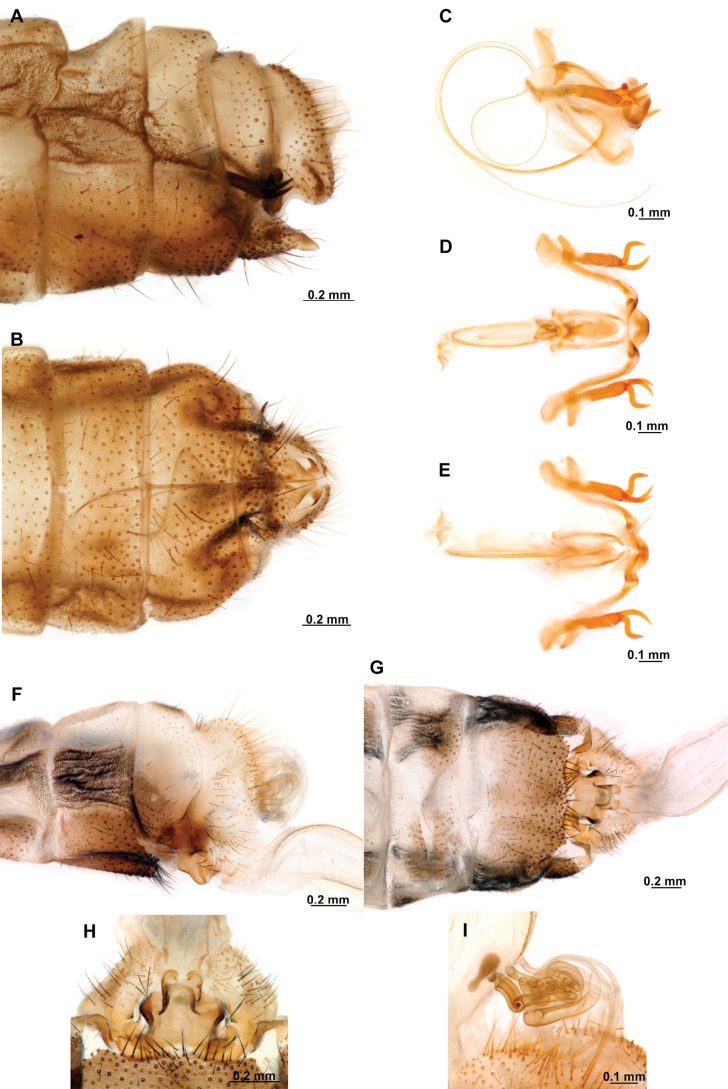
*Trichosceliabanksi* Enderlein, 1910 **A** male terminalia, lateral **B** same, ventral **C** male genitalia, lateral **D** same, dorsal **E** same, ventral **F** female terminalia, lateral **G** same, ventral **H** gonapophyses VIII ventral **I** spermatheca.

***Male genitalia*** (Fig. 84A–E). Tergite IX slightly narrower medially than laterally, lateral margin rounded, with posteroventral notch. Sternite VIII subrectangular; sternite IX triangular, posteromedially forming an obtuse angle, glabrous, dorsally canaliculated, remaining surface with abundant long, thin setae; in lateral view acuminate, with apex slightly bent and reaching the posterior margin of ectoproct. Gonocoxite IX short and thickened, base spatulate; apex with three digitiform processes, two apical, subequal and laterally curved, and a longer preapical process that is twisted or recurved laterally. Ectoproct ovoid, setose; ventral surface with flattened, sclerotized anterior lobe, posteriorly continuous by wide concavity whose posterior margin is more sclerotized. Gonocoxites X forming a short, thickened, ventrally canaliculate sclerite; anterior ½ expanded, posterior apex bilobed, and with two preapical, lateroventral processes. Gonostyli X with thin and curved base, with two lateral processes, the rest of the structure long, curved ventrally and coiled anteriorly, forming an internal loop before protruding from the abdomen. Gonapophyses X rod-shaped, straight, posterior apex dorsally bent, surrounding membrane set with minute spinules; gonapophyses arranged in a V-shaped structure, joined by membrane covering gonostyli X base and set with microspinules. Gonocoxites XI U-shaped, medial lobe expanded and elaborated with two differentiated parts: dorsal part as a quadrangular lobe; ventral part as a convex area covered with microspinules, ventral margin incised; between these parts a narrow, less sclerotized region is present. Lateral arms of gonocoxites XI sigmoid, with anterior apex latero-ventrally bent.

***Female genitalia*** (Fig. 84F–I). Sternum VII rectangular. Tergite VIII slightly narrower dorsally than laterally, enclosing the spiracle of the eighth abdominal segment, lateral margin U-shaped. Membrane between tergite VIII and tergite IX + ectoproct sclerotized, forming a concave, triangular plate. Gonocoxites VIII forming a narrow plate, with two lateral concavities; medial part of gonapophyses VIII broad, keel-shaped, posteromedially with bilobed process; lateral part as a triangular plate, ventrally forming a flattened and curved lobe, covered with microspinules. Tergite IX + ectoproct trapezoidal. Gonocoxite IX long and narrow, as long as the last four abdominal segments together. Bursa copulatrix funnel-shaped, membranous, long, thin, proximally sclerotized and concave; spermatheca complex and entangled; proximal section long, thin, forming four coils; medial section thicker than proximal section, forming multiple convolutions, with notable widening. Distal section thin, forming several convolutions, progressively wider towards apex, distally with a trumpet-shaped invagination. Fertilization canal duct short, thin, sigmoid; fertilization canal short, thickened, J-shaped, covered with microfilaments.

##### Distribution.

Costa Rica (Cartago, Guanacaste, Heredia, Puntarenas), Guatemala (Suchitepequez), Honduras (El Paraíso), Mexico (Chiapas, Oaxaca, Querétaro, Quintana Roo, Tabasco, Veracruz), Nicaragua (Río San Juan).

##### Remarks.

For a long time, the identity of *T.banksi* was poorly understood due to lack of enough details in the original description by Enderlein (1910). Since its description, the species was known only from its type locality in Chiapas, Mexico and it was believed that the type was destroyed (L. Stange pers. comm.). After examining the type series of *T.santareni* and observing high resolution images of the holotype of *T.banksi*, it was concluded that both are indeed the same species. Considering that *T.banksi* is the oldest name, here *T.santareni* is newly synonymized with it.

The name of *T.santareni* was previously known from the Mexican states of Chiapas, Quintana Roo and Tabasco. Here the distribution range of *T.banksi* is extended to Costa Rica (Cartago, Guanacaste, Heredia, Puntarenas), Guatemala (Suchitepequez), Honduras (El Paraíso), and is reported from the Mexican states of Oaxaca, Querétaro, and Veracruz for the first time.

This species is quite similar to the Central American *T.flavomaculata*, but can be distinguished from it by genitalic features, particularly the male gonocoxites IX with three digitiform processes and the distinctive morphology of the female gonapophyses VIII.

#### ﻿﻿ Trichoscelia basella

Taxon classificationAnimaliaNeuropteraRhachiberothidae

(Westwood, 1867)

Figs 85 , 86

Mantispa (Trichoscelia) basella Westwood, 1867: 504. Holotype: male, “Amazonas” [probably Brazil], no specific locality (OUMNH). Specimen examined. Erroneously synonymized with T.iridella (Westwood) by Penny (1982a): 431.

##### Material examined.

***Holotype***. Brazil • ♂; **Amazonas**; 1861; Bates leg.; “Holotype male of *Trichosceliabasella* Westwood, 1867, ascertained by R.G. Beard 1968, *basella* Westwood, this is the unique male type of *T.basella* Westwood, 1867, the (lectoallotype) *iridella* is a female specimen, male of which is in BMNH” “*Trichosceliabasella* Westwood, 1867; Type Westwood Trans Ent. Soc. 1867, 504, Coll. Hope Oxon”; OUMNH.

##### Other material.

Brazil • 1 ♀; **Rondônia**, 62 Km SW Ariquemes nr. Fazenda Rancho Grande; 5–17 Oct. 1993; J.E. Eger leg.; MV and black lights; FSCA. • 1 ♂; 62 Km SW, Ariquemes, Fzda. Rancho Grande; 07 Oct. 1993; C.W. & L.B. O’ Brien leg.; merc. Vap. & UV light; *Trichosceliairidella* det. Penny, 1994; CAS. • 1 ♀; Rondônia, Fazenda Rancho Grande, 62 Km S. Ariquemes; 12–22 Nov. 1991; L.G. Bezark & D.E. Russell leg.; *Trichosceliavaria*, det. N. Penny, 1997; CAS.

Bolivia • 1 ♀; [**La Paz**] Tumupasa; Dec. 1921–1922; W.M. Mann leg., Mulford Biol. Expl.; USNMENT01541898; USNM.

##### Diagnosis.

This species is recognized by the wide pale brown or orangish area of the pterostigmata. This species can be separated from *T.iridella* because a gonocoxite IX short and notably curved, whose posterior apex is laterodorsally recurved, and equipped with five digitiform processes of different sizes, of which three are apical and medially located and two are preapical and lateral. On the female genitalia, the medial part of gonapophyses VIII is convex, keel-shaped, posteromedially bilobed, with two dorsally bent, rounded, lateral fins.

##### Description.

***Measurements*.** Male (*n* = 2). Forewing length: 9.1–9.4 mm; Hind wing length: 7.0–7.2 mm. Female (*n* = 3): Forewing length: 8.0–8.6 mm; Hind wing length: 6.3–6.7 mm.

**Figure 85.**
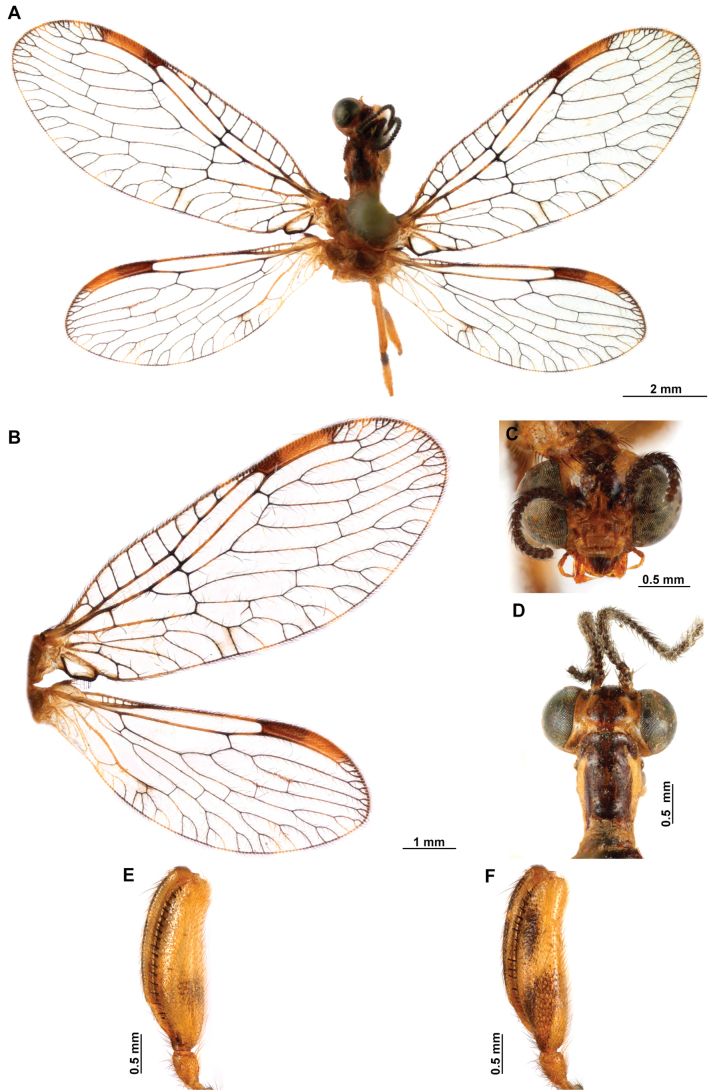
*Trichosceliabasella* (Westwood, 1867) **A** male habitus, dorsal **B** wings **C** head, frontal **D** pronotum, dorsal **E** forefemur, anterior surface **F** same, posterior surface.

***Coloration* (Fig. 85)**. ***Head*.** Mostly yellow, vertexal region with brown band extending from occiput to interantennal region. Antenna with scape mainly brown, distally with brown setae; pedicel and flagellum brown with dark brown setae. Frons brown; clypeus brown with yellow anterior margin; hypostomal bridge yellow. Labrum dark brown with yellow posterolateral corners; mandible brown; maxilla yellow with dark brown palpus; postmentum yellow, prementum yellow, ligule dark brown, labial palpus pale brown. ***Thorax*.** Pronotum yellow with medial band and anterior margin brown; episternum mostly brown, with pale brown setae; postfurcasternum yellow. Meso- and metanotum mainly brown, with area adjacent to wing base yellow. Meso-pre-episternum mostly brown. Mesopleuron with sclerites mainly brown with yellow margins; metapleuron mainly yellow; setation mainly pale brown or yellow. ***Foreleg*.** Coxa yellow with brown apex, setation mainly yellow; trochanter yellow. Femur mostly yellow, with distal area on posterior surface composed of brown pentagonal markings with yellow center, setation yellow. Tibia mainly brown, with irregular yellow areas on the anterior surface, setation mostly yellow. Basitarsus yellow with pale brown areas; remaining tarsomeres yellow. ***Mid- and hind leg*.** Mid-leg with mostly brown coxa, trochanter and femur yellow, tibia yellow with brown spot at base; tarsus pale brown; setation mainly yellow, brown at tibial base and on tarsus. Hind leg mainly yellow, with basal 1/3 of tibia brown; setation mainly yellow, brown at the base of tibia and on tarsus. Tibial spurs pale amber. ***Wings*.** Forewing hyaline, longitudinal veins alternating brown and yellow, subcostal veinlets and crossveins brown; pterostigma yellow, with proximal and distal ends brown, veinlets pale brown; wing margin alternating yellow and brown. Hind wing mainly hyaline with an amber marking at base of first branch of CuA; pterostigma with proximal 1/3 and apex brown, the rest yellow. Proximal portion of C and C + Sc yellow, Sc brown, Cu predominantly yellow, anal veins yellow; remaining longitudinal veins alternating brown and yellow; subcostal veinlets pale brown; crossveins brown except for 1m-cu yellow; wing margin alternating yellow and brown. ***Abdomen*.** Tergites with extensive posteromedial brown spot, area surrounding anterior margin yellow. Sternites mainly yellow. Pleural membrane yellow with brown areas.

***Morphology* (Fig. 85)**. ***Head*.** Semi-triangular, smooth, vertexal region slightly domed over compound eyes, with rows of short, reclined setae; coronal suture discernible; paraocular area concave. Compound eye hemispheric, as wide as ¾ of the interocular distance at torulus level. Antenna moniliform, scape slightly longer than wide, slightly distally expanded, with thin and short setae; pedicel slightly longer than wide; flagellum slightly dorsoventrally flattened, basal flagellomeres ~ 2× as wide as long, progressively narrower towards the apex, all articles with a medial ring of thickened setae. Frons trapezoidal and narrow; clypeus rectangular, narrow. Postgena broad; hypostomal bridge not fused to postgena. Labrum rounded; maxillary palpus with first two palpomeres short, third and fifth 4× as long as wide, fourth palpomere 3× as long as wide. Labial palpus with third palpomere expanded and rounded; palpimacula broadly ovoid. ***Thorax*.** Pronotum 1.5× as long as wide, with a groove contiguous to lateral and distal margins; posterior margin, medial area and anterior margin slightly raised with long and thickened setae arising flush the pronotal surface; episternum with long, thin setae; postfurcasternum trapezoidal. Mesonotum slightly wider than long, mesoscutum with abundant erect, long, thickened setae on medial area; scutellum with long, thickened setae. Metanotum ~ 2× as wide as long, scutum with convex lateral regions; metascutellum pentagonal, with the posterior area slightly raised. Pteropleura covered with abundant long, thin setae. ***Foreleg*.** Coxa cylindrical, almost as long as femur, with abundant fine, long setae. Trochanter subconical, setose, dorsally with thickened setae on distal margin. Femur robust, sub-cylindrical, setose; closing surface with rows of specializations fully developed; laterally with rows of thickened setae with globular base adjacent to the rows of processes. Tibia curved and shorter than the femur, setose, distal region of anterior surface with a patch of clavate setae, closing surface keeled, with a row of prostrate, flattened setae. Basitarsus elongated, basal ½ with clavate setae on anterior surface, ventrally keeled with prostrate setae; lanceolate process short, reaching the base of third tarsomere, with plug-shaped Stitz organ at apex; second tarsomere 4× as long as wide, third tarsomere slightly longer than wide, fourth tarsomere 2× as long as wide. ***Mid- and hind leg*.** Mid-leg covered with abundant fine, long setae; tibia slightly flattened, thin; basitarsus ~ 4× as long as it is wide, second slightly longer than wide, third and fourth tarsomeres as long as wide; fifth tarsomere ~ 3× as long as wide; first four tarsomeres with a pair of thickened setae laterally on the distal margin of plantar surface. Hind leg densely covered with long, thin setae, shorter on tarsus; tibia slightly thickened; tarsomeres similar to those of the mid-leg. ***Wings*.** Forewing oval, venation setose, trichosors present along wing margin except at wing base; costal space narrow, humeral vein forked; 12 or 13 subcostal veinlets present, of which some are forked; pterostigma approximately rectangular, narrow, smoothly curved, with numerous veinlets mainly simple and incomplete; subcostal space with a single medially located crossvein; Sc vein abruptly bent posteriad at proximal margin of pterostigma to merge the RA; *rarp2* gently curved, three RP branches arising from each anterior radial cell; M fused basally to RA; base of RP located near separation of M and R, M fork near such separation; 1r-m located between RP base and MA base, forming a small trapezoidal cell; five or six crossveins present. Cu vein deeply forked, CuP proximally angled, touching A1, distally bifurcated at level of divergence of M and R. 1A simple ending almost at level of separation of M and R; 2A distally forked, slightly beyond the level of CuP angle; jugal lobe with abundant setae on posterior margin. Hind wing oval, smaller and narrower than forewing; costal space narrow and reduced, with four or five veinlets; C and Sc fused at proximal ¼ of wing length, subcostal space without crossveins, Sc vein abruptly curved posteriad at proximal margin of pterostigma to merge the RA; pterostigma elongated and narrow; radial space widened with single crossvein; three veins arising from *rarp1*, one or two from *rarp2*. 1r-m sigmoid, connecting the base of M and base of RP, distally connected again to M stem through short crossvein. M vein forked slightly beyond R fork. Cu vein deeply forked, CuA long, sinuous, distally forked, first branch generally candelabrum-shaped; CuP distally anteriorly curved, pectinate. A1 arched; A2 simple, short, and arched. ***Abdomen*.** Cylindrical, tergites quadrangular with scattered setae; intertergal membranes between abdominal segments III and IV widened; sternites rectangular.

**Figure 86.**
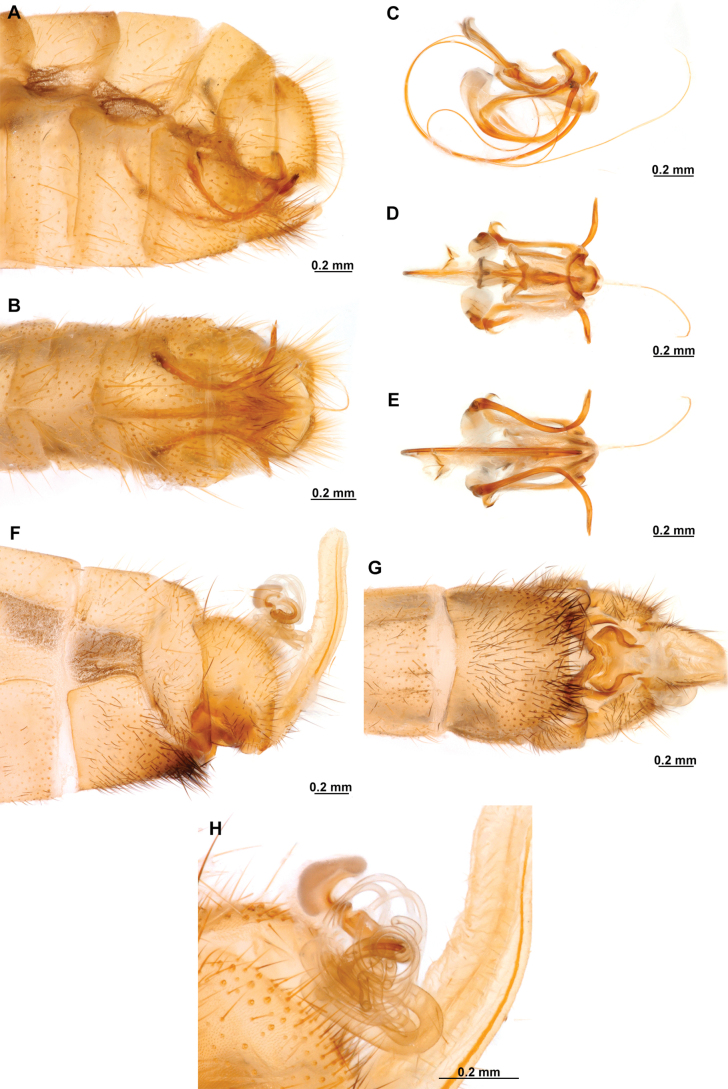
*Trichosceliabasella* (Westwood, 1867) **A** male terminalia, lateral **B** same, ventral **C** male genitalia, lateral **D** same, dorsal **E** same, ventral **F** female terminalia, lateral **G** same, ventral **H** spermatheca.

***Male genitalia*** (Fig. 86A–E). Tergite IX narrower dorsally than laterally, posteroventrally notched. Sternite VIII rectangular. Sternite IX triangular, setose, apex acuminate and slightly curved ventrally, dorsal surface canaliculate; in lateral view slightly exceeding the posterior margin of ectoproct. Gonocoxite IX short and notably curved, base spatulate, apex laterodorsally recurved, with five digitiform processes of different sizes, of which three are apical and medially located and two are preapical and lateral. Ectoproct approximately ovoid, moderately setose, ventrally with an anterior, semi-triangular, bulging area, followed by well-sclerotized posterior concave area, covered with microtrichia. Gonocoxites X forming a straight, elongated, ventrally canaliculate sclerite, anterior apex expanded; posterior apex bilobed, connected to gonostyli X, two short, preapical, lateroventral processes are connected to gonapophyses X with a membrane. Gonostyli X with base not thickened, curved, with two lateral processes; the rest of the structure abruptly anteroventrally curved, long, anteriorly coiled, forming two internal loops before protruding from abdomen. Gonapophyses X rod-shaped, long, straight, posterior apex dorsally bent, with surrounding membrane set with microspinules; gonapophyses joined by a membrane covering the base of gonostyli X. Gonocoxites XI U-shaped; median lobe enlarged and elaborated, composed of two differentiated parts; dorsal part as a rounded anterodorsally projected lobe; ventral part as a rounded, convex area covered with minute spinules; ventral margin U-shaped; area between these parts less sclerotized. Lateral arms of gonocoxites XI short, straight with anterior apex expanded and bent laterally.

***Female genitalia*** (Fig. 86F–H). Sternite VII trapezoidal with posteromedial margin slightly concave, setose. Tergite VIII slightly narrower medially than laterally, enclosing the spiracle of the eighth abdominal segment, lateral margin blade-shaped. Membrane between tergite VIII and tergite IX + ectoproct sclerotized, forming a concave triangular plate. Gonocoxites VIII forming a trapezoidal plate, with two lateral concavities; gonapophyses VIII with medial part convex, keel-shaped, posteromedially bilobed, with two dorsally bent, rounded, lateral fins; lateral part of gonapophyses VIII as a small, oval, concave plate, which is connected to tergite IX + ectoproct. Tergite IX + ectoproct quadrangular. Gonocoxite IX long and narrow, as long as the last four abdominal segments together. Bursa copulatrix funnel-shaped, long membranous, with proximal region widened, sclerotized, and concave. Spermatheca complex and entangled; proximal section long, thin, forming four coils, terminating in an innermost spiral; medial section thicker than proximal section, entangled and forming multiple convolutions. Thin distal section, forming several convolutions, progressively wider towards the apex, distally with a trumpet-shaped invagination. Fertilization canal duct short, thin, sigmoid; fertilization canal short, thickened, C-shaped, covered with microfilaments.

##### Distribution.

Brazil (Rondônia), Bolivia (La Paz).

##### Remarks.

This species was synonymized with *T.iridella* by Penny (1982a), however after examining and comparing the holotypes of *T.basella* and *T.iridella* deposited in the Oxford University Museum and in the Natural History Museum of London it was concluded that: 1) *T.basella* is a distinct valid species and 2) the synonymy proposed by Penny (1982a) is herein invalidated. Furthermore, the species described as *T.iridella* by Penny (1982a) is actually *T.nassonovi*. After examining high resolution images of the paralectotype female of *T.iridella* deposited in the Oxford University Museum (OUMNH) it is concluded that it is a female of *T.basella*. The true *T.iridella* is a species known only from the lectotype male deposited in the Natural History Museum of London and will be described and diagnosed in its respective section below.

In view of the uncertainty of the records presented by Penny (1982a), the only valid records of *T.basella* are the ones provided herein which are from Brazil (Rondônia) and Bolivia (La Paz). The holotype of *T.basella* and the paralectotype of *T.iridella* have only “Amazonas” listed as type locality.

This species is markedly similar to *T.iridella* and *T.nassonovi*, as all of them have mostly or completely hyaline wings, however *T.basella* has a brown pterostigma with a wide orangish area. In addition, the strongly arched male gonocoxites IX set with five apical digitiform processes rapidly separates this species from the aforementioned ones.

#### ﻿﻿ Trichoscelia fenella

Taxon classificationAnimaliaNeuropteraRhachiberothidae

(Westwood, 1852)

Figs 87 , 88

Mantispa (Trichoscelia) fenella Westwood, 1852: 269. Holotype: male, Brazil, Pará (NHMUK), specimen examined.
Trichoscelis fenella (Westwood, 1852). Hagen (1861): 323.
Trichoscelia fenella (Westwood, 1852). Hagen (1866): 461.
Mantispa (Trichoscelia) egella Westwood, 1867: 502. Syntypes: female, Brazil, Amazonas (NHMUK), specimens examined.
Trichoscelia egella (Westwood, 1867). Gerstaecker (1888): 120. New synonym.
Anisoptera amoenula Gerstaecker, 1888: 119. Holotype: female, Brazil, Pará (ZIMG), photographs examined.
Trichoscelia amoenula (Gerstaecker, 1888). Penny (1977): 37.

##### Material examined.

***Holotype*** of *Trichosceliafenella*.

Brazil • ♂; **Pará**; 50/2; “*Mantispafenella* Westwood, Holotype male *Trichosceliafenella*Westwood 1852, R.G. Beard 1968”, Above label reads on overside 50/2; NHMUK 013802771; NHMUK.

***Lectotype*** of *Trichosceliaegella*.

Brazil • ♀; [**Amazonas**] Ega; *Trichosceliaegella* Westwood., NHMUK 013802769; syntype blue label; red type label; NHMUK. Note: Lectotype and paralectotype of *T.egella* are herein designated since Westwood (1867), did not explicitly designated a specimen as the holotype. The specimens deposited in the Natural History Museum, London and cited here were labeled as syntypes.

***Paralectotype*** of *Trichosceliaegella*.

Brazil • sex unknown; [**Amazonas**] Ega; *Trichosceliaegella* Westwood “perhaps one of the missing male types? Beard 1968”, “Abdomen missing”, Syntype (blue label); NHMUK 013802770; NHMUK.

***Holotype*** of *Anisopteraamoenula*.

Brazil • ♂; **Pará**, Itaituba; Hahnel leg.; *Trichosceliaamoenula* ♂ det. R. Hall (HNRS) 1988, genitalia prepared; II 27486; ZIMG.

##### Other material.

Brazil – **Amazonas** • 1 ?; Fonteboa; 1554; McLachLan leg.; B.M. 1938-674; NHMUK 013802777; *Trichosceliaegella* Westwood; Abdomen missing; NHMUK. • 1 ♀; same data as for preceding; BMNH(E) 1201810; NHMUK 013802776; NHMUK. 1 ♂; same data as for preceding; BMNH(E) 1201810; NHMUK 013802779; NHMUK. • 1 ♂; same data as for preceding; NHMUK 013802778; NHMUK. • 1 ♂; same data as for preceding; BMNH(E) 1201811; NHMUK 013802774; NHMUK. • 1 ♀; same data as for preceding; NHMUK 013802784; NHMUK. • 1 ♂; Ega; Bates leg.; McLachlan Coll. B.M. 1938-674; BMNH(E) 1201808; NHMUK 013802775; NHMUK. – **Mato Grosso** • 1 ♂; Nova Ubiratã, ESEC Rio Ronuro; 13°6'43.92"S, 54°26'36.96"W; 11–16 Jun. 2007; A.C. Domahovski leg.; sweep; DZUP. – **Rondônia** • 1 ♂; Vilhena; 19 Oct. 1986; C. Elias leg.; DZUP. • 1 ♂; Vilhena, POLONOROESTE; 9 Oct. 1986; C. Elias leg.; DZUP. • 1 ♀; same data as for preceding; 15 Oct. 1986; DZUP.

Colombia • 1 ♂; **Caquetá**, Florencia, vereda Paraíso, Finca Paraíso; 01°44.47N, 75°37.40W; 716 msnm; 15 Feb–01 Mar. 2017; Y. Ramos-Pastrana leg.; bosque secundário, trampa Malaise; LEUA. French Guiana – **Montsinery-Tonnegrande** • 1 ♀; Amazone Nature Lodge, 30 Km SE Roura on Kaw Rd; 04°33.570'N, 52°12.433'W; 305 m; 21–29 Jan. 2013; J.B. Heppner leg.; FSCA. – **Roura** • 1 ♀; Montagne des Chevaux, Carriere du Calion, Crete avec foret sur quartzite érodée; 4°44'31.54"N, 52°25'53.02"W; 20 Oct. 2018; charaxes trap; CSCA. • 1 ♀; same data as for preceding; 03 Jan. 2015; flight interception trap; CSCA. • 1 ♀; same data as for preceding; 05 May. 2015; flight interception trap; CSCA. • 1 ♂; same data as for preceding; 08 Aug. 2018; Malaise; CSCA. • 1 ♀; same data as for preceding; 24 Sep. 2016; Malaise; CSCA.

Suriname • 1 ♀; s/ Para Weq Zanderij, Krabba; 01 Nov. 1964; in wasp nest, *Polybiaoccidentalis* var. Platyceph., Geijskes; *Trichosceliaegella* det. L. Stange, 1995; FSCA.

##### Diagnosis.

This species is distinguished from other in the genus by having the head mostly brown, the pronotum varies from nearly completely orange, orange with anterior ½ brown-infuscate to uniformly brown. On the forewing, the pterostigma is brown with apex orange, the anterodistal region is orange, whereas the wing apex is brown. On the wing membrane, there is an amber spot on the area adjacent to the proximal margin of pterostigma and 1ra-rp, which is variable in size. A second spot on the MP fork may be present or absent, and when present can be from faint to well-marked and similar to the anterior spot. On the male genitalia, the gonocoxite IX is short and gently curved, whit posterior apex laterally recurved, equipped with six or seven digitiform processes, arranged as a brush. On the female genitalia, the medial part of the gonapophyses VIII forms a broad U-shaped canal, which is distally bilobed; the lateral part appears as a concave plate with bulging ventral area.

##### Description.

***Measurements*.** Male (*n* = 4). Forewing length: 7.0–7.9 mm; Hind wing length: 5.2–5.8 mm. Female (*n* = 4): Forewing length: 6.3–7.8 mm; Hind wing length: 4.8–5.8 mm.

**Figure 87.**
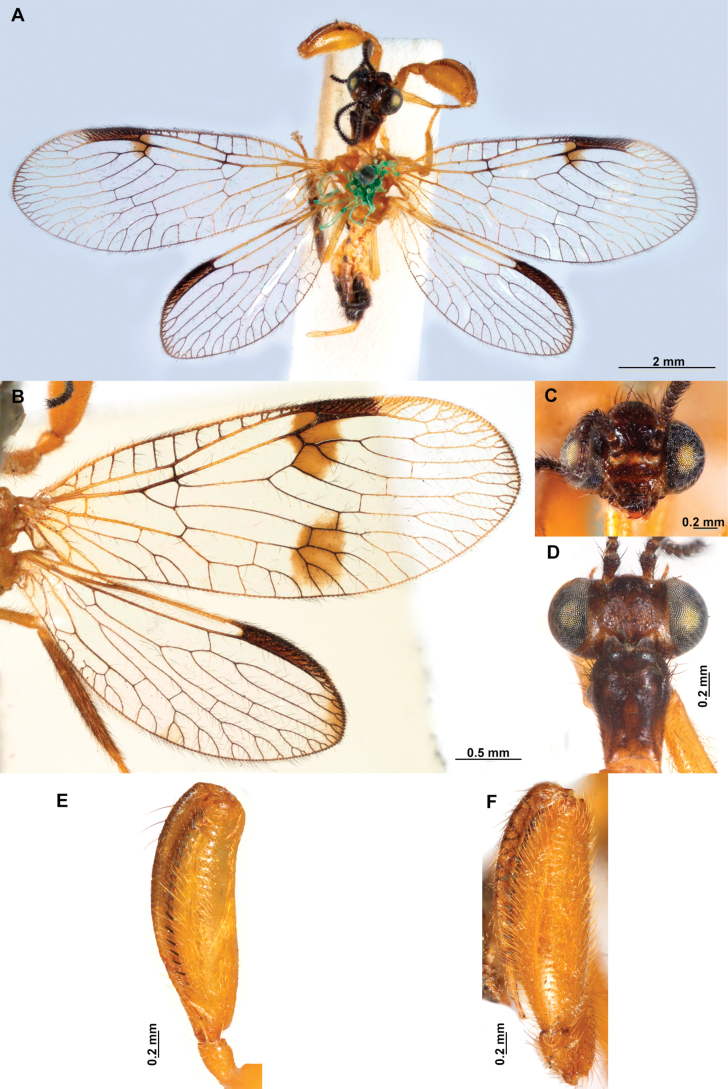
*Trichosceliafenella* (Westwood, 1852) **A** male habitus, dorsal **B** wings **C** head, frontal **D** pronotum, dorsal **E** forefemur, anterior surface **F** same, posterior surface.

***Coloration* (Fig. 87)**. ***Head*.** Mostly brown; vertexal region sometimes dark brown, paraocular area paler; frons with pale to dark brown setae. Antenna uniformly dark brown. Postgena either uniformly brown or pale brown with a darker longitudinal band; hypostomal bridge pale brown. Clypeus brown; labrum sometimes darker; mandible brown at base, pale amber at apex; maxilla orange with brown palpus, last palpomere pale er; postmentum pale brown, prementum orange, ligula orange to pale brown, palpus brown, palpimacula pale brown. ***Thorax*.** Pronotum mostly dark brown, posterolaterally gradually changing to orange, setae with same color as cuticle; episternum brown with concolorous setae; postfurcasternum orange. Mesonotum uniformly orange with pale brown setae; metanotum uniformly orange. Pteropleura uniformly orange. ***Foreleg*.** Coxa, trochanter and femur uniformly orange; tibia mostly pale brown, with orange apex; basitarsus orange, remaining tarsomeres orange with pale brown setae. ***Mid- and hind leg*.** Mid-leg orange; first four tarsomeres with pale brown setae laterally on the distal margin of plantar surface. Hind leg with coxa, trochanter and femur orange; tibia brown with orange base and apex; tarsus as in mid-leg. ***Wings*.** Forewing hyaline, with an amber area distally on subcostal field and area surrounding the 1ra-rp; sometimes with an amber mark on area surrounding MP posterior branch or a broad spot on area surrounding intramedial crossvein and MP fork; pterostigma either completely brown or with proximal ¾ brown and orange apex; venation as follows: medial area of ​​C, subcostal veinlets on distal ½ of costal field, 1sc-ra, preapical region of RA, 1ra-rp, preapical region of RP and proximal ½ of its branches and most of M vein brown, the rest orange. Hind wing hyaline, sometimes with apex amber, pterostigma brown; wing base, and most of C + Sc and RA orange; branches of remaining veins brown; wing margin brown. ***Abdomen*.** Abdominal segments I–V orange, segment VI bicolor, terminal segments brown.

***Morphology* (Fig. 87)**. ***Head*.** Diamond-shaped in frontal view, moderately setose, smooth, vertexal region domed above compound eyes, laterally with rows of short, reclined setae; coronal suture not discernible; paraocular area concave. Compound eye hemispheric, as wide as interocular distance at toruli level. Antenna moniliform; scape ~ 2× as long as wide, slightly distally expanded, pedicel slightly longer than wide, both with thickened setae; flagellum dorsoventrally compressed, with 41–44 flagellomeres, 2× as wide as long, progressively narrower towards the apex, all with medial ring of thickened setae. Frons pentagonal, narrow; postgena broad; hypostomal bridge not fused to postgena. Clypeus trapezoidal, narrow; labrum rounded. Maxillary palpus with first two palpomeres short, third and fifth 3× as long as wide, fourth 2× as long as wide; labial palpus with third palpomere expanded and rounded; palpimacula broadly ovoid. ***Thorax*.** Pronotum slightly longer than wide, with a groove contiguous to the lateral and distal margins; posterior margin, medial area and anterior margin slightly elevated with long, thickened setae limiting flush on pronotal surface; episternum with long, thin setae; postfurcasternum trapezoidal. Mesonotum as wide as long, mesoscutum with abundant erect, long, thickened setae on medial area; scutellum with long, thickened setae. Metanotum ~ 2× as wide as long, with two lateral cusps on scutum. Pteropleura covered with abundant short, thin setae. ***Foreleg*.** Coxa as long as femur, cylindrical, with abundant fine, long setae; trochanter subconical, densely setose, dorsally with longer and thicker, pedicellate setae near distal margin; femur thickened, sub-cylindrical, with abundant fine, long setae. Closing surface with both rows of integumentary specializations fully developed; posteroventral row proximally with one or two more developed processes (ratio process/seta 1:1), anteroventral row with a more developed sub-basal process (ratio process/seta 1:1); rows of thickened setae with globular base fully developed, extending almost the entire length of closing surface. Tibia almost as long as femur, curved, setose, closing surface keeled with prostrate setae, anterior surface apical patch of clavate setae. Basitarsus elongated, ventrally keeled and with single row of prostrate setae, anterior surface with clavate setae on proximal ½, lanceolate process short, reaching the base of fourth tarsomere, with plug-shaped Stitz organ at apex; second tarsomere 3× as long as wide, third tarsomere as long as wide, fourth tarsomere as long as second. ***Mid- and hind leg*.** Mid-leg covered with abundant, long, fine setae, tibia slightly flattened and medially widened; basitarsus ~ 4× as long as wide, third and fourth as long as wide, second slightly longer than wide; fourth tarsomere ~ 2× as long as wide, first four tarsomeres with a pair of lateral, thickened setae on the distal margin of plantar surface. Hind leg densely covered with long thin setae, shorter on tarsus, tibia slightly thickened and longer than femur; tarsomeres similar to those of the mid-leg. ***Wings*.** Forewing oval, venation densely setose, trichosors present along wing margin except at base of posterior margin; costal space narrow, humeral vein forked, 11–13 subcostal veinlets present; pterostigma approximately rectangular, smoothly curved, with numerous incomplete veinlets; subcostal space with a single medially located crossvein, Sc vein abruptly bent posteriad at the proximal margin of pterostigma to merge the RA; *rarp2* gently curved, two or three veins arising from each anterior radial cell; M fused basally to R; base of RP located near the separation of M and R, M fork near such separation; 1r-m located between RP base and M fork forming a small, trapezoidal cell; three gradate crossveins present. Cu deeply forked, CuA forked opposite to RP; CuP proximally angulated, almost touching A1, distally forked at level of divergence of M and R. 1A simple ending almost at level of separation of M and R; 2A distally forked, at level of CuP angle. Jugal lobe with abundant setae on the posterior margin. Hind wing oval, smaller and narrower than forewing, venation densely setose; costal space narrow and reduced, with three or four veinlets; C and Sc fused at proximal ¼ of wing length, subcostal space without crossveins, progressively widened towards apex, Sc vein abruptly curved posteriad at proximal margin of pterostigma to merge the RA; pterostigma elongated and narrow; area between RA and RP widened with single oblique crossvein; two veins arising from *rarp1*, two or three from *rarp2*. 1r-m sigmoid, connecting M base and RP base, distally connected again to M stem through a short crossvein. M vein forked beyond the level R fork. Cu vein deeply forked, CuA long, sinuous, distally forked, first branch candelabrum-shaped; CuP with apex anteriorly curved with two or three branches. A1 arched; A2 short and simple. ***Abdomen*.** Cylindrical, slightly medially expanded; tergites quadrangular, those of segments III and IV with posteromedial concavity; sternites quadrangular.

**Figure 88.**
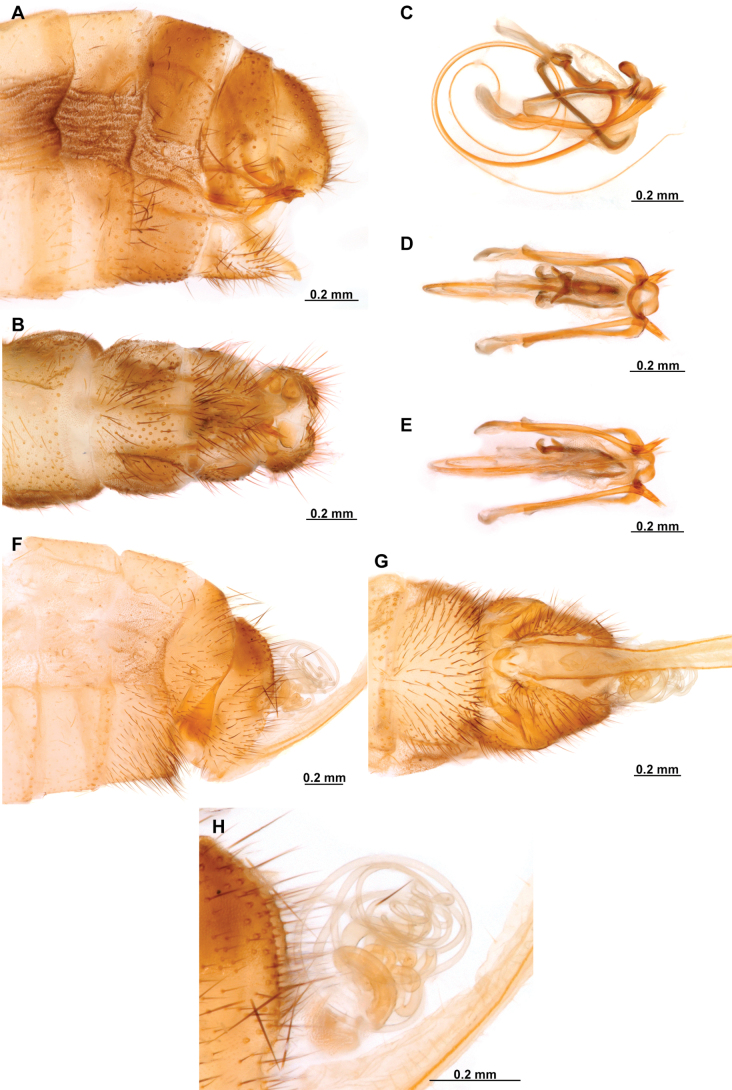
*Trichosceliafenella* (Westwood, 1852) **A** male terminalia, lateral **B** same, ventral **C** male genitalia, lateral **D** same, dorsal **E** same, ventral **F** female terminalia, lateral **G** same, ventral **H** spermatheca.

***Male genitalia*** (Fig. 88A–E). Tergite IX narrower dorsally than laterally, lateral margin triangular, with a posteroventral notch. Sternite VIII rectangular. Sternite IX approximately triangular with abundant long, thin setae; posteromedially blunt, glabrous, dorsally canaliculated; in lateral view tongue-shaped, slightly curved ventrally and nearly reaching or surpassing posterior margin of ectoproct. Gonocoxite IX short, gently curved, base flattened; apex laterally recurved, with six or seven digitiform processes, arranged as a brush. Ectoproct approximately ovoid, moderately setose, anteroventral area bulging and rounded, followed by posterior concave area; both well sclerotized and covered with microtrichia. Gonocoxites X forming a short, straight, ventrally canaliculate sclerite, anterior apex slightly expanded; posterior apex with preapical, ventrolateral processes connected. Gonostyli X with thickened, straight base and with two lateral processes; the rest of the structure abruptly anteroventrally curved and anteriorly coiled forming two internal loops before protruding from abdomen. Gonapophyses X rod-shaped, straight, anterior apex spatulate, dorsolaterally curved, posterior end dorsally curved with surrounding membrane covered with microspinules; gonapophyses arranged in a V-shaped structured, joined by a membrane covering the base of gonostyli X. Gonocoxites XI U-shaped, median lobe elaborated, consisting of two differentiated parts: dorsal part as a quadrangular lobe; ventral part as a wide, posteroventral, convex region whose surface is covered with microspinules; ventral margin V-shaped; area between these parts less sclerotized. Lateral arms of gonocoxites XI short, curved with anterior apex curved.

***Female genitalia*** (Fig. 88F–H). Sternite VII sub-quadrate. Tergite VIII medially narrower than laterally, enclosing spiracle of eighth abdominal segment. Membrane between tergite VIII and tergite IX+ ectoproct sclerotized, forming a concave triangular plate. Gonocoxites VIII forming a trapezoidal plate, with two lateral concavities; gonapophyses VIII with medial part forming a broad U-shaped canal, distally bilobed; lateral part of gonapophyses VIII as a concave plate with bulging ventral area. Tergite IX + ectoproct semi-triangular, with rounded posterior margin. Gonocoxite IX long, straight and narrow, as long as the last four abdominal segments together. Bursa copulatrix funnel-shaped, membranous, long, and thin. Spermatheca complex and entangled; proximal section long, thin, with five or six coils; medial section entangled, but mostly wider than proximal section, forming multiple convolutions, slightly wider proximally, and progressively thinner. Distal section progressively widened, distally with a trumpet-shaped invagination. Fertilization canal duct short, sigmoid; fertilization canal short, thickened, J-shaped, covered with microfilaments.

##### Distribution.

Brazil (Amazonas, Mato Grosso, Pará, Rondônia), Colombia (Caquetá), French Guiana (Montsinery-Tonnegrande, Roura), Suriname.

##### Remarks.

The label record of the type of *Anisopteraamoenula* is from Itaituba, Pará, and the syntypes of *T.egella* were collected in Tefé (before Ega), Amazonas, Brazil. Other specimens from the Natural History Museum of London were collected in Fonte Boa, Amazonas, Brazil. The holotype of *T.fenella* was collected in Pará. Here the first records of this species from French Guiana, Colombia and Suriname are provided.

After a careful examination and comparison of the types of *T.egella* and *T.fenella*, it was concluded that both are the same species. The former is herein synonymized with *T.fenella*. The differences in the coloration of the wing veins and on the spots on the membrane are explained as intraspecific variation. The spots located on the adjacent area to the proximal pterostigma margin and on the MP fork vary from absent or faint to strongly marked. Additionally, the male genitalic morphology of both species was found to be identical.

#### ﻿﻿ Trichoscelia flavomaculata

Taxon classificationAnimaliaNeuropteraRhachiberothidae

Ardila-Camacho & Contreras-Ramos sp. nov.

https://zoobank.org/BBBEA554-5F31-40DA-BB9F-9915F3346DEE

Figs 89 , 90

##### Type locality.

Panama, **Panamá**: Nusagandi area, Cordillera Igar (trail), 19 May. 1993, E. Riley leg.

##### Material examined.

***Holotype*** male, pinned. Original label: “Panama, **Panamá**, Nusagandi area, Cordillera Igar (trail), 19 May. 1993, E. Riley leg., TAMU-ENTO X0071158” TAMU. ***Paratypes*.** Costa Rica • 1 ♂; [**Puntarenas**]; Golfito; 07 Aug. 1957; A. Menke leg.; FSCA.

Panama – **Canal Zone** • 1 ♂; Ft. Clayton; Apr. 1944; K.E. Friek leg.; CAS. – [**Panama Oeste**] • 1 ♂; Barro Colorado Island; 10–17 May. 1964; WD & SS Duckworth leg.; USNMENT01541906; USNM. • 1 ♀; Barro Colorado Island; 07 Jul. 1978; E.M. Fisher leg.; CAS. • 1 ♀; Barro Colorado Island; 29 Apr. 1937; S.W. Frost leg.; USNMENT01541907; USNM.

##### Other material.

COSTA RICA – **Guanacaste** • 1 ♀; Macizo Miravalles Estación Cabro Muco; 1100 m; M. Zumbado leg.; Mercury Light; L-N, 299769, 411243, 73960, INB0003720495, INBIOCRI; MNCR. – **Heredia** • 1 ♀; Est. EL Ceibo, Braulio Carrillo, N.P. Heredia; 400–600 m; Nov. 1989; M. Zumbado leg.; 527700, 256500, 500, CRI000134626; MNCR. • 1 ♂; same data as for preceding; CRI000134635; MNCR. • 1 ♂; 11 km SE la virgen; 450–550 m; 24 Feb. 2003; INBIO-OET-ALAS transect, INB0003622781, 10–24.II.2003 05-RVC-001 Transect; MNCR. • 1 ♀; Est. Biol. La Selva; 10°26'N, 84°01'W; 150 m; 15 Mar. 1993; R. Vargas C. leg.; CRI002738969; MNCR. • 1 ♀; Pr. La Selva Biol. Sta. 3 Km S. Pto Viejo; 10°26'N, 84°01'W; 13 Aug. 1996; H.A. Hespenheide leg.; INB0003659646; MNCR. – **Limón** • 1 ♀; Circuito Reviente Pacho Espavel, R.B. Hitoy Cerere; 150–650 m; 12 Oct. 1993; G. Carballo & M. Zumbado leg.; L-S, 184200, 643300, 2377, CRI001932620; MNCR. • 1 ♂; Sector Cerro Cocorí, Finca de E. Rojas; 150 m; 10–30 Sep. 1992; E. Rojas leg.; L-N 286000, 567500, CRI000951335; MNCR. • 1 ♂; Sector Cerro Cocorí, Finca de E. Rojas; 150 m; 28 May–17 Jun. 1992; E. Rojas leg.; L-N, 286000, 567500, CRI000764149; MNCR. • 1 ♀; R.B. Hitoy Cerere. Est. Hitoy Cerere; 0–100 m; May. 2003; E. Rojas leg.; L-N, 184500, 643470, 86095, IMB0004011118, INBIOCRI; MNCR. • 1 ♀; [Limón?] La Lola; 09 Mar. 1958; M.J. Stelzer leg.; No. 58-1, USNMENT01541900; USNM. – **Puntarenas** • 1 ♂; Est. Sirena, P.N. Corcovado; 0–100 m; Apr. 1992; G. Fonseca leg.; L-S, 270500, 508300, CRI000794100; MNCR. • 1 ♀; P.N. Miguel Antonio Quepos; 80 m; Apr. 1992; L-N, 370900, 448800, 1181, CRI001718645; MNCR. • 1 ♀; P.N. Corcovado, Cerro Puma; 100–300 m; 19 Jun–08 Jul. 2003; M. Moraga, A. Azofeita, K. Caballero leg.; Malaise trap; L-S, 267700, 518900, 74501, INB0003736428, INBIOCRI; MNCR.

Panama – **Panama Oeste** • 1 ♀; Barro Colorado; Jun. 1940; J. Zetek leg.; No. 4669, Lot. No. 40-22219, USNMENT01541905; USNM. – [**Panamá**] • 1 ♀; Cabima; 18 May. 2011; A. Busck leg.; USNMENT01541902; *Symphrasisbanksi* Enderlein det. N. Banks; USNM.

##### Diagnosis.

This species is separated from *T.banksi* by having the forefemur less dark pigmented, generally nearly completely yellow, or with less extensive brown markings. On the male genitalia, the gonocoxites IX are narrow and elongated, with posterior apex slightly laterally projected and equipped with three apical and three or four preapical minute processes. On the female genitalia, the medial part of the gonapophysis VIII is arch-shaped, with bifid posteromedial projection.

##### Etymology.

The name of this new species is a combination of the Latin –*flavus*– meaning yellow and –*macula*– meaning spot or stain, in reference to the yellow spots on the body of this species. An adjective in the nominative case.

##### Description.

***Measurements*.** Male (*n* = 5). Forewing length: 7.27–8.25 mm; Hind wing length: 5.64–6.37 mm. Female (*n* = 9): Forewing length: 7.45–8.66 mm; Hind wing length: 6.07–7.06 mm.

**Figure 89.**
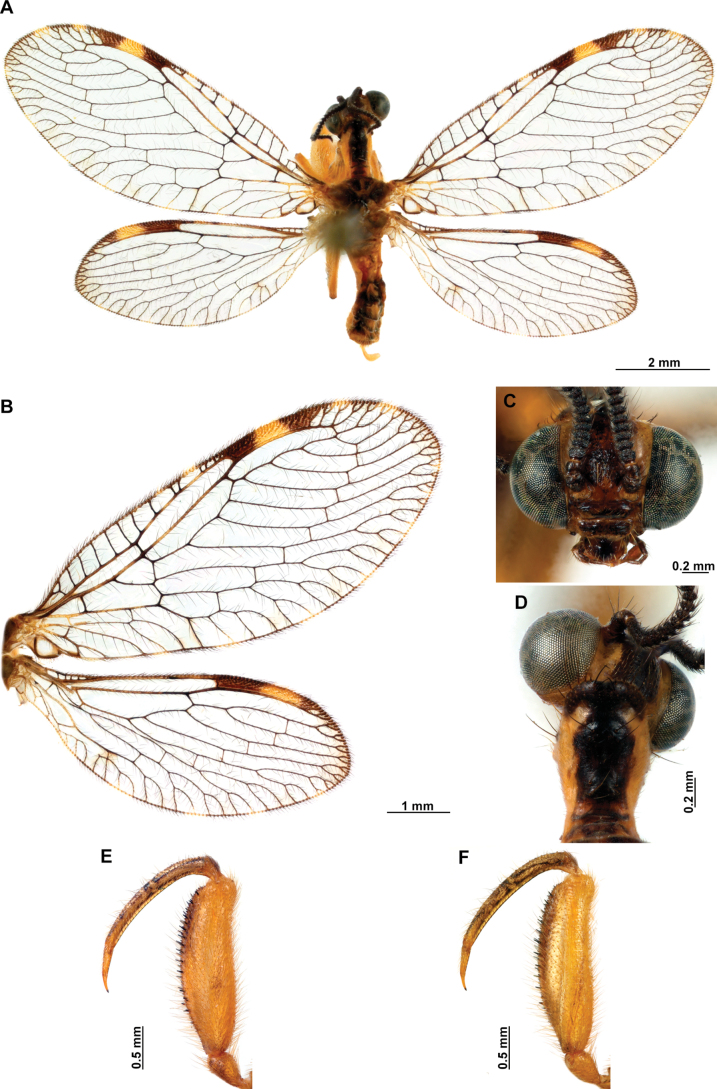
*Trichosceliaflavomaculata* Ardila-Camacho & Contreras-Ramos, sp. nov. **A** male habitus, dorsal **B** wings **C** head, frontal **D** pronotum, dorsal **E** forefemur, anterior surface **F** same, posterior surface.

***Coloration*** (Fig. 89). ***Head*.** Mainly yellow, vertexal region with longitudinal brown band extending from occipital ridge to supraantennal area; with pale brown or yellow setae. Antennal scape yellow at base, brown towards apex; pedicel and flagellum dark brown. Frons with brown transverse stripe; clypeus with brown transverse stripe and yellow margins; labrum brown; mandible pale brown; maxillary palpus dark brown; labium pale brown, palpus darker, palpimacula pale brown. ***Thorax*.** Pronotum yellow with brown longitudinal, medial band and anterior margin. Episternum bicolor, brown on anterior ½, yellow posteriorly; postfurcasternum yellow with brown suffusions. Meso- and metanotum pale brown, margins of sclerites yellowish brown; pteropleura yellow with brown spots. ***Foreleg*.** Pale brown, coxa with yellowish areas; trochanter yellowish brown. Femur either completely yellow or mostly yellow with pale brown areas on anterior and posterior surfaces; tibia brown with yellowish areas, apex with pale clavate setae. Basitarsus yellowish brown with dark brown apex; tarsomeres 2–4 pale brown. ***Mid- and hind leg*.** Mid-leg with coxa, trochanter and femur yellow, tibia with dark brown basal ¼, the rest of the surface yellow to pale brown; basitarsus yellowish brown, remaining tarsomeres brown. Hind leg with coxa, trochanter and femur yellow; tibia with basal ½ brown to dark brown, distal ½ yellow; tarsi as in mid-leg. ***Wings*.** Forewing hyaline, sometimes amber area surrounding first branch of CuA, and apex of CuP; pterostigma brown laterally and yellow or pale brown at center; venation mostly dark brown with yellow areas on C, Sc, RA, basal ½ of CuA, base of CuP, and base of anal veins. Some specimens with main veins alternating dark brown and yellow, except anal veins mostly yellow and branches of RP brown; posterior and distal margins alternating dark brown and yellow. Hind wing hyaline, base of subcostal space smoky; pterostigma brown with yellow or pale brown preapical area; main veins mostly brown with yellow areas in dry specimens; in fresh specimens, C + Sc yellow, alternating yellow and brown at base of RP and M, proximal ½ of CuA, most of CuP and anal veins yellow; posterior and distal margins alternating yellow and brown, some specimens with brown distal margin. ***Abdomen*.** Tergites mostly yellow with medial, longitudinal, brown stripe, extending laterally at the posterior margin, last tergites mostly yellow; tergites completely brown in pinned specimens; sternites yellow, except for the seventh segment of female dark brown; males with sternites VIII and IX pale brown; pleural membrane dark brown with yellow spots.

***Morphology*** (Fig. 89). ***Head*.** Semi-triangular in frontal view, sparsely setose, smooth, vertexal region slightly domed above compound eyes; paraocular area concave; coronal suture discrete. Compound eye hemispheric, as wide as ¾ interocular distance at torulus level. Antenna filiform, scape ~ 2× as long as wide; flagellum with 33–40 flagellomeres, those of basal ½ 2× as wide as long, progressively narrower towards apex; all flagellomeres with medial ring of thickened setae. Labial palpus with third palpomere expanded, palpimacula broadly ovoid. ***Thorax*.** Pronotum longer than wide, with groove contiguous to lateral and anterior margins; in lateral view anterior margin, medial region and posterior margin raised, with thickened, pedicellate setae. Mesonotum 1.5× as wide as long, with scattered, thick setae; metanotum 1.5× as wide as long, glabrous. Pteropleura covered with abundant fine, long setae. ***Foreleg*.** Coxa slightly shorter than femur, cylindrical, with abundant fine, long setae; trochanter subtrapezoidal; femur robust, with abundant fine, short setae; closing surface with double row of integumentary specializations fully developed; laterally to each row of processes a row of thickened setae with globular base present. Tibia almost as long as femur, curved, setose; closing surface keeled, with prostrate setae; anterior surface with patch of clavate setae at apex. Basitarsus elongated, equipped with clavate setae, proximally on anterior surface, ventrally keeled, with row of prostrate setae; lanceolate process reaching apex of third tarsomere, equipped with apical plug-shaped Stitz organ. ***Mid- and hind leg*.** Mid-leg covered with abundant fine, long setae, shorter on tarsus. Hind leg densely covered with long, thin setae, except on tarsus shorter. ***Wings*.** Forewing oval, trichosors present along wing margin except at base, venation setose; costal space narrow, humeral vein sometimes forked, 10–12 subcostal veinlets; pterostigma elongated and narrow, composed of numerous, mostly incomplete veinlets; subcostal space with single, medially located crossvein; Sc vein bent posteriad at proximal margin of pterostigma to merge the RA. *rarp2* gently curved with two or three veins arising from it, two or three from *rarp1*. 4–8 gradate crossveins present. M fused basally to R; RP base located near separation of M and R, M fork near such separation, 1r-m located between RP base and M base, forming a small trapezoidal cell; M fork opposite to R fork. Cu deeply forked, CuA long; CuP proximally angled, touching A1, distally forked, ending at posterior margin at level of R fork. A1 simple, ending at posterior margin at level of separation of M and R; A2 forked. Hind wing smaller and narrower than forewing; costal space narrow and reduced, with 4–6 veinlets; C and Sc fused at 1/5 of wing length; Sc vein curved posteriad at proximal margin of pterostigma to merge the RA. Pterostigma elongated, narrow, curved, composed of numerous, incomplete veinlets; radial space with single crossvein, oblique; two or three veins arising from *rarp1*, two from *rarp2*. M forked beyond R fork. Cu deeply forked, CuA, sinuous, long, distally forked, first branch candelabrum-shaped; CuP distally anteriorly bent, pectinate. Cubitoanal space with two crossveins. A1 arched, A2 short and arched. ***Abdomen*.** Medially widened, tergites subquadrate; sternites rectangular.

**Figure 90.**
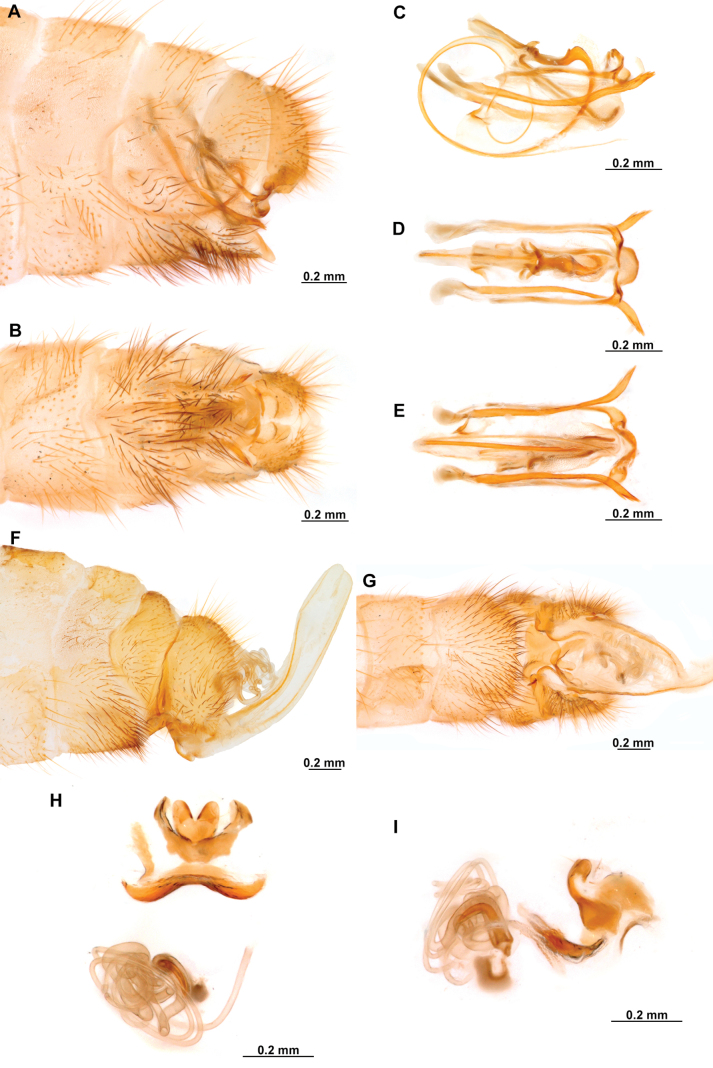
*Trichosceliaflavomaculata* Ardila-Camacho & Contreras-Ramos, sp. nov. **A** male terminalia, lateral **B** same, ventral **C** male genitalia, lateral **D** same, dorsal **E** same, ventral **F** female terminalia, lateral **G** same, ventral **H** gonapophyses VIII ventral **I** spermatheca.

***Male genitalia*** (Fig. 90A–E). Tergite IX dorsally narrower than laterally, lateral margin rounded, posteriorly notched. Sternite VIII rectangular. Sternite IX pentagonal, setose, posteromedially forming an obtuse angle; in lateral view acute reaching posterior margin of ectoproct. Gonocoxites IX narrow, elongated, gently curved; base spatulate; apex slightly laterally projected with three apical and three or four preapical minute processes. Ectoproct ovoid, setose; ventral surface with anterior area convex, covered with microtrichia, posteriorly continued by concave area continuous ventromedially with sclerotized sulcus. Gonocoxites X forming a short, straight, ventrally canaliculate sclerite; anterior apex slightly expanded; posterior apex bilobed, and with two preapical, lateroventral processes. Gonostyli X with straight and thin base, with two lateral processes, the rest of the structure ventrally curved, anteriorly coiled, forming two loops before reaching sternite IX. Gonapophyses X straight, narrow, posterior apex dorsally bent, with surrounding membrane set with microspinules; gonapophyses subparallel, joined by membrane covering base of gonostyli X. Gonocoxites XI thin, U-shaped, medial lobe elaborated, with two differentiated parts: dorsal part as an arch; ventral part a convex area covered with microspinules, ventral edge incised V-shaped; between these parts a narrow, less sclerotized area present. Lateral arms of gonocoxites XI straight.

***Female genitalia*** (Fig. 90F–I). Tergite VIII narrower dorsally than laterally, enclosing spiracle of eighth abdominal segment, lateral margin triangular. Sternum VII trapezoidal in ventral view. Gonocoxites VIII as a trapezoidal plate, with two lateral concavities; gonapophysis VIII medial part arch-shaped, with bifid posteromedial projection; lateral part as triangular, convex plate. Membrane between tergite VIII and tergite IX + ectoproct sclerotized, concave, triangular. Tergite IX + ectoproct trapezoidal. Gonocoxite IX elongated, sinuous and narrow, as long as the last four abdominal segments combined. Bursa copulatrix funnel-shaped, long, with proximal region sclerotized and concave, the rest membranous. Spermatheca entangled; proximal section long, thin, forming two coils, ending in a more internal spiral; medial section entangled, wider than proximal section; distal section progressively widened towards apex, slightly wider than medial section, distally forming a trumpet-shaped invagination; fertilization canal duct narrow, short, sinuous; fertilization canal J-shaped, covered with microfilaments.

##### Distribution.

Costa Rica (Guanacaste, Heredia, Limón, Puntarenas), Panama (Canal Zone, Panamá).

##### Remarks.

This species is known from Central America (Costa Rica and Panama). It is difficult to separate from *T.banksi* and *T.andina* as all of them have a very similar body color pattern and overlap their distribution ranges in Central America. However, *T.flavomaculata* is generally paler with extensive yellow areas; the forefemur is typically completely yellow while in *T.banksi* and *T.andina* it exhibits noticeable brown marks. The male gonocoxite IX of this species is similar to that of *T.andina*, although in *T.flavomaculata* it is narrower and the median lobe of the gonocoxites XI lack a prominent ventral lobe.

#### ﻿﻿ Trichoscelia geraldoi

Taxon classificationAnimaliaNeuropteraRhachiberothidae

Machado, 2018

Figs 91 , 92

Trichoscelia geraldoi Machado, 2018: 186. Holotype: male, Brazil, Amazonas (INPA).

##### Material examined.

French Guiana – **Camopi** • 1 ♀; Mont Saint-Marcel, Mont St.-Marcel, Inselberg culminant án; 635 m; 2°23'29"N, 53°0'50"W; 10 Mar. 2017; light trap; CSCA. –[**Montsinéry-Tonnegrande**] • 3 ♂; 5 Km E Tonnegrande; 04°48.176'N, 52° 25.525'W; 45 m; J.E. Eger leg.; MV light; FSCA. – **Roura** • 1 ♀; Montagne des Chevaux, Carriere du Galion, Créte avec forét sur quartzie érodee; 4°44'31.54"N, 52°25'53.02"W; 01 Sep. 2018; charaxes trap; CSCA. – [**Saint-Laurent-du-Maroni**] • 1 ♀; Paul Isnard, “Citron”; 8–18 Mar. 1986; G. Tavakilian leg.; TAMU-ENTO X0752761; TAMU.

##### Diagnosis.

This species is similar to *T.vespiformis* in the coloration pattern, being mostly blackish brown with basal abdominal segments whitish or cream. On the forewing, the membrane surrounding 1sc-ra, distal region of subcostal field, RP stem, 1ra-rp, base of RP branches of first anterior radial cell, and area adjacent to M fork is amber. On the male genitalia, the gonocoxite IX is long and arched, thickened, with posterior apex equipped with five or six digitiform processes. On the female genitalia, the medial part of the gonapophyses VIII is composed by two trapezoidal, medially joined plates forming a keel, posteromedially set with a bilobed process. The lateral part of gonapophyses VIII is a trapezoidal, convex plate, covered with microtrichia.

##### Description.

***Measurements*.** Male (*n* = 3). Forewing length: 10.0–10.8 mm; Hind wing length: 7.6–7.8 mm. Female (*n* = 1): Forewing length: 10.6 mm; Hind wing length: 8.0 mm.

**Figure 91.**
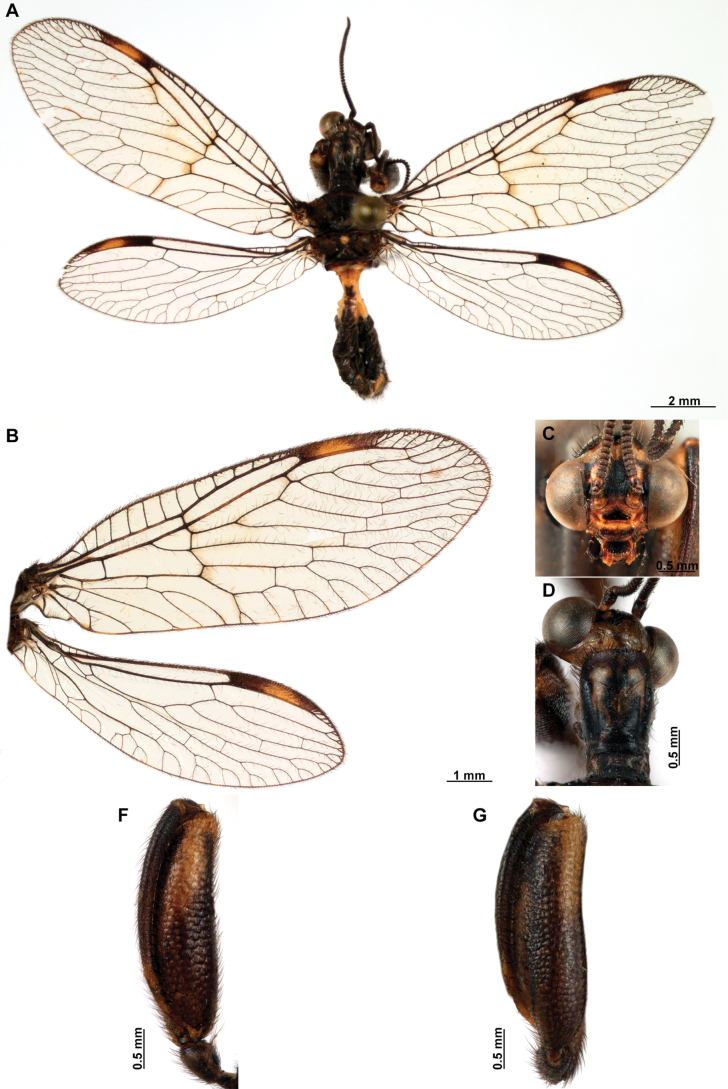
*Trichosceliageraldoi* Machado, 2018. **A** male habitus, dorsal **B** wings **C** head, frontal **D** pronotum, dorsal **E** forefemur, anterior surface **F** same, posterior surface.

***Coloration*** (Fig. 91). ***Head*.** Mainly ocher, vertexal region with a large dark brown area. Antennal scape with ocher base, the rest dark brown, pedicel and flagellum dark brown. Frons with brown triangular mark, clypeus brown with ocher posterior margin. Hypostomal bridge with dark brown suffusions. Labrum dark brown, paler, towards lateral margins; mandible dark brown with ocher base; maxilla ocher, with dark brown palpus; labium ocher, submentum with brown medial area, setae pale brown palpus brown, palpimacula pale brown. ***Thorax*.** Pronotum dark brown, with a pair of pale lateral markings near anterior margin, setae dark brown; episternum bicolor, dark brown on the anterior ½, pale on posterior ½; postfurcasternum dark brown. Mesonotum dark brown; metanotum brown with pale yellow central area. Pre-episternum dark brown. Pteropleura dark brown, anepisternum with pale yellow ventral margin on both segments, setae predominantly dark brown. ***Foreleg*.** Coxa and trochanter dark brown; femur dark brown with ocher area on distal 1/3 on anterior and posterior surfaces; setae dark brown. Tibia brown, paler at base and apex; setae with same color as cuticle, clavate setae pale brown. Basitarsus brown, clavate setae pale; second to fourth tarsomere pale brown. ***Mid- and hind leg*.** Mid-leg with dark brown coxa and trochanter, femur dark brown with apical 1/3 ocher, tibia dark brown with ocher area at apex, tarsus pale brown; first four tarsomeres with dark brown setae at apex, laterally on plantar surface. Hind leg with brown coxa and trochanter, femur brown with ocher apex, tibia brown, tarsus as in the mid-leg. ***Wings*.** Forewing hyaline, sometimes with amber medial area, sometimes restricted to area adjacent to 1sc-r, R fork, 1r-m, M fork, 2m-cu and first branch of CuA; pterostigma dark brown with ocher medial area; longitudinal veins alternating dark and pale brown, subcostal veinlets and crossveins dark brown; wing margin alternating dark and pale brown. Hind wing hyaline; pterostigma brown with ocher preapical area; venation mostly brown; posterior and distal margin brown. ***Abdomen*.** Tergites of abdominal segments I–III pale yellow with a dark brown medial mark, tergites VII and VIII in male or only VII in female pale yellow with dark brown medial markings, tergites of remaining segments dark brown. Sternites of segments I–III pale yellow, the rest dark brown. Pleural membrane pale yellow in first three abdominal segments, dark brown in the rest.

***Morphology*** (Fig. 91). ***Head*.** Diamond-shaped in frontal view, moderately setose, smooth; vertexal region domed above compound eyes, paraocular area concave; coronal suture discrete. Postgena broad, hypostomal bridge not completely fused with it. Compound eye hemispheric, as wide as ¾ interocular distance at toruli level. Antenna moniliform, scape ~ 2× as long as wide, slightly distally expanded, with thin and short setae, thicker and longer on the ventral surface; pedicel as long as wide; flagellum not dorsoventrally compressed, with 46–49 flagellomeres, ~ 2× as wide as long on basal ½, the remaining 1.5× as wide as long, progressively smaller towards the apex; all flagellomeres with medial row of thickened setae. Maxillary palpus with first palpomere as long as wide, second slightly longer than wide, third palpomere 3× as long as wide, fourth 2.5× as long as wide, fifth palpomere as long as third. Submentum oval with long setae; labial palpus with first palpomere 2× as long as wide, second 3× as long as wide, third slightly longer than second, basal ½ expanded, palpimacula ovoid. ***Thorax*.** Pronotum 1.5× as long as wide, with a furrow contiguous to lateral and anterior margins; in lateral view anterior margin, medial region and posterior margin slightly elevated, with thickened, pedicellate setae; episternum with short, thin setae; postfurcasternum trapezoidal. Mesonotum almost as wide as long, with thick, pedicellate and on medial area of scutum and scutellum. Metanotum ~ 2× as wide as long as, mostly glabrous. Pteropleura covered with abundant short, thin setae. ***Foreleg*.** Coxa slightly shorter than femur, cylindrical, setose; trochanter subtrapezoidal with a protrusion on anterior surface; femur thickened, setose; closing surface with double row of tubercle-shaped specializations present, both with a more developed sub-basal process (process/seta ratio 1:1); rows of thickened setae with globular base, fully developed, extending over almost the entire closure surface. Tibia almost as long as the femur, curved, setose, closing surface with prostrate setae; with a patch of clavate setae apically on inner surface; basitarsus elongated, ventrally keeled, with single row of prostrate setae, anterior surface with patch of clavate setae; lanceolate process reaching the base of fourth tarsomere, with a plug-shaped Stitz organ at apex; second tarsomere 4× as long as wide, third tarsomere as long as wide, fourth tarsomere 2× as long as wide. ***Mid- and hind leg*.** Mid-leg with coxa and trochanter covered with interspersed long, short and thin setae; tibia and femur with interspersed long, short and thin setae; basitarsus 3.5 times as long as wide, second tarsomere slightly longer than wide, third and fourth as long as wide, fifth tarsomere ~ 2× as long as wide, all covered with short, thin setae; the first four tarsomeres with a pair of thickened setae laterally on the distal margin of plantar surface. Hind leg longer than mid-leg, setae mainly short and thin, some longer and thickened; tibia 1.5× as long as femur; tarsomeres to those of mid-leg. ***Wings*.** Forewing oval, trichosors present along wing margin except at base, venation setose; costal space only slightly widened on proximal ½; humeral vein simple, 12–15 subcostal veinlets; pterostigma elongated and narrow, composed of numerous, mainly incomplete veinlets; subcostal space with a single medially located crossvein; Sc vein bent posteriad at proximal margin of pterostigma to merge the RA; *rarp2* gently curved, with 2–4 veins arising from it, two or three from *rarp1*; M basally fused to R; RP base located near separation of M and R, M fork near such separation, opposite to R fork; 1r-m located between RP base and MA base, forming a small trapezoidal cell; four or five gradate crossveins present. Cu vein deeply forked, CuP basally angulated, approaching A1, distally forked, opposite to separation of M and R. 1A simple, reaching the level of separation of M and R; A2 distally forked, at level of CuP angle. Hind wing smaller and narrower than forewing; costal space narrow and reduced, with five or six veinlets; Sc and C fused at 1/5 of wing length, Sc curved posteriad a proximal pterostigma margin to merge the RA; pterostigma elongated, narrow, curved, composed of numerous incomplete veinlets; radial space with single crossvein, oblique; two veins arising from *rarp1*, one or two from *rarp2*. M vein forked beyond R fork; 1 r-m sigmoid, connecting M base and RP base, distally connected again to M stem through short crossvein. Cu deeply forked, CuA long and sinuous, distally forked, first branch candelabrum-shaped; CuP distally anteriorly bent, with two or three branches. Cubitoanal space with two crossveins. A1 arched, A2 short and arched. ***Abdomen*.** Cylindrical, tergites quadrangular; intertergal membranes between segments III–V in both sexes broad, covered with microtrichia, and with two glabrous lateral areas; sternites rectangular.

**Figure 92.**
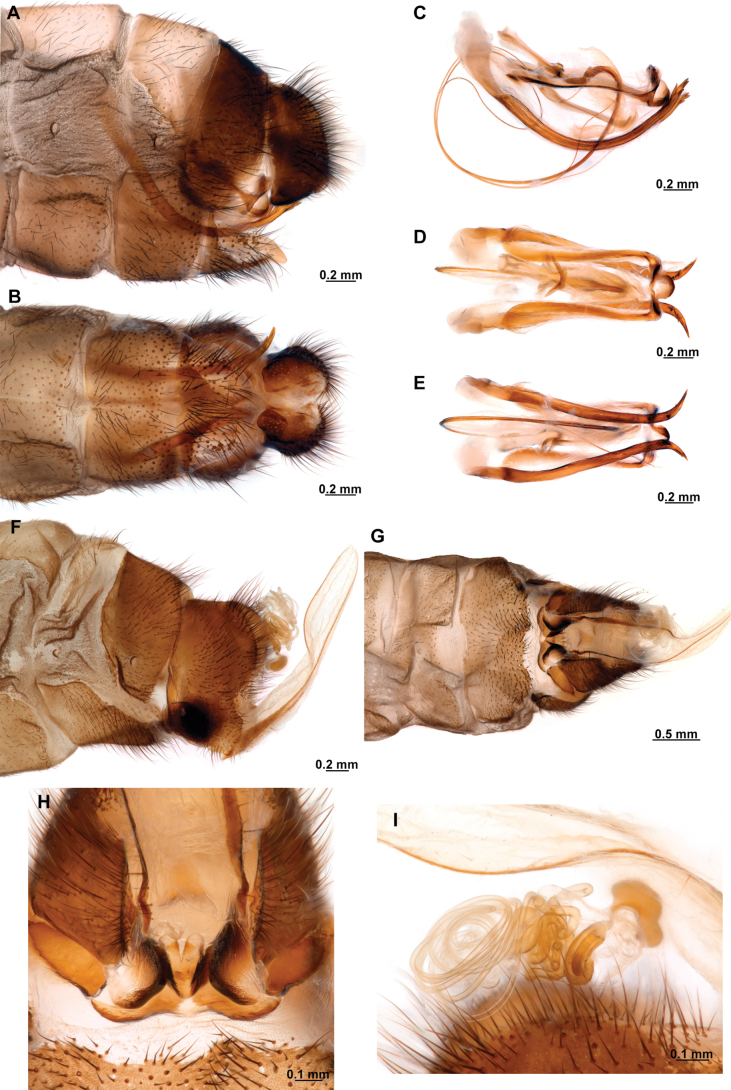
*Trichosceliageraldoi* Machado, 2018 **A** male terminalia, lateral **B** same, ventral **C** male genitalia, lateral **D** same, dorsal **E** same, ventral **F** female terminalia, lateral **G** same, ventral **H** gonapophyses VIII ventral **I** spermatheca.

***Male genitalia*** (Fig. 92A–E). Tergite IX slightly narrower medially than laterally, lateral margin rounded, with posteroventral notch. Sternite VIII rectangular; sternite IX pentagonal, setose, posteromedially forming an obtuse angle, glabrous, dorsally canaliculate; in lateral view acuminate, with the apex slightly bent and not reaching posterior margin of ectoproct. Gonocoxite IX long and arched, thickened, base spatulate, curved inwards; apex with five or six digitiform processes. Ectoproct trapezoidal, covered with abundant, short, thin setae; ventral surface with anterior, quadrangular, bulging area; posterior inner area concave, terminating in a fold, entire surface covered with microtrichia. Gonocoxites X forming a short, thickened, ventrally canaliculate sclerite; anterior apex expanded, posterior apex bilobed, two additional preapical processes are connected to gonapophyses X with a membrane. Gonostyli X with thickened, bent base, set with two lateral processes, the rest long, ventrally curved and anteriorly coiled, forming two internal loops before protruding from the abdomen. Gonapophyses X straight, thin, posterior apex dorsally bent, surrounding membrane set with microtrichia; gonapophyses arranged in a V-shaped structure, joined by a membrane covering the base of gonostyli X. Gonocoxites XI U-shaped, median lobe elaborated, with two differentiated parts; dorsal part as a rounded lobe; ventral part a convex area covered with microspinules; ventral margin with V-shaped incision; between these parts a narrow, less sclerotized region is present. Lateral arms slightly sinuous, anterior apex laterally curved.

***Female genitalia*** (Fig. 92F–I). Sternite VII trapezoidal. Tergite VIII almost as wide medially as laterally, enclosing the spiracle of the eighth abdominal segment, lateral margin trapezoidal. Membrane between tergite VIII and tergite IX + ectoproct sclerotized, forming a concave, triangular plate. Gonocoxites VIII forming a trapezoidal plate, with two lateral concavities; gonapophyses VIII with a medial part composed of two trapezoidal, medially joined plates forming a keel, posteromedially with a bilobed process; lateral part of gonapophyses VIII trapezoidal, convex, covered with microtrichia, and connected to tergite IX + ectoproct. Tergite 9 + ectoproct quadrangular. Gonocoxite IX long and narrow, as long as the last three abdominal segments together. Bursa copulatrix short, funnel-shaped, proximal region, sclerotized, concave, the rest membranous; spermatheca complex and entangled; proximal region long, thin forming six coils, ending in complex entanglement; medial section thicker than proximal section, entangled, forming multiple convolutions. Distal section thin, recurved, progressively wider towards the apex, distally with a trumpet-shaped invagination. Fertilization canal duct short, thin, sigmoid; fertilization canal short, thickened, J-shaped, covered with microfilaments.

##### Distribution.

Brazil (Amazonas), French Guiana (Camopi, Maripasoula, Montsinéry-Tonnegrande, Roura, Saint-Laurent-du-Maroni).

##### Remarks.

In the original description by Machado (2018), this species was recorded from Amazonas, Brazil and Maripasoula, French Guiana. Herein, new records from Camopi, Montsinéry-Tonnegrande, Roura, and Saint-Laurent-du-Maroni in French Guiana are provided.

This species has a general coloration pattern mimicking *Polybiadimorpha* Richards, 1978 (Hymenoptera: Vespidae) (R. Lopes, pers. Comm.). This same coloration is expressed by *T.vespiformis*, and both are evidently closely related. The distinction between both species needs the study of the genital sclerites, and both species can be differentiated by the overall shape and number of processes of the male gonocoxites IX. The female genitalia are also useful for the separation of both species, particularly the shape of the tergite IX + ectoproct and the presence of gonocoxites VIII.

#### ﻿﻿ Trichoscelia gorgonensis

Taxon classificationAnimaliaNeuropteraRhachiberothidae

Ardila-Camacho, 2015

Trichoscelia gorgonensis Ardila-Camacho, 2015: 424. Holotype: male, Colombia, Cauca (Gorgona Island) (MUSENUV).

##### Diagnosis.

This species is distinguished from its congeners because both wings have most of the CuA, CuP, and anal veins yellow, with the membrane between them yellow. On the male genitalia, the sternite IX with lateral margins rounded, and posteromedial process with apex blunt. The gonocoxite IX is long, equipped with three apical and two preapical digitiform processes.

Note: The type series designated by Ardila-Camacho and García (2015) was not available for the present study, so the diagnosis and the inclusion to the taxonomic key of the genus presented herein are based on the original description. For illustrations of diagnostic characters, plus detailed description of the morphology and coloration see Ardila-Camacho and García (2015).

##### Description.

See Ardila-Camacho and García (2015).

##### Distribution.

Colombia (Gorgona Island, Cauca).

##### Remarks.

*Trichosceliagorgonensis* is known only from its original description which was based on a series of specimens collected in the Gorgona island. This locality is situated 35 Km west to the Pacific coast of Colombia and belongs to the department of Cauca. This species is morphologically and probably closely related to *T.fenella*, *T.basella* and other Amazonian species since the overall morphology of the male gonocoxite IX is markedly similar in all of them.

#### ﻿﻿ Trichoscelia involuta

Taxon classificationAnimaliaNeuropteraRhachiberothidae

Ardila-Camacho & Contreras-Ramos sp. nov.

https://zoobank.org/00916BBE-92AF-4AE0-9054-D92581463CA3

Figs 93 , 94

##### Type locality.

Colombia, **Meta**: San Martín, Reserva Rey Zamuro-Mataredonda; 3°30'47"N, 73°23'10.69"W; 243 m; 20 May. 2023; M.N. Espitia leg.

##### Material examined.

***Holotype*** male, pinned, genitalia stored in a separate vial. Original label: “Colombia, **Meta**, San Martín, Reserva Rey Zamuro-Mataredonda; 3°30'47"N, 73°23'10.69"W; 243 m; 20 May. 2023; M.N. Espitia leg.” UNAB. ***Paratype*.** Brazil • 1 ♂; **Rondônia**, 62 Km SW Ariquemes, nr Fzda. Rancho Grande, 30 Mar.–10 Apr. 1992, J.E. Eger leg.; FSCA.

##### Diagnosis.

This species is separated from *T.umbrata*, because the forefemur has a dark-brown honey-comb pattern of markings, extending from dorsal surface to anterior and posterior surfaces. The region of the vertex has a Y-shaped brown mark. On the male genitalia, the gonocoxite IX is long, with dorsally curved base; the posterior apex is dorsolaterally curved, and equipped with four digitiform processes: two short, apical, and two preapical of which the inner one is longer and thickened. The gonostyli X forms several coils and two internal loops.

##### Etymology.

The name of this new species comes from the Latin –*involvere*– meaning involved or enveloped in allusion to the coiled shape of the male gonostyli X of this species.

##### Description.

***Measurements*.** Male (*n* = 1). Forewing length: 8.6 mm; Hind wing length: 7.7 mm.

**Figure 93.**
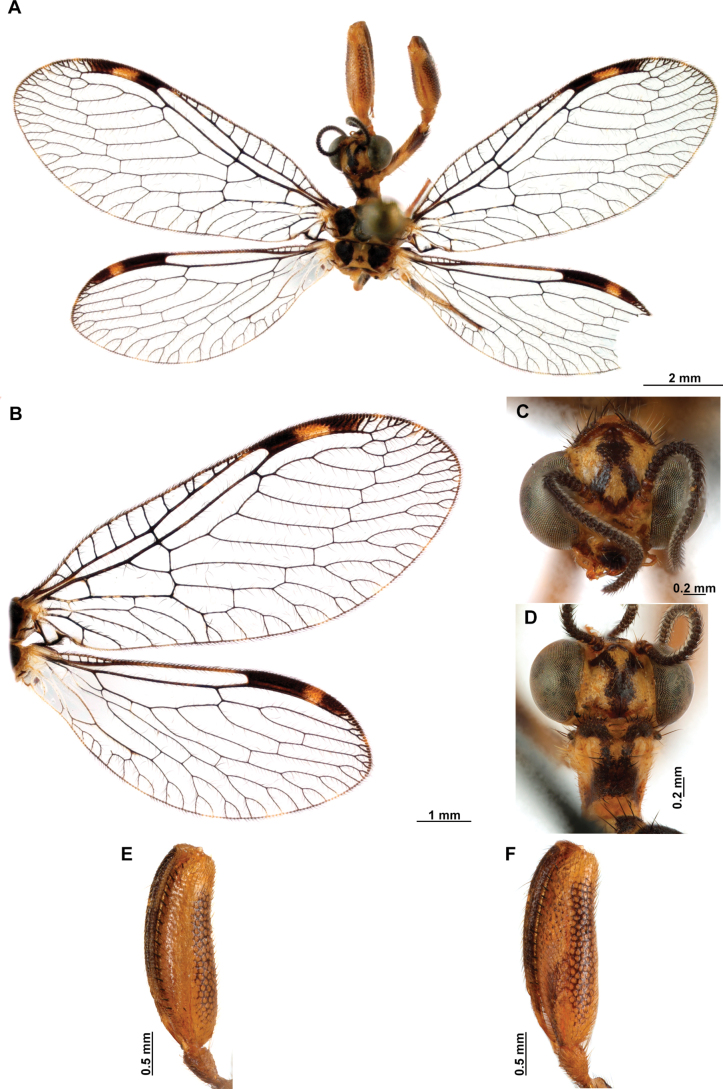
*Trichosceliainvoluta* Ardila-Camacho & Contreras-Ramos, sp. nov. **A** male habitus, dorsal **B** wings **C** head, frontal **D** pronotum, dorsal **E** forefemur, anterior surface **F** same, posterior surface.

***Coloration*** (Fig. 93). ***Head*.** Yellow, vertexal region with brown band extended from occipital ridge to supraantennal region, forked at middle, reaching laterally to toruli; covered with pale brown setae. Antennal scape yellow with brown distal margin; pedicel pale brown; flagellum brown. Interantennal area with M-shaped, brown mark; frons with brown suffusion; clypeus brown at center and anterior margin; labrum brown; mandible amber. Labium and maxilla yellow with pale brown areas; palpimacula yellow. ***Thorax*.** Pronotum yellow, with medial mark that bifurcates anterolaterally and posterolaterally towards corners; episternum yellow with anterior brown marking; postfurcasternum yellow. Mesonotum yellow with brown lateral spots on scutum, anteromedially with two triangular markings, scutellum yellow; metanotum yellow with lateral, circular brown spots on scutum; scutellum yellow with central brown marking. Pre-episternum mostly brown. Mesopleura yellow with brown markings on episternum, epimeron with brown marking near the pleural wing process; metapleura yellow. ***Foreleg*.** Coxa yellow with brown markings dorsally at base and apex; trochanter yellow, brown ventrally. Femur yellow, with an elongated area composed of brown markings forming a honeycomb pattern on anterior and posterior surfaces; area adjacent to closing area on posterior surface with brown spots and subbasal area. Tibia brown, with paler base and apex, clavate setae yellow. Basitarsus pale brown, with darker area proximally on posterior surface, clavate setae yellow; eutarsus yellow. ***Mid- and hind leg*.** Mid-leg coxa yellow with brown basal marking, trochanter and femur yellow, tibia yellow with brown base, tarsus pale brown. Hind leg coxa as in mid-leg, trochanter yellow, femur brown with yellow apex, tibia brown with yellow distal 1/3, tarsus as in mid-leg. ***Wings*.** Forewing hyaline, pterostigma brown with yellow medial area; longitudinal veins mostly dark brown, subcostal veinlets and crossveins dark brown; wing margin alternating brown and yellow. Hind wing hyaline; pterostigma brown with yellow preapical area; venation mostly brown, except C + Sc and proximal area of Cu and anal veins yellow; posterior and distal margin alternating yellow and brown. ***Abdomen*.** Cleared.

***Morphology* (Fig. 93). *Head*.** Diamond-shaped in frontal view, moderately setose, smooth; vertexal region domed vertex above compound eyes, paraocular area concave; coronal suture discrete. Postgena broad view, hypostomal bridge delimited by ridge. Compound eye hemispheric, as wide as ¾ of interocular distance at torulus level. Antenna moniliform, scape 1.5× as long as wide, slightly distally expanded; pedicel as long as wide; flagellum not dorsoventrally compressed, with 35 flagellomeres, those of basal 1.5× as wide as long, the rest almost as wide as long, progressively smaller towards apex; all flagellomeres with medial row of thickened setae. Maxillary palpus with first palpomere as long as wide, second and third 2× as long as wide, fourth ~ 2× as long as wide, fifth as long as third. Postmentum oval with long and thickened setae, palp with first palpomere 1.5× as long as wide, second 2.5× as long as wide, third as long as second, widely expanded, palpimacula ovoid. ***Thorax*.** Pronotum 1.5× as long as wide, with furrow contiguous to lateral and anterior margins; in lateral view anterior margin, medial region and posterior margin slightly elevated, with thickened, pedicellate setae; episternum with long thin setae; postfurcasternum trapezoidal. Mesonotum 1.5× wider than long, with thick, pedicellate setae on medial area. Metanotum ~ 2× as wide as long, glabrous. Pteropleura covered with abundant short and thin setae, some thicker on mesanepisternum. ***Foreleg*.** Coxa slightly shorter than femur, cylindrical, with long and thin setae; trochanter subtrapezoidal, covered with short, thin setae; femur thickened, with abundant short and thin setae; closing surface with double row of tubercle-shaped specializations fully developed, both with a more developed sub-basal process (process/setae ratio 1:1); rows of thickened, pedicellate setae adjacent to each row of processes extending over almost the entire closure surface. Tibia almost as long as femur, curved, setose, with short and thin setae, closing surface with prostrate setae; with a patch of clavate setae apically on anterior surface; basitarsus elongated, ventrally keel and with a row of prostrate setae on proximal ½, anterior surface with patch of clavate setae proximally, lanceolate process, reaching the base of fourth tarsomere, with plug-shaped Stitz organ at apex; second tarsomere ~ 4× as long as wide, third tarsomere as long as wide, fourth tarsomere 2× as long as wide. ***Mid- and hind leg*.** Mid-leg with coxa and trochanter covered with long, thin setae; tibia and femur with interspersed long, short, thin setae; basitarsus 3.5 times as long as wide, second tarsomere slightly longer than wide, third and fourth as long as wide, fifth tarsomere ~ 2× as long as wide, all covered with short, thin setae; the first four tarsomeres with a pair of thickened setae laterally on distal margin of plantar surface. Hind leg longer than mid-leg, with thin setae; tibia 1.5× as long as femur, with short, thin setae; tarsomeres as in mid-leg. ***Wings*.** Forewing oval, trichosors present along wing margin except at base, venation setose; costal space slightly widened medially, humeral vein simple or forked; 9–11 subcostal veinlets present; pterostigma elongated, narrow, gently curved, composed of numerous, mainly incomplete veinlets; subcostal space with single medially located crossvein; Sc vein bent posteriad at proximal margin of pterostigma to merge the RA; *rarp2* curved, with three veins arising from it, three from *rarp1*; M fused basally to R; base of RP located near the separation of M and R, M fork near such separation, opposite to R fork; 1r-m located between RP base and MA base, forming a trapezoidal cell; six gradate crossveins present. Cu deeply forked, CuP basally angled, approaching A1, forked slightly before separation of M and R. A1 simple, reaching the level of separation of M and R; A2 distally forked at level of Cup angle. Hind wing smaller and narrower than forewing; costal space narrow and reduced, with six veinlets; Sc and C fused at 1/5 of wing length; Sc diverging at proximal margin of pterostigma to merge the RA; pterostigma elongated, narrow, curved, composed of numerous incomplete veinlets; radial space with single crossvein, oblique; three veins arising from *rarp1*, one from *rarp2*. M forked beyond R fork; 1 r-m sigmoid, connecting M base and RP base, distally connected again to M stem through short crossvein. Cu deeply forked, CuA long and sinuous, distally forked, first branch candelabrum-shaped; CuP distally forked anteriorly curved, pectinate. Cubitoanal space with two crossveins. A1 arched, A2 short and arched. ***Abdomen*.** Cylindrical, tergites quadrangular; intertergal membranes between segments III–V, covered with microtrichia, and with glabrous lateral areas; sternites rectangular.

**Figure 94.**
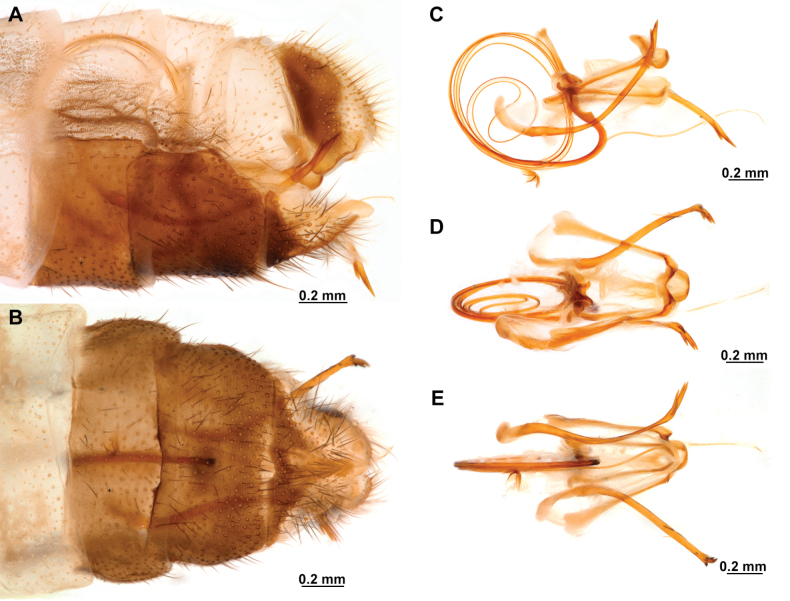
*Trichosceliainvoluta* Ardila-Camacho & Contreras-Ramos, sp. nov. **A** male terminalia, lateral **B** same, ventral **C** male genitalia, lateral **D** same, dorsal **E** same, ventral.

***Male genitalia*** (Fig. 94). Tergite IX medially narrower than laterally, lateral margin with posterior notch. Sternum VIII triangular; sternite IX pentagonal, posteromedially forming an acute angle, glabrous, dorsally canaliculated, the rest of the surface with abundant long and thin setae; in lateral view acuminate, with apex slightly curved, reaching posterior margin of ectoproct. Gonocoxite IX long and thickened, sigmoidal, base spatulate, dorsally curved; apex dorsolaterally curved, with four digitiform processes, two apical, subequal, and short; one outer, preapical, and short; and another inner, longer, and thickened. Ectoproct triangular, setose; ventral surface with convex and sclerotized anterior area, followed posteriorly by curved groove. Gonocoxites X forming a short, thickened, ventrally canaliculate sclerite; posterior apex with two dorsolateral processes, and two lateroventral processes. Gonostyli X with thickened, straight base, set with lateral processes, the rest of the structure long, curved ventrally and coiled anteriorly, forming several coils and two internal loops, before protruding from the abdomen. Gonapophyses X straight, thin, with dorsally curved posterior apex; gonapophyses arranged in V-shaped structure, joined by membrane covering base of gonostyli X. Gonocoxites XI U-shaped, median lobe expanded and elaborated, with two differentiated parts: dorsal part as a trapezoidal lobe; ventral part as a convex area covered with granules, with a U-shaped ventral border; between these parts a narrow less sclerotized is present. Lateral arms of gonocoxites XI straight, anterior apex flattened and expanded.

##### Distribution.

Brazil (Rondônia), Colombia (Meta).

##### Remarks.

This species is known from the Brazilian state of Rondônia and Meta, in Colombia, which suggest a wide distribution in the Amazonia. Based on its body coloration with yellow and dark brown marks, plus the hyaline wings this species is quite similar to other Amazonian species like *T.nassonovi*, *T.basella*, *T.umbrata*, or *T.karijona*. Considering the morphology of the male genitalia, this species appears to be closely related to *T.basella* and *T.umbrata*.

#### ﻿﻿ Trichoscelia iridella

Taxon classificationAnimaliaNeuropteraRhachiberothidae

(Westwood, 1867)

Figs 95 , 96

Mantispa (Trichoscelia) iridella Westwood, 1867: 503. Holotype: male, Brazil (NHMUK), specimen examined.
Trichoscelia iridella (Westwood, 1867). Penny, 1982a: 431.

##### Material examined.

***Lectotype*.** Brazil • ♂; [**Pará**] Tapajos; 53/27; *iridella* Westwood, red type label, Lectotype male, *Trichosceliairidella*Westwood 1867, designated by R.G. Beard 1968; NHMUK 011250017; NHMUK.

##### Diagnosis.

This species is distinguished from its congeners because the wings are completely hyaline. The pterostigma of both wings are brown, with a wide yellowish area on the forewing and a small apical yellowish area on the hind wing. On the male genitalia, the gonocoxite IX is long and arched, with posterior apex laterodorsally recurved, thickened, and equipped with 11 or 12 processes of different sizes, arranged in a cluster. The dorsal part of the medial lobe of the gonocoxites XI is an acuminate, anteriorly curved process.

##### Description.

***Measurements*.** Male (*n* = 1). Forewing length: 9.2 mm; Hind wing length: 6.8 mm.

**Figure 95.**
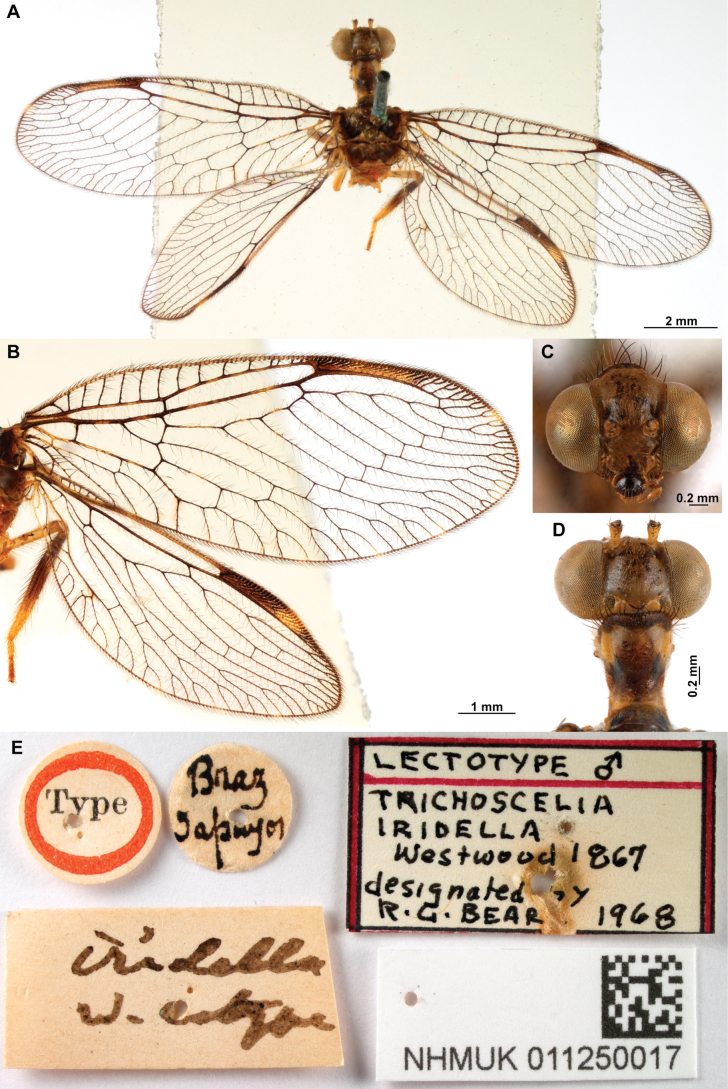
*Trichosceliairidella* (Westwood, 1867) **A** male holotype habitus, dorsal **B** wings **C** head, frontal **D** pronotum, dorsal **E** holotype labels.

***Coloration*** (Fig. 95). ***Head*.** Mostly brown, vertexal region with dark brown markings and yellow setae. Postgena yellow; hypostomal bridge pale brown with dark margins. Antennal scape mainly yellow, distally with dark brown suffusion, with brown setae. Clypeus pale brown, with dark medial marking; labrum dark brown; mandible dark brown at base, paler at apex. Maxilla yellow with brown palpus, the last palpomere paler. Postmentum yellow, prementum pale brown, ligula dark brown; labial palpus with first three palpomeres dark brown, the last pale brown, palpimacula pale brown. ***Thorax*.** Pronotum yellow with anterior margin and large medial area brown, with interspersed dark brown and pale brown setae; episternum bicolor, with yellow and brown areas, pale brown setae present; postfurcasternum yellow. Meso-pre-episternum brown; mesoscutum mainly brown, scutellum yellow with dark brown central area, setation mostly dark brown; metanotum mainly brown, with yellow anteromedial area. Mesopleuron with sclerites brown with yellow margins, metapleuron mainly yellow. ***Foreleg*.** Coxa with irregular pale - and dark brown areas, setation pale brown. ***Mid- and hind leg*.** Coxae brown, trochanter yellow, femora and tibiae with proximal 2/3 dark brown, apex yellow; tarsi pale brown, ventrally with dark brown setae. ***Wings*.** Forewing hyaline, proximal region of subcostal field amber; venation alternating brown and yellow, branches of RP and M, mostly brown; pterostigma brown with wide yellow medial area. Setation predominantly brown, not always corresponding to the cuticle color. Hind wing hyaline, venation predominantly brown, distal region of C + Sc, base of Cu, A2 and base of posterior margin yellow; pterostigma brown with yellow preapical area; membrane surrounding base of first branch of CuA amber.

***Morphology*** (Fig. 95). ***Head*.** Semi-triangular in frontal view, moderately setose, smooth; vertex slightly domed above compound eyes, with rows of short, reclined setae; paraocular area concave; coronal suture discernible. Postgena broad postgena, hypostomal bridge not entirely fused to it. Compound eye hemispheric, as wide as 3/4 of the interocular distance at torulus level. Antenna moniliform, scape ~ 2× as long as wide, slightly distally expanded. Frons trapezoidal, narrow; clypeus oval; labrum narrow, rounded. Maxillary palpus with the first two short, the third and fifth 3× as long as wide, the fourth 2× as long as wide. Maxillary palpus with third palpomere expanded and rounded with widely ovoid palpimacula. ***Thorax*.** Pronotum 1.5× as long as wide, with a groove contiguous to the lateral and distal margins; anterior margin, medial area, and posterior margin slightly raised with long and thickened setae arising flush the pronotal surface; episternum mainly with long, thin setae; postfurcasternum trapezoidal. Mesonotum slightly wider than long, mesoscutum and scutellum with abundant long, thickened setae on medial area. Metanotum ~ 2× as wide as long. Pteropleura covered with abundant long, thin setae. ***Foreleg*.** Coxa cylindrical and elongated, with abundant fine, long setae. ***Mid- and hind leg*.** Mid-leg covered with abundant fine, long setae; tibia slightly flattened; basitarsus ~ 4× as long as wide, third and fourth as long as wide, second slightly longer than wide; fifth tarsomere ~ 2× as long as wide, the first four tarsomeres with paired, thickened, lateral setae on the distal margin of plantar surface. Hind tibia slightly thickened, longer than femur; tarsomeres similar to those of mid-leg. ***Wings*.** Forewing oval, venation setose, trichosors present along wing margin except at base of posterior margin; costal space slightly expanded proximally, humeral vein simple or forked; 14 subcostal veinlets present; pterostigma approximately narrow, gently curved, with numerous simple veinlets; subcostal space with single, medially located crossvein; Sc vein abruptly bent posteriorly at proximal margin of pterostigma to merge the RA; *rarp2* gently curved, with three or four RP branches, four from *rarp1*; M fused basally to R; RP base located near the separation of M and R, M fork near such separation; 1r-m located between RP base and M fork forming a small trapezoidal cell; six or seven gradate crossveins present. Cu deeply forked, CuA forked opposite the RP stem; CuP proximally angulated, touching A1, distally forked at level of divergence of M and R. 1A simple, ending at posterior margin, almost at level of separation of M and R; 2A distally forked, slightly beyond the level of CuP angle. Hind wing oval, smaller and narrower than forewing; costal space narrow and reduced, with four veinlets; C and Sc fused at proximal ¼ of wing length, Sc vein abruptly, posteriorly curved at the proximal margin of pterostigma to merge the RA; pterostigma elongated and narrow; radial space widened with single crossvein, which is oblique; three veins arising from *rarp1*, two from *rarp2*. 1r-m sigmoid, connecting M base and RP base, distally connected again to M stem through a short crossvein. M forked slightly beyond the R fork. Cu deeply forked, CuA long, sinuous, distally forked; first branch candelabrum-shaped; CuP distally anteriorly curved, pectinate. A1 arched, A2 simple, short. ***Abdomen*.** Cylindrical, without processes or pores, tergites of abdominal segments III and IV with wide posteromedian concavity.

**Figure 96.**
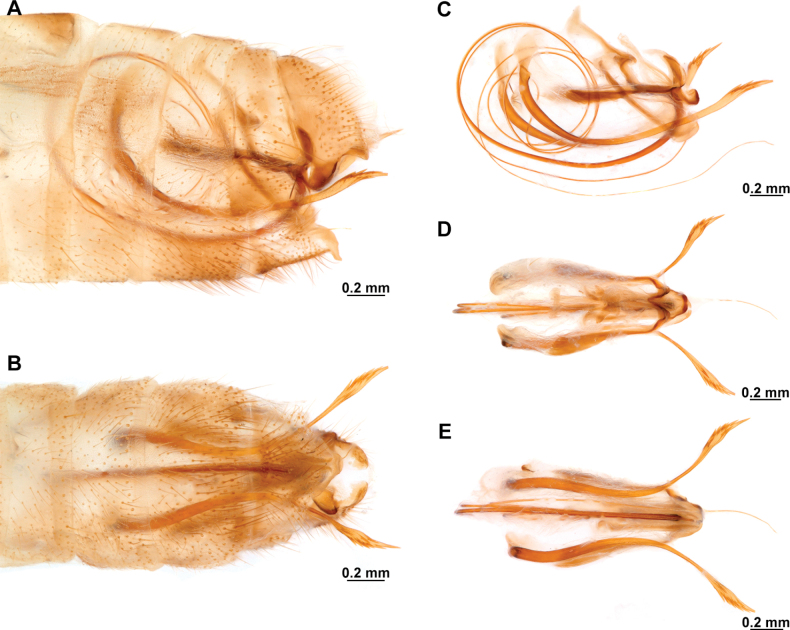
*Trichosceliairidella* (Westwood, 1867) **A** male terminalia, lateral **B** same, ventral **C** male genitalia, lateral **D** same, dorsal **E** same, ventral.

***Male genitalia*** (Fig. 96). Tergite IX narrower dorsally than laterally, notched posterolaterally. Sternite VIII rectangular, posteriorly broadly concave. Sternite IX pentagonal, setose, apex blunt and ventrally bent, dorsally canaliculate; in lateral view not surpassing posterior margin of ectoproct. Gonocoxite IX long and arched, base flattened; apex laterodorsally recurved, thickened, with 11 or 12 processes of different sizes, organized in a cluster. Ectoproct approximately ovoid, moderately setose, ventrally with anterior, triangular bulging area covered with microtrichia and a well-sclerotized posterior concave area. Gonocoxites X forming a short, ventrally canaliculate sclerite, with anterior apex expanded and dorsally bent; posterior apex bilobed, connected to gonostyli X and two short, preapical, lateroventral processes connected to gonapophyses X with a membrane. Gonostyli X base unthickened, bent, forming an obtuse angle, with two lateral processes; the rest of the structure abruptly anteroventrally curved, long and anteriorly coiled, forming an internal loop before protruding from abdomen. Gonapophyses X long, straight and thin, united, posterior apex dorsally bent, whose surrounding membrane is set with minute spinules; gonapophyses joined by a membrane covering the base of gonostyli X. Gonocoxites XI U-shaped, medial lobe enlarged and elaborated, consisting of two differentiated parts: dorsal part as an acuminate, anteriorly curved process; ventral part as curved arch, which is ventromedially incised, and posterior surface is covered with minute spinules; area between these parts less sclerotized. Lateral arms of gonocoxites short, straight, flattened, anterior apex spatulate.

##### Distribution.

Brazil (Pará).

##### Remarks.

This species was redescribed by Penny (1982a) and Penny and da Costa (1983) and was recorded from Brazil (Amazonas), Ecuador (Napo), Panama (Cabal Zone), and Trinidad (Penny 1982a; Ardila-Camacho et al. 2018). However, most of these records are actually from *T.nassonovi* or from other species. Indeed, the redescription presented by Penny (1982a) is likely a composite of several species. Since herein, the synonymy of *T.basella* was invalidated, and many of the aforementioned records are from other species, the only valid record of this species is that of the lectotype of *T.iridella* which is from the Tapajós river from Pará, Brazil.

The body coloration and the hyaline wings of this species and its similitude with *T.basella* lead to propose both species as the same (Penny 1982a). However, the genitalia of the true *T.iridella* is quite distinctive and shares several attributes with other completely different species. For instance, the male gonocoxites IX are quite similar to those of *T.vespiformis*, while the dorsal part of the median lobe of the gonocoxites XI is similar to that of *T.anae*.

#### ﻿﻿ Trichoscelia karijona

Taxon classificationAnimaliaNeuropteraRhachiberothidae

Ardila-Camacho, 2015

Figs 97 , 98

Trichoscelia karijona Ardila-Camacho, 2015: 427. Holotype: male, Colombia, Caquetá (IAvH), specimen examined.

##### Material examined.

***Holotype*.** Colombia • ♂; **Caquetá**, PNN Chiribiquete, Puerto Abeja, bosque coluvial (Calle 6-550 m); 310 m; 24 Jan–01 Feb. 2000; C. Arenas leg.; Malaise; IAVH-E; IAvH. ***Paratypes***. Colombia – **Caquetá** • 1 ♂; PNN Chiribiquete, Puerto Abeja, bosque coluvial, (Calle 6-550); 310 m; 02–12 Feb. 2000; C. Arenas leg.; Malaise; IAvH-E 115979; IAvH. – **Meta** • 1 ♂; PNN Tinigua, vereda Bajo Raudal; 2°16'N, 73°48'W; 460 m; 29 Jun–20 Jul. 2002; C. Sanchez leg.; Malaise, M.2332; IAvH. – **Putumayo** • 1 ♂; PNN La Paya, La Nueva Paya; 0°02'S, 75°12'W; 210 m; 31 Jan–03 Feb. 2003; C. Sarmiento leg.; Malaise, M.3423; IAvH. – **Vichada** • 1 ♀; PNN El Tuparro, bosque de sabana; 5°21'N, 67°51'W; 100 m; 17–26 Dec. 2000; W. Villalva leg.; Malaise, M1739; IAvH.

##### Other material.

Colombia • 1 ♂; **Putumayo**, Mocoa, Vda. Pueblo Viejo, Fca. Villa Loca; 01°11'28.1"N, 76°38'48.3"W; 690 m; 28 Feb. 2016; E. Delgado leg.; aerial net; UNAB.

French Guiana – **Camopi** • 1 ♂; Mont Saint-Marcel, Mont St.-Marcel, Inselberg culminant á; 2°23'29"N, 53°0'50"W; 635 m; 25 Sep. 2016; Thounrend, Slam leg.; CSCA. – **Roura** • 1 ♀; Montagne des Chevaux, Carriere du Galion, Crete avec forét sur quartzite érodée; 4°44'31.54"N, 52°25'53.02"W; 15 Sep. 2018; charaxes trap; CSCA.

Guyana • 1 ♂; [**Potario-Siparuni**], Potaro; Jan. 1921; G.E. Bodrif leg.; “Pres. by Imp. Bur. Ent. Brit. Mus. 1927-71”; NHMUK 013802781; *Anisopterasequella* Westwood, det. Esben-Petersen; NHMUK.

##### Diagnosis.

This species is recognized based on its broadly oval, hyaline forewing. The pterostigma of both wings is dark brown, sometimes with small, pale, medial area on the forewing. On the male genitalia, the gonocoxite IX is short, thin, and noticeably arched with posterior apex laterally projected, equipped with a long preapical process, and sometimes with a shorter one near the apex. On the female genitalia, the gonapophyses VIII form an arcuate, convex plate, posteromedially equipped with a bifid process; the lateral part of the gonapophyses VIII is trapezoidal, and forms a ventral flattened lobe, which is covered with microtrichia.

##### Description.

***Measurements*.** Male (*n* = 5). Forewing length: 6.8–7.8 mm; Hind wing length: 5.0–6.0 mm.

**Figure 97.**
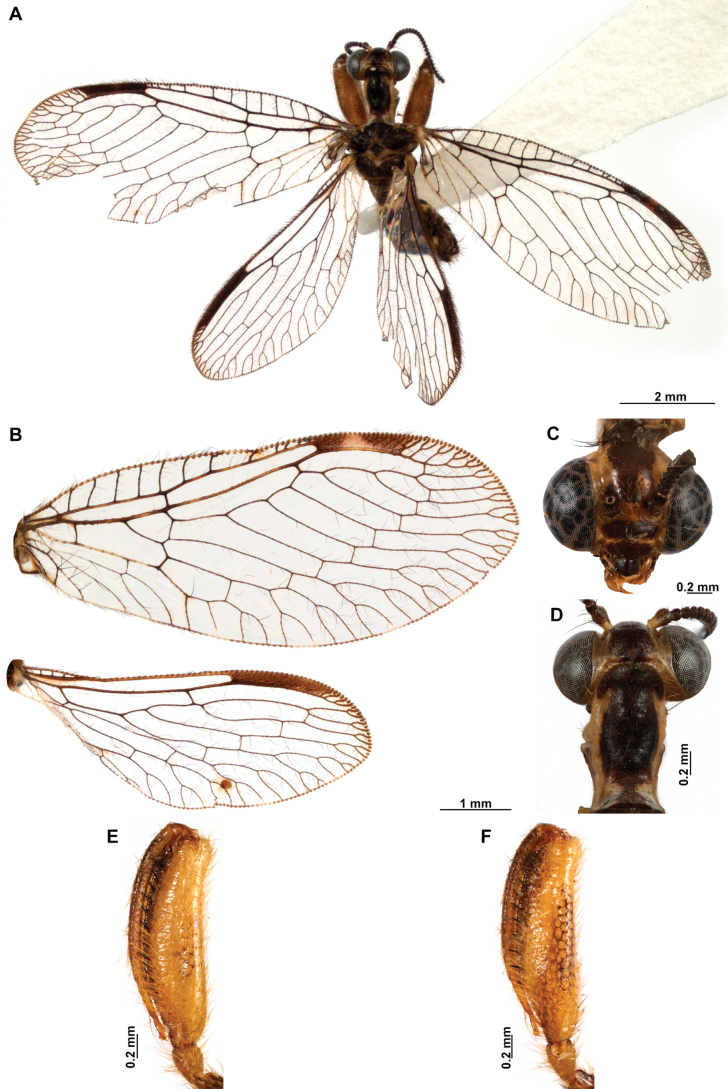
*Trichosceliakarijona* Ardila-Camacho, 2015 **A** male habitus, dorsal **B** wings **C** head, frontal **D** pronotum, dorsal **E** forefemur, anterior surface **F** same, posterior surface.

***Coloration*** (Fig. 97). ***Head*.** Mainly yellow, vertexal area with longitudinal, brown band extending from occiput to supraantennal area where it is forked; pale brown setae present. Antennal scape yellow at base, brown on remaining surface, pedicel brown; flagellum brown. Frons brown with yellow margins; clypeus brown with yellow posterolateral corners; labrum brown; mandible yellow with brown suffusions at base, pale amber at apex. Maxilla yellow with a brown spot on the stipes, palpus dark brown; labium yellow, palpus dark brown, yellow palpimacula, brown setae. ***Thorax*.** Pronotum yellow with longitudinal, medial, broad brown band that extends laterally on anterior margin and posterior area, with concolorous setae; episternum bicolor, with brown and yellow; postfurcasternum yellow. Pre-episternum brown. Mesonotum predominantly brown, with yellow on area surrounding sutures, scutellum brown at center and yellow at margins; metanotum dark brown with a white central area, anterior and posterior edges yellow. Pteropleura yellow with brown areas, metakatepisternum completely yellow. ***Foreleg*.** Coxa yellow with brown areas; trochanter yellow with brown suffusions ventrally. Femur yellow, posterior surface with wide brown area at center that extends proximally towards the ventral surface; this area is composed of brown polygonal marks with yellow center, forming a honeycomb pattern; anterior surface with brown suffusions at base; setae mostly yellow, brown near closing surface. Tibia yellow with brown suffusions, interspersed yellow and brown setae, clavate setae yellow. Basitarsus with yellow proximal ½, pale amber lanceolate process; second to fourth tarsomere yellow. ***Mid- and hind leg*.** Mid-leg with brown coxa, trochanter yellow, femur yellow, tibia with brown proximal ½, and yellow on the rest; tarsus yellow, progressively changing to pale brown. Hind leg with coxa mainly brown with a yellow area at center, trochanter and femur yellow, tibia brown on proximal ½, yellow on the rest; tarsus as in mid-leg. ***Wings*.** Forewing hyaline; pterostigma brownish with pale yellow medial area, sometimes barely perceptible; longitudinal veins predominantly brown, with large proximal area of Cu and A1 yellow; subcostal veinlets and crossveins brown; wing margin alternating brown and yellow. Hind wing hyaline, sometimes with amber on stem of first branch of CuA; pterostigma either completely brown or with small, pale yellow, preapical area; venation mostly brown, except proximal region of Cu and anal veins yellow; wing margin alternating yellow and brown. ***Abdomen*.** Tergites of abdominal segments I and II yellow with a brown medial marking, tergites III–VIII brown with yellow lateral areas. Sternites yellow, with brown lateral markings, progressively larger on each segment. Pleural membrane predominantly yellow on first three abdominal segments, with extensive brown areas on remaining segments.

***Morphology* (Fig. 97)**. ***Head*.** Diamond-shaped in front view, smooth, vertexal area domed above compound eyes; paraocular area concave; coronal suture discrete. Postgena broad, hypostomal bridge not completely fused with it. Compound eye, as wide as ¾ interocular distance at torulus level. Antenna moniliform, scape 2× as long as wide, slightly distally expanded; pedicel as long as wide; flagellum slightly dorsoventrally compressed, with 36 flagellomeres, those of basal ½ 1.5× as wide as long, the rest almost as wide as long, progressively smaller towards the apex; all flagellomeres with a medial row of thickened setae. Submentum oval with long setae, labial palpus with first palpomere 1.5× as long as wide, the second 3× as long as wide, third as long as the second, widely expanded, palpimacula broadly ovoid. Maxillary palpus with first two palpomeres as long as wide, third palpomere 3× as long as wide, the fourth ~ 2× as long as wide, fifth palpomere slightly shorter than third. ***Thorax*.** Pronotum 1.5× as long as wide, with a furrow contiguous to lateral and anterior margins; in lateral view anterior margin, medial region and posterior margin slightly raised, with thickened, pedicellate setae; episternum with long, fine setae; postfurcasternum trapezoidal. Mesonotum 1.5× wider than long, with thick, pedicellate setae on medial area. Metanotum ~ 2× as long as wide, mostly glabrous. Pteropleura covered with abundant short, thin setae. ***Foreleg*.** Coxa slightly shorter than the femur, cylindrical, with abundant short, thin setae; trochanter conical, with protuberance on anterior surface; femur thickened, with abundant short, thin setae; closing surface with double row of tubercle-shaped specializations fully developed, both with a more developed sub-basal process (anteroventral row process/seta ratio 2:1; posteroventral row 1:1); rows of thickened setae with globular-base extending over almost the entire closure surface. Tibia almost as long as the femur, curved, setose; closing surface with prostrate, flattened setae; with a patch of clavate apically on inner surface; basitarsus ventrally keeled and with a row of prostrate on proximal ½, anterior surface with clavate setae proximally, lanceolate process elongated, reaching the base of fourth tarsomere, with a plug-shaped organ of Stitz at apex; second tarsomere 4× as long as wide, third tarsomere as long as wide, fourth tarsomere 2× as long as wide. ***Mid- and hind leg*.** Mid-leg with coxa and trochanter covered with long, thin setae; tibia slightly longer than femur; basitarsus 4× as long as wide, second tarsomere slightly longer than wide, third and fourth as long as wide, fifth tarsomere ~ 2× as long as wide; the first four tarsomeres with a pair of thickened setae laterally on distal margin of plantar surface. Hind leg longer than mid-leg, tibia slightly flattened, 1.5× as long as femur; tarsomeres similar to those of mid-leg. ***Wings*.** Forewing oval, trichosors present along wing margin except at base of posterior margin; venation setose; costal space slightly widened medially, humeral vein simple or forked, 11 or 12 subcostal veinlets; pterostigma elongated, narrow, composed of numerous veinlets, mainly incomplete; subcostal space with a single medially located crossvein; Sc vein bent posteriorly at proximal margin of pterostigma to merge the RA; *rarp2* curved, with two or three veins arising from it, three from *rarp1*; M vein fused basally to R; base of RP located near separation of M and R, M fork near such separation, opposite to R fork; 1r-m located between RP base and MA base, forming a small trapezoidal cell; five or six gradate crossveins present. Cu vein deeply forked; CuP basally angled, approaching A1, distally forked opposite to separation of M and R. A1 simple, reaching the level of separation of M and R; A2 distally forked at the level of CuP angle. Hind wing smaller and narrower than forewing; costal space narrow and reduced, with three or four veinlets; Sc and C fused at proximal 1/5 of wing length, Sc curving posteriorly at proximal margin of pterostigma to merge the RA; pterostigma elongated, narrow, curved, composed of numerous incomplete veinlets; radial space with single crossvein, oblique; two or three veins arising from *rarp1*, one from *rarp2*. M forked slightly beyond R fork; 1 r-m sigmoid, connecting M base and RP base, distally connected again to M stem through short crossvein. Cu deeply forked, CuA long, sinuous, distally forked, first branch candelabrum-shaped; CuP distally forked, distally anteriorly curved, pectinate; A1 arched, A2 short and arched. ***Abdomen*.** Cylindrical, tergites quadrangular; intertergal membranes between segments III–V in both sexes broad, covered with microtrichia, and with two glabrous lateral areas; sternites rectangular, except segments II and III trapezoidal.

**Figure 98.**
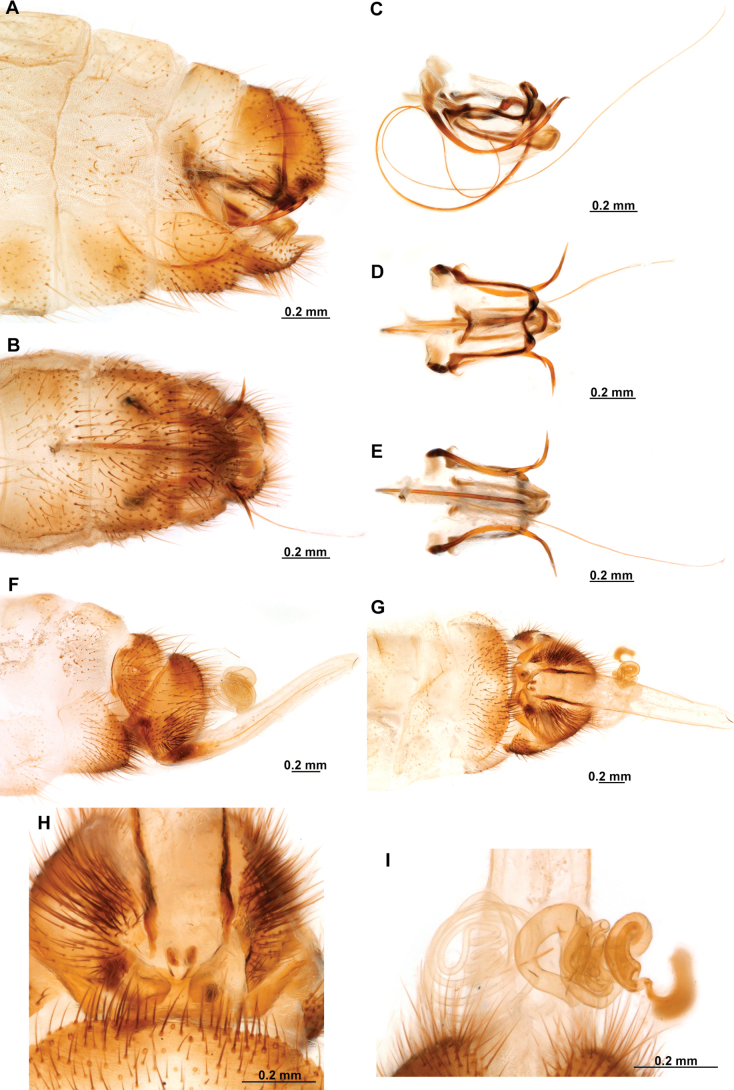
*Trichosceliakarijona* Ardila-Camacho, 2015 **A** male terminalia, lateral **B** same, ventral **C** male genitalia, lateral **D** same, dorsal **E** same, ventral **F** female terminalia, lateral **G** same, ventral **H** gonapophyses VIII ventral **I** spermatheca.

***Male genitalia*** (Fig. 98A–E). Tergite IX dorsally narrower than laterally, lateral margin with anterior corner hooked and curved towards central body axis, posterior corner with wide notch. Sternum VIII rectangular; sternite IX pentagonal, convex, posteromedially forming an acute angle, glabrous, dorsally canaliculate, the rest of the surface with abundant long and thin setae; lateral view spoon-shaped, with apex reaching posterior margin of ectoproct. Gonocoxite IX short, thin, and noticeably arched, base spatulate; apex laterally projected, with long preapical process, and sometimes with a shorter one near the apex. Ectoproct ovoid, setose; ventral surface with a sclerotized, semi-triangular anterior lobe covered with minute spinules, followed posteriorly by a broad concave semicircular plate. Gonocoxites X forming a short, thin, ventrally canaliculate sclerite; anterior apex slightly expanded; posterior apex bilobed, connected to gonostyli X, two additional processes are preapically located and connected to gonapophyses X with a membrane. Gonostyli X with thin and straight base, with two lateral processes, the rest of the structure, long, curved ventrally and anteriorly coiled, forming two loops before protruding from abdomen. Gonapophyses X straight and thin, posterior apex dorsally bent; gonapophyses arranged in a V-shape structure, joined by membrane covering gonostyli X base. Gonocoxites XI U-shaped, with medial lobe expanded and elaborated, with two differentiated parts: dorsal part as a rounded, concave, anterodorsally projected lobe; ventral part consisting of a convex area covered with microspinules; ventral edge U-shaped; between these parts a narrow, less-sclerotized region is present. Lateral arms sinuous, anterior apex spatulate, laterally bent.

***Female genitalia*** (Fig. 98F–I). Sternite VII rectangular, with posterior margin broadly rounded, setose. Tergite VIII remarkably wider dorsally than laterally, enclosing the spiracle of eighth abdominal segment, lateral margin triangular. Membrane between tergite VIII and tergite IX + ectoproct sclerotized, forming a concave, triangular plate. Gonocoxites VIII forming a trapezoidal plate, with two lateral concave areas; gonapophyses VIII as an arcuate, convex plate, posteromedially with bifid process; laterally with two trapezoidal plates, which form ventral flattened lobe, covered with microtrichia; such plate is connected to tergite IX + ectoproct. Tergite IX + ectoproct ovoid. Gonocoxite IX long and narrow, as long as the last four abdominal segments together. Bursa copulatrix membranous, long, thin, with the proximal part sclerotized, widened and concave; spermatheca complex and entangled; proximal section long, thin, forming four coils, terminating in an innermost spiral; medial section thicker than proximal section, entangled, forming multiple convolutions. Distal section progressively widened towards apex, distally with a trumpet-shaped invagination. Fertilization canal duct short, thin, sigmoid; fertilization canal short, thickened, J-shaped, covered with microfilaments.

##### Distribution.

Colombia (Caquetá, Meta, Putumayo, Vichada), French Guiana (Camopi, Roura) Guyana (Potaro-Siparuni).

##### Remarks.

This species was previously known only from its original description and was known from the Colombian Amazonian (Caquetá, Meta, Putumayo, and Vichada). Here the first records from French Guiana and Guyana are presented.

This species is markedly similar to *T.sequella* in the general body coloration pattern and hyaline wings, particularly the dark brown pterostigma, generally lacking a faint pale medial area. The separation of the females requires a careful examination of the coloration and of the genitalia. Conversely the male genitalia of this species are quite similar to that of *T.nassonovi*, and probably both species are closely related.

#### ﻿﻿ Trichoscelia larizae

Taxon classificationAnimaliaNeuropteraRhachiberothidae

Ardila-Camacho & Contreras-Ramos sp. nov.

https://zoobank.org/C249D257-0A39-4EB8-AA37-150045AC7BFE

Figs 99 , 100

##### Type locality.

Panama, **Colón**: Porto Bello, 09 Mar. 1911, August Buck, N. Banks leg.

##### Material examined.

***Holotype*** male, pinned, genitalia stored in a separate vial. Original label: “Panama [**Colón**] Porto Bello, 09 Mar. 1911, August Buck, N. Banks” FSCA. ***Paratype***. Panama • 1 ♀; [**Colón**] Porto Bello; 21 Apr. 2012; A. Busk leg.; USNMENT01541909; USNM.

##### Diagnosis.

This species is closely related to *T.basella*; however, the costal field of the forewing in *T.larizae* is narrower. On the male genitalia, the gonocoxites IX are straighter and shorter than in *T.basella*, in which the base is noticeably expanded and dorsally curved inwards. In addition, the apex of the male gonocoxite IX of both species has a similar design, although in *T.larizae* it is laterally curved and equipped with four digitiform processes of which two are apical and medially located and two are preapical laterally located.

##### Etymology.

This species is named after Sara Lariza Rivera Gasperín, Mexican entomologist, and expert on Melolonthinae beetles, who supported this research in many ways.

##### Description.

***Measurements*.** Male (*n* = 1). Forewing length: 7.69 mm; Hind wing length: 5.7 mm.

**Figure 99.**
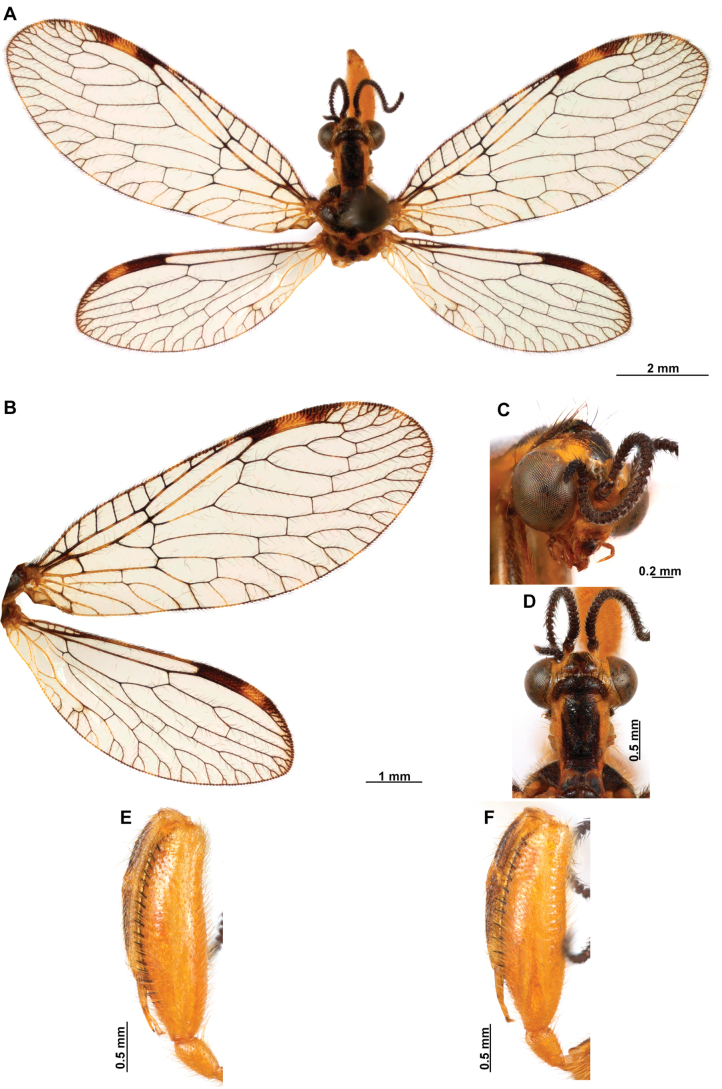
*Trichoscelialarizae* Ardila-Camacho & Contreras-Ramos, sp. nov. **A** male habitus, dorsal **B** wings **C** head, frontolateral **D** pronotum, dorsal **E** forefemur, anterior surface **F** same, posterior surface.

***Coloration* (Fig. 99)**. ***Head*.** Mainly yellow, vertexal region with longitudinal brown band extending from occipital ridge to supraantennal area; with pale brown setae. Antennal scape yellow at base, brown towards apex; pedicel and flagellum dark brown. Frons with brown transverse stripe; clypeus yellow with brown suffusions; labrum brown, yellow on margins; mandible yellow; maxillary palpus pale brown; labium yellow, palpus pale brown, palpimacula yellow. ***Thorax*.** Pronotum yellow with brown longitudinal, medial band and anterior margin. Episternum bicolor, brown on anterior ½, yellow posteriorly; postfurcasternum yellow. Meso- and metanotum with sclerites brown with yellow margins; mesopleuron yellow with brown marks, metapleuron mostly yellow. ***Foreleg*.** Coxa yellow with anterior surface brown; trochanter and femur yellow; tibia yellow with brown marks. Basitarsus pale brown; tarsomeres 2–4 yellow with pale brown suffusions. ***Mid- and hind leg*.** Coxae brown, remaining podomeres yellow. ***Wings*.** Forewing hyaline with base of subcostal field dark amber; pterostigma brown, yellow at middle; venation alternating dark brown and yellow, except Anal veins mostly yellow; posterior and distal margins alternating dark brown and yellow. Hind wing hyaline, base of subcostal space dark amber; pterostigma brown with yellow preapical area; longitudinal veins mostly brown except anal veins and large areas of Cu yellow; posterior and distal margins alternating yellow and brown. ***Abdomen*.** Cleared.

***Morphology* (Fig. 99)**. ***Head*.** Semi-triangular in frontal view, sparsely setose, smooth, vertexal region slightly domed above compound eyes; paraocular area concave; coronal suture not discernible. Compound eye hemispheric, as wide as ¾ interocular distance at torulus level. Antenna filiform, scape ~ 2× as long as wide; flagellum with 38–40 flagellomeres, those of basal ½ 2× as wide as long, progressively narrower towards apex; all flagellomeres with medial ring of thickened setae. First two palpomeres of maxillary palpus as long as wide, third palpomere 3× as long as wide; fourth palpomere two times as long as wide; fifth palpomere slightly longer than third. Labial palpus with first palpomere 1.5× as long as wide; second 4× as long as wide; third palpomere expanded, slightly longer than second, palpimacula broadly ovoid. ***Thorax*.** Pronotum longer than wide, with groove contiguous to lateral and anterior margins; in lateral view anterior margin, medial region and posterior margin raised, with thickened setae arising flush the pronotal surface. Mesonotum 1.5× as wide as long, with scattered, fine setae; metanotum two times as wide as long, glabrous. Pteropleura covered with abundant, fine setae. ***Foreleg*.** Coxa slightly shorter than femur, cylindrical, with abundant fine, long setae; trochanter subtrapezoidal; femur robust, with abundant fine, short setae; closing surface with double row of integumentary specializations fully developed; laterally to each row of processes a row of thickened setae with globular base present. Tibia almost as long as femur, curved, setose; closing surface keeled, with prostrate setae; anterior surface with patch of clavate setae at apex. Basitarsus elongated, equipped with clavate setae, proximally on anterior surface, ventrally keeled, with row of prostrate setae; lanceolate process reaching the middle of fourth tarsomere, equipped with apical plug-shaped Stitz organ. ***Mid- and hind leg*.** Mid-leg covered with abundant fine, long setae, shorter on tarsus. Hind leg longer than midleg, densely covered with long, thin setae, except on tarsus shorter; plantar surface of fifth four tarsomeres with two stout setae, laterally on each side. ***Wings*.** Forewing oval, trichosors present along wing margin except at base, venation setose; costal space narrow, humeral vein sometimes forked, 11–13 subcostal veinlets; pterostigma elongated and narrow, composed of numerous, mostly incomplete veinlets; subcostal space with single, medially located crossvein; Sc vein bent posteriad at proximal margin of pterostigma to merge the RA; *rarp2* gently curved with two veins arising from it, three from *rarp1*; four gradate crossveins present. Media fused basally to R; RP base located near separation of M and R, M fork near such separation, 1r-m located between RP base and MA base, forming a small trapezoidal cell; M fork opposite to R fork. Cu deeply forked, CuA long; CuP proximally angled, touching A1, distally forked, ending at posterior margin at level of separation of M and R. A1 simple, ending at posterior margin at level of separation of M and R; A2 forked. Hind wing smaller and narrower than forewing; costal space narrow and reduced, with four veinlets; C and Sc fused at 1/5 of wing length; Sc vein curved posteriad at proximal margin of pterostigma to merge the RA. Pterostigma elongated, narrow, curved, composed of numerous, incomplete veinlets; radial space with single crossvein, oblique; two veins arising from *rarp1*, one from *rarp2*. Media forked beyond R fork. Cubitus deeply forked; CuA, sinuous, long, distally forked, first branch candelabrum-shaped; CuP distally anteriorly bent, pectinate. Cubitoanal space with two crossveins. A1 arched, A2 short and arched. ***Abdomen*.** Cylindrical, tergites subquadrate; sternites rectangular.

**Figure 100.**
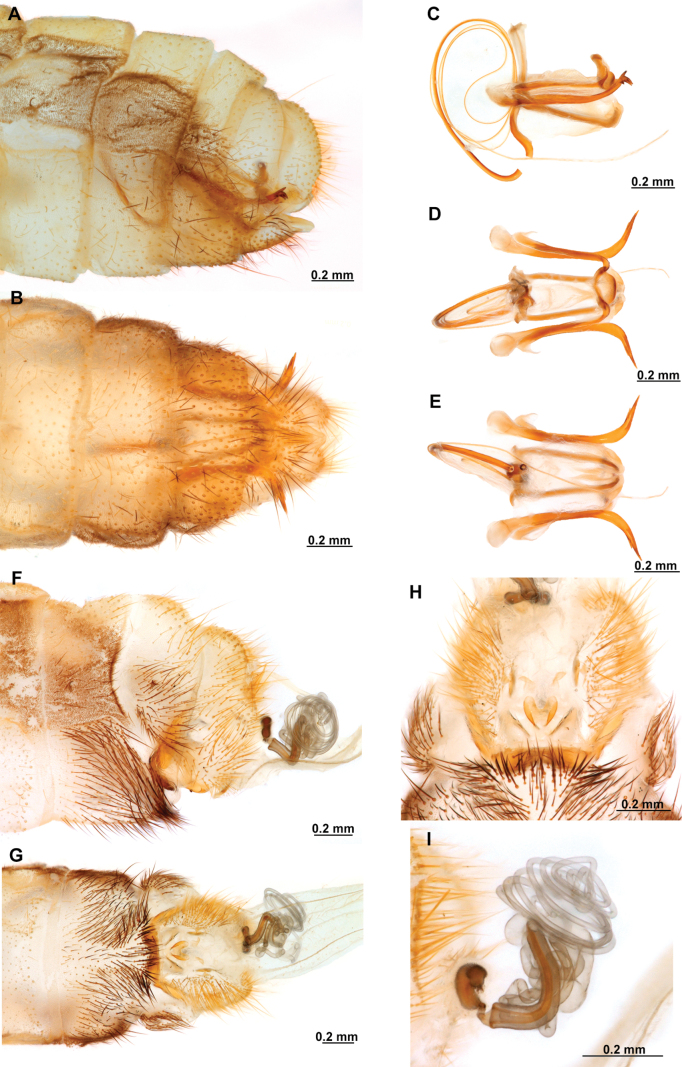
*Trichoscelialarizae* Ardila-Camacho & Contreras-Ramos, sp. nov. **A** male terminalia, lateral **B** same, ventral **C** male genitalia, lateral **D** same, dorsal **E** same, ventral **F** female terminalia, lateral **G** same, ventral **H** gonapophyses VIII ventral **I** spermatheca.

***Male genitalia*** (Fig. 100A–E). Tergite IX dorsally narrower than laterally, lateral margin posteriorly notched. Sternite VIII trapezoidal. Sternite IX pentagonal, setose, posteromedially forming an acute angle; in lateral view acute reaching posterior margin of ectoproct. Gonocoxites IX narrow and elongated; base spatulate; apex laterally curved, with four processes, of which two are apical and medially located, and two are preapical and laterally located. Ectoproct ovoid; ventral surface with anterior area convex, covered with microtrichia; this surface is posteriorly continued by ventromedial sclerotized sulcus. Gonocoxites X forming a short, straight, ventrally canaliculate sclerite; anterior apex slightly expanded; posterior apex bilobed, and with two preapical, lateroventral processes. Gonostyli X with straight and thin base, with two lateral processes, the rest of the structure ventrally curved, anteriorly coiled, forming two loops before protruding from abdomen. Gonapophyses X straight, narrow, posterior apex dorsally bent, with surrounding membrane set with microtrichia; gonapophyses subparallel, joined by membrane covering base of gonostyli X. Gonocoxites XI thin, U-shaped, medial lobe elaborated, with two differentiated parts: dorsal part a quadrangular lobe; ventral part a convex area covered with microspinules, ventral edge incised U-shaped; between these parts a trapezoidal, less sclerotized area present. Lateral arms of gonocoxites XI straight with anterior apex incurved.

***Female genitalia*** (Fig. 100F–I). Sternite VII subrectangular, broader medially than laterally. Tergite VIII slightly narrower medially than laterally, enclosing the spiracle of eighth abdominal segment, lateral margin blade-shaped. Membrane between tergite VIII and tergite IX + ectoproct sclerotized, forming a concave triangular plate. Gonocoxites VIII forming a trapezoidal plate, with two lateral concavities; gonapophyses VIII with keel-shaped medial part, posteromedially with bilobed process; lateral part a trapezoidal, convex, plate. Tergite IX + ectoproct ovoid. Gonocoxite IX long and narrow, as long as the last three abdominal segments together; gonapophyses IX as two small sclerites located on the inner surface on the base of gonocoxites IX. Bursa copulatrix funnel-shaped, membranous, long, thin, proximal portion sclerotized and concave; spermatheca complex and entangled; proximal section long, thin, forming six coils, ending in an innermost spiral; medial section slightly thicker than proximal section, entangled, forming multiple convolutions. Distal section thin, forming several convolutions, progressively wider towards apex, distally with a trumpet-shaped invagination. Fertilization canal duct short, thin, sigmoid; fertilization canal short, thickened, J-shaped, covered with microfilaments.

##### Distribution.

Panama (Colón).

##### Remarks.

Based on the male genitalia configuration, this species appears to be closely related to several Amazonian species such as *T.basella*, *T.fenella*, *T.involuta*, and *T.umbrata* and the Mesoamerican *T.flavomaculata* and *T.tobari*, being probably an intermediary species between the aforementioned groups. This species is known only from Panama, yet its distribution range could reach other countries of Central America and the Darien Gap in Choco, Colombia.

#### ﻿﻿ Trichoscelia latifascia

Taxon classificationAnimaliaNeuropteraRhachiberothidae

McLachlan, 1867

Figs 101 , 102

Trichoscelia latifascia McLachlan, 1867: 255. Holotype: male, Brazil, Amazonas (NHMUK), specimen examined.
Anisoptera jocosa Gerstaecker, 1888: 117. Holotype: female, Peru, Huánuco (NHRS), photographs examined.
Trichoscelia jocosa (Gerstaecker, 1888). Penny (1977): 37.
Symphrasis thaumasta Navás, 1915: 197. Holotype: male, Brazil, Amazonas (NMBS).
Trichoscelia thaumasta (Navás, 1915). Penny (1977): 37.

##### Material examined.

***Holotype*** of *Trichoscelialatifascia*.

Brazil • ♂; [**Amazonas**] Ega; Bates leg.; *Trichoscelialatifascia* Westwood, *Trichoscelialatifascia*McLachlan 1867, R.B. Beard; McLachlan Coll. B.M. 1938-674, type red label”; NHMUK 013802772; NHMUK.

***Holotype*** of *Anisopterajocosa*.

Peru • ♀; [**Huánuco**] Pachitea; *Anisopterajocosa* Gerst. Det. Esben-Petersen; 199; NHRS-GULI 000070207; NHRS.

##### Other material.

Ecuador • 1 ♂; **Napo**, Limoncocha; 00°24'S, 76°36'W; 280 m; 04 Jul. 1975; #5419, Carl W. Rettenmeyer, Ruth Chadab, V-168, reared from nest of *Polybiarejecta* (F.) det. L. Stange, 2004; FSCA. • 1 ♂; same data as for preceding; 28 Jun. 1975; #5419-C; FSCA. • 1 ♀; same data as for preceding; 29 Jun. 1975; #5419F; FSCA. • 1 ♀; same data as for preceding; 29 Jun. 1975; #5419; FSCA. • 1 ♂; same data as for preceding; 01 Jul. 1975; #5419-S; FSCA. • 2 ♀; 25 Km e. Puerto Napo, Cabañas Aliñahui rainforest; 450 Km; Sep. 1997; B & B Valentine leg.; FSCA. • 1 ♀; vic. Puerto Misahuallí; 1650–1900 ft.; 6–19 Sep. 1998; J.E. Eger leg.; FSCA. • 1 ♂ 1 ♀; 25 Km E Puerto Napo, Aliñahui; 1°00'S, 77°22'W; 450 m a.s.l.; Feb. 1994; E.S. Ross leg.; CAS. • 1 ♂; Coca; 249 m; 9–19 Feb. 1986; McKamey leg.; CAS.

##### Diagnosis.

This species is easily separated from all its congeners because its coloration pattern of orange and dark brown or black. The head and the anterior ½ of the pronotum are black. The posterior surface of the forefemur is orange with the basal region dark brown. The forewing has an anterior and a posterior amber spot at the level of the RP fork, plus a wide, longitudinal, preapical band. The hind wing has the costal space, the base of the subcostal space, and the wing apex amber; an amber area is present between the CuA and the posterior wing margin. On the male genitalia, the gonocoxite IX is long, arched, with posterior apex laterodorsally recurved, and equipped with four or five apical processes, of which two or three are medial and two are lateral; an additional long and curved process is dorsally located on distal ¼ of the gonocoxite length. On the female genitalia, the gonapophyses VIII form a semi-triangular, convex, medial part, set with a posteromedial bifid process; the lateral part is a quadrangular plate, with convex ventral surface.

##### Description.

***Measurements*.** Male (*n* = 3). Forewing length: 9.8–10.0 mm; Hind wing length: 7.3–7.4 mm. Female (*n* = 4). Forewing length: 9.9–10.0 mm; Hind wing length: 7.3–7.6 mm.

**Figure 101.**
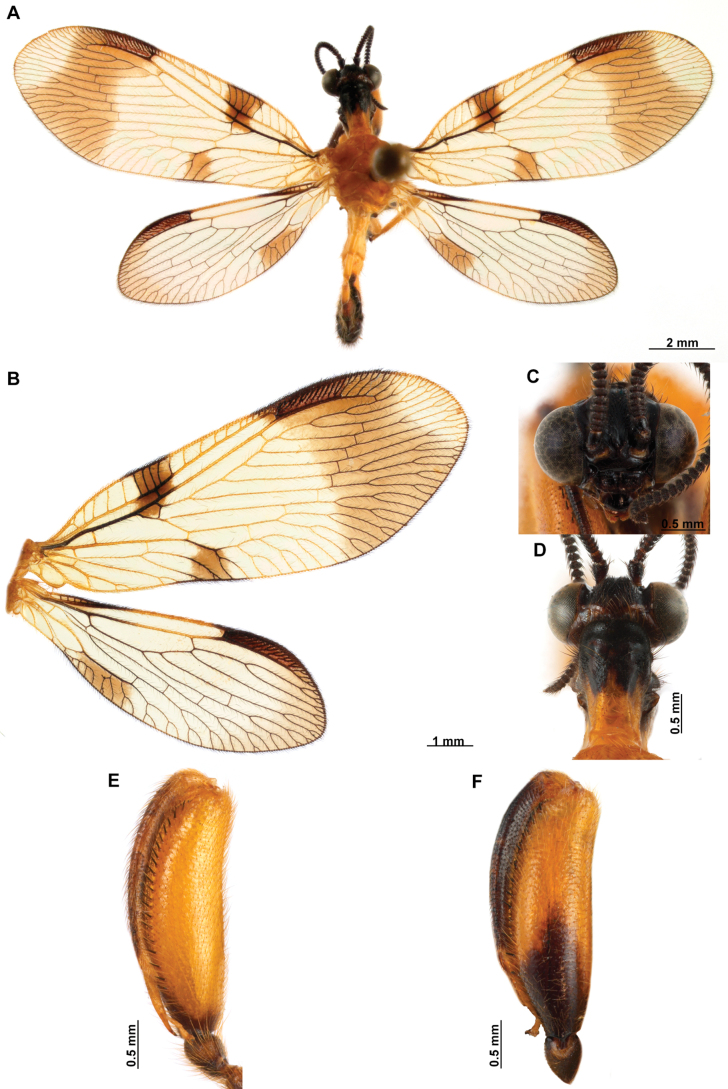
*Trichoscelialatifascia* McLachlan, 1867 **A** male habitus, dorsal **B** wings **C** head, frontal **D** pronotum, dorsal **E** forefemur, anterior surface **F** same, posterior surface.

***Coloration* (Fig. 101)**. ***Head*.** Mostly blackish brown; vertex with dark brown setae. Antenna uniformly dark brown. Frons, clypeus and labrum dark brown; mandible dark amber at base, paler at apex. ***Thorax*.** Pronotum with dark brown anterior ½ and lateral margin, posterior ½ orange; episternum dark brown; postfurcasternum orange. Meso- and metanotum uniformly orange. Pre-episternum mostly brown. Pteropleura mostly orange, with brown mesokatepisternum. ***Foreleg*.** Coxa orange coxa, except proximal 1/3 and apex brown, setae mainly orange. Trochanter brown, paler on anterior surface, interspersed brown and orange setae present. Femur orange, posterior surface with dark brown mark extending from base to preapical area; anterior surface with brown base. Tibia mostly brown, with orange base and apex; basitarsus mainly orange, with amber apex; remaining tarsomeres orange. ***Mid- and hind leg*.** Med leg with coxa and trochanter brown, interspersed orange and brown setae; femur orange with proximal 1/3 brown; tibia and tarsus orange, plantar surface with brown setae. Hind leg similar to mid-leg; femur orange with brown base; tibia dark brown, except base orange; tarsus orange mainly with brown setae. ***Wings*.** Forewing mostly hyaline with two broad amber spots, one surrounding the 1sc-r and extending towards costal field and *rm1*; the other is surrounding first branch of CuA and apex of CuP; at wing apex a broad transverse band extending from pterostigma to posterior margin is present; pterostigma brown. Venation mainly orange, brown inside amber areas; R+M, proximal part of R, proximal ½ of Sc, and Cu base brown. Hind wing mostly hyaline, with slightly suffused apex, costal field and base of subcostal field amber; a large amber area lies between Cu and posterior margin at M fork level. Pterostigma brown. Venation mostly brown, C + Sc, medial area of ​​RA, base of M, Cu, anal veins orange; posterior margin orange at base and on middle. ***Abdomen*.** Abdominal segments I–V orange, segment VI mainly orange with brown areas, remaining segments dark brown in both sexes.

***Morphology* (Fig. 101)**. ***Head*.** Diamond-shaped in frontal view, setose, smooth; vertexal region domed over compound eyes, laterally with short, reclined rows of setae; paraocular area concave; coronal suture not discernible. Compound eye hemispheric, as wide as 3/4 of the interocular distance at torulus level. Postgena broad, hypostomal bridge not entirely continuous to it. Antenna moniliform, scape ~ 2× as long as wide, slightly distally expanded; pedicel slightly longer than wide; flagellum dorsoventrally compressed, with 43–45 flagellomeres, those of flagellar base discoidal, progressively narrower towards the apex; all flagellomeres with medial ring of thickened setae. Frons narrow, pentagonal; clypeus narrow, trapezoidal; labrum rounded. Maxillary palpus with the first two palpomeres short, the third and fifth 3× as long as wide, the fourth 2× as long as wide. Labial palpus with third palpomere expanded and rounded, palpimacula broadly ovoid. ***Thorax*.** Pronotum slightly longer than wide, with groove contiguous to the lateral and distal margins; posterior margin, medial area and anterior margin slightly elevated with long, thickened setae arising flush the pronotal surface; episternum mainly with long, thin setae; postfurcasternum trapezoidal. Mesonotum slightly wider than long, scutum and scutellum with abundant, long, thickened setae present on medial area. Metanotum ~ 2× as wide as long, with two lateral cusps on scutum. Pteropleura covered with abundant short, thin setae. ***Foreleg*.** Coxa as long as femur, cylindrical, with abundant fine and short setae; trochanter subconical, densely setose, dorsally with long, thick, pedicellate setae near distal margin; femur robust, sub-cylindrical, covered with fine and short setae. Closing surface with both rows of specializations fully developed, composed of tubercle-shaped specializations, alternating thickened and thin processes. Posteroventral row proximally with a more developed sub-basal process (process/seta ratio 3:1); anteroventral row with a more developed sub-basal process (process/seta ratio 2:1); thickened setae with globular base present as two rows adjacent to the process rows, extending almost the entire length of the closing surface. Tibia almost as long as the femur, curved, setose, closing surface keeled with a row of flattened prostrate setae, anterior surface with a patch of clavate setae on apex. Basitarsus elongated, ventrally keeled and with a row of prostrate setae on proximal 2/3, anterior surface with clavate setae on proximal ½, lanceolate process short, reaching the base of fourth tarsomere, with plug-shaped Stitz organ at tip; second tarsomere 3× as long as wide, third tarsomere as long as wide, last tarsomere 2× as long as wide. ***Mid- and hind leg*.** Mid-leg covered with abundant fine and short setae; basitarsus ~ 4× as long as wide, second slightly longer than wide, third and fourth tarsomere as long as wide, fifth tarsomere ~ 2× as long as wide; first four tarsomeres with a pair of thickened setae laterally on distal margin of plantar surface. Hind leg densely covered with short thin setae, shorter on tarsus; tibia slightly thickened and flattened, longer than femur; tarsomeres similar to those of the mid-leg. ***Wings*.** Forewing oval, venation densely setose, trichosors present along wing margin except at base; costal space proximally slightly expanded, humeral vein forked, 14–16 subcostal veinlets present, some of which are forked; pterostigma approximately rectangular, straight, with numerous, mainly incomplete veinlets; subcostal space with a single medially located crossvein; Sc vein abruptly bent posteriad at proximal margin of pterostigma to merge the RA; *rarp2* gently curved with 2–4 veins arising from it, 3–6 from *rarp1*; M fused basally to R; base of RP located near separation of M and R, M fork near such separation; 1r-m located between RP base and M fork forming a small trapezoidal cell; 5–7 gradate crossveins present. Cubitus deeply forked, CuA forked slightly beyond the base of RP; CuP proximally angled, almost touching A1, forked distally slightly before divergence of M and R. 1A distally forked, ending at posterior margin, almost at level of separation of M and R; 2A distally forked at CuP angle level. Hind wing oval, notably smaller and narrower than forewing; costal space narrow and reduced, with five or six subcostal veinlets; C and Sc fused at proximal ¼ of wing length, Sc vein abruptly bent posteriad at proximal margin of pterostigma to merge the RA; pterostigma elongated and narrow; radial space with crossvein, oblique; three veins arising from *rarp1*, 1–3 from *rarp2*; 1–3 gradate crossveins. 1r-m sigmoid, connecting M base and RP base, distally connected again to M stem through a short crossvein. M vein forked beyond M fork. Cu deeply forked, CuA long, sinuous, distally branched; CuP distally forked, terminal portion anteriorly curved, pectinate. A1 arched, A2 short and simple. ***Abdomen*.** Cylindrical, slightly distally expanded, without processes or pores; tergites quadrangular, those of segments III and IV with posteromedial concavity; sternites quadrangular.

**Figure 102.**
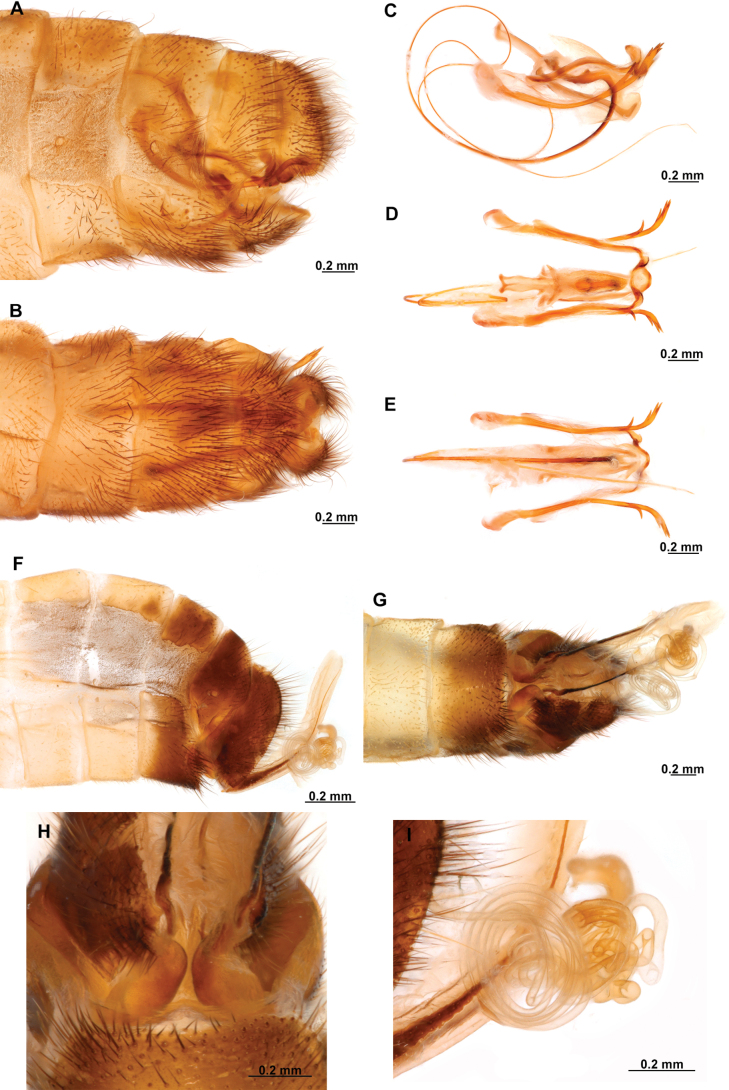
*Trichoscelialatifascia* McLachlan, 1867 **A** male terminalia, lateral **B** same, ventral **C** male genitalia, lateral **D** same, dorsal **E** same, ventral **F** female terminalia, lateral **G** same, ventral **H** gonapophyses VIII ventral **I** spermatheca.

***Male genitalia*** (Fig. 102A–E). Tergite IX slightly narrower dorsally than laterally, lateral margin triangular, approaching sternite IX. Sternite VIII rectangular with concave posterior margin. Sternite IX subpentagonal, posteromedial portion glabrous, blunt, dorsally canaliculate; in lateral view slightly ventrally curved and not reaching posterior margin of ectoproct. Gonocoxite IX long, arched, base spatulate; apex laterodorsally recurved, with four or five apical processes, of which two or three are medial and two lateral; an additional long and curved process is dorsally located on distal ¼ of gonocoxite length. Ectoproct approximately ovoid, setose, ventrally with anterior bulging area, covered with microtrichia; posteriorly towards the inner margin a more sclerotized, semi-triangular plate is present. Gonocoxites X forming a short, straight, ventrally canaliculate sclerite; anterior apex slightly expanded, posterior apex bilobed, and with two short ventrolateral processes connected to gonapophyses X with a membrane. Base of the gonostyli X thickened, curved, with two lateral processes; the rest of the structure abruptly anteroventrally curved and anteriorly coiled, forming two loops before protruding from abdomen. Gonapophyses X straight, narrow, posterior apex dorsally bent, with surrounding membrane set with minute spinules; gonapophyses arranged in a V-shaped structure, joined by a membrane covering the gonostyli X base. Gonocoxites XI approximately U-shaped, median lobe enlarged and elaborated, composed of two differentiated parts: dorsal part as a concave, arcuate lobe; ventral part as a short convex region whose surface is covered with minute spinules; ventral margin incised; area between these parts less sclerotized. Lateral arms of gonocoxites XI short and sinuous.

***Female genitalia*** (Fig. 102F–I). Sternite VII subtrapezoidal. Tergite VIII dorsally slightly narrower than dorsally, enclosing the spiracle of the eighth abdominal segment. Membrane between tergite VIII and tergite IX + ectoproct sclerotized, forming a concave triangular plate. Gonocoxites VIII forming a trapezoidal plate, with two lateral concavities; gonapophyses VIII with medial part convex, semi-triangular, with posteromedial bifid process; lateral part as a quadrangular plate, with convex ventral surface, connected to tergite IX + ectoproct. Tergite IX + ectoproct trapezoidal, with rounded posterior margin. Gonocoxite IX long, sinuous and narrow, as long as the last four abdominal segments together. Bursa copulatrix membranous, long and thin, progressively narrower. Spermatheca complex and entangled; proximal section long and thin forming six coils, ending in a spiral; medial section thicker than proximal section, entangled, and forming multiple convolutions. Thin distal section, with multiple convolutions, distally with a trumpet-shaped invagination. Fertilization canal duct short, thin and sigmoid; fertilization canal short, thickened, pod-shaped, covered with microfilaments.

##### Distribution.

Brazil (Amazonas), Ecuador (Napo), Peru (Huánuco).

##### Remarks.

The type of *Anisopterajocosa* was collected in the province of Huánuco, Peru, and the types of *Symphrasisthaumasta* and *Trichoscelialatifascia* were collected in Tefé, Brazil, in the state of Amazonas, although this information is not specified in the original descriptions nor in the labels of the types. The synonymies of this species were corroborated by means the examination of the holotype *Trichoscelialatifascia* and high-resolution photographs of *Anisopterajocosa*. The synonymy of *Symphrasisthaumasta* is warranted as in the original description by Navás (1915), the wing pattern perfectly matches with the holotype of *T.latifascia*. Herein the first record from Ecuador (Napo) is presented.

This species presents a remarkable body coloration pattern very similar to that of *Anchietafasciatellus* which consist in extensive orange areas with black or dark brown stripes or spots. This is a mimetic pattern very common in different insect groups and whose models could be probably species of Braconidae which bear repugnant glands on the abdomen.

The male genital sclerites of this species are similar to those of *T.varia* and probably both species are closely related.

#### ﻿﻿ Trichoscelia nassonovi

Taxon classificationAnimaliaNeuropteraRhachiberothidae

(Navás, 1912)

Figs 103 , 104

Symphrasis nassonovi Navás, 1912: 537. Lectotype: male, Peru, Lima (ZIL). Designated by Krivokhatsky, (1995): 11, specimen examined.
Trichoscelia nassonovi (Navás, 1912). Krivokhatsky (1995): 11.
Symphrasis trifasciata Stitz, 1913: 44. Holotype: male, Bolivia (ZMB), specimen examined.
Trichoscelia trifasciata (Stitz, 1913). Penny (1977): 37.

##### Material examined.

Lectotype of *Trichoscelianassonovi*.

Peru • ♂; Callanga; 2–3000 m; Staudinger leg.; *Symphrasisnassonovi* Nav. Long. Navás det., type red label; Lectotypus *Symphrasisnassonovi* Nav. Design. V. Krivokhatsky, 1994, red label; ZIN.

***Paralectotype*** of *Trichoscelianassonovi*.

Peru • ♂; Callanga; 2–3000 m; Staudinger leg.; *Symphrasisnassonovi* Nav. Long. Navás det., type red label; type red label; Lectotypus *Symphrasisnassonovi* Nav. Design. V. Krivokhatsky, 1994, red label; ZIN.

***Holotype*** of *Trichosceliatrifasciata*.

Bolivia • ♂; Steinbach; *Symphrasistrifasciata* Stitz; Holotype male of *Trichosceliatrifasciata* (Stitz, 1913) R.G. Beard; Holotype male, *Trichosceliatrifasciata* (Stitz, 1913) prep. By R.G. Beard 1968; Type red label; Holotypus Nr., Zool. Mus. Berlin; ZMB.

##### Other material.

Bolivia – **Cercado** • 1 ♀; Tarija, Bermejo; 03 Mar. 1969; R. Golbach leg.; FSCA. – **La Paz** • 1 ♂; 8 Km S. Chulumani, Apa Apa; 16°22'S, 67°30.4'W; 1950–2100 m; 24 Mar. 2001; ex: malaise trap, “Barcode of life DNA voucher specimen” “SimpleID CCDB32125-B04” BOLD Proc.ID: SICOB111-18, USNMENT01454272; USNM. • 1 ♂; [La Paz] Benavides; Jul.; H. Smith leg.; MCZ. – **Santa Cruz** • 1 ♀; Estación Experimental general Saavedra; Sep. 1973; L. Stange leg.; *Trichosceliavaria* (Walker) det. L. Stange, 1979; FSCA. • 1 ♂; [Santa Cruz] E. Buena Vista; J. Steinbach leg.; FSCA. • 1 ♀; Province Sara; 450 m; J. Steinbach; Carn. Mus. Acc. 4545, MCZ-ENT 00681681; MCZ.

Brazil – **Amazonas** • 1 ♀; Reserva Ducke, 26 Km NE Manaus; 14 Mar. 1978; J. Arias & N. Penny leg.; Malaise trap; *Trichosceliairidella* det. L. Stange, 1983; FSCA. • 1 ♂ 1 ♀; Amazonas AM 010, Km 26 Reserva Ducke; 28 Feb. 1978; J. Arias leg.; Malaise trap; FSCA. • 1 ♂ 1 ♀; Amazonas, Reserva Ducke, 26 Km NE Manaus; 04 Apr. 1978; J. Arias & N. Penny leg.; Malaise trap; FSCA. • 1 ♀; Amazonas, vic. Manaus, Reserva Ducke; 24 Jul. 1981; G.B. Firchild leg.; Flight trap; FSCA. • 1 ♂; Amazonas, Reserva Ducke, 26 Km NE Manaus; 14 Aug. 1978; J. Arias & N. Penny leg.; Malaise trap; *Trichosceliairidella* det. L. Stange, 1983; FSCA. • 1 ♂; Amazonas, AM 010, Km 26 Reserva Ducke; 21 Feb. 1978; Jorge Arias leg.; Malaise trap; FSCA. • 1 ♂; Amazonas, AM 010, Km 26, Reserva Ducke; 10 Jan. 1978; C.D.C. light trap 15-2; FSCA. • 1 ♀; Amazonas, Reserva Ducke, 26 Km NE Manaus; 21 Mar. 1978; J. Arias & N. Penny leg.; Malaise trap; FSCA. • 1 ♂; Amazonas, Reserva Ducke, 26 Km NE. Manaus; 07 Mar. 1978; J. Arias & N. Penny leg.; Malaise trap; FSCA. • 1 ♂ 1 ♀; Amazonas, AM 010, Km 26 Reserva Ducke; 07 Feb. 1978; J. Arias leg.; Malaise trap; FSCA. • 1 ♂; Amazonas, 18.1 km E. Campinas field sta. Km 60 N. Manaus; 02°30'S, 060°15'W; 22 Feb. 1979; Montgomery, Erwin, Schimel, Krischik, Date, Bacon leg.; “terra firme forest, canopy fogged with Pyrethrum, sample 22”; USNMENT01541918; USNM. – **Ceará** • 2 ♀; Crato; 900 m; May. 1969; M. Alvarenga; FSCA. • 1 ♀; same data as for preceding; Malaise trap; FSCA. – **Rondônia** • 1 ♂; 62 Km SW Ariquemes, nr Fzda. Rancho Grande; 23–25 Mar. 1996; U. Schmitz, BLT; FSCA.

Ecuador • 1 ♂; **Napo**, Limoncocha; 00°24'S, 76°36'W; 15 Aug. 1972; C.W. Rettenmeyer leg.; #4928-A, “emerged 29–31 Aug. 1972”; USNMENT0151913; *Trichosceliairidella* det. N.D. Penny; USNM. • 1 ♂; same data as for preceding; 15 Aug. 1972; R. Chadab leg.; #366, USNMENT01541914; USNM.

French Guiana • 1 ♂; MTK sun; 20 Aug. 2015; CSCA. • 1 ♂; MCN PVB; 08 Sep. 2018; CSCA. – **Camopi** • 1 ♂; Mont Saint-Marcel, Mont St.-Marcel, Inselberg culminant án; 635 m; 264577 N, 276133 E; 10 Mar. 2017; automatic light trap (pink); CSCA. – **Matoury** • 1 ♀; Mont Matoury, Mont Grand Matoury, Colline entourée, de savanes et zones degrades; 4°51'31.00"N, 52°22'36.00"W; 30 Aug. 2014, forest edge, flight interception trap; CSCA. – **Roura** • 1 ♀; Montagne des Chevaux, Carriere du Calion, Crete avec foret sur quartzite érodée; 4°44'31.54"N, 52°25'53.02"W; 01 Dec. 2018; charaxes trap; CSCA. • 1 ♀; same data as for preceding; 03 Nov. 2018; automatic light trap (pink); CSCA. • 1 ♀; same data as for preceding; 02 Feb. 2016; CSCA. • 1 ♀; same data as for preceding; 11 Mar. 2017; automatic light trap (pink); CSCA. • 1 ♂; same data as for preceding; 03 Jan. 2015; flight interception trap; CSCA. • 1 ♀; same data as for preceding; 24 Sep. 2016; Malaise; CSCA. • 1 ♂; same data as for preceding; 19 Mar. 2016; automatic light trap (blue); CSCA. • 1 ♀; same data as for preceding; 30 Jan. 2016; automatic light trap (pink); CSCA.

PERU – **Huánuco** • 1 ♂; Cueva de las Pavas, near Tingo María; 11–12 Jul. 1974; L. Stange & C. Porter leg.; FSCA. – [**Ica**?] • 1 ?; Callanga; McLachlan Coll. B.M. 1938-674; Abdomen missing; NHMUK 013802780, NHMUK. – **Madre de Dios** • 1 ♂; Río Tambopata Res., 30 Km (air) SW Puerto Maldonado; 290 m; 20–31 Oct. 1982; R. Wilkerson leg.; FSCA. • 1 ♀; Manu, Pakitza; 12°07'S,70°58'W; 250 m; 9–23 Sep. 1988; O. Flint & N. Adams leg.; USNMENT01541919; USNM. • 1 ♀; same data as for preceding; “Barcode of Life DNA voucher specimen” “Simple IDCCDB-32125-B03” “BOLD Proc ID SICOB110-1B”, USNMENT01454271, USNM. • 1 ♀; CICRA, Trail 2, W; 267 m; 12°33'39.744"S, 70°6'23.22"W; 22–23 Nov. 2013; J. Caballero leg.; Malaise trap.; USNMENT01541927; USNM. • 1 ♀; Rio Tambopata Res, 30 air Km SW pto. Maldonado; 290 m; 16–20 Nov. 1979; J.B. Heppner leg.; subtropical moist forest; USNMENT01541911; USNM.

Trinidad And Tobago – [**Tunapuna-Piarco**] • 1 ♂ 1 ♀; Simla Field Sta., Arima Valley; 2–13 May. 1977; P. Feinsinger leg.; Malaise trap, Trop. Rain Forest; FSCA. – [**Couva-Tabaquite-Talparo**?] • 1 ♂; Monserrat; 30 Jun. 1905; Aug. Busck leg.; *Symphrasissequella* West. Det. N. Banks; FSCA. • 1 ♂; River Estate; 16 Oct. 1918; H. Morrison leg.; A-768; USNMENT01541915; USNM. • 1 ♂; Monserrat; 30 Jun. 2005; A. Busck leg.; USNMENT01541910; *Trichosceliairidella* det. N.D. Penny, 1981; USNM. • 1 ?; Monserrat; 30 Jun. 2005; A. Busck leg.; USNMENT01541916; USNM.

##### Diagnosis.

This species is distinguished from other South American species because the pterostigma of both wings is brown with a small, yellow area. The forewing membrane has amber infuscations on the area adjacent to crossveins, and apical forks of longitudinal veins. These marks may be from faint to well-marked. On the male genitalia, the gonocoxite IX is thin, short, and strongly curved medially, with base inwardly curved; the apex is curved lateroventrally with two processes, of which the shorter, preapical one lies dorsally; a third shorter process may be present. On the female genitalia, the gonapophyses VIII medial part is quadrangular, with a posteromedial bifid process; the lateral part is an oval plate, with convex ventral surface, covered with microspinules.

##### Description.

***Measurements*.** Male (*n* = 7). Forewing length: 6.9–11.2 mm; Hind wing length: 5.4–8.5 mm. Female (*n* = 3). Forewing length: 6.4–7.4 mm; Hind wing length: 5.5–5.9 mm.

**Figure 103.**
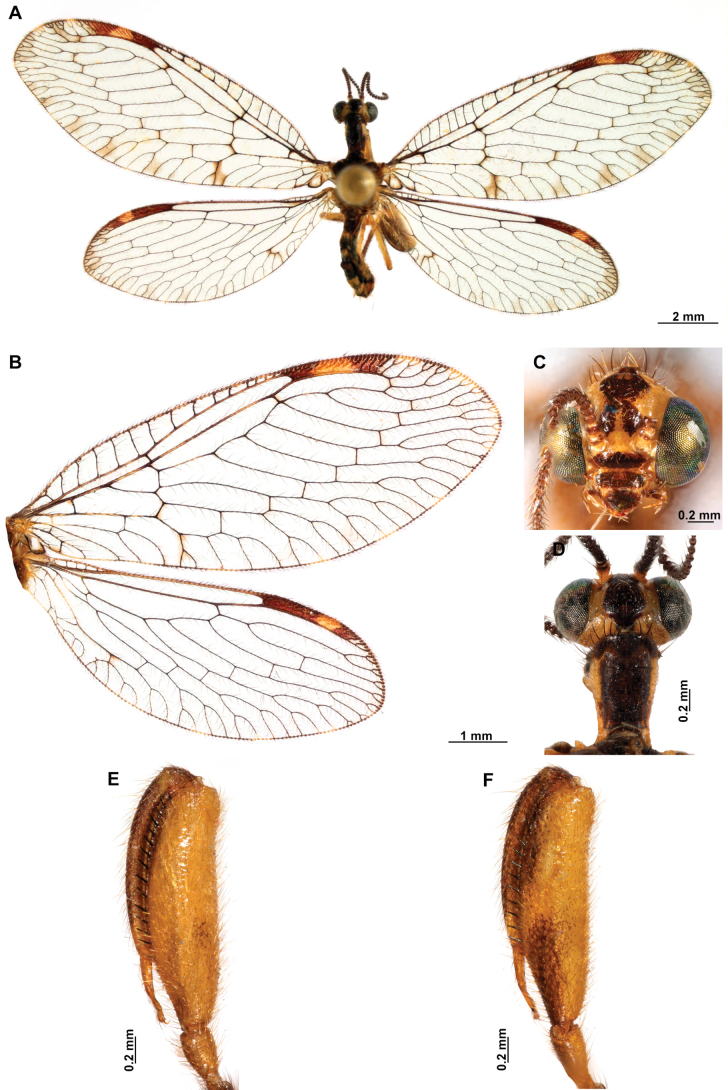
*Trichoscelianassonovi* (Navás, 1912) **A** male habitus, dorsal **B** wings **C** head, frontal **D** pronotum, dorsal **E** forefemur, anterior surface **F** same, posterior surface.

***Coloration* (Fig. 103)**. ***Head*.** Mainly yellow; region of vertex with longitudinal brown band extending from occipital ridge to supraantennal area; with pale brown setae. Postgena sometimes yellowish brown. Antennal scape yellow on basal ½, distal ½ with blackish brown suffusions, dorsally strongly marked, pedicel and flagellum brown. Frons, clypeus and labrum brown with yellow margins. Mandible pale brown; maxillary palpus brown. Labium yellow, except palpus brown, palpimacula pale brown. ***Thorax*.** Pronotum yellow with brown longitudinal, medial band and anterior margin, with concolorous setae; episternum brown, postfurcasternum yellow. Mesonotum brown, except scutellum yellow laterally, setae dark brown; metanotum brown with two small yellow dots medially on posterior margin. Pre-episternum brown. Pteropleura mostly brown, with yellow regions dorsal- and ventrally. ***Foreleg*.** Coxa pale brown, yellow at base, setae yellow to pale brown; trochanter pale brown ventrally, yellow dorsally. Femur mostly yellow, with pale brown suffusions at the base and on area adjacent to closing surface on posterior surface; anterior surface yellow with brown suffusions at base, setae mostly yellow. Tibia mostly greyish brown, yellow on apical portion, setae mostly pale brown, clavate setae yellow. Basitarsus pale brown with dark brown apex, clavate setae yellow; second to fourth tarsomere pale brown. ***Mid- hind leg*.** Mid-leg with coxa brown, trochanter yellow with brown infuscation on posterior surface, femur yellow, tibia yellow with basal 1/3 pale brown; tarsus yellow. Hind leg with coxa mainly brown, except ventrally yellow, trochanter and femur yellow, tibia with basal ½ pale brown; tarsomeres yellow with pale brown infuscations on dorsal surface. ***Wings*.** Forewing hyaline, with small area adjacent to 2m-cu and first branch of CuA smoky; pterostigma brown laterally and yellow at center; venation mainly brown, except C, RA, basal ½ of CuA and anal veins alternating yellow and brown, base of CuP yellow; posterior and distal margin alternating brown and yellow. Hind wing hyaline, with small smoky area surrounding base of first CuA branch; pterostigma brown with small, preapical, yellow area; main veins mostly brown, except base of Cu and A2 with yellow regions; posterior margin alternating yellow and brown, anterodistal margin brown. ***Abdomen*.** Tergites of abdominal segments I and II brown, brown on segments III–VIII brown with anterolateral yellow regions; ventral margin of tergites VII and VIII yellow in male; female with tergites yellow with broad brown posteromedial spot. Sternite of first abdominal segment yellow, abdominal segments II–VII with a broad yellow band on center and brown lateral regions. Pleural membrane dark brown with yellow areas. Terminal segments mostly brown.

***Morphology* (Fig. 103)**. ***Head*.** Diamond-shaped in frontal view, sparsely setose, smooth; vertexal region domed above compound eyes, with reclined setae; supra-antennal region slightly raised; paraocular area concave; coronal suture discrete. Compound eye hemispheric, as wide as ¾ of interocular distance at torulus level. Postgena broad, hypostomal bridge not completely fused with it. Antenna moniliform, scape slightly longer than wide; flagellum slightly dorsoventrally compressed, with 37 flagellomeres; those of basal ½ 1.5× as wide as long, the rest almost as wide as long; all flagellomeres with a medial row of thickened setae. Frons trapezoidal, narrow; clypeus rectangular; labrum semicircular labrum. Maxillary palpus with first two palpomeres as long as wide, third palpomere ~ 3× as long as wide, fourth 2× as long as wide, fifth palpomere as long as third. Submentum oval, palpus with first palpomere slightly longer than wide, second palpomere 3× as long as wide, third palpomere slightly longer than second, notably widened; palpimacula broadly ovoid. ***Thorax*.** Pronotum 1.5× as long as wide, with furrow contiguous to the lateral and anterior margins; in lateral view anterior margin, preapical region and posterior margin slightly elevated, with pedicellate, thickened setae; episternum with long thin setae, postfurcasternum trapezoidal. Mesonotum 1.5× as wide as long, with a few scattered thickened setae; metanotum 1.5× as wide as long, scutellum with a few long, fine setae. Pteropleura covered with abundant long, thin setae. ***Foreleg*.** Coxa slightly shorter than femur, cylindrical, with abundant long, thin setae; trochanter subtrapezoidal; femur robust, with long and thin setae; closing surface with double row of tubercle-shaped specializations fully developed; both rows with a more developed sub-basal process (process/seta ratio 1:1); rows of thickened setae with globular base, adjacent to each row of processes present, extending almost the entire length of the closure surface. Tibia almost as long as the femur, curved, setose, closing surface with flattened, prostrate setae; with a patch of clavate setae apically on anterior surface; basitarsus elongated, ventrally keeled and with a row of prostrate setae; proximal ½ of anterior surface with clavate setae; lanceolate process reaching the base of fourth tarsomere, with plug-shaped Stitz organ at apex; second tarsomere 4× as long as wide, third tarsomere as long as wide, fourth tarsomere 2× as long as wide. ***Mid- and hind leg*.** Mid-leg covered with abundant long, thin setae, shorter on tarsus; basitarsus 3× as long as wide, second tarsomere 1.5× as long as wide, third and fourth tarsomere as long as wide, fifth tarsomere 2× as long as wide; the first four tarsomeres with two pairs of thickened setae, laterally, on distal margin of plantar surface. Hind leg longer than mid-leg, setose, tibia 1.5× as long as femur; tarsomeres to those of the mid-leg. ***Wings*.** Forewing oval, venation setose, trichosors present along wing margin except at base; costal space slightly widened medially; humeral vein sometimes forked; 12–14 subcostal veinlets; pterostigma elongated and narrow, composed of numerous veinlets, mainly incomplete; subcostal space with a single medially located crossvein; Sc vein bent posteriad at proximal margin of pterostigma to merge the RA; *rarp2* curved, with two or three veins arising from it, three or four from *rarp1*; M basally fused to R; base of RP located near separation of M and R, M fork near such separation, opposite to R fork; 1r-m located between RP base and MA base, forming a small trapezoidal cell; five or six gradate crossveins present. Cu vein deeply forked; CuP basally angled, approaching A1, distally forked. 1A simple, reaching the level of separation of M and R; A1 distally forked. Hind wing smaller and narrower than forewing; costal space narrow and reduced, with 4–6 veinlets; Sc fused to C at 1/5 of wing length, distally curved posteriad at proximal pterostigma margin to merge RA; pterostigma elongated, narrow, curved, composed of numerous incomplete veinlets; radial space with a single crossvein, oblique; two or three veins arising from *rarp1*, one or two from *rarp2*; three gradate crossveins present. M vein forked beyond the R fork; 1 r-m sigmoid, connecting M base and RP base, distally connected again to M stem through short crossvein. Cu deeply forked, CuA long and sinuous, distally forked, the first branch candelabrum-shaped; CuP distally forked, distally, anteriorly curved, pectinate. Cubitoanal space with two crossveins. A1 arched, A2 short and arched. ***Abdomen*.** Cylindrical, tergites quadrangular; intertergal membranes between segments III–V of male covered with microtrichia, with two glabrous lateral areas; sternites of segments I and II trapezoidal, the rest rectangular.

**Figure 104.**
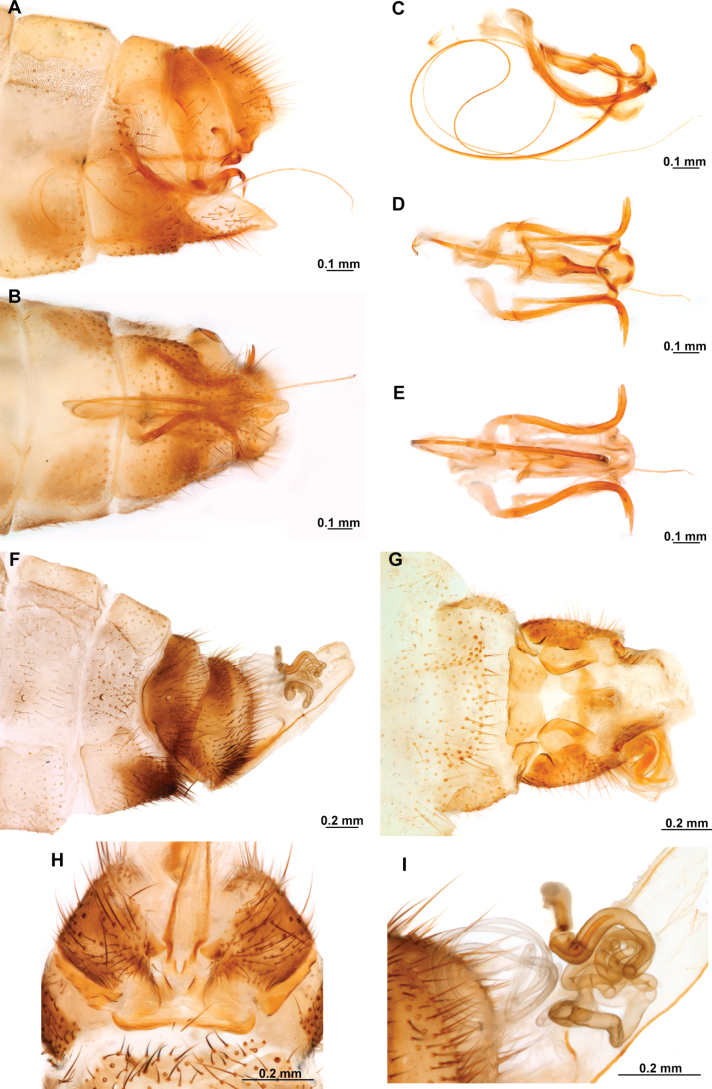
*Trichoscelianassonovi* (Navás, 1912) **A** male terminalia, lateral **B** same, ventral **C** male genitalia, lateral **D** same, dorsal **E** same, ventral **F** female terminalia, lateral **G** same, ventral **H** gonapophyses VIII ventral **I** spermatheca.

***Male genitalia*** (Fig. 104A–E). Tergite IX narrower dorsally than laterally, posteroventrally forming a curved process, continuous by slightly sclerotized, concave area. Sternite VII subrectangular, posterior margin concave, posterior corners rounded; sternite IX with abundant long and thin setae, pentagonal, posteromedially produced in a blunt lobe, dorsally canaliculate, acuminate in lateral view reaching posterior margin of ectoproct. Gonocoxite IX thin, short, strongly curved medially, base spatulate, inwardly curved; apex curved lateroventrally with two processes, of which the shorter, preapical one lies dorsally. Ectoproct ovoid, setose; ventral surface with quadrangular outgrowth, strongly sclerotized on anterior corner; posteriorly on inner surface with concave, sclerotized area. Gonocoxites X forming a straight, thickened, ventrally canaliculate sclerite; anterior apex widened; posterior apex with short dorsal processes and two preapical lateral processes. Gonostyli X with curved, thickened base with two lateral processes; the rest of the structure long, curved ventrally and coiled anteriorly, forming two loops before protruding from abdomen. Gonapophyses X straight, narrow, with posterior apex bent dorsally with surrounding membrane set with minute spinules; gonapophyses subparallel, joined by a membrane covering base of gonostyli X. Gonocoxites XI U-shaped, medial lobe with two differentiated parts; dorsal part as an arch composed of two parts medially fused; ventral part consisting of a convex area with a broadly incised, U-shaped ventral edge; between these parts a narrow, less sclerotized region is present. Lateral arms of gonocoxites XI straight.

***Female genitalia*** (Fig. 104F–I). Sternite VII rectangular. Tergite VIII medially slightly narrower than laterally, enclosing the spiracle of the eighth abdominal segment; lateral margin forming an acute angle. Membrane between tergite VIII and tergite IX + ectoproct sclerotized, forming a concave, triangular plate. Gonocoxites VIII forming a trapezoidal plate, with two lateral concavities; gonapophyses VIII with medial part folded, quadrangular, with posteromedial bifid process; lateral part as an oval plate, with convex ventral surface, covered with microspinules, connected to tergite IX + ectoproct. Tergite IX + ectoproct broadly ovoid. Gonocoxite IX long, sinuous and narrow, as long as the last four abdominal segments together. Bursa copulatrix membranous, long, thin, proximal part sclerotized and concave; spermatheca entangled; proximal section long, thin, forming four coils, ending in several convolutions; medial section thicker than proximal section, entangled, forming multiple convolutions. Distal section thin, forming several convolutions, progressively wider towards apex, distally with a trumpet-shaped invagination. Fertilization canal duct short, thin, and sigmoid; fertilization canal short, thickened, pod-shaped, covered with microfilaments.

##### Distribution.

Bolivia (Cercado, La Paz, Santa Cruz), Brazil (Amazonas, Ceará, Rondônia), Ecuador (Napo), French Guiana, Peru (Huánuco, Ica, Lima, Madre de Dios), Panama (Canal Zone), Trinidad and Tobago (Couva-Tabaquite-Talparo, Tunapuna-Piarco).

##### Remarks.

This is probably the most common and widespread species of *Trichoscelia* in Northern South America. It was originally described from Peru (probably Ica) and was until now known only by the lectotypes. Herein, the first records from Bolivia, Brazil, Ecuador, French Guiana, and Trinidad and Tobago are presented. This species was also previously registered from Panama as *T.iridella* by Ardila-Camacho et al. (2018). Additionally, herein the range of distribution in Peru is extended to the provinces of Huánuco, Lima, and Madre de Dios.

Most of the records presented by Penny (1982a) of *T.iridella* are probably of this species, although because it was not possible to study the specimens of this genus deposited in the Instituto Nacional de Pesquisas da Amazônia (INPA), it is not possible to corroborate the identifications made by this author.

This species is sometimes difficult to separate from other Amazonian congeners due to its general coloration pattern, sometimes with completely hyaline wings. The coloration of the pterostigma and the apically bifid male gonocoxites IX can help to recognize this species easily.

#### ﻿﻿ Trichoscelia pennyi

Taxon classificationAnimaliaNeuropteraRhachiberothidae

Ardila-Camacho & Contreras-Ramos sp. nov.

https://zoobank.org/B5D60875-1171-47F5-8CC1-91FE53485397

Figs 105 , 106

##### Type locality.

Ecuador, **Napo**: 20 Km E Puerto Napo, Aliñahui, 1°00'S, 77°25'W, elev. 450 m, Jul. 1995, E. Ross leg.

##### Material examined.

***Holotype*** male, pinned. Original label: “Ecuador, **Napo**, 20 Km E Puerto Napo, Aliñahui, 1°00'S, 77°25'W, elev. 450 m, XII.1995, E. Ross leg.” CAS. ***Paratypes*.** Ecuador • 1 ♂; **Napo**, 20 Km E Puerto Napo, Cabañas Aliñahui; 01°00'S, 77°25'W; 450 m; 17 Dec. 1995; E.S. Ross leg.; CAS. • 1 ♀; same data as for preceding; 1°03'S, 77°40'W; Sep.1997; E.S. Ross leg.; LDL#0517; CAS.

##### Other material.

Colombia • 1 ♂; **Meta**, Villavicencio, Vda. El Cairo y alrededores; 1100 m; 07 Apr. 2004; O. Vargas leg.; UPN.

##### Diagnosis.

This species is separated from others in the genus because the pronotum has an inverted Y-shaped, brown mark. On the male genitalia, the sternite IX is pentagonal, and posteromedially forms an acute angle. The gonocoxite IX is short and thickened, with three digitiform processes on the posterior apex, which are strongly recurved and somewhat intertwined. On the female genitalia, the gonapophyses VIII form a narrow and arched sclerite, medially set with two lateral, blunt processes.

##### Etymology.

This species is named after the prominent Neuropterologist Norman D. Penny, who first addressed the taxonomy of the subfamily Symphrasinae in the Amazonia in a revisionary way, allowing other researchers progress in the study of this complex group.

##### Description.

***Measurements*.** Male (*n* = 2). Forewing length: 7.9–8.3 mm; Hind wing length: 6.3–6.6 mm. Female (*n* = 1): Forewing length: 8.4 mm; Hind wing length: 6.5 mm.

**Figure 105.**
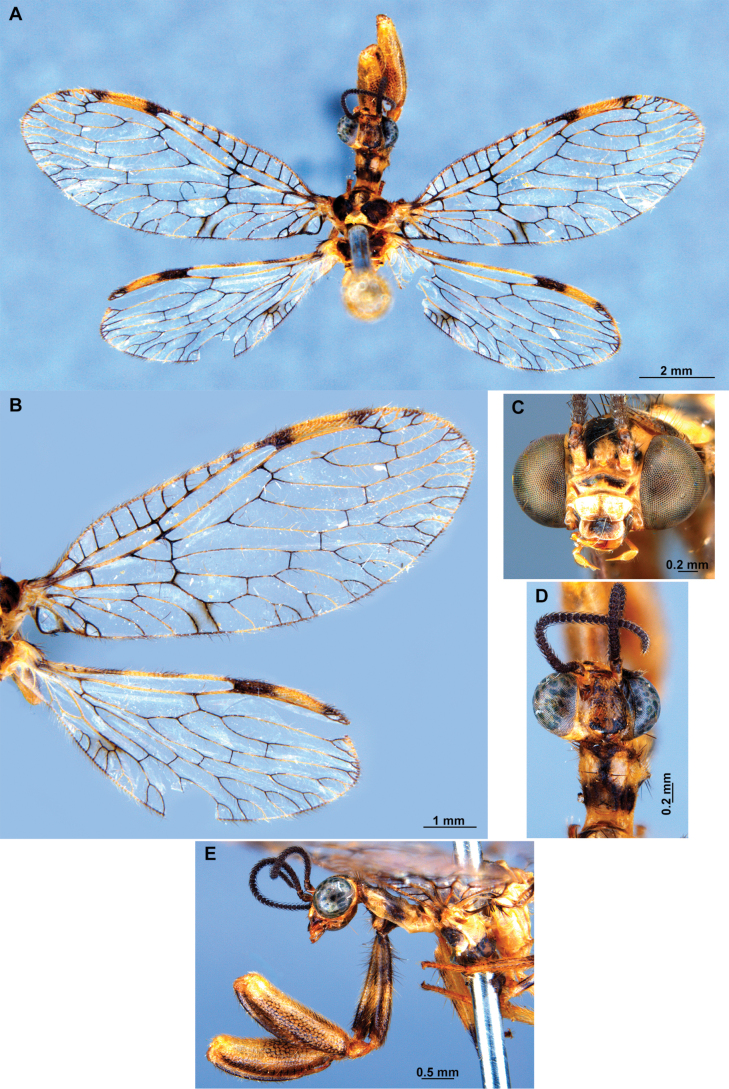
*Trichosceliapennyi* Ardila-Camacho & Contreras-Ramos, sp. nov. **A** male habitus, dorsal **B** wings **C** head, frontal **D** pronotum, dorsal **E** forelegs, lateral.

***Coloration* (Fig. 105)**. ***Head*.** Mainly yellow, vertexal region with broad, longitudinal, dark brown band extended from occipital ridge to supraantennal area where it forks, with yellow setae. Antennal scape yellow at base, brown on the rest; pedicel brown; flagellum brown. Frons brown at center, yellow at periphery; clypeus yellow, labrum mostly brown, yellow at margins; mandible brown. Maxilla yellow with brown areas, palpus pale brown. Labium yellow, labial palpus with basal article dark brown, the rest pale brown, palpimacula pale brown. ***Thorax*.** Pronotum yellow with inverted Y-shaped brown mark; anterior margin of pronotum brown; episternum bicolor, brown on anterior ½, yellow on posterior ½; postfurcasternum yellow. Mesonotum with lateral, dark brown areas; anterior area to parapsidal suture yellow with lateral yellow areas; scutellum yellow. Metanotum mostly dark brown. Meso-pre-episternum dark brown. Mesopleuron yellow with broad, brown areas; metapleuron mostly yellow. ***Foreleg*.** Coxa mostly brow, with small yellow areas; trochanter yellow with brown suffusions. Femur yellow, with wide, brown, honey-comb-shaped pattern on anterior and posterior surfaces; posterior surface with area adjacent to closing surface brown. Tibia mostly brown. Tarsus pale brown. ***Mid- and hind leg*.** Mid-leg with brown coxa, trochanter yellow; femur yellow with sub-basal brown ring; tibia mostly brown; tarsus yellow becoming pale brown towards the apex. Hind leg with coxa and trochanter yellow; femur as in mid-leg; tibia yellow with brown base; tarsus as in mid-leg. ***Wings*.** Forewing hyaline, sometimes with amber on area adjacent to first branch of CuA and apex of CuP; pterostigma brown with wide, yellow, medial area; longitudinal veins alternating dark brown and yellow, crossveins and subcostal veinlets brown; posterior and distal margin alternating brown and yellow. Hind wing hyaline, sometimes with amber on area adjacent to stem of first branch of CuA; pterostigma brown with yellow, preapical area; venation mostly alternating brown and yellow, except for most of the Cubital and anal veins yellow; posterior and distal margin alternating yellow and brown. ***Abdomen*.** Cleared.

***Morphology* (Fig. 105)**. ***Head*.** Diamond-shaped in frontal view, moderately setose, smooth; vertexal region domed above compound eyes; paraocular area concave; coronal suture discrete. Compound eye hemispheric, as wide as ¾ interocular distance at torulus level. Broad postgena, hypostomal bridge not completely fused with it. Antenna submoniliform, scape 1.5× as long as wide, slightly distally expanded; pedicel as long as wide; flagellum slightly dorsoventrally compressed, with 39 flagellomeres, those of basal ½ 3× as wide as long, the rest almost as wide as long, progressively smaller towards apex; all flagellomeres with a medial ring of thickened setae. Maxillary palpus the first two palpomeres as long as wide, third palpomere 4× as long as wide, fourth ~ 2× as long as wide, fifth palpomere tapering, slightly shorter than the third. Postmentum oval with long setae; labial palpus with first palpomere 1.5× as long as wide, second 3× as long as wide, third as long as the second, widely expanded, palpimacula broadly ovoid. ***Thorax*.** Pronotum 1.5× as long as wide, with furrow contiguous to lateral and anterior margins; in lateral view anterior margin, medial region, and posterior margin slightly elevated, set with thickened, pedicellate setae; episternum with fine setae, postfurcasternum trapezoidal. Mesonotum as long as wide, with thick, pedicellate on medial area. Metanotum ~ 2× as wide as long, glabrous. Pteropleura covered with abundant thin setae. ***Foreleg*.** Coxa slightly shorter than femur, cylindrical, with abundant, interspersed long and thin setae; trochanter subtrapezoidal; femur robust, with short and thin setae; closing surface with double row of tubercle-shaped specializations fully developed, both with a more developed sub-basal process (process/seta ratio 1:1); rows of thickened setae with globular base adjacent to each row of processes present, extending over almost the entire closing surface. Tibia almost as long as femur, curved, with short and thin setae; closing surface with prostrate setae; anterior surface with a patch of clavate setae at apex; basitarsus elongated, ventrally keeled and with a row of prostrate setae on proximal ½, anterior surface with a patch of clavate setae proximally; lanceolate process, reaching the base of fourth tarsomere, with plug-shaped Stitz organ at apex; second tarsomere 3× as long as wide, third tarsomere as long as wide, fourth tarsomere 2× as long as wide. ***Mid- and hind leg*.** Coxae covered with long, thin setae; trochanter with short setae; femora with interspersed short and long, thin setae; tibiae with abundant long, thin setae; on both legs, basitarsus 4× as long as wide, second tarsomere slightly longer than wide, third and fourth as long as wide, fifth tarsomere two times as long as wide, all covered with short, thin setae; the first four tarsomeres with a pair of thickened setae laterally on distal margin of plantar surface. Hind leg longer than mid-leg, tibia 1.5× as long as femur; tarsomeres similar to those of mid-leg. ***Wings*.** Forewing oval, trichosors present along wing margin except at wing base, venation setose; costal space slightly widened medially, humeral vein simple or forked, 7–11 subcostal veinlets; pterostigma elongated and narrow, composed of numerous veinlets, mainly incomplete; subcostal space with a single, medially located crossvein; Sc vein bent posteriad at proximal margin of pterostigma to merge the RA; *rarp2* curved, with two or three veins arising from it, two or three from *rarp1*; M fused basally to R; RP base located near separation of M and R, M fork near such separation, opposite to R fork; 1r-m located between RP base and MA base, forming a small trapezoidal cell; four gradate crossveins present. Cu vein deeply forked; CuP basally angled, approaching A1, distally forked, slightly before to separation of M and R. A1 simple, reaching the level of CuP fork; A2 distally forked, at level of CuP angle. Hind wing smaller and narrower than forewing; costal space narrow and reduced, with four or five veinlets; Sc and C fused at 1/5 of wing length, Sc abruptly curved posteriad at proximal margin of pterostigma, to merge the RA; pterostigma elongated, narrow, curved, composed of numerous incomplete veinlets; radial space with a single crossvein, oblique; two veins arising from *rarp1*, one from *rarp2*; two or three gradate crossveins present. M forked beyond R fork; 1 r-m sigmoid, connecting M base and RP base, distally connected again with M stem through short crossvein. Cu deeply forked, CuA long and sinuous, distally forked, first branch candelabrum-shaped; CuP distally anteriorly curved, pectinate. Cubitoanal space with two crossveins. A1 simple, A2 short and arched. ***Abdomen*.** Cylindrical, tergites quadrangular; sternites rectangular.

**Figure 106.**
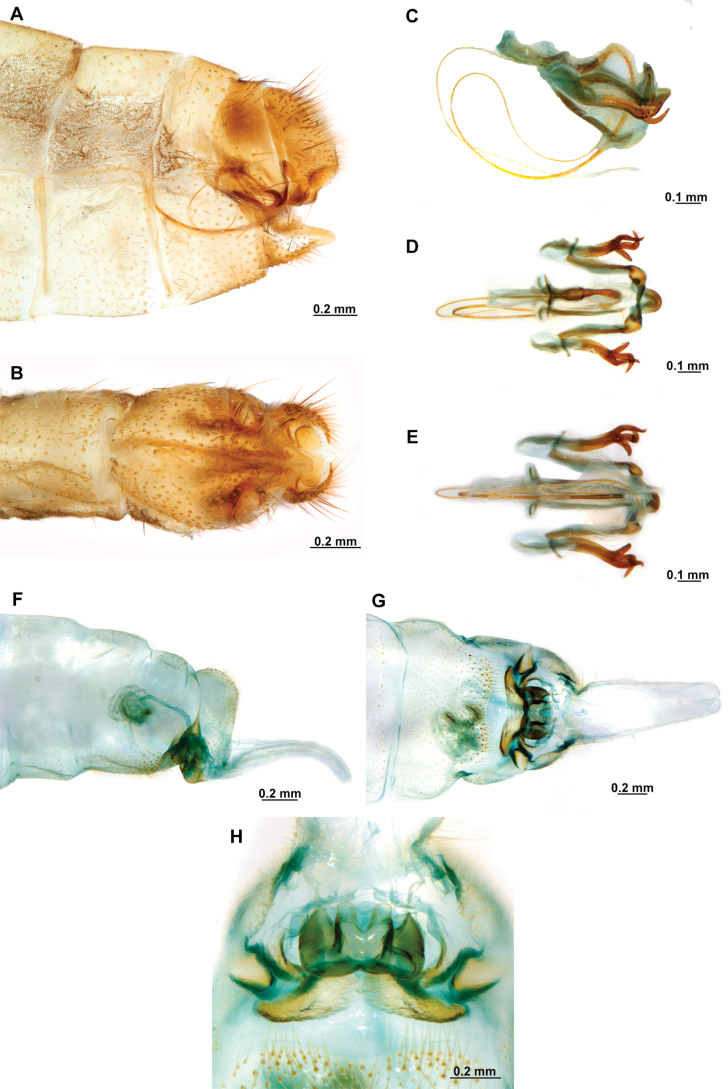
*Trichosceliapennyi* Ardila-Camacho & Contreras-Ramos, sp. nov. **A** male terminalia, lateral **B** same, ventral **C** male genitalia, lateral **D** same, dorsal **E** same, ventral **F** female terminalia, lateral **G** same, ventral **H** gonapophyses VIII ventral.

***Male genitalia*** (Fig. 106A–E). Tergite IX narrower medially than laterally, lateral margin rounded, with posteroventral notch. Sternite VIII subtrapezoidal; sternite IX pentagonal, posteromedially forming an acute angle, glabrous, dorsally canaliculated, remaining surface with abundant thin setae; in lateral view acuminate, with apex not reaching the posterior margin of ectoproct. Gonocoxite IX short and thickened, base spatulate; apex with three digitiform, subequal processes, which are strongly recurved and somewhat intertwined. Ectoproct subtrapezoidal, setose; ventral surface with sclerotized, anterior lobe, posteriorly continuous by wide concavity. Gonocoxites X forming a short, thickened, ventrally canaliculate sclerite; anterior apex thicker, posterior apex bilobed, and with two preapical, lateroventral processes. Gonostyli X with thin and curved base, with two lateral processes, the rest of the structure long, curved ventrally and coiled anteriorly, forming two internal loops before protruding from the abdomen. Gonapophyses X rod-shaped, straight, posterior apex dorsally bent; gonapophyses subparallel, joined by membrane covering gonostyli X. Gonocoxites XI U-shaped, medial lobe expanded and elaborated with two differentiated parts: dorsal part as a quadrangular lobe; ventral part as a convex area covered with microspinules, ventral margin V-shaped; between these parts a narrow, less sclerotized region is present. Lateral arms of gonocoxites XI sigmoid, with anterior apex latero-ventrally bent.

***Female genitalia*** (Fig. 106F–G). Sternum VII subtrapezoidal. Tergite VIII slightly narrower dorsally than laterally, enclosing the spiracle of the eighth abdominal segment, lateral margin D-shaped. Membrane between tergite VIII and tergite IX + ectoproct sclerotized, forming a concave, triangular plate. Gonocoxites VIII forming a narrow plate, with two lateral concavities; medial part of gonapophyses VIII narrow, arched, medially with two lateral, blunt processes; lateral part as a small, trapezoidal, concave plate. Tergite IX + ectoproct ovoid. Gonocoxite IX long and narrow, as long as the last four abdominal segments together; gonapophyses IX as tiny sclerites situated on inner surface of the base of gonocoxites IX. Bursa copulatrix funnel-shaped, membranous, long, and thin; spermatheca complex and entangled; proximal section long, thin, forming four coils; medial section thicker than proximal section, forming multiple convolutions; distal section progressively wider towards apex, distally with a trumpet-shaped invagination. Fertilization canal duct short, thin, sigmoid; fertilization canal short, J-shaped, covered with microfilaments.

##### Distribution.

Colombia (Meta), Ecuador (Napo).

##### Remarks.

This species is distributed in Colombia and Ecuador, in the provinces (or departments) of Meta and Napo, respectively, with records near the eastern slope of the Andes. This species has a body coloration pattern markedly similar to that of *T.andina* and *T.banksi*, but the genitalic morphology immediately allies this species with the latter species, so both are probably closely related.

#### ﻿﻿ Trichoscelia sequella

Taxon classificationAnimaliaNeuropteraRhachiberothidae

(Westwood, 1867)

Figs 107 , 108

Mantispa (Trichoscelia) sequella Westwood, 1867: 503. Holotype: female, “Amazonas” [Probably Brazil], no specific locality (OUMNH), photographs examined.
Trichoscelia sequella (Westwood, 1867). Penny (1982a): 435.

##### Material examined.

***Holotype*.** Brazil • ♀; **Amazonas**; H.W. Bates leg.; 1861; Type Neur.: No. 9, *MantispaTrichosceliasequella* Westwood, HOPE Dept. Oxford; Type, Westwood Trans. Ent. Soc. 1867, p. 503, Coll. Hope Oxon; OUMNH.

##### Other material.

Brazil • 1 ♀; **Amazonas**, Fonte Boa; 1554; *Trichosceliasequella* Westwood; McLachlan Coll. B.M. 1938-674; NHMUK 013802782; NHMUK. • 1 ♂; Ega; Bates; *Trichosceliasequella* Westwood; McLachlan Coll. B.M. 1938-674; NHMUK 013802783; NHMUK.

Colombia • 1 ♀; **Vichada**, Gaviotas; 4°33'48"N, 70°55'18"W; 180 m; 20 Aug. 1995; F. Cortés leg.; bosque de galería; Malaise, IAvH-E 115981; *Trichosceliakarijona*, det. A. Ardila, 2015; IAvH.

##### Diagnosis.

This species is recognized from *T.karijona* by its yellow body coloration pattern. The forefemur has a dorsoproximal brown area that forms a honeycomb pattern. The wing venation is nearly completely brown, and the pterostigma of both wings are dark brown. On the male genitalia, the gonocoxite IX is remarkably short, thickened, with posterior apex set with three or four subequal digitiform processes, hand-like arranged and laterally curved. On the female genitalia, the gonapophyses VIII medial part forms a rectangular, convex plate, with a posteromedial bifid process; the lateral part is a bulging, rounded plate covered with microtrichia.

##### Description.

***Measurements*.** Male (*n* = 1). Forewing length: 6.9 mm; Hind wing length: 5.2 mm. Female (*n* = 2): Forewing length: 7.6–7.7 mm; Hind wing length: 5.9–6.1 mm.

**Figure 107.**
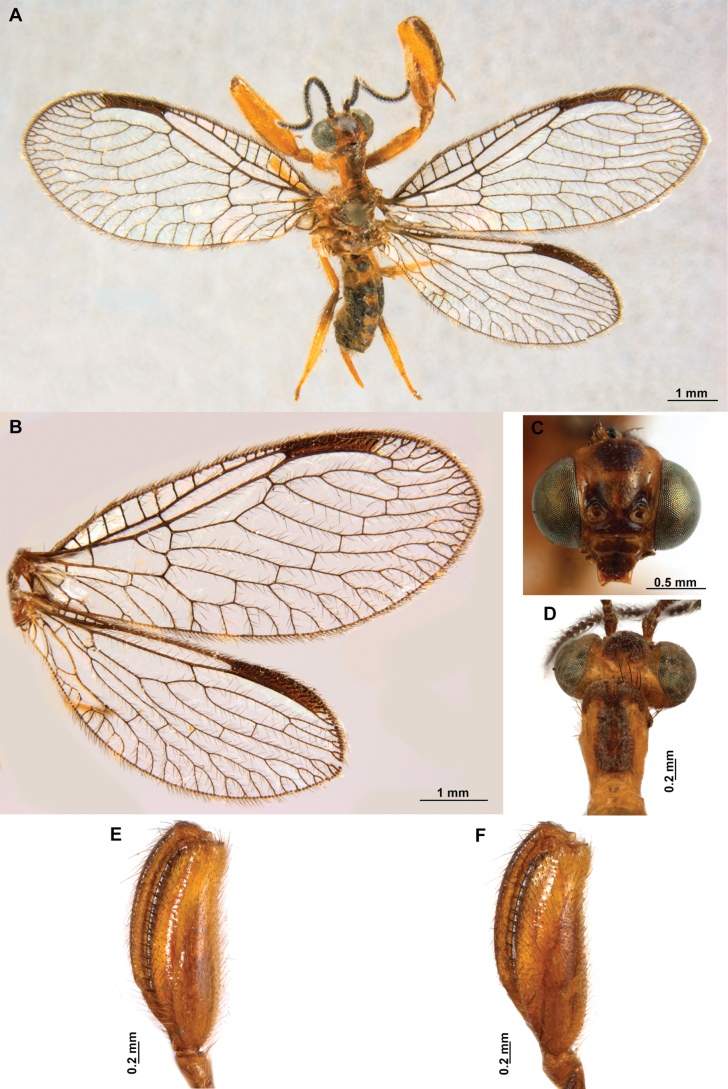
*Trichosceliasequella* (Westwood, 1867) **A** female habitus, dorsal **B** wings **C** head, frontal **D** pronotum, dorsal **E** forefemur, anterior surface **F** same, posterior surface.

***Coloration*** (Fig. 107). ***Head*.** Mainly yellow, vertexal region with longitudinal brown band extending from occipital ridge to supraantennal area, laterally reaching paraocular area; with pale brown setae. Antenna brown flagellum. Frons brown with yellow lateral and anterior margins, clypeus brown with yellow margins, labrum brown; mandible pale brown. Maxilla yellow, with pale brown palpus; labium yellow with pale brown setae on postmentum, labial palpus pale brown, palpimacula yellow. ***Thorax*.** Pronotum yellow with brown longitudinal, medial, narrow band and anterior margin; episternum bicolor, brown on anterior ½, yellow on posterior ½, postfurcasternum yellow. Mesonotum with predominantly pale brown scutum, yellow on area adjacent to sutures, scutellum laterally yellow, pale brown at center; metanotum yellow, pale brown at center. Pre-episternum yellow, with brown at center. Pteropleura yellow. ***Foreleg*.** Coxa yellow with the proximal ½ brown; trochanter pale brown. Femur yellow, with wide brown area on posterior and dorsal surfaces; setae mostly yellow. Tibia brown, setae mostly yellow. Basitarsus pale brown, clavate setae pale brown; second to fourth tarsomere pale brown. ***Mid- and hind leg*.** Mid-leg with coxa, trochanter and femur yellow; tibia brown with yellow apex; tarsus yellow, plantar surface with pale brown setae on the distal margin. Hind leg with coxa, trochanter and femur yellow, tibia brown on proximal ½, yellow on distal ½; first four tarsomeres yellow, fifth tarsomere brown. ***Wings*.** Forewing hyaline; pterostigma dark brown; venation dark brown, except at the base of Cu paler; posterior margin with yellow areas on proximal 1/3. Hind wing hyaline; pterostigma dark brown; venation dark brown, except for base of CuA paler; posterior margin with pale brown areas on proximal 1/3. ***Abdomen*.** Tergites yellow with brown posteromedial area. Pleural membrane dark brown. Sternites yellow with brown lateral areas.

***Morphology*** (Fig. 107). ***Head*.** Diamond-shaped in frontal view, moderately setose, smooth, vertexal area domed above compound eyes, paraocular area concave; coronal suture discrete. Antenna moniliform, scape ~ 2× as wide as long, slightly distally expanded; pedicel as long as wide; flagellum slightly dorsoventrally compressed, with 38 flagellomeres, those of basal ½ 1.5× as wide as long, the rest almost as wide as long, progressively smaller towards flagellar apex; all flagellomeres covered with medial ring of thickened setae. Compound eye, hemispherical, as wide as interocular distance at torulus level. Postgena broad, hypostomal bridge not completely fused with it. Maxillary palpus with first two palpomeres as long as wide, third palpomere 3× as long as wide, fourth ~ 2× as long as wide, fifth slightly shorter than third. Submentum oval with long setae, labial palpus with first palpomere 1.5× as long as wide, second 3× as long as wide, third as long as second, widely expanded, palpimacula widely ovoid. ***Thorax*.** Pronotum 1.5× as long as wide, with furrow contiguous to lateral and anterior margins; in lateral view anterior margin, medial region and posterior margin slightly elevated, with thickened, pedicellate setae; episternum with long fine setae, postfurcasternum trapezoidal. Mesonotum 1.5× wider than long, with thick, pedicellate setae on medial area. Metanotum ~ 2× as long as wide, mostly glabrous. Pteropleura covered with abundant short, thin setae. ***Foreleg*.** Coxa slightly shorter than femur, cylindrical, with abundant long and thin setae; trochanter subtrapezoidal, with protuberance on anterior surface, covered with short, thin setae; femur robust, covered with short and thin setae; closing surface with double row of tubercle-shaped specializations fully developed, posteroventral row with a more developed sub-basal process (process/seta ratio 2:1), anteroventral row with a more developed basal process (process/seta ratio 2:1) ; rows of thickened setae with globular base adjacent to each row of processes extending over almost the entire closing surface. Tibia almost as long as femur, curved, setose, closing surface with prostrate setae; with patch of clavate setae apically on anterior surface; basitarsus elongated, ventrally keeled and with a row of prostrate setae on proximal ½, anterior surface with patch clavate setae proximally; lanceolate process reaching the base of the fourth tarsomere, with a plug-shaped Stitz organ at apex; second tarsomere 4× as long as wide, third tarsomere as long as wide, fourth tarsomere 2× as long as wide. ***Mid- and hind leg*.** Mid-leg with coxa and trochanter covered with long, thin setae; tibia and femur with short, thin setae; basitarsus 3× as long as wide, second tarsomere slightly longer than wide, third and fourth as long as wide, fifth tarsomere ~ 2× as long as wide, all covered with short, thin setae; the first four tarsomeres with a pair of thickened setae laterally on distal margin of plantar surface. Hind leg longer than mid-leg, setose; tibia 1.5× as long as femur; tarsomeres similar to those of mid-leg. ***Wings*.** Forewing oval, trichosors present along wing margin, except at base, venation setose; costal space narrow, humeral vein forked, 9–11 subcostal veinlets; pterostigma elongated and narrow, composed of numerous veinlets, mainly incomplete; subcostal space with single, medially located crossvein; Sc vein curved posteriad at proximal margin of pterostigma to merge the RA; *rarp2* curved, with two or three veins arising from it, two from *rarp1*; M fused basally to R; RP base located near separation of M and R, M fork near such separation, opposite to R fork; 1r-m located between RP base and MA base, forming small trapezoidal cell; four gradate crossveins present. Cu vein deeply forked; CuP basally angled, approaching A1, distally forked, slightly before separation of M and R. A1 simple, reaching the level of CuP fork; A2 distally forked, at level of CuP angle. Hind wing smaller and narrower than forewing. Costal space narrow and reduced, with four veinlets; Sc and C fused at 1/5 of wing length, Sc curved posteriad at proximal margin of pterostigma, to merge the RA; pterostigma elongated, narrow, curved, composed of numerous veinlets; radial space with single crossvein, oblique; two veins arising from *rarp1*, one from *rarp2*; two or three gradate series present. M forked slightly beyond R fork; 1 r-m sigmoid, connecting M base and RP, distally connected again to M stem through short crossvein. Cu deeply forked, CuA long and sinuous, distally forked, first branch generally candelabrum-shaped; CuP distally anteriorly curved, pectinate. Cubitoanal space with two crossveins. A1 arched, A2 short and arched. ***Abdomen*.** Cylindrical, tergites quadrangular; intertergal membranes between segments III–VI in male and III–V in female broad, covered with microtrichia; sternites rectangular.

**Figure 108.**
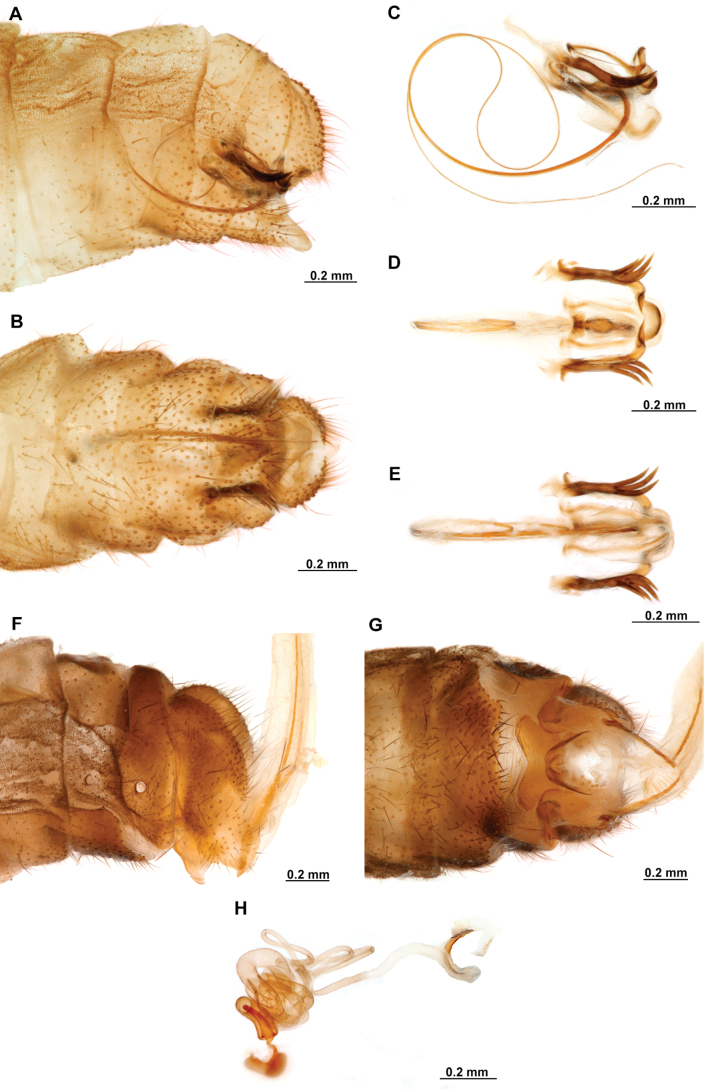
*Trichosceliasequella* (Westwood, 1867) **A** male terminalia, lateral **B** same, ventral **C** male genitalia, lateral **D** same, dorsal **E** same, ventral **F** female terminalia, lateral **G** same, ventral **H** spermatheca.

***Male genitalia*** (Fig. 108A–E). Tergite IX dorsally slightly narrower than laterally, lateral margin rounded, with posterior notch. Sternite VIII subrectangular; Sternite IX triangular, lateral borders with small, medial emargination; posteromedially forming an obtuse angle, glabrous, dorsally canaliculated, the rest of the surface with abundant long, thin setae; in lateral view acuminate, reaching posterior margin of ectoproct. Gonocoxite IX remarkably short, thickened, base spatulate; apex with three or four subequal digitiform processes, hand-like arranged, laterally curved. Ectoproct semi-triangular, setose; ventral surface with convex, sclerotized anterior area, covered with microspinules, followed posteriorly by broad sclerotized groove. Gonocoxites X forming a short, thickened, ventrally canaliculate sclerite; anterior apex expanded; posterior apex bilobed, with two, short, preapical, lateroventral. Gonostyli X with thin and curved base, with two lateral processes, the rest of the structure long, ventrally curved and anteriorly coiled, forming two internal loops before protruding from abdomen. Gonapophyses X straight, thin, posterior apex dorsally bent with surrounding membrane set with minute spinules; gonapophyses arranged in a V-shape structure, joined by membrane covering base of gonostyli X. Gonocoxites XI U-shaped, medial lobe expanded and elaborate with two differentiated parts: dorsal part as a quadrangular lobe; ventral part as a convex area covered with microspinules, with U-shaped ventral edge; between these parts a narrow, less sclerotized region is present. Lateral arms of gonocoxites XI curved, with anterior apex laterally curved.

***Female genitalia*** (Fig. 108F–H). Sternite VII trapezoidal. Tergite VIII narrower medially than laterally, enclosing the spiracle of eighth abdominal segment, lateral margin triangular. Membrane between tergite VIII and tergite IX + ectoproct sclerotized, forming a concave, triangular plate. Gonocoxites VIII forming a trapezoidal plate with two lateral concavities; gonapophyses VIII with rectangular, convex medial part, with a posteromedial bifid process; lateral part as a bulging, rounded plate covered with microtrichia. Tergite IX + ectoproct ovoid. Gonocoxite IX long and narrow, as long as the last four abdominal segments together. Bursa copulatrix funnel-shaped, membranous, long, thin, proximally sclerotized, curved; spermatheca complex and entangled; proximal section long, thin, forming three coils, ending in an innermost spiral; medial section thicker than proximal section, entangled, forming multiple convolutions. Distal section thin, forming several convolutions, progressively wider towards apex, distally with trumpet-shaped invagination. Fertilization canal duct short, thin and sigmoid; fertilization canal short, thickened, pod-shaped, covered with microfilaments.

##### Distribution.

Brazil (Amazonas), Colombia (Vichada).

##### Remarks.

The label of the holotype female of this species has only “Amazonas” as collecting data. An additional male deposited in the Natural History Museum of London (NHMUK) was collected in Fonte Boa, in the state of Amazonas in Brazil. A male specimen from Guyana reported by Penny (1982a), was wrongly identified, and actually belongs to *T.karijona*. A female specimen from Colombia (Vichada) was reported by Ardila-Camacho and García (2015), although it was identified as *T.karijona*.

The separation between *T.sequella* and *T.karijona* is difficult as both species are quite similar. The separation requires a careful examination of the coloration and the spermatheca, but the male genitalia is markedly different between both species. *Trichosceliasequella* appears to be closely related to *T.pennyi* and *T.banksi* due to the overall morphology of the male genitalia, and particularly of the gonocoxites IX.

#### ﻿﻿ Trichoscelia tobari

Taxon classificationAnimaliaNeuropteraRhachiberothidae

(Navás, 1914)

Figs 109 , 110

Symphrasis tobari Navás, 1914: 26. Holotype: male, Mexico, Tabasco (NHMUK).
Trichoscelia tobari (Navás, 1914). Penny (1977): 37.

##### Material examined.

***Holotype*.** Mexico; ♂; **Tabasco**, Teapa; January; H.H.S., Godman-Salvin collection 1913-214; *Symphrasistobari* Nav. Navás S.J. det.; Typus red label; Type H.T.; Typus red label; NHMUK 01380276; NHMUK.

##### Other material.

Mexico – **Jalisco** • 1 ♀; Chamela Research Station; 19°29.873'N, 105°02.674'W; 08 Jul. 2003; 375’; F.G. Andrews leg.; at lights; CSCA. – **Oaxaca** • 1 ♂; 63 mi. W Tehuantepec; 21 Jul. 1952; E.E. Gilbert, C.D. MacNeil leg.; FSCA. • 1 ♂; Pochutla, Candelaria Loxichá, Santa Rita; 541 m; 15.93027, -96.53472; 12 Aug. 2018; F. Acevedo, A. Ramírez & D. Curoe leg.; CNIN. – **Quintana Roo** • 1 ♀; Bacalar, Limones; 18°59'26"N, 88°09'04"W; 21 m; 19 Jul. 2014; WACH-JJRR, trampa de luz-LED roja; CNIN. – **Veracruz** • 2 ♀; 30 mi. South Acayucan; 22 Apr. 1962; F.D. Parker, L.A. Stange leg.; *Trichosceliatobari* det., L. Stange, 1965; FSCA. • 1 ♀; 11.7 mi. E Xalapa, 2835; 23 Aug. 1967; Ball, T.L. Erwin, R.E. Leech leg.; U.V light; collection of R.G. Beard, MCZ-ENT 00681788; MCZ. • 1 ♂; Veracruz, Estación de Biología Los Tuxtlas; 120 m; 20 Feb. 1985; A. Ibarra leg.; 03387; CNIN.

##### Diagnosis.

This species is separated from other species from Mexico and Central America by the presence of three apical flagellomeres pale yellow. The forefemur is yellow with a broad, brown band adjacent to the closing area on the posterior surface. The forewing has the area surrounding crossveins, base of RP, M fork, first branch of CuA, and apex of CuP amber. On the male genitalia, the gonocoxite IX is short, curved, with posterior apex laterodorsally curved, and set with four digitiform processes, two apical, medially situated, and two preapical, laterally located. On the female genitalia, the gonapophyses VIII medial part is convex, keel-shaped, and posteromedially set with a short, thickened M-shaped process; the lateral part is a triangular, convex plate.

##### Description.

***Measurements*.** Male (*n* = 2). Forewing length: 6.3–7.3 mm; Hind wing length: 5.7–5.8 mm. Female (*n* = 3): Forewing length: 7.3–8.3 mm; Hind wing length: 5.5–6.4 mm.

**Figure 109.**
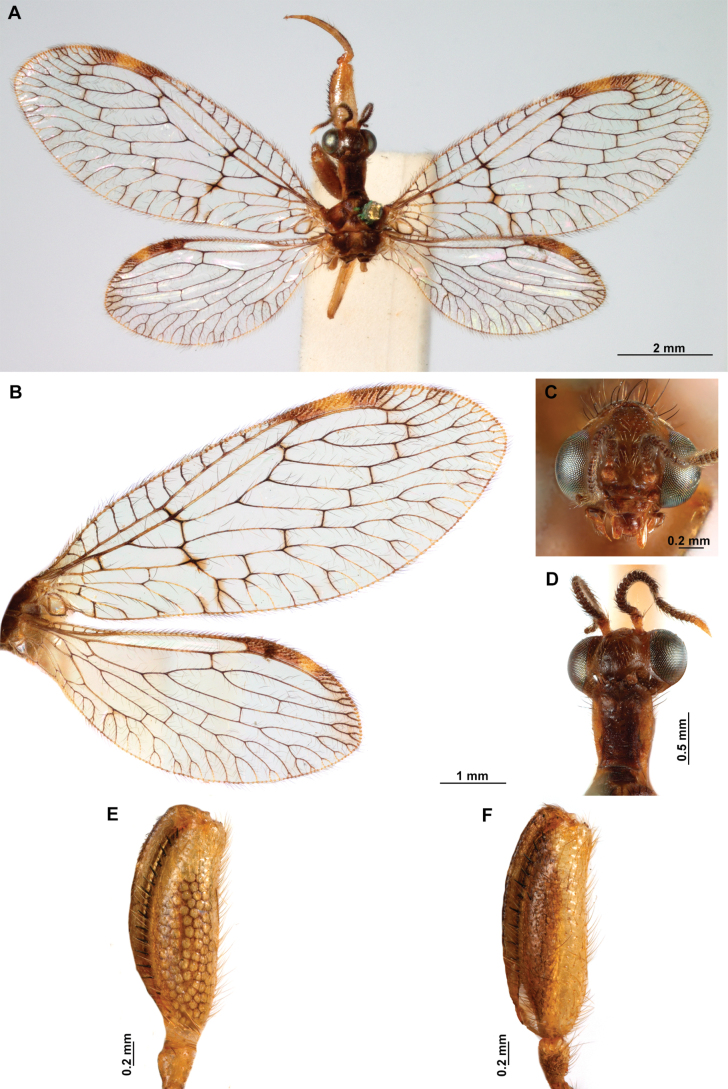
*Trichosceliatobari* (Navás, 1914) **A** male habitus, dorsal (abdomen removed) **B** wings **C** head, frontal **D** pronotum, dorsal **E** forefemur, anterior surface **F** same, posterior surface.

***Coloration*** (Fig. 109). ***Head*.** Mostly yellow, vertexal region with pale brown band, anteriorly forked, with abundant yellow setae. Hypostomal bridge brown. Antennal scape mainly yellow, distally with brown infuscations; pedicel brown with yellow areas; flagellum brown, with the last three flagellomeres pale yellow. Frons with two faint brown markings beneath toruli. Clypeus and labrum yellow with brown suffusions; mandible yellow at base, pale amber at apex. Maxilla yellow, maxillary palpus brown, with paler basal palpomere. Postmentum yellow with brown suffusions and pale brown setae, prementum yellow, ligula yellow with brown suffusions, labial palpus brown with yellow palpimacula. ***Thorax*.** Pronotum yellow with brown wide medial band and anterior margin, lateral areas with brown suffusions; episternum bicolor, brown and yellow, with pale brown setae; postfurcasternum pale yellow. Mesonotum yellow, scutum brown on area adjacent to wing bases, medially with two pairs of brown spots, scutellum brown with yellow edges and setae; metanotum with brown sclerites with yellow margins. Pteropleura yellow, with broad brown areas on sclerites. ***Foreleg*.** Coxa yellow with base and apex brown suffused, trochanter ventrally brown, dorsally yellow. Femur yellow with brown band adjacent to closing area on posterior surface; anterior surface with area adjacent to closing surface on the basal ½ brown with yellow circular areas at setal bases. Tibia yellow with brown suffusions, with interspersed brown and yellow setae; basitarsus basal ½ yellow with brown areas, lanceolate process pale amber; tarsomeres 2–4 pale amber. ***Mid- and hind leg*.** Mid-leg with brown coxa, with setae of the same color, remaining podomeres yellow with setae of the same color, tarsomeres with the plantar surface with amber setae on the distal margin of the first four tarsomeres; pale amber pretarsal claws. Hind leg with brown coxa, yellow trochanter and femur; tibia brown with proximal 1/3 brown, remainder yellow, mostly pale brown setae, pale brown tibial spines; tarsus with the first three tarsomeres yellow, the rest pale amber, yellow and pale brown setae intermingled, plantar surface with pale amber setae distally in the first four tarsomeres; pale amber pretarsal claws. ***Wings*.** Forewing hyaline, area surrounding crossveins, base of RP, M fork, first branch of CuA, and apex of CuP dark amber; pterostigma pale brown with yellow area at middle; longitudinal veins alternating yellow and dark brown, subcostal veinlets and crossveins dark brown; wing margin mainly pale brown. Hind wing hyaline, with area around RP base, 1ra-rp, apical area beyond pterostigma, and apical branches of CuP dark amber; pterostigma pale brown, with preapical yellow spot; longitudinal veins mainly brown, with pale and dark areas; base of Cu and anal veins mainly yellow; wing margin alternating yellow and pale brown, crossveins mostly dark brown, subcostal veinlets pale brown. ***Abdomen*.** Tergites yellow with brown, posteromedial, triangular spot. Sternites II and III mainly brown, sternites IV–VII yellow with brown infuscations. Abdominal apex mainly brown. Pleural membrane brown.

***Morphology*** (Fig. 109). ***Head*.** Diamond-shaped in frontal view, vertexal region domed above compound eyes, laterally with rows of short, reclined setae; paraocular area concave; coronal suture not discernible. Antenna moniliform, scape ~ 2× as long as wide, slightly distally expanded; pedicel slightly longer than wide; flagellum slightly dorsoventrally compressed, with 34 flagellomeres, 2× as wide as long, narrower towards apex; all flagellomeres with medial ring of thickened setae. Compound eye hemispheric, as wide as ¾ of interocular distance at torulus level. Frons pentagonal narrow; clypeus trapezoidal, narrow; labrum rounded. Postgena broad, hypostomal bridge not completely fused with it. Maxillary palpus with first two short, third and fifth 3× as long as wide, the fourth 2× as long as wide. Labial palpus with third palpomere expanded and rounded, palpimacula broadly ovoid. ***Thorax*.** Pronotum slightly longer than wide, with groove contiguous to lateral and distal margins; anterior margin, medial area, and posterior margin, slightly elevated with long, pedicellate, thickened setae; episternum with long, thin setae; postfurcasternum trapezoidal. Mesonotum slightly wider than long, with abundant long, thickened setae on medial area. Metanotum ~ 2× as wide as long, glabrous. Pteropleura covered with abundant long, thin setae. ***Foreleg*.** Coxa as long as femur, cylindrical, with abundant fine, long setae; trochanter subconical, dorsally set with long and thick, pedicellate setae; femur robust, with abundant fine and long setae. Closing surface with both rows of integumentary specializations fully developed, with tubercle-shaped processes; posteroventral row proximally with a more developed process (process/seta ratio 1:1), anteroventral row with a more developed sub-basal process (process/seta ratio 1:1); thickened setae with globular base forming two rows adjacent to rows of processes, extending almost over the entire length of closing surface. Tibia almost as long as femur, curved, setose, closing surface keeled with row of prostrate setae; anterior surface with clavate setae at apex. Basitarsus elongated, ventrally keeled, with row of prostrate setae on proximal ½, anterior surface with clavate setae; lanceolate process, reaching the base of fourth tarsomere, with plug-shaped Stitz organ at apex; second tarsomere 4× as long as wide, third tarsomere as long as wide, last tarsomere slightly shorter than second. ***Mid- and hind leg*.** Mid-leg covered with abundant long, fine setae, slightly thickened tibia; basitarsus 4× as long as wide, second 1.5× as long as wide, third and fourth as long as wide, fifth tarsomere ~ 2× as long as wide; first four tarsomeres with a pair of thickened setae, laterally on the distal margin of plantar surface. Hind leg longer than mid-leg, densely covered with long, thin setae, shorter on tarsus; tibia slightly thickened, tarsomeres similar to those of the mid-leg. ***Wings*.** Forewing oval, trichomes present along wing margin except at base of posterior margin, venation setose; costal space narrow, humeral vein forked, 12 or 13 subcostal veinlets present; pterostigma rectangular, smoothly curved, with numerous incomplete veinlets; subcostal space with single medially located crossvein, Sc vein abruptly bent posteriad at proximal margin of pterostigma to merge the RA; *rarp2* gently curved, 1–3 veins arising from it, three from *rarp1*; M basally fused to R; RP base located near separation of M and R, M fork near such separation; 1r-m located between RP base and M fork forming a small trapezoidal cell; four gradate crossveins present. M vein deeply forked, CuA forked opposite the stem of RP; CuP proximally angled, almost touching A1, distally forked at level of divergence of M and R. 1A simple, ending at posterior margin, almost at level of separation of M and R; 2A distally forked, slightly beyond the level of CuP angle. Hind wing oval, smaller and narrower than forewing; costal space narrow and reduced, with three or four veinlets; C and Sc fused at proximal ¼ of wing length, Sc vein abruptly curved posteriad at proximal margin of pterostigma to merge the RA; pterostigma elongated, narrow, with numerous incomplete veinlets; radial space with single crossvein, oblique; two or three veins arising from *rarp1*, one or none from *rarp2*. Two gradate crossveins present. 1r-m sigmoid, connecting M base and RP base, distally connected again to M stem through short crossvein. M forked beyond R fork. Cu deeply forked, CuA long, sinuous, distally forked, first branch forked; CuP distally slightly anteriorly curved, pectinate. Cubitoanal space with two crossveins. A1 arched, A2 short and simple. ***Abdomen*.** Cylindrical, slightly distally expanded, without processes or pores.

**Figure 110.**
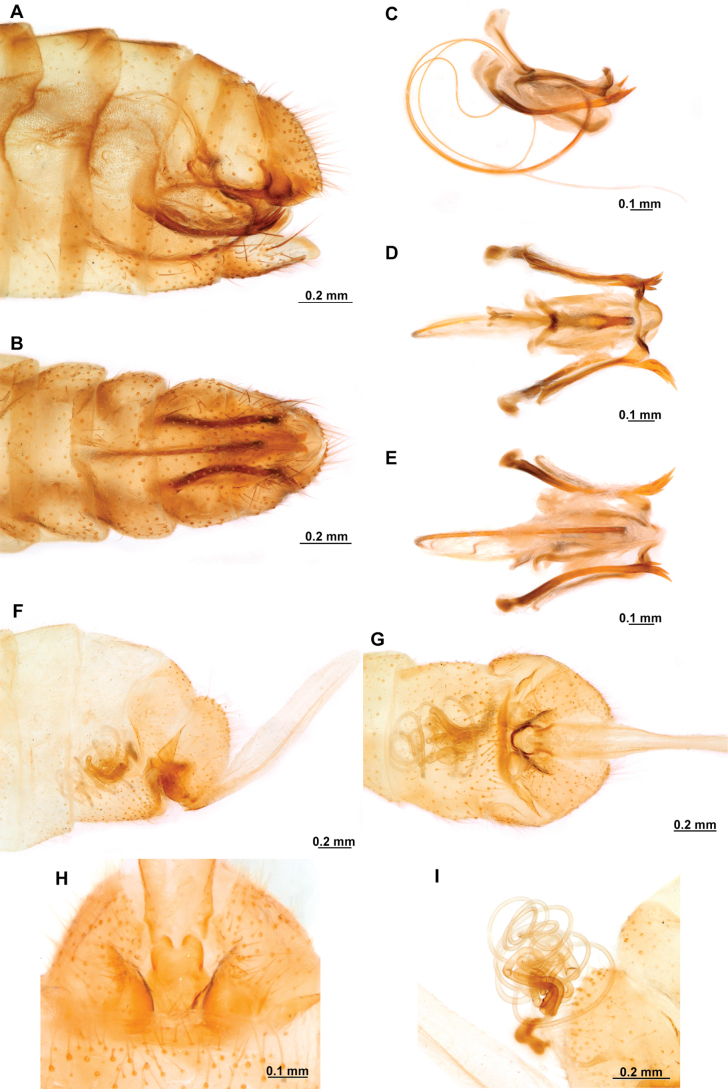
*Trichosceliatobari* (Navás, 1914) **A** male terminalia, lateral **B** same, ventral **C** male genitalia, lateral **D** same, dorsal **E** same, ventral **F** female terminalia, lateral **G** same, ventral **H** gonapophyses VIII ventral **I** spermatheca.

***Male genitalia*** (Fig. 110A–E). Tergite IX medially narrower than laterally, lateral margin rounded, with a posterior notch. Sternum VIII quadrangular. Sternite IX approximately pentagonal; the posteromedially glabrous, dorsally canaliculated; straight in lateral view, reaching posterior margin of ectoproct; remaining surface with abundant long and thin setae. Gonocoxite IX short, curved with spatulate base; apex laterodorsally curved, with four digitiform processes, two apical, medially situated, and two preapical, laterally located. Ectoproct approximately triangular, setose; ventrally with anterior slightly convex area, followed by concave area posteriorly; both covered with microtrichia. Gonocoxites X forming a straight, ventrally canaliculate sclerite, anterior apex expanded and dorsally curved; posterior apex spatulated, slightly bent, preapically with lateral projection on each side. Base of gonostyli X thickened and curved, with two lateral processes; the rest of the structure long, abruptly anteroventrally curved and anteriorly coiled, forming two internal loops before protruding from abdomen. Gonapophyses X straight, narrow, anterior apex curved, posterior apex dorsally bent with surrounding membrane set with minute spinules; gonapophyses arranged in a V-shaped structure, joined by a membrane forming a covering on base of gonostyli X. Gonocoxites XI approximately U-shaped, medial lobe enlarged and elaborated, consisting of two differentiated parts: dorsal part as broad and rounded lobe; ventral part as broad, convex region whose ventral margin is U-shaped in ventral view; the surface is covered with minute spinules; area between these parts less sclerotized. Gonocoxites XI arms straight to gently curved, anterior apex curved.

***Female genitalia*** (Fig. 110F–I). Sternite VII subtrapezoidal. Tergite VIII medially posteriorly incised, narrower than laterally, laterally widened, enclosing the spiracle of eighth abdominal segment, lateral margin triangular. Membrane between tergite VIII and tergite IX+ ectoproct sclerotized, forming a concave triangular plate. Gonocoxites VIII forming a trapezoidal, concave plate; gonapophyses VIII with medial part convex, keel-shaped, posteromedially with a short, thickened M-shaped process; lateral part as a triangular, convex plate. Tergite IX + ectoproct subrectangular. Gonocoxite IX long and narrow, as long as the last four abdominal segments together. Bursa copulatrix proximally funnel-shaped, sclerotized, concave, the rest membranous, long, and thin; spermatheca complex and entangled; proximal section long, thin, forming five coils, ending in an innermost spiral; medial section slightly thicker than proximal section, entangled, forming multiple convolutions. Distal section thin, progressively broader towards apex, forming two coils, distally with trumpet-shaped invagination. Fertilization canal duct short, thin, sigmoid; fertilization canal short, thickened, J-shaped, covered with microfilaments.

##### Distribution.

Mexico (Jalisco, Oaxaca, Quintana Roo, Tabasco, Veracruz).

##### Remarks.

Prior to this work *T.tobari* was known only from the original description. The type of this species was collected in the Mexican state of Tabasco and has never been reported from any other locality. Herein, the distribution of this species in Mexico is extended to the states of Jalisco, Oaxaca, Quintana Roo, and Veracruz.

*Trichosceliatobari* is easily distinguished by the pale antennal tips and the mark on the posterior surface of the forefemur. The morphology of the genital sclerites of this species suggest it is closely related to *T.flavomaculata*, *T.umbrata*, *T.basella*, *T.fenella* and *T.involuta* among others, which have a similar configuration of the male gonocoxites IX.

#### ﻿﻿ Trichoscelia umbrata

Taxon classificationAnimaliaNeuropteraRhachiberothidae

Ardila-Camacho, 2018

Figs 111 , 112

Trichoscelia umbrata Ardila-Camacho, 2018: 299. Holotype: male, Colombia, Caquetá (UNAB).

##### Material examined.

***Holotype*.** Colombia • ♂; **Caquetá**, Florencia, Macagual; 1°30'N, 75°39'W; 300 m; 13 Oct. 2014; C. García leg.; trampa de luz; UNAB.

##### Diagnosis.

This species is separated from *T.anae* because the subcostal space is narrow, and the antennal flagellum is completely dark in *T.umbrata*. Moreover, this species is distinguished from all other species because the forewing has the basal region of the costal and subcostal spaces dark amber, the latter also dark pigmented on the hind wing. Furthermore, on both wings, the area adjacent to posterior and apical margins presents intermittent dark infuscations ranging from cubital space to wing apex. The male genitalia are similar to those of *T.tobari* due to short gonocoxites IX equipped with four apical processes, and the similar coiling pattern of the gonostyli X. However, the overall shape of the gonocoxites XI, and particularly of the median lobe separate both species.

##### Description.

***Measurements*.** Male (*n* = 1). Forewing length: 7.5 mm; Hind wing length: 5.6 mm.

**Figure 111.**
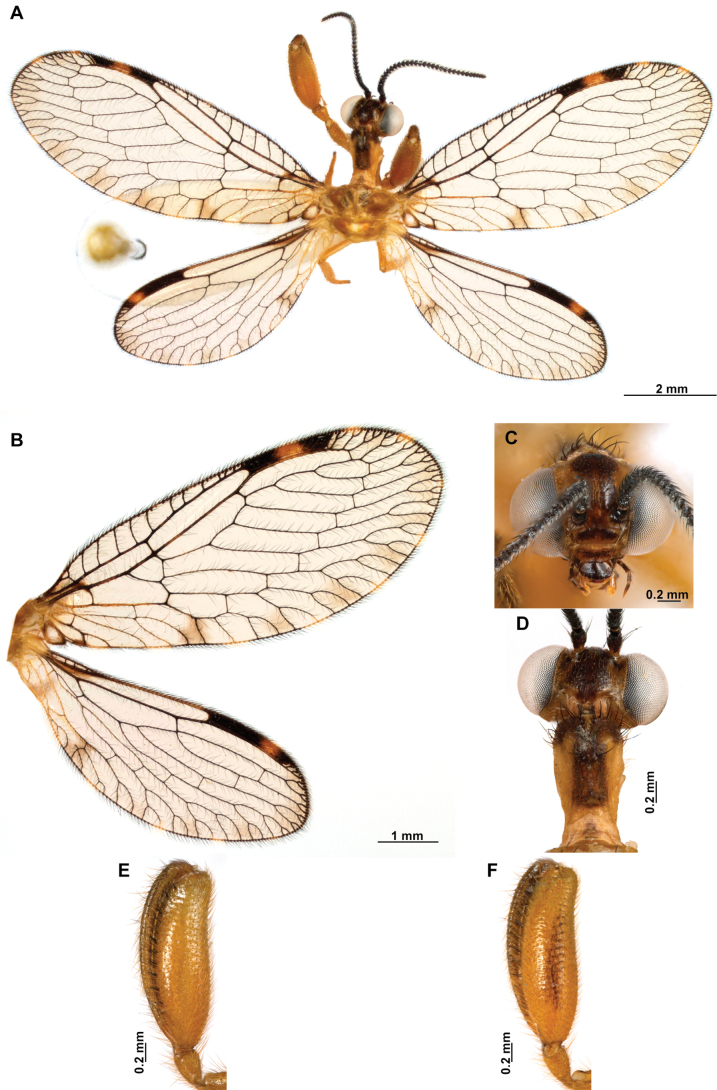
*Trichosceliaumbrata* Ardila-Camacho, 2018 **A** male habitus, dorsal (abdomen removed) **B** wings **C** head, frontal **D** pronotum, dorsal **E** forefemur, anterior surface **F** same, posterior surface.

***Coloration* (Fig. 111)**. ***Head*.** Mainly yellow, vertexal region with longitudinal brown band extending from occipital ridge to supraantennal area where it forks, covered with dark brown setae. Antennal scape dark brown, paler at base; pedicel dark brown; flagellum dark brown; frons brown with yellow margins; clypeus brown with yellow margins, labrum brown. Mandible brown, with pale amber apex; maxilla yellow, with dark brown palpus; labium yellow, palpus dark brown, palpimacula pale brown. ***Thorax*.** Pronotum yellow with brown broad, medial, longitudinal band and anterior margin; episternum yellow with dark brown suffusions and yellow setae; postfurcasternum yellow. Mesonotum yellow, with four dark brown markings on central area of scutum; scutellum yellow; metanotum pale yellow. Pre-episternum yellow. Pteropleura yellow. ***Foreleg*.** Coxa and trochanter yellow. Femur yellow with brown, longitudinal band on center of posterior surface forming a honeycomb pattern in which setal bases are yellow. Tibia brown, except on anterior surface and apex of posterior surface yellow, clavate setae yellow. Basitarsus yellow with blackish brown suffusions on proximal ½, pale amber in the rest; second to fourth tarsomere yellow with brown setae. ***Mid- and hind leg*.** Mid and hind leg yellow; first four tarsomeres with pale brown setae on plantar surface. ***Wings*.** Forewing hyaline, with smoky areas at base of costal and subcostal fields and on areas adjacent to terminal forks of Cu, M and RP, as well as on jugal lobe; pterostigma brown with yellow medial area; longitudinal veins mainly brown, except proximal ½ of CuA, areas of CuP, and anal veins yellow; crossveins and subcostal veinlets dark brown; posterior and distal margin alternating brown and yellow. Hind wing with yellow base, membrane hyaline with smoky areas at base of subcostal field and on membrane adjacent to terminal portion of CuP; pterostigma brown with small, yellow, preapical area; venation mostly brown, except pale brown C + Sc and proximal ½ of CuA and CuP and anal veins yellow; wing margin alternating yellow and dark brown, except at base, completely yellow. ***Abdomen*.** Abdominal segments 1-4 with yellow Tergites, with a brown area adjacent to the posterior margin; the remaining Tergites yellow; sternites yellow, except the eighth brown. The setae in Tergites and sternites corresponding to the color of the cuticle. Dark brown pleural membrane.

***Morphology* (Fig. 111)**. ***Head*.** Diamond-shaped in frontal view, moderately setose, smooth, vertexal region domed above compound eyes, with rows of reclined setae; paraocular area concave; coronal suture discrete. Compound eye hemispheric, as wide as ¾ of interocular distance at torulus level. Postgena broad, hypostomal bridge not completely fused with it. Antenna moniliform, scape ~ 2× as long as wide, slightly distally expanded; pedicel slightly longer than wide; flagellum slightly dorsoventrally compressed, with 34 flagellomeres, those of basal ½ 1.5× as wide as long, the rest almost as wide as long, progressively smaller towards apex; all flagellomeres with medial row of thickened setae. Maxillary palpus with first two palpomeres slightly longer than wide, third palpomere 3× as long as wide, fourth ~ 2× as long as wide, fifth palpomere slightly shorter than third. Submentum oval with long setae; labial palpus with first palpomere 1.5× as long as wide, second 3× as long as wide, third as long as the second, widely expanded, palpimacula broadly ovoid. ***Thorax*.** Pronotum 1.5× as long as wide, with furrow contiguous to lateral and anterior margins; in lateral view anterior margin, medial region and posterior margin slightly elevated, with thickened, pedicellate setae; episternum with long fine setae, postfurcasternum trapezoidal. Mesonotum slightly wider than long, with thick, pedicellate setae on medial area. Metanotum ~ 2× as wide as long, with two lateral cusps and a medial depression, mostly glabrous. Pteropleura covered with abundant long, thin setae. ***Foreleg*.** Coxa slightly shorter than femur, cylindrical, with abundant long and thin setae; trochanter subconical, with thickened, pedicellate setae near distal margin on dorsal surface; femur robust, with abundant short, thin setae; closing surface with double row of tubercle-shaped specializations fully developed, both with a more developed sub-basal process (process/setae ratio 2:1); rows of thickened, pedicellate setae with globular base adjacent to each row of processes present, extending over almost the entire closure surface. Tibia almost as long as femur, curved, with short and thin setae, closing surface with prostrate setae; with a patch of clavate setae apically on inner surface; basitarsus elongated, ventrally keeled and with a row of prostrate setae; anterior surface with a patch of clavate setae proximally, lanceolate process reaching the base of fourth tarsomere, with a plug-shaped Stitz organ at apex; second tarsomere 4× as long as wide, third tarsomere as long as wide, fourth tarsomere 2× as long as wide. ***Mid- and hind leg*.** Mid-leg with coxa and trochanter covered with long, thin setae; tibia and femur with short, thin setae; basitarsus 3× as long as wide, second tarsomere slightly longer than wide, third and fourth as long as broad, fifth tarsomere 2× as long as wide, all covered with short, thin setae; the first four tarsomeres with a pair of thickened setae laterally on distal margin of plantar surface. Hind leg longer than mid-leg, with short and thin setae; tibia 1.5× as long as femur; tarsomeres similar to those of the middle leg. ***Wings*.** Forewing oval, trichosors present along wing margin except at base, venation setose; costal space narrow, humeral vein forked, 11 subcostal veinlets; pterostigma elongated and narrow, composed of numerous, mainly incomplete veinlets; subcostal space with single medially located crossvein; Sc vein bent posteriad at proximal margin of pterostigma to merge the RA; *rarp2* curved, with two veins arising from it, three from *rarp1*; M fused basally to R; RP base located near separation of M and R, M fork near such separation, opposite to R fork; 1r-m located between RP base and MA base, forming a small trapezoidal cell; five gradate crossveins present. Cu vein deeply forked; CuP basally angled, approaching A1, distally forked, opposite to separation of M and R. 1A simple, reaching the level of separation of M and R; A1 distally forked, at the level of CuP angle. Hind wing smaller and narrower than forewing; costal space narrow and reduced, with three or four veinlets; Sc and C fused at 1/5 of wing length, Sc curved posteriad at proximal margin pterostigma to merge the RA; pterostigma elongated, narrow, curved, composed of numerous incomplete veinlets; radial space with single crossvein, oblique; two or three veins arising from *rarp1*, one or none from *rarp2*. M vein forked beyond R fork; 1r-m sigmoid, connecting M base and RP base, distally connected again to M stem through short crossvein. Cu deeply forked, CuA long and sinuous, distally forked, first branch candelabrum-shaped; CuP distally anteriorly curved, pectinate. Cubitoanal space with two crossveins. A1 arched, A2 short and arched. ***Abdomen*.** Cylindrical, tergites quadrangular; intertergal membranes between segments III–VI broad, covered with microtrichia, and with two glabrous lateral markings; sternites rectangular, except those of segments II and III.

**Figure 112.**
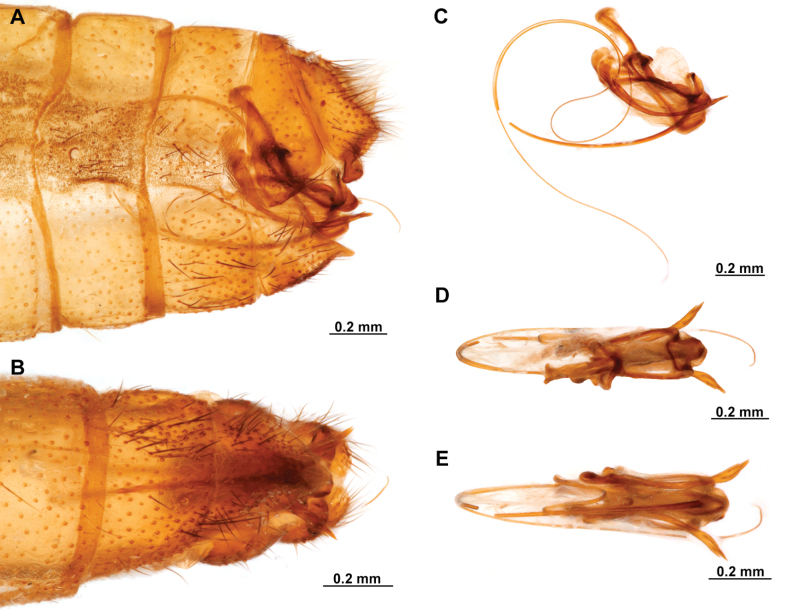
*Trichosceliaumbrata* Ardila-Camacho, 2018 **A** male terminalia, lateral **B** same, ventral **C** male genitalia, lateral **D** same, dorsal **E** same, ventral.

***Male genitalia*** (Fig. 112). Tergite IX, notably narrower medially than laterally, lateral margin quadrangular. Sternite VIII rectangular; sternite IX pentagonal, posteromedially forming an acute angle, glabrous, dorsally canaliculated, the rest of the surface with abundant long and thin setae; in lateral view acuminate, reaching the posterior margin of the ectoproct. Gonocoxite IX short, thickened and arched, base spatulate; apex laterally curved, with four digitiform processes, two apical subequal, two longer lateral ones. Ectoproct semi-triangular, setose; ventral surface with a well-developed and sclerotized quadrangular anterior lobe, set with microspinules; posterior ½ with well sclerotized concave area. Gonocoxites X forming a short, straight, ventrally canaliculate sclerite; anterior apex expanded, posterior apex bilobed, with two additional, lateral preapical processes. Gonostyli X with thickened, bent base, with two lateral processes; the rest of the structure, long, ventrally curved and anteriorly coiled, forming two internal loops before protruding from the abdomen. Gonapophyses X straight, thin, posterior apex dorsally bent with surrounding membrane devoid of spinules; gonapophyses subparallel, joined by membrane covering base of gonostyli X. Gonocoxites XI U-shaped, medial lobe expanded and elaborate, with two differentiated parts; dorsal part as a concave, anterodorsally projected, quadrangular lobe; ventral part as a convex area set with microspinules, with ventral edge deeply incised, U-shaped; between these parts a narrow, less sclerotized region is present. Lateral arms of gonocoxites XI arched.

##### Distribution.

Colombia (Caquetá).

##### Remarks.

This species is known solely from its original description (Ardila-Camacho et al. 2018). The holotype was collected in the Colombian Amazonia, and no additional records have been reported since then. *Trichosceliaumbrata* is distinguished by its intermittent shaded areas on the posterior margin of the wings, remembering *Trichosceliaanae* and species of the genus *Plega.* However, the genitalic features of this species allies it with *T.basella*, *T.fenella*, and *T.involuta*.

#### ﻿﻿ Trichoscelia varia

Taxon classificationAnimaliaNeuropteraRhachiberothidae

(Walker, 1853)

Figs 113 , 114

Raphidia varia Walker, 1853: 212. Holotype: sex unknown, Brazil (NHMUK), specimen examined.
Trichoscelis varia (Walker, 1853). Hagen (1861): 323.
Trichoscelia varia (Walker, 1853). McLachlan (1867): 261.
Symphrasis varia (Walker, 1853). Enderlein (1910): 374.
Mantispa myrapetrella Westwood, 1867: 505. Lectotype: male, “in nidis vespae (Myrapetraescutellaris) Americae meridionalis” (OUMNH). Designated by Penny (1982a): 436, specimen examined.
Symphrasis myrapetrella (Westwood, 1867). Hagen (1877): 2010.

##### Material examined.

***Holotype*** of *Raphidiavaria*.

Brazil • sex unknown; Río Janeay [**Rio de Janeiro**?]; 1837 or 1838; Holotype sex? *Raphidiavaria*Walker 1853, Genotype of *Symphrasis* Hagen, remounted 16 Jul. 1968, Robert Beard; ex nest of *Myrapetrascutellaris* White 1841; From nest of *Myrapetrascutellaris* 5.13_8; abdomen missing; *varia*; green holotype label; right wings glued on an old card; NHMUK 013802773; NHMUK.

***Lectotype*** of *Mantispamyrapetrella*.

Unknown locality • ♂; *Mantispamyrapetrella*Westwood 1867, desig. By R.G. Beard 1968; Type Neur: No. 16 *Mantispamyrapetrella* Westwood 1/8 HOPE DEPT. OXFORD; *Trichosceliavaria* (Walker, 1853) ♂, det R.G. Beard; “in nidis vespae (*Myrapetrascutellaris*) Americae meridionalis”; genitalia cleared and stored in a microvial; OUMNH.

***Paralectotypes*** of *Mantispamyrapetrella*.

Unknown locality • 1 ♂; *Mantispamyrapetrella*Westwood 1867, desig. By R.G. Beard 1968; Type Neur: No. 16 *Mantispamyrapetrella* Westwood 3/8 HOPE DEPT. OXFORD; genitalia cleared and stored in a microvial; OUMNH. • 1 ♀, *Mantispamyrapetrella*Westwood 1867, desig. By R.G. Beard 1968; Type Neur: No 16 *Mantispamyrapetrella* Westwood, 1/8, HOPE DEPT. OXFORD; OUMNH.

##### Other material.

Unknown locality • 1 ♀; 1909; G.A. Poujade; *Symphrasisvaria* Walker female det. D.E. Kimmins; This locality label appears to be incorrect, D.E. Kimmins; Museum Paris, Fontainebleau, coll. G.A. Poujade, 1909, 1930-130; NHMUK 013802788; NHMUK.

Argentina – **Buenos Aires** • 1 ♂; La Plata; 03 Dec. 1916; *Symphrasisvaria* Walk. Det. S.J. Navás; FSCA. • 1 ♂; La Plata; 03 Dec. 1915; *Symphrasisvaria* det. P. Navás S.J.; MCZ-ENT 00681633; MCZ. • 1 ♀; Pereyra; 05 Jan. 1952; *Trichosceliavaria* det. N. Penny, 1981; USNMENT01541920; USNM. • 1 ♀; Delta del Río Sarmiento; Bachmann leg.; lar 45, in nest of *Polybia*; *Trichosceliavaria* det. N. Penny, 1981; USNMENT01541926; USNM. • 2 ♂ 2 ♀; La Plata; 03 Dec. 1916; R.G. Beard leg.; *Symphrasisvaria* Walker det by P. Navás, excollection Mus. Barcelona; MCZ. – **Catamarca** • 4 ♀; 3 Km N. Belén; 05 Dec. 1970; C. Porter & L. Stange leg.; FSCA. • 1 ♀; 6 Km S. de Belén; 25 Nov. 1975; L. Stange leg.; FSCA. • 1 ♂; same data as for preceding; *Trichosceliavaria* (Walker) det. L. Stange, 1975; TAMU-ENTO X0405468; TAMU. – [**Córdoba**] • 1 ♂; Alta Gracia; 22 Feb. 1926; C. Bruch leg.; *Symphrasisvaria* Walk., ex nido de *Polybiascutellaris*, Olivos, 20 Nov. 1926, det. Navás; FSCA. • 1 ♀; Córdoba, Punilla, Tanti; 950 m; Feb.; M. Vizmark; *Trichosceliavaria* det. N. Penny; USNMENT01541921; USNM. – **La Rioja** • 1 ♂ 1 ♀; Belén; 19 Dec. 1971; Stange & Porter leg.; FSCA. • 1 ♀; Santa Cruz; 1700 m. a.s.l.; 06 Feb–05 Mar. 2002; P. Fidalgo leg.; trampa Malaise; FSCA. • 1 ♂; Anillaco; 05 Jul. 2006; Fidalgo leg.; FSCA. – **Salta** • 1 ♂; Yacochuya, (Cafayate); 1959 m; 1–15 Jan. 1969; Willink, Terán, Stange leg.; Malaise; FSCA. • 1 ♀; same data as for preceding; 1950 m; 1–15 Oct. 1968; FSCA. • 1 ♀; same data as for preceding; 1950 m; 16–31 Oct. 1968; FSCA. – **Santa Fé** • 1 ♂; 01 Feb. 1927; Bridaroli S. leg.; *Symphrasisvaria* det. G. Williner; USNMENT01541922; USNM. – **Tucumán** • 1 ♂; Horco Molle; 25 Nov. 1970; L. Stange leg.; FSCA. • 1 ♀; Horco Molle; 18 Feb. 1973; L. Stange leg.; FSCA. • 1 ♂; Las Cejas, La Soledad; Nov. 1970; L. Stange leg.; FSCA. • 1 ♀; San Pedro de Colalao; Nov. 1969; L. Stange leg.; FSCA.

Bolivia • 1 ♀; **Santa Cruz**, 4–5 Km N. Achira, Rd to Amboro; 22 Oct. 2000; M.C. Thomas leg.; FSCA. • 1 ♀; El Refugio, Los Volcanes; 18°06'S, 63°36'W; 1800 feet; 18 Sep. 2012; P. Skelley & J. Wappes leg.; UV light; FSCA.

Brazil – Unknown locality • 1 ♂; 02 Jul. 1968; Paul H.V. Iberig leg.; det. as *Anisopteranotha* by L. Navás 1907 Econge MNHN; det. N. Banks; MCZ. • 1 ♂; 1899; Paul H.V. Iberig leg.; MCZ. – **Bahía** • 1 ♂ 1 ♀; 13 Nov. 2012; D.M. Takya & A.P.M. Santos leg.; light trap; DZUP. – **Ceará** • 1 ♀; Cascavel; Nov. 1939; D.C. Blues leg.; *Trichosceliavaria* det N. Penny, 1981; USNMENT01541923; USNM. • 1 ♀; same data as for preceding; USNMENT01541924; USNM. – **Esp**í**rito Santo** • 1 ♀; Linhares, Parque Soretama; Oct. 1962; M. Alvarenga leg.; FSCA. – **Mato Grosso** • 1 ♀; 12°50'S, 51°47'W; 14 Oct. 1968; O.W. Richards; Campo, R.S. & R.G.S. Expedition B.M. leg.; “Cocoon in cells of *Polybiaruficeps* Schrottky”; 1968-260, NHMUK 013802787; NHMUK. • 1 ♀; same data as for preceding; R.S. & R.G.S. Expedition B.M. 1968-260 leg.; “Cocoon in cells of *Polybiaruficeps* Schrottky, female emergence 17.X”; NHMUK 013802791; NHMUK. • 1 ♂; Corumba; 14–28 Dec. 1919; Cornell Univ. Expedition leg.; Lot 569 Sub 141; MCZ-ENT 00681793; MCZ. • 1 ♂ 1 ♀ 1 ?; Corumba; 17 Dec. 1919; bred from nest of *Protopolybiasedula*, Col. From Limestone cliff. by J. Chester Bradely and R. Gordon Harris, Lot 569 sub. Lot 141; Cornell Univ. Expedition lot. 569 sub 141; MCZ. – **Minas Gerais** • 1 ♂; Nova Lima; 850 m; 8–9 Oct. 1985; S.E. Miller leg.; USNMENT01541930; USNM. • 1 ♂; S. Gonçalo Rio Abaixo, Est. Amb. Peti-Cemig; 19°53'02"S, 43°22'21"W; 07–14 May. 2012; A. Lima, A. Kumagai & P. Dias leg.; Pennsylvania; DZUP. • 1 ♀; same data as for preceding; 12–19 Sep. 2012; luminosa; DZUP. • 1 ♀; Pq. N. Serra dos Cipós, Trilha Carijó, KM 15: 13 Dec. 2011; D.M. Takya & A.P.M. Santos; light trap; DZUP. • 1 ♀; Serra do Salitre, RPPN Cachoeira do Campo; 19°09'46"S, 46°33'60"W; 14 Oct. 2012; Lima & Kumagai leg.; black light trap; UFMG. • 1 ♂; S. Gonçalo Rio Abaixo, Est. Amb. Peti-Cemig; 19°53'02"S, 43°22'21"W; 14–21 Aug. 2012; Lima & Kumagai leg.; light trap; UFMG. • 1 ♂; Belo Horizonte, UFMG; 21 Oct. 2013; AFL leg.; UFMG. – **Rio de Janeiro** • 1 ♀; Rio Macacu, 650 m, (N. of Cach. de Macacu); 16 Oct. 1985; S.E. Miller leg.; USNMENT01541925; USNM. • 1 ♀; Petropolis; 650 m; 20 Oct. 1985; S.E. Miller leg.; USNMENT01541929; USNM. • 1 ♀; Itatiaia, PN Itatiaia, alojamento 2; 22°27'08"S, 44°36'28"W; 25–28 Oct. 2019; A.C. Domahovski leg.; light trap; DZUP. • 1 ♂; Itatiaia, PN Itatiaia; 22°27'14.4"S, 44°36'28.8"W; 29 Oct. 2011; G. Melo leg.; light trap; DZUP. • 1 ♀; same data as for preceding; 22°27'16"S, 44°36'30"W; 29 Dec. 2018–02 Jan. 2019; A.P.M. Santos & D.M. Takiya leg.; light trap; DZUP. – **São Paulo**​ • 1 ♀; Salesópolis, Est. Biol. Boraceia; 850 m; 20 Nov. 1963; Rabello & Medeiros leg.; light, 20h; *Trichosceliazikani* Navás, det. L. Stange, 1991; FSCA. • 1 ♀; Etat de Saint Paul; 1899; H. Von Jhering; det. as *Symphrasisvaria*; FSCA. • 2 ♀; Teodoro Sampaio, Pq. Est. Morro do Diabo; 22°36'17"S, 52°18'06"W; 16 Nov. 2010; R. Lara and team leg.; malaise; DZUP. • 1 ♂; Luiz Antonio, Estação Ecológica Jataí; 21°36'47"S, 47°49'04"W; 19 Dec. 2007; R. Lara and team leg.; light trap; DZUP. • 1 ♀; same data as for preceding; 16 Jan. 2008; DZUP. • 1 ♂ 2 ♀; same data as for preceding; 30 Jan. 2008; DZUP. • 2 ♀; same data as for preceding; 13 Feb. 2008; DZUP. • 2 ♀; same data as for preceding; 12 Mar. 2008; DZUP. • 1 ♀; same data as for preceding; 01 Oct. 2008; DZUP. • 1 ♂; Etat de Saint Paul; 1808; H. Von Jhering; MCZ.

Uruguay • 1 ♂; **Montevideo**; *Trichosceliavaria* (Walker 1853) male, det. R.G. Beard; *Myrapetrascutellaris* Montevideo; McLachlan Coll. B.M. 1938-674; *Trichosceliavaria* or *M.myrapetrella*; NHMUK 013802794; NHMUK. • 1 ♂; same data as for preceding; *Trichosceliavaria* (Walker 1853) male, det. R.G. Beard 1968; *Trichosceliavaria* or *M.scutellaris*; nest of *Myrapetrascutellaris*; McLachlan Coll. B.M. 1938-674; NHMUK 013802786; NHMUK. • 1 ♀; same data as for preceding; *Trichosceliavaria* (Walker, 1853) female, det. R.G. Beard 1968; *Symphrasisvaria* or *M.myrapetrella*; nest of *Myrapetrascutellaris*, Montevideo; McLachlan Coll. B.M. 1938-674; NHMUK 013802785; NHMUK. • 1 ♀; same data as for preceding; *Trichosceliavaria* (Walker, 1853) female, det. R.G. Beard 1968; *Trichosceliavaria* or *M.scutellaris*; ex nest of *Myrapetrascutellaris* Montevideo; McLachlan Coll. B.M. 1938-674; NHMUK 013802789; NHMUK. • 1 ♂; same data as for preceding; *Trichosceliavaria* (Walker 1853) male, det. R.G. Beard; *Trichosceliavaria* or *M.myrapetrella*; Esc. *Myrapetrascutellaris* Montevideo; McLachlan Coll. B.M. 1938-674; NHMUK 013802790; NHMUK. • 1 ♀; same data as for preceding; *Trichosceliavaria* (Walker, 1853) female, det. R.G. Beard 1968, *Trichosceliavaria* or *M.myrapetrella*; abdomen missing; Esc. *Myrapetrascutellaris* Montevideo; NHMUK 013802793; NHMUK. • pupal exhubia and cocoon; Montevideo; *Trichosceliavaria*; McLachlan Coll. B.M. 1938-674; NHMUK 013802792; NHMUK. • 8 ♂ 13 ♀; Depto. Prado; 1960; J. Baltar; in *Polybia* sp. nest; FSCA. • 1 ♀; Depto. Prado; 1960; J. Baltar; in *Polybia* sp. nest; det. L. Stange, 1991; CAS. • 1 ♀; Depto. Prado; 1960; J. Baltar; in *Polybia* sp. nest; TAMU-ENTO X0745159; TAMU. • 1 ♂ 1 ♀ 1 pupa; Montevideo; 1862; McLachlan leg.; “M. lacht gezogen amd Noflt on *Myrapetrascutellaris*, 1862”; MCZ.

##### Diagnosis.

This species is separated from others in the genus because of its short antennae with flagellomeres approximately as wide as long. The pronotum is approximately as long as wide, and the forefemur is remarkably narrower than in other species of the genus. The wing maculation is composed of amber marks on the areas adjacent to crossveins, and forks of longitudinal veins which vary from faint to well-marked. Strongly marked specimens have extensive markings on wing apexes. On the male genitalia, the gonocoxite IX is long, thin and almost straight; the posterior apex is curved, with two to four digitiform processes, of which, when present two are preapical, dorsally situated and widely separated. On the female genitalia, the gonapophyses VIII medial part is keel-shaped, with posteromedial bilobed process, whose lobes are slightly elongated; the lateral part is an oval, ventrally convex plate.

##### Description.

***Measurements*.** Male (*n* = 4). Forewing length: 7.9–8.7 mm; Hind wing length: 5.4–6.7 mm. Female (m=6). Forewing length: 8.4–9.6 mm; Hind wing length: 6.5–7.7 mm.

**Figure 113.**
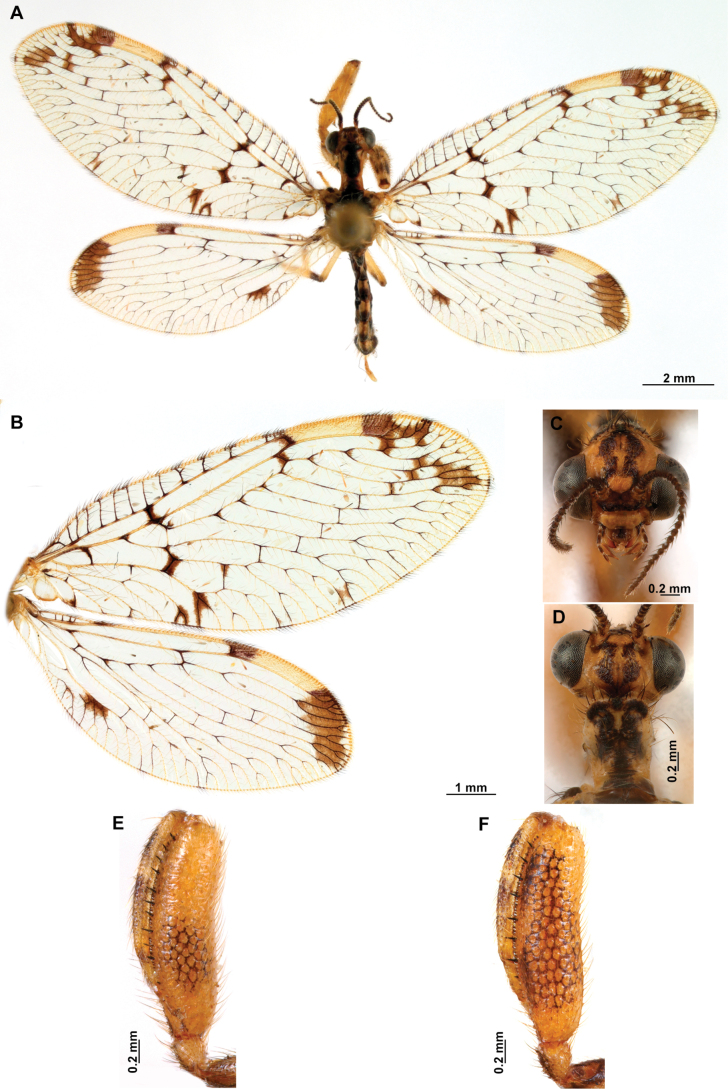
*Trichosceliavaria* (Walker, 1853) **A** male habitus, dorsal (abdomen removed) **B** wings **C** head, frontal **D** pronotum, dorsal **E** forefemur, anterior surface **F** same, posterior surface.

***Coloration*** (Fig. 113). ***Head*.** Mainly yellow, vertexal region either with a pair of sinuous bands touching or fusing at supraantennal area or with single, broad band extending from the occipital ridge to supraantennal area where it forks; covered with yellow setae. Hypostomal bridge brown. Antennal scape yellow with dark brown laterodistal spot; pedicel brown; flagellum brown. Frons brown with yellow anterior margin; clypeus yellow, sometimes with brown suffusions; labrum yellow with brown marking at center; mandible yellow, with brown suffusions at base, apex pale amber. Maxilla yellow, with brown palpus; labium with pale brown setae on the submentum, palpus pale brown, palpimacula yellow. ***Thorax*.** Pronotum yellow with longitudinal, medial, broad brown band, which may have yellow areas, anterior margin brown, lateral areas sometimes with brown mark; with concolorous setae; episternum brown, with central yellow area; postfurcasternum yellow. Mesonotum yellow, variegated with broad brown areas, scutellum yellow laterally and brown at center; metanotum with broad brown areas, sometimes with yellow regions laterally on scutum, medially pale yellow, scutellum with brown mark at center. Pre-episternum brown. Pteropleura with large brown areas and yellow regions adjacent to sutures. ***Foreleg*.** Coxa yellow with brown areas at base and apex, sometimes connected on dorsal and ventral surfaces; trochanter yellow with brown area on ventral surface. Femur yellow, with one or two brown areas on anterior and posterior surfaces, varying in extent and intensity, setal bases in these areas yellow. Tibia yellow with large brown areas, clavate setae yellow. Basitarsus basal ½ yellow with pale brown suffusions; lanceolate process pale amber, prostrate setae yellow; second to fourth tarsomere yellow to pale brown. ***Mid- and hind leg*.** Mid-leg with dark brown coxa, trochanter brown with yellow apex, femur yellow with brown ring at middle, tibia yellow with brown mark at base, tarsus yellow; plantar surface of the first four tarsomeres, with dark brown setae. Hind leg with dark brown coxa, yellow trochanter with brown mark at apex, femur yellow, tibia yellow with brown base, tarsus yellow changing to pale brown towards apex. ***Wings*.** Forewing hyaline, area adjacent to crossveins, separation of M and R, forks of M and R, base of RP branches, and apical branches of main veins amber; sometimes strong maculation on wing apex is present; pterostigma brown with yellow medial area; longitudinal veins alternating dark brown and yellow, crossveins and subcostal veinlets dark brown; wing margin alternating brown and yellow. Hind wing hyaline, with amber markings on area adjacent to stem of first branch of CuA, and on wing apex; pterostigma brown with large, yellow, preapical area; venation alternating yellow and dark brown; posterior and distal margin alternating yellow and brown. ***Abdomen*.** Tergites of abdominal segments I and II dark brown, with yellow margins; tergites III–VIII yellow with brown spot on anterior and posterior margins, which are connected by medial line in males; females with posterior 1/3 and medial line dark brown. Sternites yellow, with brown lateral markings, sternite VIII completely dark brown. Pleural membrane yellow with extensive brown areas at the center of each segment.

***Morphology*** (Fig. 113). ***Head*.** Diamond-shaped in frontal view, moderately setose, smooth, vertexal region domed above compound eyes, with rows of reclined setae; paraocular area concave; coronal suture discrete. Compound eye hemispheric, slightly wider than 1/2 of interocular distance at torulus level. Postgena broad, hypostomal bridge not completely fused with it. Antenna moniliform, scape ~ 2× as long as wide, sub-cylindrical; pedicel as long as wide; flagellum slightly dorsoventrally compressed, with 27–30 flagellomeres, slightly wider than long at base, the rest as wide as long, progressively smaller towards apex; all flagellomeres with medial ring of thickened setae. Maxillary palpus with the first two palpomeres as long as wide, third palpomere 3× as long as wide, the fourth ~ 2× as long as wide, fifth palpomere slightly shorter than third. Submentum oval with long setae, palpus with first palpomere slightly longer than wide, second 2× as long as wide, third slightly longer than second, widely expanded, palpimacula broadly ovoid. ***Thorax*.** Pronotum as long as wide, with furrow contiguous to lateral and anterior margins; in lateral view anterior margin, medial region and posterior margin slightly elevated, with thickened, pedicellate setae; episternum with long fine setae; postfurcasternum trapezoidal. Mesonotum 1.5× wider than long, with thick, pedicellate setae on medial area. Metanotum ~ 2× as long as wide, mostly glabrous. Pteropleura covered with abundant short and thin setae, longer in mesanepisternum. ***Foreleg*.** Coxa slightly shorter than femur, cylindrical, with abundant, thin setae; trochanter trapezoidal; femur robust with short and thin setae; closing surface with double row of tubercle-shaped specializations fully developed, both with a more developed sub-basal process (process/setae ratio 1:1); rows of thickened setae with globular base adjacent to each row of processes present, extending over almost the entire closing surface. Tibia almost as long as femur, curved, with short and thin setae, closing surface with prostrate setae; anterior surface with patch of clavate setae at apex; basitarsus elongated, ventrally keeled with a row of prostrate setae, anterior surface on proximal ½, with a patch of clavate setae; lanceolate process approaching distal margin of third tarsomere, with plug-shaped Stitz organ at apex; second tarsomere 4× as long as wide, third tarsomere as long as wide, fourth tarsomere 2× as long as wide. ***Mid- and hind leg*.** Mid-leg with thin setae; basitarsus 3× as long as wide, second tarsomere 2× as long as wide, third slightly longer than wide, fourth as long as wide, fifth tarsomere 2× as long as wide; the first four tarsomeres with a pair of thickened setae laterally on the distal margin of plantar surface. Hind leg longer than mid-leg, densely setose, tibia 1.5× as long as femur; tarsomeres similar to those of mid-leg. ***Wings*.** Forewing oval, trichosors present along wing margin except at base, venation setose; costal space slightly widened medially, humeral vein branched or forked, 14–16 subcostal veinlets, of which one or two are forked; pterostigma elongated and narrow, composed of numerous veinlets, mainly entire; subcostal space with single, medially located crossvein; Sc vein bent posteriad at proximal margin of pterostigma to merge the RA; *rarp2* curved, with two or three veins arising from it, three from *rarp1*; M fused basally to R; base of RP located near separation of M and R, M fork near such separation, opposite to R fork; 1r-m located between RP base and MA base, forming a small trapezoidal cell; 4–6 gradate crossveins present. Cu vein deeply forked; CuP basally angled, approaching A1, distally forked opposite to separation of M and R. A1 forked at apex, reaching the level of separation of M and R; A2 distally forked, slightly beyond the level CuP angle. Hind wing smaller and narrower than forewing; costal space narrow and reduced, with 5–7 veinlets; Sc and C fused at 1/5 of wing length, Sc curved posteriad at proximal margin of pterostigma to merge the RA; pterostigma elongated, narrow, curved, composed of numerous generally complete veinlets; radial space with single crossvein, oblique; three veins arising from *rarp1*, two from *rarp2*. Three gradate crossveins present. M vein forked beyond R fork; 1 r-m sigmoid, connecting M base and RP base, distally connected again to M stem through short crossvein. Cu deeply forked, CuA long, sinuous, distally branched, first branch candelabrum-shaped; CuP distally anteriorly curved, pectinate. Cubitoanal space with one or two crossveins. A1 arched, A2 short and arched. ***Abdomen*.** Cylindrical, tergites; intertergal membrane between segments III–V expanded; sternites rectangular, except in sternite II trapezoidal.

**Figure 114.**
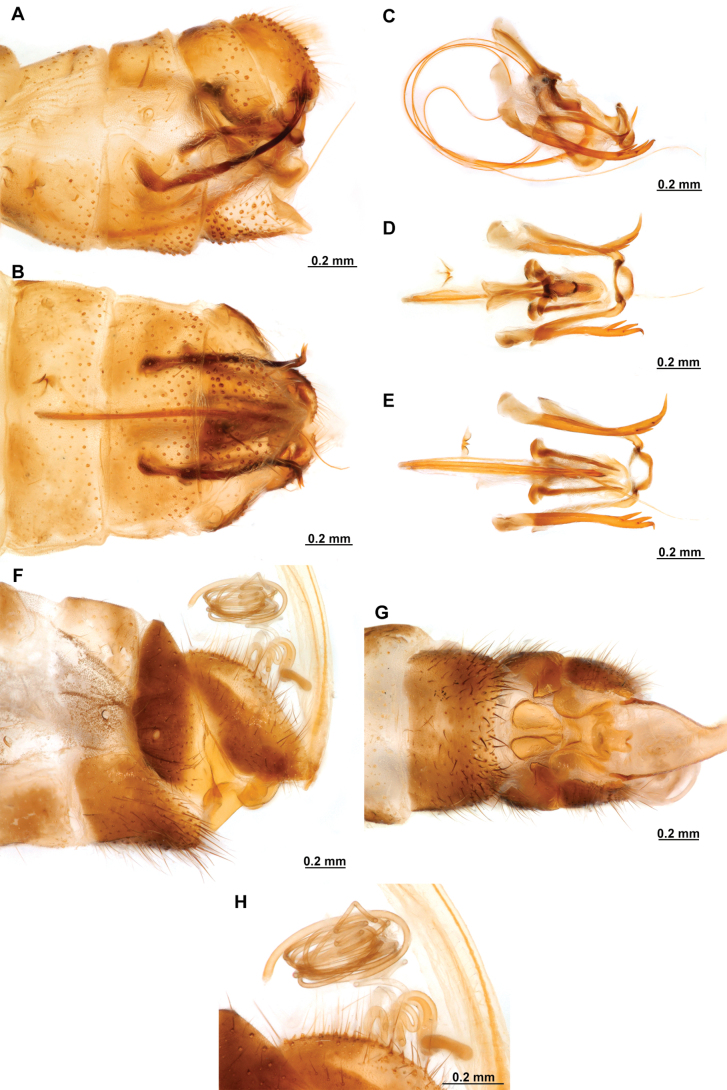
*Trichosceliavaria* (Walker, 1853) **A** male terminalia, lateral **B** same, ventral **C** male genitalia, lateral **D** same, dorsal **E** same, ventral **F** female terminalia, lateral **G** same, ventral **H** spermatheca.

***Male genitalia*** (Fig. 114A–E). Tergite IX medially slightly narrower than laterally, lateral margin quadrangular, with posterior notch. Sternite VIII rectangular; sternite IX pentagonal, posteromedially forming an acute angle, glabrous, dorsally canaliculate, the rest of the surface with abundant short, thin setae; in lateral view acuminate, reaching posterior margin of the ectoproct. Gonocoxite IX long, thin and almost straight, base spatulate, dorsally bent; apex curved, with three or four digitiform processes, of which one or two are apical and two are preapical, dorsally situated and widely separated. Ectoproct triangular, setose; ventral surface with anterior, well-sclerotized, convex, circular area covered with microtrichia, continued posteriorly by well sclerotized, curved sulcus. Gonocoxites X forming a short, ventrally canaliculate sclerite, anterior ½ expanded, posterior apex bilobed, with median concavity, and two short preapical processes. Gonostyli X with thickened, short, and curved base, with two lateral processes, the rest of the structure long, curved ventrally and coiled anteriorly, forming two internal loops before protruding from abdomen. Gonapophyses X straight, thin, posterior apex bent dorsally with surrounding membrane set with microspinules; gonapophyses arranged in a V-shaped structure, joined by a membrane covering the base of gonostyli X. Gonocoxites XI U-shaped, median lobe expanded and elaborated, with two differentiated parts: dorsal part as an anterodorsally projecting rounded lobe; ventral part a convex area covered with microspinules, with U-shaped ventral edge; between these parts a broad less sclerotized region is present. Lateral arms of gonocoxites XI curved, anterior apex lateroventrally curved.

***Female genitalia*** (Fig. 114F–H). Sternite VII trapezoidal. Tergite VIII narrower medially than laterally, enclosing the spiracle of the eighth abdominal segment, lateral margin fusiform. Membrane between tergite VIII and tergite IX + ectoproct sclerotized, forming a concave triangular plate. Gonocoxites VIII forming a rectangular plate, with two lateral concavities; gonapophyses VIII medial part keel-shaped, with posteromedial bilobed process, whose lobes are slightly elongated; lateral part an oval, ventrally convex plate. Tergite IX + ectoproct triangular. Gonocoxite IX long and narrow, as long as the last four abdominal segments together. Bursa copulatrix funnel-shaped, membranous, elongated, proximally sclerotized and concave; spermatheca complex and entangled; proximal section long, thin, forming six coils, ending in an innermost spiral; medial section as wide as proximal section, entangled, forming several convolutions. Distal section progressively broadened towards the apex, J-shaped, distally with a trumpet-shaped invagination. Fertilization canal duct short, thin, sigmoid; fertilization canal short, thickened, J-shaped, covered with microfilaments.

##### Distribution.

Argentina (Buenos Aires, Catamarca, Córdoba, La Rioja, Salta, Santa Fe, Tucumán), Bolivia (Santa Cruz), Brazil (Bahía, Ceará, Espírito Santo, Maranhão, Mato Grosso, Minas Gerais, Paraná, Rio de Janeiro, Rio Grande do Norte, Rio Grande do Sul, São Paulo, Santa Catarina, Tocantins), Uruguay (Montevideo).

##### Remarks.

This one of the most studied species of the subfamily and is probably the most common and widespread species of Symphrasinae in southern South America. Previously, *T.varia* was known from Argentina, Brazil, Uruguay, Venezuela, and Suriname (Penny 1977). Here the first record from Bolivia (Santa Cruz) is presented, and the first records for the Brazilian states of Bahía, Espírito Santo and Minas Gerais are also provided. This latter record was published by Silva et al. (2016), yet the species was cited as *Plegazikani*.

A recent study of Mantispidae in Brazil reported *T.varia* from Amazonas, Pará, Maranhão, Ceará, Rio Grande do Norte, Acre, Rondônia, Mato Grosso, Tocantins, Rio de Janeiro, São Paulo, Santa Catarina, and Rio Grande do Sul (Alvim et al. 2019). Herein, based on the material studied for this revision, the records from northern South America, i.e., Venezuela and Suriname are more likely erroneous as suggested by Penny and da Costa (1983). Additionally, the records of the Amazonian states of Brazil (i.e., Acre, Amazonas, Pará, and Rondônia) (Alvim et al. 2019) are also probably doubtful and likely result of misidentification of other species.

This species has important variation in the body coloration pattern and male genitalia. In Argentina and Uruguay, specimens with faint marks on the wings are frequently collected, while in Southern Brazil, specimens with strong maculation on the wings are common. Such variation has led to a considerable confusion about the identity of this species (Penny 1982a; Penny and da Costa 1983). For instance, Penny and da Costa (1983) redescribed this species two times, i.e., as *T.varia* and as *T.zikani*, and Silva et al. (2016) reported this species from Minas Gerais as *P.zikani*. Moreover, the number and arrangement of processes on the male gonocoxites IX is variable as well, having from two to four processes of which when present a preapical processes can be well separated from the apical ones. The number and arrangement are variable not only in specimens from different localities (e.g., Argentina, Brazil) but also in the same specimen on each side of the body.

The name of *Trichosceliazikani* was erroneously introduced by Penny (1982a) and Penny and da Costa (1983) without revising the type specimen, whose original combination is *Plegazikani* Navás, 1936 (Ohl 2004). This mistake was then fixed by Ohl (2004) indicating that the type had all the diagnostic characteristics of the genus *Plega*. Herein, this correction is further confirmed as *Plegazikani* is redescribed as part of this revision.

Even though *T.varia* expresses an important variation, the pattern of marks on the wings (no matter if these are faint or strongly marked), the antennal flagellomeres as long as wide, and the narrow forefemur of this species are useful characters to recognize this species.

#### ﻿﻿ Trichoscelia vespiformis

Taxon classificationAnimaliaNeuropteraRhachiberothidae

Ardila-Camacho & Contreras-Ramos sp. nov.

https://zoobank.org/9BEE3B0D-01C2-4878-89E0-ED18DE41C509

Figs 115 , 116

##### Type locality.

Brazil, **Rondônia**: 62 Km SW Ariquemes nr Fzda. Rancho Grande, 3–15 Dec. 1996; J.E. Eger leg. MV & black lights.

##### Material examined.

***Holotype*** male, pinned. Original label: “Brazil, **Rondônia**, 62 Km SW Ariquemes nr Fzda. Rancho Grande, 3–15 Dec. 1996; J.E. Eger leg., MV & black lights” DZUP. ***Paratypes***. Brazil • 1 ♂ 3 ♀; **Rondônia**, 62 Km SW Ariquemes nr Fzda. Rancho Grande; 3–15 Dec. 1996; J.E. Eger leg.; MV & black lights; FSCA.

##### Diagnosis.

This species has the same coloration pattern exhibited by *T.geraldoi*, mostly composed of blackish brown, with reduced cream areas. Like in *T.geraldoi*, the basal abdominal segments are cream colored. On the male genitalia, the gonocoxite IX is long, arched, and thin, with the posterior apex thickened, and equipped with 10 or 11 digitiform processes arranged in a cluster. The dorsal part of the median lobe of the gonocoxites XI is rounded, and broadly concave, while in *T.geraldoi* it is straighter. On the female genitalia, the gonocoxites + gonapophyses VIII are completely fused; the medial part is composed of two medially joined, trapezoidal plates forming a keel equipped with a posteromedial bilobed process, whose lobes are short and blunt; the lateral part of gonapophyses VIII is an enlarged, trapezoidal plate. Furthermore, the tergite IX + ectoproct is semi-triangular, and the spermatheca is longer and thinner than in *T.geraldoi*.

##### Etymology.

The specific epithet of this species is a composite of the Latin –*vespa*– meaning wasp and the suffix *formis*, which means having form of, alluding to the mimicry pattern of this species with a vespid wasp.

##### Description.

***Measurements*.** Male (*n* = 2). Forewing length: 10.0–10.2 mm; Hind wing length: 7.5–7.7 mm. Female (*n* = 3). Forewing length: 10.3–10.7 mm; Hind wing length: 7.7–7.9 mm.

**Figure 115.**
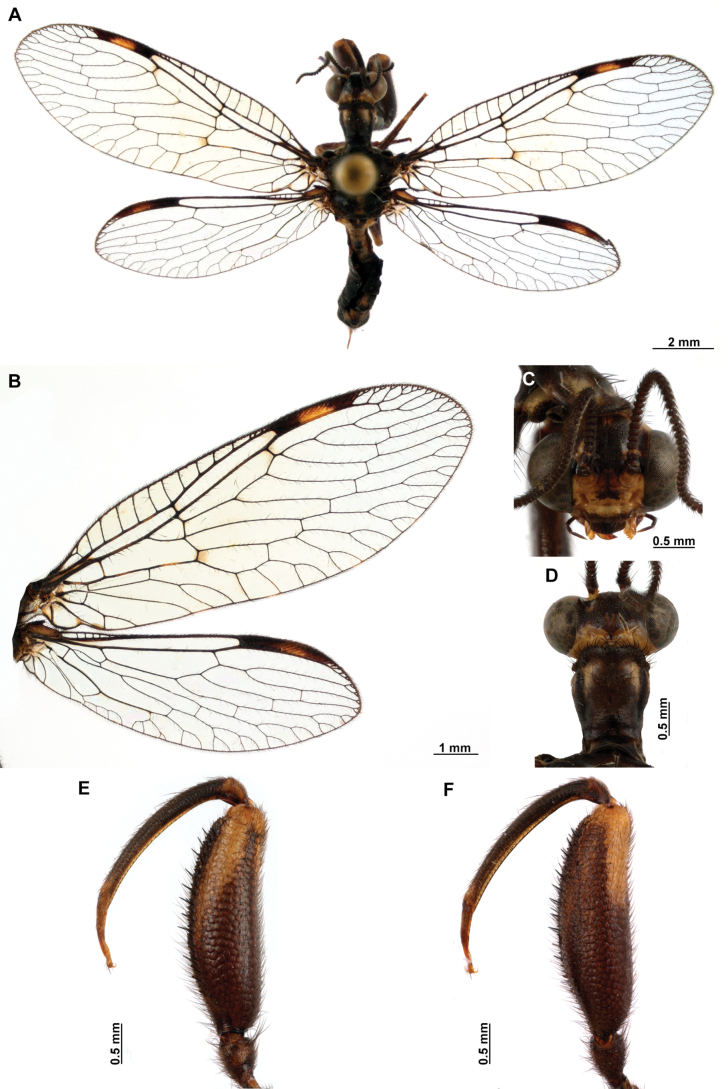
*Trichosceliavespiformis* Ardila-Camacho & Contreras-Ramos, sp. nov. **A** female habitus, dorsal **B** wings **C** head, frontal **D** pronotum, dorsal **E** forefemur, anterior surface **F** same, posterior surface.

***Coloration*** (Fig. 115). ***Head*.** Mainly cream, vertexal region blackish brown, with a pair of cream-colored lateral markings on paraocular area, setae blackish brown; postgena cream; hypostomal bridge ocher. Antennal scape cream on proximal ½, dark brown on distal ½, setae blackish brown; pedicel and flagellum dark brown. Frons with brown triangular mark; clypeus either with posterior ½ cream and anterior ½ brown, or brown with cream margins; labrum dark brown, paler towards lateral margins; mandible dark brown at base, changing to amber towards apex; maxilla cream proximally, changing to amber towards apex, palpus dark brown; submentum brown with pale areas, pale brown setae, labial palpus dark brown, palpimacula pale brown. ***Thorax*.** Pronotum blackish brown, with a pair of cream markings near the anterior margin, setae blackish brown; episternum dark brown, with a cream marking on center; postfurcasternum blackish brown. Mesonotum blackish brown; metanotum blackish brown with central cream marking. Pre-episternum blackish brown. Pteropleura dark brown, with ventral margin of anepisterna cream, ventral margin of metakatepisternum cream. ***Foreleg*.** Coxa and trochanter blackish brown; femur dark brown femur with cream area on distal 1/3 extending apically towards the dorsal surface. Tibia brown, clavate setae pale brown. Basitarsus brown, second to fourth tarsomere pale brown. ***Mid- and hind leg*.** Mid-leg with coxa and trochanter blackish brown; femur blackish brown, with cream apex; tibia dark blackish brown at base, apex, and dorsal surface, the rest cream; basitarsus pale brown, eutarsus brown. Hind leg with coloration similar to that of mid-leg, except for tibia, completely brown. ***Wings*.** Forewing hyaline, sometimes with amber transverse band on proximal 1/3 (area adjacent to 1sc-r, R fork, 1r-m, M fork, 2m-cu and first branch of CuA) and on distal area of subcostal field and adjacent to 1ra-rp; pterostigma dark brown with yellow medial area; longitudinal veins either mostly dark brown, or with yellow areas on proximal ½ of wing; subcostal veinlets and crossveins dark brown; wing margin alternating dark brown and yellow or pale brown. Hind wing hyaline; pterostigma brown with pale brown preapical area; venation dark brown. ***Abdomen*.** Tergites of first two abdominal segments cream with a dark brown medial mark; tergites VII and VIII of male cream with a blackish brown medial mark, tergites VI and VII in female brown with lateral paler areas; tergites of remaining segments blackish brown. Sternites of segments I–III cream-colored, the rest blackish brown. Pleural membrane pale yellow on first three abdominal segments, dark brown in the rest.

***Morphology*** (Fig. 115). ***Head*.** Diamond-shaped in frontal view, moderately setose, rugose, vertexal region domed above compound eyes; paraocular area concave; coronal suture discrete. Postgena broad, hypostomal bridge not completely fused to it. Compound eye hemispheric, as wide as ¾ of interocular distance at toruli level. Antenna moniliform, scape ~ 2× as long as wide, sub-cylindrical, with thin and short setae, thicker and longer on ventral surface; pedicel as long as wide; flagellum not dorsoventrally compressed, with 46–49 flagellomeres, ~ 2× as wide as long on the basal ½, the remaining 1.5× as wide as long, progressively smaller towards the apex; all flagellomeres with medial row of short, thickened setae. Maxillary palpus with the first two palpomeres as long as wide, third palpomere 3× as long as wide, fourth 2× as long as wide, fifth palpomere as long as the third. Submentum oval with long setae; labial palpus with first palpomere 1.5× as long as wide, second 3× as long as wide, third slightly longer than second, basal ½ expanded, palpimacula ovoid. ***Thorax*.** Pronotum 1.5× as long as wide, with a furrow contiguous to lateral and anterior margins; in lateral view anterior margin, medial region and posterior margin slightly elevated, with pedicellate, thickened setae, anterior margin also with long and thin setae; episternum with short, thin setae, postfurcasternum trapezoidal ventrally projecting towards body medial axis. Mesonotum slightly wider than long, with thick, pedicellate on medial area. Metanotum ~ 2× as long as wide, glabrous. Pteropleura covered with abundant short, thin setae. ***Foreleg*.** Coxa slightly shorter than femur, cylindrical, setose; trochanter globular; femur thickened, setose; closing surface with double row of tubercle-shaped specializations, fully developed, both with a more developed sub-basal process (ratio process/setae 1:1); rows of thickened, pedicellate setae with globular base adjacent to each row of processes present, extending over almost the entire closure surface. Tibia almost as long as femur, curved, closing surface with prostrate setae, anterior surface with apical patch of clavate setae; basitarsus elongated, ventrally keeled, with single row of prostrate setae; anterior surface with patch of clavate setae; lanceolate process reaching the base of fourth tarsomere, with plug-shaped Stitz organ at apex; second tarsomere 4× as long as wide, third tarsomere as long as wide, fourth tarsomere 2× as long as wide. ***Mid- and hind leg*.** Mid-leg with interspersed long and short, thin setae; basitarsus 3.5 times as long as wide, second tarsomere slightly longer than wide, third and fourth as long as wide, fifth tarsomere 2× as long as wide; the first four tarsomeres with lateral pair of thickened setae on distal margin of plantar surface. Hind leg longer than mid-leg, densely setose; tibia 1.5× as long as femur; tarsomeres similar to those of mid-leg. ***Wings*.** Fore wing oval, trichosors present along wing margin except at the base, venation setose; costal space narrow, humeral vein forked, 12–14 subcostal veinlets; pterostigma elongated and narrow, composed of numerous venules, mainly incomplete; subcostal space with single, medially located crossvein; Sc vein bent posteriad at proximal margin of pterostigma to merge the RA; *rarp2* gently curved, with three veins arising from it, two from *rarp1*; M basally fused to R; RP base located near separation of M and R, M near such separation, opposite to R fork; 1r-m located between RP base and MA base, forming a small trapezoidal cell; four graded transverse veins present. Cu vein deeply forked; CuP basally angled, approaching A1, distally forked, opposite to separation of M and R. 1A simple, reaching the level of separation of M and R; A2 distally forked at level of CuP angle. Hind wing smaller and narrower than forewing; costal space narrow and reduced, with five veinlets; Sc and C fused at 1/5 of wing length, Sc curved posteriad at proximal pterostigma margin to merge the RA; pterostigma elongated, narrow, curved, composed of numerous, incomplete veinlets; radial space with a single crossvein, oblique; two veins arising from *rarp1*, one from *rarp2*. M forked beyond the R fork; 1 r-m sigmoid, connecting M base and RP base, distally connected again to M stem through short crossvein. Cu deeply forked, CuA long, sinuous, distally forked, first branch candelabrum-shaped; CuP distally anteriorly bent, pectinate. Cubitoanal space with two crossveins. A1 arched, A2 short and arched. ***Abdomen*.** Cylindrical, tergites quadrangular; intertergal membranes between segments II–VII in male and III–VI in female broad, covered with microtrichia; sternites rectangular.

**Figure 116.**
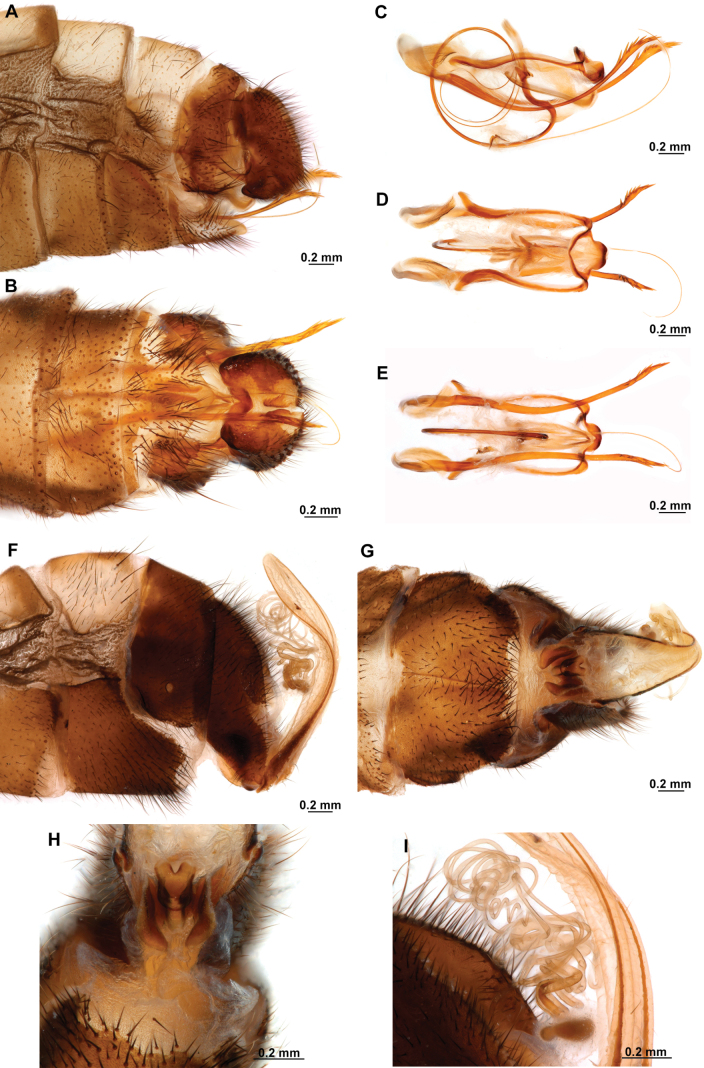
*Trichosceliavespiformis* Ardila-Camacho & Contreras-Ramos, sp. nov. **A** male terminalia, lateral **B** same, ventral **C** male genitalia, lateral **D** same, dorsal **E** same, ventral **F** female terminalia, lateral **G** same, ventral **H** gonapophyses VIII ventral **I** spermatheca.

***Male genitalia*** (Fig. 116A–E). Tergite IX dorsally slightly narrower than laterally, lateral margin posteroventrally notched, curved towards the central axis of abdomen. Sternite VIII rectangular; sternite IX semi-triangular, setose, posteromedially forming an obtuse angle, glabrous, dorsally canaliculate; in lateral view acuminate, with the apex not reaching posterior margin of ectoproct. Gonocoxite IX long, arched, and thin, base spatulate, curved inwards; apex thickened with 10 or 11 digitiform processes arranged as a cluster. Ectoproct trapezoidal; ventrally with convex area covered with microtrichia on inner surface, posteriorly keeled, sclerotized area, covered with microtrichia is present. Gonocoxites X forming a short, thickened, ventrally canaliculate sclerite, anterior apex expanded, posterior apex bilobed, two additional preapical processes connected to gonapophyses X are present. Gonostyli X with thickened and straight base, set with two lateral processes, the rest of the structure, long, ventrally curved, and anteriorly coiled, forming two internal loops before protruding from the abdomen. Gonapophyses X straight, thin, posterior apex dorsally bent, with surrounding membrane covered with granules; gonapophyses arranged in a V-shaped structure, joined by a membrane covering base of gonostyli X. Gonocoxites XI U-shaped, median lobe elaborated, with two differentiated parts: dorsal part as a rounded lobe; ventral part consisting of a convex area covered with microspinules; ventral margin with V-shaped incision; between these parts a narrow, less-sclerotized region is present. Lateral arms of gonocoxites XI with anterior apex laterally curved.

***Female genitalia*** (Fig. 116F–I). Sternite VII trapezoidal. Tergite VIII almost as wide medially as laterally, enclosing the spiracle of the eighth abdominal segment, ventral margin quadrangular. Membrane between tergite VIII and tergite IX + ectoproct sclerotized, forming a concave, triangular plate. Gonocoxites + gonapophyses VIII with a medial part composed of two medially joined, trapezoidal plates forming a keel, posteromedially with bilobed process, whose lobes are short and blunt; lateral part of gonapophyses VIII an enlarged trapezoidal plate, connected to tergite IX + ectoproct. Tergite IX + ectoproct semi-triangular. Gonocoxite IX long and narrow, as long as the last three abdominal segments together. Bursa copulatrix short, funnel-shaped, proximal region, sclerotized, concave, the rest membranous; spermatheca complex and entangled; proximal section long and thin, forming around 10 coils, ending in a more internal spiral; medial section slightly thicker than proximal section, entangled, forming multiple convolutions. Distal section almost as wide as medial section, recurved, progressively wider towards apex, distally with trumpet-shaped invagination. Fertilization canal duct short, thin, sigmoid; fertilization canal reniform, covered with microfilaments.

##### Distribution.

Brazil (Rondônia).

##### Remarks.

This species is known only from its type locality in the Brazilian state of Rondônia. The body coloration of this species is basically indistinguishable from that of *T.geraldoi*, with which it shares the same mimicry pattern resembling the vespid wasp *Polybiadimorpha*. Nonetheless, the genitalia of both species are well differentiated, and can be easily separated with the morphology of the male gonocoxites IX and the female gonocoxites VIII.

Considering the morphology of the male genitalia, this species appears to be closely related to *T.geraldoi*, *T.basella*, *T.involuta*, *T.fenella* and other Amazonian species.

#### ﻿﻿ Trichoscelia wintertoni

Taxon classificationAnimaliaNeuropteraRhachiberothidae

Ardila-Camacho & Contreras-Ramos sp. nov.

https://zoobank.org/50D33147-84E0-4245-BA85-CED850620DA8

Figs 117 , 118

##### Type locality.

Brazil, **Rond**ô**nia**: 62 Km SW Ariquemes, nr Fzda. Rancho Grande, 5–17 Oct. 1993, J.E. Eger leg., M.V. & black lights.

##### Material examined.

***Holotype*** male, pinned, genitalia cleared and stored in a separate vial. Original label: “Brazil, **Rond**ô**nia**, 62 Km SW Ariquemes, nr Fzda. Rancho Grande, 5–17 Oct. 1993, J.E. Eger leg., M.V. & black lights” DZUP.

##### Diagnosis.

This species is distinguished by the color pattern of the forewing venation, having extensive yellowish areas on medial region of C, Sc, and RA, most of the RP, proximal region of RP branches, and sub-basal region of M branches. Among the species of the genus, *T.wintertoni* sp. nov. is recognized because the forefemur is distinctively incrassate. On the male genitalia, the Gonocoxite IX is short and thickened, with posterior apex dorsolaterally curved, and equipped with three apical digitiform processes, two lateral, long, and subequal, and one preapical, medially located, and shorter.

##### Etymology.

This species is named after Shaun Winterton, a prominent entomologist, neuropterologist, and great human being who has advanced our understanding of the evolution of Neuropterida enormously.

##### Description.

***Measurements*.** Male (*n* = 1). Forewing length: 9.3 mm; Hind wing length: 6.8 mm.

**Figure 117.**
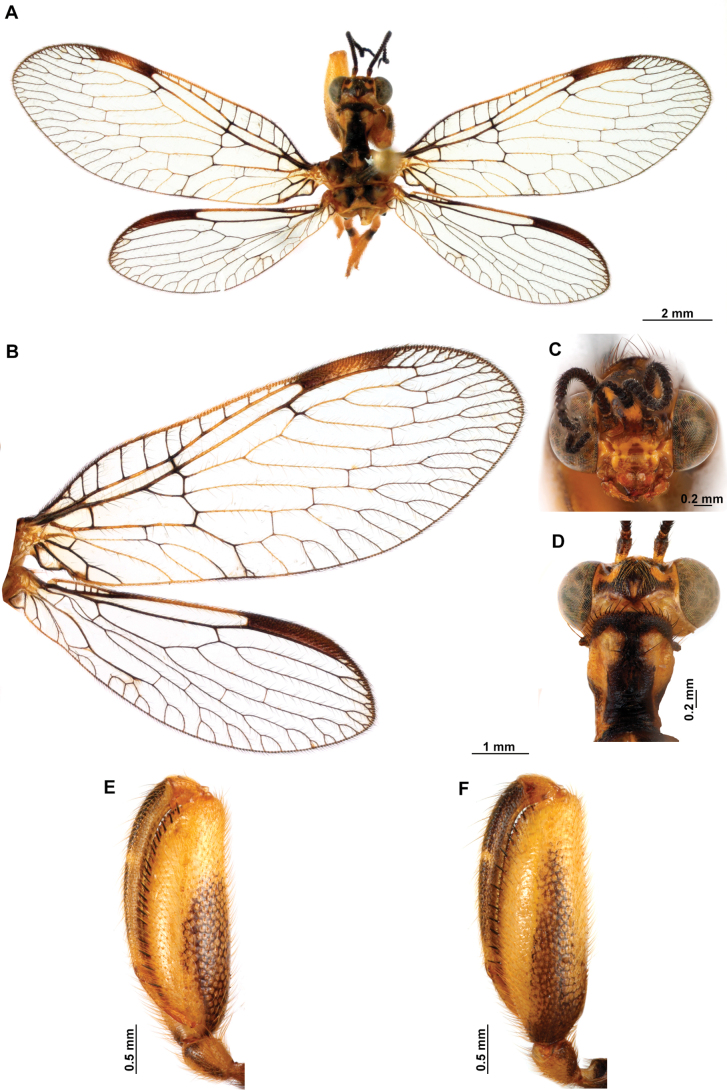
*Trichosceliawintertoni* Ardila-Camacho & Contreras-Ramos, sp. nov. **A** female habitus, dorsal **B** wings **C** head, frontal **D** pronotum, dorsal **E** forefemur, anterior surface **F** same, posterior surface.

***Coloration* (Fig. 117)**. ***Head*.** Yellow, vertexal region with brown mark that is anteriorly forked, and laterally projected towards ocular margin; medially enclosing yellow area adjacent to coronal suture; setae pale brown on posterior 1/3, anteriorly changing to yellow. Antennal scape yellow on proximal ½, brown on distal ½; pedicel and flagellum brown. Labrum pale brown, with yellow lateral edges; mandible proximally yellow, distally pale amber; maxilla predominantly yellow, except pale amber galea and lacinia; labium mainly yellow, ligula brown ligula, labial palpus pale brown, palpimacula yellow. ***Thorax*.** Pronotum yellow with brown, medial band that is extended laterally on anterior margin, and two posterolateral spots; episternum yellow with anterior brown marking; postfurcasternum yellow with posterior dark brown area. Mesonotum yellow, with brown lateral areas on scutum, anteromedially with two brown triangular markings, scutellum yellow; metanotum yellow, scutum with dark brown, arched lateral marks, scutellum with anterior brown spot. Pre-episternum mostly brown. Mesopleuron yellow with brown markings on episternum; metapleuron yellow. ***Foreleg*.** Coxa mostly brown, dorsally with yellow areas; trochanter yellow, with brown bands on posterior and ventral surfaces. Femur yellow, with an elongated area composed of brown markings forming a honeycomb pattern extending anterodorsally on proximal ½ and medially on posterior surface. Tibia yellow, with two extensive dark brown areas on posterior and dorsal surfaces; clavate setae yellow. Basitarsus mostly pale brown; eutarsus yellow. ***Mid- and hind leg*.** Mid-leg with coxa brown, trochanter, femur, and tibia yellow, tarsus pale brown. Hind leg similar to mid-leg, except for base of tibia brown. ***Wings*.** Forewing hyaline, pterostigma brown with paler medial area; longitudinal veins with brown and yellow areas, subcostal veinlets and crossveins brown; wing margin brown, paler on medial area of costal margin and base of posterior margin. Hind wing hyaline; pterostigma uniformly brown; venation mostly brown, except C + Sc pale brown; wing margin brown, paler at base. ***Abdomen*.** Cleared.

***Morphology* (Fig. 117)**. ***Head*.** Diamond-shaped in frontal view, moderately setose, smooth; vertexal region domed above compound eyes; paraocular area concave; coronal suture discernible, with adjacent muscle attachment mark. Postgena wide, hypostomal bridge delimited by ridge. Compound eye hemispheric, as wide as ¾ of interocular distance at torulus level. Antenna moniliform, scape 2× as long as wide, slightly distally expanded, pedicel as long as wide; flagellum slightly dorsoventrally compressed, with 45 flagellomeres, 1.5× as wide as long on basal region, progressively changing to almost as wide as long, smaller towards the apex; all articles with medial row of thickened setae. Maxillary palpus with first two palpomeres as long as wide, third 3.5 times as long as wide, fourth 2.5× as long as wide, fifth slightly shorter than third. Postmentum oval with long and thickened setae, palpus with first palpomere 1.5× as long as wide, second 2.5× as long as wide, third slightly longer than second, widely expanded, palpimacula ovoid. ***Thorax*.** Pronotum 1.5× as long as wide, with furrow contiguous to lateral and anterior margins; in lateral view anterior margin, medial region and posterior margin slightly elevated, with thickened, pedicellate setae; episternum with long, thin setae; postfurcasternum trapezoidal. Mesonotum 1.5× wider than long, with thick, pedicellate setae on medial area of scutum and scutellum. Metanotum 2× as wide as long, glabrous. Pteropleura covered with abundant short and thin setae, some thicker on mesanepisternum. ***Foreleg*.** Coxa slightly shorter than femur, cylindrical, with abundant thin setae; trochanter subtrapezoidal; femur notably robust, ovoid, with abundant short and thin setae; closing surface with double row of tubercle-shaped specializations, both with a more developed sub-basal process (process/setae ratio 2:1); rows of thickened setae with globular base adjacent to each row of processes present, extending over almost the entire closure surface. Tibia almost as long as femur, curved, setose, closing surface with prostrate setae; with patch of clavate setae apically on anterior surface; basitarsus elongated, ventrally keeled, with single row of prostrate setae on proximal ½, anterior surface with patch of clavate setae proximally; lanceolate process reaching the base of fourth tarsomere, with plug-shaped Stitz organ at apex; second tarsomere 4× as long as wide, third tarsomere as long as wide, fourth tarsomere 2× as long as wide. ***Mid- and hind leg*.** Mid-leg coxa covered with long, thin setae; trochanter, femur, tibia, and tarsus, covered with short, thin setae; basitarsus 4× as long as wide, second tarsomere slightly longer than wide, third and fourth as long as wide, fifth tarsomere 2× as long as wide; the first four tarsomeres with a pair of thickened setae laterally on distal margin of plantar surface. Hind leg longer than mid-leg; tibia, 1.5× as long as femur; tarsomeres as in mid-leg. ***Wings*.** Forewing oval, trichosors present along wing margin except at basal ¼ of posterior margin, venation setose; costal space slightly widened medially, humeral vein forked; 12 subcostal veinlets present; pterostigma elongated, narrow, gently curved, composed of numerous, mainly incomplete veinlets; subcostal space with single, medially located crossvein; Sc vein bent posteriad at proximal margin of pterostigma to merge the RA; *rarp2* curved, with three veins arising from it, two from *rarp1*; M fused basally to R; base of RP located near the separation of M and R, M fork near such separation, opposite to R fork; 1r-m located between RP base and MA base forming a trapezoidal cell; four gradate crossveins present. Cu vein deeply forked; CuP basally angulated, approaching A1, forked slightly before separation of M and R. A1 simple, reaching the level of separation of M and R; A2 distally fork, at the level of CuP angle. Hind wing smaller and narrower than anterior; costal space narrow and reduced, with six veinlets; Sc and C fused at 1/5 of wing length; Sc curved posteriad and proximal margin of pterostigma to merge the RA; pterostigma elongated, narrow, curved, composed of numerous incomplete veinlets; radial space with a single crossvein, oblique; two veins arising from *rarp1*, one from *rarp2*. M forked beyond R fork; 1r-m sigmoid, distally forming a right angle, connecting M base and RP base, distally connected again to M stem through vestigial crossvein. Cu deeply forked, CuA long and sinuous, distally forked, first branch candelabrum-shaped; CuP distally forked, distally anteriorly curved, pectinate. Cubitoanal space with two crossveins. A1 arched, A2 short, arched, distally forked. ***Abdomen*.** Cylindrical, tergites quadrangular; intertergal membranes between segments III–V, covered with microtrichia, with glabrous lateral areas; sternites rectangular.

**Figure 118.**
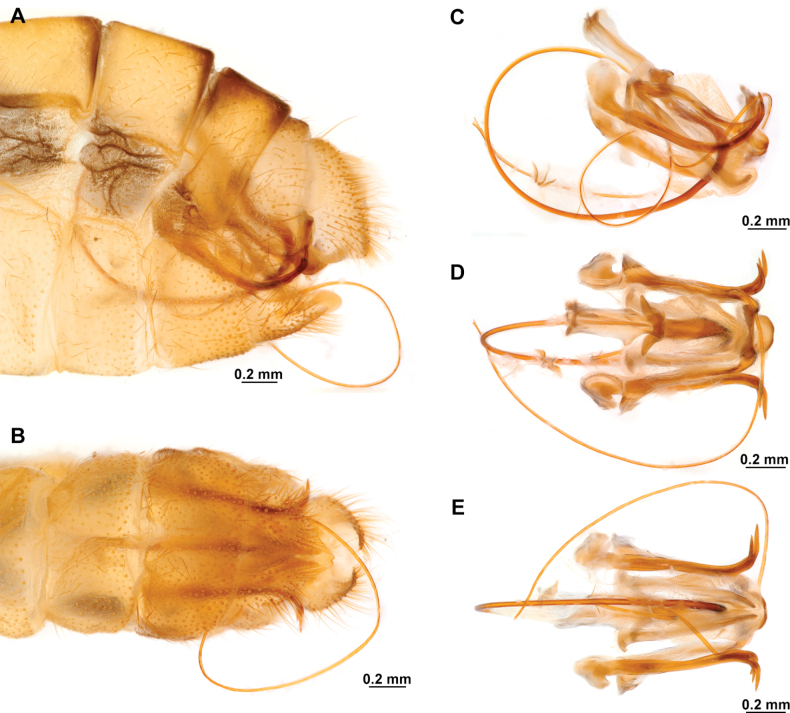
*Trichosceliawintertoni* Ardila-Camacho & Contreras-Ramos, sp. nov. **A** male terminalia, lateral **B** same, ventral **C** male genitalia, lateral **D** same, dorsal **E** same, ventral.

***Male genitalia*** (Fig. 118). Tergite IX medially narrower than laterally, lateral margin trapezoidal, with posterior notch. Sternum VIII rectangular; sternite IX pentagonal, posteromedially with small incision, glabrous, dorsally canaliculated, the rest of the surface with abundant long and thin setae; in lateral view with blunt apex, not reaching posterior margin of ectoproct. Gonocoxite IX short and thickened, base spatulate, dorsally bent; apex dorsolaterally curved, with three apical digitiform processes, two lateral, long, subequal, and one preapical, medially located, shorter. Ectoproct triangular, setose; ventral surface with an ovoid, convex, sclerotized anterior area, continued posteriorly by less sclerotized, concave area. Gonocoxites X forming a short, straight, ventrally canaliculate sclerite; anterior apex slightly expanded, posterior apex bilobed, and with two lateroventral processes. Gonostyli X with thickened and curved base, with two lateral processes, the rest of the structure long, curved ventrally and coiled anteriorly, before protruding from the abdomen. Gonapophyses X straight, thin, with dorsally bent posterior apex; gonapophyses arranged in a V-shaped structure, joined by membrane covering base of gonostyli X. Gonocoxites XI U-shaped median lobe expanded and elaborated, with two differentiated parts: dorsal part a quadrangular lobe; ventral part as a convex area covered with microspinules, with a V-shaped ventral edge; between these parts a broad, less sclerotized region is present. Lateral arms of gonocoxites XI sigmoid, anterior apex flattened and expanded.

##### Distribution.

Brazil (Rondônia).

##### Remarks.

This species is known only from its type locality in Rondônia, Brazil. Based on the markedly robust forefemur and coloration pattern, *T.wintertoni* is quite distinctive and easy to recognize among other Amazonian species. The morphology of the male genitalia allies this species with others from the Amazonia, like *T.basella*, *T.geraldoi*, and *T.involuta*.

## ﻿﻿Discussion

In this work a comprehensive taxonomic revision of the extant species of subfamily Symphrasinae is presented. This group is composed of three genera, *Anchieta* (11 spp.) distributed from Costa Rica to Southern Brazil, *Plega* (28 spp.) found from southwestern United States to southern Brazil, and *Trichoscelia* (21 spp.) known from southern Mexico to Argentina. It is clear that the center of species diversity of this group is in northern South America, Central America and Mexico. The three genera are found in South and Central America, with several species exhibiting plesiomorphic character states found in the Amazonia, Andean region, and Panama Isthmus (e.g., *A.fasciatellus*, *A.partheniellus*, *P.radicaudata*, *Trichoscelia* spp.). Interestingly, such species share many characteristics with rhachiberothines, particularly on the structure of the foreleg. Two species from Mexico, namely *P.insolita* and *P.drepanicoides* also express several characters that allies symphrasines and rhachiberothines. For example, in the former the structure of the foreleg and prothorax is noticeably similar to those of rhachiberothines, while in the latter the female genitalia exhibit a paired sternite VII (or gonocoxites VII), a character expressed by *Mucroberotha*, *Rhachiberotha*, and even by certain extinct mantispoids like Dipteromantispidae (Ardila-Camacho et al. 2021a).

The divergence between Symphrasinae and Rhachiberothinae was estimated ~ 115 Mya in the Cretaceous based on reduced representation of the genome (Winterton et al. 2018). On the other hand, several fossils of the Symphrasinae and Paraberothinae have been discovered in amber deposits of Myanmar (Lu and Liu 2021; Li et al. 2023), which is also from the Cretaceous period. These data suggest a very ancient divergence of these groups, although the existence of *Symphrasiteseocenicus*, a compression fossil from the Eocene of Germany and the numerous rhachiberothid fossils discovered from different deposits from the Cretaceous and Eocene suggest these groups had a more widespread distribution during the Mesozoic (Wedmann and Makarkin 2007; Nakamine et al. 2020). Interestingly, the divergence between Rhachiberothidae (including Symphrasinae) and other mantispoids was calculated in the early Jurassic (185–190 Mya) (Winterton et al. 2018). The long evolutionary history of these groups and their noticeable morphological divergence, plus the extinction of several lineages, would explain the difficulties in understanding the phylogenetic relationships of the rhachiberothid subfamilies.

The extant members of the crown group of Symphrasinae underwent an important diversification in the New World, with their center of origin probably in northern South America. This highly specialized group is evidently distinct from other mantispoids, exhibiting many derived character states within Rhachiberothidae, although features of the foreleg (specifically of the foretarsus) provide a clear indication of its close relationship with other rhachiberothids (Ardila-Camacho et al. 2021a). The mean divergence between *Plega* and *Trichoscelia* was calculated between 25 and 30 Mya during the Oligocene (Winterton et al. 2018), and *Anchieta* probably diverged from them shortly before, but also during the Paleogene.

In the present study, the subfamily Symphrasinae was recovered as monophyletic, with *Anchieta* as sister group of *Plega* + *Trichoscelia* based on careful examination of all species included in this taxonomic revision. The monophyly of this subfamily was supported by numerous unambiguous character changes. Among these, the presence of hypostomal bridge is unique to Symphrasinae, although it is also expressed by some Coniopterygidae and Nemopteridae (Zimmermann et al. 2009; Randolf et al. 2017; Randolf and Zimmermann 2019). A robust forefemur with convex anterior and posterior surfaces is also observed within the raptorial Mantispoidea in *Hoelzeliellamanselli* and Dipteromantispidae (Aspöck and Aspöck 1997; Li et al. 2020). The anteroventral row of integumentary specializations reduced to proximal region and apex, in which the proximal-most process is a curved, primary process (with a loss of in *Trichoscelia*), the presence of thickened setae with globular base adjacent to the rows of processes, the proximally curved closing surface are until now only observed in the raptorial leg of this subfamily. Among these characters the basal primary process of the anteroventral row of integumentary specializations was previously interpreted as a synapomorphy of *Anchieta* + *Plega* (Penny and da Costa 1983). On the wings, the Sc vein fused to RA at pterostigma level is also expressed by certain berothids, and the extinct mantispoid subfamilies Paraberothinae and Doratomantispinae (Nakamine et al. 2020; Li et al. 2022). On the HW, the C and Sc veins are fused slightly beyond the level of R fork, a condition observed in *Nolima*, although in this genus such fusion occurs much before R fork level (Ardila-Camacho et al. 2021a). On the male genitalia, the gonocoxites IX are equipped with digitiform processes, although secondary loss of this condition is observed in a group of species of *Plega* (i.e., *P.obtusa*, *P.oswaldi*, *P.acevedoi*, *P.longicornis* and *P.spinosa*) and some *Anchieta*. Moreover, the paired male gonapophyses X, not fused to gonocoxites X are expressed in Nallachinae, *Rhachiberothasheilae*, and Doratomantispinae (Ardila-Camacho et al. 2021a; Li et al. 2022). Finally, the absence of callus cerci on female terminalia is expressed in other mantispoids like Mantispinae, or even in other Neuroptera.

Other characters expressed by members of Symphrasinae include the procoxal insertion approximately at mid-length of prothorax, the 4-segmented foretarsus in both sexes, with the basitarsus exhibiting a lanceolate process, the presence of more than two trichosors on the posterior wing margin of FW between the apex of two longitudinal veins, the presence of two crossveins on the radial space of FW, the anterior branch of A1 of HW not fused to CuP, the female gonocoxites + gonapophyses VIII forming an unpaired, complex structure, and the hose-shaped gonocoxites IX (ovipositor). Among Neuroptera, this latter structure is only present in Dilaridae as a result of parallelism (Aspöck and Aspöck 2008).

The genus *Anchieta* is a striking and highly derived taxon composed of species expressing Batesian mimicry with Braconidae, Vespidae, and Meliponini (Apidae) (Rasmussen and Ardila-Camacho 2021). Synapomorphies supporting the monophyly of this genus include the rectangular frontal sclerite, the short, raised area on the posterior margin of pronotum, a blunt process on anterior surface of foretrochanter, the posteroventral row of integumentary specializations with single sub-basal, primary process, a rectangular pterostigma of FW, and a single crossvein on the cubitoanal space of the HW. On the female genitalia, the distal section is noticeably expanded, and the fertilization canal duct is spiral-shaped. In the phylogenetic analysis performed here, *A.fasciatellus* was recovered as sister to the rest of the species, exhibiting several characters in common with *Plega* and *Trichoscelia* such as the gently curved sub-stigmal cell of FW and the intracubital crossvein of the HW subparallel to the longitudinal wing axis. Furthermore, the mimicry pattern of this species resembling parasitoid wasps of the family Braconidae equipped with abdominal repugnant glands is unique within the genus. This species is distributed in Panama, and in the Magdalena Basin and the Caribbean region in Colombia, being separated from the vast majority of its congeners (mostly Amazonian) by the mountainous chains of the Andes. Consequently, this species probably diverged from the ancestor of the rest of *Anchieta* species during the uplifting of the Central Andes during the Paleogene (~ 65–34 Mya) (Hoorn et al. 2010). This group underwent important diversification across the Amazonia and the Atlantic Forest in Brazil, where species mimicking vespid wasps and stingless bees evolved. The evolution of these mimicry patterns with the wasp mimicking species as sister to the bee-mimicking clade, apparently trace the evolution of the wasps and bees (Peters et al. 2017). This clade is supported by the straight substigmal cell of FW, the hind wing reaching the proximal margin of pterostigma of FW, the reclined intracubital crossvein of the HW, and the expanded and dorsally curved anterior region of the male gonocoxites X. The bee-mimicking lineage for its part, is supported by the expanded hind tibia, and the presence of keeled processes on abdominal tergites (although not including tergites V, VI, and VII).

The sister-group relationship between the genera *Plega* and *Trichoscelia* is herein proposed for the first time. Geographical distribution of both genera is largely overlapped, and a species of *Plega* from South America (i.e., *P.radicaudata*) exhibits a foreleg remarkably similar to that of the genus *Trichoscelia*. Characters supporting the monophyly of this group include the trapezoidal pterostigma of the FW, which is generally dark with pale medial area, the candelabrum-shaped first branch of CuA of HW, the presence of two crossveins on the cubitoanal space of the HW, the posteromedial canal on the dorsal surface of the male sternite IX, the modifications on the ventral region of the male ectoproct, the anteriorly coiled male gonostyli X forming two loops, and the complex and enlarged median lobe of gonocoxites XI.

The genus *Trichoscelia* has its diversity concentrated in the Amazonia and in the Northern part of South America, including the Andes of Colombia, Ecuador and Peru. Several species are also found in Central America and the Neotropical portion of Mexico. This genus appears to be highly specialized, as all the known records of association of the larvae are exclusively with vespid wasps of the tribe Epiponini (Polistinae). Previous to this work, species of this genus were classified within two species groups, namely the *T.fenella* group and the *T.varia* group, based on the width of the basal antennal flagellomeres (Penny 1982a). Nonetheless, these groups were not recovered as monophyletic in the analysis presented here, and consequently this classification is entirely abandoned. The majority of the species of *Trichoscelia* are small-sized, and with conserved and simplified morphology making difficult the understanding of their phylogenetic relationships. The wing venation and male and female genitalia are noticeably uniform, and basically the species are separated by coloration patterns and the overall morphology of the male gonocoxites IX and the female gonocoxites + gonapophyses VIII. As most of the species are distributed in the Amazon, herein it is hypothesized that this genus underwent a rapid diversification probably related to processes like the continuous uplifting of the Andes and the retreatment of the Pebas system, i.e., a continually shifting pattern of streams swamps and lakes of varying salinity with marine influence from the Caribbean mostly during the Neogene (Wesselingh et al. 2002; Wesselingh 2006; Hoorn et al. 2010), generating dispersal and vicariant events. Climatic fluctuations during the Pleistocene allowed cycles of forest retraction and expansion through glacial/interglacial dynamics (refugia hypothesis) (Gomes da Rocha and Kaefer 2019), plus the formation of the Panama Isthmus would serve as likely explanations for the diversification and present distribution of *Trichoscelia*.

Even though the genus exhibits a conserved morphology, synapomorphies supporting its monophyly are clearly defined and include: a broadly ovoid palpimacula, the posteroventral row of thickened setae with globular base present nearly over the entire extension of femoral closing surface, CuA of FW forming a well-defined obtuse angle, presence of a crossvein connecting the sigmoid 1r-m to M stem on the HW, and male ectoproct equipped with posterior bulging area and anterior concave surface on ventral region.

The genus *Plega*, constitutes the most diverse genus of Symphrasinae, with species occurring from Southern Brazil to Southwestern United States. Numerous characters including the ventrally fused, collar-like postfurcasterna (with a reversal in *P.insolita*), the development of two primary processes on the proximal ½ of the posteroventral row of integumentary specializations of the forefemur, the presence of Stinger-shaped setae on the rows of integumentary specializations of the forefemur, a pentagonal male sternite IX with rounded posterolateral corners, the structure of the distal section of the spermatheca, and the triangular and concave proximal portion of the fertilization canal duct support the monophyly of this genus. The South American species of *Plega* exhibit numerous plesiomorphies, and were recovered as a clade sister of the remaining species of *Plega* in the analysis performed herein. Several species present a remarkably narrowed forefemur, with two fully developed rows of integumentary specializations on the forefemur of one species (i.e., *P.radicaudata*), a character expressed by *Rhachiberotha*, *Rhachiella*, and *Hoelzeliella* (Ardila-Camacho et al. 2021a). The results presented here, recovered *P.zikani* from Southern Brazil as sister of the rest of the species, which form a clade supported by the presence of lateral, setose protuberances on the supraantennal region. The first clade in this latter lineage which includes species from South America (*P.duckei*, *P.paraensis*, *P.hagenella*, *P.bowlesi*, and *P.radicaudata*), plus some species from Central America and Southern Mexico (*P.hagenella*, *P.disrupta*, and *P.yucatanae*) is supported by a single homoplastic character (i.e., bursa copulatrix with membranous and sclerotized areas). A smaller clade within this lineage is composed of *P.hagenella* + (*P.bowlesi* + *P.radicaudata*), and is supported by two synapomorphies of the male genitalia: presence of an anterior, flattened lobe, anteriorly set with conical setal bases on the ectoproct, and the presence of a concave covering or dome which forms a curved process on the median lobe of the gonocoxites XI. The distribution of this group of species appears to indicate, that the center of origin of *Plega* is in South America, and considering the distribution of some species in Central America and southern Mexico, dispersal events and subsequent diversification since around ~20 Ma ago probably took place during the closure of the Panama Isthmus (O’Dea et al. 2016).

A large clade sister to the aforementioned lineage includes the rest of the species of *Plega*, in which *P.drepanicoides* and *P.pseudohagenella*, from Mexico and Central America, respectively form a laddered relationship on the topology. A clade composed of species from Mexico and Central America is supported by a single homoplastic character (i.e., female gonapophyses IX as two tiny sclerites concealed on the inner surface of the base of gonocoxites IX). This clade is composed by *P.vangiersbergenae*, *P.stangei*, *P.lachesis*, *P.obtusa*, *P.oswaldi*, and *P.spinosa*. All the species of this group exhibit a markedly similar morphology, and among these, the clade *P.obtusa* + (*P.oswaldi* + *P.spinosa*) is supported by the presence of an enlarged, digitiform, caudally projected process on the ventromedial, posterior area of the male gonocoxites XI. All of these species evolved in Central America and Southern Mexico, except for *P.obtusa* which is found in Northern Mexico (Sonora) and Arizona (near the boundary between Mexico and the United States). The diversification of this group could be related to vicariant events due to the presence of numerous mountainous barriers and valleys such as the highlands of Chiapas-Guatemala, the Tehuantepec Isthmus, Sierra Madre Oriental, Sierra Madre del Sur, and the Trans-Mexican Volcanic Belt (Morrone 2005). Together with the former lineage, these species would be included within the *P.hagenella* group, a classification proposed by Parker and Stange (1965) and Penny (1982a) based on the morphology of the basal antennal flagellomeres (i.e., as wide as long). Nonetheless, because all the aforementioned species do not form a monophyletic group, the usage of that classification is herein abandoned.

The sister group of the clade mentioned above includes most of the species previously classified within the *P.signata* group, i.e., *P.banksi*, *P.dactylota*, *P.signata*, and *P.fumosa*, which was defined by the presence of discoidal basal antennal flagellomeres (Parker and Stange 1965; Penny 1982a). Herein, the *P.signata* group is redefined, adding *P.mixteca*, *P.flammata*, *P.insolita*, *P.megaptera*, and *P.sonorae*. However, *P.duckei*, an anomalous species belonging to the South American lineage of the genus, and with a different antennal morphology, is here excluded from that group of species. The new concept of the *P.signata* group is supported by four homoplastic characters: basal antennal flagellomeres >3 times as wide as long, the unmodified supra-antennal region, the gently curved substigmal cell of FW, and the male gonocoxites X only slightly wider on anterior apex. This lineage is distributed from Central America, South- and Central Mexico to Southwestern United States, although its center of origin is apparently the former region, with a subsequent dispersal towards the Nearctic. In this clade, *P.mixteca* from Central America and southern and central Mexico was recovered as sister of the rest of the species. Then, *P.dactylota*, a widespread species occurring from Central Mexico to southern United States was recovered as sister to the remaining species of the group. Two sister lineages form a clade supported by two homoplastic characters, and are primarily constituted by Nearctic species. The first includes *P.fumosa*, a species endemic from Michoacan, (Sierra Madre del Sur) as sister to *P.flammata* (Peninsula of Baja California) and *P.signata* (Peninsula of Baja California, California, Nevada, and a small portion of Arizona). This clade is supported by a single homoplastic character (i.e., the undeveloped ventromedial, posterior area of the male gonocoxites XI). The sister relationship of the latter species, is supported by the presence of an enlarged, curved, rugose process on the ventral region of the gonocoxites XI median lobe. The last clade is constituted by four species (*P.insolita*, *P.banksi*, *P.megaptera*, and *P.sonorae*) distributed in xeric areas of Northern Mexico and Southern United States. This group is supported by the presence of a curved process with bilobed apex on the ventral region of the male gonocoxites XI. *Plegainsolita*, is a rather distinctive species recovered as sister to *P.banksi* + (*P.sonorae* + *P.megaptera*). This species is distributed in Sinaloa and Sonora near the Pacific coast, while *P.banksi* is distributed from Sonora and Sinaloa to Arizona. The latter two species, i.e., *P.megaptera* from Chihuahua, and *P.sonorae* from Sonora, are separated by the Mountain chains of Sierra Madre Occidental.

## Supplementary Material

XML Treatment for Symphrasinae

XML Treatment for Anchieta

XML Treatment for Achieta apiculasaeva

XML Treatment for Anchieta bellus

XML Treatment for Anchieta fasciatellus

XML Treatment for Anchieta fumosellus

XML Treatment for Anchieta nebulosus

XML Treatment for Anchieta nothus

XML Treatment for Anchieta partheniellus

XML Treatment for Anchieta remipes

XML Treatment for Anchieta romani

XML Treatment for Anchieta sophiae

XML Treatment for Anchieta tinctus

XML Treatment for Plega

XML Treatment for Plega acevedoi

XML Treatment for Plega banksi

XML Treatment for Plega bowlesi

XML Treatment for Plega dactylota

XML Treatment for Plega disrupta

XML Treatment for Plega drepanicoides

XML Treatment for Plega duckei

XML Treatment for Plega flammata

XML Treatment for Plega fumosa

XML Treatment for Plega hagenella

XML Treatment for Plega insolita

XML Treatment for Plega lachesis

XML Treatment for Plega longicornis

XML Treatment for Plega megaptera

XML Treatment for Plega mixteca

XML Treatment for Plega obtusa

XML Treatment for Plega oswaldi

XML Treatment for Plega paraensis

XML Treatment for Plega pseudohagenella

XML Treatment for Plega radicaudata

XML Treatment for Plega signata

XML Treatment for Plega sonorae

XML Treatment for Plega spinosa

XML Treatment for Plega stangei

XML Treatment for Plega vangiersbergenae

XML Treatment for Plega yucatanae

XML Treatment for Plega zikani

XML Treatment for Trichoscelia

XML Treatment for Trichoscelia anae

XML Treatment for Trichoscelia andina

XML Treatment for Trichoscelia banksi

XML Treatment for Trichoscelia basella

XML Treatment for Trichoscelia fenella

XML Treatment for Trichoscelia flavomaculata

XML Treatment for Trichoscelia geraldoi

XML Treatment for Trichoscelia gorgonensis

XML Treatment for Trichoscelia involuta

XML Treatment for Trichoscelia iridella

XML Treatment for Trichoscelia karijona

XML Treatment for Trichoscelia larizae

XML Treatment for Trichoscelia latifascia

XML Treatment for Trichoscelia nassonovi

XML Treatment for Trichoscelia pennyi

XML Treatment for Trichoscelia sequella

XML Treatment for Trichoscelia tobari

XML Treatment for Trichoscelia umbrata

XML Treatment for Trichoscelia varia

XML Treatment for Trichoscelia vespiformis

XML Treatment for Trichoscelia wintertoni

## ﻿﻿Additional information

### Author ORCIDs

Adrian Ardila-Camacho https://orcid.org/0000-0002-3750-8671

Renato José Pires Machado https://orcid.org/0000-0003-3155-3639

Michael Ohl https://orcid.org/0000-0002-3120-7915

Atilano Contreras-Ramos https://orcid.org/0000-0001-8044-1348

### Data availability

All of the data that support the findings of this study are available in the main text or Supplementary Information.

## Supplementary materials

﻿Supplementary material 1 Data matrix Data type winc https://binary.pensoft.net/file/1038367This dataset is made available under the Open Database License (http://opendatacommons.org/licenses/odbl/1.0/). The Open Database License (ODbL) is a license agreement intended to allow users to freely share, modify, and use this Dataset while maintaining this same freedom for others, provided that the original source and author(s) are credited.Adrian Ardila-Camacho, Renato José Pires Machado, Michael Ohl, Atilano Contreras-Ramos
